# Halogenated Rocaglate Derivatives: Pan-antiviral Agents against Hepatitis E Virus and Emerging Viruses

**DOI:** 10.1021/acs.jmedchem.3c01357

**Published:** 2023-12-21

**Authors:** Catherine Victoria, Göran Schulz, Mara Klöhn, Saskia Weber, Cora M. Holicki, Yannick Brüggemann, Miriam Becker, Gisa Gerold, Martin Eiden, Martin H. Groschup, Eike Steinmann, Andreas Kirschning

**Affiliations:** † Institute of Organic Chemistry, Leibniz University Hannover, Schneiderberg 1B, 30167 Hannover, Germany; ∇ Department of Molecular and Medical Virology, Ruhr-University Bochum, 44801 Bochum, Germany; § Federal Research Institute in Animal Health (FLI), Südufer 10, 17493 Greifswald, Insel Riems, Germany; ∥ Institute for Biochemistry and Research Center for Emerging Infections and Zoonoses (RIZ), University of Veterinary Medicine Hannover, Bünteweg 2, 30559 Hannover, Germany; ⊥ Wallenberg Centre for Molecular Medicine (WCMM), Umeå University, 901 87 Umeå, Sweden; # Department of Clinical Microbiology, Virology, Umeå University, 901 87 Umeå, Sweden

## Abstract

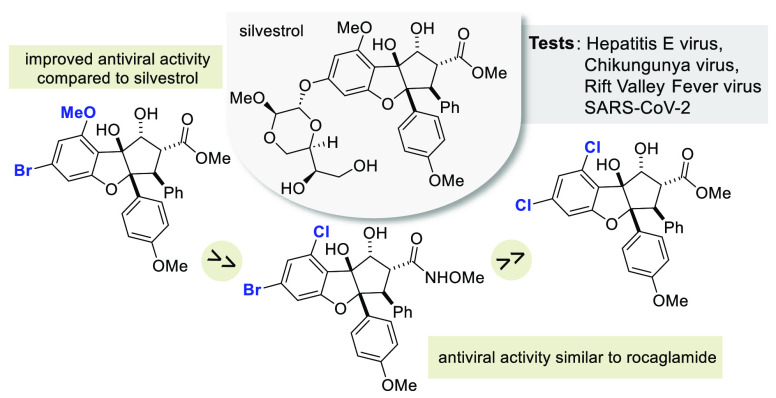

The synthesis of a library of halogenated rocaglate derivatives belonging to the flavagline class of natural products, of which silvestrol is the most prominent example, is reported. Their antiviral activity and cytotoxicity profile against a wide range of pathogenic viruses, including hepatitis E, Chikungunya, Rift Valley Fever virus and SARS-CoV-2, were determined. The incorporation of halogen substituents at positions 4′, 6 and 8 was shown to have a significant effect on the antiviral activity of rocaglates, some of which even showed enhanced activity compared to CR-31-B and silvestrol.

## Introduction

Rocaglates are natural products that belong to the flavaglines, a natural product class with more than 100 members to date.^1−3^ They are found in several tree species of the genus *Aglaia* (Meliaceae) that grow in subtropical and tropical forests of Southeast Asia, Northern Australia and the Pacific region.^4^

**Figure 1:**
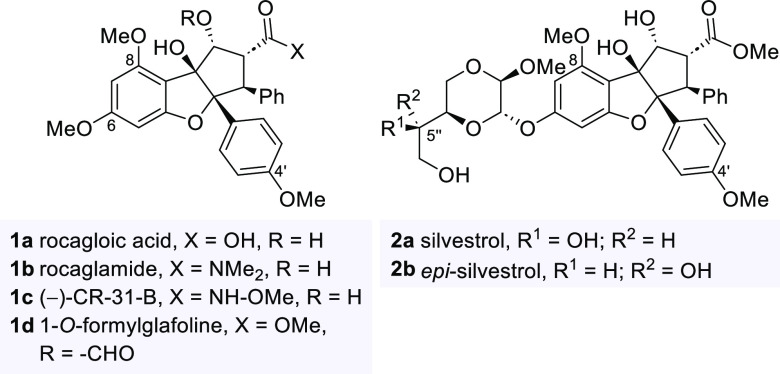
Structures of rocagloic acid (**1a**) and rocaglamide (**1b**), derivatives CR-31-B (**1c**) and 1-*O*-formylglafoline (**1d**) as well as silvestrol (**2a**) and its 5′-epimer (**2b**).

The first rocaglate extracts collected revealed significant activity against P-388 lymphatic leukemia in CDF1 mice and inhibitory activity *in vitro* against cells derived of human epidermoid carcinoma of the nasopharynx (κB cells). The antileukemic effect was attributed to the 1*H*-cyclopenta[*b*]benzofurans rocagloic acid (**1a**, Figure 1) and rocaglamide (**1b**).^5^ Later, antiviral properties against the Newcastle disease virus (NDV) were reported^6^ and the biological target of flavaglines was studied for the natural product silvestrol (**2a**) and 1-*O*-formylglafoline (**1d**). The excellent broadband antiviral activity of silvestrol (**2a**) was substantiated for highly pathogenic Ebola virus,^7^ as well as Zika virus, Hepatitis E virus (HEV) and viruses from the *Coronaviridae* and *Picornaviridae* family without pronouced cytotoxic effects for immortalized cell lines (Huh-7 and MRC-5).^8^ Translation initiation is a key process in viral proliferation. Because RNA viruses do not encode their own translational machinery, they rely on host protein synthesis. In the past, targeting the translation machinery of the host has been extensively studied and proposed as a therapeutic strategy for the treatment of viral infections. It is widely accepted that rocaglates exert their biological activity by stimulation of eIF4Af-RNA clamping.^9^ The eukaryotic initiation factor 4a (eIF4A) is an ATP-dependent RNA helicase, responsible for unwinding the secondary structure of mRNAs. Flavaglines force an engagement between eIF4A and RNA that prevents eIF4A from participating in the ribosome-recruitment step of translation. Recently, Iwasaki and co-workers resolved the structure of the human complex composed of eIF4A1, AMPPNP, rocaglamide **1b** and polypurine RNA, providing the molecular basis of rocaglamide RNA sequence selectivity. From these X-ray studies it was found that in particular the dimethoxy-substituted aromatic ring A in **1b** is directed toward the polypurine RNA. As such, ring A is stacked with the adenine base of A7 and guanine base of G8 nearly in parallel.^10^

Synthetic efforts had led to new rocaglate variants and derivative (−)-CR-31-B (**1c**) has to be noted as it was also found to inhibit the replication of Zika-, Lassa-, Crimean Congo hemorrhagic fever virus and *Coronaviridae* family members.^11−13^ It was precisely this promising biological potential of rocaglates that triggered synthetic programs culminating in the first total synthesis by Trost et al. in 1990^14^ and follow-up synthetic programs by the groups of Désaubry,^15−17^ Porco,^18,19^ Tremblay,^20^ Burns,^21^ Ishibashi^22^ and Reich^23^ that provided rocaglate-derived compound libraries.

The majority of these studies primarily focused on the substitution of the methoxy groups at C6 and C4′ and variation of the amide moiety. Both showed a profound effect on biological activity. Unsurprisingly, several halogenated rocaglates were also part of these libraries, as halogens are of great importance in medicinal chemistry. They give, in most cases, advantages to biophysical and -chemical properties of related compounds. Halogen substitution can enhance metabolic stability, lipophilicity and electronegativity. Moreover, introduction of halogen substituents can also provide halogen bonding (XB), which might lead to enhanced activity.^24−26^ In these preliminary studies, it was revealed that chlorine at C6 and a chlorine or bromine substituent at C4′ lead to a significant improvement in the inhibition of translation initiation.^14−16,20,27,28^ However, the possible impact of the small and highly electronegative fluorine atom as a substituent at C6 or C4′ is so far unknown. Furthermore, no derivatives halogenated at the C8 position have been reported to date.

Consequently, we initiated a program to synthesize and biologically evaluate a library of so far unknown halogenated rocaglate derivatives and tested them against several emerging RNA viruses, including HEV, Chikungunya (CHIKV), Rift Valley fever (RVFV) and SARS-CoV-2 viruses. As part of this program, we also aimed to identify the most practical synthetic route among several options for accessing the target derivatives.

## Results and Discussion

### General Considerations on the Syntheses

To date, the majority of rocaglate syntheses are based on a biomimetic approach starting from 3-hydroxyflavones (flavonol) and cinnamic acid derivatives, first described by Porco and co-workers in 2004.^29^ This process first involves UV light-mediated [3+2]-cycloaddition via an excited-state intramolecular proton transfer leading to the aglain core. Subsequently, skeletal rearrangements via a ketol shift and *anti*-selective reduction of the resulting ketone lead to the cyclopenta[*b*]benzofuran core present in the rocaglates. Excellent substrate selection and high diastereoselectivity for the establishment of the five stereocenters in only three steps are compelling reasons for the superiority of this route.

Surprisingly, synthetic access to the required 5,7,4′-substituted flavonols still poses a major challenge. In previous studies on flavaglines, the flavonols were most commonly prepared via an Algar–Flynn–Oyamada (AFO) reaction^14,22^ or alternatively a Baker–Venkataraman synthesis.^20,22,30^

**Scheme 1:**
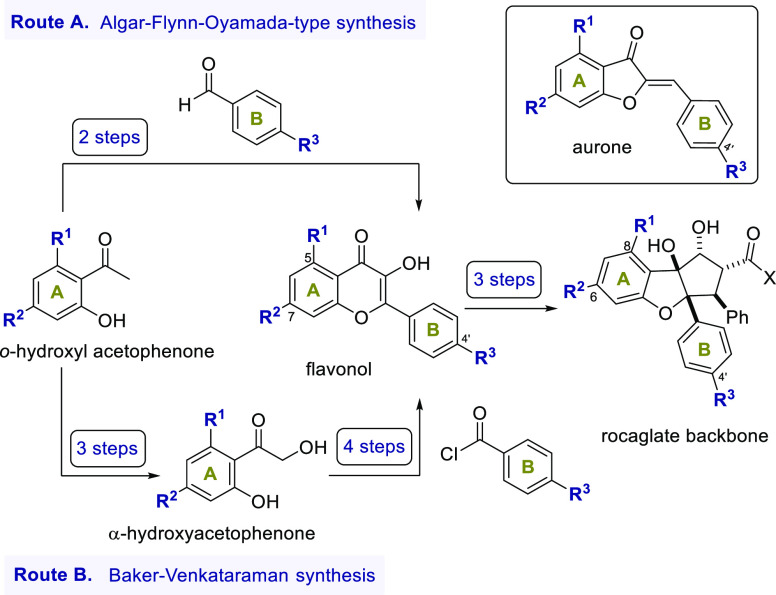
Synthetic Approaches to Rocaglate Derivatives with 5,7,4′-Substituted Flavonols as Key Intermediates and Structure of Aurones

The first route represents an oxidative cyclization of the corresponding chalcone with NaOH, KOH or K_2_CO_3_ in combination with hydrogen peroxide (Scheme 1, Route A). Although this biomimetic approach allows for rapid access to flavonols, its substrate scope is however rather restricted. In particular, electron-donating substituents at C5 and C7 or electron-withdrawing substituents at C4′ favor the formation of the corresponding aurone instead of the flavonoid.^31,32^ It should be noted, however, that in principle an alternative type of cyclization to the aurone skeleton is conceivable and possible.

The Baker–Venkataraman synthesis (Scheme 1, Route B)^20^ requires a larger number of steps but is supposedly more versatile with respect to substrate scope, as the different electronic properties of the substituents at C5 and C7 have little effect on the formation of flavonol.

The synthesis commenced from the corresponding *o*-hydroxyl acetophenones. A Rubottom oxidation sequence leads to the α-hydroxyacetophenones from which the bisbenzoates are formed by esterification. Depending on the desired substitution pattern on the B ring, various benzoic acid or benzoyl chloride derivatives can be used.^20,22^ Next, the sequence proceeds through a base-mediated Baker–Venkataraman rearrangement, followed by acid-catalyzed condensation and saponification of the enol ester that yields the flavonol. However, the aforementioned reaction sequence involves harsh basic and acidic conditions, which can limit the application of some protecting and functional groups.

### Synthesis of Rocaglates Based on the Baker–Venkataraman Rearrangement

**Scheme 2:**
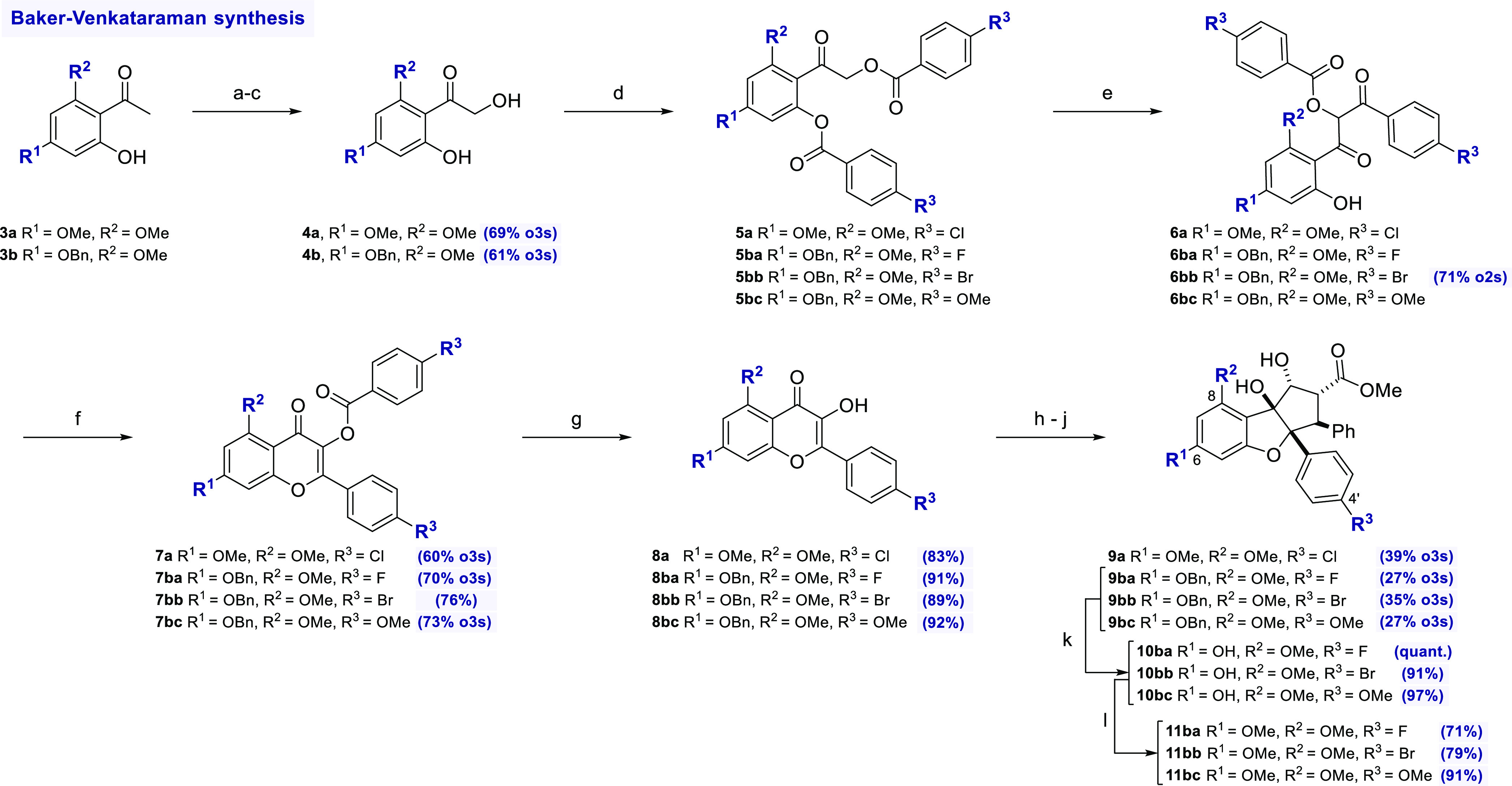
Synthesis of Rocaglates by the Baker–Venkataraman Route Reagents and conditions: a) TBSOTf, Et_3_N, CH_2_Cl_2_, 0 °C; b) *m*CPBA, NaHCO_3_, CH_2_Cl_2_, 0 °C to rt; c) *p*TsOH, THF/H_2_O, reflux; d) 4-DMAP, Et_3_N, CH_2_Cl_2_, rt; e) LiHMDS, THF, −20 °C; f) AcOH, H_2_SO_4_, rt (rt to 80 °C for R^3^ = Cl; 60 °C for R^1^ = OBn, R^3^ = Br, then BnBr, K_2_CO_3_, acetone, reflux); g) 5% NaOH, EtOH, 80 °C; h) *trans*-methyl cinnamate, CHCl_3_/TFE 7:3, UV light (365 nm), – 5 °C; (i) NaOMe, MeOH, reflux; j) Me_4_NBH(OAc)_3_, AcOH, MeCN, rt; k) Pd/C, H_2_, THF, rt; l) TMSCHN_2_, PhMe/MeOH, rt. Abbreviations: TBS = *t*-butyldimethylsilyl, *m*CPBA = *meta*-chloroperbenzoic acid, *p*TsOH = *para*-toluenesulfonic acid, 4-DMAP = 4-dimethyaminopyridine, LiHMDS = lithium hexamethyldisilazide, Ac = acyl, Bn = benzyl, TFE = 2,2,2-trifluoroethanol, TMS = trimethylsilyl.

To investigate the influence of halogen substituents at C4′, we resorted to the Baker–Venkataraman route, since the electron-withdrawing effect of fluorine, chlorine and bromine in the AFO reaction strongly favors the formation of aurone. Based on studies by Tremblay et al.,^20^ we established a reliable, high-yielding and scalable linear route (Scheme 2) where acetophenones **3a** and **3b** served as starting materials (see the Supporting Information).

Rubottom oxidation and formation of the α-hydroxyacetophenones **4**, followed by double esterification with various 4-substituted benzoyl chlorides, furnished precursors **5** that are required for the Baker–Venkataraman rearrangement, consistently in excellent yields. In the presence of LiHMDS as a base, the anionic rearrangement led to the phenol **6**. Next, a ring-closing condensation reaction led to the formation of flavonol esters **7**. We found that elevated temperatures were required for substrates with chlorine or bromine substitution at C4′, while complete conversion was already observed at room temperature (rt) for substrates that bear a methoxy or fluorine substituent at this position. Subsequent saponification with sodium hydroxide gave the corresponding flavonols **8a**–**bc** in excellent yields.^33^

As mentioned before, these harsh acidic/basic reaction conditions were accompanied by several limitations. Incorporation of acid-labile protecting groups like MOM on the phenol functionality, as well as flavonols with sensitive structural modifications on the B-ring such as the pyridine ring as well as electron-withdrawing groups such as 4-nitrobenzene, is not feasible.

With the flavonols in hand, using methyl cinnamate, the synthesis proceeded with a UV light-mediated [3+2]-cycloaddition, followed by a ketol shift and finally diastereoselective reduction of the ketone according to the protocol of Rizzacassa et al.^34^ Methyl rocaglates **9a**–**bc** were obtained in good yields. In the cases where a benzyloxy group was installed at C6, we were able to convert it to the corresponding methoxy ethers **11ba**–**bc** via deprotection with H_2_, Pd/C and methylation with trimethylsilyldiazomethane.^20^

### Flavonol Synthesis Based on Algar–Flynn–Oyamada-Type Reactions

**Scheme 3:**
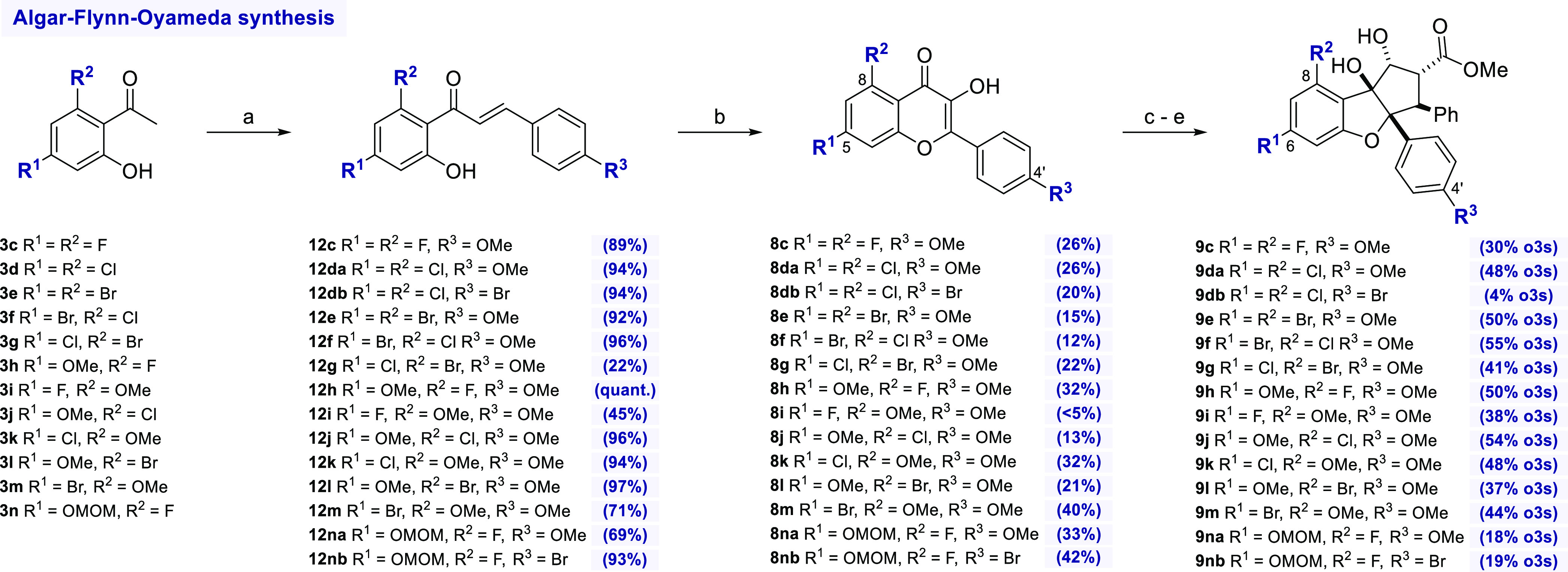
Synthesis of Rocaglates by the Algar–Flynn–Oyamada Route Reagents and conditions: a) 4-R^3^-C_6_H_4_CO_2_H, NaOEt, EtOH, rt; b) NaOH (aq.), H_2_O_2_, MeOH, 0 °C to rt; c) *trans*-methyl cinnamate, CHCl_3_/TFE 7:3, UV light (365 nm), −5 °C; d) MeONa, MeOH, reflux; e) NMe_4_B(OAc)_3_H, AcOH, MeCN, rt.

Next, we turned our attention toward the modification of the C6 and C8 positions of rocaglates. As mentioned above, the AFO synthesis is a promising approach for the synthesis of flavonols that possess an electron-withdrawing substituent at C5 and C7 (corresponding to C6 and C8 in the corresponding rocaglate) and an electron-withdrawing substituent at C4′. Accordingly, we prepared a series of new halogenated rocaglates via the route depicted in Scheme 3. The acetophenones **3c**–**i** and **3n** were prepared from their respective 3,5-substituted phenols by acetylation followed by Fries rearrangement, whereas **3j**–**m** were synthesized from their respective 3,5-dimethoxy halobenzenes by acylation and mono-demethylation (see Supporting Information).

According to a procedure by Sale et al.,^35^ the acetophenones could be easily converted into chalcones **12c**–**n** in the presence of sodium ethoxide as a base. The subsequent AFO reaction using a mixture of NaOH and H_2_O_2_ gave the desired flavonols **8c**–**n** in acceptable yields. Remarkably, this protocol also allowed the synthesis of flavonols **8db** and **8nb** bearing electron-withdrawing substituents at the C4′ position. However, in these cases, significant proportions of corresponding aurones (see Scheme 1) were also formed. Analogous to flavonols **8a**–**bc** prepared via the Baker–Venkataraman route, compounds **8c**–**n** were converted to rocaglate derivatives **9c**–**n** using the established sequence. With the exception of the 4′-bromo rocaglates **9db** and **9nb**, yields of about 50% over three steps were obtained for the major *endo*-diastereomer.

### Conversion of Rocaglate Methyl Esters to the Corresponding Amides

**Scheme 4:**
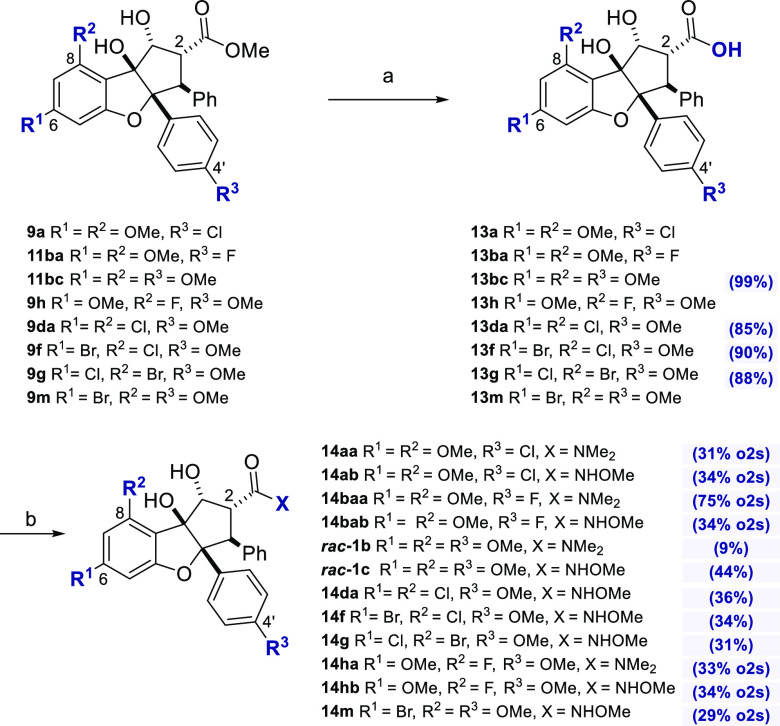
Transformation of Selected Methyl Esters to the Corresponding *N,N*-Dimethyl and *N*-Methoxymethyl Amides Reagents and conditions: a) LiOH, MeOH, 45 °C. b) HNMe_2_·HCl or MeONH·HCl, EDC·HCl, HOBt·H_2_O, *i*Pr_2_NEt, CH_2_Cl_2_, rt or for *rac*-**1b** HNMe_2_·HCl, Et_3_N, 4-DMAP, EDC·HCl, DMF, 0 °C → rt. EDC = 1-ethyl-3-(3-dimethylaminopropyl)carbodiimide.

Starting from the new rocaglate methyl esters, selected members of this library were converted into amides (Scheme 4). It was previously demonstrated that the incorporation of both an *N,N*-dimethylamide and an *N*-methoxyamide group can result in significantly improved antiviral activity.^14,23^

### Biological Studies

In total, we prepared 33 rocaglates as racemic mixtures via two different routes, with 30 of the derivatives containing one or more halogen atoms. Since it is known from previous work that the presence of a benzyloxy group at position 6 leads to decreased translational inhibition,^21^ compounds **9ba**, **9bb** and **9bc** were excluded from the study of antiviral activity. In addition to the resynthesized (±)-rocaglamide (*rac*-**1b**), (±)-CR-31-B (*rac*-**1c**) and (±)-methylrocaglate (**11bc**), commercial (−)-silvestrol (**2a**) also served as a reference compound.

**Figure 2:**
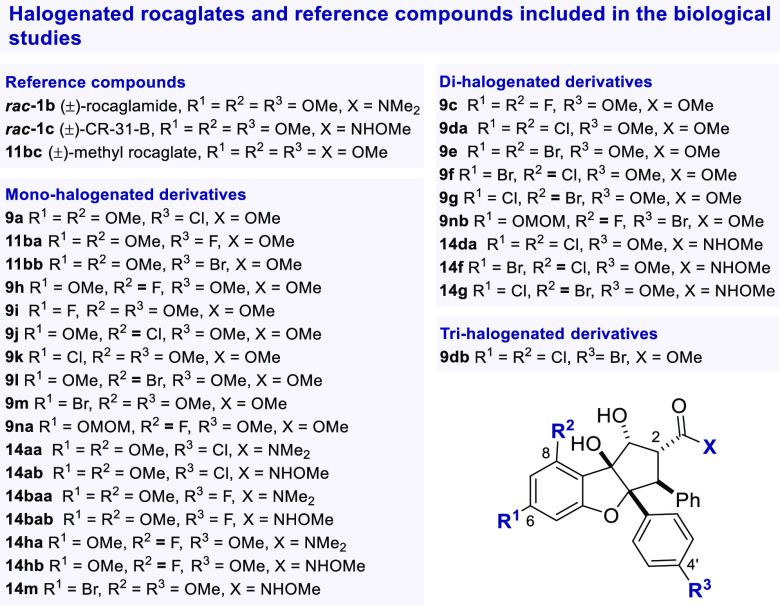
Synthesized rocaglates selected for biological evaluation.

**Figure 3:**
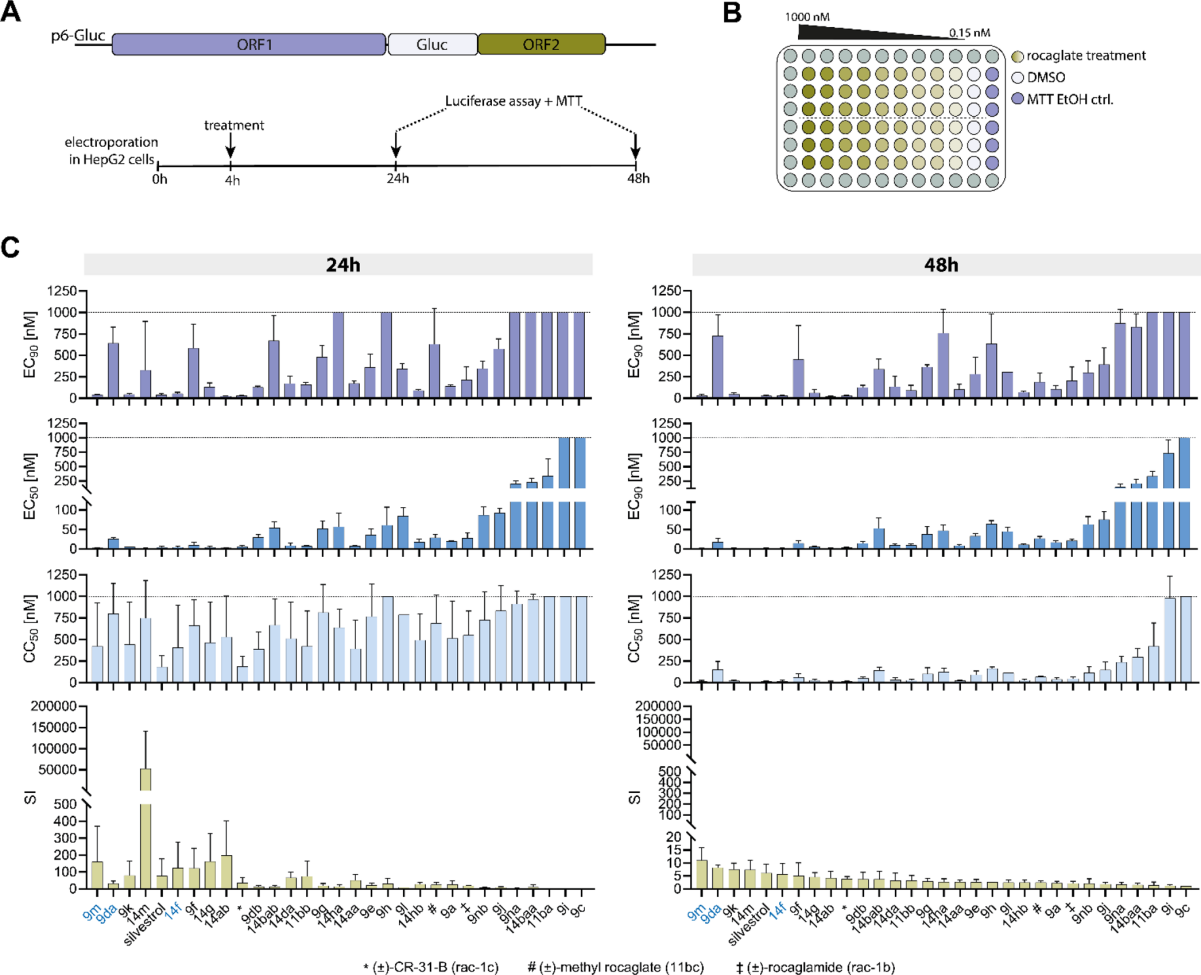
Antiviral efficacy of halogenated rocaglates against HEV. A) Schematic representation of assay setup. B) Plate layout for *in vitro* testing. C) EC_90_, EC_50_, CC_50_ and SI values derived from dose–response curves at 24 and 48 h post-electroporation.

Hepatitis E viruses are characterized by a highly structured 5′ untranslated region (5′ UTR) and rely on cap-dependent translation for their efficient replication.^36^ Herein, we assessed structure–activity relationships of our new halogenated rocaglates and their potential as antiviral agents against HEV replication by transfecting hepatoma cells (HepG2) with the HEV-3 replicon p6-Gluc and treating these cells with the compounds listed in Figure 2 in concentrations ranging from 0.15 to 1000 nM (Figure 3A,B). Luciferase activity and MTT assays were conducted to measure HEV RNA replication and cell viability, respectively. The obtained EC_50_, EC_90_, CC_50_ and selectivity index (SI) values are summarized in Figure 3C and Table 1.

**Table 1:** Overview of Halogenated Rocaglates Synthesized in the Present Work and Their Corresponding Efficacy against HEV at 24 and 48 h^a^

					24 h (in nM)				48 h (in nM)			
compd	R^1^	R^2^	R^3^	X	EC_50_	EC_90_	CC_50_	SI	EC_50_	EC_90_	CC_50_	SI
**9m**	Br	OMe	OMe	OMe	3.1	40.7	420.9	160.4	1.4	30.6	13.8	11.0
**9da**	Cl	Cl	OMe	OMe	25.4	642.3	797.7	31.7	17.5	725.3	148.9	8.2
**9k**	Cl	OMe	OMe	OMe	5.4	45.1	442.9	79.3	2.5	45.5	19.9	7.5
**14m**	Br	OMe	OMe	NHOMe	1	328.3	748.2	52782.3	0.2	1.6	1	7.4
(−)-silvestrol (**2a**)	dioxanyloxy	OMe	OMe	OMe	4.2	40.5	180.3	76.7	2.2	28.1	13.4	6.1
**14f**	Br	Cl	OMe	NHOMe	4.6	53.4	407.1	123.2	2.6	30.2	15.6	5.7
**9f**	Br	Cl	OMe	OMe	9.9	582.8	663.4	122.2	14.9	453.5	61.0	5.1
**14g**	Cl	Br	OMe	NHOMe	4.1	131.1	464.2	162.7	5.5	62.9	26.3	4.6
**14ab**	OMe	OMe	Cl	NHOMe	2.8	23.8	528.8	197.9	2.3	18.2	10.0	4.2
(±)-CR-31-B (*rac***-1c**)	OMe	OMe	OMe	NHOMe	6.1	29.0	188.3	35.4	3.7	27.3	14.3	3.8
**9db**	Cl	Cl	Br	OMe	29.6	128.0	388.8	13.4	14.7	123.9	48.0	3.7
**14bab**	OMe	OMe	F	NHOMe	53.7	670.1	666.0	13.5	52.9	338.2	142.9	3.7
**14da**	Cl	Cl	OMe	NHOMe	8.6	172.5	510.7	65.9	9.8	134.5	31.8	3.3
**11bb**	OMe	OMe	Br	OMe	7.3	157.7	420.5	74.1	9.4	91.3	27.1	3.2
**9g**	Cl	Br	OMe	OMe	52.1	481.5	812.5	19.0	37.9	364.6	101.0	2.8
**14ha**	OMe	F	OMe	NMe_2_	56.9	>1000	637.6	14.3	47.1	758.7	120.3	2.7
**14aa**	OMe	OMe	Cl	NMe_2_	7.2	174.5	390.3	50.8	8.9	101.6	22.7	2.7
**9e**	Br	Br	OMe	OMe	36.1	361.5	765.6	22.1	33.2	282.4	90.2	2.6
**9h**	OMe	F	OMe	OMe	61.0	>1000	>1000	30.4	64.3	633.7	163.2	2.5
**9l**	OMe	Br	OMe	OMe	83.6	343.2	787.1	9.7	44.7	304.7	113.9	2.5
**14hb**	OMe	F	OMe	NHOMe	17.2	91.2	491.2	28.5	10.9	69.8	27.5	2.5
(±)-methyl rocaglate (**11bc**)	OMe	OMe	OMe	OMe	28.4	630.9	689.8	24.4	29.9	187.8	65.3	2.5
**9a**	OMe	OMe	Cl	OMe	19.4	139.8	512.6	26.4	16.8	105.4	38.8	2.2
(±)-rocaglamide (*rac***-1b**)	OMe	OMe	OMe	NMe_2_	27.8	213.1	549.5	19.7	21.0	201.3	44.5	2.1
**9nb**	OMOM	F	Br	OMe	86.9	345.4	727.1	8.1	63.2	298.0	113.3	2.1
**9j**	OMe	Cl	OMe	OMe	92.6	576.6	832.1	9.3	75.7	393.5	147.8	1.8
**9na**	OMOM	F	OMe	OMe	202.2	>1000	911.8	4.6	146.1	873.3	235.6	1.8
**14baa**	OMe	OMe	F	NMe_2_	228.6	>1000	965.7	4.4	208.2	828.8	296.9	1.5
**11ba**	OMe	OMe	F	OMe	338.4	>1000	>1000	10.9	334.3	>1000	421.4	1.4
**9i**	F	OMe	OMe	OMe	>1000	>1000	>1000	1.0	731.7	>1000	904.5	1.3
**9c**	F	F	OMe	OMe	>1000	>1000	>1000	1.0	>1000	>1000	>1000	1.0

a Designations of R^1^–R^3^ and X Are Presented in Figure 2. SI values represent mean SI values calculated from three biological replicates and therefore do not necessarily represent the ratio between EC_50_ and CC_50_ values listed in the table.

In accordance with previous findings for non-halogenated compounds,^14,23^ an example of chlorinated C2-methyl ester **9a** (EC_90_ = 105.4 nM) showed to be inferior in potency compared to its corresponding dimethylamide **14aa** (EC_90_ = 101.6 nM) and its methoxyamide **14ab** (EC_90_ = 18.2 nM). To further support this outcome, the same series of derivatives with fluorine instead of chlorine were tested. The result proved to be similar, with methoxyamide as the most potent member (**14bab**, EC_90_ = 338.2 nM) compared to its dimethylamide **14baa** (EC_90_ = 828.8 nM) and ester **11ba** (EC_90_ > 1000 nM) (X = NHOMe > NMe_2_ > OMe), respectively.

The observed improvement in EC_90_ values for amides may be attributed by the fact that carbonyl groups of the amide serve as better hydrogen bond donors to Gln195 of eIF4A compared to methyl esters.^17,18^ Notably, enhanced inhibition of HEV replication was observed in the C4′-bromo methyl ester **11bb** (EC_90_ = 91.3 nM) compared to C4′-chlorine **9a** (EC_90_ = 105.4 nM) and C4′-fluorine methyl ester **11ba** (EC_90_ > 1000 nM). Moreover, **9a** and amide derivatives **14aa** (EC_90_ = 101.6 nM) and **14ab** (EC_90_ = 18.2 nM) displayed superior HEV inhibition compared to C4′-methoxy substituents (*rac***-1b**, *rac***-1c** and **11bc**). In contrast, fluorine functionalization in **11ba**, **14baa** and **14bab** at position C4′ resulted in decreased activity and cytotoxicity for methyl esters, *N*-dimethylamides and *N*-methoxyamides relative to C4′-methoxy derivatives (compare **14bab** [EC_90_ = 338.2 nM; CC_50_ = 142.9 nM] with *rac*-**1c** [EC_90_ = 27.3 nM, CC_50_ = 14.3 nM], **14baa** [EC_90_ = 828.8 nM, CC_50_ = 296.9 nM] with *rac*-**1b** [EC_90_ = 201.3 nM; CC_50_ = 44.5 nM] and **11ba** [EC_90_ > 1000 nM; CC_50_ = 421.4 nM] with **11bc** [EC_90_ = 187.8 nM; CC_50_ = 65.3 nM]). These observations corresponded to the EC_90_ trends Br > Cl > OMe > F and Cl > OMe > F for methyl esters and carbonyl amides, respectively. To further elucidate the influence of halogen functionalization, we examined halogenated rocaglates substituted with Br, Cl and F at positions 6 and 8, or both, concerning their antiviral activity against HEV replication. The C8-bromo methyl ester **9l** (EC_90_ = 304.7 nM) displayed marginally reduced activity compared to compound **9e** (EC_90_ = 282.4 nM) (C8, C6-bromine substitution). Conversely, the introduction of a bromine atom solely at position C6 in **9m** (EC_90_ = 30.6 nM; CC_50_ = 13.8 nM) significantly enhanced both activity and cytotoxicity. A similar trend was observed for chlorine-substituted derivatives (compare **9j** [EC_90_ = 393.5 nM] with **9da** [EC_90_ = 725.3 nM] and **9k** [EC_90_ = 45.5 nM]). However, C6- and C8-bromine substitutions generally produced more active compounds than their C6- and C8-chlorine counterparts. Also, addition of a bromine atom (position C4) to an already halogenated derivative enhanced activity (compare **9na** [EC_90_ = 873.3 nM] with **9nb** [EC_90_ = 298.0 nM] or **9da** [EC_90_ = 725.3 nM] with **9db** [EC_90_ = 123.9 nM]).

Fluorine functionalization at position C8 in carbonyl amides **14ha** (EC_90_ = 758.7 nM) and **14hb** (EC_90_ = 69.8 nM) led to reduced activity compared to non-halogenated amides *rac***-1b** and *rac***-1c**. Intriguingly, the introduction of a fluorine moiety at position C6 in **9c** (EC_90_ > 1000 nM) and **9i** (EC_90_ > 1000 nM) completely diminished antiviral activity in hepatoma cells.

Collectively, these findings demonstrate that bromine functionalization yielded the most significant improvement of activities when substituted at position C6 (C6 > C4′ > C8), while chlorine substitutions led to the most potent increase in activity for position C4′ (C4′ > C8). Conversely, fluorine functionalization at C4′ and C8 resulted in reduced antiviral activity and cytotoxicity and entirely abrogated activity when introduced at the C6 position (C8 > C4′ > C6). Based on calculated SI values, two additional trends were observed. First, substitutions on ring A (position C6 and C8) tend to result in improved SI values compared to C4′ or C2 substitutions. Also, derivatives with improved activity were observed to have better SI values than less potent derivatives.

**Figure 4:**
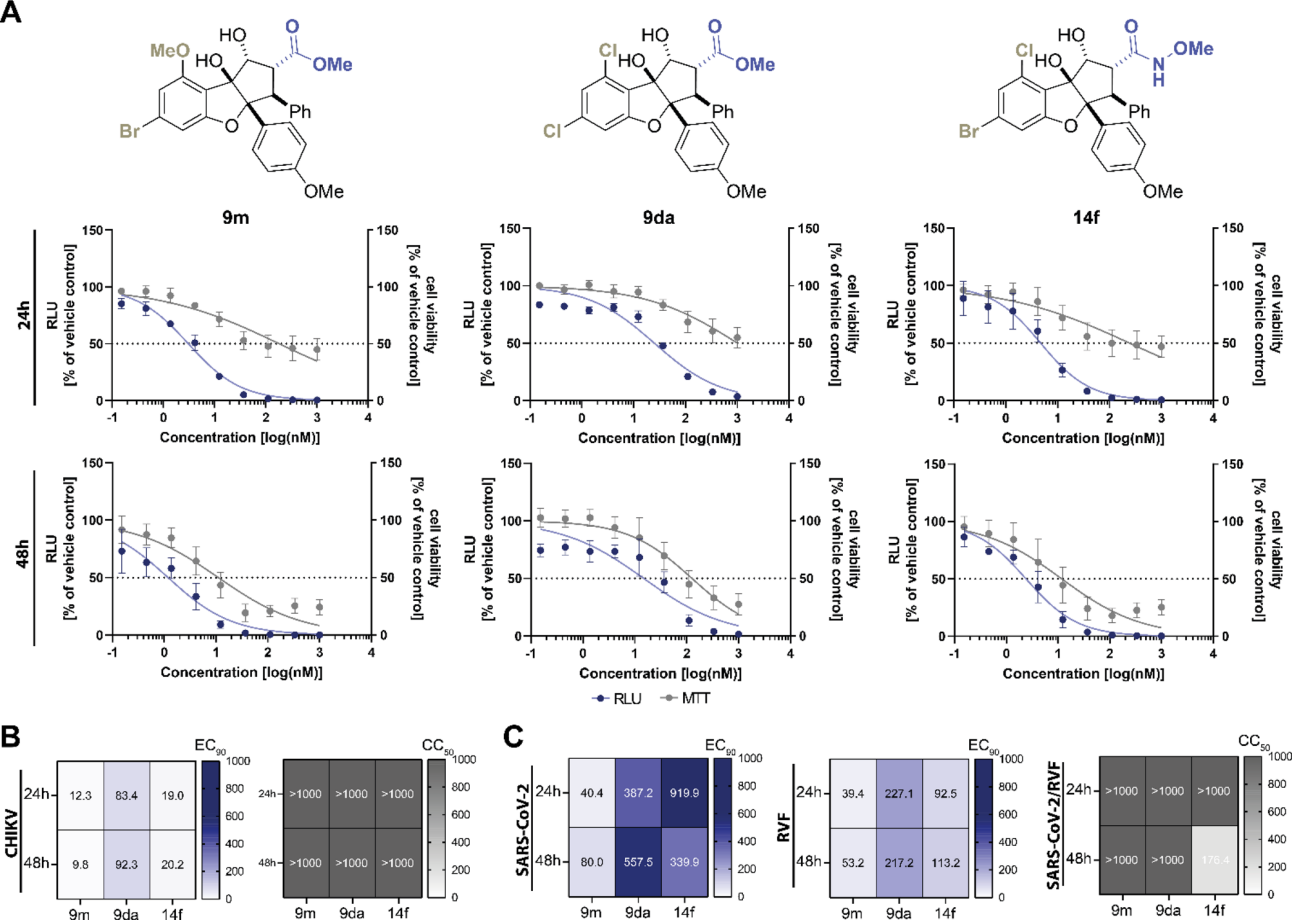
Pan-antiviral inhibition of HEV, Chikungunya virus (CHIKV), Rift Valley fever virus (RVFV) and SARS-CoV-2 replication by **9m**, **9da** and **14f**. A) HEV subgenomic replicon HEVp6-Gluc was electroporated into HepG2 cells. Cells were treated with **9m**, **9da** and **14f** at concentrations ranging from 0.15 nM to 1000 nM for 24 and 48 h. Depicted are nonlinear fit response curves representative of three biological replicates for HEVp6-Gluc (dark blue lines), and cell viability was monitored by MTT assay (gray lines). Error bars indicate standard deviation, *n* = 3. B) Huh-7 cells were treated with different concentrations (0.15 nM to 1000 nM) of **9m**, **9da** and **14f** and infected at a MOI of 2.5 with infectious clone CHIKV LR2006-OPY1 expressing GFP under the control of a subgenomic promotor. GFP expression as measure of infection (left panel) and cell viability (right panel) were measured by live cell imaging and MTT assay, respectively. C) Vero-E6 cells were infected with SARS-CoV-2 or RVF strain MP-12 at a multiplicity of infection (MOI) of 0.1. Supernatants were collected at 24 h post infection (hpi) or 48 hpi and subjected to RT-qPCR analysis as measure of infection (left and middle panel). Cell viability was determined by MTT assay (right panel).

Based on selectivity indices calculated for 48-h treated compounds, we identified **9m** and **9da** as the most promising rocaglates in our investigation (Figure 3A). Consequently, we evaluated the antiviral efficacy of **9da** and **9m** against CHIKV, RVF and SARS-CoV-2. Derivative **9k** and **14m** were not included, due to high structural similarity of **9k** to **9m** and high toxicity observed for **14m** at 48 h. Therefore, we also selected derivative **14f** for further analysis. The C6, C8-chloro-functionalized methyl ester **9da** proved to be the least active derivative for all tested viruses (Figure 4A–C, Table 2). *N*-methoxyamide **14f** exhibited less activity than the C6-bromo-functionalized **9m** for Chikungunya virus (CHIKV ) [EC_90_ = 20.2 nM vs EC_90_ = 9.8 nM], Rift Valley fever virus (RVFV) [EC_90_ = 113.2 nM vs EC_90_ = 53.2 nM] and SARS-CoV-2 [EC_90_ = 339.9 nM vs EC_90_ = 80.0 nM], while **14f** and **9m** showed similar activity against HEV. Finally, we evaluated the influence of the cell density on the antiviral activity of exemplified for **9m** by comparing the standard protocol cell density to that of a confluent monolayer. As depicted in Figure S1, cell viability improved when cell density was higher. However, at the same time the antiviral response of **9m** decreased, which is likely due to the greater number of cells replicating the HEV genome, necessitating a higher dose of the drug to achieve the same reduction of replication (Figure S1).

**Table 2:** Overview of Halogenated Rocaglates Synthesized in the Present Work and Their Corresponding Efficacy against Chikungunya Virus (CHIKV), SARS-CoV-2 and Rift Valley Fever Virus (RVFV) at 24 and 48 h, Respectively^a^

					24 h (in nM)				48 h (in nM)			
compound	R^1^	R^2^	R^3^	X	EC_50_	EC_90_	CC_50_	SI	EC_50_	EC_90_	CC_50_	SI
**CHIKV**												
**9m**	Br	OMe	OMe	OMe	2.1	12.3	>1000	637.2	2.9	9.8	>1000	428.1
**9da**	Cl	Cl	OMe	OMe	18.1	83.4	>1000	57.5	22.4	92.3	>1000	44.9
**14f**	Br	Cl	OMe	NHOMe	3.1	19.0	>1000	402.1	3.4	20.2	>1000	317.4

**SARS-CoV-2**												
**9m**	Br	OMe	OMe	OMe	33.9	40.39	>1000	29.5	47.8	80.0	>1000	20.9
**9da**	Cl	Cl	OMe	OMe	110.8	387.2	>1000	9.0	176.5	557.5	>1000	5.7
**14f**	Br	Cl	OMe	NHOMe	51.4	919.9	>1000	19.5	189.5	339.9	176.4	0.9

**RVFV**												
**9m**	Br	OMe	OMe	OMe	26.6	39.4	>1000	38.4	43.32	53.2	>1000	23.1
**9da**	Cl	Cl	OMe	OMe	101.7	227.1	>1000	9.8	142.6	217.2	>1000	7.0
**14f**	Br	Cl	OMe	NHOMe	42.1	92.51	>1000	23.8	106.4	113.2	176.4	1.7

a Designations of R^1^–R^3^ and X are presented in Figure 2.

## Conclusion

**Figure 5:**
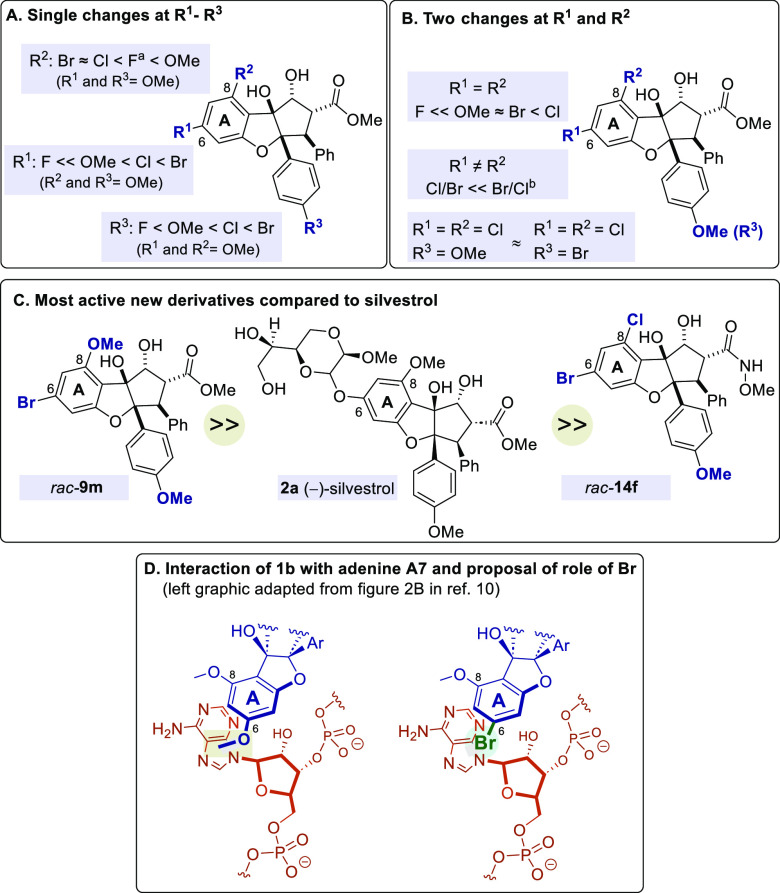
Short summary of SAR analysis and proposed interaction of bromine substituent at C6 of ring A with the polypurine chain. ^*a*^–C(O)NMe_2_ and −C(O)NHOMe instead of −CO_2_Me ester. ^*b*^–C(O)NHOMe shows improved activity over −CO_2_Me ester.

One of the most promising targets for inhibition of viral protein synthesis is the eukaryotic initiation factor (eIF) 4F complex (comprised of eIF4A, 4E and 4G). Due to a highly structured viral 5′-untranslated region (5′UTR), a large number of RNA viruses require the DEAD-box RNA helicase activity of eIF4A to unwind the viral genome and to allow for the recruitment and scanning of the 43*S*-pre-initiation complexes (43*S*-PIC) during translation initiation.^37^ Intriguingly, several previous studies have reported that inhibition of the eIF4A complex by rocaglates could prevent replication of different RNA viruses *in vitro* and *in vivo.*(38) In this study, a library of 27 halogenated derivatives of rocaglamide was synthesized via two different synthetic routes. Subsequent biological evaluation of the modified rocaglate derivatives revealed an potential antiviral effect on hepatitis E (HEV) and moderate antiviral activities against Chikungunya (CHIKV), Rift Valley river virus (RVFV) and SARS-CoV-2 viruses. In addition, the compounds exerted some cytostatic effects, which was reflected by the low to moderate SI values. The biological tests revealed various structure–activity findings about the rocaglates, especially with regard to positions 4′, 6 and 8 (Figure 5A–C). For the 4′ position, an increase in activity of F < OMe < Cl < Br was found. The bromine derivative is thus more active than the rocaglate with the methoxy group found in the natural products. The fluorine derivative, on the other hand, exerts hardly any antiviral activity. For the 6 position the trend is as follows. Here fluorine leads to complete loss of antiviral activity followed by OMe < Cl < Br. Finally, the replacement of the methoxy group in position 8 gave the following relationship: Br ∼ Cl < F < OMe. Replacing the methoxy groups at positions 6 and 8 with two identical substituents results in the following picture: F ≪ MeO ∼ Br < Cl. The antiviral activity of the dichloro derivative **9da** is further enhanced when the methoxy group at C4′ is replaced by bromine, as in rocaglate **9db**. Finally, it was found that the best halogen combination at positions 6 and 8 is bromine at C6 and chlorine at C8 in rocaglate derivative **9f**.

It is remarkable that the medicinal-chemically relevant halogen fluorine shows a negative influence on the antiviral properties of rocaglates, at least in particular at positions 4′ and 6, less so at position 8.

Another trend worth mentioning is the fact that substitutions at the A ring (C6, C8) lead overall to better SI values in terms of activity than modifications at C4′ or at C2 (ester to amide). In general, more antiviral active derivatives show on average a better SI value than derivatives with lower activity.

This study contributes to the elucidation of new structure–activity relationship for a series of antiviral compounds targeting a panel of human pathogenic viruses. We identified compounds **9m** and **14f**, which are all more potent than the natural product (±)-rocaglamide (*rac***-1b**) and similarly potent as (−)-silvestrol (**2a**), as potential candidates for further studies. The cytotoxicity of these compounds is comparatively low warranting further explorations. Finally, one may speculate about the special effect of halogen substitution presented in this work. The report by Iwasaki and co-workers^10^ on the resolved structure of the human complex composed of eIF4A1, AMPPNP, rocaglamide **1b** and polypurine RNA provides insight into this matter, because ring A in **1b**, that we modified with halogen substituents, is directed toward the polypurine RNA, specifically the adenine base of A7 and guanine base of G8. Halogen bonding,^39^ which resembles the electron density donation-based weak interaction of halogens with Lewis bases, including nucleobases,^40^ may provide a rationale for the observations reported here. A telling example is clindamycin, a halogenated ribosome binder that binds into the 50S subunit.^41^ It contains one chlorine atom that is directed toward the sugar edge of guanosine and forms an interaction with the guanine nitrogen atom.^40^

Particularly, the introduction of bromine at position 6 in ring A leads to improved antiviral properties and this may be associated with halogen bonding toward the adenine base of A7 and guanine base at G8 (Figure 5D). In the future, structural biology studies should provide a deeper understanding of the halogen effect observed here.

## Experimental Section

### Chemical Synthesis: General Methods

All experiments involving water-sensitive compounds were carried out in dried glassware under argon or nitrogen. Anhydrous solvents (MeCN, CH_2_Cl_2_, Et_2_O, PhMe) were obtained from a M. Braun MB solvent purification system or commercial solvents were used as supplied. Petroleum ether and dichloromethane were distilled before application and triethylamine was dried over KOH and distilled as well. Commercial reagents were used as supplied. Thin-layer chromatography (TLC) was performed on aluminum-backed plates precoated (0.25 mm) with silica gel 60 F254 with a suitable solvent system and was visualized using UV fluorescence and/or developed with KMnO_4_, anisaldehyde or vanillin stain followed by brief heating. For column chromatography, silica gel (35–70 μm) was used. Alternatively, a Biotage SP purification system was used. Biotage silica cartridges were used as supplied. All compounds are >95% pure. The purity of tested compounds was determined by analytical liquid chromatography of solutions of the compounds in DMSO-*d*_6_. Waters Alliance 2695 LC with a Waters Acquity 2996 photodiode array detector equipped with a Varian Polaris C18-A column (5.0 μm, 50 mm × 2.0 mm). The mobile phases were (A) 0.1% formic acid in water and (B) 0.1% formic acid in acetonitrile. After injection the gradient holds were at A/B (90%/10%) for 1.00 min followed by a gradient to A/B (0%/100%) over 1.75 min, a 0.05 min flush at 0%/100% (A/B) and a 1.20 min re-equilibration at A/B (90%/10%) at a flow rate of 0.8 mL/min and a column temperature of 45 °C. ^1^H NMR spectra are represented as follows: chemical shift, multiplicity (s = singlet, d = doublet, t = triplet, q = quartet, qi = quintet, sx = sextet, sp = septet, bs = broad singlet, m = multiplet), coupling constant (*J*) in hertz (Hz), integration and assignment. ^13^C NMR spectra are represented as follows: chemical shift, substitution (p = primary, s = secondary, t = tertiary, q = quaternary) and assignment. ^19^F NMR spectra are represented as follows: multiplicity (s = singlet, d = doublet, t = triplet, q = quartet, qi = quintet, sx = sextet, sp = septet, bs = broad singlet, m = multiplet), coupling constant (*J*) in hertz (Hz), integration and assignment. The numbering of the carbon and hydrogen atoms of the rocaglates synthesized follows the IUPAC nomenclature. A list of all rocaglates including the numbering of the carbon and hydrogen atoms is provided in the Supporting Information. ^1^H NMR, ^13^C NMR and ^19^F NMR spectra were recorded using a Bruker Ultrashield 500 MHz with Avance-III HD console, a Bruker Ascend 400 MHz with Avance-III console, a Bruker Ascend 400 MHz with Avance-III HD console, a Bruker Ultrashield 400 MHz with Avance-I console and a Bruker Ascend 600 MHz with Avance Neo console. High-resolution mass spectrometry (HRMS) data was measured with a Micromass LCT with lockspray source. The injection proceeded in loop-mode with a HPLC system by Waters (Alliance 2695). Alternatively, mass spectra were recorded with an Acquity-UPLC system by Waters in combination with a Q-Tof Premier mass spectrometer by Waters in lockspray mode. The ionization happened by electrospray ionization (ESI) or by chemical ionization at atmospheric pressure (APCI). The calculated and found mass are reported. GC/MS analyses were carried out with an HP 6890 chromatograph with KAS 4, coupled to an HP 5973 quadrupole mass selective detector. Samples were analyzed on an Optima 5 column (poly(5% phenyl–95% methylsiloxane), 30 m × 0.32 mm i.d. × film thickness 0.25 μm). Carrier gas, He; injector temp., 60 to 300 °C at 12 °C/min, splitless; temp. program: 50 °C (isothermal 1 min) to 300 °C, at 20 °C/min and held isothermal for 6 min at 300 °C; ion source: EI, ionization energy, 70 eV; electron mass spectra were acquired over the mass range of 40–500 amu.

### Synthesis of (±)-Methyl (1*R*,2*R*,3*S*,3a*R*,8b*S*)-3a-(4-chlorophenyl)-1,8b-dihydroxy-6,8-dimethoxy-3-phenyl-2,3,3a,8b-tetrahydro-1*H*-cyclopenta[*b*]benzofuran-2-carboxylate (**9a**)

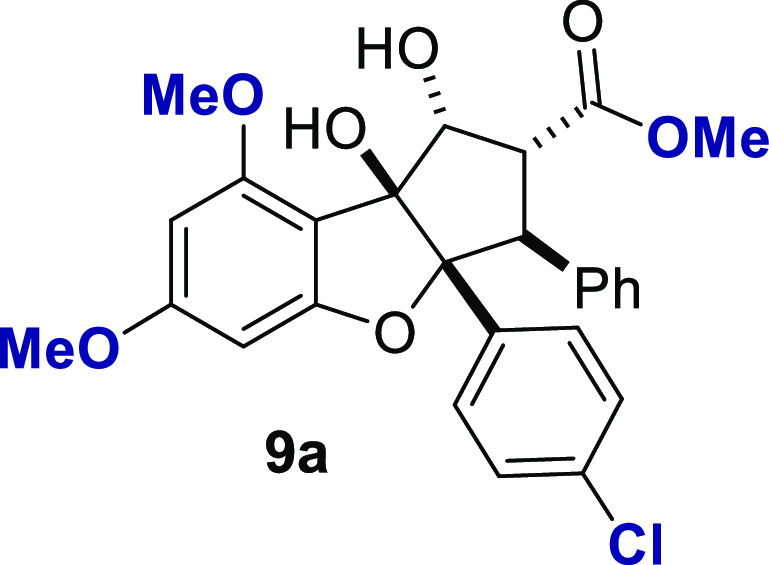



#### 2-Hydroxy-1-(2-hydroxy-4,6-dimethoxyphenyl)ethan-1-one (**4a**)

To a solution of 1-(2-hydroxy-4,6-dimethoxyphenyl)ethan-1-one (**3a***+)* (3.52 g, 17.9 mmol, 1.00 equiv) in dry CH_2_Cl_2_ (36 mL) were added freshly distilled Et_3_N (9.3 mL, 47.9 mmol, 2.67 equiv) and TBSOTf (9.5 mL, 41.3 mmol, 2.30 equiv) at 0 °C and stirred at the same temperature for 4 h. The reaction was terminated by the addition saturated aqueous NaHCO_3_ (50 mL) and warmed up to rt. The layers were separated and the aqueous layers were extracted with CH_2_Cl_2_ (3 × 50 mL). The collected organic layers were washed with brine, dried over MgSO_4_, filtered and concentrated *in vacuo*. The crude/biphasic solution was diluted with Et_2_O, washed with a saturated aqueous NH_4_Cl solution, dried over MgSO_4_, filtered and concentrated *in vacuo*. The solvent residue was removed under high vacuum and the crude TBS-enol ether as thick red syrup was used directly for the next step. A suspension of NaHCO_3_ (3.21 g, 38.2 mmol, 2.50 equiv) and *m*CPBA (77 wt%, 6.04 g, 35.0 mmol, 1.60 equiv) in dry CH_2_Cl_2_ (44 mL) was prepared and stirred at rt for 30 min. A solution of crude TBS-enol ether (6.50 g) in dry CH_2_Cl_2_ (23 mL) was then added to the *m*CPBA suspension at 0 °C and stirred for 30 min. The reaction mixture was warmed up to rt and stirred for 2 h. The reaction was terminated by dilution with CH_2_Cl_2_ and washed extensively with NaHCO_3_ (sat., aq.) to remove the *m*CPBA residue. The organic layers were washed with water, NaCl (sat., aq.), dried over MgSO_4_, filtered and concentrated *in vacuo*. The thick red syrup crude was used for the next reaction without further purification. To the epoxide crude (6.69 g) in THF/H_2_O (10:1, 66 mL) was added *p*TsOH·H_2_O (0.29 mg, 1.5 mmol, 0.10 equiv) and stirred under refluxing conditions for 6 h. The reaction was cooled down to rt and extracted with EtOAc, washed with NaHCO_3_(sat., aq.), water, NaCl (sat., aq.), dried over MgSO_4_, filtered and concentrated *in vacuo*. The crude extract was stirred under refluxing conditions in EtOH for 1 h and slowly precipitated overnight at rt. The suspension was filtered and washed with cold EtOH to afford **4a** as a pale-orange solid (1.09 g, 5.13 mmol, 60% over three steps). *R*_f_ = 0.42 (petroleum ether/EtOAc 1:1); ^**1**^**H NMR** (CDCl_3_, 400 MHz): δ [ppm] 13.22 (s, 1H, O*H*), 6.11 (d, *J* = 2.3 Hz, 1H, Ar*H*), 5.94 (d, *J* = 2.3 Hz, 1H, Ar*H*), 4.71 (s, 2H, C*H*_2_OH), 3.87 (s, 3H, OC*H*_3_), 3.84 (s, 3H, OC*H*_3_); ^**13**^**C NMR** (CDCl_3_, 100 MHz): δ [ppm] 201.9 (q, *C*=O), 167.3 (q, Ar*C*), 167.1 (q, Ar*C*), 163.32 (q, Ar*C*), 93.8 (t, Ar*C*H), 91.0 (t, Ar*C*H), 68.9 (s, *C*H_2_OH), 55.73 (p, O*C*H_3_), 55.71 (p, O*C*H_3_). The analytical data are consistent with those reported in the literature.^21^

#### 2-(2-((4-Chlorobenzoyl)oxy)-4,6-dimethoxyphenyl)-2-oxo-ethyl 4-chlorobenzoate (**5a**)

To a solution of alcohol **4a** (1.09 g, 5.16 mmol, 1.00 equiv) in dry CH_2_Cl_2_ (14 mL) were added DMAP (0.03 mg, 0.26 mmol, 0.05 equiv) and freshly distilled triethylamine (2.2 mL, 15.5 mmol, 3.00 equiv) and cooled down to 0 °C. To the cold suspension was added 4-chlorobenzoyl chloride (0.36 mL, 2.69 mmol, 2.00 equiv) and warmed up to rt. The orange suspension was stirred at rt for 3 h, before the reaction was terminated by the addition of HCl solution (1 M, 15 mL). The layers were separated and the aqueous layers were extracted with CH_2_Cl_2_. The collected organic layers were washed with NaCl (sat., aq.), dried over MgSO_4_, filtered and concentrated *in vacuo*. The crude product **5a** was used for the next step without further purification. *R*_f_ = 0.50 (petroleum ether/EtOAc 2:1); ^**1**^**H NMR** (CDCl_3_, 400 MHz): δ [ppm] 8.08 (d, *J* = 8.4 Hz, 2H, 2× Ar*H*), 7.94 (d, *J* = 8.4 Hz, 2H, 2× Ar*H*), 7.43 (d, *J* = 8.4 Hz, 2H, 2× Ar*H*), 7.38 (d, *J* = 8.4 Hz, 2H, 2× Ar*H*), 6.42 (d, *J* = 3.3 Hz, 2H, 2× Ar*H*), 5.27 (s, 2H, CH_2_), 3.89 (s, 3H, OCH_3_), 3.85 (s, 3H, OCH_3_).

#### 1-(4-Chlorophenyl)-3-(2-hydroxy-4,6-dimethoxyphenyl)-1,3-dioxopropan-2-yl 4-chlorobenzoate (**6a**)

To a solution of diester **5a** (2.52 g, 5.16 mmol, 1.00 equiv) crude in dry THF (29 mL) was added LiHMDS (1.0 M in THF, 15.5 mL, 15.5 mmol, 3.00 equiv) at −20 °C. The resulting red solution was stirred at the same temperature for 1 h. The reaction was terminated by the addition of NH_4_Cl (sat., aq.) and warmed up to rt for 5 min. The layers were separated, the aqueous layers were extracted with EtOAc. The collected organic layers were washed with NaCl (sat., aq.), dried over MgSO_4_, filtered and carefully concentrated *in vacuo*. The crude product **6a** was used for the next step without further purification. *R*_f_ = 0.54 (petroleum ether/EtOAc 1:1); ^**1**^**H NMR** (CDCl_3_, 400 MHz): δ [ppm] 13.17 (s, 1H, O*H*), 8.04 (d, *J* = 8.8 Hz, 2H, 2× Ar*H*), 7.96 (d, *J* = 8.7 Hz, 2H, 2× Ar*H*), 7.50 (d, *J* = 7.8 Hz, 2H, 2× Ar*H*), 7.42 (d, *J* = 8.7 Hz, 2H, 2× Ar*H* “), 7.38 (s, 1H, C*H*), 6.12 (d, *J* = 2.2 Hz, 1H, Ar*H*), 5.84 (d, *J* = 2.2 Hz, 1H, Ar*H*), 3.82 (s, 3H, OCH_3_), 3.36 (s, 3H, OCH_3_).

#### 2-(4-Chlorophenyl)-5,7-dimethoxy-4-oxo-4*H*-chromen-3-yl 4-chlorobenzoate (**7a**)

To a solution of crude **6a** (2.52 g) in AcOH (65 mL) was added H_2_SO_4_ (1.37 mL, 25.8 mmol, 5.00 equiv), stirred at 80 °C and monitored by TLC. After all the starting material was consumed, the acidic solution was poured into ice water and stirred for 15 min. The resulting precipitate was filtered with Büchner funnel and washed with cold water. The solid was dried and recrystallized in EtOH to give **7a** (1.46 g, 3.10 mmol, 60% over three steps) as a beige solid. *R*_f_ = 0.61 (petroleum ether/EtOAc 1:1); ^**1**^**H NMR** (CDCl_3_, 400 MHz): δ [ppm] 8.11 (d, *J* = 8.7 Hz, 2H, 2× Ar*H*), 7.82 (d, *J* = 8.8 Hz, 2H, 2× Ar*H*), 7.45 (dd, *J* = 13, 5.7 Hz, 4H, 4× Ar*H*), 6.56 (d, *J* = 2.2 Hz, 1H, Ar*H*), 6.36 (d, *J* = 2.1 Hz, 1H, Ar*H*), 3.93 (s, 3H, OC*H*_3_), 3.92 (s, 3H, OC*H*_3_).

#### 2-(4-Chlorophenyl)-3-hydroxy-5,7-dimethoxy-4*H*-chromen-4-one (**8a**)

To a suspension of **7a** (1.46 g, 3.10 mmol, 1.00 equiv) was added an aqueous NaOH solution (5 wt%, 4.5 mL, 5.82 mmol, 1.88 equiv) in EtOH (42 mL). The reaction was heated to 80 °C and stirred for 1 h. After starting material was fully consumed, the reaction was terminated by the addition of an HCl solution (aq., 1 M, 5.82 mL, 5.82 mmol, 1.88 equiv). The precipitate was filtered and washed with cold EtOH to afford **8a** as a yellow solid (854 mg, 2.56 mmol, 83%). *R*_f_ = 0.78 (petroleum ether/EtOAc 1:1); ^**1**^**H NMR** (CDCl_3_, 400 MHz): δ [ppm] 8.16 (d, *J* = 8.8 Hz, 2H, 2× Ar*H*), 7.49 (d, *J* = 8.9 Hz, 2H, 2× Ar*H*), 7.46 (m, 1H, O*H*), 6.56 (d, *J* = 2.1 Hz, 1H, Ar*H*), 6.36 (d, *J* = 3.2 Hz, 1H, Ar*H*), 3.98 (s, OC*H*_3_), 3.92 (s, OC*H*_3_). ^**13**^**C NMR** (CDCl_3_, 100 MHz): δ [ppm] 171.9 (q, *C*=O), 164.7 (q, Ar*C*), 160.6 (q, Ar*C*), 158.9 (q, Ar*C*), 140.7 (q, *C*=COH), 138.4 (q, *C*OH), 135.5 (q, Ar*C*), 129.6 (q, Ar*C*), 128.8 (t, 2× Ar*C*), 128.4 (t, 2× Ar*C*), 106.2 (q, Ar*C*), 95.8 (t, Ar*C*), 92.3 (t, Ar*C*), 56.4 (p, CH_3_O), 55.9 (p, CH_3_O); **HRMS (ESI**^**+**^**)***m*/*z* calcd for C_17_H_13_ClO_5_ [M+H]^+^ 333.0530, found 333.0533.

#### (±)-Methyl (1*R*,2*R*,3*S*,3a*R*,8b*S*)-3a-(4-chlorophenyl)-1,8b-dihydroxy-6,8-dimethoxy-3-phenyl-2,3,3a,8b-tetrahydro-1*H*-cyclopenta[*b*]benzofuran-2-carboxylate (**9a**)

To a solution of **8a** (508 mg, 1.53 mmol, 1.00 equiv) in dry 2,2,2-TFE (13 mL) and dry CHCl_3_ (31 mL) was added methyl cinnamate (3.51 g, 21.7 mmol, 14.2 equiv). The clear solution was degassed with argon for 15 min, followed by UV-irradiation (100 W, 365 nm) at −5 °C for 10–16 h. After the reaction was finished, the solvent was removed *in vacuo* and the excess of methyl cinnamate was removed by silica gel purification (petroleum ether/EtOAc 10:1, then 4:1, then EtOAc). The product mixture was used directly for the next step. To the solution of cycloadduct crude (727 mg) in dry MeOH (49 mL) was added NaOMe (25 wt% in MeOH, 902 μL, 4.17 mmol, 2.84 equiv) and stirred under refluxing conditions for 1 h The reaction was terminated by the addition of NH_4_Cl (sat., aq.). The aqueous layers were extracted with EtOAc. The collected organic layers were washed with water, NaCl (sat., aq.), dried over MgSO_4_, filtered and concentrated *in vacuo*. The ketone crude product was directly used for the next step. A solution of Me_4_NBH(OAc)_3_ (2.34 g, 8.92 mmol, 6.42 equiv) and freshly distilled AcOH (839 μL, 14.5 mmol, 10.4 equiv) in dry MeCN (36 mL) was prepared and stirred at rt for 10 min. To this solution was added ketone crude product (688 mg) in dry MeCN (23 mL). The reaction was carried out under light exclusion and stirred for 19 h at rt. The reaction was terminated by the addition of NaK-tartrate (sat., aq.) and NH_4_Cl (sat., aq.). The layers were separated and the aqueous layers were extracted with CH_2_Cl_2_. The collected organic layers were washed with water and NaCl (sat., aq.), dried over MgSO_4_, filtered and concentrated *in vacuo*. The crude product was purified by silica gel column chromatography (petroleum ether/EtOAc 3:1, then 2:1) to yield **9a** (272 mg, 0.55 mmol, 39% over three steps) as a pale-yellow foam. *R*_f_ = 0.43 (8% MeOH in CH_2_Cl_2_). ^**1**^**H NMR** (DMSO-*d*_6_, 400 MHz): δ [ppm] 7.12–6.96 (m, 4H, *H*-3′, *H*-5′, *H*-2″, *H*-6″), 7.09–7.07 (m, 3H, *H*-2′, *H*-6′, *H*-4″), 6.92 (d, *J* = 7.3 Hz, 2H, *H*-3″, *H*-5″), 6.28 (d, *J* = 1.9 Hz, 1H, *H*-5), 6.11 (d, *J* = 1.9 Hz, 1H, *H*-7), 5.24 (s, 1H, O*H*), 5.12 (d, *J* = 4.9 Hz, 1H, O*H*), 4.66 (t, *J* = 5.1 Hz, 1H, *H*-1), 4.23 (d, *J* = 14 Hz, 1H, *H*-3), 3.99 (dd, *J* = 14, 5.3 Hz, 1H, *H*-2), 3.78 (s, 3H, C*H*_3_O-6), 3.72 (s, 3H, C*H*_3_O-8), 3.55 (s, 3H, C*H*_3_O-11); ^**13**^**C NMR** (DMSO-*d*_6_, 100 MHz): δ [ppm] 170.3 (*C*=O), 162.8 (q, *C*-6), 160.3 (q, *C*-4a), 157.8 (q, *C*-8), 138.0 (q, *C*-1′), 135.9 (q, *C*-1″), 130.9 (q, *C*-4′), 129.4 (t, *C*-2′, *C*-6′), 127.7 (t, *C*-2″, *C*-6″), 127.6 (t, *C*-3″, *C*-5″), 126.3 (t, *C*-3′, *C*-5′), 125.9 (t, *C*-4″), 107.9 (q, *C*-8a), 101.2 (q, *C*-3a), 93.5 (q, *C*-8b), 91.9 (t, *C*-7), 88.3 (t, *C*-5), 78.8 (t, *C*-1), 55.5 (p, H_3_*C*O-6/8), 55.4 (p, H_3_*C*O-6/8), 54.8 (t, *C*-3), 51.5 (p, H_3_*C*O-11), 51.0 (t, *C*-2); **HRMS (ESI**^**+**^**)***m*/*z* calc. for C_27_H_25_FO_7_Na [M+Na]^+^ 503.1477, found 503.1482; **HPLC purity** ∼100.00%.

### Synthesis of (±)-Methyl (1*R*,2*R*,3*S*,3a*R*,8b*S*)-3a-(4-fluorophenyl)-1,8b-dihydroxy-6,8-dimethoxy-3-phenyl-2,3,3a,8b-tetrahydro-1*H*-cyclopenta[*b*]benzofuran-2-carboxylate (**11ba**)

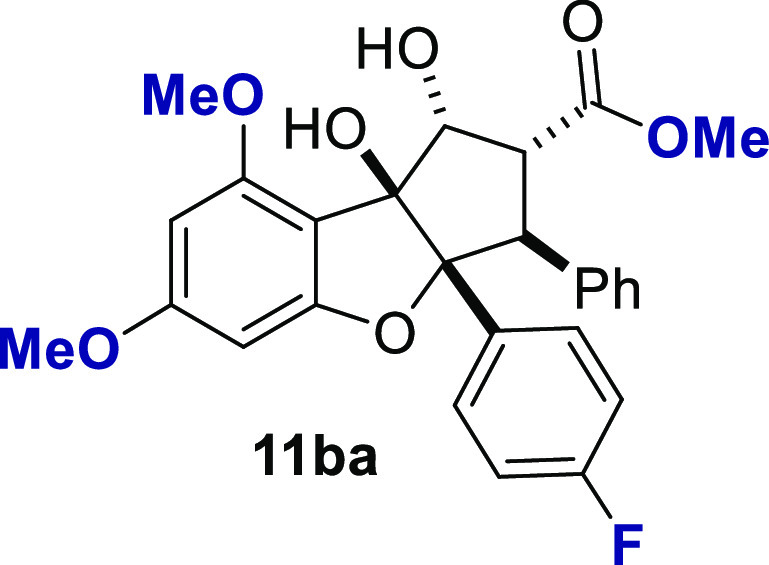



#### 1-(4-(Benzyloxy)-2-hydroxy-6-methoxyphenyl)-2-hydroxyethan-1-one (**4b**)

A solution of 4-benzyloxy-2-hydroxy-6-methoxyacetophenone (**3b**) (16.9 g, 62.0 mmol, 1.00 equiv) in CH_2_Cl_2_ (125 mL) was cooled to 0 °C, treated with Et_3_N (21.6 mL, 155 mmol, 2.50 equiv) and TBSOTf (32.8 mL, 143 mmol, 2.30 equiv) and stirred at 0 °C for 2.5 h. The reaction was terminated by the addition NaHCO_3_ solution (sat., aq.) and was allowed to warm to rt. The phases were separated and the aqueous phase was extracted with CH_2_Cl_2_ (3×). The combined organic phases were dried over MgSO_4_, filtered and concentrated under reduced pressure. The yielding two-phasic mixture of the product and triethylammonium triflate was diluted with Et_2_O and NH_4_Cl solution (sat., aq.) and the phases were separated. The aqueous phase was extracted with Et_2_O (100 mL, 3×). The organic phases were combined, dried over MgSO_4_, filtered and concentrated under reduced pressure. The TBS-enol ether was collected as a salmon-colored solid (31.8 g) and dissolved in CH_2_Cl_2_ (60.0 mL) and added to a suspension of *m*CPBA (77 wt%, 21.4 g, 86.8 mmol, 1.40 equiv) and NaHCO_3_ (11.2 g, 133 mmol, 2.15 equiv) in CH_2_Cl_2_ (240 mL) at 0 °C. The resulting mixture was allowed to warm to rt and stirred for 2 h. Then, the reaction mixture was diluted with CH_2_Cl_2_ (300 mL), washed with NaHCO_3_ (sat., aq.) and H_2_O, dried over MgSO_4_ and filtered. After concentration under reduced pressure, the crude epoxide was obtained as a brown viscous oil (32.1 g) and dissolves in a mixture of THF (320 mL) and H_2_O (32.0 mL). The solution was treated with *p*TsOH·H_2_O (1.18 g, 6.20 mmol, 10 mol %). The orange reaction mixture was heated under refluxing conditions for 6 h. The mixture was allowed to cool to rt and partitioned between EtOAc and NaHCO_3_ solution (sat., aq.). The organic phase was dried over MgSO_4_, filtered and concentrated under reduced pressure. After purification by column chromatography (petroleum ether/EtOAc 5:1 → 2:1) the desired product **4b** was obtained as a pale-brown solid (10.9 g, 37.8 mmol, 61% over three steps). *R*_f_ = 0.21 (petroleum ether/EtOAc 3:1); ^**1**^**H NMR** (CDCl_3_, 400 MHz): δ [ppm] 13.21 (s, 1H, O*H*), 7.43–7.34 (m, 5H, 5× Ar*H*), 6.19 (d, *J* = 2.3 Hz, 1H, Ar*H*), 6.02 (d, *J* = 2.3 Hz, 1H, Ar*H*), 5.08 (s, 2H, C*H*_2_), 4.72 (s, 2H, C*H*_2_OH), 3.86 (s, 3H, OC*H*_3_); ^**13**^**C NMR** (CDCl_3_, 100 MHz): δ [ppm] 202.1 (q, *C*=O), 167.4 (q, Ar*C*), 166.3 (q, Ar*C*), 163.3 (q, Ar*C*), 135.7 (q, Ar*C*), 128.9 (t, 2× Ar*C*), 128.6 (t, Ar*C*H), 127.8 (t, Ar*C*H), 103.6 (q, Ar*C)*, 94.8 (t, Ar*C*H), 91.7 (t, Ar*C*H), 70.6 (s, *C*H_2_), 68.8 (s, *C*H_2_OH), 55.9 (p, O*C*H_3_). The analytical data are consistent with those reported in the literature.^20^

#### 2-(4-(Benzyloxy)-2-((4-fluorobenzoyl)oxy)-6-methoxyphen-yl)-2-oxoethyl 4-fluorobenzoate (**5ba**)

DMAP (21 mg, 0.17 mmol, 0.05 equiv) and Et_3_N (1.46 mL, 10.4 mmol, 3.00 equiv) were added into a solution of **4b** (1.00 g, 3.47 mmol, 1.00 equiv) in CH_2_Cl_2_ (12 mL), followed by the addition of 4-fluorobenzoyl chloride (0.82 mL, 6.94 mmol, 2.00 equiv) at 0 °C. The orange suspension was warmed up to rt and stirred for 3 h. The reaction was terminated by the addition of HCl (1 M, 10 mL) and extracted with CH_2_Cl_2_ (3 × 30 mL). The organic layers were washed with NaCl solution (sat., aq., 50 mL), dried over MgSO_4_, filtered and concentrated *in vacuo*. The crude extract **5ba** as a pale-orange solid (1.99 g) was directly used for the next step. *R*_f_= 0.38 (petroleum ether/EtOAc 2:1); ^**1**^**H NMR** (CDCl_3_, 400 MHz): δ [ppm] 8.16 (dd, *J* = 9.0, 5.4 Hz, 2H, 2× Ar*H*), 8.02 (dd, *J* = 9.0, 5.4 Hz, 2H, 2× Ar*H*), 7.42–7.33 (m, 5H, 5× Ar*H*), 7.10 (td, *J* = 22, 8.4 Hz, 4H, 4× Ar*H*), 6.53 (d, *J* = 2.2 Hz, 1H, Ar*H*), 6.49 (d, *J* = 2.2 Hz, 1H, Ar*H*), 5.27 (s, 2H, OC*H*_2_Ph), 5.08 (s, 2H, C*H*2), 3.86 (s, 3H, OC*H*_3_).

#### 2-(4-(Benzyloxy)-2-hydroxy-6-methoxyphenyl)-2-oxoethan-e-1,1-diyl bis(4-fluorobenzoate) (**6ba**)

LiHMDS (1 M in THF 10.4 mL, 10.4 mmol, 3.00 equiv) was added to the crude extract **5ba** (3.47 mmol) in THF (19 mL) at −30 °C and stirred at the same temperature for 1.5 h. The reaction was terminated by the addition of a saturated aqueous NH_4_Cl solution at −30 °C and warmed up to rt. The mixture was extracted with EtOAc (3 × 50 mL). The organic layers were washed with water and NaCl solution (sat., aq., 100 mL), dried over MgSO_4_, filtered and concentrated *in vacuo*. The crude extract **6ba** as a yellow foam (1.84 g) was used directly in the next step without further purification. *R*_f_ = 0.40 (petroleum ether/EtOAc 2:1); ^**1**^**H NMR** (CDCl_3_, 400 MHz): δ [ppm] 13.18 (s, 1H, -O*H*), 8.12 (dd, *J* = 9.0, 5.4 Hz, 2H, 2× Ar*H*), 8.06 (dd, *J* = 8.9, 5.3 Hz, 2H, 2× Ar*H*), 7.40–7.33 (m, 5H, 5× Ar*H*), 7.39 (s, 1H, C*H*) 7.20 (t, *J* = 8.6 Hz, 2H, 2× Ar*H*), 7.12 (t, *J* = 8.7 Hz, 2H, 2× Ar*H*), 6.20 (d, *J* = 2.3 Hz, 1H, Ar*H*), 5.92 (d, *J* = 2.3 Hz, 1H, Ar*H*), 5.07 (s, 2H, OC*H*_2_Ph), 3.34 (s, 3H, OC*H*_3_).

#### 7-(Benzyloxy)-2-(4-fluorophenyl)-5-methoxy-4-oxo-4+H-chromen-3-yl 4-fluorobenzoate (**7ba**)

Concentrated H_2_SO_4_ (0.92 mL, 17.3 mmol, 5.00 equiv) was added to crude **6ba** (3.47 mmol) dissolved in CH_3_COOH (43 mL) and stirred at rt for 16 h, monitored by TLC. In the presence of starting material, additional H_2_SO_4_ (0.92 mL, 17.3 mmol, 5.00 equiv) was added to the dark brown solution and stirred for further 16 h at rt. After full conversion of the starting material, the reaction mixture was poured into ice water and stirred for 15 min. The suspension was filtered by Büchner funnel. The precipitate was dissolved in CH_2_Cl_2_ and concentrated *in vacuo*. The crude extract was purified by silica gel column chromatography (PE/EtOAc = 4:1, then 2:1) to yield ester **7ba** (1.25 g, 2.42 mmol, 70% over three steps) as a yellow foam. *R*_f_ = 0.67 (petroleum ether/EtOAc 1:1); ^**1**^**H NMR** (CDCl_3_, 400 MHz): δ [ppm] 8.20 (dd, *J* = 9.0, 5.4 Hz, 2H, 2× Ar*H*), 7.89 (dd, *J* = 9.1, 5.3 Hz, 2H, 2× Ar*H*), 7.48–7.36 (m, 5H, 5× Ar*H*), 7.15 (td, *J* = 8.6, 3.8 Hz, 4H, 4× Ar*H*), 6.64 (d, *J* = 2.2 Hz, 1H, Ar*H*), 6.47 (d, *J* = 2.2 Hz, 1H, Ar*H*), 5.16 (s, 2H, OC*H*_2_Ph), 3.91 (s, 3H, OC*H*_3_).

#### 7-(Benzyloxy)-2-(4-fluorophenyl)-3-hydroxy-5-methoxy-4*H*-chromen-4-one (**8ba**)

NaOH (5% aqueous, 1.09 mL, 1.42 mmol, 1.88 equiv) was added to **7ba** (390 mg, 0.76 mmol, 1.00 equiv) in EtOH (10 mL). The yellow suspension was stirred 3 h at rt. The reaction was terminated by the addition of an aqueous HCl solution (1 M, 1.42 mmol, 1.42 mL, 1.88 equiv) and precipitated yellow solids. The suspension was filtered and the precipitate was washed with cold EtOH to give pure product **8ba**. The mother liquor was concentrated and was purified further by silica gel column chromatography (petroleum ether/EtOAc 2:1, then 1:1) to give total product **8ba** as a yellow solid (264 mg, 0.69 mmol 91%). *R*_f_ = 0.33 (petroleum ether/EtOAc 1:2); ^**1**^**H NMR** (CDCl_3_, 400 MHz): δ [ppm] 8.22 (dd, *J* = 9.1, 5.4 Hz, 2H, 2× Ar*H*), 7.48–7.37 (m, 5H, 5× Ar*H*), 7.20 (t, *J* = 8.8 Hz, 2H, 2× Ar*H*), 6.65 (d, *J* = 2.0 Hz, 1H, Ar*H*), 6.46 (d, *J* = 2.1 Hz, 1H, Ar*H*), 5.17 (s, 2H, OC*H*_2_Ph), 3.98 (s, 3H, OC*H*_3_); ^**13**^**C NMR** (CDCl_3_, 100 MHz): δ [ppm] 172.0 (q, *C*=O), 163.7 (q, Ar*C*), 163.3 (q, *d*, *J* = 251 Hz, Ar*C*), 160.7 (q, Ar*C*), 158.9 (q, Ar*C*), 141.2 (q, *C*=COH), 138.0 (q, *C*OH), 135.5 (2× Ar*C*), 129.4 (t, d, *J* = 8.5 Hz, 2× Ar*C*), 128.8 (t, 2× Ar*C*), 128.6 (t, Ar*C*), 127.7 (t, 2× Ar*C*), 127.3 (q, *d*, *J* = 3.2 Hz, Ar*C*) 115.7 (t, *d*, *J* = 22 Hz, 2× Ar*C*), 106.4 (q, Ar*C*), 96.3 (t, Ar*C*), 93.4 (t, Ar*C*), 70.7 (s, O*C*H_2_Ph), 56.5 (p, O*C*H_3_); **HRMS (ESI**^**+**^**)***m*/*z* calcd for C_23_H_17_FO_5_Na [M-Na]^+^ 415.0958, found 415.0964.

#### (±)-Methyl-6-(benzyloxy)-3a-(4-fluorophenyl)-1,8b-dihydroxy-8-methoxy-3-phenyl-2,3,3a,8b-tetrahydro-1*H*-cyclopenta[*b*]benzofuran-2-carboxylate (**9ba**)

Methyl cinnamate (1.19 g, 7.38 mmol, 14.20 equiv) was added to flavonol **8ba** (204 mg, 0.52 mmol, 1.00 equiv) in CHCl_3_ (10.4 mL) and 2,2,2-trifluoroethanol (4.3 mL). The solution was degassed with argon for 20 min and irradiated (100 W, 365 nm) at −10 °C under argon atmosphere for 16–40 h. After starting material **8bb** was fully consumed, the reaction mixture was concentrated *in vacuo* and the methyl cinnamate excess was removed by silica gel column chromatography (petroleum ether/EtOAc 4:1, then 1:1). The desired cycloadduct was obtained as a mixture of isomers as a yellow foam (228 mg). The isomer mixture was used submitted for subsequent reaction without further purification. The mixture (228 mg, 0.41 mmol, 1.00) was dissolved in dry MeOH (13.5 mL) and NaOMe (25 wt% in MeOH, 250 μL, 1.16 mmol, 2.84 equiv) was added. The reaction was stirred under refluxing conditions for 1 h. The reaction was terminated by the addition of NH_4_Cl solution (sat., aq., 10 mL) and extracted with EtOAc (3 × 30 mL). The organic layers were washed with water and NaCl (sat., aq.), dried over MgSO_4_, filtered and concentrated *in vacuo* to give the desired keto ester mixture as a yellow foam (228 mg) and used the directly for the next step. A suspension of Me_4_NBH(OAc)_3_ (688 mg, 2.62 mmol, 6.42 equiv) and freshly distilled CH_3_COOH (246 μL, 4.24 mmol, 10.4 equiv) in dry MeCN (10.5 mL) was prepared and stirred at rt for 5 min. To the prepared suspension was added the keto ester mixture (228 mg, 0.41 mmol, 1.00 equiv) and stirred for 16 h at rt under light protection. The reaction was terminated by the addition of NH_4_Cl solution (sat., aq.) and NaK-tartrate (sat., aq.) solution and extracted with CH_2_Cl_2_ (3 × 60 mL). The organic layers were washed with water and NaCl (sat., aq.), dried over MgSO_4_, filtered and concentrated *in vacuo*. The crude extract was purified by silica column chromatography (petroleum ether/EtOAc 3:2) to give racemic *endo*-product **9ba** (107 mg, 0.19 mmol, 47% over three steps) as a pale-yellow foam. *R*_f_ = 0.52 (EtOAc); ^**1**^**H NMR** (CDCl_3_, 400 MHz): δ [ppm] 7.47–7.36 (m, 5H, *H*-3‴, *H*-4‴, *H*-5‴, *H*-6‴, *H*-7‴), 7.18–7.15 (m, 2H, *H*-2′, *H*-6′), 7.07–7.05 (m, 3H, *H*-2″, *H*-4″, *H*-6″), 6.86–6.80 (m, 4H, *H*-3′, *H*-5′, *H*-3″, *H*-5″), 6.36 (d, *J* = 1.7 Hz, 1H, *H*-5), 6.22 (d, *J* = 1.7 Hz, 1H, *H*-7), 5.09 (s, 2H, *H*-1‴), 5.03 (d, *J* = 6.5 Hz, 1H, *H*-1), 4.33 (d, *J* = 14 Hz, 1H, *H*-3), 3.91–3.86 (m, 1H, *H*-2), 3.86 (s, 3H, C*H*_3_O-8), 3.65 (s, 3H, C*H*_3_O-11); ^**13**^**C NMR** (CDCl_3_, 100 MHz): δ [ppm] 170.4 (q, *C*-11), 163.3 (q, *C*-8), 161.9 (q, *d*, *J* = 247 Hz, *C*-4′), 160.6 (q, *C*-4a), 156.9 (q, *C*-6), 136.6 (q, *C*-1″), 136.4 (C-2‴), 130.4 (q, *d*, *J* = 3.3 Hz, *C*-1′), 129.6 (t, *d*, *J* = 8.1 Hz, 2C, C-2′, C-6′), 128.7 (t, *C*-4‴, *C*-6‴), 128.2 (t, *C*-5‴), 127.8 (t, *C*-2″, *C*-6″), 127.7 (t, *C*-3″, *C*-5″), 127.6 (t, *C*-3‴, *C*-7‴), 126.7 (t, *C*-4″), 114.1 (t, *d*, *J* = 21 Hz, *C-*3′, *C*-5′), 107.7 (q, *C*-8a), 101.7 (*C*-3a), 93.7 (t, *C*-7), 93.5 (t, *C*-5), 90.5 (*C-*8b), 79.6 (t, *C*-1), 70.5 (s, *C*-1‴), 55.8 (p, H_3_*C*O-11), 55.1 (t, *C*-3), 52.1 (p, H_3_*C*O-8), 50.3 (t, *C*-2). **HRMS (ESI**^**+**^**)***m*/*z* calcd for C_33_H_29_FO_7_Na [M+Na]^+^ 579.1795, found 579.1799.

#### (±)-Methyl-3a-(4-fluorophenyl)-1,6,8b-trihydroxy-8-methoxy-3-phenyl-2,3,3a,8b-tetrahydro-1*H*-cyclopenta[*b*]benzofuran-2-carboxylate (**10ba**)

Pd/C (10%, 6.6 mg, 0.006 mmol, 0.05 equiv) and **9ba** (51 mg, 0.09 mmol, 1.00 equiv) was dissolved in THF (0.92 mL). Hydrogen gas was bubbled through the black suspension for 10 min at rt. The reaction was carried under H_2_-atmosphere (high-pressure hydrogen balloons were attached) for 16 h. After the reaction was finished (monitored by TLC), the reaction mixture was filtered over Celite to remove the Pd/C, rinsed with CH_2_Cl_2_ and concentrated *in vacuo* to give crude phenol **10ba** (43 mg) as a yellow foam. The crude product was submitted for subsequent reaction without further purification. *R*_f_ = 0.20 (petroleum/EtOAc 1:2).

#### *(* ± *)-Methyl-3a-(4-fluorophenyl)-1,8b-dihydroxy-6,8-dime-thoxy-3-phenyl-2,3,3a,8b-tetrahydro-1*H*-cyclopenta-[*b*]benzofuran-2-carboxylate (***11ba***)*

To crude **10ba** (50 mg, 0.11 mmol, 1.00 equiv) in toluene/MeOH (1:1, 7 mL) was added TMSCHN_2_ (2 M in hexanes, 0.86 mL, 1.72 mmol, 16.0 equiv) and stirred 3 h at rt. After the reaction was finished (monitored by TLC), the solvent was removed *in vacuo*. The crude extract was purified by silica gel column chromatography to afford **11ba** (38 mg, 0.08 mmol, 71% over two steps) as a colorless foam. *R*_f_ = 0.29 (petroleum/EtOAc 1:2); ^**1**^**H NMR** (DMSO-*d*_6_, 400 MHz): δ [ppm] 7.12 (q, *J* = 4.8 Hz, 2H, *H*-2′, *H*-6′), 7.05 (t, *J* = 7.4 Hz, 2H, *H*-3″, *H*-5″), 6.98 (t, *J* = 7.3 Hz, 1H, *H*-4″), 6.89 (d, *J* = 7.4 Hz, 2H, *H*-2″, *H*-6″), 6.83 (t, *J* = 8.9 Hz, 2H, *H*-3′, *H*-5′), 6.29 (d, *J* = 1.9 Hz, 1H, *H*-5), 6.12 (d, *J* = 1.9 Hz, 1H, *H*-7), 5.22 (s, 1H, -O*H*), 5.10 (d, *J* = 4.8 Hz, 1H, -O*H*), 4.68 (t, *J* = 5.2 Hz, 1H, *H*-1), 4.21 (d, *J* = 14 Hz, *H*-3), 3.97 (dd, *J* = 14, 5.5 Hz, 1H, *H*-2), 3.78 (s, 3H, C*H*_3_O-6), 3.73 (s, 3H, C*H*_3_O-8), 3.55 (s, 3H, C*H*_3_O-11) ppm. ^**13**^**C NMR** (DMSO-*d*_6_, 100 MHz): δ [ppm] 170.7 (q, *C*-11), 163.2 (q, *C*-8), 161.1 (q, *C*-4′), 160.8 (q, *C*-4a), 158.3 (q, *C*-6), 138.5 (q, *C*-1″), 133.3 (q, *d*, *J* = 2.9 Hz, 1C, C-1′), 129.9 (t, *d*, *J* = 8.0 Hz, *C*-2′, *C*-6′), 128.1 (t, *C*-2″, *C*-6″), 127.9 (t, *C*-3″, *C*-5″), 126.4 (t, *C*-4″), 113.5 (t, *d*, *J* = 21 Hz, *C*-3′, *C*-5′), 108.5 (q, *C-*8a), 41.6 (q, *C*-3a), 93.8 (q, *C*-8b), 92.3 (t, *C*-7), 88.8 (t, *C*-5), 79.2 (t, *C*-1), 55.9 (p, H_3_*C*O-6), 55.8 (p, H_3_*C*O-8), 55.2 (t, *C*-3), 51.8 (p, H_3_*C*O-11), 51.4 (t, *C*-2) ppm. **HRMS (ESI**^**+**^**)***m*/*z* calcd for C_27_H_25_O_7_FNa [M+Na]^+^ 503.1482, found 503.1489; **HPLC Purity** 98.08%.

### Synthesis of (±)-Methyl (1*R*,2*R*,3*S*,3a*R*,8b*S*)-3a-(4-bromophenyl)-1,8b-dihydroxy-6,8-dimethoxy-3-phenyl-2,3,3a,8b-tetrahydro-1*H*-cyclopenta[*b*]benzofuran-2-carboxylate (**11bb**)

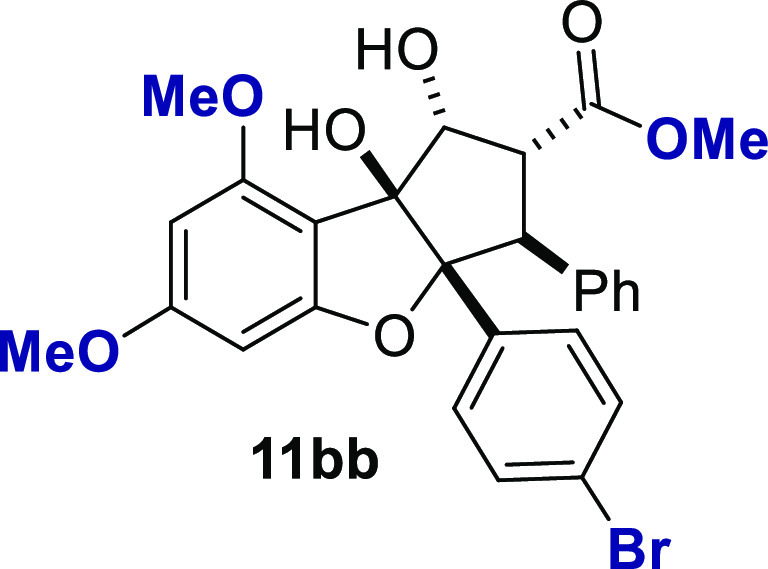



#### 2-(4-(Benzyloxy)-2-((4-bromobenzoyl)oxy)-6-methoxyphenyl)-2-oxoethyl 4-bromobenzoate (**5bb**)

A solution of the α-hydroxy ketone **4b** (3.07 g, 10.6 mmol, 1.00 equiv) in CH_2_Cl_2_ (35.5 mL) was treated with 4-DMAP (65.0 mg, 532 μmol, 5 mol %) and triethylamine (4.45 mL, 31.9 mmol, 3.00 equiv). The mixture was cooled to 0 °C and 4-bromobenzoyl chloride (4.67 g, 21.3 mmol, 2.00 equiv) was added and stirred at rt for 3.5 h. The solution was terminated by the addition of HCl (1.00 M in H_2_O) and the phases were separated. The aqueous phase was extracted with CH_2_Cl_2_ and the combined organic phases were dried over MgSO_4_, filtered and concentrated under reduced pressure. The desired bisbenzoate **5bb** was obtained as a yellow foam (6.97 g) and was used directly for the next step. *R*_f_ = 0.50 (petroleum ether/EtOAc 2:1).

#### 1-(4-(Benzyloxy)-2-hydroxy-6-methoxyphenyl)-3-(4-bromophenyl)-1,3-dioxopropan-2-yl 4-bromobenzoate (**6bb**)

A solution of crude bisbenzoate **5bb** (6.97 g, 10.7 mmol, 1.00 equiv) in THF (59.2 mL) was cooled to −20 °C and treated with LiHMDS solution (1.00 M in THF, 32.0 mL, 32.0 mmol, 3.00 equiv). The mixture was stirred at −20 °C for 30 min. Then, the reaction was terminated by the addition of NH_4_Cl solution (sat., aq.) and warmed to rt. The aqueous phase was extracted with EtOAc (3×) and the combined organic phases were dried over MgSO_4_, filtered and concentrated under reduced pressure. The residue was suspended in EtOH and heated under refluxing conditions for 15 min. After cooling to rt, the suspension was filtered and the solid was washed with cold EtOH. The desired phenol **6bb** was obtained as a pale-yellow solid (4.93 g, 7.54 mmol, 71% over two steps). *R*_f_ = 0.50 (petroleum ether/EtOAc 2:1); ^**1**^**H NMR** (CDCl_3_, 400 MHz): δ [ppm] 13.14 (bs, 1H, O*H*), 7.96 (d, *J* = 8.6 Hz, 2H, 2× Ar*H*), 7.89 (d, *J* = 8.6 Hz, 2H, 2× Ar*H*), 7.67 (d, *J* = 8.6 Hz, 2H, 2× Ar*H*), 7.59 (d, *J* = 8.6 Hz, 2H, 2× Ar*H*), 7.40–7.34 (m, 6H, 5× Ar*H*, C*H*O), 6.20 (d, *J* = 2.1 Hz, 1H, Ar*H*), 5.93 (d, *J* = 2.1 Hz, 1H, Ar*H*), 5.06 (s, 2H, OC*H*_2_Ph), 3.35 (s, 3H, OC*H*_3_); ^**13**^**C NMR** (CDCl_3_, 100 MHz): δ [ppm] 193.3 (q, *C*=O), 190.0 (q, *C*=O), 167.9 (q, *C*(=O)O), 166.4 (q, Ar*C*), 164.9 (q, Ar*C*), 161.5 (q, Ar*C*), 135.7 (q, Ar*C*), 133.5 (q, Ar*C*), 132.6 (t, 2× Ar*C*), 132.1 (t, 2× Ar*C*), 131.8 (t, 2× Ar*C*), 130.3 (t, 2× Ar*C*), 129.6 (q, Ar*C*), 129.3 (q, Ar*C*), 128.9 (t, 2× Ar*C*), 128.6 (t, Ar*C*H), 127.8 (t, 2× Ar*C*), 127.7 (q, Ar*C*), 104.6 (t, Ar*C*H), 95.3 (t, Ar*C*H), 91.9 (t, Ar*C*H), 76.9 (t, H*C*O), 70.6 (s, O*C*H_2_Ph), 55.6 (p, O*C*H_3_). The analytical data are consistent with those reported in the literature.^20^

#### 7-(Benzyloxy)-2-(4-bromophenyl)-5-methoxy-4-oxo-4*H*-chromen-3-yl 4-bromobenzoate (**7bb**)

A suspension of crude phenol **6bb** (4.42 g, 6.76 mmol, 1.00 equiv) in AcOH (92.0 mL) was treated with H_2_SO_4_ (96 wt%, 2.09 mL, 35.4 mmol, 5.24 equiv) and stirred at 50 °C for 20 h. The reaction mixture was poured into ice-cold H_2_O, the yellow suspension was filtered and the precipitate was washed with H_2_O. The wet solid was suspended in a minimal amount of EtOH and heated under refluxing conditions for 45 min. After cooling to rt, the mixture was filtered, the precipitate was washed with cold EtOH and dried under reduced pressure to give a mixture of **7bb** and ∼40% of the debenzylated flavonol ester. The solid was dissolved in DMF (65.0 mL) and treated with BnBr (807 μL, 6.76 mmol, 1.00 equiv) and K_2_CO_3_ (1.87 g, 13.5 mmol, 2.00 equiv), stirred at rt for 2.5 h and then diluted with CH_2_Cl_2_ (100 mL) and NaCl solution (sat., aq., 100 mL). The phases were separated and the organic phase was dried over MgSO_4_, filtered and concentrated under reduced pressure. The residue was suspended in a minimal amount of EtOH and heated to reflux for 1 h, cooled to rt and filtered. After washing with cold EtOH and drying under reduced pressure, the desired 3-benzyloxyflavonate **7bb** was obtained as a yellow solid (3.25 g, 5.11 mmol, 76%). *R*_f_ = 0.60 (petroleum ether/EtOAc 1:1); ^**1**^**H NMR** (CDCl_3_, 400 MHz): δ [ppm] 8.03 (d, *J* = 8.2 Hz, 2H, 2× Ar*H*), 7.74 (d, *J* = 8.3 Hz, 2H, 2× Ar*H*), 7.63 (d, *J* = 8.4 Hz, 2H, 2× Ar*H*), 7.58 (d, *J* = 8.3 Hz, 2H, 2× Ar*H*), 7.45–7.38 (m, 5H, 5× Ar*H*), 6.63 (s, 1H, Ar*H*), 6.47 (s, 1H, Ar*H*), 5.16 (s, 2H, OC*H*_2_Ph), 3.91 (s, 3H, OC*H*_3′_); ^**13**^**C NMR** (CDCl_3_, 100 MHz): δ [ppm] 170.4 (q, *C*=O), 163.9 (q, Ar*C*), 163.5 (q, O*C*=O), 161.5 (q, Ar*C*), 159.3 (q, Ar*C*), 152.7 (q, *C*=C–O), 135.6 (q, Ar*C*), 134.8 (q, C = *C*-O), 132.14 (t, 4× Ar*C*), 132.09 (t, 2× Ar*C*), 129.6 (t, 2× Ar*C*), 129.2 (q, Ar*C*), 129.0 (t, 2× Ar*C*), 128.9 (q, Ar*C*), 128.7 (t, Ar*C*H), 127.81 (q, Ar*C*), 127.76 (t, 2× Ar*C*), 125.8 (q, Ar*C*), 109.1 (q, Ar*C*), 97.0 (t, Ar*C*H), 93.7 (t, Ar*C*H), 70.8 (s, O*C*H_2_Ph), 56.5 (p, O*C*H_3_). The analytical data are consistent with those reported in the literature.^20^

#### 7-(Benzyloxy)-2-(4-bromophenyl)-3-hydroxy-5-methoxy-4*H*-chromen-4-one (**8bb**)

A suspension of the benzoate **7bb** (1.00 g, 1.57 mmol, 1.00 equiv) in EtOH (20.8 mL) was treated with NaOH solution (5 wt% in H_2_O, 2.39 mL, 3.14 mmol, 2.00 equiv). The yellowish suspension was stirred at 80 °C for 1.75 h. The reaction mixture was allowed to cool to rt and was neutralized with HCl (1.00 M in H_2_O, 3.30 mL, 3.30 mmol, 2.10 equiv). The resulting suspension was filtered on a Büchner funnel and the precipitate was washed with a small amount of cold ethanol. The solid was dried under reduced pressure to constant weight to give the desired 3-hydroxyflavone **8bb** as a yellowish solid (634 mg, 1.40 mmol) in 89% yield. *R*_f_ = 0.48 (petroleum ether/EtOAc 2:1); ^**1**^**H NMR** (CDCl_3_, 400 MHz): δ [ppm] 8.09 (d, *J* = 8.6 Hz, 2H, 2× Ar*H*), 7.64 (d, *J* = 8.6 Hz, 2H, 2× Ar*H*), 7.49–7.37 (m, 5H, 5× Ar*H*), 6.65 (d, *J* = 1.7 Hz, 1H, Ar*H*), 6.45 (d, *J* = 1.7 Hz, 1H, Ar*H*), 5.16 (s, 2H, OC*H*_2_Ph), 3.98 (s, 3H, OC*H*_3_)*;*^**13**^**C NMR** (CDCl_3_, 100 MHz): δ [ppm] 172.1 (q, *C*=O), 163.9 (q, Ar*C*), 160.8 (q, Ar*C*), 159.0 (q, Ar*C*), 141.0 (q, *C*=COH), 138.6 (q, *C*OH), 135.6 (q, Ar*C*), 131.9 (t, 2× Ar*C*), 130.2 (q, Ar*C*), 129.0 (t, 2× Ar*C*), 128.8 (t, 2× Ar*C*), 128.7 (t, Ar*C*H), 127.8 (t, 2× Ar*C*), 124.1 (q, Ar*C*), 106.5 (q, Ar*C*), 96.5 (t, Ar*C*H), 93.5 (t, Ar*C*H), 70.8 (s, O*C*H_2_Ph), 56.5 (p, O*C*H_3_). The analytical data are consistent with those reported in the literature.^20^

#### (±)-Methyl (1*R*,2*R*,3*S*,3a*R*,8b*S*)-6-(benzyloxy)-3a-(4-bromophenyl)-1,8b-dihydroxy-8-methoxy-3-phenyl-2,3,3a,8b-tetrahydro-1*H*-cyclopenta[*b*]benzofuran-2-carboxylate (**9bb**)

Methyl cinnamate (3.20 g, 19.7 mmol, 14.2 equiv) was added to a solution of flavonol **8bb** (629 mg, 1.39 mmol, 1.00 equiv) in dry chloroform (28.3 mL) and freshly distilled 2,2,2-trifluoroethanol (11.3 mL). The reaction mixture was degassed for 30 min, then cooled to −5 °C and irradiated with UV light (λ_max_ = 365 nm) until it no longer fluoresced greenish (24 h). Subsequently, the solvent was removed under reduced pressure. The remaining amount of methyl cinnamate was then removed by column chromatography (petroleum ether/EtOAc 5.5:1 → 1:1). The desired cycloadduct was obtained as a mixture of isomers as a yellowish foam (629 mg). Without any further purification the product of the first step (629 mg, 1.02 mmol, 1.00 equiv) was dissolved in MeOH (40.9 mL). Then NaOMe solution (25 wt% in MeOH, 799 μL, 3.37 mmol, 3.30 equiv) was added and the mixture was heated under refluxing conditions for 1 h. Subsequently, the reaction was terminated by the addition of NH_4_Cl solution (sat., aq.). The phases were separated and the aqueous phase was extracted with EtOAc (3×). The organic phases were combined, dried over MgSO_4_, filtered and the solvent was removed under reduced pressure. The desired keto ester was obtained as a mixture of isomers as a yellow, glassy foam (629 mg) and used directly for the next step. A mixture of (CH_3_)_4_N(OAc)_3_BH (1.73 g, 6.56 mmol, 6.42 equiv) and freshly distilled AcOH (612 μL, 10.6 mmol, 10.4 equiv) in MeCN (9.00 mL) was stirred for 5 min at rt. Then, a solution of the product of the second step (629 mg, 1.02 mmol, 1.00 equiv) in MeCN (6.00 mL) was added. The mixture was protected from light and stirred for 19 h at rt. The reaction was then terminated by adding NH_4_Cl solution (sat., aq.) and sodium potassium tartrate solution (aq., 2.00 M). The phases were separated and the aqueous layer was extracted with CH_2_Cl_2_ (3×). The combined organic layers were dried over MgSO_4_, filtered and concentrated under reduced pressure. Column chromatography (petroleum ether/EtOAc 5:1 → 1:1) was then performed to obtain the racemic *endo*-product **9bb** as a pale-yellow solid (293 mg, 483 μmol, 35% over three steps). *R*_f_ = 0.52 (petroleum ether/EtOAc 1:1); ^**1**^**H NMR** (CDCl_3_, 400 MHz): δ [ppm] 7.47–7.34 (m, 5H, *H*-3‴, *H*-4‴, *H*-5‴, *H*-6‴, *H*-7‴), 7.26 (d, *J* = 8.7 Hz, *H*-3′, *H*-5′), 7.08–7.05 (m, 5H, *H*-2′, *H*-6′, *H*-3″, *H*-4″, *H*-5″), 6.89–6.86 (m, 2H, *H*-2″, *H*-6″), 6.36 (d, *J* = 1.9 Hz, 1H, *H*-5), 6.22 (d, *J* = 1.9 Hz, 1H, *H*-7), 5.09 (s, 2H, *H*-1‴), 5.01 (dd, *J* = 6.5, 1.4 Hz, 1H, *H*-1), 4.35 (d, *J* = 14.2 Hz, 1H, *H*-3), 3.81 (dd, *J* = 14.2, 6.5 Hz, 1H, *H*-2), 3.86 (s, 3H, C*H*_3_O-8), 3.66 (s, 3H, C*H*_3_O-11), 3.59 (s, 1H, O*H*-8b), 1.85 (s, 1H, O*H*-1); ^**13**^**C NMR** (CDCl_3_, 100 MHz): δ [ppm] 170.5 (q, *C*-11), 163.5 (q, *C*-6), 160.8 (q, *C*-4a), 157.1 (q, *C*-8), 136.6 (q, *C*-2‴), 136.5 (q, *C*-1″), 133.9 (q, *C*-1′), 130.4 (t, *C*-3′, *C*-5′), 129.6 (t, *C*-2′, *C*-6′), 128.9 (t, *C*-4‴, *C*-6‴), 128.4 (t, *C*-5‴), 128.0 (t, *C*-3″, *C*-5″), 127.8 (t, *C*-2″, *C*-6″), 127.7 (t, *C*-3‴, *C*-7‴), 126.9 (t, *C*-4″), 121.8 (q. *C*-4′), 107.6 (q, *C*-8a), 101.8 (q, *C*-3a), 93.9 (q, *C*-8b), 93.6 (t, *C*-7), 90.6 (t, *C*-5), 79.7 (t, *C*-1), 70.7 (s, *C*-1‴), 55.9 (p, H_3_*C*O-8), 55.1 (t, *C*-3), 52.2 (p, H_3_*C*O-11), 50.5 (t, *C*-2). The analytical data are consistent with those reported in the literature.^20^

#### (±)-Methyl (1*R*,2*R*,3*S*,3a*R*,8b*S*)-3a-(4-bromophenyl)-1,6,8b-trihydroxy-8-methoxy-3-phenyl-2,3,3a,8b-tetrahydro-1*H*-cyclopenta[*b*]benzofuran-2-carboxylate (**10bb**)

Palladium-on-carbon (10 wt%, 78.2 mg, 73.5 μmol, 20 mol %) was added to a solution of endobenzyl ether **9bb** (227 mg, 368 μmol, 1.00 equiv) in dry THF (7.35 mL) under an argon atmosphere. The atmosphere was replaced by hydrogen and an additional balloon of hydrogen was placed on the flask. The reaction mixture was stirred for 50 min at rt and then filtered over Celite. The filtrate was concentrated to dryness and gave the desired phenol **10bb** as a colorless solid (177 mg, 336 μmol, 91%). **R**_**f**_ = 0.27 (CH_2_Cl_2_/EtOAc 19:1); ^**1**^**H NMR** (acetone-d_6_, 400 MHz): δ [ppm] 7.22 (dt, J = 9.1, 2.2 Hz, 2H, H-3′, H-5′), 7.14 (dt, J = 9.1, 2.2 Hz, 2H, H-2′, H-6′), 7.07–6.95 (m, 5H, H-2″, H-3″, H-4″, H-5″, H-6″), 6.17 (d, J = 1.8 Hz, H-5), 6.10 (d, J = 1.8 Hz, H-7), 4.90 (d, J = 5.8 Hz, H-1), 4.37 (d, J = 14.1 Hz, H-3), 4.26 (bs, 1H, OH-8b), 4.01 (dd, J = 14.1, 6.1 Hz, 1H, H-2), 3.80 (s, 3H, CH_3_O-8), 3.56 (s, 3H, CH_3_O-11); ^**13**^**C NMR** (acetone-d_6_, 100 MHz): δ [ppm] 170.9 (q, C-11), 162.4 (q, C-6), 161.6 (q, C-4a), 158.7 (q, C-8), 138.9 (q, C-1″), 136.9 (q, C-1′), 130.9 (t, C-3′, C-5′), 130.3 (t, C-2′, C-6′), 128.7 (t, C-3″, C-5″), 128.4 (t, C-2″, C-6″), 127.0 (t, C-4″), 121.0 (q. C-4′), 107.6 (q, C-8a), 102.4 (q, C-3a), 94.6 (q, C-8b), 93.4 (t, C-7), 91.7 (t, C-5), 80.6 (t, C-1), 55.8 (p, H_3_CO-8), 55.7 (t, C-3), 51.70 (p, H_3_CO-11), 51.67 (t, C-2); **HRMS (ESI**^**–**^**)***m*/*z* calcd for C_26_H_22_BrO_7_ [M-H]^−^ 525.0549, found 525.0562.

(±)-Methyl (1*R*,2*R*,3*S*,3a*R*,8b*S*)-3a-(4-bromophenyl)-1,8b-dihydroxy-6,8-dimethoxy-3-phenyl-2,3,3a,8b-tetrahydro-1*H*-cyclopenta[*b*]benzofuran-2-carboxylate (**11bb**) A solution of the phenol **10bb** (166 mg, 315 μmol, 1.00 equiv) in toluene (10.5 mL) and MeOH (10.5 mL) was treated with trimethylsilyldiazomethane (2.00 M in Et_2_O, 1.57 mL, 3.15 mmol, 10.0 equiv) and stirred for 4 h at rt. The solvents were removed under reduced pressure. The residue was purified using silica gel chromatography (petroleum ether/EtOAc 2:1) to give the desired rocaglate **11bb** as a colorless foam (135 mg, 249 μmol, 79%). **R**_**f**_ = 0.41 (petroleum ether/EtOAc 1:1); ^**1**^**H NMR** (DMSO-*d*_6_, 400 MHz): δ [ppm] 7.20 (dt, J = 9.4, 2.2 Hz, 2H, H-3′, H-5′), 7.08–7.04 (m, 4H, H-2′, H-6′, H-2″, H-6″), 7.01–6.97 (m, 1H, H-4″), 6.93 (d, J = 7.4 Hz, 2H, H-3″, H-5″), 6.28 (d, J = 2.0 Hz, 1H, H-5), 6.11 (d, J = 2.0 Hz, 1H, H-7), 5.25 (s, 1H, OH-8b), 5.12 (d, J = 4.9 Hz, 1H, OH-1), 4.65 (t, J = 5.1 Hz, 1H, H-1), 4.24 (d, J = 14.0 Hz, 1H, H-3), 4.00 (dd, J = 14.0, 5.3 Hz, 1H, H-2), 3.78 (s, 3H, CH_3_O-6), 3.72 (s, 3H, CH_3_O-8), 3.56 (s, 3H, CH_3_O-11); ^**13**^**C NMR** (DMSO-*d*_6_, 100 MHz): δ [ppm] 170.3 (q, C-11), 162.7 (q, C-6), 160.4 (q, C-4a), 157.8 (q, C-8), 138.0 (q, C-1″), 136.3 (q, C-1′), 129.8 (t, C-3′, C-5′), 129.2 (t, C-2′, C-6′), 127.7 (t, C-3″, C-5″), 127.6 (t, C-2″, C-6″), 126.0 (t, C-4″), 119.6 (q. C-4′), 107.8 (q, C-8a), 101.2 (q, C-3a), 93.4 (q, C-8b), 91.9 (t, C-7), 88.3 (t, C-5), 78.7 (t, C-1), 55.5 (p, H_3_CO-6), 55.3 (p, H_3_CO-8), 54.7 (t, C-3), 51.3 (p, H_3_CO-11), 51.1 (t, C-2); **HRMS (ESI**^**+**^**)***m*/*z* calcd for C_27_H_25_O_7_BrNa [M+Na]^+^ 563.0681, found 563.0680; **HPLC purity** 99.69%. The analytical data are consistent with those reported in the literature.^16^

### Synthesis of (±)-Methyl (1*R*,2*R*,3*S*,3a*R*,8b*S*)-1,8b-dihydroxy-6,8-dimethoxy-3a-(4-methoxyphenyl)-3-phenyl-2,3,3a,8b-tetrahydro-1*H*-cyclopenta[*b*]benzofuran-2-carboxylate (**11bc**)

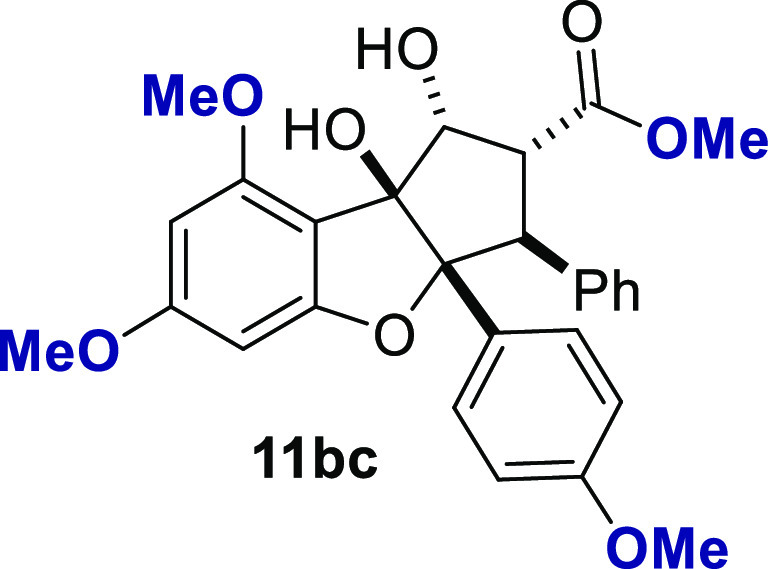



#### 2-(4-(Benzyloxy)-2-methoxy-6-((4-methoxybenzoyl)oxy)phenyl)-2-oxoethyl 4-methoxybenzoate (**5bc**)

A solution of the α-hydroxy ketone **4b** (4.27 g, 14.8 mmol, 1.00 equiv) in CH_2_Cl_2_ (40.0 mL) was treated with 4-DMAP (90.4 mg, 740 μmol, 5 mol %) and triethylamine (6.19 mL, 44.4 mmol, 3.00 equiv). The mixture was cooled to 0 °C and 4-methoxybenzoyl chloride (4.01 mL, 29.6 mmol, 2.00 equiv) was added and stirred at rt for 3 h. The solution was terminated by the addition of HCl (1.00 M in H_2_O.) and the phases were separated. The aqueous phase was extracted with CH_2_Cl_2_ (1x). The combined organic phases were dried over MgSO_4_, filtered and concentrated under reduced pressure. The desired bisbenzoate **5bc** was obtained as a yellow foam (8.24 g) and was used directly for the next step. *R*_f_ = 0.28 (petroleum ether/EtOAc 2:1).

#### 1-(4-(Benzyloxy)-2-hydroxy-6-methoxyphenyl)-3-(4-methoxyphenyl)-1,3-dioxopropan-2-yl 4-methoxybenzoate (**6bc**)

A solution of crude bisbenzoate **5bc** (8.24 g, 14.8 mmol, 1.00 equiv) in THF (80.0 mL) was cooled to −20 °C and treated with LiHMDS (1.00 M in THF, 44.4 mL, 44.4 mmol, 3.00 equiv). The mixture was stirred at −20 °C for 1 h. Then, the reaction was terminated by the addition of NH_4_Cl solution (sat., aq.) and warmed to rt. The aqueous phase was extracted with EtOAc (3×) and the combined organic phases were dried over MgSO_4_, filtered and concentrated under reduced pressure. The desired phenol **6bc** was obtained as a yellow foam (8.24 g) and used directly for the next step. *R*_f_ = 0.30 (petroleum ether/EtOAc 2:1).

#### 7-(Benzyloxy)-5-methoxy-2-(4-methoxyphenyl)-4-oxo-4*H*-chromen-3-yl 4-methoxybenzoate (**7bc**)

A suspension of crude phenol **6bc** (8.24 g, 14.8 mmol, 1.00 equiv) in AcOH (170 mL) was treated with H_2_SO_4_ (96 wt%, 4.11 mL, 74.0 mmol, 5.00 equiv) and stirred at rt for 15 h. The reaction mixture was poured into ice-cold H_2_O and stirred for 15 min. Thereby, a pale-pink precipitate was formed. The mixture was filtered on a Büchner funnel and the precipitate was washed with H_2_O. The wet solid was suspended in a minimal amount of ethanol and heated to reflux for 1 h. The mixture was allowed to cool to rt, filtered on a Büchner funnel and washed with a small amount of cold ethanol. The solid was dried under reduced pressure to constant weight to give the desired 3-benzyloxyflavonate **7bc** as a colorless solid (5.85 g, 10.9 mmol, 73% over three steps). *R*_f_ = 0.28 (petroleum ether/EtOAc 1:2; ^**1**^**H NMR** (CDCl_3_, 400 MHz): δ [ppm] 8.16 (d, *J* = 8.8 Hz, 2H, 2× Ar*H*), 7.87 (d, *J* = 9.0 Hz, 2H, 2× Ar*H*), 7.48–7.38 (m, 5H, 5× Ar*H*), 6.97–6.93 (m, 4H, 4× Ar*H*), 6.63 (d, *J* = 2.2 Hz, 1H, Ar*H*), 6.44 (d, *J* = 2.2 Hz, 1H, Ar*H*), 5.16 (s, 2H, C*H*_2_), 3.90 (s, 3H, OC*H*_3_), 3.88 (s, 3H, OC*H*_3_), 3.83 (s, 3H, OC*H*_3_); ^**13**^**C NMR** (CDCl_3_, 100 MHz): δ [ppm] 170.9 (q, *C*=O), 164.0 (q, Ar*C*), 163.9 (q, Ar*C*), 163.4 (q, O*C*=O), 161.7 (q, Ar*C*), 161.5 (q, Ar*C*), 159.3 (q, Ar*C*), 153.5 (q, *C*=C–C = O), 135.8 (q, Ar*C*), 134.1 (q, O = C-*C*=C), 132.9 (t, 2× Ar*C*), 129.8 (t, 2× Ar*C*), 129.0 (t, 2× Ar*C*), 128.6 (t, Ar*C*H), 127.8 (t, 2× Ar*C*), 122.5 (q, Ar*C*), 121.6 (q, Ar*C*), 114.2 (t, 2× Ar*C*), 113.9 (t, 2× Ar*C*H), 109.2 (q, Ar*C*), 96.7 (t, Ar*C*H), 93.6 (t, Ar*C*H), 70.7 (s, *C*H_2_), 56.4 (p, O*C*H_3_), 55.6 (p, O*C*H_3_), 55.5 (p, O*C*H_3_). The analytical data are consistent with those reported in the literature.^20^

#### 7-(Benzyloxy)-3-hydroxy-5-methoxy-2-(4-methoxyphenyl)-4*H*-chromen-4-one (**8bc**)

A suspension of the benzoate **7bc** (5.85 g, 10.9 mmol, 1.00 equiv) in EtOH (135 mL) was treated with NaOH solution (5 wt% in H_2_O, 15.5 mL, 20.4 mmol, 1.88 equiv). The yellowish suspension was stirred at 80 °C for 1 h. The reaction mixture was allowed to cool to rt and was neutralized with HCl (1.00 M in H_2_O, 20.4 mL, 20.4 mmol, 1.88 equiv). The resulting suspension was filtered on a Büchner funnel and the precipitate was washed with a small amount of cold ethanol. The solid was dried under reduced pressure to constant weight to give the desired 3-hydroxyflavone **8bc** as a yellowish solid (4.05 g, 10.0 mmol, 92%). *R*_f_ = 0.35 (petroleum ether/EtOAc 1:2); ^**1**^**H NMR** (CDCl_3_, 400 MHz): δ [ppm] 8.17 (d, *J* = 8.9 Hz, 2H, 2× Ar*H*), 7.48–7.38 (m, 5H, 5× Ar*H*), 7.36 (bs, 1H, O*H*), 7.03 (d, *J* = 9.0 Hz, 2H, 2× Ar*H*), 6.63 (d, *J* = 1.9 Hz, 1H, Ar*H*), 6.43 (d, *J* = 1.8 Hz, 1H, Ar*H*), 5.15 (s, 2H, C*H*_2_), 3.97 (s, 3H, OC*H*_3_), 3.88 (s, 3H, OC*H*_3_); ^**13**^**C NMR** (CDCl_3_, 100 MHz): δ [ppm] 172.0 (q, *C*=O), 163.5 (q, Ar*C*), 160.8 (q, Ar*C*), 160.7 (q, Ar*C*), 158.9 (q, Ar*C*), 142.4 (q, *C*=COH), 137.6 (q, *C*OH), 135.7 (q, Ar*C*), 129.0 (t, 2× Ar*C*), 128.9 (t, 2× Ar*C*), 128.6 (t, Ar*C*H), 127.8 (t, 2× Ar*C*), 123.7 (q. Ar*C*), 114.1 (t, 2× Ar*C*), 106.5 (q, Ar*C*), 96.3 (t, Ar*C*H), 93.5 (t, Ar*C*H), 70.7 (s. *C*H_2_), 56.6 (p, O*C*H_3_), 55.5 (p, O*C*H_3_). The analytical data are consistent with those reported in the literature.^20^

#### (±)-Methyl (1*R*,2*R*,3*S*,3a*R*,8b*S*)-6-(benzyloxy)-1,8b-dihydroxy-8-methoxy-3a-(4-methoxyphenyl)-3-phenyl-2,3,3a,8b-tetrahydro-1*H*-cyclopenta[*b*]benzofuran-2-carboxylate (**9bc**)

Methyl cinnamate (6.14 g, 37.9 mmol, 14.2 equiv) was added to a solution of flavonol **102** (1.01 g, 2.67 mmol, 1.00 equiv) in dry chloroform (51.2 mL) and freshly distilled 2,2,2-trifluoroethanol (22.0 mL). The reaction mixture was degassed for 30 min, then cooled to −5 °C and irradiated with UV light (λ_max_ = 365 nm) until it no longer fluoresced greenish (20 h). Subsequently, the solvent was removed under reduced pressure and the remaining amount of methyl cinnamate was removed by column chromatography (petroleum ether/EtOAc 4:1 → 1:1). The desired cycloadduct was obtained as a mixture of isomers as a yellowish foam (1.37 g). Without any further purification the product of the first step (1.37 g, 2.41 mmol, 1.00 equiv) was dissolved in MeOH (80.0 mL). Then, NaOMe solution (25 wt% in MeOH, 1.10 mL, 6.85 mmol, 2.84 equiv) was added and the mixture was heated under refluxing conditions for 1 h. Subsequently, the reaction was terminated by the addition of NH_4_Cl solution (sat., aq.). The phases were separated and the aqueous phase was extracted with EtOAc (3×). The organic phases were combined, dried over MgSO_4_, filtered and concentrated under reduced pressure. The desired keto ester was obtained as a mixture of isomers as a yellow, glassy foam (1.33 g). About half of the product (697 mg) was used for the next step without further purification. A mixture of (CH_3_)_4_N(OAc)_3_BH (2.08 g, 7.90 mmol, 6.42 equiv) and freshly distilled AcOH (732 μL, 12.8 mmol, 10.4 equiv) in MeCN (32.0 mL) was stirred for 5 min at rt. Then, a solution of the product of the second step (697 mg, 1.23 mmol, 1.00 equiv) in MeCN (21.3 mL) was added. The mixture was protected from light and stirred for 19 h at rt. The reaction was then terminated by adding NH_4_Cl solution (sat., aq.) and sodium potassium tartrate solution (aq., 2.00 M). The phases were separated and the aqueous layer was extracted with CH_2_Cl_2_ (3×). The combined organic layers were dried over MgSO_4_, filtered and concentrated under reduced pressure. Column chromatography (petroleum ether/EtOAc 3:2) was then performed to obtain the racemic *endo*-product **9bc** as a pale-yellow solid (423 mg, 744 μmol, 56% yield over three steps). *R*_f_ = 0.63 (petroleum ether/EtOAc 1:2); ^**1**^**H NMR** (CDCl_3_, 400 MHz): δ [ppm] 7.47–7.35 (m, 5H, *H*-3‴, *H*-4‴, *H*-5‴, *H*-6‴, *H*-7‴), 7.11 (d, *J* = 8.9 Hz, 2H, *H*-2′, *H*-6′), 7.08–7.05 (m, 3H, *H*-3″, *H*-4″, *H*-5″), 6.88–6.86 (m, 2H, *H*-2″, *H*-6″), 6.68 (d, *J* = 8.9 Hz, 2H, *H*-3′, *H*-5′), 6.36 (d, *J* = 1.9 Hz, 1H, *H*-5), 6.22 (d, *J* = 1.9 Hz, 1H, *H*-7), 5.09 (s, 2H, *H*-1‴), 5.03 (dd, *J* = 6.7, 1.6 Hz, 1H, *H*-1), 4.31 (d, *J* = 14.2 Hz, 1H, *H*-3), 3.90 (dd, *J* = 14.4, 6.5 Hz, 1H, *H*-2), 3.86 (s, 3H, C*H*_3_O-8), 3.71 (s, 3H, C*H*_3_O-4′), 3.67 (br, 1H, O*H*-8b), 3.65 (s, 3H, C*H*_3_O-11), 1.77 (s, 1H, O*H*-1); ^**13**^**C NMR** (CDCl_3_, 100 MHz): δ [ppm] 170.7 (q, *C*-11), 163.4 (q, *C*-6), 161.0 (q, *C*-4a), 158.9 (q, *C*-4′), 157.1 (q, *C*-8), 137.0 (q, *C*-1″), 136.6 (q, *C*-2‴), 129.1 (t, *C*-3′, *C*-5′), 128.8 (t, *C*-4‴, *C*-6‴), 128.3 (t, *C*-5‴), 128.0 (t, *C*-3″, *C*-5″), 127.9 (t, *C*-2″, *C*-6″), 127.7 (t, *C*-3‴, *C*-7‴), 126.7 (t, *C*-4″), 126.5 (q. *C*-1′), 112.9 (t, *C*-3′, *C*-5′), 108.1 (q, *C*-8a), 102.0 (q, *C*-3a), 93.8 (q, *C*-8b), 93.5 (t, *C*-7), 90.6 (t, *C*-5), 79.7 (t, *C*-1), 70.6 (s, *C*-1‴), 55.9 (p, H_3_*C*O-8), 55.3 (p, H_3_*C*O-4′), 55.1 (t, *C*-3), 52.1 (p, H_3_*C*O-11), 50.6 (t, *C*-2); **HRMS (ESI**^**+**^**)***m*/*z* calcd for C_34_H_32_O_8_Na [M+Na]^+^ 591.1995, found 591.1987. The analytical data are consistent with those reported in the literature.^20^

#### (±)-Methyl (1*R*,2*R*,3*S*,3a*R*,8b*S*)-1,6,8b-trihydroxy-8-methoxy-3a-(4-methoxyphenyl)-3-phenyl-2,3,3a,8b-tetrahydro-1*H*-cyclopenta[*b*]benzofuran-2-carboxylate (**10bc**)

Palladium-on-carbon (10 wt%, 58.8 mg, 55.2 μmol, 10 mol %) was added to a solution of benzyl ether **9bc** (314 mg, 552 μmol, 1.00 equiv) in dry THF (5.52 mL) under an argon atmosphere. The atmosphere was replaced by hydrogen and an additional balloon of hydrogen was placed on the flask. The reaction mixture was stirred for 200 min at rt and then filtered over Celite. The filtrate was concentrated to dryness and gave the desired phenol **10bc** as a colorless foam (255 mg, 533 μmol) in 97% yield. *R*_f_ = 0.16 (petroleum ether/EtOAc 1:1); ^**1**^**H NMR** (acetone-*d*_6_, 400 MHz): δ [ppm] 8.61 (s, 1H, O*H*-6), 7.12 (d, *J* = 9.0 Hz, 2H, *H*-2′, *H*-6′), 7.06–6.92 (m, 3H, *H*-3″, *H*-4″, *H*-5″), 6.92–6.90 (m, 2H, *H*-2″, *H*-6″), 6.63 (d, *J* = 9.0 Hz, 2H, *H*-3′, *H*-5′), 6.16 (d, *J* = 1.9 Hz, 1H, *H*-5), 6.11 (d, *J* = 1.8 Hz, 1H, *H*-7), 4.93 (dd, *J* = 6.4, 2.8 Hz, 1H, *H*-1), 4.28 (d, *J* = 14.1 Hz, 1H, *H*-3), 3.97 (s, 1H, O*H*-8b), 3.94 (ddd, *J* = 14.1, 6.6, 0.8 Hz, 1H, *H*-2), 3.83 (s, 3H, C*H*_3_O-4′), 3.66 (s, 3H, C*H*_3_O-8), 3.56 (s, 3H, C*H*_3_O-11); ^**13**^**C NMR** (acetone-*d*_6_, 100 MHz): δ [ppm] 170.8 (q, *C*-11), 162.1 (q, *C*-6), 161.8 (q, *C*-4a), 159.3 (q, *C*-4′), 158.7 (q, *C*-8), 139.2 (q, *C*-1″), 130.0 (t, C-2′, C-6′), 128.9 (q, *C*-1′), 128.8 (t, *C*-3″, *C*-5″), 128.2 (t, *C*-2″, *C*-6″), 126.8 (t, *C*-4″), 112.8 (t, *C*-3′, *C*-5′), 108.4 (q, *C*-8a), 102.6 (q, *C*-3a), 94.5 (q, *C*-8b), 93.2 (t, *C*-7), 91.9 (t, *C*-5), 80.8 (t, *C*-1), 55.9 (p, H_3_*C*O-8), 55.7 (t, *C*-3), 55.2 (p, H_3_*C*O-4′), 52.6 (p, H_3_*C*O-11), 51.2 (t, *C*-2). The analytical data are consistent with those reported in the literature.^42^

#### (±)-Methyl (1*R*,2*R*,3*S*,3a*R*,8b*S*)-1,8b-dihydroxy-6,8-dimethoxy-3a-(4-methoxyphenyl)-3-phenyl-2,3,3a,8b-tetrahydro-1*H*-cyclopenta[*b*]benzofuran-2-carboxylate (**11bc**)

A solution of the phenol **10bc** (75.0 mg, 157 μmol, 1.00 equiv) in toluene (5.22 mL) and methanol (5.22 mL) was treated with trimethylsilyldiazomethane (2.00 M in Et_2_O, 1.25 mL, 2.51 mmol, 16.0 equiv) and stirred for 150 min at rt. The solvents were removed under reduced pressure. The residue was purified using silica gel chromatography (petroleum ether/EtOAc 6:4) to give the desired rocaglate **11bc** as a pale-yellow foam (70.0 mg, 142 μmol) in 91% yield. *R*_f_ = 0.34 (petroleum ether/EtOAc 2:3); ^**1**^**H NMR** (DMSO-*d*_6_, 400 MHz): δ [ppm] 7.06–6.96 (m, 5H, *H*-2′, *H*-6′, *H*-3″, *H*-4″, *H*-5″), 6.87 (d, *J* = 7.4 Hz, 2H, *H*-2″, *H*-6″), 6.59 (d, *J* = 8.6 Hz, 2H, *H*-3′, *H*-5′), 6.28 (bs, 1H, *H*-5), 6.11 (bs, 1H, *H*-7), 5.07 (s, 1H, O*H*-8b), 5.01 (d, *J* = 4.4 Hz, 1H, O*H*-1), 4.69 (t, *J* = 4.9 Hz, 1H, *H*-1), 4.14 (d, *J* = 14.0 Hz, 1H, *H*-3), 3.91 (dd, *J* = 14.0, 5.5 Hz, 1H, *H*-2), 3.78 (p, *H*_3_CO-6), 3.73 (p, *H*_3_CO-8), 3.60 (s, 3H, *H*_3_CO-4′), 3.54 (s, 3H, *H*_3_CO-11); ^**13**^**C NMR** (DMSO-*d*_6_, 100 MHz): δ [ppm] 170.3 (q, *C*-11), 162.7 (q, *C*-6), 160.4 (q, *C*-4a), 157.8 (q, *C*-8), 157.5 (q, *C*-4′), 138.3 (q, *C*-1″), 128.7 (t, C-2′, C-6′), 128.5 (q. *C*-1′), 127.7 (t, *C*-3″, *C*-5″), 127.4 (t, C-2″, C-6″), 125.8 (t, C-4″), 111.8 (t, *C*-3′, *C*-5′), 108.3 (q, *C*-8a), 101.3 (q, *C*-3a), 93.2 (q, *C*-8b), 91.8 (t, *C*-7), 88.4 (t, *C*-5), 78.9 (t, *C*-1), 55.5 (p, H_3_*C*O-6), 55.3 (p, H_3_*C*O-8), 54.7 (p, H_3_*C*O-4′), 54.6 (t, *C*-3), 51.3 (p, H_3_*C*O-11), 50.6 (t, *C*-2); **HRMS (ESI**^**+**^**)***m*/*z* calcd for C_28_H_28_O_8_Na [M+Na]^+^ 515.1682, found 515.1681. **HPLC purity** 98.15%: The analytical data are consistent with those reported in the literature.^43^

### Synthesis of (±)-Methyl (1*R*,2*R*,3*S*,3a*R*,8b*S*)-6,8-difluoro-1,8b-dihydroxy-3a-(4-methoxyphenyl)-3-phenyl-2,3,3a,8b-tetrahydro-1*H*-cyclopenta[*b*]benzofuran-2-carboxylate (**9c**)

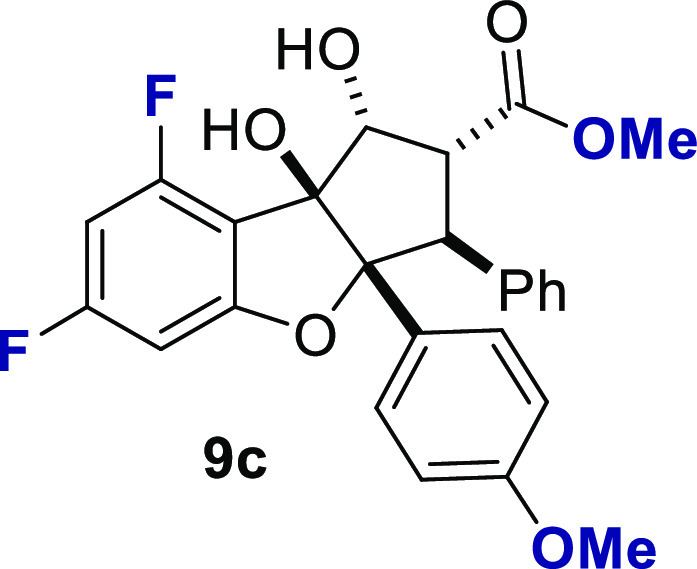



#### (*E*)-1-(2,4-Difluoro-6-hydroxyphenyl)-3-(4-methoxyphenyl)prop-2-en-1-one (**12c**)

A solution of NaOEt (378 mg, 5.56 mmol, 3.00 equiv) in EtOH (6 mL) was prepared and cooled down to rt. To this solution was added 1-(2,4-difluoro-6-hydroxyphenyl)ethan-1-one (319 mg, 1.85 mmol) and stirred for 1 h at rt. To the yellow solution was added *p*-methoxybenzaldehyde (0.23 mL, 1.85 mmol, 1.00 equiv) and stirred for 16 h at rt. The suspension was then poured to water and acidified to pH = 1 with HCl solution (aq., 1 M). The resulting yellow precipitate was filtered, washed with cold water and dried under high vacuum. The desired product chalcone **12c** was afforded (502 mg, 1.67 mmol, 89%) as a yellow solid. *R*_f_ = 0.40 (petroleum ether/EtOAc 3:1); ^**1**^**H NMR** (CDCl_3_, 400 MHz): δ [ppm] 13.72 (s, 1H, O*H*), 7.94 (dd, *J* = 15, 3.5 Hz, 1H, C(O)CH=C*H*), 7.61 (d, *J* = 8.7 Hz, 2H, 2× Ar*H*), 7.50 (dd, *J* = 15, 1.9 Hz, 1H, C(O)C*H*=CH), 6.95 (d, *J* = 8.7 Hz, 2H, 2× Ar*H*), 6.52 (ddd, *J* = 10, 2.3, 1.7 Hz, 1H, Ar*H*), 6.40 (ddd, *J* = 12, 9.1, 2.7 Hz, 1H, Ar*H*), 3.87 (s, 3H, OC*H*_3_); ^**13**^**C NMR** (CDCl_3_, 100 MHz): δ [ppm] 191.4 (q, *d*, *J* = 4.9 Hz, *C*=O), 167.9 (q, *d*, *J* = 18 Hz, Ar*C*), 165.3 (q, *d*, *J* = 18 Hz, Ar*C*), 162.9 (q, *d*, *J* = 17 Hz, Ar*C*), 162.2 (q, Ar*C*), 146.2 (t, *d*, *J* = 2.0 Hz, C(O)CH=*C*H), 130.9 (t, 2× Ar*C*), 127.3 (q, Ar*C*), 122.3 (C(O)*C*H=CH), 114.5 (t, 2× Ar*C*), 107.7 (q, dd, *J* = 14, 3.2 Hz, Ar*C*) 101.5 (t, dd, *J* = 23, 3.7 Hz, Ar*C*), 95.9 (t, dd, *J* = 30, 27 Hz, Ar*C*), 55.5 (p, O*C*H_3_); **HRMS (ESI**^**+**^**)***m*/*z* calcd for C_16_H_12_F_2_O_3_ [M+H]^+^ 291.0833, found 291.0838.

#### 5,7-Difluoro-3-hydroxy-2-(4-methoxyphenyl)-4*H*-chromen-4-one (**8c**)

Chalcone **12c** (495 mg, 2.69 mmol) was dissolved in MeOH (32 mL) and NaOH solution (aq., 30 wt%, 4.48 mL, 13.4 mmol, 5.00 equiv) and cooled down to 0 °C. To the dark orange solution was added H_2_O_2_ (aq., 30%, 0.62 mL, 26.9 mmol, 10.0 equiv). The thick yellow suspension was stirred at 0 °C for 30 min, warmed to rt and continued stirring for 16 h. After the chalcone was fully consumed, the reaction mixture was poured into HCl solution (aq., 1 M) and extracted with CH_2_Cl_2_. The collected organic layers were washed with brine, dried over MgSO_4_, filtered and concentrated *in vacuo*. The crude product was recrystallized from EtOH to afford clean product **8c** (221 mg, 0.69 mmol, 26%) as yellow crystals/solids. *R*_f_ = 0.20 (petroleum ether/EtOAc 3:1); ^**1**^**H NMR** (CDCl_3_, 400 MHz): δ [ppm] 8.17 (d, *J* = 9.1 Hz, 2H, 2× Ar*H*), 7.08 (dt, 1H, *J* = 9.1, 1.9 Hz, Ar*H*), 7.05 (d, *J* = 9.1 Hz, 2H, 2× Ar*H*), 6.88–6.83 (m, 1H, Ar*H*), 3.89 (s, 3H, OC*H*_3_); ^**13**^**C NMR** (CDCl_3_, 100 MHz): δ [ppm] 170.7 (q, *C*=O), 164.9 (q, dd, *J* = 255, 14 Hz, Ar*C*), 161.4 (q, dd, *J* = 267, 15 Hz, Ar*C*), 161.3 (q, Ar*C*), 156.8 (q, dd, *J* = 16, 6.5 Hz, Ar*C*), 144.8 (q, *C*OH), 137.6 (q, *C*=COH), 130.1 (q, *d*, *J* = 246 Hz, 1C, Ar*C*), 129.4 (t, 2× Ar*C*), 122.7 (q, Ar*C*), 114.2 (t, 2× Ar*C*), 101.3 (t, dd, *J* = 27, 24 Hz, Ar*C*), 101.1 (t, dd, *J* = 25, 5 Hz, Ar*C*), 55.5 (p, O*C*H_3_); **HRMS (ESI**^**+**^**)***m*/*z* calcd for C_16_H_12_F_2_O_3_Na [M+Na]^+^ 327.0445, found 327.0430.

#### (±)-Methyl (1*R*,2*R*,3*S*,3a*R*,8b*S*)-6,8-difluoro-1,8b-dihydroxy-3a-(4-methoxyphenyl)-3-phenyl-2,3,3a,8b-tetrahydro-1*H*-cyclopenta[*b*]benzofuran-2-carboxylate (**9c**)

To a solution of **8b** (210 mg, 0.69 mmol, 1.00 equiv) in dry 2,2,2-TFE (5.8 mL) and dry CHCl_3_ (14 mL) was added methyl cinnamate (1.59 g, 9.80 mmol, 14.2 equiv). The clear solution was degassed with argon for 15 min, followed by UV-irradiation (100 W, 365 nm) at −5 °C for 10–16 h. After the reaction was finished, the solvent was removed *in vacuo* and the excess of methyl cinnamate was removed by silica gel purification (petroleum ether/EtOAc 4:1, then EtOAc). The cycloadduct mixture was used directly for the next step. To the solution of crude cycloadduct (309 mg) in MeOH (22 mL) was added NaOMe solution (25 wt% in MeOH, 406 μL, 1.88 mmol, 2.84 equiv) and stirred under refluxing conditions for 1 h. The reaction was terminated by the addition of NH_4_Cl (sat., aq.). The aqueous layers were extracted with EtOAc and the collected organic layers were washed with NaCl (sat., aq.), dried over MgSO_4_, filtered and concentrated *in vacuo*. The foamy ketone crude product was directly used for the next step. A solution of Me_4_NBH(OAc)_3_ (467 mg, 1.78 mmol, 6.42 equiv) and freshly distilled AcOH (167 μL, 2.88 mmol, 10.4 equiv) in dry MeCN (7 mL) was prepared and stirred at rt for 10 min. To this solution was added ketone crude product (129 mg) in dry MeCN (4.5 mL). The reaction was carried out under light exclusion and stirred for 19 h at rt. The reaction was terminated by the addition of NaK-tartrate (sat., aq.) and NH_4_Cl (sat., aq.). The layers were separated and the aqueous layers were extracted with CH_2_Cl_2_. The collected organic layers were washed with water and NaCl (sat., aq.), dried over MgSO_4_, filtered and concentrated *in vacuo*. The crude product was purified by silica gel column chromatography (CH_2_Cl_2_/EA = 10:1) to yield **9c** (56 mg, 0.12 mmol, 42%) as a pale-yellow foam. *R*_f_ = 0.54 (petroleum ether/EtOAc 1:1); ^**1**^**H NMR** (DMSO-*d*_6_, 400 MHz): δ [ppm] 7.11–7.05 (m, 5H, *H*-2″, *H*-3″, *H*-4″, *H*5″, *H*-6″), 6.99 (d, *J* = 6.8 Hz, 2H, *H*-2′, *H*-6′), 6.66 (d, *J* = 8.9 Hz, 2H, *H*-3′, *H*-5′), 6.61 (dd, *J* = 8.9, 1.2 Hz, 1H, *H*-5), 6.46 (td, *J* = 9.0, 2.0 Hz, 1H, *H*-7), 4.91 (d, *J* = 5.2 Hz, 1H, *H*-1), 4.47 (d, *J* = 14.0 Hz, 1H, *H*-3), 4.00 (dd, *J* = 14.0, 5.3 Hz, 1H, *H*-2), 3.70 (s, 3H, C*H*_3_O-4′), 3.69 (s, 3H, C*H*_3_O-11); ^**13**^**C NMR** (DMSO-*d*_6_, 100 MHz): δ [ppm] 170.5 (q, *C*-11), 164.3 (q, dd, *J* = 244, 14 Hz, *C*-6), 161.0 (q, dd, *J* = 16, 12 Hz, *C*-4a), 160.2 (q, dd, *J* = 252, 16 Hz, *C*-8), 158.2 (q, *C*-4′), 138.2 (q, C-1″), 129.1 (t, C-3″, C-5″), 128.5 (q, *C*-1′), 128.1 (t, *C*-2″, *C*-6″), 127.9 (t, *C*-3′, *C*-5′), 126.4 (t, *C*-4″), 113.1 (q, dd, *J* = 20, 3.1 Hz, *C*-8a), 112.5 (2C, C-2′, C-5′), 102.8 (q, *C*-3a), 96.8 (t, *t*, *J* = 26 Hz, *C*-7), 95.0 (t, dd, *J* = 26, 3.8 Hz, 1C, *C*-5), 93.5 (q, *d*, *J* = 2.5 Hz, 1C, *C*-8b), 78.8 (t, *C*-1), 55.3 (C-3), 55.2 (C-7′), 51.9 (−CO_2_*C*H_3_), 51.6 (C-2); **HRMS (ESI**^**+**^**)***m*/*z* calcd for C_26_H_22_O_6_F_2_Na [M+Na]^+^ 491.1282, found 491.1279; **HPLC purity** 95.26%.

### Synthesis of (±)-Methyl (1*R*,2*R*,3*S*,3a*R*,8b*S*)-6,8-dichloro-1,8b-dihydroxy-3a-(4-methoxyphenyl)-3-phenyl-2,3,3a,8b-tetrahydro-1*H*-cyclopenta[*b*]benzofuran-2-carboxylate (**9da**)

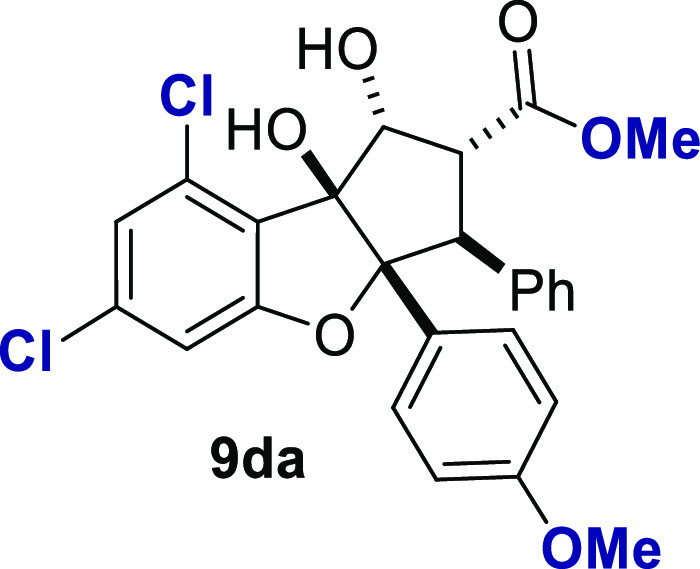



#### (*E*)-1-(2,4-Dichloro-6-hydroxyphenyl)-3-(4-methoxyphenyl)prop-2-en-1-one (**12da**)

Acetophenone **3d** (500 mg, 2.44 mmol, 1.00 equiv) was added to a solution of NaOEt (498 mg, 7.32 mmol, 3.00 equiv) in EtOH (8.41 mL). After stirring for 1 h at rt, 4-methoxybenzaldehyde (296 μL, 2.44 mmol, 1.00 equiv) was added and the reaction mixture was stirred overnight. The resulting yellow suspension was poured into H_2_O and acidified to pH = 1 with HCl (10 wt% in H_2_O). The yellow precipitate was filtered, washed with H_2_O and dried under reduced pressure. The desired chalcone **12da** was obtained as a yellow solid (744 mg, 2.30 mmol, 94%). *R*_f_ = 0.43 (petroleum ether/EtOAc 3:1); ^**1**^**H NMR** (CDCl_3_, 400 MHz): δ [ppm] 11.58 (s, 1H, O*H*), 7.82 (d, *J* = 15.5 Hz, 1H, C(O)CH=C*H*), 7.60 (d, *J* = 8.7 Hz, 2H, 2× Ar*H*), 7.51 (d, *J* = 15.4 Hz, 1H, C(O)C*H*), 7.01–6.94 (m, 4H, 4× Ar*H*), 3.87 (s, 3H, OC*H*_3_); ^**13**^**C NMR** (CDCl_3_, 100 MHz): δ [ppm] 193.6 (q, *C*=O), 162.8 (q, Ar*C*), 162.4 (q, Ar*C*), 144.9 (t, C(O)CH=*C*H), 139.7 (q, Ar*C*), 134.7 (q, Ar*C*), 130.9 (t, 2× Ar*C*H), 127.4 (q, Ar*C*), 123.6 (t, C(O)*C*H), 122.3 (t, Ar*C*H), 120.3 (q, Ar*C*), 117.3 (t, Ar*C*H), 114.7 (t, 2× Ar*C*H), 55.6 (p, O*C*H_3_). The analytical data are consistent with those reported in the literature.^44^

#### 5,7-Dichloro-3-hydroxy-2-(4-methoxyphenyl)-4*H*-chromen-4-one (**8da**)

To a suspension of chalcone **12da** (646 mg, 2.00 mmol, 1.00 equiv) in MeOH (17.2 mL), NaOH (3.00 M, aq., 2.58 mL, 7.74 mmol, 3.87 equiv) was added and cooled to 0 °C. H_2_O_2_ (30 wt% in H_2_O, 652 μL, 6.40 mmol, 3.20 equiv) was then added dropwise and the solution was stirred at 0 °C for 3 h. Subsequently, the cooling bath was removed and the mixture was stirred for another 20 h. Then, HCl (10 wt% in H_2_O) was added, leading to the formation of a yellow precipitate. The suspension was then extracted with CH_2_Cl_2_ (4×). The combined organic layers were dried over MgSO_4_, filtered and concentrated under reduced pressure. The crude material was purified by recrystallization from EtOAc to give the desired product **8da** as a pale-yellowish solid (172 mg, 510 μmol, 26%). *R*_f_ = 0.42 (petroleum ether/EtOAc 4:1); ^**1**^**H NMR** (CDCl_3_, 400 MHz): δ [ppm] 8.18 (d, *J* = 9.0 Hz, 2H, 2× Ar*H*), 7.53 (d, *J* = 1.8 Hz, 1H, Ar*H*), 7.40 (d, *J* = 1.8 Hz, 1H, Ar*H*), 7.17 (s, 1H, O*H*), 7.05 (d, *J* = 9.0 Hz, 2H, 2× Ar*H*), 3.90 (s, 3H, OC*H*_3_); ^**13**^**C NMR** (CDCl_3_, 100 MHz): δ [ppm] 171.7 (q, *C*=O), 161.5 (q, Ar*C*), 156.6 (q, Ar*C*), 144.2 (q, *C*=COH), 138.6 (q, Ar*C*), 138.2 (q, *C*OH), 134.5 (q, Ar*C*), 129.5 (t, 2× Ar*C*H), 127.6 (t, Ar*C*H), 122.7 (q, Ar*C*), 117.6 (t, Ar*C*H), 116.6 (q, Ar*C*), 114.4 (t, 2× Ar*C*H), 55.6 (p, O*C*H_3_); **HRMS (EI)***m*/*z* calcd for C_16_H_10_Cl_2_O_4_ [M]^+^ 335.9956, found 335.9971.

#### (±)-Methyl (1*R*,2*R*,3*S*,3a*R*,8b*S*)-6,8-dichloro-1,8b-dihydroxy-3a-(4-methoxyphenyl)-3-phenyl-2,3,3a,8b-tetrahydro-1*H*-cyclopenta[*b*]benzofuran-2-carboxylate (**9da**)

Methyl cinnamate (1.14 g, 7.03 mmol, 14.2 equiv) was added to a solution of flavonol **8da** (167 mg, 495 μmol, 1.00 equiv) in dry chloroform (9.71 mL) and freshly distilled 2,2,2-trifluoroethanol (4.13 mL). The reaction mixture was degassed for 30 min, then cooled to −5 °C and irradiated with UV light (λ_max_ = 365 nm) until it no longer fluoresced greenish (20 h). Subsequently, the solvent was removed under reduced pressure. The remaining amount of methyl cinnamate was then removed by column chromatography (petroleum ether/EtOAc 9:1 → 1:1). The desired cycloadduct was obtained as a mixture of isomers as a yellowish foam (185 mg). Without any further purification the product of the first step (185 mg, 370 μmol, 1.00 equiv) was dissolved in MeOH (13.7 mL). Then, NaOMe solution (200 μL, 25 wt% in MeOH, 1.20 mmol, 3.25 equiv) was added and the mixture was heated under refluxing conditions for 1 h. Subsequently, the reaction was terminated by the addition of NH_4_Cl solution (sat., aq.). The phases were separated and the aqueous phase was extracted with EtOAc (3×). The organic phases were combined, dried over MgSO_4_, filtered and the solvent was removed under reduced pressure. The desired keto ester was obtained as a mixture of isomers as an orange solid (185 mg) and used directly for the next step. A mixture of (CH_3_)_4_N(OAc)_3_BH (626 mg, 2.38 mmol, 6.42 equiv) and freshly distilled AcOH (221 μL, 3.86 mmol, 10.4 equiv) in MeCN (9.62 mL) was stirred for 5 min at rt. Then, a solution of the product of the second step (185 mg, 370 μmol, 1.00 equiv) in MeCN (6.39 mL) was added. The mixture was protected from light and stirred for 19 h at rt. The reaction was then terminated by adding NH_4_Cl solution (sat., aq.) and sodium potassium tartrate solution (aq., 2.00 M). The phases were separated and the aqueous layer was extracted with CH_2_Cl_2_ (3×). The combined organic layers were dried over MgSO_4_, filtered and concentrated under reduced pressure. Column chromatography (CH_2_Cl_2_/EtOAc 1:0 → 9:1) was then performed to obtain the racemic *endo*-product **9da** as a colorless foam (119 mg, 237 μmol, 48% over three steps). *R*_f_ = 0.21 (petroleum ether/EtOAc 7:3); ^**1**^**H NMR** (DMSO-*d*_6_, 400 MHz): δ [ppm] 7.14 (d, *J* = 1.7 Hz, 1H, *H*-5), 7.07–6.95 (m, 8H, *H*-7, *H*-2′, *H*-6′, *H*-2″, *H*-3″, *H*-4″, *H*-5″, *H*-6″), 6.57 (d, *J* = 9.0 Hz, 2H, *H*-3′, *H*-5′), 5.72 (d, *J* = 6.1 Hz, 1H, O*H*-1), 5.69 (s, 1H, O*H*-8b), 4.69 (dd, *J* = 5.8, 4.6 Hz, 1H, *H*-1), 4.38 (d, *J* = 14.0 Hz, 1H, *H*-3), 4.06 (dd, *J* = 14.0, 4.5 Hz, 1H, *H*-2), 3.59 (s, 3H, *H*_3_CO-11), 3.58 (s, 3H, *H*_3_CO-4″); ^**13**^**C NMR** (DMSO-*d*_6_, 100 MHz): δ [ppm] 170.2 (q, *C*-11), 160.6 (q, *C*-4a), 157.6 (q, *C*-4′), 138.0 (q, *C*-1″), 134.3 (q, *C*-6), 132.5 (q, *C*-8a), 128.5 (t, *C*-2′, *C*-6′), 128.0 (q, *C*-1′), 127.9 (t, *C*-3″, *C*-5″), 127.5 (t, *C*-2″, *C*-6″), 125.8 (t, *C*-4″), 125.6 (q, *C*-8), 120.9 (t, *C*-7), 111.9 (t, *C*-3′, *C*-5′), 109.2 (t, *C*-5), 102.3 (q, *C*-3a), 93.5 (q, *C*-8b), 78.2 (t, *C*-1), 54.9 (t, *C*-3), 54.7 (p, H_3_*C*O-4′), 51.7 (t, *C*-2), 51.5 (p, H_3_*C*O-11); **HRMS (ESI**^**+**^**)***m*/*z* calcd for C_26_H_22_Cl_2_O_6_Na [M+Na]^+^, 523.0691 found 523.0676. **HPLC purity** 98.31%.

### Synthesis of (±)-Methyl (1*R*,2*R*,3*S*,3a*R*,8b*S*)-3a-(4-bromophenyl)-6,8-dichloro-1,8b-dihydroxy-3-phenyl-2,3,3a,8b-tetrahydro-1*H*-cyclopenta[*b*]benzofuran-2-carboxylate (**9db**)

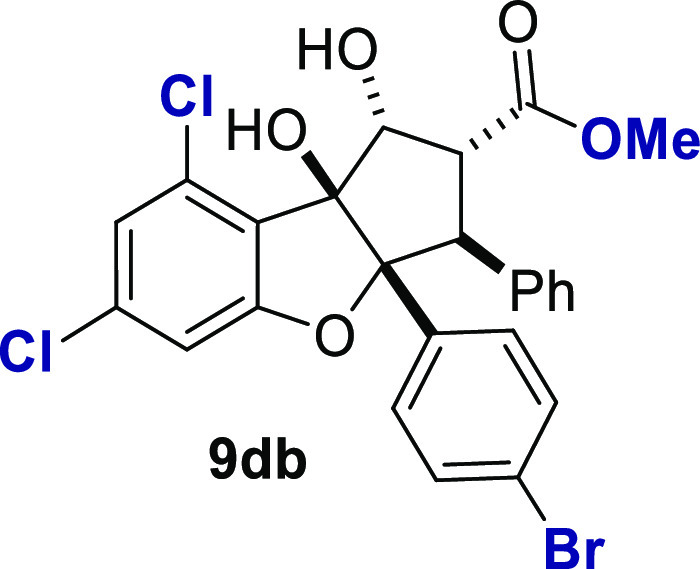



#### (*E*)-3-(4-Bromophenyl)-1-(2,4-dichloro-6-hydroxyphenyl)prop-2-en-1-one (**12db**)

Acetophenone **3d** (500 mg, 2.44 mmol, 1.00 equiv) was added to a solution of NaOEt (498 mg, 7.32 mmol, 3.00 equiv) in EtOH (8.41 mL). After stirring for 1 h at rt, 4-bromobenzaldehyde (451 mg, 2.44 mmol, 1.00 equiv) was added and the reaction mixture was stirred overnight. The resulting yellow suspension was poured into H_2_O and acidified to pH = 1 with HCl (10 wt% in H_2_O). The yellow precipitate was filtered, washed with H_2_O and dried under reduced pressure. The desired compound **12db** was obtained as a yellow solid (856 mg, 2.30 mmol, 94%). *R*_f_ = 0.57 (petroleum ether/EtOAc 3:1); ^**1**^**H NMR** (CDCl_3_, 400 MHz): δ [ppm] 11.51 (s, 1H, O*H*), 7.73 (d, *J* = 15.6 Hz, 1H, C(O)CH=C*H*), 7.61 (d, *J* = 15.6 Hz, 1H, C(O)C*H*), 7.57 (d, *J* = 8.4 Hz, 2H, 2× Ar*H*), 7.48 (d, *J* = 8.4 Hz, 2H, 2× Ar*H*), 7.02 (d, *J* = 2.0 Hz, 1H, Ar*H*), 6.98 (d, *J* = 2.0 Hz, 1H, Ar*H*); ^**13**^**C NMR** (CDCl_3_, 100 MHz): δ [ppm] 193.7 (q, *C*=O), 163.0 (q, Ar*C*), 143.1 (t, C(O)CH=*C*H), 140.3 (q, Ar*C*), 134.8 (q, Ar*C*), 133.5 (q, Ar*C*), 132.51 (t, 2× Ar*C*H), 130.2 (t, 2× Ar*C*H), 126.5 (t, C(O)*C*H), 125.6 (q, ArC), 122.5 (t, ArC*H*), 119.9 (q, Ar*C*), 117.5 (t, Ar*C*H); **HRMS (ESI**^–^**)***m*/*z* calcd for C_15_H_8_BrCl_2_O_2_ [M–H]^−^ 368.9085, found 368.9085.

#### 2-(4-Bromophenyl)-5,7-dichloro-3-hydroxy-4*H*-chromen-4-one (**8db**)

To a suspension of chalcone **12db** (744 mg, 2.00 mmol, 1.00 equiv) in MeOH (17.2 mL), NaOH (3.00 M, aq., 2.58 mL, 7.74 mmol, 3.87 equiv) was added and cooled to 0 °C. H_2_O_2_ (30 wt% in H_2_O, 652 μL, 6.40 mmol, 3.20 equiv) was then added dropwise and the solution was stirred at 0 °C for 3 h. Subsequently, the cooling bath was removed and the mixture was stirred for another 20 h. Then, HCl (10 wt% in H_2_O) was added, leading to the formation of a yellow precipitate. Subsequently, the suspension was extracted with CH_2_Cl_2_ (4×). The combined organic layers were dried over MgSO_4_, filtered and concentrated under reduced pressure. The crude material was purified by recrystallization from EtOAc to give the desired product **8db** as a pale-yellowish solid (155 mg, 402 μmol, 20%). *R*_f_ = 0.57 (petroleum ether/EtOAc 4:1); ^**1**^**H NMR** (CDCl_3_, 400 MHz): δ [ppm] 8.09 (d, *J* = 8.4 Hz, 2H, 2× Ar*H*), 7.67 (d, *J* = 8.2 Hz, 2H, 2× Ar*H*), 7.56 (s, 1H, Ar*H*), 7.43 (s, 1H, Ar*H*); ^**13**^**C NMR** (CDCl_3_, 100 MHz): δ [ppm] 171.9 (q, *C*=O), 156.7 (q, Ar*C*), 142.7 (q, *C*=COH), 139.2 (q, Ar*C*), 139.0 (q, *C*OH), 134.7 (q, Ar*C*), 132.2 (t, 2× Ar*C*H), 129.3 (q, Ar*C*), 129.1 (t, 2× Ar*C*H), 127.8 (t, Ar*C*H), 125.3 (q, Ar*C*), 117.6 (t, Ar*C*H), 116.5 (q, Ar*C*); **HRMS (EI)***m*/*z* calcd for C_15_H_7_BrCl_2_O_3_ [M]^+^ 335.9956, found 335.8955.

#### (±)-Methyl (1*R*,2*R*,3*S*,3a*R*,8b*S*)-3a-(4-bromophenyl)-6,8-dichloro-1,8b-dihydroxy-3-phenyl-2,3,3a,8b-tetrahydro-1*H*-cyclopenta[*b*]benzofuran-2-carboxylate (**9db**)

Methyl cinnamate (1.29 g, 7.98 mmol, 14.2 equiv) was added to a solution of flavonol **8db** (217 mg, 562 μmol, 1.00 equiv) in dry chloroform (11.0 mL) and freshly distilled 2,2,2-trifluoroethanol (4.68 mL). The reaction mixture was degassed for 30 min, then cooled to −5 °C and irradiated with UV light (λ_max_ = 365 nm) until it no longer fluoresced greenish (20 h). Subsequently, the solvent was removed under reduced pressure. The remaining amount of methyl cinnamate was then removed by column chromatography (petroleum ether/EtOAc 1:0 → 3:1). The desired cycloadduct was obtained as a mixture of isomers as a yellowish oil (235 mg). Without any further purification the product of the first step (235 mg, 429 μmol, 1.00 equiv) was dissolved in MeOH (15.9 mL). Then, NaOMe solution (232 μL, 25 wt% in MeOH, 1.39 mmol, 3.25 equiv) was added and the mixture was heated under refluxing conditions for 1 h. Subsequently, the reaction was terminated by the addition of NH_4_Cl solution (sat., aq.). The phases were separated and the aqueous phase was extracted with EtOAc (3×). The organic phases were combined, dried over MgSO_4_, filtered and the solvent was removed under reduced pressure. The desired keto ester was obtained as a mixture of isomers as a yellowish solid (155 mg) and used directly for the next step. A mixture of (CH_3_)_4_N(OAc)_3_BH (478 mg, 1.82 mmol, 6.42 equiv) and freshly distilled AcOH (168 μL, 2.94 mmol, 10.4 equiv) in MeCN (7.34 mL) was stirred for 5 min at rt. Then, a solution of the product of the second step (155 mg, 283 μmol, 1.00 equiv) in MeCN (4.87 mL) was added. The mixture was protected from light and stirred for 19 h at rt. The reaction was then terminated by adding NH_4_Cl solution (sat., aq.) and sodium potassium tartrate solution (aq., 2.00 M). The phases were separated and the aqueous layer was extracted with CH_2_Cl_2_ (3×). The combined organic layers were dried over MgSO_4_, filtered and concentrated under reduced pressure. Column chromatography (CH_2_Cl_2_/EtOAc 1:0 → 9:1) was then performed to obtain the racemic *endo*-product **9db** as a colorless foam (12.0 mg, 21.8 μmol, 4% over three steps). *R*_f_ = 0.38 (petroleum ether/EtOAc 7:3); ^**1**^**H NMR** (DMSO-*d*_6_, 400 MHz): δ [ppm] 7.20 (d, *J* = 8.7 Hz, 2H, *H*-3′, *H*-5′), 7.17 (d, *J* = 1.6 Hz, 1H, *H*-5), 7.09–6.97 (m, 8H, *H*-7, *H*-2′, *H*-6′, *H*-2″, *H*-3″, *H*-4″, *H*-5″, *H*-6″), 5.85 (s, 1H, *H*O-8b), 5.77 (d, *J* = 6.1 Hz, 1H, *H*O-1), 4.68 (t, *J* = 5.0 Hz, 1H, *H*-1), 4.43 (d, *J* = 13.9 Hz, 1H, *H*-3), 4.11 (dd, *J* = 13.9, 4.4 Hz, 1H, *H*-2), 3.59 (s, 3H, C*H*_3_O-11); ^**13**^**C NMR** (DMSO-*d*_6_, 100 MHz): δ [ppm] 170.1 (q, *C*-11), 160.4 (q, *C*-4a), 137.6 (q, *C*-1″), 135.6 (q, *C*-1′), 134.4 (q, *C*-6), 132.3 (q, *C*-8a), 129.6 (t, *C*-2′, *C*-6′), 129.3 (t, *C*-3′, *C*-5′), 127.8 (t, *C*-3″, *C*-5″), 127.6 (t, *C*-2″, *C*-6″), 126.0 (t, *C*-4″), 125.3 (q, *C*-8), 121.1 (t, *C*-7), 119.9 (q, *C*-4′), 109.3 (t, *C*-5), 102.1 (q, *C*-3a), 93.7 (q, *C*-8b), 78.1 (t, *C*-1), 54.9 (t, *C*-3), 51.7 (t, *C*-2), 51.6 (p, H_3_*C*O-11); **HRMS (ESI**^**+**^**)***m*/*z* calcd for C_25_H_19_BrCl_2_O_5_Na [M+Na]^+^ 570.9702 found 570.9691; **HPLC Purity** 97.03%.

### Synthesis of (±)-Methyl (1*R*,2*R*,3*S*,3a*R*,8b*S*)-6,8-dibromo-1,8b-dihydroxy-3a-(4-methoxyphenyl)-3-phenyl-2,3,3a,8b-tetrahydro-1*H*-cyclopenta[*b*]benzofuran-2-carboxylate (**9e**)

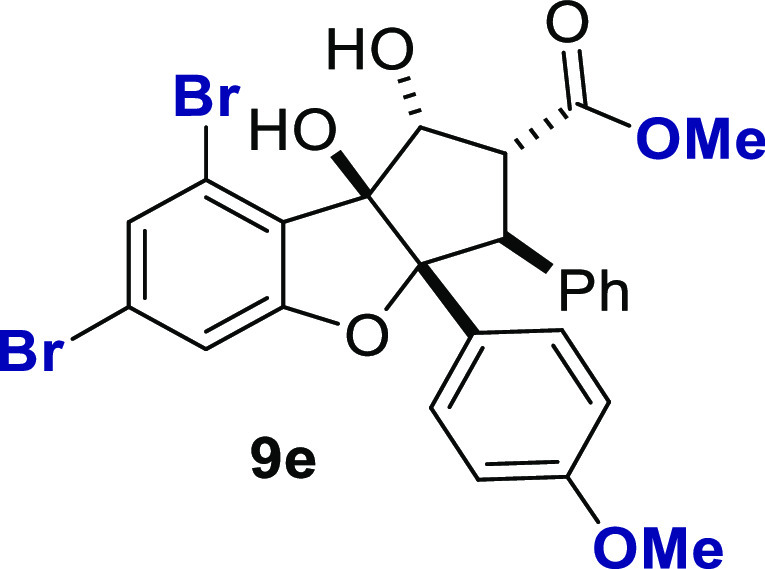



#### (*E*)-1-(2,4-Dibromo-6-hydroxyphenyl)-3-(4-methoxyphenyl)prop-2-en-1-one (**12e**)

Acetophenone **3e** (717 mg, 2.44 mmol, 1.00 equiv) was added to a solution of NaOEt (498 mg, 7.32 mmol, 3.00 equiv) in EtOH (8.41 mL). After stirring for 1 h at rt, 4-methoxybenzaldehyde (296 μL, 2.44 mmol, 1.00 equiv) was added and the reaction mixture was stirred overnight. The resulting yellow suspension was poured into H_2_O and acidified to pH = 1 with HCl (10 wt% in H_2_O). The yellow precipitate was filtered, washed with H_2_O and dried under reduced pressure. The desired compound **12e** was obtained as a yellow solid (923 mg, 2.24 mmol, 92%). *R*_f_ = 0.36 (petroleum ether/EtOAc 3:1); ^**1**^**H NMR** (CDCl_3_, 400 MHz): δ [ppm] 10.94 (s, 1H, O*H*), 7.78 (d, *J* = 15.5 Hz, 1H, C(O)CH=C*H*), 7.60 (d, *J* = 8.7 Hz, 2H, 2× Ar*H*), 7.46 (d, *J* = 15.5 Hz, 1H, C(O)C*H*), 7.38 (d, *J* = 1.7 Hz, 1H, Ar*H*), 7.17 (d, *J* = 1.8 Hz, 1H, Ar*H*), 6.95 (d, *J* = 8.7 Hz, 2H, 2× Ar*H*), 3.87 (s, 3H, OC*H*_3_); ^**13**^**C NMR** (CDCl_3_, 100 MHz): δ [ppm] 194.2 (q, *C*=O), 162.4 (q, Ar*C*), 161.8 (q, Ar*C*), 144.6 (t, C(O)CH=*C*H), 131.0 (t, 2× Ar*C*H), 128.3 (t, Ar*C*H), 127.8 (q, Ar*C*), 127.4 (q, Ar*C*), 123.5 (t, C(O)*C*H), 122.9 (q, Ar*C*), 122.5 (q, Ar*C*), 120.7 (t, Ar*C*H), 114.8 (t, 2× Ar*C*H), 55.6 (p, O*C*H_3_); **HRMS (ESI**^–^**)***m*/*z* calcd for C_16_H_11_O_3_Br [M–H]^−^ 408.9075, found 408.9068.

#### 5,7-Dibromo-3-hydroxy-2-(4-methoxyphenyl)-4*H*-chromen-4-one (**8e**)

To a suspension of chalcone **12e** (824 mg, 2.00 mmol, 1.00 equiv) in MeOH (17.2 mL), NaOH (3.00 M, aq., 2.58 mL, 7.74 mmol, 3.87 equiv) was added and cooled to 0 °C. H_2_O_2_ (30 wt% in H_2_O, 652 μL, 6.40 mmol, 3.20 equiv) was then added dropwise and the solution was stirred at 0 °C for 3 h. Subsequently, the cooling bath was removed and the mixture was stirred for another 20 h. Then, HCl (10 wt% in H_2_O) was added, leading to the formation of a yellow precipitate. Subsequently, the suspension was extracted with CH_2_Cl_2_ (4×). The combined organic layers were dried over MgSO_4_, filtered and concentrated under reduced pressure. The crude material was purified by recrystallization from EtOAc to give the desired product **8e** as a pale-yellowish solid (125 mg, 293 μmol, 15%). *R*_f_ = 0.39 (petroleum ether/EtOAc 4:1); ^**1**^**H NMR** (CDCl_3_, 400 MHz): δ [ppm] 8.18 (d, *J* = 9.0 Hz, 2H, 2× Ar*H*), 7.78 (d, *J* = 1.8 Hz, 1H, Ar*H*), 7.75 (d, *J* = 1.8 Hz, 1H, Ar*H*), 7.04 (d, *J* = 9.0 Hz, 2H, 2× Ar*H*); ^**13**^**C NMR** (CDCl_3_, 100 MHz): δ [ppm] 171.7 (q, *C*=O), 161.5 (q, Ar*C*), 156.3 (q, Ar*C*), 144.1 (q, *C*=COH), 138.0 (q, *C*OH), 133.7 (t, Ar*C*H), 129.5 (t, 2× Ar*C*H), 126.9 (q, Ar*C*), 122.7 (q, Ar*C*), 121.3 (t, Ar*C*H), 121.2 (q, Ar*C*), 117.6 (q, Ar*C*), 114.4 (q, 2× Ar*C*H), 55.6 (p, O*C*H_3_); **HRMS (EI)***m*/*z* calcd for C_16_H_10_Cl_2_O_4_ [M]^+^ 423.8946, found 423.8943. The analytical data are consistent with those reported in the literature.^45^

#### (±)-Methyl (1*R*,2*R*,3*S*,3a*R*,8b*S*)-6,8-dibromo-1,8b-dihydroxy-3a-(4-methoxyphenyl)-3-phenyl-2,3,3a,8b-tetrahydro-1*H*-cyclopenta[*b*]benzofuran-2-carboxylate (**9e**)

Methyl cinnamate (665 mg, 4.10 mmol, 14.2 equiv) was added to a solution of flavonol **8e** (123 mg, 289 μmol, 1.00 equiv) in dry chloroform (5.66 mL) and freshly distilled 2,2,2-trifluoroethanol (2.41 mL). The reaction mixture was degassed for 30 min, then cooled to −5 °C and irradiated with UV light (λ_max_ = 365 nm) until it no longer fluoresced greenish (20 h). Subsequently, the solvent was removed under reduced pressure. The remaining amount of methyl cinnamate was then removed by column chromatography (petroleum ether/EtOAc 9:1 → 1:1). The desired cycloadduct was obtained as a mixture of isomers as a yellowish solid (170 mg). Without any further purification the product of the first step (170 mg, 289 μmol, 1.00 equiv) was dissolved in MeOH (10.7 mL). Then, NaOMe solution (156 μL, 25 wt% in MeOH, 939 μmol, 3.25 equiv) was added and the mixture was heated under refluxing conditions for 1 h. Subsequently, the reaction was terminated by the addition of NH_4_Cl solution (sat., aq.). The phases were separated and the aqueous phase was extracted with EtOAc (3×). The organic phases were combined, dried over MgSO_4_, filtered and the solvent was removed under reduced pressure. The desired keto ester was obtained as a mixture of isomers as an orange solid (158 mg) and used directly for the next step. A mixture of (CH_3_)_4_N(OAc)_3_BH (454 mg, 1.72 mmol, 6.42 equiv) and freshly distilled AcOH (160 μL, 2.80 mmol, 10.4 equiv) in MeCN (6.98 mL) was stirred for 5 min at rt. Then, a solution of the product of the second step (158 mg, 269 μmol, 1.00 equiv) in MeCN (4.63 mL) was added. The mixture was protected from light and stirred for 19 h at rt. The reaction was then terminated by adding NH_4_Cl solution (sat., aq.) and sodium potassium tartrate solution (aq., 2.00 M). The phases were separated and the aqueous layer was extracted with CH_2_Cl_2_ (3×). The combined organic layers were dried over MgSO_4_, filtered and concentrated under reduced pressure. Column chromatography (CH_2_Cl_2_/EtOAc 1:0 → 9:1) was then performed to obtain the racemic *endo*-product **9e** as a pale-yellow foam (84.8 mg, 144 μmol, 50% over three steps). *R*_f_ = 0.26 (petroleum ether/EtOAc 7:3); ^**1**^**H NMR** (DMSO-*d*_6_, 400 MHz): δ [ppm] 7.30 (d, *J* = 1.5 Hz, 1H, *H*-5), 7.26 (d, *J* = 1.5 Hz, 1H, *H*-7), 7.07–7.03 (m, 2H, *H*-2″, *H*-6″), 6.99–6.96 (m, 5H, *H*-2′, *H*-6′, *H*-3″, *H*-4″, *H*-5″), 6.56 (dt, *J* = 9.9, 2.5 Hz, 2H, *H*-3′, *H*-5′), 5.65 (t, *J* = 3.0 Hz, 2H, *H*O-1, *H*O-8b), 4.68 (dd, *J* = 5.9, 4.4 Hz, 1H, *H*-1), 4.41 (d, *J* = 13.9 Hz, 1H, *H*-3), 4.05 (dd, *J* = 14.0, 4.3 Hz, 1H, *H*-2), 3.59 (s, 3H, *H*_3_CO-11), 3.56 (s, 3H, *H*_3_CO-4′); ^**13**^**C NMR** (DMSO-*d*_6_, 100 MHz): δ [ppm] 170.3 (q, *C*-11), 160.9 (q, *C*-4a), 157.6 (q, *C*-4′), 138.0 (q, *C*-1″), 128.5 (t, *C*-2′, *C*-6′), 128.1 (q, C-1′), 127.9 (t, *C*-3″, *C*-5″), 127.7 (q, *C*-8a), 127.5 (t, *C*-2″, *C*-6″), 126.3 (t, *C*-7), 125.8 (t, *C*-4″), 122.3 (q, *C*-6), 121.1 (q, *C*-8), 112.3 (t, *C*-5), 111.8 (t, *C*-3′, *C*-5′), 102.3 (q, *C*-3a), 94.0 (q, *C*-8b), 77.9 (t, *C*-1), 54.9 (t, *C*-3), 54.7 (p, H_3_*C*O-4′), 51.57 (t, *C*-2), 51.5 (p, H_3_*C*O-11); **HRMS (ESI**^**+**^**)***m*/*z* calcd for C_26_H_22_Br_2_O_6_Na [M+Na]^+^ 610.9681 found 610.9686; **HPLC purity** ∼100.00%.

### Synthesis of (±)-Methyl (1*R*,2*R*,3*S*,3a*R*,8b*S*)-6-bromo-8-chloro-1,8b-dihydroxy-3a-(4-methoxyphenyl)-3-phenyl-2,3,3a,8b-tetrahydro-1*H*-cyclopenta[*b*]benzofuran-2-carboxylate (**9f**)

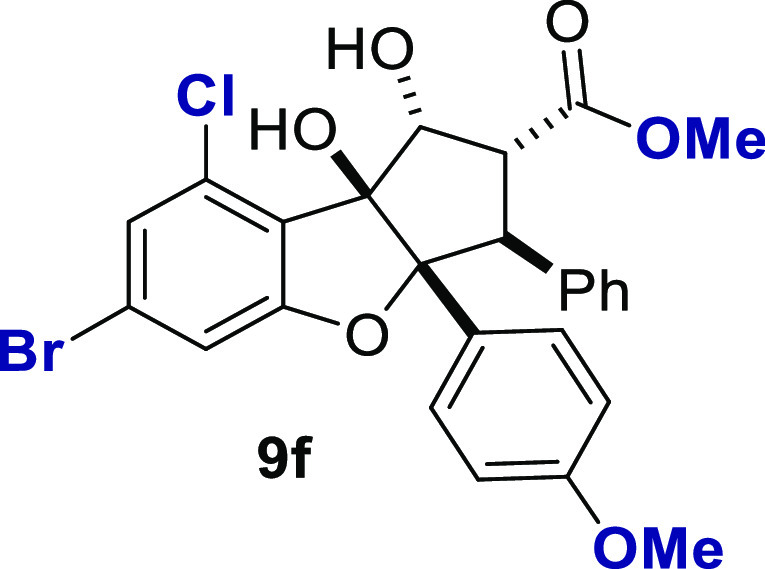



#### (*E*)-1-(4-Bromo-2-chloro-6-hydroxyphenyl)-3-(4-methoxyphenyl)prop-2-en-1-one (**12f**)

Acetophenone **3f** (1.19 g, 4.77 mmol, 1.00 equiv) was added to a solution of NaOEt (970 mg, 14.3 mmol, 3.00 equiv) in EtOH (16.0 mL). After stirring for 1 h at rt, 4-methoxybenzaldehyde (580 μL, 4.77 mmol, 1.00 equiv) was added and the reaction mixture was stirred overnight. The resulting yellow suspension was poured into H_2_O and acidified to pH = 1 with HCl (10 wt% in H_2_O). The yellow precipitate was filtered, washed with H_2_O and dried under reduced pressure. The desired compound **12f** was obtained as a yellow solid (1.62 g, 4.59 mmol, 96%). *R*_f_ = 0.33 (petroleum ether/EtOAc 3:1); ^**1**^**H NMR** (CDCl_3_, 400 MHz): δ [ppm] 11.49 (bs, 1H, O*H*), 7.81 (d, *J* = 15.5 Hz, 1H, C(O)CH=C*H*), 7.59 (d, *J* = 8.8 Hz, 2H, 2× Ar*H*), 7.49 (d, *J* = 15.5 Hz, 1H, C(O)C*H*), 7.16 (d, *J* = 1.8 Hz, 1H, Ar*H*), 7.13 (d, *J* = 1.8 Hz, 1H, Ar*H*), 6.94 (d, *J* = 8.8 Hz, 2H, 2× Ar*H*), 3.86 (s, 3H, OC*H*_3_); ^**13**^**C NMR** (CDCl_3_, 100 MHz): δ [ppm] 193.7 (q, *C*=O), 162.6 (q, Ar*C*), 162.4 (q, Ar*C*), 144.9 (t, C(O)CH=*C*H), 134.6 (q, Ar*C*), 131.0 (t, 2× Ar*C*H), 127.8 (q, Ar*C*), 127.4 (q, Ar*C*), 125.0 (t, Ar*C*H), 123.6 (t, C(O)*C*H), 120.7 (q, Ar*C*), 120.3 (t, Ar*C*H), 114.7 (t, 2× Ar*C*H), 55.6 (p, O*C*H_3_); **HRMS (ESI**^–^**)***m*/*z* calcd for C_16_H_11_O_3_ClBr [M–H]^−^ 364.9580, found 364.9582.

#### 7-Bromo-5-chloro-3-hydroxy-2-(4-methoxyphenyl)-4*H*-chromen-4-one (**8f**)

To a suspension of chalcone **12f** (1.62 g, 4.42 mmol, 1.00 equiv) in MeOH (53.3 mL), NaOH (3.00 M, aq., 7.58 mL, 22.7 mmol, 5.15 equiv) was added and cooled to 0 °C. H_2_O_2_ (35 wt% in H_2_O., 1.46 mL, 17.0 mmol, 3.84 equiv) was then added dropwise and the solution was stirred at 0 °C for 3 h. Subsequently, the cooling bath was removed and the mixture was stirred for another 18 h. Then, HCl (10 wt% in H_2_O) was added, leading to the formation of a yellow precipitate. Subsequently, the suspension was extracted with CH_2_Cl_2_ (4×). The combined organic layers were dried over MgSO_4_, filtered and concentrated under reduced pressure. The crude material was purified by recrystallization from EtOH to give the desired product **8f** as a yellow solid (203 mg, 531 μmol, 12%). *R*_f_ = 0.30 (petroleum ether/EtOAc 4:1); ^**1**^**H NMR** (CDCl_3_, 600 MHz): δ [ppm] 8.18 (dt, *J* = 9.9, 2.6 Hz, 2H, 2× Ar*H*), 7.71 (d, *J* = 1.8 Hz, 1H, Ar*H*), 7.54 (d, *J* = 1.8 Hz, 1H, Ar*H*), 7.17 (bs, 1H, *OH*), 7.04 (dt, *J* = 9.9, 2.6 Hz, 2H, 2× Ar*H*), 3.90 (3H, OC*H*_3_); ^**13**^**C NMR** (CDCl_3_, 150 MHz): δ [ppm] 171.7 (q, *C*=O), 161.5 (q, Ar*C*), 156.5 (q, Ar*C*), 144.1 (q, *C*=COH), 138.2 (q, *C*OH), 134.4 (q, Ar*C*), 130.2 (t, Ar*C*H), 129.6 (t, 2× Ar*C*H), 126.4 (q, Ar*C*), 122.7 (q, Ar*C*), 120.6 (t, Ar*C*H), 116.9 (q, Ar*C*), 114.4 (t, 2× Ar*C*H), 55.6 (p, O*C*H_3_); **HRMS (EI)***m*/*z* calcd for C_16_H_10_ClO_4_Br [M]^+^ 379.9451, found 379.9469.

#### (±)-Methyl (1*R*,2*R*,3*S*,3a*R*,8b*S*)-6-bromo-8-chloro-1,8b-dihydroxy-3a-(4-methoxyphenyl)-3-phenyl-2,3,3a,8b-tetrahydro-1*H*-cyclopenta[*b*]benzofuran-2-carboxylate (**9f**)

Methyl cinnamate (1.17 g, 7.20 mmol, 14.2 equiv) was added to a solution of flavonol **8f** (194 mg, 507 μmol, 1.00 equiv) in dry chloroform (10.4 mL) and freshly distilled 2,2,2-trifluoroethanol (4.14 mL). The reaction mixture was degassed for 30 min, then cooled to −5 °C and irradiated with UV light (λ_max_ = 365 nm) until it no longer fluoresced greenish (14 h). Subsequently, the solvent was removed under reduced pressure. The remaining amount of methyl cinnamate was then removed by column chromatography (petroleum ether/EtOAc 5:1 → 1:1). The desired cycloadduct was obtained as a mixture of isomers as a yellowish solid (262 mg). Without any further purification the product of the first step (262 mg, 482 μmol, 1.00 equiv) was dissolved in MeOH (19.3 mL). Then, NaOMe solution (377 μL, 25 wt% in MeOH, 1.59 mmol, 3.30 equiv) was added and the mixture was heated under refluxing conditions for 1 h. Subsequently, the reaction was terminated by the addition of NH_4_Cl solution (sat., aq.). The phases were separated and the aqueous phase was extracted with EtOAc (3×). The organic phases were combined, dried over MgSO_4_, filtered and the solvent was removed under reduced pressure. Product **E21** was obtained as a mixture of isomers as an orange solid (262 mg) and used directly for the next step. The desired keto ester was obtained as a mixture of isomers as an orange solid (262 mg) and used directly for the next step. A mixture of (CH_3_)_4_N(OAc)_3_BH (814 mg, 3.09 mmol, 6.42 equiv) and freshly distilled AcOH (288 μL, 5.02 mmol, 10.4 equiv) in MeCN (4.25 mL) was stirred for 5 min at rt. Then, a solution of the product of the second step (262 mg, 482 μmol, 1.00 equiv) in MeCN (2.83 mL) was added. The mixture was protected from light and stirred for 19 h at rt. The reaction was then terminated by adding NH_4_Cl solution (sat., aq.) and sodium potassium tartrate solution (aq., 2.00 M). The phases were separated and the aqueous layer was extracted with CH_2_Cl_2_ (3×). The combined organic layers were dried over MgSO_4_, filtered and concentrated under reduced pressure. Column chromatography (CH_2_Cl_2_/EtOAc 1:0 → 9:1) was then performed to obtain the racemic *endo*-product **9f** as a colorless foam (153 mg, 280 μmol, 55% over three steps). *R*_f_ = 0.32 (petroleum ether/EtOAc 7:3); ^**1**^**H NMR** (DMSO-*d*_6_, 400 MHz): δ [ppm] 7.27 (d, *J* = 1.5 Hz, 1H, *H*-5), 7.14 (d, *J* = 1.5 Hz, 1H, *H*-7), 7.07–6.95 (m, 7H, *H*-2′, *H*-6′, *H*-2″, *H*-3″, *H*-4″, *H*-5″, *H*-6″), 6.57 (d, *J* = 9.0 Hz, 2H, *H*-3′, *H*-5′), 5.72 (d, *J* = 6.2 Hz, 1H, *H*O-1), 5.69 (s, 1H, *H*O-8b), 4.69 (dd, *J* = 6.0, 4.6 Hz, 1H, *H*-1), 4.37 (d, *J* = 14.0 Hz, 1H, *H*-3), 4.05 (dd, *J* = 14.1, 4.4 Hz, 1H, *H*-2), 3.59 (s, 3H, *H*_3_CO-11), 3.58 (s, 3H, *H*_3_CO-4′); ^**13**^**C NMR** (DMSO-*d*_6_, 100 MHz): δ [ppm] 170.2 (q, *C*-11), 160.8 (q, *C*-4a), 157.6 (q, *C*-4′), 138.0 (q, *C*-1″), 132.8 (q, *C*-8a), 128.5 (t, *C*-2′, *C*-6′), 128.0 (q, C-1′), 127.9 (t, *C*-3″, *C*-5″), 127.5 (t, *C*-2″, *C*-6″), 126.1 (t, *C*-8), 125.8 (t, *C*-4″), 123.5 (q, *C*-7), 122.2 (q, *C*-6), 112.0 (t, *C*-5), 111.9 (t, *C*-3′, *C*-5′), 102.2 (q, *C*-3a), 93.6 (q, *C*-8b), 78.1 (t, *C*-1), 54.9 (t, *C*-3), 54.7 (p, H_3_*C*O-4′), 51.7 (t, *C*-2), 51.5 (p, H_3_*C*O-11); **HRMS (ESI**^**+**^**)***m*/*z* calcd for C_26_H_22_BrClO_6_Na [M+Na]^+^ 567.0186 found 567.0181; **HPLC purity** 99.72%.

### Synthesis of (±)-Methyl (1*R*,2*R*,3*S*,3a*R*,8b*S*)-8-bromo-6-chloro-1,8b-dihydroxy-3a-(4-methoxyphenyl)-3-phenyl-2,3,3a,8b-tetrahydro-1*H*-cyclopenta[*b*]benzofuran-2-carboxylate (**9g**)

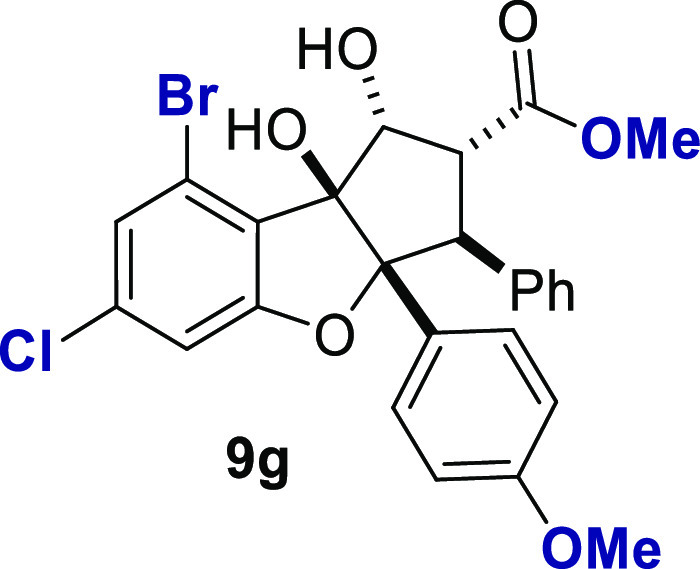



#### (*E*)-1-(2-Bromo-4-chloro-6-hydroxyphenyl)-3-(4-methoxyphenyl)prop-2-en-1-one (**12g**)

Acetophenone **3g** (900 mg, 3.61 mmol, 1.00 equiv) was added to a solution of NaOEt (736 mg, 10.8 mmol, 3.00 equiv) in EtOH (68.5 mL). After stirring for 1 h at rt, 4-methoxybenzaldehyde (439 μL, 3.61 mmol, 1.00 equiv) was added and the reaction mixture was stirred overnight. The resulting yellow suspension was poured into H_2_O and acidified to pH = 1 with HCl (10 wt% in H_2_O). The yellow precipitate was filtered, washed with H_2_O and dried under reduced pressure. The desired compound **12g** was obtained as a yellow solid (287 mg, 781 μmol, 22%). *R*_f_ = 0.62 (petroleum ether/EtOAc 3:2); ^**1**^**H NMR** (CDCl_3_, 600 MHz): δ [ppm] 11.04 (bs, 1H, O*H*), 7.78 (d, *J* = 15.5 Hz, 1H, C(O)CH=C*H*), 7.60 (d, *J* = 8.7 Hz, 2H, 2× Ar*H*), 7.47 (d, *J* = 15.5 Hz, 1H, C(O)C*H*), 7.23 (d, *J* = 2.0 Hz, 1H, Ar*H*), 7.00 (d, *J* = 2.0 Hz, 1H, Ar*H*), 6.95 (d, *J* = 8.8 Hz, 2H, 2× Ar*H*), 3.87 (s, 3H, OC*H*_3_); ^**13**^**C NMR** (CDCl_3_, 150 MHz): δ [ppm] 194.1 (q, *C*=O), 162.4 (q, Ar*C*), 162.0 (q, Ar*C*), 144.5 (t, C(O)CH=*C*H), 139.7 (q, Ar*C*), 131.0 (t, 2× Ar*C*H), 127.5 (q, Ar*C*), 125.6 (t, Ar*C*H), 123.6 (t, C(O)*C*H), 122.54 (q, Ar*C*), 122.53 (q, Ar*C*), 120.7 (t, Ar*C*H), 117.7 (t, Ar*C*H), 114.8 (t, 2× Ar*C*H), 55.6 (p, O*C*H_3_); **HRMS (ESI**^+^**)***m*/*z* calcd for C_16_H_12_O_3_NaClBr [M+Na]^+^ 388.9556, found 388.9551.

#### 5-Bromo-7-chloro-3-hydroxy-2-(4-methoxyphenyl)-4*H*-chromen-4-one (**8g**)

To a suspension of chalcone **12g** (287 mg, 781 μmol, 1.00 equiv) in MeOH (9.25 mL), NaOH (3.00 M, aq., 1.34 mL, 4.02 mmol, 5.15 equiv) was added and cooled to 0 °C. H_2_O_2_ (35 wt% in H_2_O, 257 μL, 3.00 mmol, 3.84 equiv) was then added dropwise and the solution was stirred at 0 °C for 3 h. Subsequently, the cooling bath was removed and the mixture was stirred for another 16 h. Then, HCl (10 wt% in H_2_O) was added, leading to the formation of a yellow precipitate. The suspension was extracted with CH_2_Cl_2_ (4×). The combined organic layers were dried over MgSO_4_, filtered and concentrated under reduced pressure. The crude material was purified by recrystallization from EtOH to give the desired product **8g** as a yellow solid (65.0 mg, 170 μmol, 22%). *R*_f_ = 0.31 (petroleum ether/EtOAc 4:1); ^**1**^**H NMR** (CDCl_3_, 600 MHz): δ [ppm] 8.18 (dt, *J* = 9.9, 2.6 Hz, 2H, 2× Ar*H*), 7.64 (d, *J* = 2.0 Hz, 1H, Ar*H*), 7.59 (d, *J* = 2.0 Hz, 1H, Ar*H*), 7.16 (s, 1H, O*H*), 7.05 (dt, *J* = 9.9, 2.6 Hz, 2H, 2× Ar*H*), 3.90 (s, 3H, OC*H*_3_); ^**13**^**C NMR** (CDCl_3_, 150 MHz): δ [ppm] 171.7 (q, *C*=O), 161.5 (q, Ar*C*), 156.4 (q, Ar*C*), 144.2 (q, *C*=COH), 138.9 (q, Ar*C*), 137.9 (q, *C*OH), 131.2 (t, Ar*C*H), 129.5 (t, 2× Ar*C*H), 122.8 (q, Ar*C*), 121.2 (q, Ar*C*), 118.2 (t, Ar*C*H), 117.3 (q, Ar*C*), 114.4 (t, 2× Ar*C*H), 55.6 (p, O*C*H_3_); **HRMS (EI)***m*/*z* calcd for C_16_H_10_ClO_4_Br [M]^+^ 379.9451, found 379.9453.

#### (±)-Methyl (1*R*,2*R*,3*S*,3a*R*,8b*S*)-8-bromo-6-chloro-1,8b-dihydroxy-3a-(4-methoxyphenyl)-3-phenyl-2,3,3a,8b-tetrahydro-1*H*-cyclopenta[*b*]benzofuran-2-carboxylate (**9g**)

Methyl cinnamate (392 mg, 2.42 mmol, 14.2 equiv) was added to a solution of flavonol **8g** (65.0 mg, 170 μmol, 1.00 equiv) in dry chloroform (3.48 mL) and freshly distilled 2,2,2-trifluoroethanol (1.39 mL). The reaction mixture was degassed for 30 min, then cooled to −5 °C and irradiated with UV light (λ_max_ = 365 nm) until it no longer fluoresced greenish (22 h). Subsequently, the solvent was removed under reduced pressure and the remaining amount of methyl cinnamate was removed by column chromatography (petroleum ether/EtOAc 5:1 → 1:1). The crude cycloadduct was obtained as a mixture of isomers as a yellowish solid (110 mg). Without any further purification the product of the first step (110 mg) was dissolved in MeOH (6.81 mL). Then, NaOMe solution (133 μL, 25 wt% in MeOH, 562 μmol, 3.30 equiv) was added and the mixture was heated under refluxing conditions for 1 h. Subsequently, the reaction was terminated by the addition of NH_4_Cl solution (sat., aq.). The phases were separated and the aqueous phase was extracted with EtOAc (3×). The organic phases were combined, dried over MgSO_4_, filtered and the solvent was removed under reduced pressure. The product was obtained as a mixture of isomers as an orange solid (110 mg) and used directly for the next step. The crude keto ester was obtained as a mixture of isomers as an orange solid (110 mg) and used directly for the next step. A mixture of (CH_3_)_4_N(OAc)_3_BH (288 mg, 1.09 mmol, 6.42 equiv) and freshly distilled AcOH (102 μL, 1.77 mmol, 10.4 equiv) in MeCN (1.50 mL) was stirred for 5 min at rt. Then, a solution of the product of the second step (110 mg) in MeCN (1.00 mL) was added. The mixture was protected from light and stirred for 19 h at rt. The reaction was then terminated by adding NH_4_Cl solution (sat., aq.) and sodium potassium tartrate solution (aq., 2.00 M). The phases were separated and the aqueous layer was extracted with CH_2_Cl_2_ (3×). The combined organic layers were dried over MgSO_4_, filtered and concentrated under reduced pressure. Column chromatography (CH_2_Cl_2_/EtOAc 1:0 → 9:1) was then performed to obtain the racemic *endo*-product **9g** as a pale-yellow foam (38.0 mg, 69.6 μmol, 41% over three steps). *R*_f_ = 0.55 (CH_2_Cl_2_/EtOAc 9:1); ^**1**^**H NMR** (DMSO-*d*_6_, 400 MHz): δ [ppm] 7.18 (d, *J* = 1.7 Hz, 1H, *H*-5), 7.15 (d, *J* = 1.7 Hz, 1H, *H*-7), 7.07–7.03 (m, 2H, *H*-2″, *H*-6″), 7.00–6.95 (m, 5H, *H*-2′, *H*-6′, *H*-3″, *H*-4″, *H*-5″), 6.56 (dt, *J* = 10.1, 2.6 Hz, 2H, *H*-3′, *H*-5′), 5.65 (t, *J* = 3.0 Hz, 2H, *H*O-1, *H*O-8b), 4.68 (dd, *J* = 5.9, 4.4 Hz, 1H, *H*-1), 4.41 (d, *J* = 14.0 Hz, 1H, *H*-3), 4.05 (dd, *J* = 13.9, 4.3 Hz, 1H, *H*-2), 3.59 (s, 3H, *H*_3_CO-11), 3.57 (s, 3H, *H*_3_CO-4′); ^**13**^**C NMR** (DMSO-*d*_6_, 100 MHz): δ [ppm] 170.3 (q, *C*-11), 160.8 (q, *C*-4a), 157.6 (q, *C*-4′), 138.0 (q, *C*-1″), 134.4 (q, *C*-6), 128.5 (t, *C*-2′, *C*-6′), 128.1 (q, C-1′), 127.9 (t, *C*-3″, *C*-5″), 127.5 (t, *C*-2″, *C*-6″), 127.2 (q, *C*-8a), 125.8 (t, *C*-4″), 123.7 (t, *C*-7), 120.8 (q, *C*-8), 111.9 (t, *C*-3′, *C*-5′), 109.5 (t, *C*-5), 102.4 (q, *C*-3a), 93.9 (q, *C*-8b), 78.0 (t, *C*-1), 54.9 (t, *C*-3), 54.7 (p, H_3_*C*O-4′), 51.7 (t, *C*-2), 51.5 (p, H_3_*C*O-11); **HRMS (ESI**^**+**^**)***m*/*z* calcd for C_26_H_22_BrClO_6_Na [M+Na]^+^ 567.0186 found 567.0172; **HPLC purity** 99.77%.

### Synthesis of (±)-Methyl (1*R*,2*R*,3*S*,3a*R*,8b*S*)-8-fluoro-1,8b-dihydroxy-6-methoxy-3a-(4-methoxyphenyl)-3-phenyl-2,3,3a,8b-tetrahydro-1*H*-cyclopenta[*b*]benzofuran-2-carboxylate (**9h**)

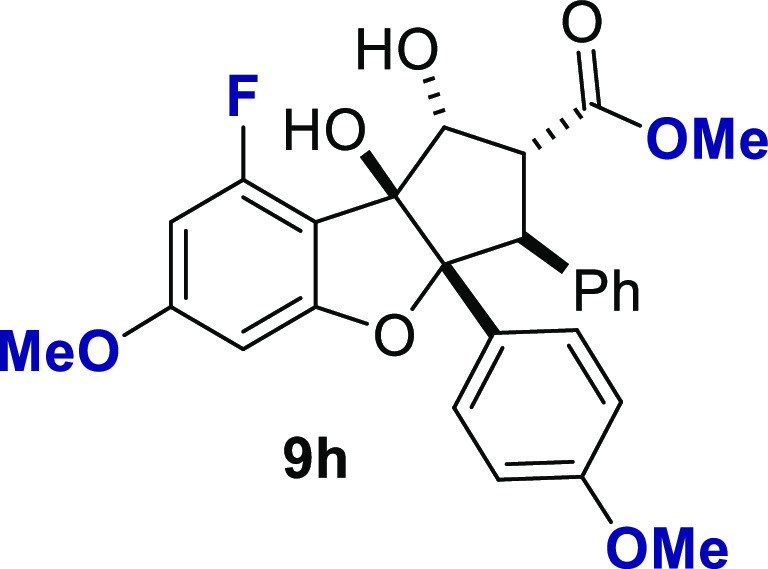



#### (*E*)-1-(2-Fluoro-6-hydroxy-4-methoxyphenyl)-3-(4-methoxyphenyl)prop-2-en-1-one (**12h**)

A suspension of NaOEt (221 mg, 3.26 mmol, 3.00 equiv) in dry EtOH (3.6 mL) was cooled down to rt, followed by the addition of 1-(2,4-difluoro-6-hydroxyphenyl)ethan-1-one (200 mg, 1.09 mmol, 1.00 equiv) at the same temperature. The suspension was stirred for 1 h, before *p*-anisaldehyde (132 μL, 1.09 mmol, 1.00 equiv) was added. The orange solution was stirred for 16 h at rt. The resulting orange suspension was poured into cold water and acidified to pH = 1 with HCl solution (aq., 1 M). The precipitate was filtered, washed with water and dried *in vacuo*. The crude was purified over silica gel chromatography (petroleum ether/EtOAc 10:1) to afford chalcone **12h** as a yellow-orange solid (221 mg, 0.73 mmol, 67%). *R*_f_ = 0.31 (petroleum ether/EtOAc 4:1); ^**1**^**H NMR** (CDCl_3_, 400 MHz): δ [ppm] 13.97 (s, 1H, O*H*), 7.89 (dd, *J* = 15, 3.7 Hz, 1H, C(O)CH=C*H*), 7.60 (dt, *J* = 9.5, 2.5 Hz, 2H, 2× Ar*H*), 7.52 (td, *J* = 15, 1.5 Hz, 1H, C(O)C*H*=CH), 6.28 (dd, *J* = 2.5, 1.1 Hz, 1H, Ar*H*), 6.20 (dd, *J* = 14, 2.5 Hz, 1H, Ar*H*), 3.86 (s, 3H, OC*H*_3_), 3.84 (s, 3H, OC*H*_3_); ^**13**^**C NMR** (CDCl_3_, 100 MHz): δ [ppm] 190.8 (q, *d*, *J* = 14 Hz, *C*=O), 167.2 (q, *d*, *J* = 7.7 Hz, Ar*C*), 165.7 (q, *d*, *J* = 17 Hz, Ar*C*), 164.2 (q, *d*, *J* = 253 Hz, Ar*C*), 161.9 (q, Ar*C*), 144.9 (t, *d*, *J* = 1.7 Hz, C(O)CH=*C*H), 130.6 (t, 2× Ar*C*), 127.6 (q, Ar*C*), 122.8 (t, *d*, *J* = 17 Hz, Ar*C*), 114.5 (t, 2× Ar*C*), 104.9 (q, *d*, *J* = 14 Hz, Ar*C*), 97.6 (t, *d*, *J* = 2.7 Hz, Ar*C*), 95.4 (t, *d*, *J* = 29 Hz, Ar*C*), 55.9 (p, *C*H_3_), 55.4 (p, *C*H_3_O); **HRMS** (ESI^+^) *m*/*z* calcd for C_17_H_16_O_4_F [M+H]^+^ 303.1033, found 303.1034.

#### 5-Fluoro-3-hydroxy-7-methoxy-2-(4-methoxyphenyl)-4*H*-chromen-4-one (**8h**)

Chalcone **12i** (41 mg, 0.13 mmol, 1.00 equiv) was suspended in MeOH (1.6 mL) and NaOH (aq., 3 M, 0.67 mmol, 5.00 equiv). The mixture was sonicated for 5 min until everything was dissolved, then cooled down to 0 °C. H_2_O_2_ (aq., 30%, 34 μL, 0.30 mmol, 2.25 equiv) was then added to the cooled down mixture. The resulting yellow suspension was stirred at rt for 16 h. The reaction was terminated by the addition of HCl solution (aq., 1 M). The solution was extracted with CH_2_Cl_2_. The organic layers were washed with brine, dried over MgSO_4_, filtered and concentrated *in vacuo*. The crude was precipitated in EtOH to give **8h** as a yellow solid (14 mg, 0.04 mmol, 32%). *R*_f_ = 0.25 (petroleum ether/EtOAc 2:1); ^**1**^**H NMR** (CDCl_3_, 400 MHz): δ [ppm] 8.18 (d, *J* = 8.9 Hz, 2H, 2× Ar*H*), 7.04 (d, *J* = 8.9 Hz, 2H, 2× Ar*H*), 7.04 (s, 1H, Ar*H*), 6.66 (dd, *J* = 12, 2.3 Hz, 1H, Ar*H*), 3.92 (s, 3H, OC*H*_3_), 3.89 (s, 3H, OC*H*_3_); ^**13**^**C NMR** (CDCl_3_, 100 MHz): δ [ppm]; 170.9 (q, *d*, *J* = 1.7 Hz, *C*=O), 163.8 (q, *d*, *J* = 14 Hz, Ar*C*), 161.3 (q, *d*, *J* = 262 Hz, Ar*C*), 160.9 (q, Ar*C*), 157.5 (q, *d*, *J* = 6.9 Hz, Ar*C*), 143.9 (q, Ar*C*), 137.4 (C = *C*OH), 129.2 (t, 2× Ar*C*), 123.2 (*C*=COH), 114.1 (2× Ar*C*), 105.8 (q, *d*, *J* = 13 Hz, Ar*C*), 100.9 (t, *d*, *J* = 23 Hz, Ar*C*), 96.7 (t, *d*, *J* = 3.7 Hz, Ar*C*), 56.1 (p, *C*H_3_O), 55.4 (p, *C*H_3_O); **HRMS (ESI**^**+**^**)***m*/*z* calcd for C_15_H_13_O_5_FNa [M+Na]^+^ 339.0645, found 339.0650.

#### (±)-Methyl (1*R*,2*R*,3*S*,3a*R*,8b*S*)-8-fluoro-1,8b-dihydroxy-6-methoxy-3a-(4-methoxyphenyl)-3-phenyl-2,3,3a,8b-tetrahydro-1*H*-cyclopenta[*b*]benzofuran-2-carboxylate (**9h**)

Methyl cinnamate (635 mg, 3.91 mmol, 14.20 equiv) was added to flavonol **8h** (87.2 mg, 0.28 mmol) in dry CHCl_3_ (5.5 mL) and freshly distilled 2,2,2-trifluoroethanol (2.3 mL). The solution was degassed with argon for 20 min and irradiated (100 W, 365 nm) at −10 °C under argon atmosphere for 16–40 h. After the starting material was fully consumed, the reaction mixture was concentrated *in vacuo* and purified by silica gel column chromatography (petroleum ether/EtOAc 4:1, then 1:1) to give cycloadduct mixture as a pale-yellow foam. To cycloadduct mixture (131 mg) in dry MeOH (9.1 mL) was added NaOMe (25 wt% in MeOH, 168 μL, 0.78 mmol, 2.84 equiv). The orange solution was stirred under refluxing conditions for 1 h. The reaction was terminated by the addition of NH_4_Cl (sat., aq.) and extracted with EtOAc. The organic layers were washed with water and NaCl (sat., aq.), dried over MgSO_4_, filtered and concentrated *in vacuo* to give the ketone crude as a yellow foam. A solution of Me_4_NBH(OAc)_3_ (423 mg, 1.61 mmol, 6.42 equiv) and freshly distilled CH_3_COOH (158 μL, 2.60 mmol, 10.41 equiv) were stirred in dry MeCN (6.4 mL) at rt for 5 min. A solution of ketone crude (120 mg) crude in dry MeCN (4.2 mL) was added to the suspension and stirred for 16 h at rt under light protection. The reaction was terminated by the addition of NH_4_Cl and NaK-tartrate (sat., aq.) and extracted with CH_2_Cl_2_ (3 × 15 mL). The organic layers were washed with water and NaCl (sat., aq.), dried over MgSO_4_ and concentrated *in vacuo*. The crude extract was purified by silica column chromatography (petroleum ether/EtOAc 5:1, then 3:1) to give **9c** as a pale-yellow foam (45 mg, 0.09 mmol, 37% over three steps). *R*_f_ = 0.53 (petroleum ether/EtOAc 3:2); ^**1**^**H NMR** (DMSO-*d*_6_, 400 MHz): δ [ppm] 7.10–7.04 (m, 3H, *H*-2″, *H*-4″,*H*-6″), 7.09–7.05 (m, 2H, *H*-2′, *H*-6′), 7.00–6.98 (m, 2H, *H*-3″, *H*-5″), 6.67–6.63 (dt, *J* = 9.9, 2.6 Hz, 2H, H-3′, H-5′), 6.42 (dd, *J* = 11, 2.0 Hz, 1H, H-5), 6.28 (dd, *J* = 11, 2.9 Hz, 1H, *H*-7), 4.90 (d, *J* = 5.4 Hz, 1H, *H*-1), 4.47 (d, *J* = 14 Hz, 1H, *H*-3), 3.98 (dd, *J* = 14, 5.5 Hz, 1H, H-2), 3.82 (s, 3H, C*H*_3_O-6′), 3.69 (s, 3H, C*H*_3_O-11), 3.68 (s, 3H, C*H*_3_O-4′); ^**13**^**C NMR** (DMSO-*d*_6_, 100 MHz): δ [ppm] 171.5 (q, *C*-11), 163.8 (q, *d*, *J* = 13 Hz, *C*-6), 161.6 (q, *d*, *J* = 12 Hz, *C*-4a), 160.5 (q, d, *J* = 249 Hz, *C*-8), 158.9 (q, *C*-4′), 136.6 (q, *C*-1″), 128.7 (t, *C*-1″, *C-*2″), 127.9 (t, *C*-2′, *C*-6′), 127.8 (t, *C*-3″, *C*-5″), 126.6 (t, *C*-4″), 126.1 (q, *C*-1′), 112.9 (t, *C*-3′, *C*-5′), 106.5 (q, *d*, *J* = 20 Hz, *C*-8a), 102.3 (q, *C*-3a), 95.7 (t, *d*, *J* = 24 Hz, *C*-7), 93.5 (t, *d*, *J* = 2.2 Hz, *C*-8b), 92.7 (t, *d*, *J* = 3.8 Hz, *C*-5), 78.6 (t, *C*-1), 55.9 (p, *C*H_3_O-6), 55.8 (t, *C*-3), 55.1 (p, *C*H_3_O-4′), 52.3 (p, *C*H_3_O-11), 50.7 (t, *C*-2); **HRMS (ESI**^**+**^**)***m*/*z* calcd for C_27_H_25_O_7_NaF [M+Na]^+^ 503.1482; found 503.1461; **HPLC purity** 96.04%.

### Synthesis of (±)-Methyl (1*R*,2*R*,3*S*,3a*R*,8b*S*)-6-fluoro-1,8b-dihydroxy-8-methoxy-3a-(4-methoxyphenyl)-3-phenyl-2,3,3a,8b-tetrahydro-1*H*-cyclopenta[*b*]benzofuran-2-carboxylate (9i)

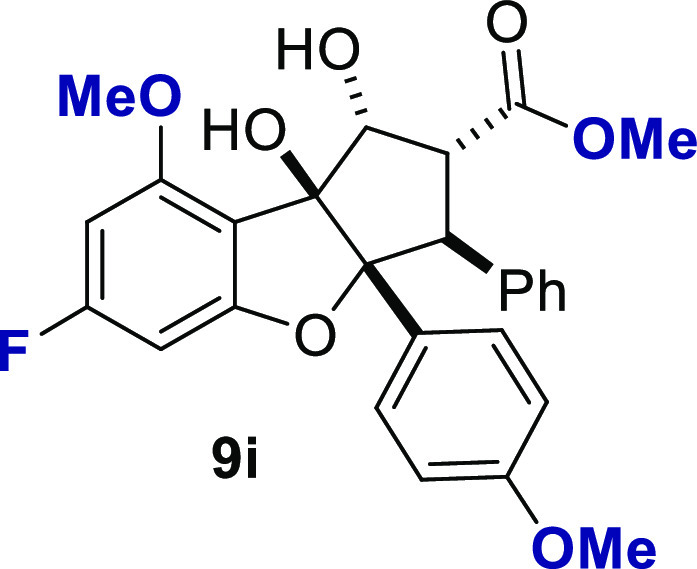



#### (*E*)-1-(4-Fluoro-2-hydroxy-6-methoxyphenyl)-3-(4-methoxyphenyl)prop-2-en-1-one (**12i**)

A suspension of NaOEt (221 mg, 3.26 mmol, 3.00 equiv) in dry EtOH (3.6 mL) was cooled down to rt, followed by the addition of 1-(4-fluoro-2-hydroxy-6-methoxyphenyl)ethan-1-one (200 mg, 1.09 mmol, 1.00 equiv) at the same temperature. The suspension was stirred for 1 h, before *p*-anisaldehyde (132 μL, 1.09 mmol, 1.00 equiv) was added. The orange solution was stirred for 16 h at rt. The resulting orange suspension was poured into cold water and acidified to pH = 1 with HCl (aq., 1 M). The precipitate was filtered, washed with water, dissolved in EtOAc, dried over MgSO_4_, filtered and concentrated *in vacuo*. The crude was purified over silica gel chromatography (petroleum ether/EtOAc 10:1) to afford **12i** as a yellow-orange solid (149 mg, 0.49 mmol, 45%). *R*_f_ = 0.29 (petroleum ether/EtOAc 3:1); ^**1**^**H NMR** (CDCl_3_, 400 MHz): δ [ppm] 7.83 (d, *J* = 16 Hz, 1H, C(O)CH=C*H*), 7.73 (d, *J* = 15 Hz, 1H, C(O)C*H*=CH), 7.57 (dt, *J* = 9.6, 2.4 Hz, 2H, 2× *ArH*), 6.94 (dt, *J* = 9.7, 2.5 Hz, 2H, 2× Ar*H*), 6.31 (dd, *J* = 10, 2.5 Hz, 1H, Ar*H*), 6.16 (dd, *J* = 11, 2.5 Hz, 1H, Ar*H*), 3.95 (s, 3H, OC*H*_3_), 3.86 (s, 3H, OC*H*_3_); ^**13**^**C NMR** (CDCl_3_, 100 MHz): δ [ppm] 193.3 (q, *C*=O), 167.5 (q, *d*, *J* = 253 Hz, Ar*C*), 167.5 (q, *d*, *J* = 17 Hz, Ar*C*), 162.9 (q, *d*, *J* = 14 Hz, Ar*C*), 161.7 (q, Ar*C*), 143.6 (t, C(O)CH=*C*H), 130.3 (t, 2× Ar*C*), 127.9 (q, Ar*C*), 122.8 (t, C(O)*C*H=CH), 114.3 (t, 2× Ar*C*), 108.8 (q, Ar*C*), 97.8 (t, *d*, *J* = 24 Hz, Ar*C*), 90.9 (t, *d*, *J* = 27 Hz, Ar*C*), 56.2 (*C*H_3_O), 55.8 (*C*H_3_O); **HRMS (ESI**^**+**^**)***m*/*z* calcd for C_17_H_15_O_4_FNa [M+Na]^+^ 325.0852; found: 325.0868.

#### 7-Fluoro-3-hydroxy-5-methoxy-2-(4-methoxyphenyl)-4*H*-chromen-4-one (**8i**)

Chalcone **12i** (36 mg, 0.12 mmol, 1.00 equiv) was suspended in MeOH (1.4 mL) and NaOH (aq., 3 M, 0.59 mmol, 5.00 equiv). The mixture was sonicated for 5 min until dissolved, then cooled down to 0 °C. H_2_O_2_ (aq., 30%, 30 μL, 0.26 mmol, 2.25 equiv) was then added to the cool mixture. The resulting yellow suspension was stirred at rt for 16 h. The reaction was terminated by the addition of HCl (aq., 1 M). The solution was extracted with CH_2_Cl_2_. The organic layers were washed with NaCl (sat., aq.), dried over MgSO_4_, filtered and concentrated *in vacuo*. The crude was reprecipitated in EtOH to give **8i** as a yellow solid (11.4 mg, 0.04 mmol, 30%). *R*_f_ = 0.78 (petroleum ether/EtOAc 1:1); ^**1**^**H NMR** (CDCl_3_, 400 MHz): δ [ppm] 8.17 (d, *J* = 9.0 Hz, 2H, 2× *ArH*), 7.04 (d, *J* = 9.0 Hz, 2H, 2× Ar*H*), 6.84 (dd, *J* = 9.2, 2.2 Hz, 1H, Ar*H*), 6.55 (dd, *J* = 11, 2.2 Hz, 1H, Ar*H*), 4.02 (s, 3H, OC*H*_3_), 3.89 (s, 3H, OC*H*_3_); ^**13**^**C NMR** (CDCl_3_, 100 MHz): δ [ppm] 171.2 (q, *C*=O), 165.9 (q, *d*, *J* = 252 Hz, Ar*C*), 161.4 (q, *d*, *J* = 13 Hz, Ar*C*), 160.9 (q, Ar*C*), 158.0 (q, *d*, *J* = 17 Hz, Ar*C*), 143.2 (q, *d*, *J* = 2.0 Hz, Ar*C*), 137.7 (q, *C*OH), 129.1 (t, 2× Ar*C*), 123.1 (q, *C*=COH), 114.1 (t, 2× Ar*C*), 108.6 (q, *d*, *J* = 2.3 Hz, Ar*C*), 96.4 (t, *d*, *J* = 25 Hz, Ar*C*), 95.1 (t, *d*, *J* = 27 Hz, Ar*C*), 56.8 (*C*H_3_O), 55.4 (*C*H_3_O); **HRMS (ESI**^**+**^**)***m*/*z* calcd for C_17_H_14_FO_5_ [M+H]^+^ 317.0825, found 317.0814.

#### (±)-Methyl (1*R*,2*R*,3*S*,3a*R*,8b*S*)-6-fluoro-1,8b-dihydroxy-8-methoxy-3a-(4-methoxyphenyl)-3-phenyl-2,3,3a,8b-tetrahydro-1*H*-cyclopenta[*b*]benzofuran-2-carboxylate (**9i**)

To a solution of **8i** (47 mg, 0.15 mmol, 1.00 equiv) in dry 2,2,2-TFE (1.2 mL) and dry CHCl_3_ (3 mL) was added methyl cinnamate (342 mg, 2.11 mmol, 14.2 equiv). The clear solution was degassed with argon for 15 min, followed by UV-irradiation (100 W, 365 nm) at −5 °C for 10–16 h. After the starting material was fully consumed, the solvent was removed *in vacuo* and the excess of methyl cinnamate was removed by silica gel purification (petroleum ether/EtOAc 4:1, then EtOAc). The cycloadduct mixture was used directly for the next step. To a solution of the cycloadduct mixture (39.7 mg) in MeOH (3 mL) was added NaOMe solution (25 wt% in MeOH, 51 μL, 0.24 mmol, 2.84 equiv) and refluxed for 1 h. The reaction was terminated by the addition of NH_4_Cl (sat., aq.). The aqueous layers were extracted with EtOAc. The collected organic layers were washed with NaCl (sat., aq.), dried over MgSO_4_, filtered and concentrated *in vacuo*. The foamy ketone crude was directly used for the next step. A solution of Me_4_NBH(OAc)_3_ (140 mg, 0.53 mmol, 6.42 equiv) and freshly distilled AcOH (50 μL, 0.86 mmol, 10.4 equiv) in dry MeCN (2 mL) was prepared and stirred at rt for 10 min. To this solution was added ketone crude (40.0 mg) in dry MeCN (1.4 mL). The reaction was carried out under light exclusion and stirred for 19 h at rt. The reaction was terminated by the addition of NaK-tartrate (sat., aq.) and NH_4_Cl (sat., aq.). The layers were separated and the aqueous layers were extracted with CH_2_Cl_2_. The collected organic layers were washed with water and NaCl (sat., aq.), dried over MgSO_4_, filtered and concentrated *in vacuo*. The crude was purified by silica gel column chromatography (petroleum ether/EtOAc 3:2) to yield **9i** (20 mg, 0.04 mmol, 50%) as a pale-yellow foam. *R*_f_ = 0.38 (petroleum ether/EtOAc 1:1); ^**1**^**H NMR** (DMSO-*d*_6_, 400 MHz): δ [ppm] 7.05–6.97 (m, 3H, *H*-3″, *H*-4″, *H*-5″), 7.01–6.98 (m, 2H, *H*-2′, *H*-6′), 6.91–6.88 (m, 2H, *H*-2″, *H*-6″), 6.57 (dt, *J* = 9.9, 2.6 Hz, 2H, *H*-3′, *H*-5′), 6.50 (dd, *J* = 9.5, 2.1 Hz, 1H, *H*-7), 6.41 (dd, *J* = 12, 2.1 Hz, 1H, *H*-5), 5.24 (s, O*H*), 4.66 (t, *J* = 5.3 Hz, 1H, *H*-1), 4.19 (d, *J* = 14 Hz, 1H, *H*-3), 3.95 (dd, *J* = 14, 5.2 Hz, 1H, *H*-2), 3.74 (s, 3H, *OCH*_3_-8), 3.59 (s, 3H, OC*H*_3_-4′), 3.55 (s, 3H, OC*H*_3_-11); ^**13**^**C NMR** (DMSO-*d*_6_, 100 MHz): δ [ppm] 170.3 (q, *C*-11), 164.6 (q, *d*, *J* = 241 Hz, *C*-6), 160.1 (q, *d*, *J* = 17 Hz, *C*-4a), 158.2 (q, d, *J* = 145 Hz, *C*-8), 157.6 (q, *C*-4′), 138.2 (q, *C*-1″), 128.6 (t, *C*-2, *C*-6′), 127.7 (t, *C*-2″, *C*-6″), 127.5 (t, *C*-3″, *C*-5″), 125.6 (t, *C*-4″), 111.8 (t, *C*-3′, *C*-5′), 111.7 (q, *d*, *J* = 2.5 Hz, *C*-8a), 101.9 (q, *C*-3a), 93.0 (q, *C*-8b), 92.2 (t, d, *J* = 27 Hz, *C*-5), 90.2 (t, *d*, *J* = 27 Hz, *C*-7), 78.7 (t, *C*-1), 55.8 (p, *C*H_3_O-8) 54.8 (*C*H_3_O-4′), 54.7 (t, *C*-3), 51.4 (t, *C*-2), 51.1 (*C*H_3_O-11); **HRMS (ESI**^**+**^**)***m*/*z* calc for C_27_H_25_FO_7_Na [M+Na]^+^ 503.1477, found: 503.1482; **HPLC purity** 96.60%.

### Synthesis of (±)-Methyl (1*R*,2*R*,3*S*,3a*R*,8b*S*)-8-chloro-1,8b-dihydroxy-6-methoxy-3a-(4-methoxyphenyl)-3-phenyl-2,3,3a,8b-tetrahydro-1*H*-cyclopenta[*b*]benzofuran-2-carboxylate (**9j**)

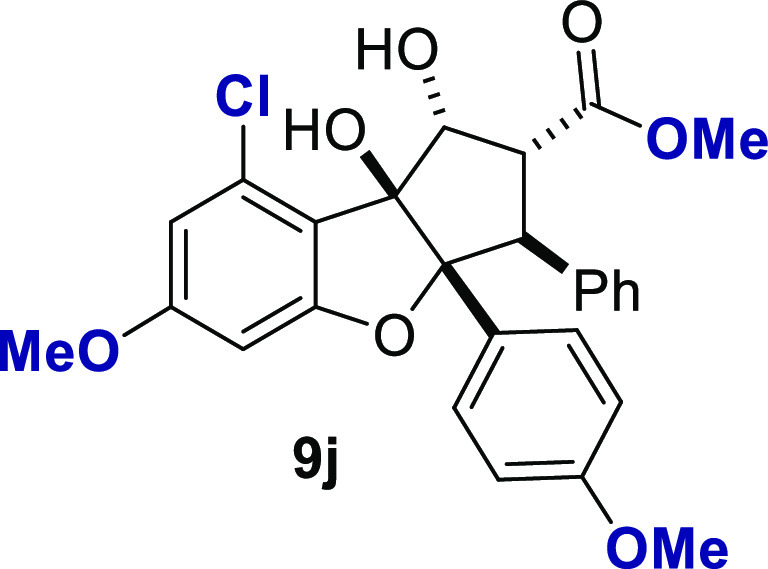



#### (*E*)-1-(2-Chloro-6-hydroxy-4-methoxyphenyl)-3-(4-methoxyphenyl)prop-2-en-1-one (**12j**)

Acetophenone **3j** (979 mg, 4.88 mmol, 1.00 equiv) was added to a solution of NaOEt (996 mg, 14.6 mmol, 3.00 equiv) in EtOH (16.8 mL). After stirring for 1 h at rt, 4-methoxybenzaldehyde (593 μL, 4.88 mmol, 1.00 equiv) was added and the reaction mixture was stirred overnight. The resulting yellow suspension was poured into H_2_O and acidified to pH = 1 with HCl (10 wt% in H_2_O). The yellow precipitate was filtered, washed with H_2_O and dried under reduced pressure. The desired compound **12j** was obtained as a yellow solid (1.49 g, 4.67 mmol, 96%). *R*_f_ = 0.31 (petroleum ether/EtOAc 4:1); ^**1**^**H NMR** (CDCl_3_, 400 MHz): δ [ppm] 12.60 (s, 1H, O*H*), 7.76 (d, *J* = 15.5 Hz, 1H, C(O)CH=C*H*), 7.63 (d, *J* = 15.4 Hz, 1H, C(O)C*H*), 7.59 (dt, *J* = 8.7, 2.4 Hz, 2H, 2× Ar*H*), 6.95 (dt, *J* = 8.8, 2.4 Hz, 2H, 2× Ar*H*), 6.58 (d, *J* = 2.5 Hz, 1H, Ar*H*), 6.41 (d, *J* = 2.5 Hz, 1H, Ar*H*), 3.86 (s, 3H, OC*H*_3_), 3.84 (s, 3H, OC*H*_3_); ^**13**^**C NMR** (CDCl_3_, 100 MHz): δ [ppm] 193.3 (q, *C*=O), 165.8 (q, Ar*C*), 164.0 (q, Ar*C*), 163.0 (q, Ar*C*), 143.2 (t, C(O)CH=*C*H), 135.6 (q, Ar*C*), 130.6 (t, 2× Ar*C*H), 127.8 (q, Ar*C*), 124.3 (t, C(O)*C*H), 115.0 (q, Ar*C*), 114.6 (t, 2× Ar*C*H), 110.9 (t, Ar*C*H), 100.4 (t, Ar*C*H), 55.9 (p, O*C*H_3_), 55.6 (p, O*C*H_3_); **HRMS (ESI**^–^**)***m*/*z* calcd for C_17_H_14_ClO_4_ [M–H]^−^ 317.0581, found 317.0593.

#### 5-Chloro-3-hydroxy-7-methoxy-2-(4-methoxyphenyl)-4*H*-chromen-4-one (**8j**)

To a suspension of chalcone **12j** (1.49 g, 4.67 mmol, 1.00 equiv) in MeOH (40.2 mL), NaOH (3.00 M, aq., 6.03 mL, 18.1 mmol, 3.87 equiv) was added and cooled to 0 °C. H_2_O_2_ (30 wt% in H_2_O, 1.52 mL, 15.0 mmol, 3.20 equiv) was then added dropwise and the solution was stirred at 0 °C for 3 h. Subsequently, the cooling bath was removed and the mixture was stirred for another 20 h. Then, HCl (10 wt% in H_2_O) was added, leading to the formation of a yellow precipitate. The suspension was then extracted with CH_2_Cl_2_ (4×). The combined organic layers were dried over MgSO_4_, filtered and concentrated under reduced pressure. The crude material was purified by recrystallization from EtOH to give the desired product **8j** as a yellowish solid (192 mg, 595 μmol, 13%). *R*_f_ = 0.33 (petroleum ether/EtOAc 3:2); ^**1**^**H NMR** (CDCl_3_, 400 MHz): δ [ppm] 8.16 (dt, *J* = 9.1, 2.5 Hz, 2H, 2× Ar*H*), 7.20 (s, 1H, O*H*), 7.03 (dt, *J* = 9.1, 2.4 Hz, 2H, 2× Ar*H*), 6.98 (d, *J* = 2.4 Hz, 1H, Ar*H*), 6.87 (d, *J* = 2.5 Hz, 1H, Ar*H*), 3.91 (s, 3H, OC*H*_3_), 3.89 (s, 3H, OC*H*_3_); ^**13**^**C NMR** (CDCl_3_, 100 MHz): δ [ppm] 171.8 (q, *C*=O), 162.7 (q, Ar*C*), 161.1 (q, Ar*C*), 158.3 (q, Ar*C*), 143.3 (q, *C*=COH), 137.6 (q, *C*OH), 134.4 (q, Ar*C*), 129.2 (t, 2× Ar*C*H), 123.3 (q, Ar*C*), 116.9 (t, Ar*C*H), 114.2 (t, 2× Ar*C*H), 112.0 (q, Ar*C*), 99.8 (t, Ar*C*H), 56.2 (p, O*C*H_3_), 55.5 (p, O*C*H_3_); **HRMS (CI**^**+**^**)***m*/*z* calcd for C_17_H_14_ClO_5_ [M+H]^+^ 333.0530, found 333.0514.

#### (±)-Methyl (1*R*,2*R*,3*S*,3a*R*,8b*S*)-8-chloro-1,8b-dihydroxy-6-methoxy-3a-(4-methoxyphenyl)-3-phenyl-2,3,3a,8b-tetrahydro-1*H*-cyclopenta[*b*]benzofuran-2-carboxylate (**9j**)

Methyl cinnamate (1.37 g, 8.45 mmol, 14.2 equiv) was added to a solution of flavonol **8j** (192 mg, 595 μmol, 1.00 equiv) in dry chloroform (11.7 mL) and freshly distilled 2,2,2-trifluoroethanol (4.96 mL). The reaction mixture was degassed for 30 min, then cooled to −5 °C and irradiated with UV light (λ_max_ = 365 nm) until it no longer fluoresced greenish (20 h). Subsequently, the solvent was removed under reduced pressure. The remaining amount of methyl cinnamate was then removed by column chromatography (petroleum ether/EtOAc 9:1 → 1:1). The desired cycloadduct was obtained as a mixture of isomers as a yellowish solid (289 mg). Without any further purification the product of the first step (289 mg, 584 μmol, 1.00 equiv) was dissolved in MeOH (21.6 mL). Then NaOMe solution (315 μL, 25 wt% in MeOH, 1.90 mmol, 3.25 equiv) was added and the mixture was heated under refluxing conditions for 1 h. Subsequently, the reaction was terminated by the addition of NH_4_Cl solution (sat., aq.). The phases were separated and the aqueous phase was extracted with EtOAc (3×). The organic phases were combined, dried over MgSO_4_, filtered and concentrated under reduced pressure. Product **E27** was obtained as a mixture of isomers as a yellow foam (289 mg) and used directly for the next step. The desired keto ester was obtained as a mixture of isomers as a yellow foam (289 mg) and used directly for the next step. A mixture of (CH_3_)_4_N(OAc)_3_BH (986 mg, 3.75 mmol, 6.42 equiv) and freshly distilled AcOH (348 μL, 6.08 mmol, 10.4 equiv) in MeCN (15.2 mL) was stirred for 5 min at rt. Then, a solution of the product of the second step (289 mg, 584 μmol, 1.00 equiv) in MeCN (10.1 mL) was added. The mixture was protected from light and stirred for 19 h at rt. The reaction was then terminated by adding NH_4_Cl solution (sat., aq.) and sodium potassium tartrate solution (aq., 2.00 M). The phases were separated and the aqueous layer was extracted with CH_2_Cl_2_ (3×). The combined organic layers were dried over MgSO_4_, filtered and concentrated under reduced pressure. Column chromatography (CH_2_Cl_2_/EtOAc 1:0 → 9:1) was then performed to obtain the racemic *endo*-product **9j** as a colorless foam (160 mg, 323 μmol, 54% over three steps). *R*_f_ = 0.46 (CH_2_Cl_2_/EtOAc 19:1); ^**1**^**H NMR** (DMSO-*d*_6_, 400 MHz): δ [ppm] 7.07–6.94 (m, 7H, *H*-7, *H*-2′, H-6′, *H*-2″, *H*-3″, *H*-4″, *H*-5″, *H*-6″), 6.61 (d, *J* = 2.1 Hz, 1H, *H*-5), 6.56 (d, *J* = 9.0 Hz, 2H, *H*-3′, *H*-5′), 6.49 (d, *J* = 2.1 Hz, 1H, *H*-7), 5.56 (d, *J* = 6.1 Hz, 1H, O*H*-1), 5.43 (s, 1H, O*H*-8b), 4.65 (dd, *J* = 5.6, 4.9 Hz, 1H, *H*-1), 4.34 (d, *J* = 14.0 Hz, 1H, *H*-3), 4.02 (dd, *J* = 14.0, 4.5 Hz, 1H, *H*-2), 3.78 (s, 3H, *H*_3_CO-8), 3.58 (s, 6H, *H*_3_CO-11, *H*_3_CO-4′); ^**13**^**C NMR** (DMSO-*d*_6_, 100 MHz): δ [ppm] 170.4 (q, *C*-11), 161.6 (q, *C*-6), 161.1 (q, *C*-4a), 157.5 (q, *C*-4′), 138.3 (q, *C*-1″), 131.9 (q, *C*-8), 128.6 (q, *C*-1′), 128.6 (t, *C*-2′, *C*-6′), 127.8 (t, *C*-3″, *C*-5″), 127.5 (t, *C*-2″, *C*-6″), 125.8 (t, *C*-4″), 118.5 (q, *C*-8a), 111.8 (t, *C*-3′, *C*-5′), 107.6 (t, *C*-7), 101.9 (q, *C*-3a), 94.8 (t, *C*-5), 93.6 (q, *C*-8b), 78.2 (t, *C*-1), 55.9 (p, H_3_*C*O-6), 54.9 (t, *C*-3), 54.7 (p, H_3_*C*O-4′), 51.7 (t, *C*-2), 51.5 (p, H_3_*C*O-11); **HRMS (ESI**^**+**^**)***m*/*z* calcd for C_27_H_25_ClO_7_Na [M+Na]^+^ 519.1187 found 519.1182; **HPLC purity** 99.76%.

### Synthesis of (±)-Methyl (1*R*,2*R*,3*S*,3a*R*,8b*S*)-6-chloro-1,8b-dihydroxy-8-methoxy-3a-(4-methoxyphenyl)-3-phenyl-2,3,3a,8b-tetrahydro-1*H*-cyclopenta[*b*]benzofuran-2-carboxylate (**9k**)

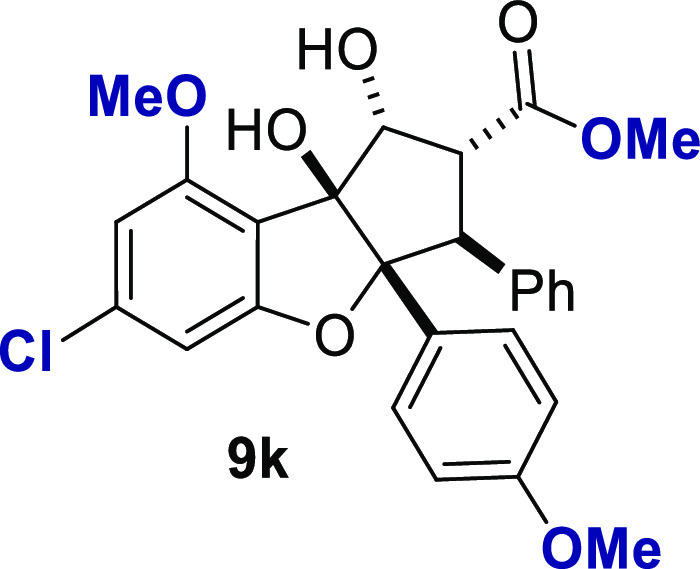



#### (*E*)-1-(4-Chloro-2-hydroxy-6-methoxyphenyl)-3-(4-methoxyphenyl)prop-2-en-1-one (**12k**)

Acetophenone **3k** (814 mg, 4.06 mmol, 1.00 equiv) was added to a solution of NaOEt (828 mg, 12.2 mmol, 3.00 equiv) in EtOH (14.0 mL). After stirring for 1 h at rt, 4-methoxybenzaldehyde (493 μL, 4.05 mmol, 1.00 equiv) was added and the reaction mixture was stirred overnight. The resulting yellow suspension was poured into H_2_O and acidified to pH = 1 with HCl (10 wt% in H_2_O). The yellow precipitate was filtered, washed with H_2_O and dried under reduced pressure. The desired compound **12k** was obtained as a yellow solid (1.22 g, 3.92 mmol, 94%). *R*_f_ = 0.28 (petroleum ether/EtOAc 4:1); ^**1**^**H NMR** (CDCl_3_, 400 MHz): δ [ppm] 13.62 (s, 1H, O*H*), 7.83 (d, *J* = 15.6 Hz, 1H, C(O)CH=C*H*), 7.72 (d, *J* = 15.5 Hz, 1H, C(O)C*H*), 7.58 (dt, *J* = 8.8, 2.4 Hz, 2H, 2× Ar*H*), 6.95 (dt, *J* = 8.8, 2.4 Hz, 2H, 2× Ar*H*), 6.58 (d, *J* = 2.5 Hz, 1H, Ar*H*), 6.41 (d, *J* = 2.5 Hz, 1H, Ar*H*), 3.96 (s, 3H, OC*H*_3_), 3.86 (s, 3H, OC*H*_3_); ^**13**^**C NMR** (CDCl_3_, 100 MHz): δ [ppm] 193.7 (q, *C*=O), 165.7 (q, Ar*C*), 161.9 (q, Ar*C*), 161.5 (q, Ar*C*), 144.0 (t, C(O)CH=*C*H), 141.8 (q, Ar*C*), 130.5 (t, 2× Ar*C*H), 128.0 (q, Ar*C*), 124.7 (t, C(O)*C*H), 114.7 (t, 2× Ar*C*H), 111.5 (t, Ar*C*H), 110.6 (q, Ar*C*), 102.9 (t, Ar*C*H), 56.4 (p, O*C*H_3_), 55.6 (p, O*C*H_3_); **HRMS (ESI**^–^**)***m*/*z* calcd for C_17_H_14_ClO_4_ [M–H]^−^ 317.0578, found 317.0593.

#### 7-Chloro-3-hydroxy-5-methoxy-2-(4-methoxyphenyl)-4*H*-chromen-4-one (**8k**)

To a suspension of chalcone **12k** (935 mg, 2.93 mmol, 1.00 equiv) in MeOH (25.2 mL), NaOH (3.00 M, aq., 3.78 mL, 11.4 mmol, 3.87 equiv) was added and cooled to 0 °C. H_2_O_2_ (30 wt% in H_2_O, 957 μL, 9.39 mmol, 3.20 equiv) was then added dropwise and the solution was stirred at 0 °C for 3 h. Subsequently, the cooling bath was removed and the mixture was stirred for another 20 h. Then, HCl (10 wt% in H_2_O) was added, leading to the formation of a yellow precipitate. The suspension was then extracted with CH_2_Cl_2_ (4×). The combined organic layers were dried over MgSO_4_, filtered and concentrated under reduced pressure. The crude material was purified by recrystallization from EtOH to give the desired product **8k** as a bright orange solid (305 mg, 945 μmol, 32%). *R*_f_ = 0.21 (petroleum ether/EtOAc 3:2); ^**1**^**H NMR** (CDCl_3_, 400 MHz): δ [ppm] 8.16 (d, *J* = 8.7 Hz, 2H, 2× Ar*H*), 7.28 (s, 1H, O*H*), 7.17 (s, 1H, Ar*H*), 7.03 (d, *J* = 8.8 Hz, 2H, 2× Ar*H*), 6.75 (s, 1H, Ar*H*), 4.02 (s, 3H, OC*H*_3_), 3.89 (s, 3H, OC*H*_3_); ^**13**^**C NMR** (CDCl_3_, 100 MHz): δ [ppm] 172.1 (q, *C*=O), 161.1 (q, Ar*C*), 160.1 (q, Ar*C*), 157.1 (q, Ar*C*), 143.1 (q, *C*=COH), 139.9 (q, Ar*C*), 138.0 (q, *C*OH), 129.3 (t, 2× Ar*C*H), 123.1 (q, Ar*C*), 114.2 (t, 2× Ar*C*H), 110.6 (t, Ar*C*H), 110.2 (q, Ar*C*), 106.5 (t, Ar*C*H), 56.9 (p, O*C*H_3_), 55.5 (p, O*C*H_3_); **HRMS (CI**^**+**^**)***m*/*z* calcd for C_17_H_14_ClO_5_ [M+H]^+^ 333.0530, found 333.0515.

#### (±)-Methyl (1*R*,2*R*,3*S*,3a*R*,8b*S*)-6-chloro-1,8b-dihydroxy-8-methoxy-3a-(4-methoxyphenyl)-3-phenyl-2,3,3a,8b-tetrahydro-1*H*-cyclopenta[*b*]benzofuran-2-carboxylate (**9k**)

Methyl cinnamate (2.18 g, 13.4 mmol, 14.2 equiv) was added to a solution of flavonol **8k** (305 mg, 945 μmol, 1.00 equiv) in dry chloroform (18.5 mL) and freshly distilled 2,2,2-trifluoroethanol (7.88 mL). The reaction mixture was degassed for 30 min, then cooled to −5 °C and irradiated with UV light (λ_max_ = 365 nm) until it no longer fluoresced greenish (20 h). Subsequently, the solvent was removed under reduced pressure. The remaining amount of methyl cinnamate was then removed by column chromatography (petroleum ether/EtOAc 9:1 → 1:1). The desired cycloadduct was obtained as a mixture of isomers as a yellowish solid (421 mg). Without any further purification the product of the first step (421 mg, 850 μmol, 1.00 equiv) was dissolved in MeOH (31.5 mL). Then NaOMe solution (459 μL, 25 wt% in MeOH, 2.76 mmol, 3.25 equiv) was added and the mixture was heated under refluxing conditions for 1 h. Subsequently, the reaction was terminated by the addition of NH_4_Cl solution (sat., aq.). The phases were separated and the aqueous phase was extracted with EtOAc (3×). The organic phases were combined, dried over MgSO_4_, filtered and the solvent was removed under reduced pressure. The desired keto ester was obtained as a mixture of isomers as a yellow foam (421 mg) and used directly for the next step. A mixture of (CH_3_)_4_N(OAc)_3_BH (1.44 g, 5.45 mmol, 6.42 equiv) and freshly distilled AcOH (506 μL, 8.84 mmol, 10.4 equiv) in MeCN (22.1 mL) was stirred for 5 min at rt. Then, a solution of the product of the second step (421 mg, 850 μmol, 1.00 equiv) in MeCN (14.7 mL) was added. The mixture was protected from light and stirred for 19 h at rt. The reaction was then terminated by adding NH_4_Cl solution (sat., aq.) and sodium potassium tartrate solution (aq., 2.00 M). The phases were separated and the aqueous layer was extracted with CH_2_Cl_2_ (3×). The combined organic layers were dried over MgSO_4_, filtered and concentrated under reduced pressure. Column chromatography (CH_2_Cl_2_/EtOAc 1:0 → 9:1) was then performed to obtain the racemic *endo*-product **9k** as a yellowish foam (225 mg, 452 μmol, 48% over three steps). *R*_f_ = 0.31 (CH_2_Cl_2_/EtOAc 19:1); ^**1**^**H NMR** (DMSO-*d*_6_, 400 MHz): δ [ppm] 7.06–6.95 (m, 5H, *H*-7, *H*-2′, H-6′, *H*-2″, *H*-4″, *H*-6″), 6.91 (d, *J* = 7.3 Hz, 2H, *H*-3″, *H*-5″), 6.74 (d, *J* = 1.6 Hz, 1H, *H*-7), 6.59 (d, *J* = 1.5 Hz, 1H, *H*-5), 6.57 (d, *J* = 9.0 Hz, 2H, *H*-3′, *H*-5′), 5.33–5.32 (m, 2H, O*H*-1, O*H*-8b), 4.69 (t, *J* = 5.2 Hz, 1H, *H*-1), 4.22 (d, *J* = 14.0 Hz, 1H, *H*-3), 3.97 (dd, *J* = 14.0, 5.1 Hz, 1H, *H*-2), 3.75 (s, 3H, *H*_3_CO-8), 3.58 (s, 3H, *H*_3_CO-4″), 3.55 (s, 3H, *H*_3_CO-11); ^**13**^**C NMR** (DMSO-*d*_6_, 100 MHz): δ [ppm] 170.4 (q, *C*-11), 160.2 (q, *C*-4a), 158.1 (q, *C*-8), 157.6 (q, *C*-4′), 138.2 (q, *C*-1″), 134.8 (q, *C*-6), 128.7 (t, *C*-2′, *C*-6′), 128.3 (q, *C*-1′), 127.8 (t, *C*-3″, *C*-5″), 127.5 (t, *C*-2″, *C*-6″), 125.9 (t, *C*-4″), 114.8 (q, *C*-8a), 111.9 (t, *C*-3′, *C*-5′), 104.5 (t, *C*-5), 103.4 (t, *C*-7), 101.8 (q, *C*-3a), 93.2 (q, *C*-8b), 78.6 (t, *C*-1), 55.9 (p, H_3_*C*O-8), 54.85 (t, *C*-3), 54.81 (p, H_3_*C*O-4′), 51.5 (p, H_3_*C*O-11), 51.3 (t, *C*-2); **HRMS (ESI**^**+**^**)***m*/*z* calcd for C_27_H_25_ClO_7_Na [M+Na]^+^ 519.1187 found 519.1173; **HPLC purity** 99.66%. The analytical data are consistent with those reported in the literature.^20^

### Synthesis of (±)-Methyl (1*R*,2*R*,3*S*,3a*R*,8b*S*)-8-bromo-1,8b-dihydroxy-6-methoxy-3a-(4-methoxyphenyl)-3-phenyl-2,3,3a,8b-tetrahydro-1*H*-cyclopenta[*b*]benzofuran-2-carboxylate (**9l**)

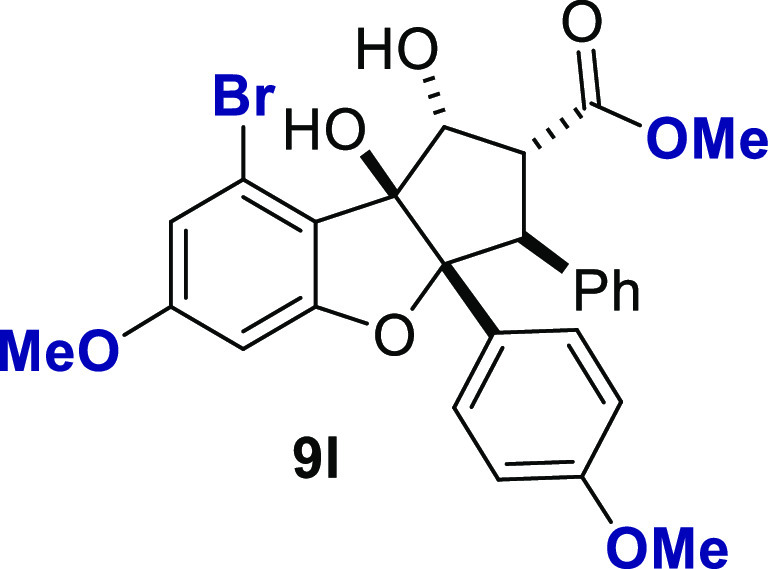



#### (*E*)-1-(2-Bromo-6-hydroxy-4-methoxyphenyl)-3-(4-methoxyphenyl)prop-2-en-1-one (**12l**)

Acetophenone **3l** (430 mg, 1.75 mmol, 1.00 equiv) was added to a solution of NaOEt (358 mg, 5.26 mmol, 3.00 equiv) in EtOH (6.05 mL). After stirring for 1 h at rt, 4-methoxybenzaldehyde (213 μL, 1.75 mmol, 1.00 equiv) was added and the reaction mixture was stirred overnight. The resulting yellow suspension was poured into H_2_O and acidified to pH = 1 with HCl (10 wt% in H_2_O). The yellow precipitate was filtered, washed with H_2_O and dried under reduced pressure. The desired compound **12l** was obtained as a yellow solid (617 mg, 1.70 mmol, 97%). *R*_f_ = 0.34 (petroleum ether/EtOAc 4:1); ^**1**^**H NMR** (CDCl_3_, 400 MHz): δ [ppm] 12.14 (s, 1H, O*H*), 7.74 (d, *J* = 15.6 Hz, 1H, C(O)CH=C*H*), 7.62 (d, *J* = 15.4 Hz, 1H, C(O)C*H*), 7.59 (d, *J* = 8.6, 2H, 2× Ar*H*), 6.94 (dt, *J* = 8.8 Hz, 2H, 2× Ar*H*), 6.82 (d, *J* = 2.6 Hz, 1H, Ar*H*), 6.45 (d, *J* = 2.6 Hz, 1H, Ar*H*), 3.86 (s, 3H, OC*H*_3_), 3.83 (s, 3H, OC*H*_3_); ^**13**^**C NMR** (CDCl_3_, 100 MHz): δ [ppm] 193.9 (q, *C*=O), 165.1 (q, Ar*C*), 164.0 (q, Ar*C*), 162.0 (q, Ar*C*), 142.7 (t, C(O)CH=*C*H), 130.6 (t, 2× Ar*C*H), 127.9 (q, Ar*C*), 124.3 (t, C(O)*C*H), 123.6 (q, Ar*C*), 117.0 (q, Ar*C*), 114.7 (t, 2× Ar*C*H), 114.5 (t, Ar*C*H), 100.9 (t, Ar*C*H), 55.9 (p, O*C*H_3_), 55.6 (p, O*C*H_3_); **HRMS (ESI**^–^**)***m*/*z* calcd for C_17_H_14_BrO_4_ [M–H]^−^ 361.0075, found 361.0071.

#### 5-Bromo-3-hydroxy-7-methoxy-2-(4-methoxyphenyl)-4*H*-chromen-4-one (**8l**)

To a suspension of chalcone **12l** (617 mg, 1.70 mmol, 1.00 equiv) in MeOH (14.6 mL), NaOH (3.00 M, aq., 2.19 mL, 6.57 mmol, 3.87 equiv) was added and the mixture was stirred for 1 h at rt. Subsequently, the solution was cooled to 0 °C, H_2_O_2_ (30 wt% in H_2_O, 554 μL, 5.44 mmol, 3.20 equiv) was added dropwise. After 3 h stirring at the same temperature, the cooling bath was removed and the mixture was stirred for another 20 h. HCl (10 wt% in H_2_O) was then added leading to the formation of a yellow precipitate. The suspension was then extracted with CH_2_Cl_2_ (3×). The combined organic layers were dried over MgSO_4_, filtered and concentrated under reduced pressure. The crude material was purified by recrystallization from EtOH to give the desired product **8l** as a bright yellow solid (135 mg, 358 μmol, 21%). *R*_f_ = 0.52 (petroleum ether/EtOAc 1:1); ^**1**^**H NMR** (CDCl_3_, 400 MHz): δ [ppm] 8.18 (d, *J* = 8.5 Hz, 2H, 2× Ar*H*), 7.26 (s, 1H, O*H*), 7.19 (s, 1H, Ar*H*)), 7.04 (d, *J* = 8.2 Hz, 2H, 2× Ar*H*), 6.94 (s, 1H, Ar*H*), 3.93 (s, 3H, OC*H*_3_), 3.89 (s, 3H, OC*H*_3_); ^**13**^**C NMR** (CDCl_3_, 100 MHz): δ [ppm] 171.8 (q, *C*=O), 162.8 (q, Ar*C*), 161.1 (q, Ar*C*), 158.1 (q, Ar*C*), 143.3 (q, *C*=COH), 137.3 (q, *C*OH), 129.2 (t, 2× Ar*C*H), 123.3 (q, Ar*C*), 121.1 (q, Ar*C*), 120.7 (t, Ar*C*H), 114.2 (t, 2× Ar*C*H), 112.6 (q, Ar*C*), 100.5 (t, Ar*C*H), 56.2 (p, O*C*H_3_), 55.6 (p, O*C*H_3_); **HRMS (EI)***m*/*z* calcd for C_17_H_13_BrO_5_ [M]^+^ 375.9946, found 375.9948.

#### (±)-Methyl (1*R*,2*R*,3*S*,3a*R*,8b*S*)-8-bromo-1,8b-dihydroxy-6-methoxy-3a-(4-methoxyphenyl)-3-phenyl-2,3,3a,8b-tetrahydro-1*H*-cyclopenta[*b*]benzofuran-2-carboxylate (**9l**)

Methyl cinnamate (1.08 g, 6.63 mmol, 14.2 equiv) was added to a solution of flavonol **8l** (176 mg, 467 μmol, 1.00 equiv) in dry chloroform (9.15 mL) and freshly distilled 2,2,2-trifluoroethanol (3.89 mL). The reaction mixture was degassed for 30 min, then cooled to −5 °C and irradiated with UV light (λ_max_ = 365 nm) until it no longer fluoresced greenish (20 h). Subsequently, the solvent was removed under reduced pressure. The remaining amount of methyl cinnamate was then removed by column chromatography (petroleum ether/EtOAc 9:1 → 1:1). The desired cycloadduct was obtained as a mixture of isomers as a yellowish solid (252 mg). Without any further purification the product of the first step (252 mg, 467 μmol, 1.00 equiv) was dissolved in MeOH (17.3 mL). Then NaOMe solution (252 μL, 25 wt% in MeOH, 1.52 mmol, 3.25 equiv) was added and the mixture was heated under refluxing conditions for 1 h. Subsequently, the reaction was terminated by the addition of NH_4_Cl solution (sat., aq.). The phases were separated and the aqueous phase was extracted with EtOAc (3×). The organic phases were combined, dried over MgSO_4_, filtered and the solvent was removed under reduced pressure. The desired keto ester was obtained as a mixture of isomers as a yellow foam (233 mg) and used directly for the next step. A mixture of (CH_3_)_4_N(OAc)_3_BH (730 mg, 2.77 mmol, 6.42 equiv) and freshly distilled AcOH (257 μL, 4.50 mmol, 10.4 equiv) in MeCN (11.2 mL) was stirred for 5 min at rt. Then, a solution of the product of the second step (233 mg, 432 μmol, 1.00 equiv) in MeCN (14.7 mL) was added. The mixture was protected from light and stirred for 19 h at rt. The reaction was then terminated by adding NH_4_Cl solution (sat., aq.) and sodium potassium tartrate solution (aq., 2.00 M). The phases were separated and the aqueous layer was extracted with CH_2_Cl_2_ (3×). The combined organic layers were dried over MgSO_4_, filtered and concentrated under reduced pressure. Column chromatography (CH_2_Cl_2_/EtOAc 1:0 → 9:1) was then performed to obtain the racemic *endo*-product **9l** as a yellow foam (92.4 mg, 171 μmol, 37% over three steps). *R*_f_ = 0.41 (CH_2_Cl_2_/EtOAc 19:1); ^**1**^**H NMR** (DMSO-*d*_6_, 400 MHz): δ [ppm] 7.07–6.94 (m, 7H, *H*-7, *H*-2′, H-6′, *H*-2″, *H*-3″, *H*-4″, *H*-5″, *H*-6″), 6.65 (d, *J* = 2.1 Hz, 1H, *H*-5), 6.63 (d, *J* = 2.1 Hz, 1H, *H*-7), 6.55 (d, *J* = 8.8 Hz, 2H, *H*-3′, *H*-5′), 5.48 (d, *J* = 5.9 Hz, 1H, O*H*-1), 5.38 (s, 1H, O*H*-8b), 4.65 (dd, *J* = 5.6, 4.5 Hz, 1H, *H*-1), 4.39 (d, *J* = 13.9 Hz, 1H, *H*-3), 4.02 (dd, *J* = 13.9, 4.4 Hz, 1H, *H*-2), 3.78 (s, 3H, *H*_3_CO-8), 3.59 (s, 3H, *H*_3_CO-11), 3.58 (s, 3H, *H*_3_CO-4′); ^**13**^**C NMR** (DMSO-*d*_6_, 100 MHz): δ [ppm] 170.4 (q, *C*-11), 161.6 (q, *C*-6), 161.3 (q, *C*-4a), 157.5 (q, *C*-4′), 138.3 (q, *C*-1″), 128.7 (q, *C*-1′), 128.6 (t, *C*-2′, *C*-6′), 127.8 (t, *C*-3″, *C*-5″), 127.5 (t, *C*-2″, *C*-6″), 125.7 (t, *C*-4″), 120.3 (q, *C*-8), 120.1 (q, *C*-8a), 111.8 (t, *C*-3′, *C*-5′), 110.5 (t, *C*-7), 102.0 (q, *C*-3a), 95.2 (t, *C*-5), 94.0 (q, *C*-8b), 78.1 (t, *C*-1), 55.8 (p, H_3_*C*O-8), 54.8 (t, *C*-3), 54.7 (p, H_3_*C*O-4′), 51.7 (t, *C*-2), 51.5 (p, H_3_*C*O-11); **HRMS (ESI**^**+**^**)***m*/*z* calcd for C_27_H_25_BrO_7_Na [M+Na]^+^ 563.0681 found 563.0663; **HPLC purity** 99.70%.

### Synthesis of (±)-Methyl (1*R*,2*R*,3*S*,3a*R*,8b*S*)-6-bromo-1,8b-dihydroxy-8-methoxy-3a-(4-methoxyphenyl)-3-phenyl-2,3,3a,8b-tetrahydro-1*H*-cyclopenta[*b*]benzofuran-2-carboxylate (**9m**)

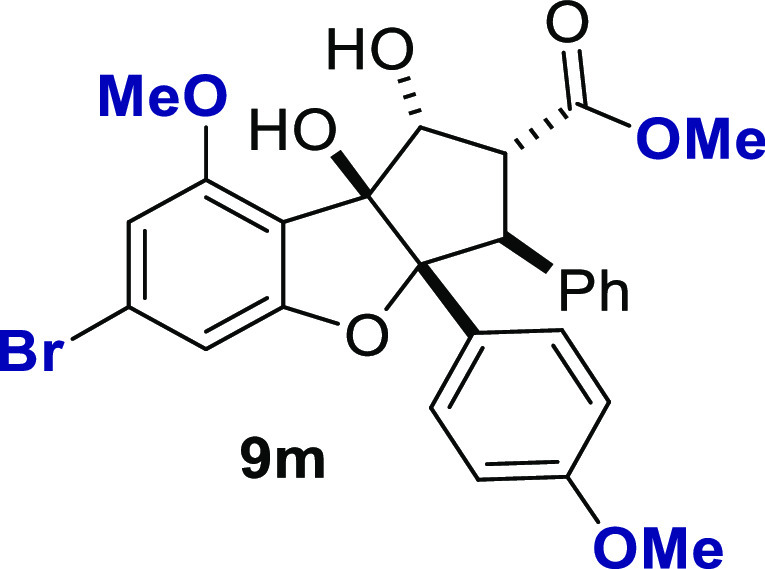



#### (*E*)-1-(4-Bromo-2-hydroxy-6-methoxyphenyl)-3-(4-methoxyphenyl)prop-2-en-1-one (**12m**)

Acetophenone **3m** (926 mg, 3.78 mmol, 1.00 equiv) was added to a solution of NaOEt (771 mg, 11.3 mmol, 3.00 equiv) in EtOH (13.0 mL). After stirring for 1 h at rt, 4-methoxybenzaldehyde (459 μL, 3.78 mmol, 1.00 equiv) was added and the reaction mixture was stirred overnight. The resulting yellow suspension was poured into H_2_O and acidified to pH = 1 with HCl (10 wt% in H_2_O). The yellow precipitate was filtered, washed with H_2_O and dried under reduced pressure. The desired compound **12m** was obtained as a yellow solid (970 mg, 2.67 mmol, 71%). *R*_f_ = 0.29 (petroleum ether/EtOAc 4:1); ^**1**^**H NMR** (CDCl_3_, 400 MHz): δ [ppm] 13.56 (s, 1H, O*H*), 7.83 (d, *J* = 15.5 Hz, 1H, C(O)CH=C*H*), 7.71 (d, *J* = 15.5 Hz, 1H, C(O)C*H*), 7.57 (d, *J* = 8.7 Hz, 2H, 2× Ar*H*), 6.94 (dt, *J* = 8.7 Hz, 2H, 2× Ar*H*), 6.81 (d, *J* = 1.8 Hz, 1H, Ar*H*), 6.58 (d, *J* = 1.7 Hz, 1H, Ar*H*), 3.95 (s, 3H, OC*H*_3_), 3.86 (s, 3H, OC*H*_3_); ^**13**^**C NMR** (CDCl_3_, 100 MHz): δ [ppm] 193.9 (q, *C*=O), 165.5 (q, Ar*C*), 161.9 (q, Ar*C*), 161.2 (q, Ar*C*), 144.0 (t, C(O)CH=*C*H), 130.5 (t, 2× Ar*C*H), 130.2 (q, Ar*C*), 128.0 (q, Ar*C*), 124.7 (t, C(O)*C*H), 114.61 (t, Ar*C*H), 114.59 (t, 2× Ar*C*H), 110.9 (q, Ar*C*), 105.8 (t, Ar*C*H), 56.4 (p, O*C*H_3_), 55.6 (p, O*C*H_3_); **HRMS (ESI**^–^**)***m*/*z* calcd for C_17_H_14_BrO_4_ [M–H]^−^ 361.0075, found 361.0076.

#### 7-Bromo-3-hydroxy-5-methoxy-2-(4-methoxyphenyl)-4*H*-chromen-4-one (**8m**)

To a suspension of chalcone **12m** (960 mg, 2.64 mmol, 1.00 equiv) in MeOH (22.7 mL), NaOH (3.00 M, aq., 3.41 mL, 10.2 mmol, 3.87 equiv) was added and the mixture was stirred for 1 h at rt. Subsequently, the solution was cooled to 0 °C, H_2_O_2_ (30 wt% in H_2_O, 862 μL, 8.46 mmol, 3.20 equiv) was added dropwise. After 3 h stirring at the same temperature, the cooling bath was removed and the mixture was stirred for another 20 h. HCl (10 wt% in H_2_O) was then added leading to the formation of a yellow precipitate. The suspension was then extracted with CH_2_Cl_2_ (3×). The combined organic layers were dried over MgSO_4_, filtered and concentrated under reduced pressure. The crude material was purified by recrystallization from EtOH to give the desired product **8m** as a bright yellow solid (396 mg, 1.05 mmol) in 40% yield. *R*_f_ = 0.25 (petroleum ether/EtOAc 1:1); ^**1**^**H NMR** (CDCl_3_, 400 MHz): δ [ppm] 8.15 (d, *J* = 9.1 Hz, 2H, 2× Ar*H*), 7.33 (d, *J* = 1.5 Hz, 1H, Ar*H*), 7.28 (s, 1H, O*H*), 7.02 (d, *J* = 9.1 Hz, 2H, 2× Ar*H*), 6.89 (d, *J* = 1.4 Hz, 1H, Ar*H*), 4.01 (s, 3H, OC*H*_3_), 3.88 (s, 3H, OC*H*_3_); ^**13**^**C NMR** (CDCl_3_, 100 MHz): δ [ppm] 172.1 (q, *C*=O), 161.1 (q, Ar*C*), 159.9 (q, Ar*C*), 157.0 (q, Ar*C*), 143.1 (q, *C*=COH), 138.1 (q, *C*OH), 129.3 (t, 2× Ar*C*H), 127.9 (q, Ar*C*), 123.1 (q, Ar*C*), 114.2 (t, 2× Ar*C*H), 113.7 (t, Ar*C*H), 110.5 (q, Ar*C*), 109.3 (t, Ar*C*H), 56.9 (p, O*C*H_3_), 55.5 (p, O*C*H_3_); **HRMS (EI)***m*/*z* calcd for C_17_H_13_BrO_5_ [M]^+^ 375.9946, found 375.9938.

#### (±)-Methyl (1*R*,2*R*,3*S*,3a*R*,8b*S*)-6-bromo-1,8b-dihydroxy-8-methoxy-3a-(4-methoxyphenyl)-3-phenyl-2,3,3a,8b-tetrahydro-1*H*-cyclopenta[*b*]benzofuran-2-carboxylate (**9m**)

Methyl cinnamate (2.20 g, 13.6 mmol, 14.2 equiv) was added to a solution of flavonol **8m** (360 mg, 954 μmol, 1.00 equiv) in dry chloroform (18.7 mL) and freshly distilled 2,2,2-trifluoroethanol (7.95 mL). The reaction mixture was degassed for 30 min, then cooled to −5 °C and irradiated with UV light (λ_max_ = 365 nm) until it no longer fluoresced greenish (20 h). Subsequently, the solvent was removed under reduced pressure. The remaining amount of methyl cinnamate was then removed by column chromatography (petroleum ether/EtOAc 9:1 → 1:1). Product **E32** was obtained as a mixture of isomers as a yellowish solid (498 mg) and used directly for the next step. The desired cycloadduct was obtained as a mixture of isomers as a yellowish solid (498 mg). Without any further purification the product of the first step (498 mg, 923 μmol, 1.00 equiv) was dissolved in MeOH (34.2 mL). Then NaOMe solution (499 μL, 25 wt% in MeOH, 3.00 mmol, 3.25 equiv) was added and the mixture was heated under refluxing conditions for 1 h. Subsequently, the reaction was terminated by the addition of NH_4_Cl solution (sat., aq.). The phases were separated and the aqueous phase was extracted with EtOAc (3×). The organic phases were combined, dried over MgSO_4_, filtered and the solvent was removed under reduced pressure. The desired keto ester was obtained as a mixture of isomers as a yellow solid (451 mg) and used directly for the next step. A mixture of (CH_3_)_4_N(OAc)_3_BH (1.41 g, 5.37 mmol, 6.42 equiv) and freshly distilled AcOH (498 μL, 8.70 mmol, 10.4 equiv) in MeCN (21.7 mL) was stirred for 5 min at rt. Then, a solution of the product of the second step (451 mg, 836 μmol, 1.00 equiv) in MeCN (14.4 mL) was added. The mixture was protected from light and stirred for 19 h at rt. The reaction was then terminated by adding NH_4_Cl solution (sat., aq.) and sodium potassium tartrate solution (aq., 2.00 M). The phases were separated and the aqueous layer was extracted with CH_2_Cl_2_ (3×). The combined organic layers were dried over MgSO_4_, filtered and concentrated under reduced pressure. Column chromatography (CH_2_Cl_2_/EtOAc 1:0 → 9:1) was then performed to obtain the racemic *endo*-product **9m** as a yellow foam (228 mg, 421 μmol, 44% over three steps). *R*_f_ = 0.30 (CH_2_Cl_2_/EtOAc 19:1); ^**1**^**H NMR** (DMSO-*d*_6_, 400 MHz): δ [ppm] 7.07–6.96 (m, 5H, *H*-2′, H-6′, *H*-2″, *H*-4″, *H*-6″), 6.91 (d, *J* = 7.4 Hz, 2H, *H*-3″, *H*-5″), 6.87 (d, *J* = 1.2 Hz, 1H, *H*-5), 6.71 (d, *J* = 1.3 Hz, 1H, *H*-7), 6.57 (d, *J* = 8.9 Hz, 2H, *H*-3′, *H*-5′), 5.32–5.31 (m, 2H, O*H*-1, O*H*-8b), 4.66 (t, *J* = 5.1 Hz, 1H, *H*-1), 4.22 (d, *J* = 14.0 Hz, 1H, *H*-3), 3.97 (dd, *J* = 14.0, 5.0 Hz, 1H, *H*-2), 3.76 (s, 3H, *H*_3_CO-8), 3.59 (s, 3H, *H*_3_CO-4′), 3.56 (s, 3H, *H*_3_CO-11); ^**13**^**C NMR** (DMSO-*d*_6_, 100 MHz): δ [ppm] 170.3 (q, *C*-11), 160.4 (q, *C*-4a), 158.2 (q, *C*-8), 157.5 (q, *C*-4′), 138.1 (q, *C*-1″), 128.6 (t, *C*-2′, *C*-6′), 128.2 (q, *C*-1′), 127.8 (t, *C*-3″, *C*-5″), 127.5 (t, *C*-2″, *C*-6″), 125.8 (t, *C*-4″), 122.8 (q, *C*-6), 115.2 (q, *C*-8a), 111.8 (t, *C*-3′, *C*-5′), 107.2 (t, *C*-7), 106.3 (t, *C*-5), 101.7 (q, *C*-3a), 93.2 (q, *C*-8b), 78.6 (t, *C*-1), 55.8 (p, H_3_*C*O-8), 54.8 (t, *C*-3), 54.7 (p, H_3_*C*O-4′), 51.4 (p, H_3_*C*O-11), 51.2 (t, *C*-2); **HRMS (ESI**^**+**^**)***m*/*z* calcd for C_27_H_25_BrO_7_Na [M+Na]^+^ 563.0681 found 563.0665; **HPLC purity** 99.12%.

### Synthesis of (±)-Methyl (1*R*,2*R*,3*S*,3a*R*,8b*S*)-8-fluoro-1,8b-dihydroxy-6-(methoxymethoxy)-3a-(4-methoxyphenyl)-3-phenyl-2,3,3a,8b-tetrahydro-1*H*-cyclopenta[*b*]benzofuran-2-carboxylate (**9na**)

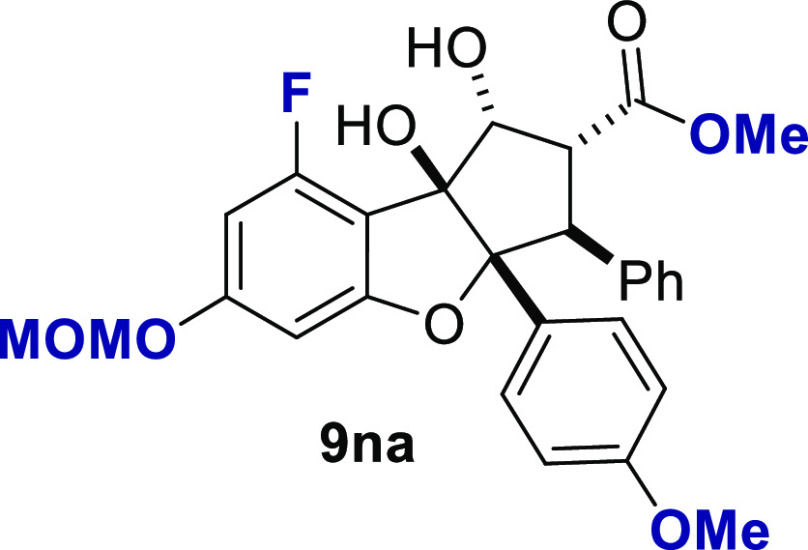



#### (*E*)-1-(2-Fluoro-6-hydroxy-4-(methoxymethoxy)phenyl)-3-(4-methoxyphenyl)prop-2-en-1-one (**12na**)

A suspension of NaOEt (191 mg, 2.80 mmol, 3.00 equiv) in dry EtOH (3.1 mL) was cooled down to rt, followed by the addition of 1-(2-fluoro-6-hydroxy-4-(methoxymethoxy)phenyl)ethan-1-one (200 mg, 0.93 mmol, 1.00 equiv) at the same temperature. The suspension was stirred for 1 h, before *p*-anisaldehyde (114 μL, 0.93 mmol, 1.00 equiv) was added. The orange solution was stirred for 16 h at rt. The resulting orange suspension was poured into cold water and acidified to pH = 1 with HCl (aq., 1 M). The precipitate was filtered, washed with water, dissolved in EtOAc, dried over MgSO_4_, filtered and concentrated *in vacuo*. The crude was purified over silica gel chromatography (petroleum ether/EtOAc 10:1) to afford **12na** as a yellow-orange solid (214 mg, 0.64 mmol, 69%). *R*_f_ = 0.20 (petroleum ether/EtOAc 6:1); ^**1**^**H NMR** (CDCl_3_, 400 MHz): δ [ppm] 13.79 (s, 1H, O*H*), 7.90 (dd, *J* = 15, 3.6 Hz, C(O)CH=C*H*), 7.60 (d, *J* = 8.7 Hz, 2H, 2× Ar*H*), 7.52 (dd, *J* = 15, 1.4 Hz, 1H,C(O)*C*H=CH), 6.94 (d, *J* = 8.8 Hz, 2H, 2× Ar*H*), 6.44 (m, 1H, Ar*H*), 6.32 (dd, *J* = 14, 2.4 Hz, 1H, Ar*H*), 5.19 (s, 2H, OC*H*_2_OCH_3_), 3.86 (s, 3H, OCH_2_OC*H*_3_), 3.48 (s, 3H, C*H*_3_O); ^**13**^**C NMR** (CDCl_3_, 100 MHz): δ [ppm] 191.1 (1, *d*, *J* = 5.0 Hz, *C*=O), 166.7 (q, *d*, *J* = 7.6 Hz, Ar*C*), 164.2 (q, *d*, *J* = 254 Hz, Ar*C*), 163.2 (q, *d*, *J* = 17 Hz, Ar*C*), 160.5 (q, Ar*C*), 145.0 (t, *d*, *J* = 1.7 Hz, C(O)CH=*C*H), 130.6 (t, 2× Ar*C*), 122.8 (t, *d*, *J* = 17 Hz, C(O)*C*H=CH), 114.5 (t, 2× Ar*C*), 105.7 (q, *d*, *J* = 14 Hz, Ar*C*), 100.3 (t, *d*, *J* = 2.9 Hz, Ar*C*), 96.2 (t, *d*, *J* = 29 Hz, Ar*C*), 94.2 (s, *C*H_2_), 56.5 (p, H_3_*C*O), 55.4 (p, H_3_*C*O); **HRMS (ESI**^**+**^**)***m*/*z* calcd for C_18_H_17_O_5_FNa [M+Na]^+^: 355.0958, found 355.0952.

#### 5-Fluoro-3-hydroxy-7-(methoxymethoxy)-2-(4-methoxyphenyl)-4*H*-chromen-4-one (**8na**)

To suspension of chalcone **12na** (214 mg, 0.64 mmol, 1.00 equiv) in MeOH (7.6 mL) and NaOH (aq., 3 M, 1.08 mL, 3.22 mmol, 5.00 equiv) was added H_2_O_2_ (aq., 30%, 149 μL, 6.44 mmol, 10.0 equiv) at 0 °C. The bright orange solution was stirred for 3 h at the same temperature. The reaction was stirred for further 16 h at rt. The resulting yellow suspension was poured into a cold aqueous HCl (aq., 1 M) and extracted with CH_2_Cl_2_. The collected organic layers were washed with water, brine, dried over MgSO_4_, filtered and concentrated *in vacuo*. The crude was recrystallized in MeOH to afford flavonol **8na** (95 mg, 0.27 mmol, 42%) as pale-yellow needle crystals. *R*_f_ = 0.50 (petroleum ether/EtOAc 1:1); ^**1**^**H NMR** (CDCl_3_, 400 MHz): δ [ppm] 8.18 (d, *J* = 9.0 Hz, 2H, 2× Ar*H*), 7.04 (d, *J* = 9.1 Hz, 2H, 2× Ar*H*), 7.00 (m, 1H, Ar*H*), 6.76 (dd, *J* = 12, 2.1 Hz, 1H, Ar*H*), 5.28 (s, 2H, C*H*_2_), 3.85 (s, 3H, *H*_3_CO), 3.52 (s, 3H, H_3_CO); ^**13**^**C NMR** (CDCl_3_, 100 MHz): δ [ppm] 170.9 (q, *d*, *J* = 1.6 Hz, *C*=O), 161.3 (q, *d*, *J* = 14 Hz, Ar*C*), 161.2 (q, *d*, *J* = 262 Hz, Ar*C*), 161.0 (q, Ar*C*), 157.3 (q, *d*, *J* = 6.8 Hz, Ar*C*), 144.0 (q, Ar*C*), 137.4 (q, *C*OH), 129.2 (t, 2× Ar*C*), 123.2 (q, *C*=COH), 114.1 (t, 2× Ar*C*), 106.4 (q, *d*, *J* = 13 Hz, Ar*C*), 101.9 (t, *d*, *J* = 23 Hz, Ar*C*), 99.4 (t, *d*, *J* = 4.0 Hz, Ar*C*), 94.6 (s, *C*H_2_), 56.6 (p, *C*H_3_O), 55.4 (p, *C*H_3_O); **HRMS (ESI**^**+**^**)***m*/*z* calcd for C_18_H_15_O_6_FNa [M+Na]^+^ 369.0750, found 369.0750.

#### (±)-Methyl (1*R*,2*R*,3*S*,3a*R*,8b*S*)-8-fluoro-1,8b-dihydroxy-6-(methoxymethoxy)-3a-(4-methoxyphenyl)-3-phenyl-2,3,3a,8b-tetrahydro-1*H*-cyclopenta[*b*]benzofuran-2-carboxylate (**9na**)

To a solution of flavonol **8nb** (96 mg, 0.28 mmol, 1.00 equiv) in dry 2,2,2-TFE (2.3 mL) and dry CHCl_3_ (5.6 mL) was added methyl cinnamate (641 mg, 3.95 mmol, 14.20 equiv). The clear solution was degassed with argon for 15 min, followed by UV-irradiation (100 W, 365 nm) at −5 °C for 10–16 h. After the flavonol was fully consumed, the solvent was removed *in vacuo* and the excess of methyl cinnamate was removed by silica gel purification (petroleum ether/EtOAc 10:1, then, 4:1, then EtOAc). The cycloadduct mixture was used directly for the next step. To the solution of cycloadduct mixture (142 mg) in MeOH (9.3 mL) was added NaOMe solution (25 wt% in MeOH, 171 μL, 0.79 mmol, 2.84 equiv) and stirred under refluxing conditions for 1 h. The reaction was terminated by the addition of NH_4_Cl (sat., aq.). The aqueous layers were extracted with EtOAc. The collected organic layers were washed with NaCl (sat., aq.), dried over MgSO_4_, filtered and concentrated *in vacuo*. The yellow foam crude product was directly used for the next step without further purification. A solution of Me_4_NBH(OAc)_3_ (365 mg, 2.25 mmol, 6.42 equiv) and freshly distilled AcOH (131 μL, 2.25 mmol, 10.4 equiv) in dry MeCN (5.6 mL) was prepared and stirred at rt for 10 min. To this solution was added crude of the ketone from the previous step (110 mg) in dry MeCN (3.6 mL). The reaction was carried out under light exclusion and stirred for 19 h at rt. The reaction was terminated by the addition of NaK-tartrate (sat., aq.) and NH_4_Cl (sat., aq.). The layers were separated and the aqueous layers were extracted with CH_2_Cl_2_. The collected organic layers were washed with water and NaCl (sat., aq.), dried over MgSO_4_, filtered and concentrated *in vacuo*. The crude product was purified by silica gel column chromatography (petroleum ether/EtOAc 3:1, then 2:1), followed by HPLC purification to yield **9nb** (71 mg, 0.14 mmol, 49% over three steps) as a pale-yellow foam. *R*_f_ = 0.29 (CH_2_Cl_2_/EtOAc 10:1);); ^**1**^**H NMR** (DMSO-*d*_6_, 400 MHz): δ [ppm] 7.09–6.98 (m, 5H, *H*-2″, *H*-3″, *H*-4″, *H*-5″, *H*-6″), 6.88 (d, *J* = 7.2 Hz, 2H, *H*-2′ and *H*-6′), 6.62 (dt, *J* = 10, 2.5 Hz, 2H, *H*-3′ and *H*-5′), 6.56 (d, *J* = 2.0 Hz, 1H, *H*-5), 6.38 (dd, *J* = 11, 2.0 Hz, 1H, *H*-7), 5.83 (d, *J* = 6.4 Hz, 1H, O*H*), 5.55 (s, 1H, O*H*), 5.22 (s, 2H, OC*H*_2_OCH_3_), 4.69 (t, *J* = 6.1 Hz, 1H, *H*-1), 4.13 (d, *J* = 14 Hz, 1H, *H*-3), 3.94 (dd, *J* = 14, 5.8 Hz, 1H, *H*-2), 3.62 (s, 3H, *H*_3_CO-4′), 3.55 (s, 3H, *H*_3_CO-11), 3.41 (s, 3H, CH_2_OC*H*_3_); ^**13**^**C NMR** (DMSO-*d*_6_, 100 MHz): δ [ppm] 170.6 (q, *C*-11), 161.1 (q, *d*, *J* = 12 Hz, *C*-6), 160.5 (q, *d*, *J* = 249 Hz, *C-*8), 160.0 (q, *d*, *J* = 12 Hz, *C*-4a), 158.1 (q, *C*-4′), 134.4 (q, *C-*1″), 129.1 (t, *C*-2′, *C*-6′), 128.3 (q, *C*-1′), 128.1 (t, *C*-3″, *C-*5″), 127.9 (t, *C*-2″, *C*-6″), 126.4 (t, *C*4″), 112.4 (t, *C*-3′, *C*-5′), 110.2 (q, *d*, *J* = 20 Hz, *C*-8a), 102.2 (q, *C*-3a), 97.1 (t, *d*, *J* = 25 Hz, *C*-7), 94.8 (t, *d*, *J* = 3.3 Hz, *C*-5), 94.5 (s, O*C*H_2_OCH_3_), 93.6 (q, *d*, *J* = 2.5 Hz, *C*-8b), 78.8 (t, *C*-1), 56.2 (p, OCH_2_O*C*H_3_), 55.3 (p, *C*H_3_O-4′), 55.2 (t, *C*-3), 51.9 (p, *C*H_3_O-11), 51.7 (t, *C*-2); **HRMS (ESI**^**+**^**)***m*/*z* calcd for C_28_H_27_O_8_FNa [M+Na]^+^ 533.1588, found 533.1586. **HPLC purity** 99.49%.

### Synthesis of (±)-Methyl (1*R*,2*R*,3*S*,3a*R*,8b*S*)-3a-(4-bromophenyl)-8-fluoro-1,8b-dihydroxy-6-(methoxymethoxy)-3-phenyl-2,3,3a,8b-tetrahydro-1*H*-cyclopenta[*b*]benzofuran-2-carboxylate (**9nb**)

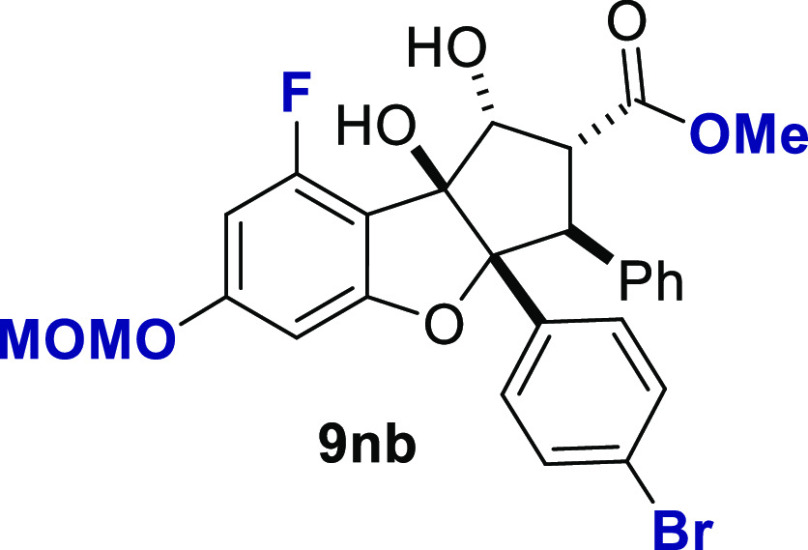



#### (*E*)-3-(4-Bromophenyl)-1-(2-fluoro-6-hydroxy-4-(methoxymethoxy)phenyl)prop-2-en-1-one (**12nb**)

A suspension of NaOEt (286 mg, 4.20 mmol, 3.00 equiv) in dry EtOH (4.7 mL) was cooled down to rt, followed by the addition of 1-(2-fluoro-6-hydroxy-4-(methoxymethoxy)phenyl)ethan-1-one (300 mg, 1.40 mmol, 1.00 equiv) at the same temperature. The suspension was stirred for 1 h, before 4-bromobenzaldehyde (256 mg, 1.40 mmol, 1.00 equiv) was added. The orange solution was stirred for 16 h at rt. The resulting orange suspension was poured into cold water and acidified to pH = 1 HCl (aq., 1 M). The precipitate was filtered, washed with water, dissolved in EtOAc, dried over MgSO_4_, filtered, concentrated and dried *in vacuo*. The crude product **12nb** (500 mg, 1.31 mmol, 93%) as a yellow-orange solid was used for next step without further purification. *R*_f_ = 0.73 (petroleum ether/EtOAc 1:1); ^**1**^**H NMR** (CDCl_3_, 400 MHz): δ [ppm] 13.58 (s, 1H, O*H*), 7.82 (dd, *J* = 15, 3.4 Hz, 1H, C(O)CH=C*H*), 7.61 (dd, *J* = 15, 1.7 Hz, 1H, C(O)C*H*=CH), 7.55 (dt, *J* = 8.6, 1.9 Hz, 2H, 2× Ar*H*), 7.49 (dt, 2H, *J* = 8.6, 1.9 Hz, 2× Ar*H*), 6.45 (q, 1H, *J* = 1.1 Hz, Ar*H*), 6.33 (dd, *J* = 14, 2.7 Hz, 1H, Ar*H*), 5.20 (s, 2H, *H*_3_CO), 3.49 (s, 3H, *H*_3_CO); ^**13**^**C NMR** (CDCl_3_, 100 MHz): δ [ppm] 190.9 (q, *d*, *J* = 4.9 Hz, *C*=O), 166.8 (q, *d*, *J* = 7.4 Hz, Ar*C*), 164.2 (q, *d*, *J* = 254 Hz, Ar*C*), 143.5 (t, *d*, *J* = 1.7 Hz, C(O)CH=*C*H), 133.7 (q, Ar*C*), 132.3 (t, 2× Ar*C*), 130.0 (t, 2× Ar*C*), 125.7 (t, *d*, *J* = 17 Hz, C(O)*C*H=CH), 125.1 (q, Ar*C*), 105.1 (q, *d*, *J* = 14 Hz, Ar*C*), 100.3 (t, *d*, *J* = 2.9 Hz, Ar*C*), 96.3 (t, *d*, *J* = 29 Hz, Ar*C*), 94.2 (s, *C*H_2_), 56.6 (p, H_3_CO); **HRMS (ESI**^**+**^**)***m*/*z* calcd for C_17_H_15_O_4_FBr [M+H]^+^ 381.0138, found 381.0128.

#### 2-(4-Bromophenyl)-5-fluoro-3-hydroxy-7-(methoxymetho-xy)-4*H*-chromen-4-one (**8nb**)

To suspension of chalcone **12nb** (500 mg, 1.31 mmol, 1.00 equiv) in MeOH (15.4 mL) and NaOH (aq., 3 M, 2.18 mL, 6.56 mmol, 5.00 equiv) was added H_2_O_2_ (aq., 30%, 304 μL, 6.44 mmol, 10.0 equiv) at 0 °C. The bright orange solution was stirred for 3 h at the same temperature. The resulting yellow suspension was stirred for further 16 h at rt. The resulting yellow suspension was poured into cold HCl (aq., 1 M) and extracted with CH_2_Cl_2_. The collected organic layers were washed with water, NaCl (sat., aq.), dried over MgSO_4_, filtered and concentrated *in vacuo*. The crude was recrystallized in MeOH to afford flavonol **8nb** as pale-yellow crystals (170 mg, 0.43 mmol, 33%). *R*_f_ = 0.60 (petroleum ether/EtOAc 1:1); ^**1**^**H NMR** (CDCl_3_, 400 MHz): δ [ppm] 8.10 (d, *J* = 8.8 Hz, 2H, 2× Ar*H*), 7.65 (d, *J* = 8.8 Hz, 2H, 2× Ar*H*), 7.01 (m, 1H, Ar*H*), 6.78 (dd, *J* = 12, 2.2 Hz, 1H, Ar*H*), 5.29 (s, 2H, C*H*_2_), 3.53 (s, 3H, *H*_3_CO); ^**13**^**C NMR** (CDCl_3_, 100 MHz): δ [ppm] 171.1 (q, *d*, *J* = 1.7 Hz, C = O), 161.7 (q, *d*, *J* = 14 Hz, Ar*C*), 161.2 (q, *d*, *J* = 263 Hz, Ar*C*), 157.3 (q, *d*, *J* = 6.55 Hz, Ar*C*), 142.3 (q, Ar*C*), 138.3 (q, COH), 131.9 (t, 2× Ar*C*), 129.6 (q, Ar*C*) 128.9 (t, 2× Ar*C*), 124.6 (q, *C*=COH), 106.4 (q, *d*, *J* = 13 Hz, Ar*C*), 102.2 (t, *d*, *J* = 23 Hz, Ar*C*), 99.4 (t, *d*, *J* = 3.9 Hz, Ar*C*), 94.8 (s, *C*H_2_), 56.5 (p, *C*H_3_O); **HRMS (ESI**^**+**^**)***m*/*z* calcd for C_17_H_13_O_5_BrF [M+H]^+^ 394.9930, found 394.9926.

#### (±)-Methyl (1*R*,2*R*,3*S*,3a*R*,8b*S*)-3a-(4-bromophenyl)-8-fluoro-1,8b-dihydroxy-6-(methoxymethoxy)-3-phenyl-2,3, 3a,8b-tetrahydro-1*H*-cyclopenta[*b*]benzofuran-2-carboxylate (**9nb**)

To a solution of flavonol **8nb** (170 mg, 0.43 mmol, 1.00 equiv) in dry 2,2,2-TFE (3.6 mL) and dry CHCl_3_ (8.6 mL) was added methyl cinnamate (991 mg, 6.11 mmol, 14.20 equiv). The clear solution was degassed with argon for 15 min, followed by UV-irradiation (100 W, 365 nm) at −5 °C for 10–16 h. After the flavonol was fully consumed, the solvent was removed *in vacuo* and the excess of methyl cinnamate was removed by silica gel purification (petroleum ether/EtOAc 4:1, then EtOAc). The cycloadduct mixture was used directly for the next step. To a solution of cycloadduct mixture (239 mg) in MeOH (14 mL) was added NaOMe solution (25 wt% in MeOH, 264 μL, 1.22 mmol, 2.84 equiv) and stirred under refluxing conditions for 1 h. The reaction was terminated by the addition of NH_4_Cl (sat., aq.). The aqueous layers were extracted with EtOAc. The collected organic layers were washed with water and NaCl (sat., aq.), dried over MgSO_4_, filtered and concentrated *in vacuo*. The yellow foam ketone crude product was directly used for the next step without further purification. A solution of Me_4_NBH(OAc)_3_ (569 mg, 2.17 mmol, 6.42 equiv) and freshly distilled AcOH (204 μL, 2.17 mmol, 10.4 equiv) in dry MeCN (8.7 mL) was prepared and stirred at rt for 10 min. To this solution was added ketone crude (188 mg, 0.34 mmol) in dry MeCN (5.6 mL). The reaction was carried out under light exclusion and stirred for 19 h at rt. The reaction was terminated by the addition of NaK-tartrate (sat., aq.) and a NH_4_Cl solution (sat, aq.). The layers were separated and the aqueous layers were extracted with CH_2_Cl_2_ (3 × 20 mL). The collected organic layers were washed with water and brine, dried over MgSO_4_, filtered and concentrated *in vacuo*. The crude product was purified by silica gel column chromatography (petroleum ether/EtOAc 3:1, then 2:1), followed by HPLC purification to yield **9nb** as a colorless foam (103 mg, 0.17 mmol, 18% over three steps). *R*_f_ = 0.33 (CH_2_Cl_2_/EtOAc 10:1); ^**1**^**H NMR** (DMSO-*d*_6_, 400 MHz): δ [ppm] 7.23 (d, *J* = 8.6 Hz, 2H, *H*-3′, *H*-5′), 7.08–7.02 (m, 4H, *H*-2″, H3″, *H*-5″, *H*-6″), 7.01–6.98 (m, 1H, *H*-4″), 6.92 (d, *J* = 7.3 Hz, 2H, *H*-2′, *H*-6′), 6.57 (d, *J* = 1.9 Hz, 1H, *H*-5), 6.39 (dd, *J* = 10.7, 1.9 Hz, 1H, *H*-7), 5.86 (d, *J* = 6.3 Hz, 1H, O*H*), 5.69 (s, 1H, O*H*), 5.22 (s, 2H, OC*H*_2_OCH_3_) 4.67 (t, *J* = 5.9 Hz, 1H, *H*-1), 4.21 (d, *J* = 14 Hz, 1H, *H*-3), 4.03 (dd, *J* = 14, 5.5 Hz, 1H, *H*-2), 3.55 (s, 3H, *H*_3_CO-11), 3.40 (s, 3H, OCH_2_OC*H*_3_); ^**13**^**C NMR** (DMSO-*d*_6_, 100 MHz): δ [ppm] 170.5 (q, *C*-11), 161.0 (q, *d*, *J* = 12 Hz, *C*-6), 160.6 (q, *d*, *J* = 291 Hz, *C*-8), 160.1 (q, *d*, *J* = 12 Hz, *C*-4a), 138.1 (q, *C*-1″), 136.0 (q, *C*-4′), 130.1 (t, *C*-2′, *C*-6′), 129.8 (t, *C*-3′, *C*-5′), 128.11 (t, *C*-2″, *C-*6″), 128.09 (t, *C*-3″, *C*-5″), 126.6 (t, *C*-4″), 120.4 (q, *C*-1′), 109.7 (q, *d*, *J* = 20 Hz, *C*-8a), 102.1 (q, *C*-3a), 97.3 (q, *d*, *J* = 24 Hz, *C*-7), 94.9 (t, *d*, *J* = 3.6 Hz, *C*-5) 94.5 (O*C*H_2_OCH_3_), 93.8 (t, *d*, *J* = 2.4 Hz, 2C, *C*-8b), 78.9 (t, *C*-1), 56.2 (p, OCH_2_O*C*H_3_), 55.3 (t, *C*-3), 51.9 (p, *C*H_3_O-11), 51.6 (t, *C*-2); **HRMS (ESI**^**+**^**)***m*/*z* calcd for C_27_H_24_O_7_BrFNa [M+Na]^+^ 581.0587, found: 581.0577; **HPLC purity** 99.23%.

### Synthesis of (±)-(1*R*,2*R*,3*S*,3a*R*,8b*S*)-3a-(4-chlorophenyl)-1,8b-dihydroxy-6,8-dimethoxy-*N,N*-dimethyl-3-phenyl-2,3,3a,8b-tetrahydro-1*H*-cyclopenta[*b*]benzofuran-2-carboxamide (**14aa**)

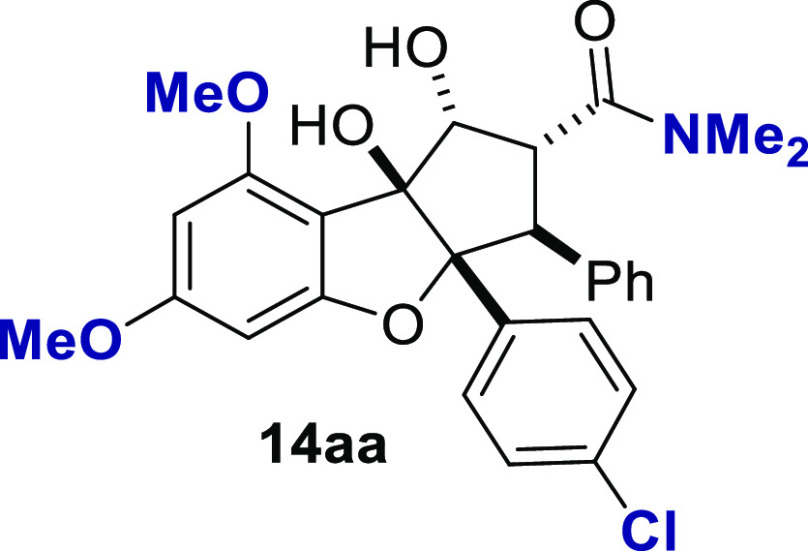



#### (±)-(1*R*,2*R*,3*S*,3a*R*,8b*S*)-3a-(4-Chlorophenyl)-1,8b-dihydroxy-6,8-dimethoxy-3-phenyl-2,3,3a,8b-tetrahydro-1*H*-cyclopenta[*b*]benzofuran-2-carboxylic acid (**13a**)

To a solution of **9a** (48 mg, 0.10 mmol, 1.00 equiv) in MeOH (1.5 mL) and H_2_O (0.25 mL) was added LiOH·H_2_O (21 mg, 0.49 mmol, 5.10 equiv). The reaction was stirred for 2 h at 50 °C and terminated by cooling down and acidified to pH = 1–2. The mixture was diluted with CH_2_Cl_2_ and water. The aqueous layers were extracted with CH_2_Cl_2_ and the collected organic layers were washed with NaCl (sat., aq.), dried over MgSO_4_, filtered and concentrated *in vacuo*. The crude product (42 mg) was used directly for the next step. *R*_f_ = 0.43 (8% MeOH in CH_2_Cl_2_).

#### (±)-(1*R*,2*R*,3*S*,3a*R*,8b*S*)-3a-(4-chlorophenyl)-1,8b-dihydroxy-6,8-dimethoxy-*N,N*-dimethyl-3-phenyl-2,3,3a,8b-tetrahydro-1*H*-cyclopenta[*b*]benzofuran-2-carboxamide (**14aa**)

To a mixture of crude **13a** (20 mg, 0.04 mmol, 1.00 equiv), EDC·HCl (12 mg, 0.06 mmol, 1.50 equiv), HOBt·H_2_O (8.5 mg, 0.05 mmol, 1.30 equiv) and HNMe_2_·HCl (17 mg, 0.21 mmol, 5.00 equiv) in dry CH_2_Cl_2_ (2.5 mL) was added freshly distilled Et_3_N (29 μL, 0.21 mmol, 5.00 equiv) dropwise at 0 °C and stirred at the same temperature for 10 min. The reaction was stirred at rt for 12 h. The reaction was terminated by the addition of HCl (aq., 1 M), followed by dilution with MeOH and CH_2_Cl_2_. The layers were separated, the aqueous layers were extracted with CH_2_Cl_2_ and the collected organic layers were washed with NaCl (sat., aq.), dried over MgSO_4_, filtered and concentrated *in vacuo*. The crude product was purified by silica gel column with 100% EtOAc to give **14aa** as a light-yellow foam (6.6 mg, 0.01 mmol, 31% over two steps). *R*_f_ = 0.66 (8% MeOH in CH_2_Cl_2_); ^**1**^**H NMR** (DMSO-*d*_6_, 400 MHz): δ [ppm] 7.14 (dt, *J* = 9.0, 2.2 Hz, 2H, *H*-3′, *H*-5′), 7.08 (dt, *J* = 8.9, 2.1 Hz, 2H, *H*-2″, *H*-6″), 7.04–7.01 (m, 2H, *H*-2′, *H*-6′), 6.98–6.94 (m, 1H, *H*-4″), 6.85 (d, *J* = 7.3 Hz, 2H, *H*-3″, *H*-5″), 6.31 (d, *J* = 1.9 Hz, 1H, *H*-5), 6.14 (d, *J* = 1.9 Hz, 1H, *H*-7), 5.20 (s, 1H, O*H*), 4.77 (dd, *J* = 6.1, 4.0 Hz, 1H, *H*-1), 4.65 (d, *J* = 4.0 Hz, 1H, O*H*), 4.31 (d, *J* = 13 Hz, 1H, *H*-3), 4.08 (dd, *J* = 13, 6.1 Hz, 1H, *H*-2), 3.78 (s, 3H, *H*_3_CO-8), 3.75 (s, 3H, *H*_3_CO-6), 3.26 (s, 3H, N(C*H*_3_)_2_), 2.75 (s, 3H, N(C*H*_3_)_2_); ^**13**^**C NMR** (DMSO-*d*_6_, 100 MHz): δ [ppm] 168.8 (q, *C*-11), 163.2 (q, *C*-6), 160.7 (q, *C*-4a), 158.2 (q, *C*-8), 139.4 (q, *C*-1′), 136.5 (q, *C*-1″), 131.2 (q, *C*-4′), 129.9 (t, *C-*3′, *C*-5′), 128.1 (t, *C*-2″, *C*-6″), 127.8 (t, *C*-2′, *C*-6′), 126.8 (t, *C*-3″, *C*-5″), 126.1 (t, *C*-4″), 108.9 (q, *C*-8b), 101.4 (q, *C*-3a), 94.2 (q, *C*-8a), 92.5 (t, *C*-7), 89.1 (t, *C*-5), 78.4 (t, *C*-1), 55.97 (t, *C*-3), 55.97 (*C*H_3_O-6/8), 55.8 (*C*H_3_O-6/8), 48.6 (t, C-2), 36.9 (p, N(*C*H_3_)_2_), 35.6 (p, N(*C*H_3_)_2_); **HRMS (ESI**^**+**^**)***m*/*z* calcd for C_28_H_28_ClNO_4_Na [M+Na]^+^ 532.1506, found 532.1503; **HPLC purity** 99.88%.

### Synthesis of (±)-(1*R*,2*R*,3*S*,3a*R*,8b*S*)-3a-(4-chlorophenyl)-1,8b-dihydroxy-*N*,6,8-trimethoxy-3-phenyl-2,3,3a,8b-tetrahydro-1*H*-cyclopenta[*b*]benzofuran-2-carboxamide (**14ab**)

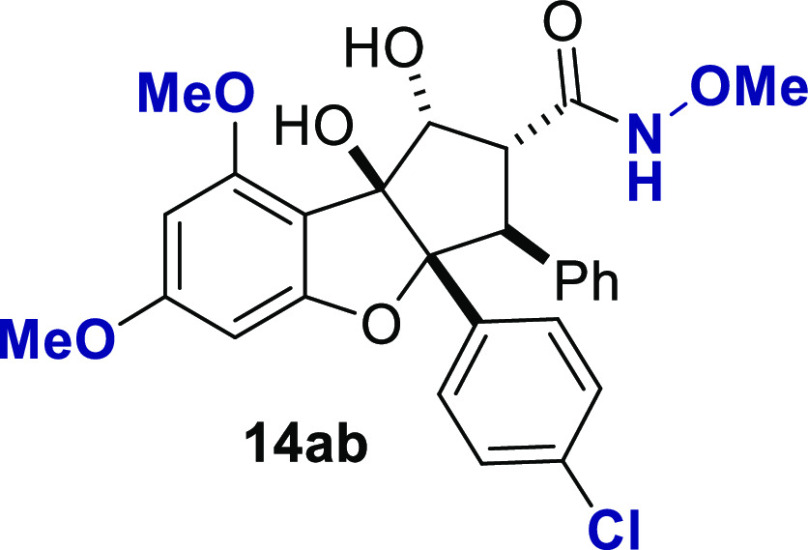



To a mixture of crude carboxylic acid **13a** (20 mg, 0.04 mmol, 1.00 equiv), EDC·HCl (12 mg, 0.06 mmol, 1.50 equiv), HOBt·H_2_O (8.5 mg, 0.05 mmol, 1.30 equiv) and H_2_NOMe·HCl (17 mg, 0.21 mmol, 5.00 equiv) in dry CH_2_Cl_2_ (2.5 mL) was added freshly distilled Et_3_N (29 μL, 0.21 mmol, 5.00 equiv) dropwise at 0 °C and stirred at the same temperature for 10 min. The reaction was stirred at rt for 12 h. The reaction was terminated by the addition of HCl (aq., 1 M), diluted with MeOH and CH_2_Cl_2_. The layers were separated, the aqueous layers were extracted with CH_2_Cl_2_ and the collected organic layers were washed with NaCl (sat., aq.), dried over MgSO_4_, filtered and concentrated *in vacuo*. The crude product was purified by silica gel column with 100% EtOAc to give **14ab** (7.2 mg, 0.01 mmol, 34% over two steps) as a light-yellow foam. *R*_f_ = 0.57 (8% MeOH in CH_2_Cl_2_); ^**1**^**H NMR** (DMSO-*d*_6_, 400 MHz): δ [ppm] 7.10–7.08 (m, 4H, *H*-2′, *H*-3′, *H*-5′, *H*-6′), 7.07–7.05 (m, 2H, *H*-2″, *H*-6″), 7.01–6.98 (m, 1H, *H*-4″), 6.94–6.92 (m, 2H, *H*-3″, *H*-5″), 6.29 (d, *J* = 1.9 Hz, 1H, *H*-5), 6.13 (d, *J* = 1.9 Hz, 1H, *H*-7), 5.20 (s, 1H, O*H*), 4.77 (d, *J* = 4.1 Hz, 1H, O*H*), 4.54 (t, *J* = 4.4 Hz, 1H, *H*-1), 4.29 (d, *J* = 14 Hz, 1H, *H*-3), 3.79 (s, 3H, *H*_3_CO-8), 3.74 (s, 3H, *H*_3_CO-6), 3.63 (dd, *J* = 14, 5.2 Hz, 1H, *H*-2), 3.51 (s, 3H, CONH(OC*H*_3_)); ^**13**^**C NMR** (DMSO-*d*_6_, 100 MHz): δ [ppm] 166.8 (q, *C*-11), 163.2 (q, *C-*6), 160.9 (q, *C*-4a), 158.3 (q, *C*-8), 138.5 (q, *C*-1″), 136.5 (q, *C*-1′), 131.4 (q, *C*-4′), 129.8 (t, *C*-3′, *C*-5′), 128.1 (*C*-3″, *C*-5″), 127.9 (t, *C*-2″, *C*-6″), 126.7 (t, *C*-2′, *C*-6′), 126.4 (C-4″), 108.4 (q, *C*-8a), 101.4 (q, *C*-3a), 94.2 (q, *C-*8b), 92.4 (t, *C*-7), 88.8 (t, *C*-5), 79.3 (t, *C*-1), 63.6 (p, CONH(O*C*H_3_)), 55.9 (p, *C*H_3_O-6), 55.8 (p, *C*H_3_O-8), 54.9 (t, *C*-3); **HRMS (ESI**^**+**^**)***m*/*z* calcd for C_28_H_28_ClNO_6_Na [M+Na]^+^ 532.1506, found 532.1503; **HPLC purity** 99.51%.

### Synthesis of (±)-(1*R*,2*R*,3*S*,3a*R*,8b*S*)-3a-(4-fluorophenyl)-1,8b-dihydroxy-6,8-dimethoxy-*N,N*-dimethyl-3-phenyl-2,3,3a,8b-tetrahydro-1*H*-cyclopenta[*b*]benzofuran-2-carboxamide (**14baa**)

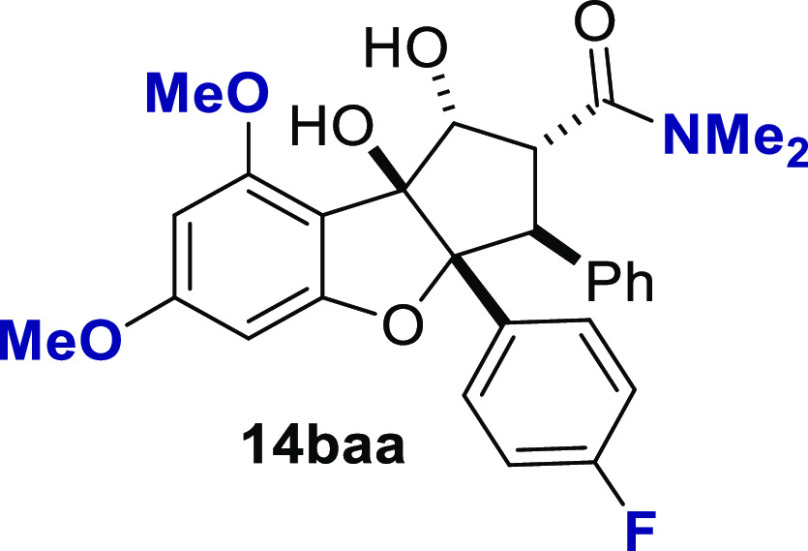



#### (1*R*,2*R*,3*S*,3a*R*,8b*S*)-3a-(4-Fluorophenyl)-1,8b-dihydroxy-6,8 -dimethoxy-3-phenyl-2,3,3a,8b-tetrahydro-1*H*-cyclopenta [*b*]benzofuran-2-carboxylic acid (**13ba**)

LiOH solution (aq. Two M, 212 μL, 0.42 mmol, 5.30 equiv) was added to **9ba** (35 mg, 0.08 mmol, 1.00 equiv) in MeOH (1.2 mL) and stirred at 50 °C for 6 h. The reaction was monitored by TLC, after the reaction was finished, the mixture was acidified to pH = 1–2 with HCl (aq., 1 M) and extracted with Et_2_O. The organic layers were washed with water and NaCl (sat., aq.), dried over MgSO_4_, filtered and concentrated *in vacuo*. The carboxylic acid crude **13ba** (34 mg) was used directly for the next step. *R*_f_ = 0.11 (EtOAc).

#### (±)-(1*R*,2*R*,3*S*,3a*R*,8b*S*)-3a-(4-fluorophenyl)-1,8b-dihydroxy-6,8-dimethoxy-*N,N*-dimethyl-3-phenyl-2,3,3a,8b-tetrahydro-1*H*-cyclopenta[*b*]benzofuran-2-carboxamide (**14baa**)

To a solution of **13ba** (17 mg, 0.037 mmol, 1.00 equiv) in dry CH_2_Cl_2_ (2.2 mL) were added HOBt·H_2_O (7.66 mg, 0.05 mmol, 1.30 equiv), EDC·HCl (10.8 mg, 0.06 mmol, 1.50 equiv) and HNMe_2_·HCl (15.3 mg, 0.19 mmol, 5.00 equiv) and cooled down to 0 °C for 5 min. Freshly distilled Et_3_N (33 μL, 0.19 mmol, 5.00 equiv) was added dropwise at 0 °C and stirred further at the same temperature for 10 min. The reaction mixture was warmed to rt and stirred for 16 h. After the reaction was finished, the mixture was concentrated *in vacuo* and purified by silica gel column chromatography (petroleum ether/EtOAc 2:1, then 1:1) to afford **14baa** as a colorless oil (14 mg, 0.028 mmol, 75% over two steps). *R*_f_ = 0.25 (EtOAc); ^**1**^**H NMR** (DMSO-*d*_6_, 400 MHz): δ [ppm] 7.15 (dd, *J* = 8.8, 5.6 Hz, 2H, *H*-2′, *H*-6′), 7.01 (t, *J* = 7.4 Hz, 2H, *H*-2″, *H*-6″), 7.01–6.96 (t, *J* = 7.2 Hz, 1H, *H*-4″), 6.84 (q, *J* = 9.0 Hz, 4H, *H*-3′, *H*-5′, *H-*3″, *H*-5″), 6.31 (d, *J* = 1.9 Hz, 1H, *H*-5), 6.14 (d, *J* = 1.8 Hz, 1H, *H*-7), 5.18 (s, 1H, O*H*), 4.78 (dd, *J* = 6.0, 4.1 Hz, 1H, *H*-1), 4.64 (d, *J* = 3.9 Hz, 1H, O*H*), 4.27 (d, *J* = 13 Hz, 1H, *H*-3), 4.05 (dd, *J* = 13, 6.2 Hz, 1H, *H*-2), 3.79 (s, 3H, *H*_3_CO-6), 3.75 (s, 3H, *H*_3_CO-8), 3.25 (s, 3H, N(C*H*_3_)_2_), 2.74 (s, 3H, -(NC*H*_3_)_2_); ^**13**^**C NMR** (DMSO-*d*_6_, 100 MHz): δ [ppm] 168.9 (q, *C*-11), 163.2 (q, *C*-5), 161.1 (q, *d*, *J* = 242 Hz, *C*-4′), 160.7 (q, *C*-4a), 158.1 (q, *C*-8), 139.4 (q, *C*-1″), 133.5 (q, *d*, *J* = 2.9 Hz, *C*-1′), 130.1 (t, *d*, *J* = 7.8 Hz, *C*-2′, *C*-6′), 128.1 (t, *C*-2″, *C*-6″), 127.8 (t, *C-*3″, *C*-5″), 126.1 (t, *C*-4″), 113.5 (d, *J* = 21 Hz, *C*-3′, *C*-5′), 109.0 (q, *C*-8a), 101.4 (q, *C*-3a), 94.0 (q, *C*-8b), 92.5 (t, *C*-7), 89.2 (t, *C*-5), 78.8 (t, *C*-1), 55.97 (p, *C*H_3_O-6), 55.95 (t, *C*-3), 55.93 (p, *C*H_3_O-8), 48.5 (t, *C*-2), 36.9 (p, CON(*C*H_3_)_2_), 35.6 (p, CON(*C*H_3_)_2_); **HRMS (ESI**^**+**^**)***m*/*z* calcd for C_28_H_29_NO_6_F [M+H]^+^ 494.1979, found 494.1978; **HPLC purity** 97.65%.

### Synthesis of (±)-(1*R*,2*R*,3*S*,3a*R*,8b*S*)-3a-(4-fluorophenyl)-1,8b-dihydroxy-*N*,6,8-trimethoxy-3-phenyl-2,3,3a,8b-tetrahydro-1*H*-cyclopenta[*b*]benzofuran-2-carboxamide (**14bab**)

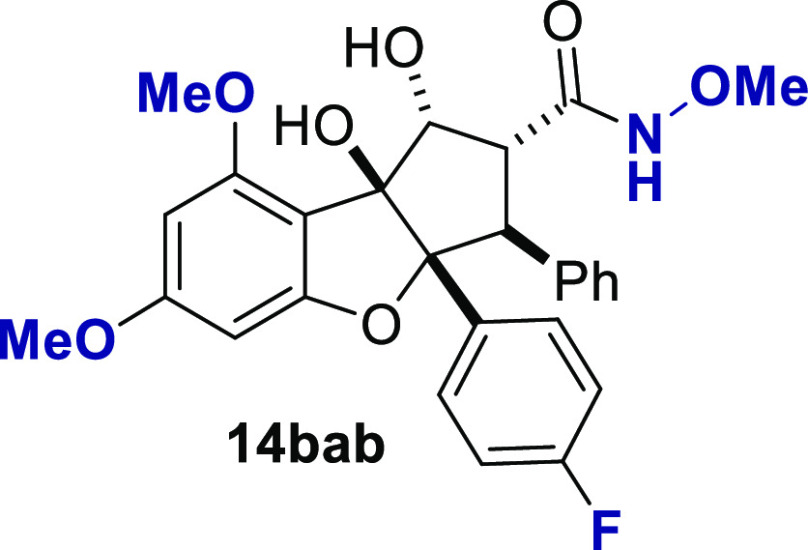



To a solution of **13ba** (18 mg, 0.037 mmol, 1.00 equiv) in dry CH_2_Cl_2_ (2.2 mL) were added HOBt·H_2_O (7.7 mg, 0.05 mmol, 1.30 equiv), EDC·HCl (11 mg, 0.06 mmol, 1.50 equiv) and H_2_NOMe·HCl (16 mg, 0.19 mmol, 5.00 equiv) and cooled down to 0 °C for 5 min. Freshly distilled Et_3_N (33 μL, 0.19 mmol, 5.00 equiv) was added dropwise at 0 °C and stirred further at the same temperature for 10 min. The reaction mixture was warmed to rt and stirred for 16 h. The mixture was then concentrated *in vacuo* and purified by silica gel column chromatography (petroleum ether/EtOAc 2:1 → 1:1) to afford **14bab** as a colorless oil (6.4 mg, 0.013 mmol, 34% over two steps). *R*_f_ = 0.21 (EtOAc); ^**1**^**H NMR** (DMSO-*d*_6_, 400 MHz): δ [ppm] 7.10 (dd, *J* = 9.0, 5.6 Hz, 2H, *H*-2′, *H*-6′), 7.04 (t, *J* = 7.4 Hz, 2H, *H*-2″, *H*-6″), 6.97 (t, *J* = 7.2 Hz, 1H, *H*-4″), 6.90–6.84 (m, 4H, *H*-3′, *H*-5′, *H*-3″, *H*-5″), 6.28 (d, *J* = 1.9 Hz, 1H, *H*-5), 6.10 (d, *J* = 1.9 Hz, 1H, *H*-7), 5.16 (s, 1H, O*H*), 4.73 (d, *J* = 4.2 Hz, 1H, O*H*), 4.54 (t, *J* = 4.6 Hz, 1H, *H*-1), 4.24 (d, *J* = 14 Hz, 1H, *H*-3), 3.59 (dd, *J* = 14, 5.3 Hz, 1H, *H*-2), 3.78 (s, 3H, *H*_3_CO-6), 3.73 (s, 3H, *H*_3_CO-8), 3.49 (s, 3H, NHOC*H*_3_); ^**13**^**C NMR** (DMSO-*d*_6_, 100 MHz): δ [ppm] 166.8 (q, *C*-11), 163.2 (q, *C-*6), 161.2 (q, *d*, *J* = 242 Hz, *C*-4′), 160.9 (q, *C*-4a), 158.3 (q, *C*-8), 138.6 (q, *C*-1″), 133.5 (q, *d*, *J* = 3.0 Hz, *C*-1′), 129.9 (t, *d*, *J* = 8.0 Hz, *C*-2′, *C*-6′), 128.1 (t, *C*-2″, *C* 6″), 127.9 (t, *C*-3″, *C-*5″), 126.4 (t, *C*-4″), 113.5 (t, *d*, *J* = 21 Hz, *C*-3′, *C*-5′), 108.6 (q, *C*-8a), 101.4 (q, *C*-3a), 94.0 (q, *C*-8b), 92.4 (t, *C*-7), 88.9 (t, *C*-5), 79.4 (t, *C*-1), 63.6 (CONH(O*C*H_3_)), 55.9 (t, C-3; p, *C*H_3_O-6), 55.8 (p, *C*H_3_-8), 48.7 (t, *C*-2); **HRMS (ESI**^**+**^**)***m*/*z* calcd for C_27_H_27_O_7_NF [M+H]^+^ 496.1772, found 496.1783; **HPLC purity** 99.43%.

### Synthesis of (±)-(1*R*,2*R*,3*S*,3a*R*,8b*S*)-1,8b-Dihydroxy-6,8-dimethoxy-3a-(4-methoxyphenyl)-*N,N*-dimethyl-3-phenyl-2,3,3a,8b-tetrahydro-1*H*-cyclopenta[*b*]benzofuran-2-carboxamide ((±)-Rocaglamide, *rac*-**1b**)

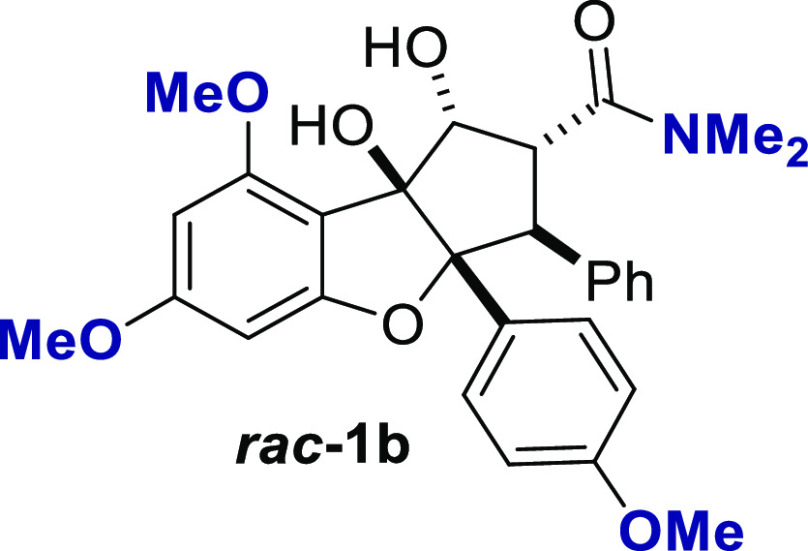



#### (±)-(1*R*,2*R*,3*S*,3a*R*,8b*S*)-1,8b-Dihydroxy-6,8-dimethoxy-3a-(4-methoxyphenyl)-3-phenyl-2,3,3a,8b-tetrahydro-1*H*-cyclopenta[*b*]benzofuran-2-carboxylic acid (**13bc**)

A solution of methyl ester **11bc** (54.0 mg, 110 μmol, 1.00 equiv) and lithium hydroxide (2.00 M in H_2_O, 280 μL, 559 μmol, 5.10 equiv) in MeOH (1.71 mL) was heated at 50 °C for 200 min. The solution was allowed to cool to rt, acidified with HCl (1.00 M in H_2_O) to pH = 1–2 and diluted with CH_2_Cl_2_ (5.00 mL) and H_2_O (5.00 mL). The organic layer was collected. The aqueous layer was extracted with CH_2_Cl_2_ (2×). The combined organic layers were dried over MgSO_4_, filtered and concentrated under reduced pressure to give the rocagloic acid (**13bc**) as a yellowish solid (52.0 mg, 109 μmol, 99%). *R*_f_ = 0.25 (EtOAc); ^**1**^**H NMR** (CDCl_3_, 400 MHz): δ [ppm] 7.05–6.93 (m, 5H, *H*-2′, *H*-6′, *H*-3″, *H*-4″, *H*-5″), 6.81 (d, *J* = 7.2 Hz, 2H, *H*-2″, *H*-6″), 6.61 (d, *J* = 9.0 Hz, 2H, *H*-3′, *H*-5′), 6.31 (d, *J* = 2.0 Hz, 1H, *H*-5), 6.14 (d, *J* = 2.0 Hz, 1H, *H*-7), 5.03 (s, 1H, O*H*-8b), 4.80 (dd, *J* = 6.5, 3.7 Hz, 1H, *H*-1), 4.58 (d, *J* = 3.6 Hz, 1H, O*H*-1), 4.21 (d, *J* = 13.5 Hz, 1H, *H*-3), 4.01 (dd, *J* = 13.4, 6.6 Hz, 1H, *H*-2), 3.79 (p, *H*_3_CO-8), 3.76 (p, *H*_3_CO-6), 3.61 (s, 3H, *H*_3_CO-4′), 3.23 (s, 3H, NC*H*_3_); ^**13**^**C NMR** (CDCl_3_, 100 MHz): δ [ppm] 174.8 (q, *C*-11), 164.2 (q, *C*-6), 161.0 (q, *C*-4a), 158.9 (q, *C*-4′), 157.1 (q, *C*-8), 136.9 (q, *C*-1″), 129.1 (t, C-2′, C-6′), 128.0 (t, *C*-3″, *C*-5″), 127.9 (t, C-2″, C-6″), 126.7 (t, C-4″), 126.5 (q. *C*-1′), 112.9 (t, *C*-3′, *C*-5′), 107.6 (q, *C*-8a), 102.0 (q, *C*-3a), 93.8 (q, *C*-8b), 92.8 (t, *C*-7), 89.6 (t, *C*-5), 79.5 (t, *C*-1), 55.9 (p, H_3_*C*O-8), 55.8 (p, H_3_*C*O-6), 55.2 (p, H_3_*C*O-4′), 55.1 (t, *C*-3), 50.4 (t, *C*-2). The analytical data are consistent with those reported in the literature.^46^

#### (±)-(1*R*,2*R*,3*S*,3a*R*,8b*S*)-1,8b-Dihydroxy-6,8-dimethoxy-3a-(4-methoxyphenyl)-*N,N*-dimethyl-3-phenyl-2,3,3a,8b-tetrahydro-1*H*-cyclopenta[*b*]benzofuran-2-carboxamide ((±)-Rocaglamide, **rac-1b**)

To a solution of rocagloic acid (**13bc**) (25.0 mg, 52.2 μmol, 1.00 equiv) in DMF (1.52 mL) was added dimethylamine hydrochloride (5.1 mg, 62.7 μmol, 1.20 equiv) and 4-DMAP (7.7 mg, 62.7 μmol, 1.20 equiv). After cooling the reaction mixture to 0 °C, EDC·HCl (12.0 mg, 62.7 μmol, 1.20 equiv) was added in portions over 5 min. After stirring for 30 min, triethylamine (8.7 μL, 62.7 μmol, 1.20 equiv) was added and the cooling bath was removed. When the starting material was fully consumed (13 h), HCl (1.00 M in H_2_O) was added and the mixture was extracted with CH_2_Cl_2_ (2×). The combined organic layers were washed with brine, dried over MgSO_4_, filtered and concentrated under reduced pressure. The crude product was purified by preparative TLC (CH_2_Cl_2_/MeOH 95:5) to afford (±)-rocaglamide (*rac***-1b)** as a colorless solid (2.4 mg, 4.75 μmol, 9%). *R*_f_ = 0.45 (CH_2_Cl_2_/MeOH 95:5); ^**1**^**H NMR** (DMSO-*d*_6_, 400 MHz): δ [ppm] 7.05–6.93 (m, 5H, *H*-2′, *H*-6′, *H*-3″, *H*-4″, *H*-5″), 6.81 (d, *J* = 7.2 Hz, 2H, *H*-2″, *H*-6″), 6.61 (d, *J* = 9.0 Hz, 2H, *H*-3′, *H*-5′), 6.31 (d, *J* = 2.0 Hz, 1H, *H*-5), 6.14 (d, *J* = 2.0 Hz, 1H, *H*-7), 5.03 (s, 1H, O*H*-8b), 4.80 (dd, *J* = 6.5, 3.7 Hz, 1H, *H*-1), 4.58 (d, *J* = 3.6 Hz, 1H, O*H*-1), 4.21 (d, *J* = 13.5 Hz, 1H, *H*-3), 4.01 (dd, *J* = 13.4, 6.6 Hz, 1H, *H*-2), 3.79 (p, *H*_3_CO-8), 3.76 (p, *H*_3_CO-6), 3.61 (s, 3H, *H*_3_*C*O-4′), 3.23 (s, 3H, NC*H*_3_), 2.74 (s, 3H, NC*H*_3_); ^**13**^**C NMR** (DMSO-*d*_6_, 100 MHz): δ [ppm] 168.5 (q, *C*-11), 162.7 (q, *C*-6), 160.3 (q, *C*-4a), 157.6 (q, *C*-8), 157.4 (q, *C*-4′), 139.2 (q, *C*-1″), 128.8 (t, C-2′, C-6′), 128.6 (q. *C*-1′), 127.7 (t, *C*-3″, *C*-5″), 127.2 (t, C-2″, C-6″), 125.5 (t, C-4″), 111.9 (t, *C*-3′, *C*-5′), 108.9 (q, *C*-8a), 101.1 (q, *C*-3a), 93.5 (q, *C*-8b), 91.9 (t, *C*-7), 88.8 (t, *C*-5), 78.2 (t, *C*-1), 55.5 (p, H_3_*C*O-8), 55.4 (p, H_3_*C*O-6), 55.3 (t, *C*-3), 54.7 (p, H_3_*C*O-4′), 47.8 (t, *C*-2), 36.4 (p, N*C*H_3_), 35.1 (p, N*C*H_3_); **HPLC purity** 95.65%. The analytical data are consistent with those reported in the literature.^47^

### Synthesis of (±)-(1*R*,2*R*,3*S*,3a*R*,8b*S*)-1,8b-Dihydroxy-*N*,6,8-trimethoxy-3a-(4-methoxyphenyl)-3-phenyl-2,3,3a,8b-tetrahydro-1*H*-cyclopenta[*b*]benzofuran-2-carboxamide ((±)-CR-31-B, *rac*-**1c**)

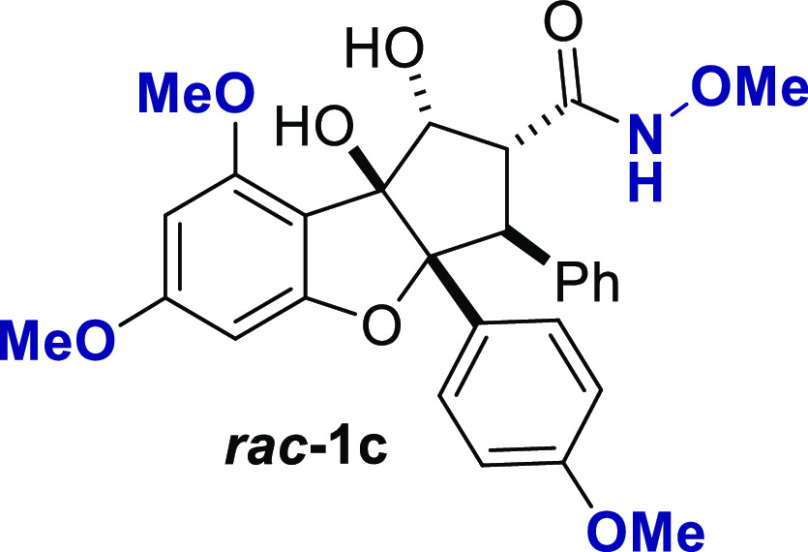



#### (±)-(1*R*,2*R*,3*S*,3a*R*,8b*S*)-1,8b-Dihydroxy-*N*,6,8-trimethoxy-3a-(4-methoxyphenyl)-3-phenyl-2,3,3a,8b-tetrahydro-1*H*-cyclopenta[*b*]benzofuran-2-carboxamide ((±)-CR-31-B, *rac*-**1c**)

To a solution of rocagloic acid (**13bc**) (25.0 mg, 52.2 μmol, 1.00 equiv) in CH_2_Cl_2_ (3.71 mL) EDC·HCl (15.0 mg, 78.4 μmol, 1.50 equiv), HOBt·H_2_O (10.7 mg, 67.9 μmol, 1.30 equiv), methoxylamine hydrochloride (21.8 mg, 261 μmol, 5.00 equiv) and triethylamine (36.2 μL, 261 μmol, 5.00 equiv) were added. The mixture was then stirred at rt for 12 h. Subsequently, the reaction was terminated by the addition of HCl (1.00 M in H_2_O), extracted with CH_2_Cl_2_ (3×), dried over MgSO_4_, filtered, concentrated and purified by flash chromatography (CH_2_Cl_2_/MeOH 95:5). (±)-CR-31-B (*rac***-1c**) was obtained as a colorless solid (11.8 mg, 23.2 μmol, 44%). *R*_f_ = 0.48 (CH_2_Cl_2_/MeOH 9:1); ^**1**^**H NMR** (DMSO-*d*_6_, 500 MHz): δ [ppm] 11.15 (s, 1H, N*H*), 7.06–6.96 (m, 5H, *H*-2′, *H*-6′, *H*-3″, *H*-4″, *H*-5″), 6.89 (d, *J* = 7.5 Hz, 2H, *H*-2″, *H*-6″), 6.60 (d, *J* = 8.8 Hz, 2H, *H*-3′, *H*-5′), 6.28 (d, *J* = 1.7 Hz, 1H, *H*-5), 6.12 (d, *J* = 1.7 Hz, 1H, *H*-7), 5.01 (s, 1H, O*H*-8b), 4.65 (d, *J* = 3.8 Hz, 1H, O*H*-1), 4.57- 4.55 (m, 1H, *H*-1), 4.18 (d, *J* = 14.1 Hz, 1H, *H*-3), 3.78 (p, H_3_*C*O-8), 3.74 (p, H_3_*C*O-6), 3.61 (s, 3H, C*H*_3_O-4′), 3.58 (dd, *J* = 14.2, 5.6 Hz, 1H, *H*-2), 3.49 (s, 3H, NHOC*H*_3_); ^**13**^**C NMR** (DMSO-*d*_6_, 125 MHz): δ [ppm] 166.4 (q, *C*-11), 162.7 (q, *C*-6), 160.5 (q, *C*-4a), 157.8 (q, *C*-8), 157.5 (q, *C*-4′), 138.3 (q, *C*-1″), 128.7 (t, C-2′, C-6′), 128.6 (q, *C*-1′), 127.8 (t, *C*-3″, *C*-5″), 127.3 (t, C-2″, C-6″), 125.8 (t, C-4″), 111.8 (t, *C*-3′, *C*-5′), 108.5 (q, *C*-8a), 101.1 (q, *C*-3a), 93.4 (q, *C*-8b), 91.8 (t, *C*-7), 88.5 (t, *C*-5), 79.0 (t, *C*-1), 63.1 (p, NHO*C*H_3_), 55.5 (p, H_3_*C*O-8), 55.4 (p, H_3_*C*O-6), 54.8 (p, H_3_*C*O-4′), 54.4 (t, *C*-3), 48.0 (t, *C*-2); **HRMS (ESI**^**+**^**)***m*/*z* calcd for C_28_H_29_NO_8_Na [M+Na]^+^ 530.1791, found 530.1792; **HPLC purity** 98.08%. The analytical data are consistent with those reported in the literature.^17^

### Synthesis of (±)-(1*R*,2*R*,3*S*,3a*R*,8b*S*)-6,8-Dichloro-1,8b-dihydroxy-*N*-methoxy-3a-(4-methoxyphenyl)-3-phenyl-2,3,3a,8b-tetrahydro-1*H*-cyclopenta[*b*]benzofuran-2-carboxamide (**14da**)

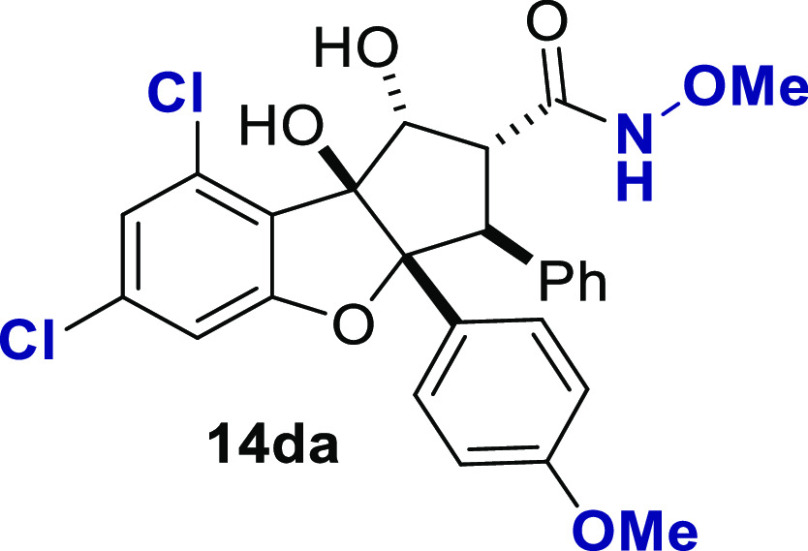



#### (±)-(1*R*,2*R*,3*S*,3a*R*,8b*S*)-6,8-Dichloro-1,8b-dihydroxy-3a-(4-methoxyphenyl)-3-phenyl-2,3,3a,8b-tetrahydro-1*H*-cyclopenta[*b*]benzofuran-2-carboxylic acid (**13da**)

A solution of methyl ester **9da** (40.0 mg, 79.8 μmol, 1.00 equiv) and lithium hydroxide solution (2.00 M in H_2_O, 203 μL, 407 μmol, 5.10 equiv) in MeOH (1.25 mL) was heated at 50 °C for 2 h. As only a low conversion could be detected by TLC, more lithium hydroxide solution (2.00 M in H_2_O, 203 μL, 407 μmol, 5.10 equiv) was added and the mixture was stirred for additional 18 h at 50 °C. The solution was then cooled, acidified with HCl (1.00 M in H_2_O) to pH = 1–2 and diluted with CH_2_Cl_2_ (5.00 mL) and H_2_O (5.00 mL). The organic layer was collected. The aqueous layer was extracted with CH_2_Cl_2_ (2×). The combined organic layers were dried over MgSO_4_, filtered and concentrated under reduced pressure to give the rocagloic acid **13da** as a yellowish solid (33.0 mg, 67.7 μmol, 85%). *R*_f_ = 0.52 (EtOAc); ^**1**^**H NMR** (DMSO-*d*_6_, 400 MHz): δ [ppm] 7.12 (d, *J* = 1.7 Hz, 1H, *H*-5), 7.07–6.89 (m, 8H, *H*-7, *H*-2′, H-6′, *H*-2″, *H*-3″, *H*-4″, *H*-5″, *H*-6″), 6.56 (d, *J* = 9.0 Hz, 2H, *H*-3′, *H*-5′), 5.59 (s, 1H, O*H*-8b), 4.63 (d, *J* = 4.2 Hz, 1H, *H*-1), 4.34 (d, *J* = 13.9 Hz, 1H, *H*-3), 3.85 (dd, *J* = 13.8, 3.9 Hz, 1H, *H*-2), 3.57 (s, 3H, *H*_3_CO-4′); ^**13**^**C NMR** (DMSO-*d*_6_, 100 MHz): δ [ppm] 172.5 (q, *C*-11), 160.8 (q, *C*-4a), 157.6 (q, *C*-4′), 138.5 (q, *C*-1″), 134.2 (q, *C*-6), 132.5 (q, *C*-8a), 128.5 (t, *C*-2′, *C*-6′), 128.4 (q, *C*-1′), 128.1 (t, *C*-3″, *C*-5″), 127.4 (t, *C*-2″, *C*-6″), 126.0 (q, *C*-8), 125.7 (t, *C*-4″), 120.8 (t, *C*-7), 111.9 (t, *C*-3′, *C*-5′), 109.1 (t, *C*-5), 102.7 (q, *C*-3a), 93.7 (q, *C*-8b), 78.1 (t, *C*-1), 55.7 (t, *C*-3), 54.8 (p, H_3_*C*O-4′), 51.9 (t, *C*-2); **HRMS (ESI**^**–**^**)***m*/*z* calcd for C_25_H_19_Cl_2_O_6_ [M-H]^−^ 485.0559, found 485.0575.

#### (±)-(1*R*,2*R*,3*S*,3a*R*,8b*S*)-6,8-Dichloro-1,8b-dihydroxy-*N*-methoxy-3a-(4-methoxyphenyl)-3-phenyl-2,3,3a,8b-tetrahydro-1*H*-cyclopenta[*b*]benzofuran-2-carboxamide (**14da**)

To a solution of rocagloic acid **13da** (17.6 mg, 36.1 μmol, 1.00 equiv) in CH_2_Cl_2_ (2.58 mL) EDC·HCl (10.4 mg, 54.2 μmol, 1.50 equiv), HOBt·H_2_O (7.7 mg, 48.4 μmol, 1.35 equiv), methoxylamine hydrochloride (15.1 mg, 181 μmol, 5.00 equiv) and triethylamine (25.0 μL, 181 μmol, 5.00 equiv) were added. The mixture was stirred at rt for 12 h. Subsequently, the reaction was terminated by the addition of HCl (1.00 M in H_2_O), extracted with CH_2_Cl_2_ (3×). The combined organic layers were dried over MgSO_4_, filtered and concentrated under reduced pressure. The crude product was purified by flash chromatography (CH_2_Cl_2_/MeOH 100:0 → 95:5). The desired rocagloic amide **14da** was obtained as a colorless solid (5.7 mg, 11.0 μmol, 31%). *R*_f_ = 0.48 (CH_2_Cl_2_/MeOH 95:5); ^**1**^**H NMR** (DMSO-*d*_6_, 400 MHz): δ [ppm] 11.27 (s, 1H, N*H*OCH_3_), 7.14 (d, *J* = 1.5 Hz, 1H, *H*-5), 7.07–6.95 (m, 8H, *H*-7, *H*-2′, H-6′, *H*-2″, *H*-3″, *H*-4″, *H*-5″, *H*-6″), 6.59 (d, *J* = 9.1 Hz, 2H, *H*-3′, *H*-5′), 5.60 (s, 1H, O*H*-8b), 5.34 (d, *J* = 5.4 Hz, 1H, O*H*-1), 4.55 (t, *J* = 4.7 Hz, 1H, *H*-1), 4.40 (d, *J* = 14.1 Hz, 1H, *H*-3), 3.67 (dd, *J* = 14.1, 4.2 Hz, 1H, *H*-2), 3.59 (s, 3H, *H*_3_CO-4′), 3.52 (s, 3H, NHOC*H*_3_); ^**13**^**C NMR** (DMSO-*d*_6_, 100 MHz): δ [ppm] 166.3 (q, *C*-11), 160.7 (q, *C*-4a), 157.7 (q, *C*-4′), 137.9 (q, *C*-1″), 134.2 (q, *C*-6), 132.6 (q, *C*-8a), 128.4 (t, *C*-2′, *C*-6′), 128.1 (q, *C*-1′), 127.9 (t, *C*-3″, *C*-5″), 127.4 (t, *C*-2″, *C*-6″), 126.9 (t, *C*-4″), 125.8 (q, *C*-8), 120.8 (t, *C*-7), 111.9 (t, *C*-3′, *C*-5′), 109.1 (t, *C*-5), 102.0 (q, *C*-3a), 93.8 (q, *C*-8b), 78.4 (t, *C*-1), 63.2 (p, NHO*C*H_3_), 54.9 (t, *C*-3), 54.8 (p, H_3_*C*O-4′), 48.9 (t, *C*-2); **HRMS (ESI**^**+**^**)***m*/*z* calcd for C_26_H_23_Cl_2_NO_6_Na [M+Na]^+^ 538.0800, found 538.0794; **HPLC purity** 95.70%.

### Synthesis of (±)-(1*R*,2*R*,3*S*,3a*R*,8b*S*)-6-Bromo-8-chloro-1,8b-dihydroxy-*N*-methoxy-3a-(4-methoxyphenyl)-3-phenyl-2,3,3a,8b-tetrahydro-1*H*-cyclopenta[*b*]benzofuran-2-carboxamide (**14f**)

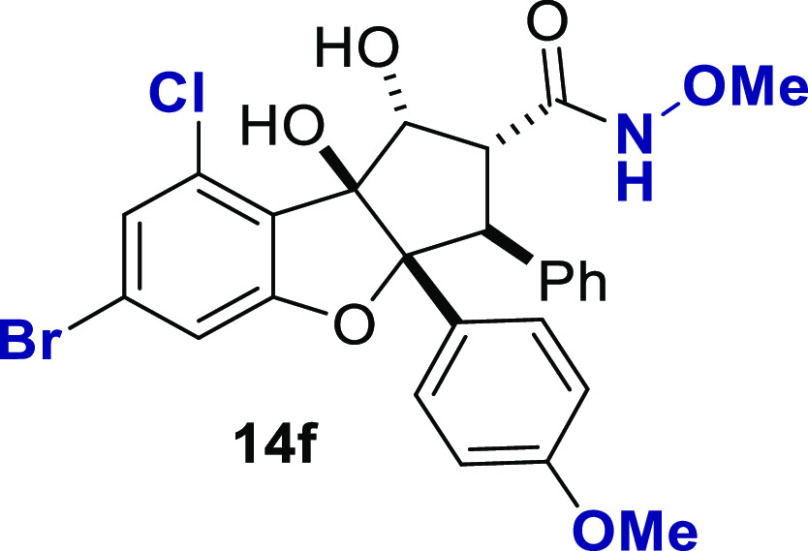



#### (±)-(1*R*,2*R*,3*S*,3a*R*,8b*S*)-6-Bromo-8-chloro-1,8b-dihydroxy-3a-(4-methoxyphenyl)-3-phenyl-2,3,3a,8b-tetrahydro-1*H*-cyclopenta[*b*]benzofuran-2-carboxylic acid (**13f**)

A solution of methyl ester **9f** (68.2 mg, 125 μmol, 1.00 equiv) and lithium hydroxide solution (2.00 M in H_2_O, 319 μL, 637 μmol, 5.10 equiv) in MeOH (10.1 mL) was heated at 50 °C for 28 h. Then, the solution was cooled, acidified with HCl (1.00 M in H_2_O) to pH = 1–2 and diluted with CH_2_Cl_2_ (10.0 mL) and H_2_O (10.0 mL). The organic layer was collected. The aqueous layer was extracted with CH_2_Cl_2_ (2×). The combined organic layers were dried over MgSO_4_, filtered and concentrated under reduced pressure to give the rocagloic acid **13f** as a yellowish solid (59.6 mg, 112 μmol, 90%). *R*_f_ = 0.56 (EtOAc); ^**1**^**H NMR** (DMSO-*d*_6_, 400 MHz): δ [ppm] 12.20 (bs, 1H, CO_2_*H*), 7.25 (d, *J* = 1.6 Hz, 1H, *H*-5), 7.12 (d, *J* = 1.6 Hz, 1H, *H*-7), 7.07–6.94 (m, 7H, *H*-2′, *H*-6′, *H*-2″, *H*-3″, *H*-4″, *H*-5″, *H*-6″), 6.56 (d, *J* = 9.0 Hz, 2H, *H*-3′, *H*-5′), 5.60 (s, 1H, *H*O-8b), 4.65 (d, *J* = 4.3 Hz, 1H, *H*-1), 4.34 (d, *J* = 13.9 Hz, 1H, *H*-3), 3.89 (dd, *J* = 13.9, 4.3 Hz, 1H, *H*-2), 3.58 (s, 3H, *H*_3_CO-4′); ^**13**^**C NMR** (DMSO-*d*_6_, 100 MHz): δ [ppm] 171.8 (q, *C*-11), 160.9 (q, *C*-4a), 157.6 (q, *C*-4′), 138.5 (q, *C*-1″), 132.7 (q, *C*-8a), 128.5 (t, *C*-2′, *C*-6′), 128.3 (q, C-1′), 128.0 (t, *C*-3″, *C*-5″), 127.4 (t, *C*-2″, *C*-6″), 126.3 (q, *C*-8), 125.7 (t, *C*-4″), 123.4 (t, *C*-7), 122.0 (q, *C*-6), 111.8 (t, *C*-5, *C*-3′, *C*-5′), 102.5 (q, *C*-3a), 93.7 (q, *C*-8b), 78.1 (t, *C*-1), 55.3 (t, *C*-3), 54.7 (p, H_3_*C*O-4′), 51.8 (t, *C*-2). **HRMS (ESI**^**–**^**)***m*/*z* calcd for C_25_H_19_ClBrO_6_ [M-H]^−^ 529.0054, found 529.0057.

#### (±)-(1*R*,2*R*,3*S*,3a*R*,8b*S*)-6-Bromo-8-chloro-1,8b-dihydroxy-*N*-methoxy-3a-(4-methoxyphenyl)-3-phenyl-2,3,3a,8b-tetrahydro-1*H*-cyclopenta[*b*]benzofuran-2-carboxamide (**14f**)

To a solution of rocagloic acid **13f** (70.0 mg, 132 μmol, 1.00 equiv) in CH_2_Cl_2_ (9.08 mL) EDC·HCl (37.9 mg, 197 μmol, 1.50 equiv), HOBt·H_2_O (31.6 mg, 178 μmol, 1.35 equiv) and triethylamine (91.7 μL, 658 μmol, 5.00 equiv) were added and was stirred at rt. After 1 h, methoxylamine hydrochloride (55.0 mg, 658 μmol, 5.00 equiv) was added and reaction mixture was stirred for additional 18 h. The reaction was terminated by addition of HCl (1.00 M in H_2_O), extracted with CH_2_Cl_2_ (3×). The combined organic layers were dried over MgSO_4_, filtered and concentrated under reduced pressure. The crude product was purified by flash chromatography (CH_2_Cl_2_/MeOH 98:2). The desired rocagloic amide **14f** was obtained as a colorless solid (58.0 mg, 103 μmol, 79%). *R*_f_ = 0.32 (CH_2_Cl_2_/MeOH 95:5); ^**1**^**H NMR** (DMSO-*d*_6_, 400 MHz): δ [ppm] 11.28 (s, 1H, N*H*OCH_3_), 7.26 (d, *J* = 1.6 Hz, 1H, *H*-5), 7.13 (d, *J* = 1.6 Hz, 1H, *H*-7), 7.07–6.95 (m, 7H, *H*-2′, *H*-6′, *H*-2″, *H*-3″, *H*-4″, *H*-5″, *H*-6″), 6.59 (d, *J* = 9.0 Hz, 2H, *H*-3′, *H*-5′), 5.60 (s, 1H, *H*O-8b), 5.34 (d, *J* = 5.4 Hz, 1H, *H*O-1), 4.55 (d, *J* = 4.8 Hz, 1H, *H*-1), 4.30 (d, *J* = 14.1 Hz, 1H, *H*-3), 3.68 (dd, *J* = 14.1, 4.2 Hz, 1H, *H*-2), 3.59 (s, 3H, *H*_3_CO-4′), 3.52 (s, 3H, NHOC*H*_3_); ^**13**^**C NMR** (DMSO-*d*_6_, 100 MHz): δ [ppm] 166.3 (q, *C*-11), 160.8 (q, *C*-4a), 157.7 (q, *C*-4′), 137.9 (q, *C*-1″), 132.9 (q, *C*-8a), 128.5 (t, *C*-2′, *C*-6′), 128.1 (q, C-1′), 127.9 (t, *C*-3″, *C*-5″), 127.4 (t, *C*-2″, *C*-6″), 126.2 (q, *C*-8), 125.9 (t, *C*-4″), 123.5 (q, *C*-7), 122.1 (q, *C*-6), 112.0 (t, *C*-5), 111.9 (t, *C*-3′, *C*-5′), 101.9 (q, *C*-3a), 93.9 (q, *C*-8b), 78.4 (t, *C*-1), 63.2 (p, NHO*C*H_3_), 54.9 (t, *C*-3), 54.8 (p, H_3_*C*O-4′), 48.9 (t, *C*-2); **HRMS (ESI**^**+**^**)***m*/*z* calcd for C_26_H_23_NO_6_ClBrNa [M+Na]^+^ 582.0295, found 582.0272; **HPLC purity** ∼100.00%.

### Synthesis of (±)-(1*R*,2*R*,3*S*,3a*R*,8b*S*)-8-Bromo-6-chloro-1,8b-dihydroxy-*N*-methoxy-3a-(4-methoxyphenyl)-3-phenyl-2,3,3a,8b-tetrahydro-1*H*-cyclopenta[*b*]benzofuran-2-carboxamide (**14g**)

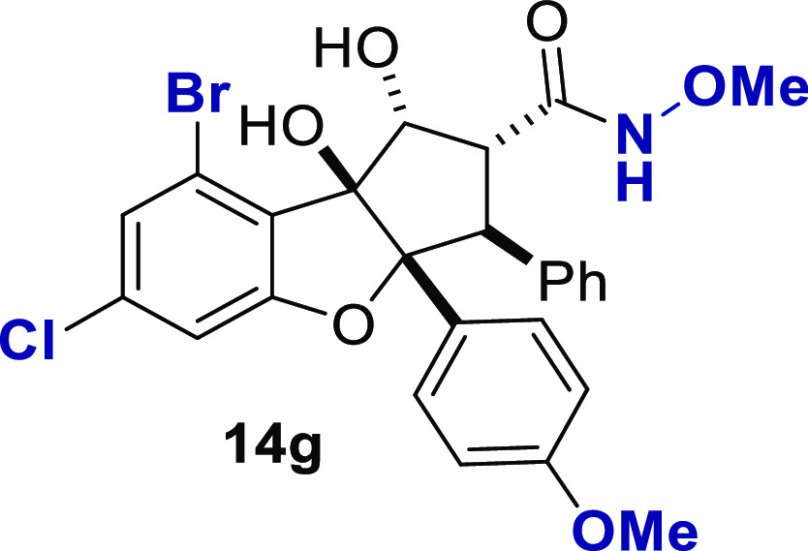



#### (±)-(1*R*,2*R*,3*S*,3a*R*,8b*S*)-8-Bromo-6-chloro-1,8b-dihydroxy-3a-(4-methoxyphenyl)-3-phenyl-2,3,3a,8b-tetrahydro-1*H*-cyclopenta[*b*]benzofuran-2-carboxylic acid (**13g**)

A solution of methyl ester **9g** (34.4 mg, 63.0 μmol, 1.00 equiv) and lithium hydroxide solution (2.00 M in H_2_O, 327 μL, 653 μmol, 10.4 equiv) in MeOH (5.08 mL) was heated at 50 °C for 21 h. Subsequently, the solution was allowed to cool to rt, acidified with HCl (1.00 M in H_2_O) to pH = 1–2 and diluted with CH_2_Cl_2_ (10.0 mL) and H_2_O (10.0 mL). The organic layer was collected. The aqueous layer was extracted with CH_2_Cl_2_ (2× 10.0 mL). The combined organic layers were dried over MgSO_4_, filtered and concentrated under reduced pressure to give the rocagloic acid **13g** as a yellowish solid (29.5 mg, 55.5 μmol, 88%). *R*_f_ = 0.56 (EtOAc); ^**1**^**H NMR** (DMSO-*d*_6_, 600 MHz): δ [ppm] 12.04 (bs, 1H, CO_2_*H*), 7.17 (d, *J* = 1.6 Hz, 1H, *H*-5), 7.14 (d, *J* = 1.6 Hz, 1H, *H*-7), 7.07–6.94 (m, 7H, *H*-2′, *H*-6′, *H*-2″, *H*-3″, *H*-4″, *H*-5″, *H*-6″), 6.55 (d, *J* = 8.9 Hz, 2H, *H*-3′, *H*-5′), 5.58 (s, 1H, *H*O-8b), 4.67 (d, *J* = 3.7 Hz, 1H, *H*-1), 4.39 (d, *J* = 14.1 Hz, 1H, *H*-3), 3.92 (dd, *J* = 14.1, 3.6 Hz, 1H, *H*-2), 3.57 (s, 3H, *H*_3_CO-4′); ^**13**^**C NMR** (DMSO-*d*_6_, 150 MHz): δ [ppm] 172.5 (q, *C*-11), 160.9 (q, *C*-4a), 157.5 (q, *C*-4′), 138.6 (q, *C*-1″), 134.2 (q, *C*-6), 128.5 (t, *C*-2′, *C*-6′), 128.2 (q, C-1′), 128.1 (t, *C*-3″, *C*-5″), 127.34 (t, *C*-2″, *C*-6″), 127.28 (q, *C*-8a), 125.6 (t, *C*-4″), 123.5 (t, *C*-7), 120.7 (q, *C*-8), 111.8 (t, *C*-3′, *C*-5′), 109.4 (t, *C*-5), 102.8 (q, *C*-3a), 94.1 (q, *C*-8b), 77.9 (t, *C*-1), 55.0 (t, *C*-3), 54.7 (p, H_3_*C*O-4′), 51.9 (t, *C*-2); **HRMS (ESI**^**–**^**)***m*/*z* calcd for C_25_H_19_ClBrO_6_ [M-H]^−^ 529.0054, found 529.0065.

#### (±)-(1*R*,2*R*,3*S*,3a*R*,8b*S*)-8-Bromo-6-chloro-1,8b-dihydroxy-*N*-methoxy-3a-(4-methoxyphenyl)-3-phenyl-2,3,3a,8b-tetrahydro-1*H*-cyclopenta[*b*]benzofuran-2-carboxamide (**14g**)

To a solution of rocagloic acid **13g** (16.5 mg, 31.0 μmol, 1.00 equiv) in CH_2_Cl_2_ (2.14 mL) EDC·HCl (8.9 mg, 46.5 μmol, 1.50 equiv), HOBt·H_2_O (7.5 mg, 41.9 μmol, 1.35 equiv) and triethylamine (21.6 μL, 155 μmol, 5.00 equiv) were added and was stirred at rt. After 1 h, methoxylamine hydrochloride (13.0 mg, 155 μmol, 5.00 equiv) was added and the reaction mixture was stirred for additional 18 h. The reaction was terminated by addition of HCl (1.00 M in H_2_O), extracted with CH_2_Cl_2_ (3×). The combined organic layers were dried over MgSO_4_, filtered and concentrated under reduced pressure. The crude product was purified by flash chromatography (CH_2_Cl_2_/MeOH 100:0 → 95:5). The desired rocagloic amide **14g** was obtained as a colorless solid (4.0 mg, 7.1 μmol, 23%). *R*_f_ = 0.30 (CH_2_Cl_2_/MeOH 95:5); ^**1**^**H NMR** (DMSO-*d*_6_, 400 MHz): δ [ppm] 11.30 (s, 1H, N*H*OCH_3_), 7.17 (d, *J* = 1.7 Hz, 1H, *H*-5), 7.15 (d, *J* = 1.7 Hz, 1H, *H*-7), 7.07–7.04 (m, 2H, *H*-2″, *H*-6″), 7.00–6.95 (m, 5H, *H*-2′, *H*-6′, *H*-3″, *H*-4″, *H*-5″), 6.58 (d, *J* = 9.0 Hz, 2H, *H*-3′, *H*-5′), 5.56 (s, 1H, O*H*-8b), 5.28 (d, *J* = 5.3 Hz, 1H, O*H*-1), 4.55 (t, *J* = 4.6 Hz, 1H, *H*-1), 4.44 (d, *J* = 14.1 Hz, 1H, *H*-3), 3.68 (dd, *J* = 14.1, 4.0 Hz, 1H, *H*-2), 3.58 (s, 3H, s, 3H, *H*_3_CO-4′), 3.53 (s, 3H, NHOC*H*_3_); ^**13**^**C NMR** (DMSO-*d*_6_, 100 MHz): δ [ppm] 166.4 (q, *C*-11), 160.8 (q, *C*-4a), 157.6 (q, *C*-4′), 138.0 (q, *C*-1″), 134.3 (q, *C*-6), 128.4 (t, *C*-2′, *C*-6′), 128.2 (q, C-1′), 127.9 (t, *C*-3″, *C*-5″), 127.43 (q, *C*-8a), 127.42 (t, *C*-2″, *C*-6″),125.9 (t, *C* 4″), 123.6 (t, *C*-7), 120.9 (q, *C*-8), 111.9 (t, *C*-3′, *C*-5′), 109.5 (t, *C*-5), 102.1 (q, *C*-3a), 94.2 (q, *C*-8b), 78.2 (t, *C*-1), 63.2 (p, NHO*C*H_3_), 54.9 (t, *C*-3), 54.8 (p, H_3_*C*O-4′), 48.9 (t, *C*-2); **HRMS (ESI**^**+**^**)***m*/*z* calcd for C_26_H_23_NO_6_ClBrNa [M+Na]^+^ 582.0295, found 582.0307; **HPLC purity** 98.49%.

### Synthesis of (±)-(1*R*,2*R*,3*S*,3a*R*,8b*S*)-8-Fluoro-3a-(4-fluorophenyl)-1,8b-dihydroxy-6-methoxy-*N,N*-dimethyl-3-phenyl-2,3,3a,8b-tetrahydro-1*H*-cyclopenta[*b*]benzofuran-2-carboxamide (**14ha**)

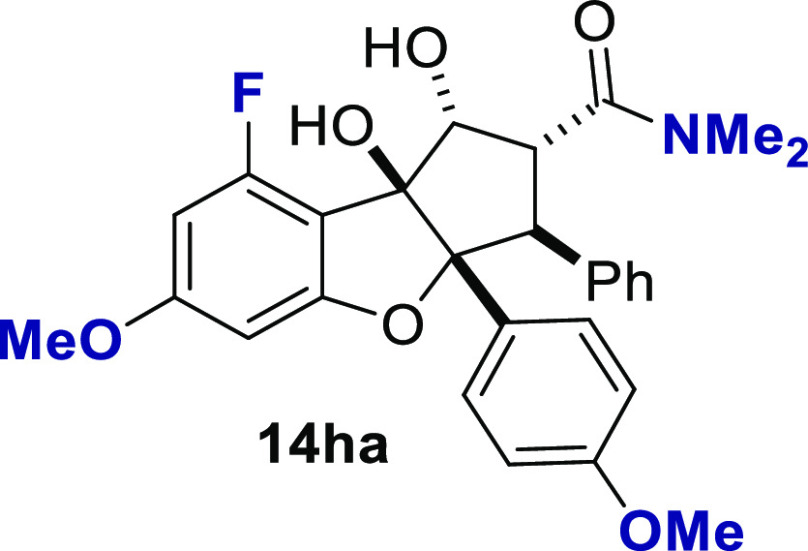



#### 8-Fluoro-1,8b-dihydroxy-6-methoxy-3a-(4-methoxyphenyl)-3-phenyl-2,3,3a,8b-tetrahydro-1*H*-cyclopenta[*b*]benzofuran-2-carboxylic acid (**13h**)

LiOH (aq., 2 M, 0.19 mL, 0.37 mmol, 5.10 equiv) was added to **9h** (35 mg, 0.07 mmol, 1.00 equiv) in MeOH (1.2 mL) and stirred for 2.5 h at 50 °C. After the ester was fully consumed, the mixture was acidified with HCl (aq., 1 M) and extracted with Et_2_O. The organic layers were washed with water and NaCl (sat., aq.), dried over MgSO_4_ and concentrated *in vacuo*. The carbo-xylic acid crude **13h** (31 mg) was used directly for the next step without further purification. *R*_f_ = 0.47 (EtOAc).

#### (±)-(1*R*,2*R*,3*S*,3a*R*,8b*S*)-8-Fluoro-3a-(4-fluorophenyl)-1,8b-dihydroxy-6-methoxy-*N,N*-dimethyl-3-phenyl-2,3,3a,8b-tetrahydro-1*H*-cyclopenta[*b*]benzofuran-2-carboxamide (**14ha**)

To carboxylic acid **13h** (10 mg, 0.02 mmol, 1.00 equiv) in dry CH_2_Cl_2_ (1.3 mL) were added HOBt·H_2_O (4.4 mg, 0.03 mmol, 1.30 equiv), EDC·HCl (6.2 mg, 0.03 mmol, 1.50 equiv) and HNMe_2_·HCl (8.8 mg, 0.11 mmol, 5.00 equiv) and cooled down to 0 °C for 5 min. Et_3_N (15 μL, 0.11 mmol, 5.00 equiv) was added dropwise at 0 °C and stirred further at the same temperature for 10 min. The reaction mixture was warmed up to rt and stirred for 16 h. After the starting material was fully consumed, the mixture was concentrated *in vacuo* and purified by silica gel column chromatography (5% MeOH in CH_2_Cl_2_) to afford **14ha** (3.8 mg, 7.7 μmol, 33% over two steps) as a colorless oil. *R*_f_ = 0.47 (EtOAc); ^**1**^**H NMR** (DMSO-*d*_6_, 400 MHz): δ [ppm] 7.06 (dt, *J* = 10, 2.5 Hz, 2H, *H*-2′ and *H*-6′), 7.02–7.01 (m, 2H, *H*-2″, *H*-6″), 6.97–6.94 (m, 1H, *H*-4″), 6.83–6.82 (m, 2H, *H*-3″, *H-*5″), 6.63 (dt, *J* = 9.9, 2.5 Hz, 2H, *H*-3′, *H*-5′), 6.50 (d, *J* = 2.0 Hz, 1H, *H*-5), 6.29 (dd, *J* = 11, 2.9 Hz, 1H, *H*-7), 5.40 (s, 1H, O*H*), 5.36 (d, *J* = 6.3 Hz, 1H, O*H*), 4.76 (t, *J* = 6.4, 1H, *H-*1), 4.19 (d, *J* = 14 Hz, 1H, *H*-3), 4.04 (dd, *J* = 14, 6.5 Hz, 1H, *H*-2), 3.78 (s, 3H, H_3_CO-6), 3.62 (s, 3H, H_3_CO-4′), 3.23 (s, 3H, -N(C*H*_3_)_2_), 2.74 (s, 3H, -N(C*H*_3_)_2_); ^**13**^**C NMR** (DMSO-*d*_6_, 100 MHz): δ [ppm] 168.9 (q, *C*-11), 162.8 (q, *d*, *J* = 13 Hz, *C*-6), 161.2 (q, *d*, *J* = 12 Hz, *C*-4a), 160.8 (q, *d*, *J* = 249 Hz, *C*-8), 158.0 (q, *C*-4a), 154.1 (q, *C*-4′), 139.4 (q, *C*-1″), 129.2 (t, *C*-2′, *C*-6′), 128.7 (q, *C*-1′), 128.2 (t, *C*-3″, *C*-5″), 127.2 (t, *C*-2″, *C*-6″), 126.1 (t, *C*-4″), 112.5 (t, *C-*3′, *C*-5′), 109.5 (q, *d*, *J* = 20 Hz, *C*-8a), 101.9 (q, *C*-3a), 95.4 (t, *d*, *J* = 25 Hz, *C*-7), 93.9 (t, *d*, *J* = 2.5 Hz, *C*-5), 92.7 (q, *d*, *J* = 2.9 Hz, *C*-8b), 77.7 (t, *C*-1), 56.3 (p, *C*H_3_O-6), 55.9 (t, C-3), 55.2 (p, *C*H_3_O-4′), 48.6 (t, *C*-2), 36.9 (p, CON(*C*H_3_)_2_), 35.6 (p, CON(*C*H_3_)_2_); **HRMS (ESI**^**+**^**)***m*/*z* calcd for C_28_H_28_FNO_6_Na [M+Na]^+^ 516.1798, found 516.1786; **HPLC purity** 98.44%.

### Synthesis of (±)-(1*R*,2*R*,3*S*,3a*R*,8b*S*)-8-Fluoro-1,8b-dihydroxy-*N*,6-dimethoxy-3a-(4-methoxyphenyl)-3-phenyl-2,3,3a,8b-tetrahydro-1*H*-cyclopenta[*b*]benzofuran-2-carboxamide (**14hb**)

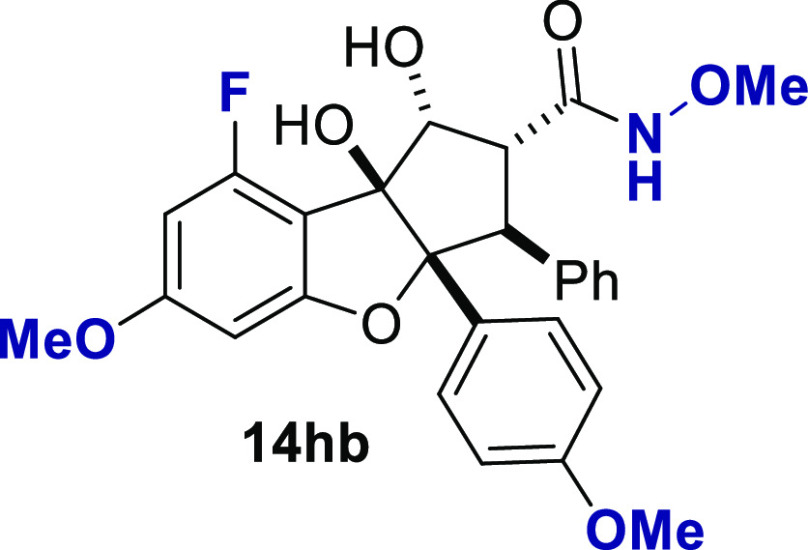



To **13h** (10 mg, 0.02 mmol, 1.00 equiv) in dry CH_2_Cl_2_ (2.2 mL) were added HOBt·H_2_O (4.4 mg, 0.03 mmol, 1.30 equiv), EDC·HCl (6.2 mg, 0.03 mmol, 1.50 equiv) and H_2_NOMe·HCl (8.9 mg, 0.11 mmol, 5.00 equiv) and cooled down to 0 °C. Et_3_N (15 μL, 0.11 mmol, 5.00 equiv) was added dropwise at 0 °C and the mixture stirred at the same temperature for 10 min. The reaction mixture was warmed up to rt and stirred for 16 h. After the reaction was finished, the mixture was concentrated *in vacuo* and purified by silica gel column chromatography (85% MeOH in CH_2_Cl_2_) to afford **14hb** as a colorless oil (3.6 mg, 7.3 μmol, 34% over two steps). *R*_f_ = 0.48 (EtOAc); ^**1**^**H NMR** (DMSO-*d*_6_, 400 MHz): δ [ppm] 11.16 (s, 1H, CON*H*(OCH_3_)), 7.06–6.97 (m, 3H, *H*-2″, *H*-4″, *H*-6″), 7.03–7.00 (m, 2H, *H*-2′, *H* 6′), 6.88 (d, *J* = 7.5 Hz, 2H, *H*-3″, *H*-5″), 6.62 (d, *J* = 8.9 Hz, 2H, *H*-3′, *H*-5′), 6.49 (d, *J* = 1.9 Hz, 1H, *H*-5), 6.29 (dd, *J* = 11, 2.0 Hz, 1H, *H*-7), 5.46 (s, 1H, O*H*), 5.35 (d, *J* = 5.9 Hz, 1H, O*H*), 4.55 (d, *J* = 5.8 Hz, 1H, *H*-1), 4.16 (d, *J* = 14 Hz, 1H, *H* 3), 3.78 (s, 3H, *H*_3_CO-4′), 3.61 (s, 3H, *H*_3_CO-6), 3.58 (dd, *J* = 14, 5.6 Hz, 1H, *H*-2), 3.48 (s, 3H, CONH(OC*H*_3_)); ^**13**^**C NMR** (DMSO-*d*_6_, 100 MHz): δ [ppm] 166.8 (q, *C*-11), 162.9 (q, *d*, *J* = 13 Hz, *C*-6), 161.4 (q, *d*, *J* = 12 Hz, *C*-4), 160.7 (q, *d*, *J* = 249 Hz, *C*-8), 158.1 (q, *C*-4′), 138.5 (q, *C*-1″), 129.1 (t, *C*-2′, *C*-6′), 128.6 (q, *C-*1′), 128.2 (t, *C*-2′, *C*-6′), 126.4 (t, *C*-4″), 112.4 (t, *C*-3′, *C*-5′), 109.1 (q, *d*, *J* = 25 Hz, *C*-7), 93.9 (q, *C*-8b), 92.6 (t, *d*, *J* = 2.7 Hz, *C*-5), 79.0 (t, *C*-1), 63.6 (p, CONH(O*C*H_3_)), 56.3 (p, *C*H_3_O-6), 55.3 (p, *C*H_3_O-4′), 55.0 (t, *C*-3), 48.7 (t, *C*-2); **HRMS (ESI**^**+**^**)***m*/*z* calcd for C_27_H_26_FNO_7_Na [M+Na]^+^ 518.1591, found 518.1592; **HPLC purity** 98.26%.

### Synthesis of (±)-(1*R*,2*R*,3*S*,3a*R*,8b*S*)-6-Bromo-1,8b-dihydroxy-*N*,8-dimethoxy-3a-(4-methoxyphenyl)-3-phenyl-2,3,3a,8b-tetrahydro-1*H*-cyclopenta[*b*]benzofuran-2-carboxamide (**14m**)

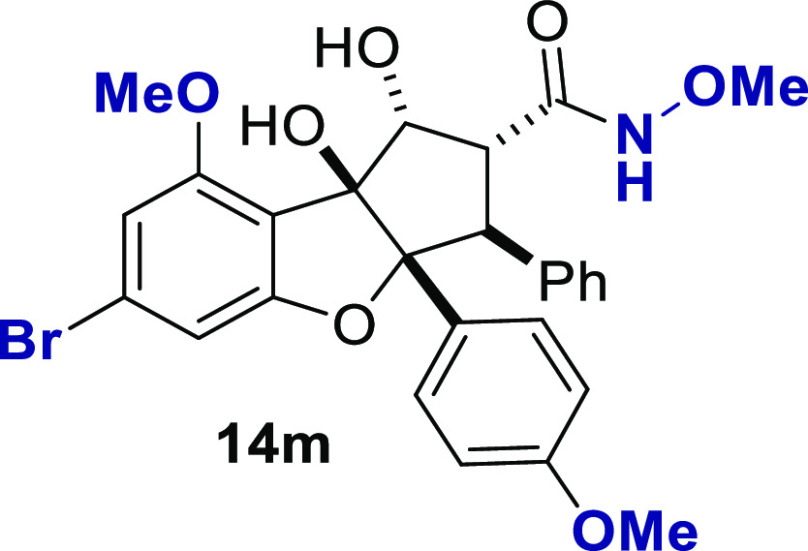



#### (±)-(1*R*,2*R*,3*S*,3a*R*,8b*S*)-6-Bromo-1,8b-dihydroxy-8-methoxy-3a-(4-methoxyphenyl)-3-phenyl-2,3,3a,8b-tetrahydro-1*H*-cyclopenta[*b*]benzofuran-2-carboxylic acid (**13m**)

A solution of methyl ester **9m** (139 mg, 257 μmol, 1.00 equiv) and lithium hydroxide solution (2.00 M in H_2_O, 257 μL, 513 μmol, 2.00 equiv) in MeOH (4.01 mL) was heated at 50 °C for 2 h. Subsequently, the solution was allowed to cool to rt, acidified with HCl (1.00 M in H_2_O) to pH = 1–2 and diluted with CH_2_Cl_2_ (10.0 mL) and H_2_O (10.0 mL). The organic layer was collected. The aqueous layer was extracted with CH_2_Cl_2_ (2×). The combined organic layers were dried over MgSO_4_, filtered and concentrated under reduced pressure to give crude rocagloic acid **13m** as a yellowish solid (135 mg) and used directly for the next step. *R*_f_ = 0.39 (EtOAc).

#### (±)-(1*R*,2*R*,3*S*,3a*R*,8b*S*)-6-Bromo-1,8b-dihydroxy-*N*,8-dimethoxy-3a-(4-methoxyphenyl)-3-phenyl-2,3,3a,8b-tetrahydro-1*H*-cyclopenta[*b*]benzofuran-2-carboxamide (**14m**)

To a solution of rocagloic acid **13f** (135 mg, 257 μmol, 1.00 equiv) in CH_2_Cl_2_ (18.3 mL) EDC·HCl (73.8 mg, 385 μmol, 1.50 equiv), HOBt·H_2_O (54.5 mg, 347 μmol, 1.35 equiv) and triethylamine (642 μL, 1.28 mmol, 5.00 equiv) were added and was stirred at rt. After 1 h, methoxylamine hydrochloride (107 mg, 1.28 mmol, 5.00 equiv) was added and reaction mixture was stirred for additional 5 h. The reaction was terminated by addition of HCl (1.00 M in H_2_O), extracted with CH_2_Cl_2_ (3×). The combined organic layers were dried over MgSO_4_, filtered and concentrated under reduced pressure. The crude product was purified by flash chromatography (CH_2_Cl_2_/MeOH 100:0 → 95:5). The desired rocagloic amide **14m** was obtained as a colorless solid (40.8 mg, 73.3 μmol, 29% over two steps). *R*_f_ = 0.33 (CH_2_Cl_2_/MeOH 95:5); ^**1**^**H NMR** (DMSO-*d*_6_, 400 MHz): δ [ppm] 11.18 (s, 1H, N*H*OCH_3_), 7.06–7.03 (m, 2H, *H*-2″, *H*-6″), 7.00–6.91 (m, 5H, *H*-2′, *H*-6′, *H*-3″, *H*-4″, *H*-5″), 6.87 (d, *J* = 1.3 Hz, 1H, *H*-5), 6.72 (d, *J* = 1.4 Hz, 1H, *H*-7, 6.59 (d, *J* = 8.9 Hz, 2H, *H*-3′, *H*-5′), 5.25 (s, 1H, O*H*-8b), 4.94 (d, *J* = 4.7 Hz, 1H, O*H*-1), 4.53 (t, *J* = 4.8 Hz, 1H, *H*-1), 4.26 (d, *J* = 14.1 Hz, 1H, *H*-3), 3.76 (s, 3H, *H*_3_CO-8), 3.62 (dd, *J* = 14.4, 5.0 Hz, 1H, *H*-2), 3.59 (s, 3H, s, 3H, *H*_3_CO-4′), 3.50 (s, 3H, NHOC*H*_3_); ^**13**^**C NMR** (DMSO-*d*_6_, 100 MHz): δ [ppm] 166.4 (q, *C*-11), 160.4 (q, *C*-4a), 158.2 (q, *C*-8), 157.6 (q, *C*-4′), 138.1 (q, *C*-1″), 128.6 (t, *C*-2′, *C*-6′), 128.2 (q, *C*-1′), 127.8 (t, *C*-3″, *C* 5″), 127.5 (t, *C*-2″, *C*-6″), 125.8 (t, *C*-4″), 122.7 (q, *C*-6), 115.4 (q, *C*-8a), 111.8 (t, *C*-3′, *C*-5′), 107.3 (t, *C*-7), 106.3 (t, *C*-5), 101.4 (q, *C*-3a), 93.4 (q, *C*-8b), 78.8 (t, *C*-1), 63.1 (p, NHO*C*H_3_), 55.9 (p, H_3_*C*O-8), 54.8 (p, H_3_*C*O-4′), 54.6 (t, *C*-3), 48.5 (t, *C*-2); **HRMS (ESI**^**+**^**)***m*/*z* calcd for C_27_H_26_NO_7_BrNa [M+Na]^+^ 578.0790, found 578.0784; **HPLC purity** 99.69%.

### Biological Evaluation: Virus Infection and Cytotoxicity

#### Cell Culture

Human hepatoma cells (HepG2) were cultured in Dulbecco’s modified Eagle’s medium (DMEM) (Invitrogen, Karlsruhe, Germany) supplemented with 10% fetal calf serum (FCS) (GE Healthcare), 100 μg/mL of streptomycin, 100 IU/mL of penicillin (Invitrogen), 2 mM l-glutamine and 1% non-essential amino acids (Invitrogen) at 37 °C in a 5% (v/v) CO_2_ incubator. Cells were grown on sterile collagen-coated (SERVA Electrophoresis GmbH, Heidelberg, Germany) culture plates. Huh7 cells were maintained in DMEM supplemented with 10% FCS, 2 mM l-glutamine, 0.1 mM non-essential amino acids and 1% penicillin/streptomycin.

African green monkey (*Chlorocebus sp.*) kidney cells (Vero E6, Collection of Cell Lines in Veterinary Medicine CCLV, Friedrich-Loeffler-Institut, Greifswald-Insel Riems, Germany) were grown and maintained in Eagle’s minimal essential medium (MEM; Biochrom GmbH, Berlin, Germany) supplemented with 10% FCS (Biochrom GmbH, Berlin, Germany) and kept under a 5% CO_2_ atmosphere at 37 °C.

#### Virus Isolates

SARS-CoV-2 isolate 2019_nCoV Muc-IMB-1 (accession no. LR824570)^48^ was kindly provided by German Armed Forces Institute of Microbiology (Munich, Germany) and propagated on Vero E6 cells. The RVFV strain MP-12 (accession nos. DQ380154, DQ380208, DQ75404)^49^ was kindly provided by Richard Elliot (University of Glasgow, Centre for virus research, United Kingdom) and propagated on Vero E6 cells (Collection of Cell Lines in Veterinary Medicine, Friedrich-Loeffler-Institut, Germany). Viruses were cultivated and titrated on Vero E6 cells, and stock titers of approximately 10^6^ TCID50 mL^–1^ were achieved.

#### Plasmids and *In Vitro* Transcription

For HEV *in vitro* replication experiments, a plasmid construct harboring the HEV-3 Kernow-C1 p6 sequence coupled with a *Gaussia* luciferase reporter gene (here referred to as p6-Gluc; a kind gift of Suzanne Emmerson, National Institutes of Health, USA) was *in vitro* transcribed according to refs (50 and 51). In brief, 2 μg of linearized plasmid DNA was transcribed with T7 Polymerase (Promega) and capped using Ribom7G Cap Analog (Promega, Madison, WI) at 37 °C for 4 h. Purified *in vitro* transcript was stored at −80 °C. For CHIKV assays, the infectious clone CHIKV LR2006-OPY1 (ECSA genotype) expressing GFP under the control of a subgenomic promoter was used as described previously.^52^ In brief, infectious virus was produced by *in vitro* transcription followed by electroporation of RNA into BHK-21 cells. Supernatant was collected 48 h after electroporation and titrated on HEK 293T.

#### Dose-Dependent Replication Assay (HEV)

For transfection of the p6-Gluc replicon, HepG2 cells were electroporated as previously reported.^53^ Briefly, 5 × 10^6^ cells were electroporated in 400 μL Cytomix containing 2 mM adenosine triphosphate and 5 mM glutathione with 5 μg of *in vitro* transcribed HEV RNA using the Gene Pulser Xcell system (Bio-Rad, Munich, Germany). Afterward, transfected cells were transferred into 12.1 mL fresh DMEM culture medium and seeded onto 96-well plates at a nonconfluent density of 2 × 10^4^ cells/well (in 50 μL volume) or at confluency (4 × 10^4^ cells/well). Four hours post transfection (p.t.), cells were treated with various compound concentrations ranging from 0.15 nM to 1000 nM in a 3-fold serial dilution. At indicated time points p.t., the supernatant was collected and used to examine the effect of rocaglamides derivatives on HEV replication. Samples were stored at 4 °C until luminometer reading.

#### *Gaussia* Luciferase Assay

To determine *Gaussia* luciferase activity, 20 μL of harvested supernatant was added per well on a 96-well LUMITRAC 600 plate, followed by the addition of 60 μL of Coelenterazine. Luminescence was detected for 1 s with a Centro XS^3^ LB 960 luminometer (Berthold Technologies) after shaking for 2 s. Samples were measured in triplicate and read sequentially.

#### Antiviral Assay (SARS-CoV-2 and RVF)

To evaluate the efficiency of the described derivates *in vitro*, Vero E6 cells from overnight cultures were infected with SARS-CoV-2 or RVFV strain MP-12 at a multiplicity of infection (MOI) of 0.1. After infection, the wells were incubated at 37 °C under a 5% CO_2_ atmosphere for 60 min and were then washed with phosphate-buffered saline. Fresh culture medium (MEM supplemented with 5% FCS) containing different compound dilution levels (1:3 dilution; start concentration 1 μM) was added. The supernatants were collected at 24 h post infection (hpi) or 48 hpi including four biological replicates.

#### Quantitative Real-Time RT-PCR (RT-qPCR) Assay

RNA from SARS-CoV-2 and RVFV MP-12 was extracted from all supernatants using the NucleoMag Vet kit (MachereyNagel, Düren, Germany) for a magnetic-bead based isolation of viral RNA according to the manufacturer’s instructions in an elution volume of 100 μL. SARS-CoV-2 RNA was detected by the E-gene Sarbeco 6-carboxyfluorescein RT-qPCR,^54^ detection limit 1 genome copy per μL RNA eluate. The presence of RVF MP-12-derived RNA was analyzed with qRT-PCR^55^ using the QuantiTect Probe RT-PCR Kit (Qiagen, Hilden, Germany).

#### Infection Assay (CHIKV)

For infections assays, 2 × 10^4^ Huh7 cells per well in a 96-well plate were seeded 24 h prior to infection. 100 μL CHIKV ECSA 3′-GFP was added at a MOI 2.5 (based on HEK 293T TCID50) to each well and incubated for 1 h at 37 °C. Meanwhile, compounds were serially diluted in growth medium from 2000 nM to 0.3 nM and 100 μL of compound dilution was added to the designated wells containing virus inoculum in triplicates. GFP expression was documented (10× magnification, 300 ms exposure) until 48 h post infection using the IncuCyte S3 imaging platform (Sartorius). Images were analyzed for total GFP fluorescence intensity per well at 24 and 48 hpi using the manufacturer’s basic analyzer tool.

#### Cell Viability Assay

Cell viability was assessed by 3-(4,5-dimethylthiazol-2-yl)-2,5-diphenyltetrazolium bromide (MTT) assay. Therefore, 0.5 mg/mL MTT substrate (Sigma) diluted in DMEM was added to cells and incubated at 37 °C and 5% CO_2_ for 1–2 h. To solubilize MTT reduction product, medium was removed and replaced with 50 μL DMSO/well. Absorbance was measured at 570 nm with a micro-absorbance reader (Tecan). As background control, cells were treated with 70% ethanol for 10 min.

To measure cellular metabolic activity in SARS-CoV-2 and RVFV infected cells, MTT assay was performed with the Cell Proliferation Kit (Roche, Basel, Schweiz) according to manufacturer’s recommendations. Briefly, Vero E6 cells (1.8 × 10^5^ cells/mL) were seated on a 96-well plate, and after 24 h the different dilutions of the compounds were added and incubated for 24 or 48 h. Afterward, 10 μL MTT was added and incubated for another 4 h, then the solubilization solution was added and the spectrophotometrical absorbance was measured after overnight incubation.

#### Statistics

Data on dose-dependent inhibition of HEV replication were fitted using a nonlinear regression model and EC_90_/CC_50_ values were calculated according to a four-parameter log–logistic model. For compounds that did not reach the half-maximum cytotoxic concentration in the dose-response assay, their CC_50_ values were assigned a default value of 1000 (which was the highest concentration tested). These values were then used to calculate selective indices. To determine EC_50_ and EC_90_ values, Prism GraphPad calculated best-fit values, which were then used to determine SI values. To calculate EC_90_ values in SARS-CoV-2 and RVFV experiments, the virus RNA load determined for nontreated virus-infected cells was set to 100% and RNA values obtained for treated cells were normalized to this value. Data analysis was performed in GraphPad Prism v9.3.1 (La Jolla, California, USA, www.graphpad.com).

## Supporting Information Available

Detailed description of chemical synthesis, analytic description of new compounds and ^1^H and ^13^NMR spectra (PDF)
Biodata for the halogenated rocaglates (CSV)

## Abbreviations Used

4-DMAP: 4-dimethylaminopyridine
Ac: acetyl
APCI: atmospheric-pressure chemical ionization
Bn: benzyl
BHK-21: baby hamster kidney cells
Bz: benzoyl
CHIKV: Chikungunya virus
DMEM: Dulbecco’s modified Eagle medium
DMSO: dimethyl sulfoxide
EDC: 1-ethyl-3-(3-(dimethylamino)propyl)carbodiimide
EI: electron ionization
ESI: electrospray ionization
GC: gas chromatography
GFP: green fluorescent protein
Gluc: *Gaussia* luciferase
HEV: hepatitis E virus
HEK 293T: human embryonic kidney cells
HOBt: hydroxybenzotriazole
HPLC: high-pressure liquid chromatography
*i*Pr: isopropyl
LiHMDS: lithium bis(trimethylsilyl)amide
*m*CPBA: *meta*-chloroperoxybenzoic acid
Me: methyl
MOM: methoxymethyl
MS: mass spectrometry
MTT: 3-(4,5-dimethylthiazol-2-yl)-2,5-diphenyltetrazolium bromide
NMR: nuclear magnetic resonance
OTf: triflate
Ph: phenyl
*p*TsOH: *para*-toluenesulfonic acid
*R*_f_: retention factor
RNA: ribonucleic acid
RVF: Rift Valley fever virus
Sars-CoV-2: severe acute respiratory syndrome coronavirus type 2
TBS: *tert*-butyldimethylsilyl
TCID50: tissue culture infection dose 50
THF: tetrahydrofuran
TMS: trimethylsilyl
TFE: 2,2,2-trifluoroethanol
TLC: thin-layer chromatography
UV: ultraviolet
