# Supplementary material for: Halogenated Rocaglate Derivatives: Pan-antiviral Agents against Hepatitis E Virus and Emerging Viruses
Source: J Med Chem. 2023 Dec 21;67(1):289–321. doi: 10.1021/acs.jmedchem.3c01357 (PMC10788925; doi:10.1021/acs.jmedchem.3c01357)
Supplement: Supplementary file 1 — jm3c01357_si_002.pdf [file jm3c01357_si_002.pdf]

Supporting Information for

Halogenated rocaglate derivatives – pan-antiviral agents against hepatitis E virus and emerging viruses

Catherine Victoria,[a‡] Göran Schulz,[a‡] Mara Klöhn,[b] Saskia Weber,[c] Cora M. Holicki,[c] Yannick Brüggemann,[b] Miriam Becker,[d] Gisa Gerold,[d,e,f], Martin Eiden,[c] Martin H. Groschup,[c] Eike Steinmann,*[b] Andreas Kirschning*[a]

[a] Institute of Organic Chemistry, Leibniz University Hannover, Schneiderberg 1B, 30167 Hannover, Germany

[b] Department of Molecular and Medical Virology, Ruhr-University Bochum, 44081 Bochum, Germany

[c] Federal Research Institute in Animal Health (FLI), Südufer 10, 17493 Greifswald - Insel Riems, Germany

[d] Institute for Biochemistry & Research Center for Emerging Infections and Zoonoses (RIZ), University of Veterinary Medicine Hannover, Bünteweg 2, 30559 Hannover, Germany

[e] Wallenberg Centre for Molecular Medicine (WCMM), Umeå University, 901 87 Umeå, Sweden

[f] Department of Clinical Microbiology, Virology, Umeå University, 901 87 Umeå, Sweden

KEYWORDS Antivirals, Hepatitis E, Rocaglates, SARS-CoV-2, Silvestrol, Total synthesis

* A. Kirschning, E-mail: andreas.kirschning@oci.uni-hannover.de

* E. Steinmann, E-mail: eike.steinmann@ruhr-uni-bochum.de

Table of Contents

1. List of all rocaglates synthesized including atom numbering S4
2. Set-up for reactions under UV-light irradiation S7
3. Chemical syntheses of *ortho*-hydroxy acetophenones **3a-3n** S8
 3.1. 1-(2-Hydroxy-4,6-dimethoxyphenyl)ethan-1-one (**3a**) S8
 3.2. 1-(4-(Benzyloxy)-2-hydroxy-6-methoxyphenyl)ethan-1-one (**3b**) S8
 3.3. 1-(2,4-Difluoro-6-hydroxyphenyl)ethan-1-one (**3c**) S9
 3.4. 1-(2,4-Dichloro-6-hydroxyphenyl)ethan-1-one (**3d**) S10
 3.5. 1-(2,4-Dibromo-6-hydroxyphenyl)ethan-1-one (**3e**) S10
 3.6. 1-(4-Bromo-2-chloro-6-hydroxyphenyl)ethan-1-one (**3f**) and 1-(2-Bromo-4-chloro-6-hydroxyphenyl)ethan-1-one (**3g**) S11
 3.7. 1-(2-Fluoro-6-hydroxy-4-methoxyphenyl)ethan-1-one (**3h**) and 1-(4-Fluoro-2-hydroxy-6-methoxyphenyl)ethan-1-one (**3i**) S12
 3.8. 1-(2-Chloro-6-hydroxy-4-methoxyphenyl)ethan-1-one (**3j**) and 1-(4-Chloro-2-hydroxy-6-methoxyphenyl)ethan-1-one (**3k**) S13
 3.9. 1-(2-Bromo-6-hydroxy-4-methoxyphenyl)ethan-1-one (**3l**) and 1-(4-bromo-2-hydroxy-6-methoxyphenyl)ethan-1-one (**3m**) S14
 3.10. 1-(2-Fluoro-6-hydroxy-4-(methoxymethoxy)phenyl)ethan-1-one (**3n**) S15
4. ^{1}H- and ^{13}C-NMR Spectra S17
 4.1. NMR spectroscopic data for the syntheses of *ortho*-hydroxy acetophenones **3a-3n** S17
 4.2. NMR spectroscopic data for the synthesis of **9a** S38
 4.3. NMR spectroscopic data for the synthesis of **11ba** S43
 4.4. NMR spectroscopic data for the synthesis of **11bb** S50
 4.5. NMR spectroscopic data for the synthesis of **11bc** S56
 4.6. NMR spectroscopic data for the synthesis of **9c** S61
 4.7. NMR spectroscopic data for the synthesis of **9da** S64
 4.8. NMR spectroscopic data for the synthesis of **9db** S67
 4.9. NMR spectroscopic data for the synthesis of **9e** S70
 4.10. NMR spectroscopic data for the synthesis of **9f** S73
 4.11. NMR spectroscopic data for the synthesis of **9g** S76
 4.12. NMR spectroscopic data for the synthesis of **9h** S79
 4.13. NMR spectroscopic data for the synthesis of **9i** S82
 4.14. NMR spectroscopic data for the synthesis of **9j** S85
 4.15. NMR spectroscopic data for the synthesis of 9k S88
 4.16. NMR spectroscopic data for the synthesis of **9l** S91
 4.17. NMR spectroscopic data for the synthesis of **9m** S94
 4.18. NMR spectroscopic data for the synthesis of **9na** S97
 4.19. NMR spectroscopic data for the synthesis of **9nb** S100
 4.20. NMR spectroscopic data for the synthesis of **14aa** S103

4.21. NMR spectroscopic data for the synthesis of **14ab** S104
4.22. NMR spectroscopic data for the synthesis of **14baa** S105
4.23. NMR spectroscopic data for the synthesis of **14bab** S106
4.24. NMR spectroscopic data for the synthesis of *rac*-**1b** S107
4.25. NMR spectroscopic data for the synthesis of *rac*-**1c** S109
4.26. NMR spectroscopic data for the synthesis of **14da** S110
4.27. NMR spectroscopic data for the synthesis of **14f** S112
4.28. NMR spectroscopic data for the synthesis of **14g** S114
4.29. NMR spectroscopic data for the synthesis of **14ha** S116
4.30. NMR spectroscopic data for the synthesis of **14hb** S117
4.31. NMR spectroscopic data for the synthesis of **14m** S118
5. HPLC traces for biologically tested compounds S119
5.1. HPLC trace of **9a** S119
5.2. HPLC trace of **11ba** S119
5.3. HPLC trace of **11bb** S119
5.4. HPLC trace of **11bc** S120
5.5. HPLC trace of **9c** S120
5.6. HPLC trace of **9da** S120
5.7. HPLC trace of **9db** S121
5.8. HPLC trace of **9e** S121
5.9. HPLC trace of **9f** S121
5.10. HPLC trace of **9g** S122
5.11. HPLC trace of **9h** S122
5.12. HPLC trace of **9i** S122
5.13. HPLC trace of **9j** S123
5.14. HPLC trace of **9k** S123
5.15. HPLC trace of **9l** S123
5.16. HPLC trace of **9m** S124
5.17. HPLC trace of **9na** S124
5.18. HPLC trace of **9nb** S124
5.19. HPLC trace of **14aa** S125
5.20. HPLC trace of **14ab** S125
5.21. HPLC trace of **14baa** S125
5.22. HPLC trace of **14bab** S126
5.23. HPLC trace of *rac*-**1b** S126
5.24. HPLC trace of *rac*-**1C** S126
5.25. HPLC trace of **14da** S127
5.26. HPLC trace of **14f** S127

5.27. HPLC trace of **14g** S127
5.28. HPLC trace of **14ha** S128
5.29. HPLC trace of **14hb** S128
5.30. HPLC trace of **14m** S128
6. Supplementary Figures S129
7. References S129

1. List of all rocaglates synthesized including atom numbering

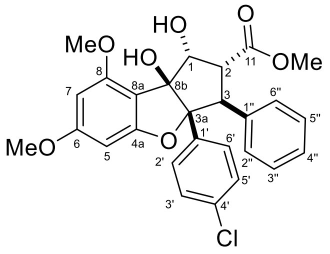

9a

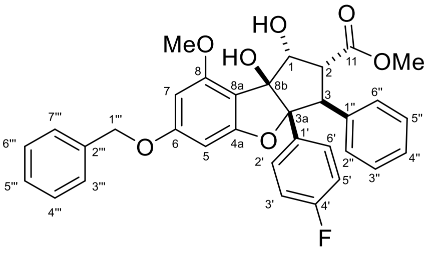

9ba

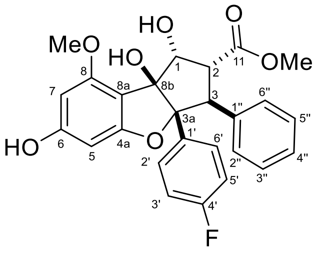

10ba

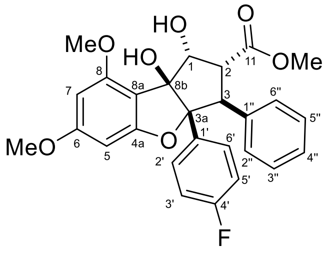

11ba

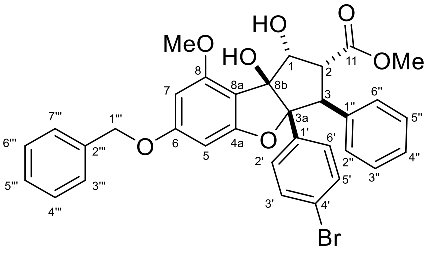

9bb

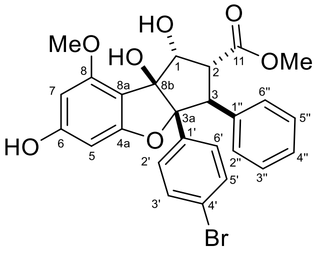

10bb

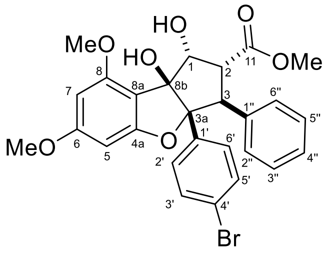

11bb

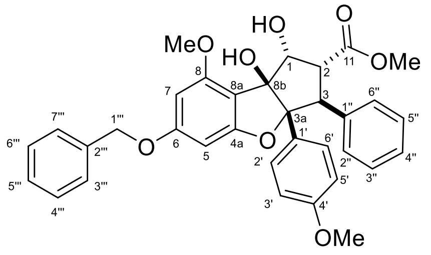

9bc

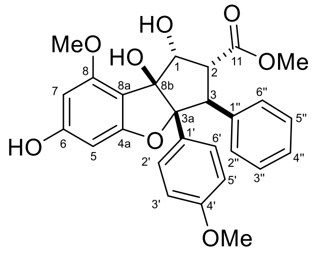

10bc

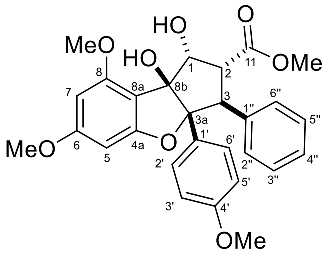

11bc

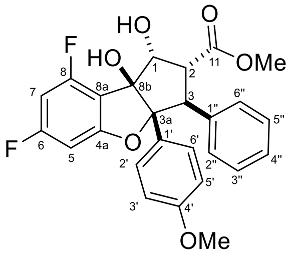

9c

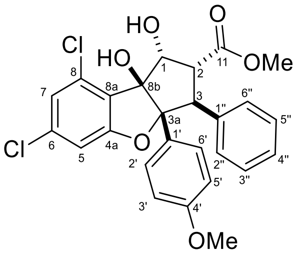

9da

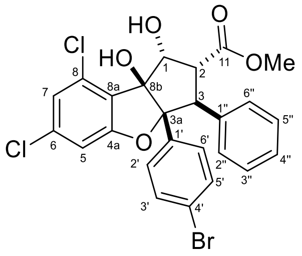

9db

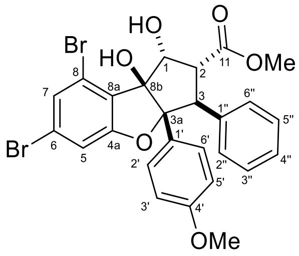

9e

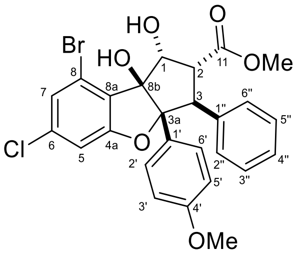

9f

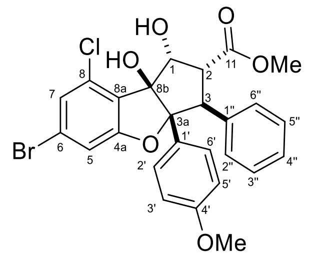

9g

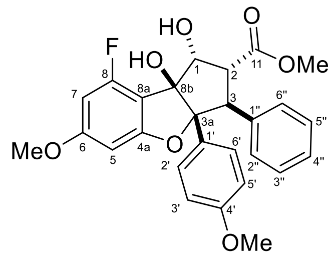

9h

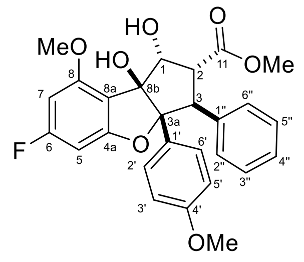

9i

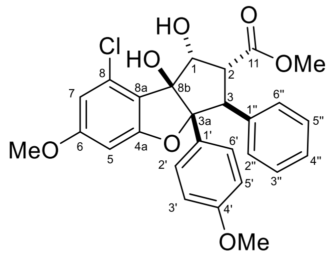

9j

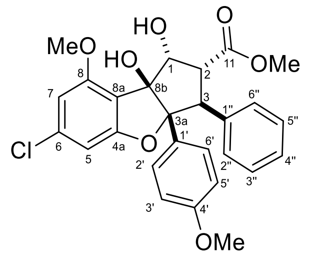

9k

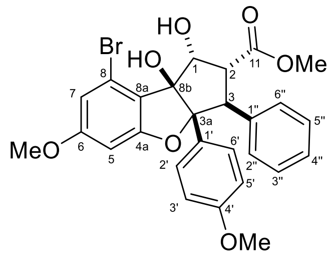

9l

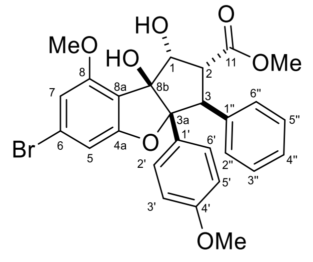

9m

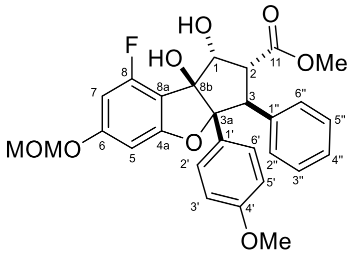

9na

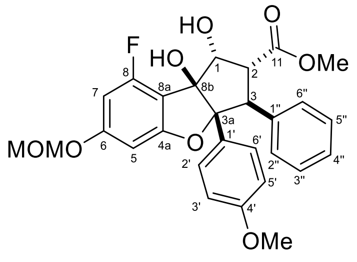

9na

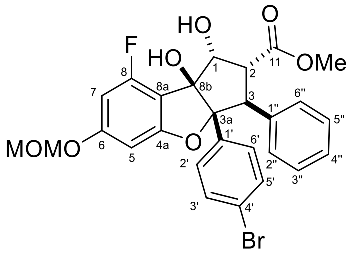

9nb

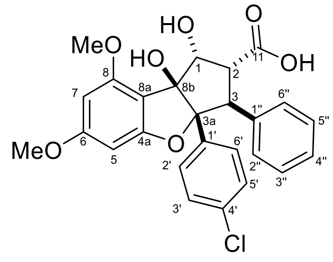

13a

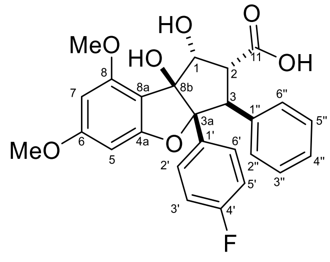

13ba

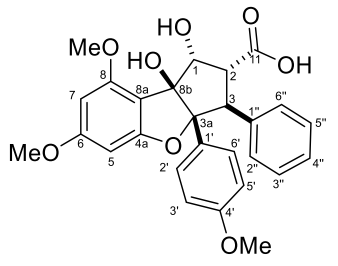

13bc

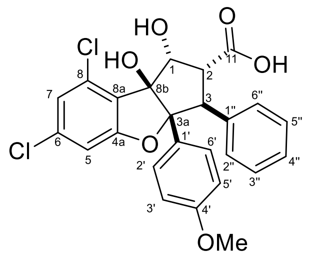

13da

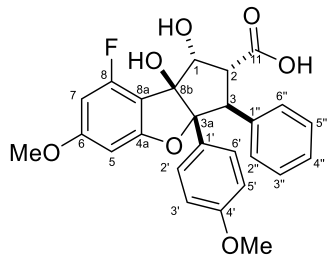

13h

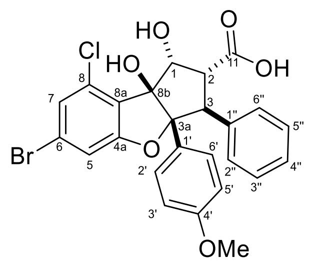

13f

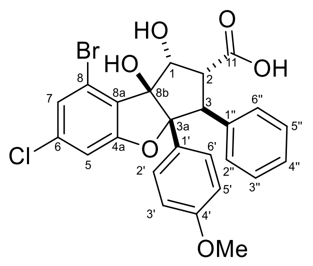

13g

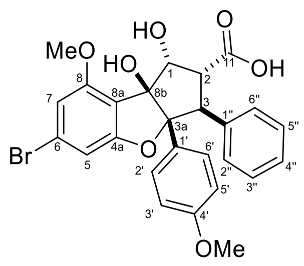

13m

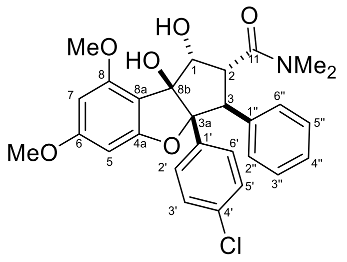

14aa

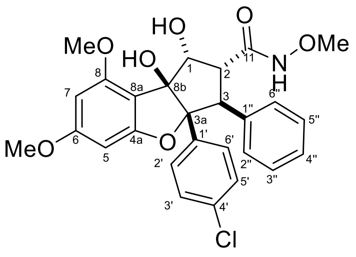

14ab

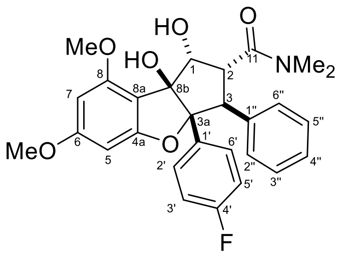

14baa

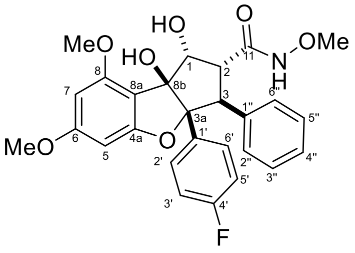

14bab

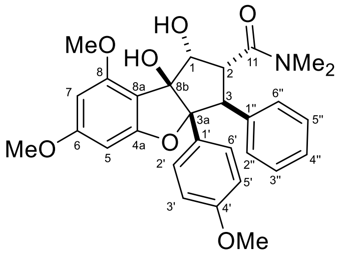

***rac*-1b**

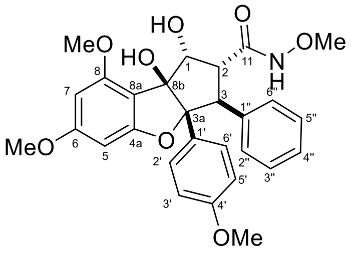

***rac*-1c**

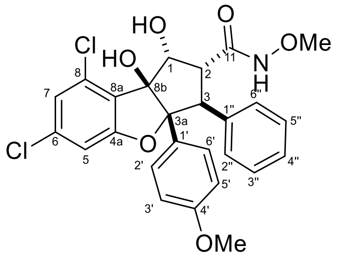

14da

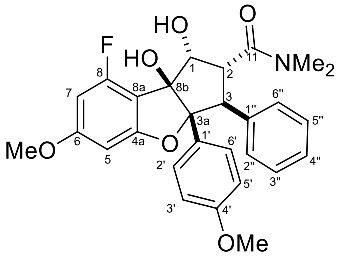

14ha

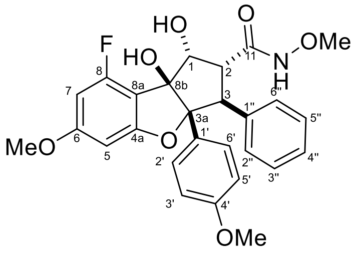

14hb

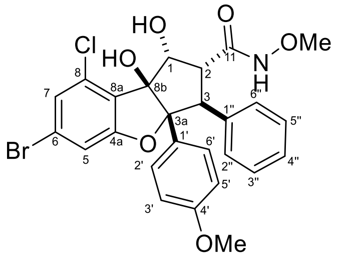

14f

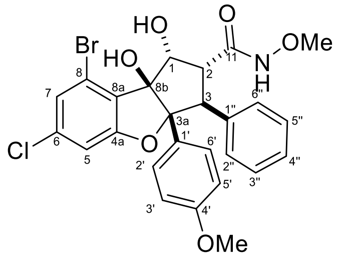

14g

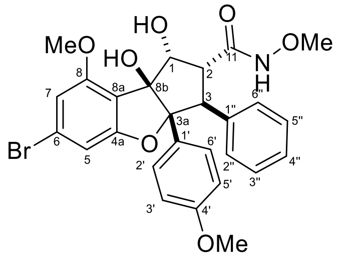

14m

2. Set-up for reactions under UV-light irradiation

Reactions under UV light irradiation were performed using a 365 nm high intensity UV lamp (100 W) by ANALYTIKJENA, an ENDRESS+HAUSER Company. The reaction temperature was adjusted to the temperature specified in the reaction procedure using a cryostat-cooled cooling bath. Standard borosilicate glassware was used.

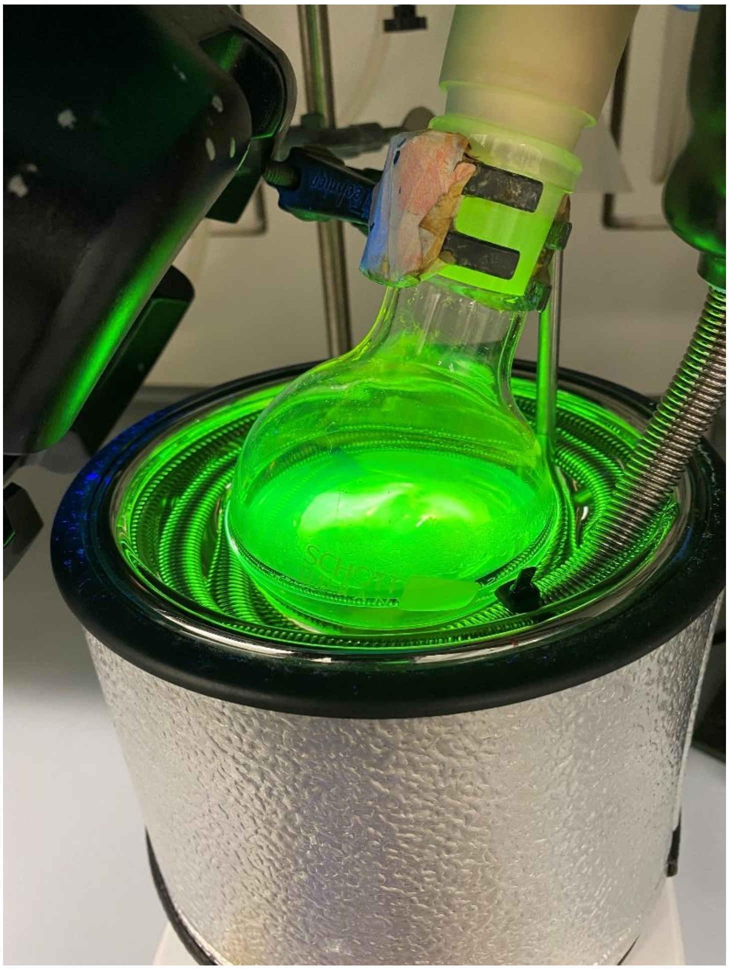

Figure S 1 Set-up for reactions under UV-light irradiation.

3. Chemical syntheses of *ortho*-hydroxy acetophenones 3a-3n

3.1. 1-(2-Hydroxy-4,6-dimethoxyphenyl)ethan-1-one (3a)

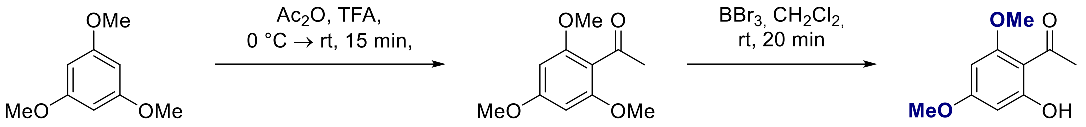

SI-1 **SI-2** **3a**

1-(2,4,6-Trimethoxyphenyl)ethan-1-one (SI-2)

An oven-dried vial was charged with 1,3,5-trimethoxybenzene (**SI-1**) (20.0 g, 119 mmol, 1.00 eq.) and TFA (130 mL). The green solution was cooled to 0 °C and acetic anhydride (22.5 mL, 238 mmol, 2.00 eq.) was added dropwise over a period of 10 min. During this process, the color of the reaction mixture changed to brown. After removing the cooling bath and stirring for 5 min at rt, the reaction mixture was cooled to 0 °C and terminated by the addition of ice-cold H_2O (500 mL). Then, the solution was neutralized with solid NaOH under cooling. The product, which precipitates as a colorless, crystalline solid, was filtered off, washed with H_2O and dried under reduced pressure at 70 °C. 1-(2,4,6-Trimethoxyphenyl)ethan-1-one (**SI-2**) was obtained as a colorless solid (24.2 g, 115 mmol, 97%).

R_f = 0.31 (petroleum ether/MTBE 2:1); **^{1}H-NMR** ($CDCl_3$, 400 MHz): δ [ppm] 6.10 (s, 2H, 2x Ar*H*), 3.82 (s, 3H, OC*H*3), 3.79 (s, 6H, 2x OC*H*3), 2.46 (s, 3H, C*H*3); **^{13}C-NMR** ($CDCl_3$, 100 MHz): δ [ppm] 201.9 (q, *C*=O), 162.5 (q, Ar*C*), 158.5 (q, 2x Ar*C*), 90.7 (t, 2x Ar*C*), 56.0 (p, 2x O*C*H3), 55.6 (p, O*C*H3), 32.7 (p, *C*H3). The analytical data are consistent with those reported in the literature.[S1]

1-(2-Hydroxy-4,6-dimethoxyphenyl)ethan-1-one (3a)

A solution of 2,4,6-trimethoxyacetophenone (**SI-2**) (1.00 g, 4.76 mmol, 1.00 eq.) in CH_2Cl_2 (15.0 mL) was treated with a solution of BBr_3 (1.00 M, 4.99 mL, 4.99 mmol, 1.05 eq.) dropwise at room temperature. The mixture was stirred for 20 min. Then, the reaction was terminated by the addition of KOH solution (4.00 M, 5.00 mL). The biphasic mixture was extracted with CH_2Cl_2 (3x). The combined organic layers were dried over $MgSO_4$, filtered an concentrated under reduced pressure. The residues was subjected to column chromatography (petroleum ether/EtOAc 10:1 → 1:1) to obtain 1-(2-hydroxy-4,6-dimethoxyphenyl)ethan-1-one (**3a**) as a colorless solid (718 mg, 3.66 mmol, 77%).

R_f = 0.53 (petroleum ether/EtOAc 2:1); **^{1}H-NMR** ($CDCl_3$, 400 MHz): δ [ppm] 6.06 (d, J = 2.3 Hz, 1H, Ar*H*), 5.92 (d, J = 2.4 Hz, 1H, Ar*H*), 3.85 (s, 3H, OC*H*3), 3.82 (s, 3H, OC*H*3), 2.61 (s, 3H, C*H*3); **^{13}C-NMR** ($CDCl_3$, 100 MHz): δ [ppm] 203.3 (q, *C*=O), 167.7 (q, Ar*C*), 166.2 (q, Ar*C*), 163.0 (q, Ar*C*), 106.1 (t, Ar*C*), 93.6 (t, Ar*C*), 90.9 (t, Ar*C*), 55.70 (p, O*C*H3), 55.69 (p, O*C*H3), 33.1 (p, *C*H3). The analytical data are consistent with those reported in the literature.[S2]

3.2. 1-(4-(Benzyloxy)-2-hydroxy-6-methoxyphenyl)ethan-1-one (3b)

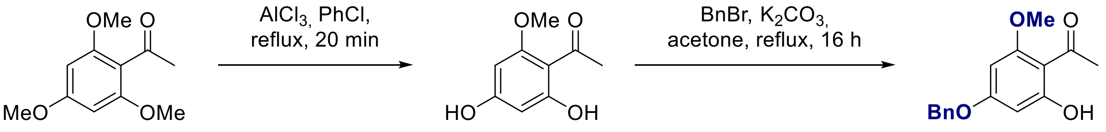

SI-2 **SI-3** **3b**

1-(2,4-Dihydroxy-6-methoxyphenyl)ethan-1-one (SI-3)

A mixture of 1-(2,4,6-trimethoxyphenyl)ethan-1-one (**SI-2**) (24.2 g, 115 mmol, 1.00 eq.), anhydrous $AlCl_3$ (30.7 g, 231 mmol, 2.00 eq.) and chlorobenzene (175 mL) was heated under refluxing conditions for 20 min. Then, the reaction mixture was cooled to 0 °C before HCl (10 wt%, aq.) was added. The resulting precipitate was filtered off and dissolved in EtOAc. The organic phase was extracted using NaOH (10 wt%, aq., 3x). The combined aqueous layers were brought to a pH of 11 with AcOH and extracted with EtOAc (6x). The combined organic phases were dried over $MgSO_4$, filtered and concentrated to obtain the desired product **SI-3** as a pale-yellow solid (9.17 g, 50.4 mmol, 44%).

R*f* = 0.33 (CH_2Cl_2/MeOH 95:5); **^{1}H-NMR** (DMSO-d_6, 400 MHz): δ [ppm] 13.81 (s, 1H, O*H*), 10.4 (s, 1H, O*H*), 5.97 (d, *J* = 2.0 Hz, 1H, Ar*H*), 5.86 (d, *J* = 2.2 Hz, 1H, Ar*H*), 3.82 (s, 3H, OC*H*$_3$), 2.52 (s, 3H, C*H*$_3$); **^{13}C-NMR** (DMSO-d_6, 100 MHz): δ [ppm] 202.3 (q, *C*=O), 166.3 (q, Ar*C*), 165.1 (q, Ar*C*), 163.3 (q, Ar*C*), 104.6 (q, Ar*C*), 95.6 (t, Ar*C*H), 91.3 (t, Ar*C*H), 55.8 (p, O*C*H$_3$), 32.6 (p, *C*H$_3$). The analytical data are consistent with those reported in the literature.[S3]

1-(4-(Benzyloxy)-2-hydroxy-6-methoxyphenyl)ethan-1-one (3b)

1-(2,4-Dihydroxy-6-methoxyphenyl)ethan-1-one (**SI-3**) (11.1 g, 60.9 mmol, 1.00 eq.) was dissolved in dry acetone (250 mL). After adding K_2CO_3 (10.1 g, 73.1 mmol, 1.20 eq.) and benzyl bromide (7.24 mL, 60.9 mmol, 1.00 eq.), the suspension was heated under refluxing conditions for 16 h. Then, the mixture was filtered and the solvent was evaporated. The crude product was purified by column chromatography (petroleum ether/EtOAc 6:1). 1-(4-(Benzyloxy)-2-hydroxy-6-methoxyphenyl)ethan-1-one (**3b**) was obtained as a yellow, highly viscous oil (12.8 g, 46.9 mmol, 77%), which became solid after drying for several hours under high vacuum.

R*f* = 0.54 (petroleum ether/EtOAc 2:1); **^{1}H-NMR** ($CDCl_3$, 400 MHz): δ [ppm] 7.43 – 7.33 (m, 5H, 5x Ar*H*) 6.14 (d, *J* = 2.2 Hz, 1H, Ar*H*), 6.01 (d, *J* = 2.2 Hz, 1H, Ar*H*), 5.07 (s, 2H, C*H*$_2$), 3.84 (s, 3H, OC*H*$_3$), 2.61 (s, 3H, C*H*$_3$); **^{13}C-NMR** ($CDCl_3$, 100 MHz): δ [ppm] 203.3 (q, *C*=O), 167.7 (q, Ar*C*), 165.3 (q, Ar*C*), 163.0 (q, Ar*C*), 136.0 (q, Ar*C*), 128.8 (t, 2x Ar*C*), 128.5 (t, Ar*C*H), 127.8 (t, 2x Ar*C*), 106.3 (q, Ar*C*), 94.5 (t, Ar*C*H), 91.4 (t, Ar*C*H), 70.4 (s, *C*H$_2$), 55.7 (p, O*C*H$_3$), 33.1 (p, *C*H$_3$). The analytical data are consistent with those reported in the literature.[S4]

3.3. 1-(2,4-Difluoro-6-hydroxyphenyl)ethan-1-one (3c)

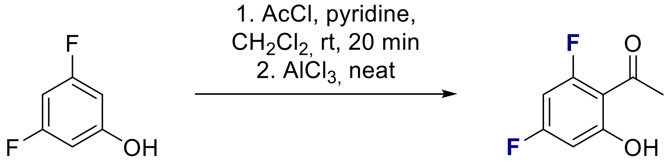

SI-4 **3c**

To a solution of 3,5-difluorophenol (**SI-4**) (3.00 g, 23.1 mmol, 1.00 eq.) in pyridine (2.69 mL, 34.6 mmol, 1.50 eq.) and dry CH_2Cl_2 (30.0 mL) was added AcCl (2.14 mL, 30.0 mmol, 1.30 eq.) slowly at room temperature. The reaction was stirred for 20 min. The reaction was terminated by the addition of a saturated aqueous $NaHCO_3$ solution (20.0 mL). The layers were separated and the aqueous layers were extracted with CH_2Cl_2. The collected organic layers were washed with water and brine, dried over $MgSO_4$, filtered and concentrated *in vacuo*. $AlCl_3$ was then added to the crude product and heated to 150 °C without cooling for 10 min. The reaction was cooled to room temperature, dissolved in EtOAc and terminated carefully with water. The layers were separated, the aqueous layers were extracted with EtOAc (3 x 50.0 mL). The collected organic layers were washed water (2 x 100 mL), brine, dried over

$MgSO_4$, filtered and concentrated *in vacuo*. The crude product was recrystallized in EtOH to afford **3c** as yellow solid (3.11 g, 18.1 mmol, 79%).

R*f* = 0.69 (petroleum ether/EtOAC = 3:1); **^{1}H-NMR** ($CDCl_3$, 400 MHz): δ [ppm] 13.13 (d, *J* = 1.3 Hz, 1H, -O*H*), 6.48 (ddd, *J* = 10, 2.5, 1.6 Hz, 1H, Ar*H*), 6.36 (ddd, *J* = 12, 8.9, 2.5 Hz, 1H, Ar*H*), 2.66 (d, *J* = 7.3 Hz, 3H, C*H*3) ppm; **^{13}C-NMR** ($CDCl_3$, 100 MHz): δ [ppm] 202.0 (q, *d*, *J* = 3.8 Hz, *C*=O), 166.9 (q, *dd*, *J* = 257, 18 Hz, Ar*C*), 165.9 (q, dd, *J* = 17, 7.2 Hz, Ar*C*), 164.7 (q, *dd*, *J* = 258, 16 Hz, Ar*C*), 107.3 (q, dd, *J*= 15, 3.2 Hz, 1C, Ar*C*), 101.3 (t, *dd*, *J* = 24, 3.8 Hz, Ar*C*H), 95.8 (t, *dd*, *J* = 29, 29 Hz, Ar*C*H), 32.1 (p, *d*, *J* = 11 Hz, *C*H3) ppm. **HRMS (CI$^+$)** *m/z* calcd. for $C_8H_7OC_2F$ $[M+H]^+$ 173.0414, found 173.0417.

3.4. 1-(2,4-Dichloro-6-hydroxyphenyl)ethan-1-one (3d)

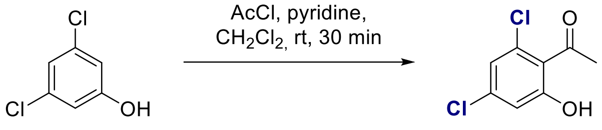

SI-5 3d

To a solution of the 3,5-dichlorophenol (**SI-5**) (10.0 g, 61.3 mmol, 1.00 eq.) and dry pyridine (7.43 mL, 92.0 mmol, 1.50 eq.) in anhydrous CH_2Cl_2 (81.8 mL) was slowly added acetyl chloride (5.70 mL, 79.8 mmol, 1.30 eq.) and the reaction was stirred at rt for 30 min. Then, $NaHCO_3$ solution (aq., sat.) was added and the mixture was extracted with CH_2Cl_2 (3x), washed with H_2O, dried over $MgSO_4$, filtered and concentrated to yield 3,5-dichlorophenyl acetate which was used without further purification. To the compound was added $AlCl_3$ (10.6 g, 79.8 mmol, 1.30 eq.) and the mixture was heated to 150 °C with stirring for 10 min. The reaction mixture was allowed to cool to rt and dissolved in EtOAc before careful terminating with H_2O. After phase separation, the aqueous phase was extracted with EtOAc (3x). The combined organic phases were washed with H_2O, dried over $MgSO_4$, filtered and concentrated under reduced pressure to furnish the crude product as an oil that crystallized on standing. After recrystallization from EtOH, acetophenone **3d** was obtained as a light brown solid (7.89 g, 38.5 mmol, 63%).

R*f* = 0.70 (CH_2Cl_2/EtOAc 85:15); **^{1}H-NMR** ($CDCl_3$, 400 MHz): δ [ppm] 12.76 (s, 1H, O*H*), 6.98 (d, *J* = 2.1 Hz, 1H, Ar*H*), 6.94 (d, *J* = 2.1 Hz, 1H, Ar*H*), 2.84 (s, 3H, C*H*3); **^{13}C-NMR** ($CDCl_3$, 100 MHz): δ [ppm] 204.6 (q, *C*=O), 164.6 (q, Ar*C*), 140.8 (q, Ar*C*), 136.1 (q, Ar*C*), 122.6 (t, Ar*C*H), 118.4 (q, Ar*C*), 117.9 (t, Ar*C*H), 33.8 (p, *C*H3). The analytical data are consistent with those reported in the literature.[S6]

3.5. 1-(2,4-Dibromo-6-hydroxyphenyl)ethan-1-one (3e)

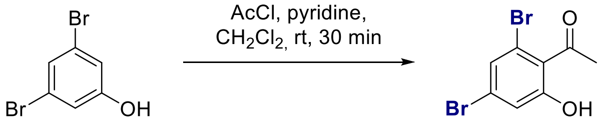

SI-6 3e

To a solution of the 3,5-dibromophenol (**SI-6**) (3.00 g, 11.9 mmol, 1.00 eq.) and dry pyridine (1.44 mL, 17.9 mmol, 1.50 eq.) in dry CH_2Cl_2 (15.9 mL) was slowly added acetyl chloride (1.10 mL, 15.5 mmol, 1.30 eq.) and the reaction was stirred at rt for 30 min. Then, $NaHCO_3$ solution (aq., sat.) was added and the mixture was extracted with CH_2Cl_2 (3x), washed with H_2O, dried over $MgSO_4$, filtered and concentrated to yield 3,5-dibromophenyl acetate which was used without further purification. To the compound was added $AlCl_3$ (2.06 g, 15.5 mmol, 1.30 eq.) and the mixture was heated to 150 °C with

stirring for 10 min. The reaction mixture was allowed to cool to rt and dissolved in EtOAc before careful terminating with H_2O. After phase separation, the aqueous phase was extracted with EtOAc (3x). The combined organic phases were washed with H_2O, dried over $MgSO_4$, filtered and concentrated under reduced pressure to furnish the crude product as an oil that crystallized on standing. After recrystallization from EtOH, acetophenone **3e** was obtained as a light brown solid (2.26 g, 7.67 mmol, 64%).

R*f* = 0.41 (petroleum ether/EtOAc 3:1); **^{1}H-NMR** ($CDCl_3$, 400 MHz): δ [ppm] 12.23 (s, 1H, O*H*), 7.37 (d, *J* = 1.9 Hz, 1H, Ar*H*), 7.15 (d, *J* = 1.9 Hz, 1H, Ar*H*), 2.86 (s, 3H, C*H*$_3$); **^{13}C-NMR** ($CDCl_3$, 100 MHz): δ [ppm] 205.2 (q, *C*=O), 163.6 (q, Ar*C*), 129.0 (q, Ar*C*), 128.7 (t, Ar*C*H), 123.7 (q, Ar*C*), 121.4 (t, Ar*C*H), 118.0 (q, Ar*C*), 33.4 (p, *C*H$_3$). The analytical data are consistent with those reported in the literature.[S7]

3.6. 1-(4-Bromo-2-chloro-6-hydroxyphenyl)ethan-1-one (3f) and 1-(2-Bromo-4-chloro-6-hydroxyphenyl)ethan-1-one (3g)

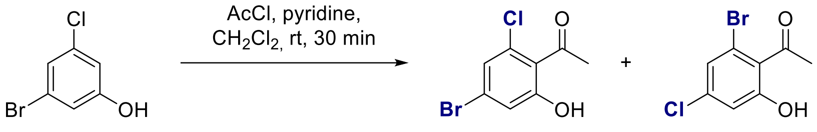

To a solution of the 3-bromo-5-chlorophenol (**SI-7**) (4.00 g, 19.3 mmol, 1.00 eq.) and dry pyridine (1.44 mL, 2.33 mmol, 1.50 eq.) in dry CH_2Cl_2 (25.7 mL) was slowly added acetyl chloride (1.79 mL, 25.1 mmol, 1.30 eq.) and the reaction was stirred at rt for 30 min. Then, $NaHCO_3$ solution (aq., sat.) was added and the mixture was extracted with CH_2Cl_2 (3x), washed with H_2O, dried over $MgSO_4$, filtered and concentrated to yield 3-bromo-5-chlorophenyl acetate which was used without further purification. To the compound was added $AlCl_3$ (3.34 g, 25.1 mmol, 1.30 eq.) and the mixture was heated to 150 °C with stirring for 10 min. The reaction mixture was allowed to cool to rt and dissolved in EtOAc before careful terminating with H_2O. After phase separation, the aqueous phase was extracted with EtOAc (3x). The combined organic phases were washed with H_2O, dried over $MgSO_4$, filtered and concentrated under reduced pressure to furnish the crude product as an oil that crystallized on standing. After recrystallization from EtOH, the mixture of isomers was separated by column chromatography (petroleum ether/EtOAc 19:1) to yield 1-(4-bromo-2-chloro-6-hydroxyphenyl)ethan-1-one (**3f**) as a pale-yellow oily solid (2.07 g, 8.30 mmol, 43%) and 1-(2-bromo-4-chloro-6-hydroxyphenyl)ethan-1-one (**3g**) as a pale-yellow solid (1.07 g, 4.29 mmol, 22%).

1-(4-Bromo-2-chloro-6-hydroxyphenyl)ethan-1-one (3f) R*f* = 0.53 (petroleum ether/EtOAc 10:1); **^{1}H-NMR** ($CDCl_3$, 400 MHz): δ [ppm] 12.69 (s, 1H, O*H*), 7.15 (d, *J* = 2.0 Hz, 1H, Ar*H*), 7.12 (d, *J* = 1.9 Hz, 1H, Ar*H*), 2.83 (s, 3H, C*H*$_3$); **^{13}C-NMR** ($CDCl_3$, 100 MHz): δ [ppm] 204.8 (q, *C*=O), 164.3 (q, Ar*C*), 153.8 (q, Ar*C*), 136.0 (q, Ar*C*), 129.0 (q, Ar*C*), 125.3 (t, Ar*C*H), 121.0 (t, Ar*C*H), 33.8 (p, *C*H$_3$); **HRMS (ESI$^-$)** *m/z* calcd. for $C_6H_5BrOClO_2$ $[M–H]^-$ 246.9161 found 246.9158.

1-(2-Bromo-4-chloro-6-hydroxyphenyl)ethan-1-one (3g) R*f* = 0.29 (petroleum ether/EtOAc 10:1); **^{1}H-NMR** ($CDCl_3$, 400 MHz): δ [ppm] 12.23 (s, 1H, O*H*), 7.37 (d, *J* = 1.9 Hz, 1H, Ar*H*), 7.15 (d, *J* = 1.9 Hz, 1H, Ar*H*), 2.86 (s, 3H, C*H*$_3$); **^{13}C-NMR** ($CDCl_3$, 100 MHz): δ [ppm] 205.0 (q, *C*=O), 163.9 (q, Ar*C*), 140.7 (q, Ar*C*), 126.1 (t, Ar*C*H), 123.8 (q, Ar*C*), 120.6 (q, Ar*C*), 118.3 (t, Ar*C*H), 33.4 (p, *C*H$_3$); **HRMS (ESI$^-$)** *m/z* calcd. for $C_6H_5BrOClO_2$ $[M–H]^-$ 246.9161 found 246.9165.

3.7. 1-(2-Fluoro-6-hydroxy-4-methoxyphenyl)ethan-1-one (3h) and 1-(4-Fluoro-2-hydroxy-6-methoxyphenyl)ethan-1-one (3i)

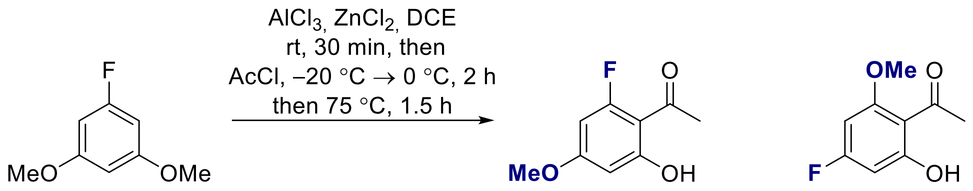

SI-8 **3h** **3i**

A suspension of $AlCl_3$ (853 mg, 6.40 mmol, 1.00 eq.) and $ZnCl_2$ (872 mg, 6.40 mmol, 1.00 eq.) in dichloroethane (8.50 mL) was stirred at room temperature for 30 min. To the suspension was added 1-fluoro-3,5-dimethoxybenzene (**SI-8**) (1.00 g, 6.40 mmol, 1.00 eq.) in dichloroethane (2.90 mL) at 0 °C. Subsequently, AcCl (0.46 mL, 6.40 mmol, 1.00 eq.) was added dropwise into the orange mixture at –20 °C and stirred at 0 °C for 2 h. The mixture was then stirred at 70 °C for 1.5 h before being poured into a cold aqueous HCl solution (1 M, 50.0 mL) and extracted with CH_2Cl_2. The organic layers were washed with water and brine, dried over $MgSO_4$ and concentrated *in vacuo*. The crude extract was purified with highly packed silica gel column chromatography (dry loading; petroleum ether/EtOAc = 20:1) to give 1-(4-Fluoro-2-hydroxy-6-methoxyphenyl)ethan-1-one **3i** as colorless solid (275 mg, 1.49 mmol, 23%) and 1-(2-Fluoro-6-hydroxy-4-methoxyphenyl)ethan-1-one **3h** as pale yellow oil at room temperature (as solid at –20 °C) (459 mg, 2.49 mmol, 39%).

1-(2-Fluoro-6-hydroxy-4-methoxyphenyl)ethan-1-one (3h) R_f = 0.42 (petroleum ether/EtOAc 4:1); **^{1}H-NMR** ($CDCl_3$, 400 MHz): δ [ppm] 13.28 (s, 1H, -O*H*), 6.24 (dd, *J* = 2.5 , 1.1 Hz, 1H, Ar*H*), 6.17 (dd, *J* = 14, 2.5 Hz, 1H, Ar*H*), 3.82 (s, 3H, -OC*H*3), 2.61 (d, *J* = 7.1 Hz, 3H, C(O)C*H*3); **^{13}C-NMR** ($CDCl_3$, 100 MHz): δ [ppm] 201.4 (*q*, d, *J* = 3.9 Hz, *C*=O), 166.0 (q, *d*, *J* = 20 Hz, Ar*C*OH), 165.9 (q, *d*, *J* = 24 Hz, 1C, Ar*C*), 164.7 (q, *d*, *J* = 260 Hz, Ar*C*), 104.6 (q, *d*, *J* = 15 Hz, Ar*C*), 97.4 (t, *d*, *J* = 3.2 Hz, Ar*C*H), 95.1 (t, *d*, *J* = 28 Hz, Ar*C*H), 55.8 (p, O*C*H3), 31.7 (p, *d*, *J* = 10 Hz, C(O)*C*H3); **HRMS (CI$^+$)** *m/z* calcd. for $C_9H_{10}O_3F$ $[M+H]^+$ 185.0614, found 185.0614.

1-(4-Fluoro-2-hydroxy-6-methoxyphenyl)ethan-1-one (3i) R_f = 0.47 (petroleum ether/EtOAc 4:1); **^{1}H-NMR** ($CDCl_3$, 400 MHz): δ [ppm] 13.78 (s, 1H, -O*H*), 6.26 (dd, *J* = 10, 2.4 Hz, 1H, Ar*H*), 6.12 (dd, *J* =11.0 and 2.4 Hz, 1H, Ar*H*), 3.88 (s, 3H, OC*H*3), 2.65 (s, 3H, C(O)C*H*3) ppm; **^{13}C-NMR** ($CDCl_3$, 100 MHz): δ [ppm] 204.0 (q, *C*=O), 167.7 (q, *d*, *J* = 253 Hz, 1C, Ar*C*F), 167.1 (q, *d*, *J* = 17 Hz, Ar*C*), 163.5 (q, *d*, *J* = 14 Hz, Ar*C*), 108.3 (q, *d*, *J* = 2.4 Hz, Ar*C*), 97.5 (t, *d*, *J* = 24 Hz, Ar*C*H), 90.7 (t, *d*, *J* = 26 Hz, Ar*C*H), 55.9 (p, O*C*H3), 33.3 (p, *C*H3); **HRMS (EI)** *m/z* calcd. for $C_9H_9FO_3$ [M] 184.0536, found 184.0540.

3.8. 1-(2-Chloro-6-hydroxy-4-methoxyphenyl)ethan-1-one (3j) and 1-(4-Chloro-2-hydroxy-6-methoxyphenyl)ethan-1-one (3k)

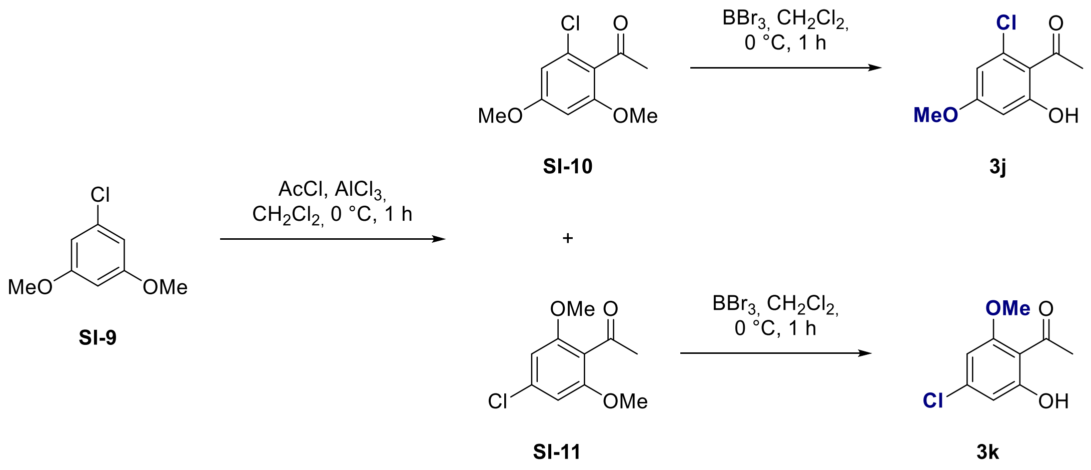

1-(2-Chloro-4,6-dimethoxyphenyl)ethan-1-one (SI-10) and 1-(4-Chloro-2,6-dimethoxyphenyl)ethan-1-one (SI-11)

1-Chloro-3,5-dimethoxybenzene (**SI-9**) (3.05 g, 17.7 mmol, 1.00 eq.) was added to a mixture of $AlCl_3$ (2.83 g, 21.2 mmol, 1.20 eq.) and acetyl chloride (1.32 mL, 18.6 mmol, 1.05 eq.) in CH_2Cl_2 (29.5 mL) at 0 °C. The reaction mixture was stirred for 1 h at the same temperature. Then, H_2O was added and the mixture was extracted with CH_2Cl_2 (3x). The combined organic layers were washed with H_2O and NaCl solution (aq., sat.), dried over $MgSO_4$, filtered and concentrated under reduced pressure. The isomers were separated by column chromatography (petroleum ether/EtOAc 50:1 → 5:1) to yield 1-(2-chloro-4,6-dimethoxyphenyl)ethan-1-one (**SI-10**) as a yellowish oil (1.58 g, 7.36 mmol, 42%) and 1-(4-chloro-2,6-dimethoxyphenyl)ethan-1-one (**SI-11**) as a colorless solid (1.08 g, 5.03 mmol, 28%).

1-(2-Chloro-4,6-dimethoxyphenyl)ethan-1-one (SI-10) R*f* = 0.26 (petroleum ether/EtOAc 4:1); **^{1}H-NMR** ($CDCl_3$, 400 MHz): δ [ppm] 6.49 (d, J = 2.1 Hz, 1H, Ar*H*), 6.36 (d, J = 2.1 Hz, 1H, Ar*H*), 3.795 (s, 3H, OC*H*$_3$), 3.786 (s, 3H, OC*H*$_3$), 2.48 (s, 3H, C*H*$_3$); **^{13}C-NMR** ($CDCl_3$, 100 MHz): δ [ppm] 201.3 (q, *C*=O), 161.3 (q, Ar*C*), 158.0 (q, Ar*C*), 131.1 (q, Ar*C*), 123.8 (q, Ar*C*), 106.2 (t, Ar*C*H), 97.6 (t, Ar*C*H), 56.0 (p, O*C*H$_3$), 55.8 (p, O*C*H$_3$), 32.1 (p, *C*H$_3$); **HRMS (ESI$^+$)** *m/z* calcd. for $C_{10}H_{11}O_3ClNa$ $[M+Na]^+$ 237.0294, found 237.0296.

1-(4-Chloro-2,6-dimethoxyphenyl)ethan-1-one (SI-11) R*f* = 0.32 (petroleum ether/EtOAc 4:1); **^{1}H-NMR** ($CDCl_3$, 400 MHz): δ [ppm] 6.56 (s, 2H, 2x Ar*H*), 3.79 (s, 6H, 2x OC*H*$_3$), 2.45 (s, 3H, C*H*$_3$): **^{13}C-NMR** ($CDCl_3$, 100 MHz): δ [ppm] 201.5 (q, *C*=O), 157.1 (q, 2x Ar*C*), 136.3 (q, Ar*C*), 118.9 (q, Ar*C*), 104.9 (t, 2x Ar*C*H), 56.1 (p, 2x O*C*H$_3$), 32.3 (p, *C*H$_3$). The analytical data are consistent with those reported in the literature.[S8]

1-(2-Chloro-6-hydroxy-4-methoxyphenyl)ethan-1-one (3j)

A solution of 1-(2-chloro-4,6-dimethoxyphenyl)ethan-1-one (**SI-10**) (1.58 g, 7.36 mmol, 1.00 eq.) in CH_2Cl_2 (7.36 mL) at 0 °C was treated with BBr_3 solution (1.00 M in CH_2Cl_2, 8.10 mL, 8.10 mmol, 1.10 eq.). The orange solution was stirred for 1 h at 0 °C. Subsequently, ice-cold H_2O was added to terminate the reaction. The layers were separated and the aqueous layer was extracted with CH_2Cl_2 (3x). The combined organic layers were washed with NaCl solution (aq., sat.), dried over $MgSO_4$, filtered and concentrated under reduced pressure. The crude product was then purified by recrystallization from EtOH to yield the desired phenol **3j** as a greenish solid (979 mg, 4.88 mmol, 66%).

R*f* = 0.39 (petroleum ether/EtOAc 9:1); **¹H-NMR** ($CDCl_3$, 400 MHz): δ [ppm] 13.46 (s, 1H, O*H*), 6.54 (d, *J* = 2.6 Hz, 1H, Ar*H*), 6.36 (d, *J* = 2.6 Hz, 1H, Ar*H*), 3.82 (s, 3H, OC*H*3), 2.80 (s, 3H, C*H*3); **¹³C-NMR** ($CDCl_3$, 100 MHz): δ [ppm] 203.7 (q, *C*=O), 167.2 (q, Ar*C*), 164.3 (q, Ar*C*), 136.8 (q, Ar*C*), 113.6 (q, Ar*C*), 111.3 (t, Ar*C*H), 100.5 (t, Ar*C*H), 55.9 (p, O*C*H3), 33.5 (p, *C*H3); **HRMS (ESI⁻)** *m/z* calcd. for $C_9H_8ClO_3$ $[M–H]^-$ 199.0162, found 199.0166.

1-(4-Chloro-2-hydroxy-6-methoxyphenyl)ethan-1-one (3k)

A solution of 1-(4-chloro-2,6-dimethoxyphenyl)ethan-1-one (**SI-11**) (1.08 g, 5.03 mmol, 1.00 eq.) in CH_2Cl_2 (5.00 mL) at 0 °C was treated with BBr_3 solution (1.00 M in CH_2Cl_2, 5.53 mL, 5.53 mmol, 1.10 eq.). The orange solution was stirred for 1 h at 0 °C. Subsequently, ice-cold H_2O was added to terminate the reaction. The layers were separated and the aqueous layer was extracted with CH_2Cl_2 (3x). The combined organic layers were washed with NaCl solution (aq., sat.), dried over $MgSO_4$, filtered and concentrated under reduced pressure. The crude product was then purified by recrystallization from EtOH to yield the desired phenol **3k** as a yellowish solid (814 mg, 4.06 mmol, 81%).

R*f* = 0.37 (petroleum ether/EtOAc 9:1); **¹H-NMR** ($CDCl_3$, 400 MHz): δ [ppm] 13.49 (s, 1H, O*H*), 6.60 (d, *J* = 2.0 Hz, 1H, Ar*H*), 6.38 (d, *J* = 2.0 Hz, 1H, Ar*H*), 3.90 (s, 3H, OC*H*3), 2.65 (s, 3H, C*H*3); **¹³C-NMR** ($CDCl_3$, 100 MHz): δ [ppm] 204.6 (q, *C*=O), 165.4 (q, Ar*C*), 162.0 (q, Ar*C*), 142.3 (q, Ar*C*), 111.3 (t, Ar*C*H), 109.9 (q, Ar*C*), 102.6 (t, Ar*C*H), 56.1 (p, O*C*H3), 33.6 (p, *C*H3). The analytical data are consistent with those reported in the literature.[S8]

3.9. 1-(2-Bromo-6-hydroxy-4-methoxyphenyl)ethan-1-one (3l) and 1-(4-bromo-2-hydroxy-6-methoxyphenyl)ethan-1-one (3m)

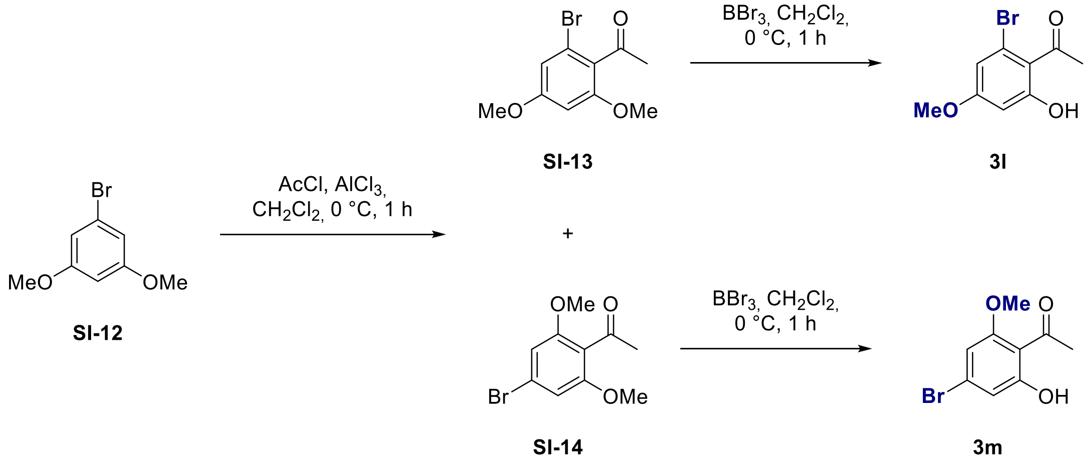

1-(2-Bromo-4,6-dimethoxyphenyl)ethan-1-one (SI-13) and 1-(4-Bromo-2,6-dimethoxyphenyl)ethan-1-one (SI-14)

1-Bromo-3,5-dimethoxybenzene (**SI-12**) (3.00 g, 13.8 mmol, 1.00 eq.) was added to a mixture of $AlCl_3$ (2.21 g, 16.6 mmol, 1.20 eq.) and acetyl chloride (1.14 mL, 14.5 mmol, 1.05 eq.) in CH_2Cl_2 (23.0 mL) at 0 °C. The reaction mixture was stirred for 1 h at the same temperature. Then, H_2O was added and the mixture was extracted with CH_2Cl_2 (3x). The combined organic layers were washed with H_2O and NaCl solution (aq., sat.), dried over $MgSO_4$, filtered and concentrated under reduced pressure. The isomers were separated by column chromatography (petroleum ether/EtOAc 50:1 → 5:1) to yield 1-(2-bromo-4,6-dimethoxyphenyl)ethan-1-one (**SI-13**) as a yellowish oil (575 mg, 2.22 mmol, 16%) and 1-(4-bromo-2,6-dimethoxyphenyl)ethan-1-one (**SI-14**) as a colorless solid (1.12 g, 4.30 mmol, 31%).

1-(2-Bromo-4,6-dimethoxyphenyl)ethan-1-one (SI-13) **R$_f$** = 0.29 (petroleum ether/EtOAc 4:1); **^{1}H-NMR** ($CDCl_3$, 400 MHz): δ [ppm] 6.67 (d, J = 2.1 Hz, 1H, Ar*H*), 6.40 (d, J = 2.1 Hz, 1H, Ar*H*), 3.79 (s, 3H, OC*H*$_3$), 3.78 (s, 3H, OC*H*$_3$), 2.49 (s, 3H, C*H*$_3$); **^{13}C-NMR** ($CDCl_3$, 100 MHz): δ [ppm] 201.2 (q, *C*=O), 161.4 (q, Ar*C*), 157.9 (q, Ar*C*), 125.9 (q, Ar*C*), 118.8 (q, Ar*C*), 109.1 (t, Ar*C*H), 98.3 (t, Ar*C*H), 56.0 (p, O*C*H$_3$), 55.8 (p, O*C*H$_3$), 31.9 (p, *C*H$_3$). The analytical data are consistent with those reported in the literature.[S9]

1-(4-Bromo-2,6-dimethoxyphenyl)ethan-1-one (SI-14) **R$_f$** = 0.39 (petroleum ether/EtOAc 4:1); **^{1}H-NMR** ($CDCl_3$, 400 MHz): δ [ppm] 6.71 (s, 2H, 2x Ar*H*), 3.79 (s, 6H, 2x OC*H*$_3$), 2.44 (s, 3H, C*H*$_3$), **^{13}C-NMR** ($CDCl_3$, 100 MHz): δ [ppm] 201.7 (q, *C*=O), 157.3 (q, 2x Ar*C*), 124.2 (q, Ar*C*), 119.6 (q, Ar*C*), 108.0 (t, 2x Ar*C*H), 56.3 (p, 2x O*C*H$_3$), 32.4 (p, *C*H$_3$). The analytical data are consistent with those reported in the literature.[S8]

1-(2-Bromo-6-hydroxy-4-methoxyphenyl)ethan-1-one (3l)

A solution of 1-(2-chloro-4,6-dimethoxyphenyl)ethan-1-one (**SI-13**) (575 mg, 2.22 mmol, 1.00 eq.) in CH_2Cl_2 (2.22 mL) at 0 °C was treated with BBr_3 solution (1.00 M in CH_2Cl_2, 2.44 mL, 2.44 mmol, 1.10 eq.). The orange solution was stirred for 1 h at 0 °C. Subsequently, ice-cold H_2O was added to terminate the reaction. The layers were separated and the aqueous layer was extracted with CH_2Cl_2 (3x). The combined organic layers were washed with NaCl solution (aq., sat.), dried over $MgSO_4$, filtered and concentrated under reduced pressure. The crude product was then purified by recrystallization from EtOH to yield the desired phenol **3l** as a light brown solid (430 mg, 1.75 mmol, 79%).

R$_f$ = 0.35 (petroleum ether/EtOAc 9:1); **^{1}H-NMR** ($CDCl_3$, 400 MHz): δ [ppm] 13.25 (s, 1H, O*H*), 6.81 (d, J = 2.6 Hz, 1H, Ar*H*), 6.40 (d, J = 2.6 Hz, 1H, Ar*H*), 3.82 (s, 3H, OC*H*$_3$), 2.85 (s, 3H, C*H*$_3$); **^{13}C-NMR** ($CDCl_3$, 100 MHz): δ [ppm] 204.2 (q, *C*=O), 166.9 (q, Ar*C*), 164.2 (q, Ar*C*), 124.8 (q, Ar*C*), 115.5 (q, Ar*C*), 115.3 (t, Ar*C*H), 101.0 (t, Ar*C*H), 55.9 (p, O*C*H$_3$), 33.5 (p, *C*H$_3$); **HRMS (ESI$^-$)** *m/z* calcd. for $C_9H_8BrO_3$ $[M–H]^-$ 242.9657, found 242.9654.

1-(4-Bromo-2-hydroxy-6-methoxyphenyl)ethan-1-one (3m)

A solution of 1-(4-bromo-2,6-dimethoxyphenyl)ethan-1-one (**SI-14**) (1.12 g, 4.30 mmol, 1.00 eq.) in CH_2Cl_2 (4.30 mL) at 0 °C was treated with BBr_3 solution (1.00 M in CH_2Cl_2, 4.73 mL, 4.73 mmol, 1.10 eq.). The orange solution was stirred for 1 h at 0 °C. Subsequently, ice-cold H_2O was added to terminate the reaction. The layers were separated and the aqueous layer was extracted with CH_2Cl_2 (3x). The combined organic layers were washed with NaCl solution (aq., sat.), dried over $MgSO_4$, filtered and concentrated under reduced pressure. The crude product was then purified by recrystallization from EtOH to yield the desired phenol **3m** as a yellowish solid (926 mg, 3.78 mmol, 88%).

R$_f$ = 0.36 (petroleum ether/EtOAc 9:1); **^{1}H-NMR** ($CDCl_3$, 400 MHz): δ [ppm] 13.44 (s, 1H, O*H*), 6.77 (d, J = 1.8 Hz, 1H, Ar*H*), 6.54 (d, J = 1.7 Hz, 1H, Ar*H*), 3.90 (s, 3H, OC*H*$_3$), 2.65 (s, 3H, C*H*$_3$); **^{13}C-NMR** ($CDCl_3$, 100 MHz): δ [ppm] 204.8 (q, *C*=O), 165.2 (q, Ar*C*), 161.8 (q, Ar*C*), 130.8 (q, Ar*C*), 114.4 (t, Ar*C*H), 110.2 (q, Ar*C*), 105.5 (t, Ar*C*H), 56.2 (p, O*C*H$_3$), 33.7 (p, *C*H$_3$). The analytical data are consistent with those reported in the literature.[S8]

3.10. 1-(2-Fluoro-6-hydroxy-4-(methoxymethoxy)phenyl)ethan-1-one (3n)

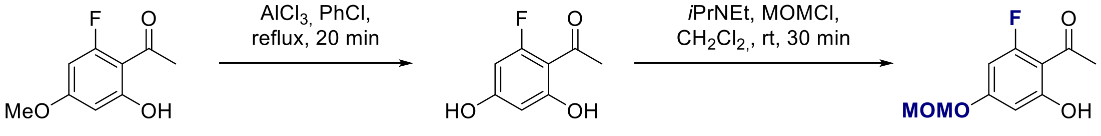

3h **SI-15** **3n**

1-(2-Fluoro-4,6-dihydroxyphenyl)ethan-1-one (SI-15)

To a solution of 1-(2-Fluoro-6-hydroxy-4-methoxyphenyl)ethan-1-one (**3h**) (3.60 g, 19.6 mmol, 1.00 eq.) in chlorobenzene (30.0 mL) was added $AlCl_3$ (3.93 g, 29.5 mmol, 1.50 eq.). The solid mixture was stirred under refluxing conditions for 20 min. The reaction was then cooled down with an ice bath and was terminated by the addition of cold an aqueous HCl solution (1 M, 50.0 mL). The suspension was filtered, the solid was dissolved in EtOAc and methanol. The organic layers were dried with $MgSO_4$, filtered and concentrated *in vacuo*. The crude product was purified *via* silica gel column chromatography (petroleum ether/EtOAc = 8:1, then 3:1, then EtOAc) to afford 1-(2-Fluoro-4,6-dihydroxyphenyl)ethan-1-one (**SI-15**) as a colorless solid (1.89 g, 11.1 mmol, 57%).

R_f = 0.18 (petroleum ether/EtOAc 6:1); **^{1}H-NMR** ($CDCl_3$, 400 MHz): δ [ppm] 12.89 (s, 1H, -OH), 6.18 (dd, *J*= 2.4 and 1.1 Hz, 1H, H-4), 6.14 (dd, *J*= 13 and 2.4 Hz, 1H, H-6), 2.62 (d, *J*= 7.2 Hz, 3H, C-8); **HRMS** (QTOF) *m/z* calc. $C_8H_6FO_3$ $[M\text{-}H]^-$ 169.0301; found 169.0293.

1-(2-Fluoro-6-hydroxy-4-(methoxymethoxy)phenyl)ethan-1-one (3n)

To a solution of 1-(2-Fluoro-4,6-dihydroxyphenyl)ethan-1-one (**SI-15**) (985 mg, 5.79 mmol, 1.00 eq.) in dry CH_2Cl_2 (23.0 mL) was added *i*Pr_2NEt (2.36 mL, 13.3 mmol, 2.30 eq.) and MOMCl (0.59 mL, 7.82 mmol, 1.35 eq.) at 0 °C and stirred at room temperature for 30 min. The reaction was terminated by the addition of saturated aqueous $NaHCO_3$ solution (30.0 mL). The layers were separated, the neutral aqueous layers were extracted with CH_2Cl_2 (3 x 50.0 mL). The collected organic layers were washed with water and brine, dried over $MgSO_4$, filtered and concentrated *in vacuo*. The crude was purified *via* silica gel column chromatography (petroleum ether/EtOAc = 6:1) to afford 1-(2-Fluoro-6-hydroxy-4-(methoxymethoxy)phenyl)ethan-1-one (**3n**) as pale-yellow thin syrup (892 mg, 4.16 mmol, 72%).

R_f = 0.40 (petroleum ether/EtOAc 6:1); **^{1}H-NMR** ($CDCl_3$, 400 MHz): δ [ppm] 13.14 (s, 1H, -O*H*), 6.39 (dd, *J* = 2.5, 1.3 Hz, 1H, Ar*H*), 6.29 (dd, *J* = 14, 2.4 Hz, 1H, Ar*H*), 5.18 (s, 2H, OCH_2OCH_3), 3.47 (s, 3H, OCH_2OCH_3), 2.61 (d, *J* = 7.1 Hz, 3H, CH_3); **^{13}C-NMR** ($CDCl_3$, 100 MHz): δ [ppm] 205.1 (q, *d*, *J* = 3.6 Hz, C=O), 165.7 (q, *d*, *J*= 7.8 Hz, Ar*C*), 164.7 (q, *d*, *J*= 255 Hz, Ar*C*F), 163.4 (q, *t*, *J* = 17 Hz, Ar*C*OH), 105.2 (q, *d*, *J* = 14.9 Hz, Ar*C*), 100.0 (t, *d*, *J* = 3.2 Hz, Ar*C*H), 96.0 (t, *d*, *J* = 28 Hz, Ar*C*H), 94.1 (s, OCH_2OCH_3), 56.5 (p, OCH_2OCH_3), 31.8 (p, *d*, *J*= 11 Hz, C(O)*C*H_3); **HRMS (CI+)** *m/z* calcd. $C_{10}H_{12}FO_4$ for $[M+H]^+$ 215.0720, found 215.0717.

4. ^{1}H- and ^{13}C-NMR Spectra

4.1. NMR spectroscopic data for the syntheses of *ortho*-hydroxy acetophenones 3a-3n

1-(2,4,6-Trimethoxyphenyl)ethan-1-one (SI-2)

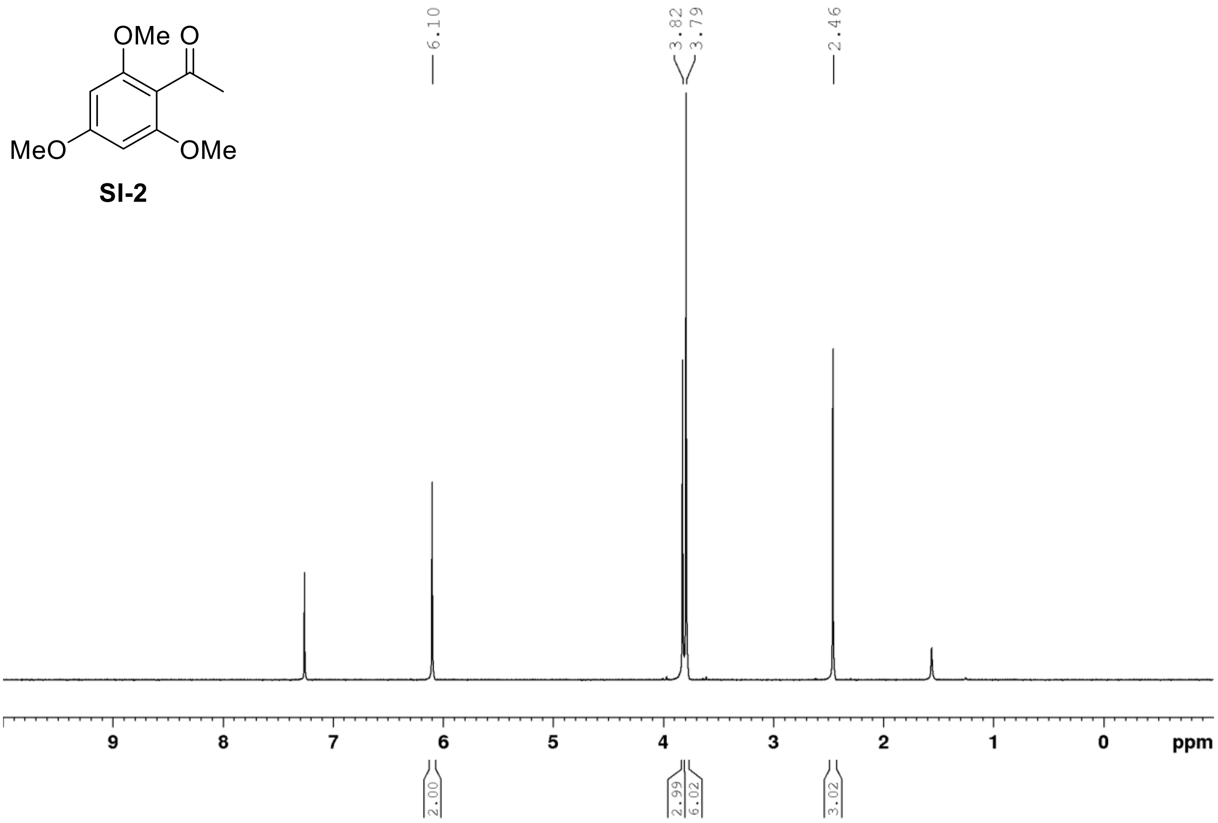

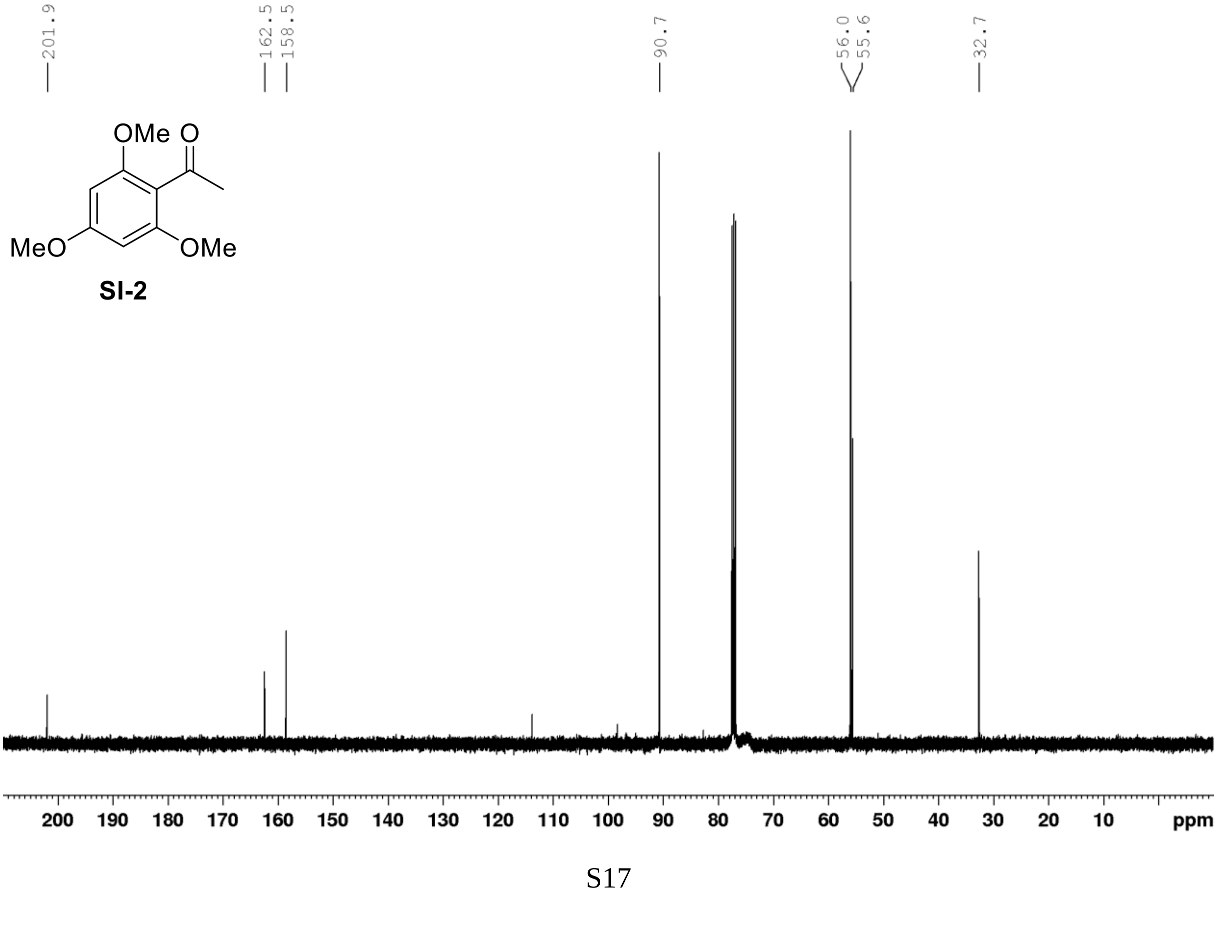

1-(2-Hydroxy-4,6-dimethoxyphenyl)ethan-1-one (3a)

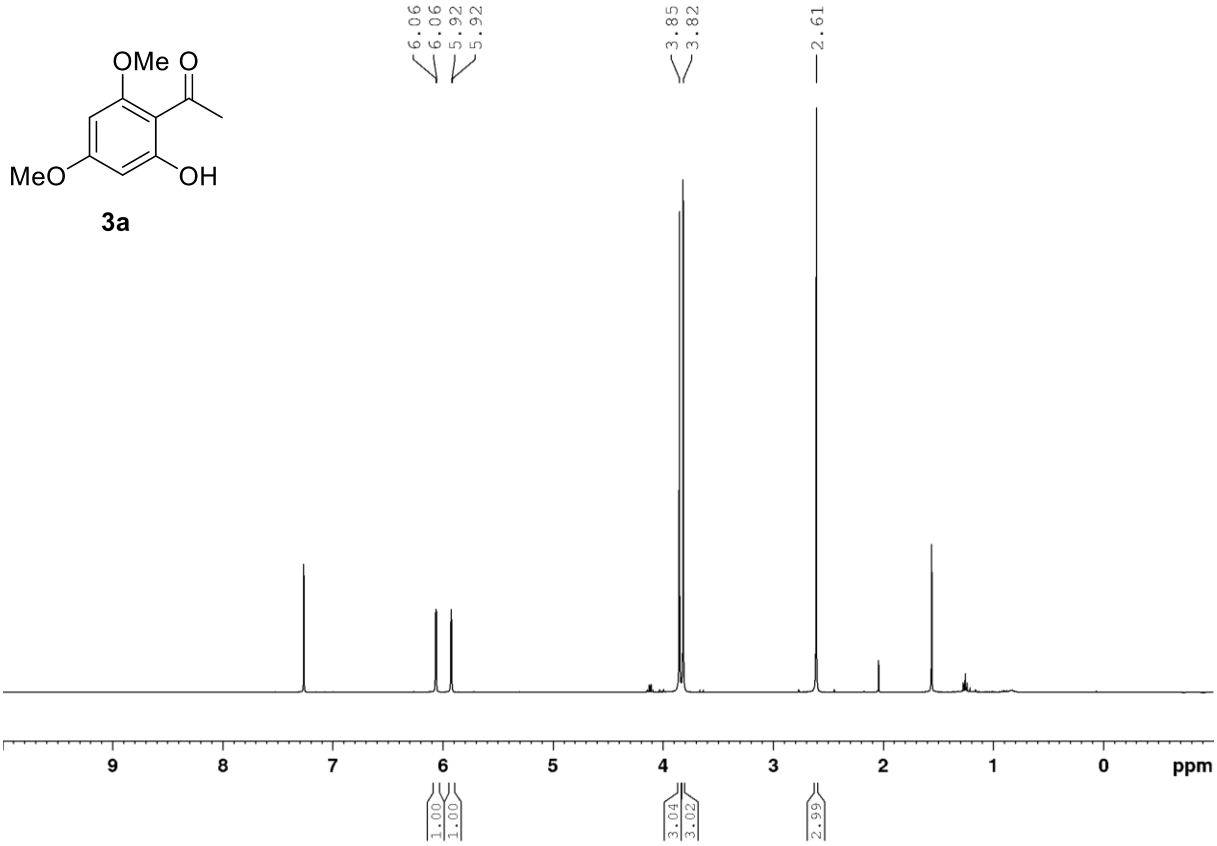

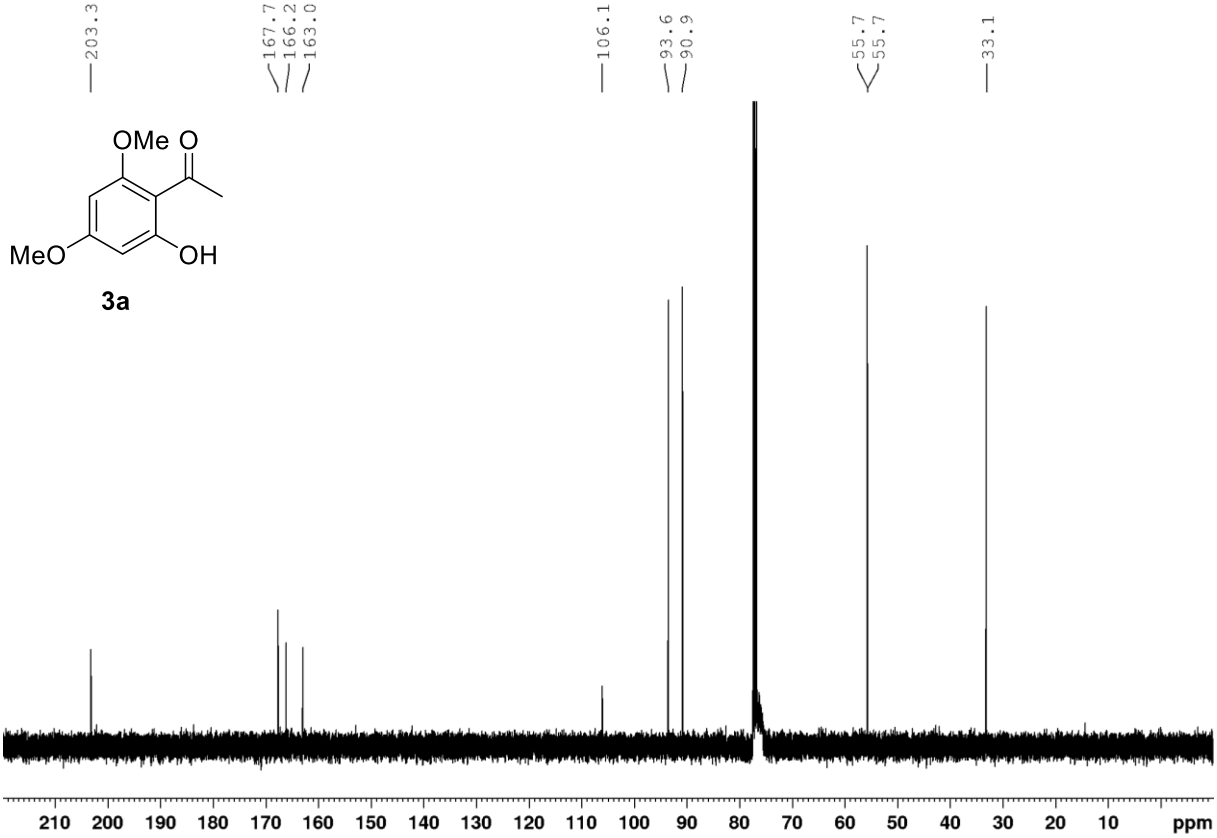

1-(2,4-Dihydroxy-6-methoxyphenyl)ethan-1-one (SI-3)

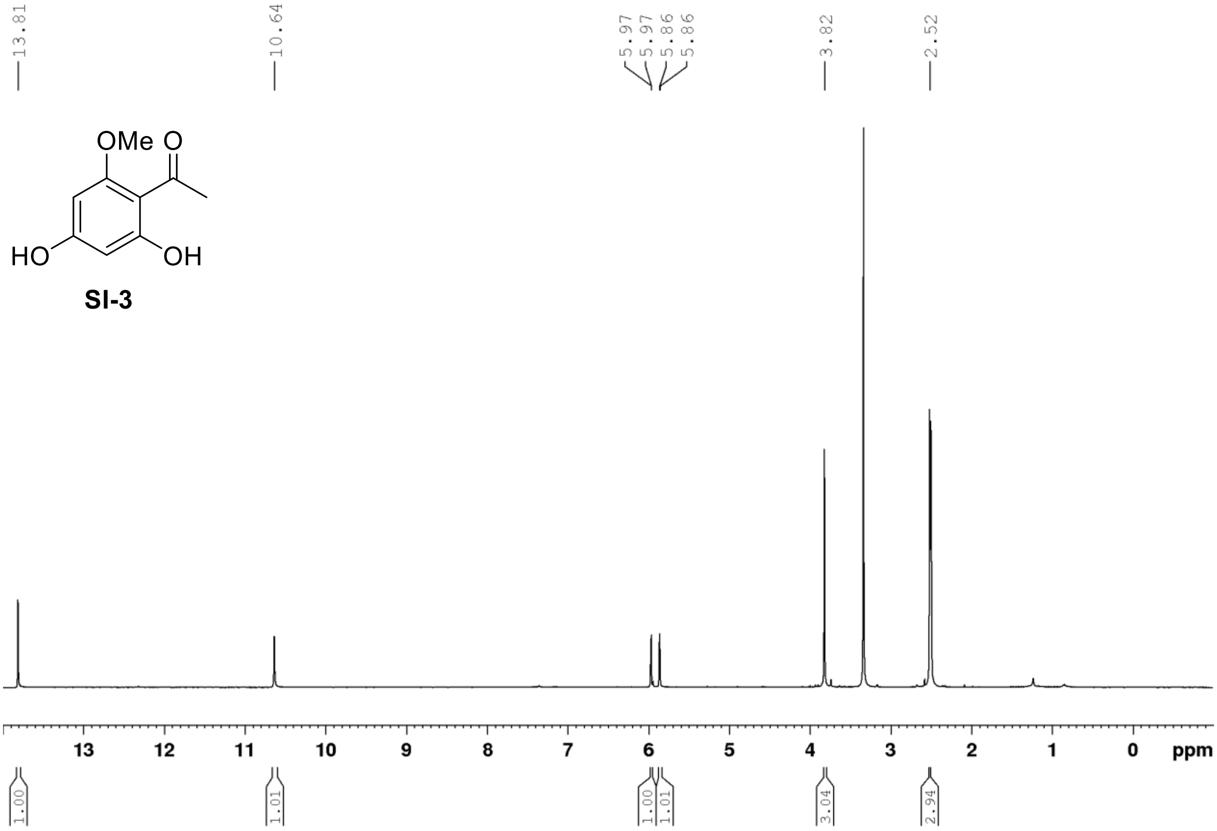

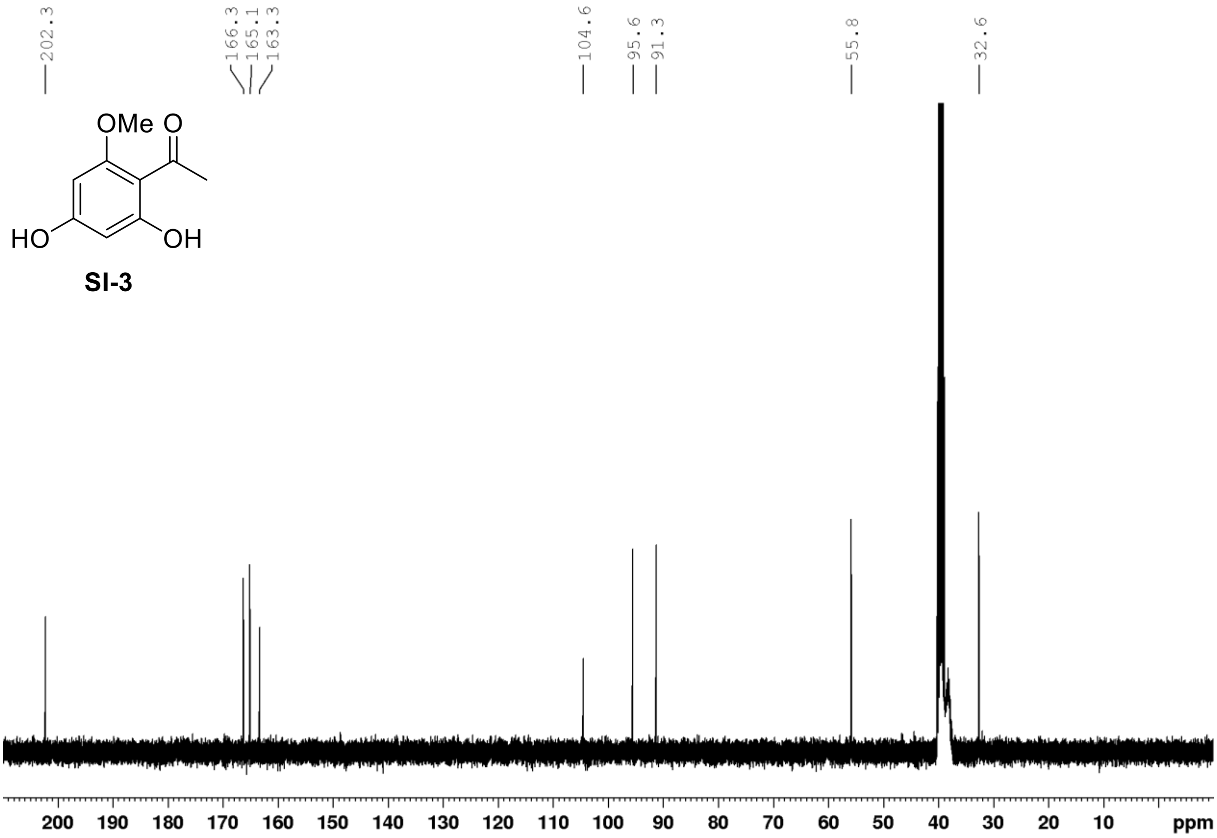

1-(4-(Benzyloxy)-2-hydroxy-6-methoxyphenyl)ethan-1-one (3b)

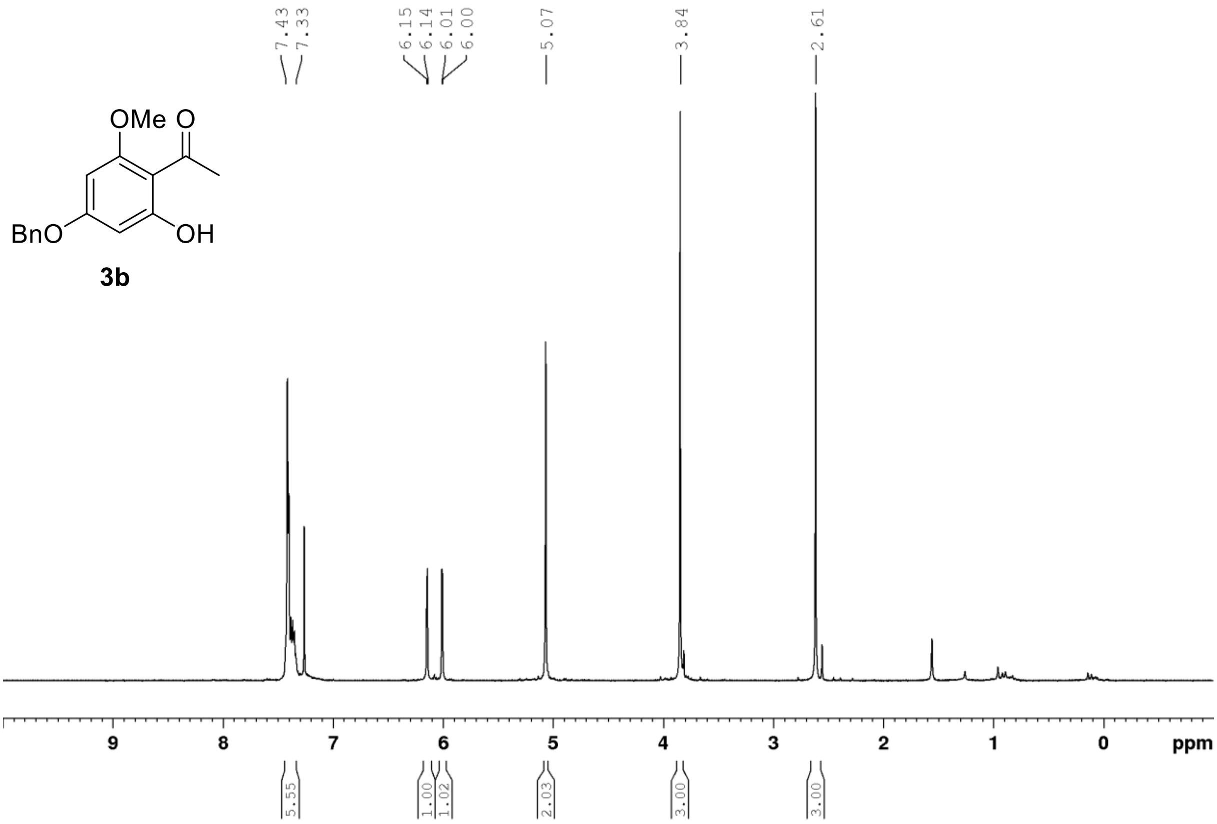

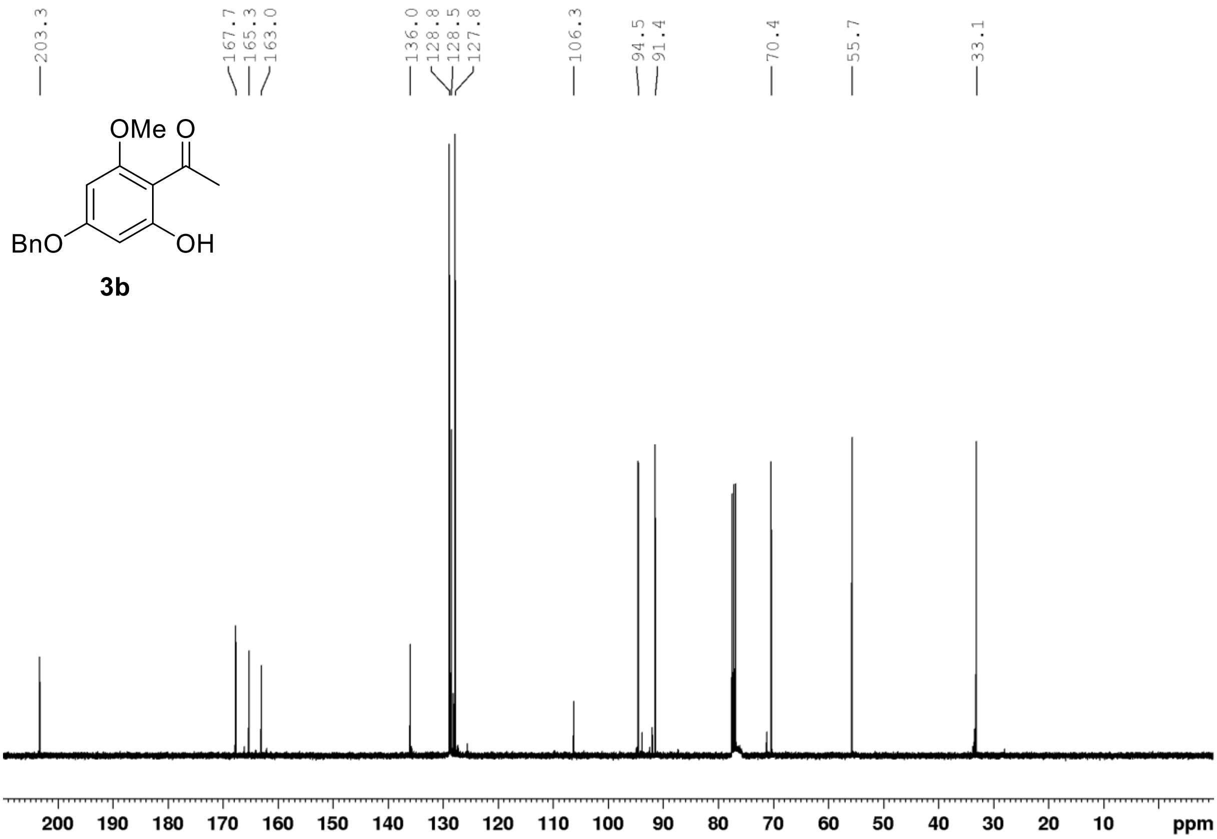

1-(2,4-Difluoro-6-hydroxyphenyl)ethan-1-one (3c)

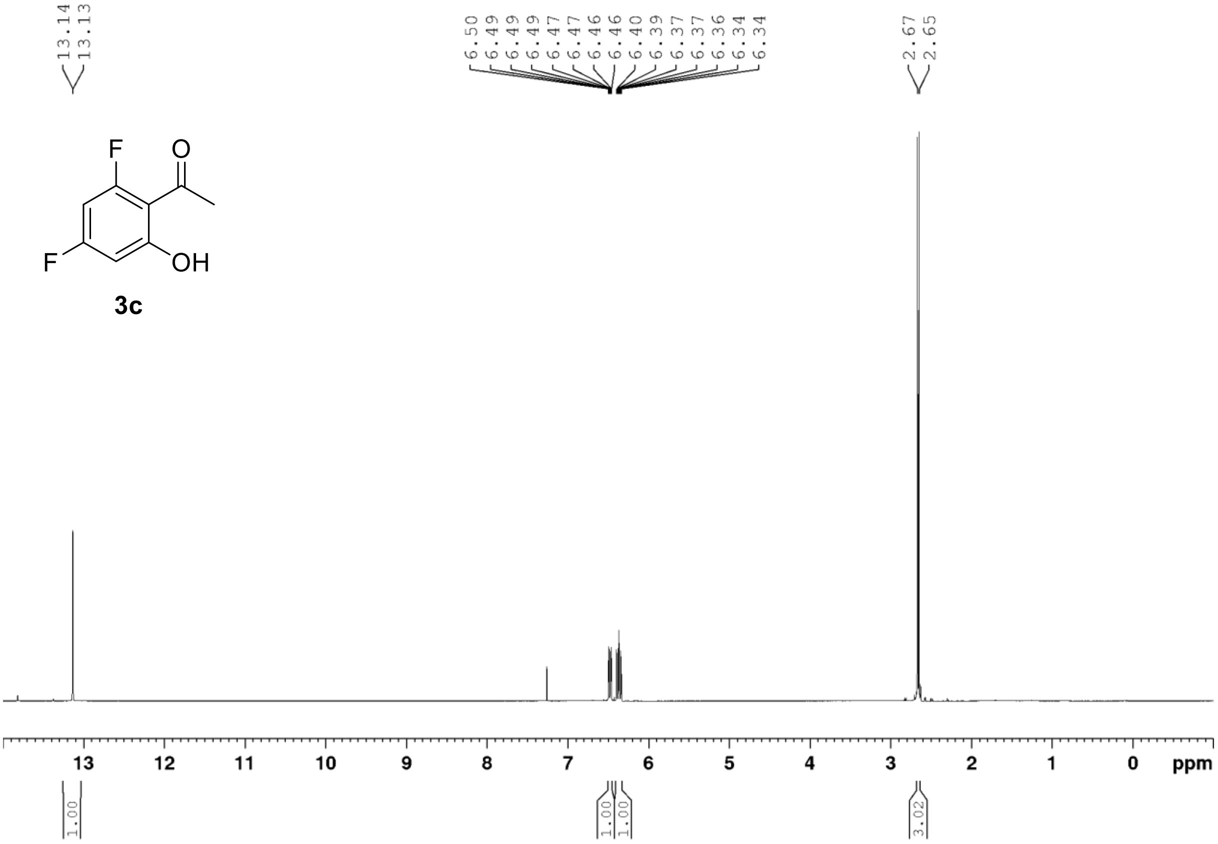

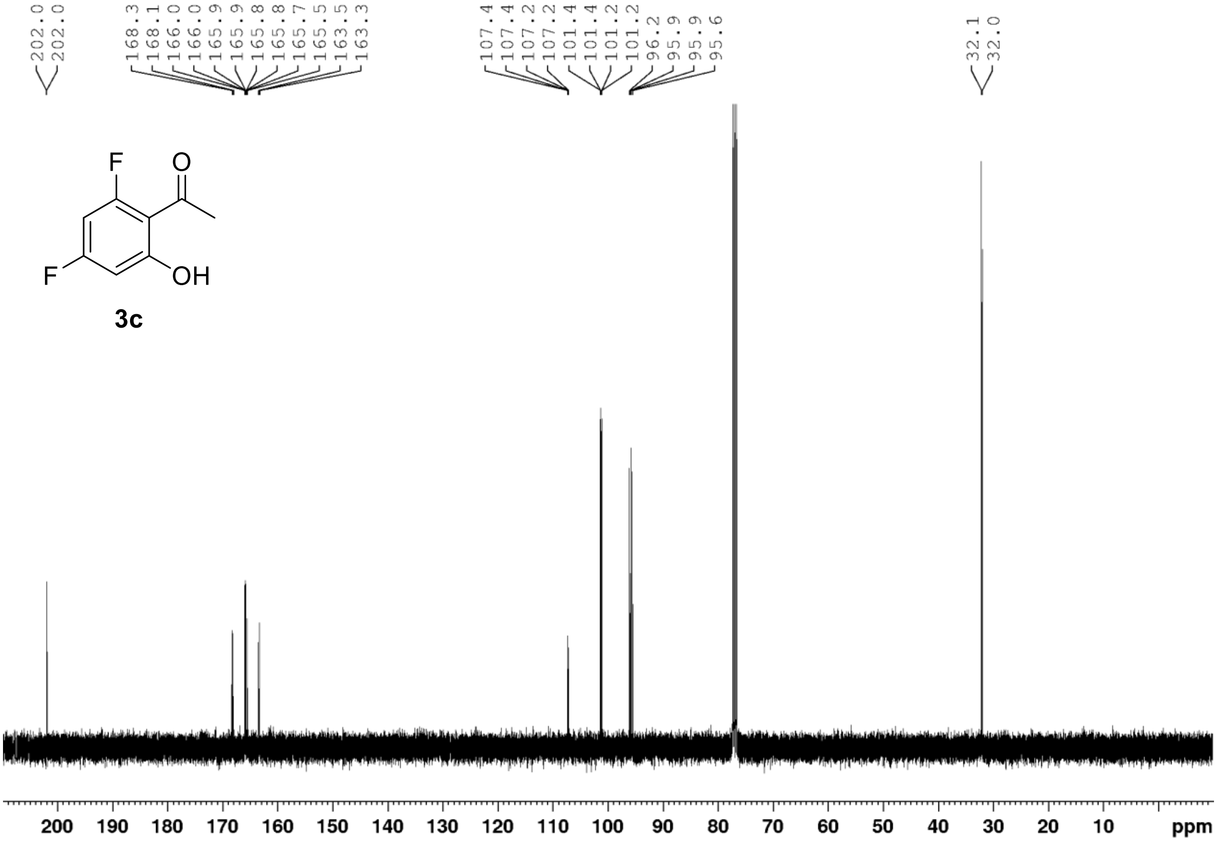

1-(2,4-Dichloro-6-hydroxyphenyl)ethan-1-one (3d)

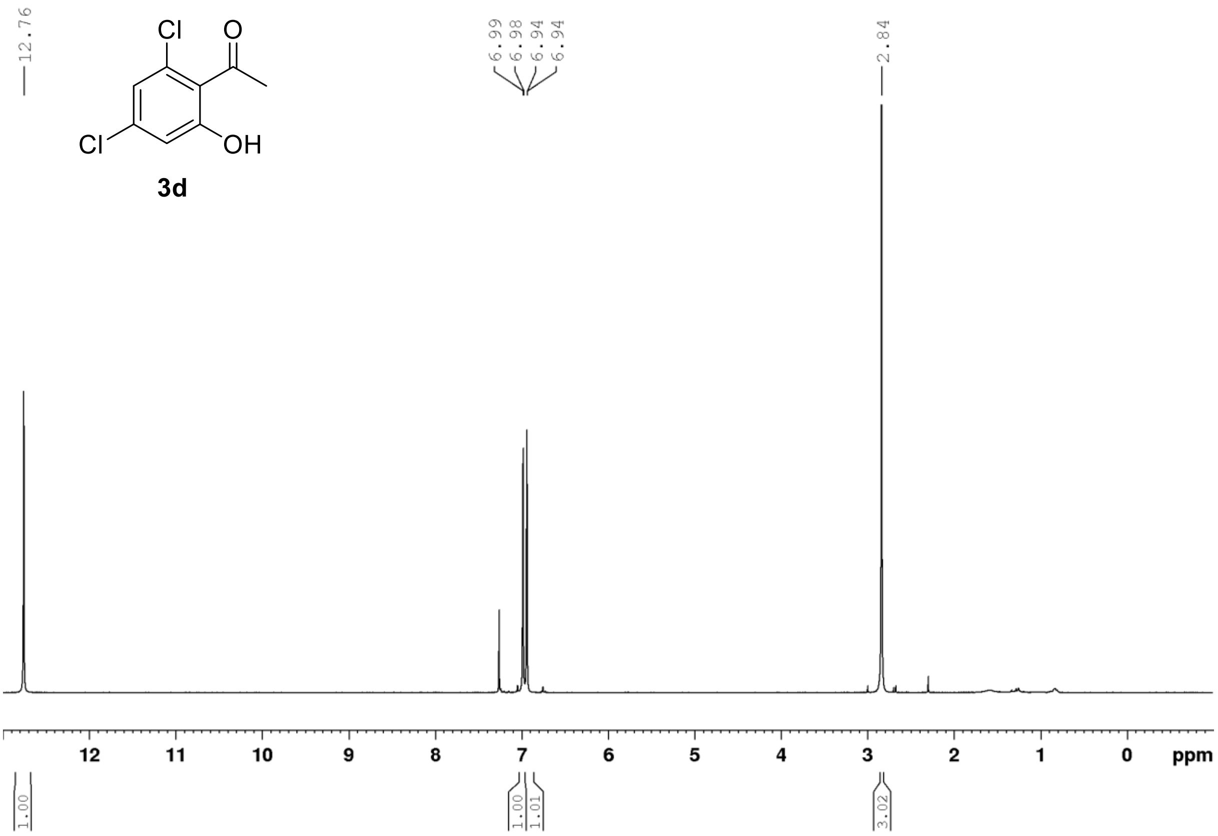

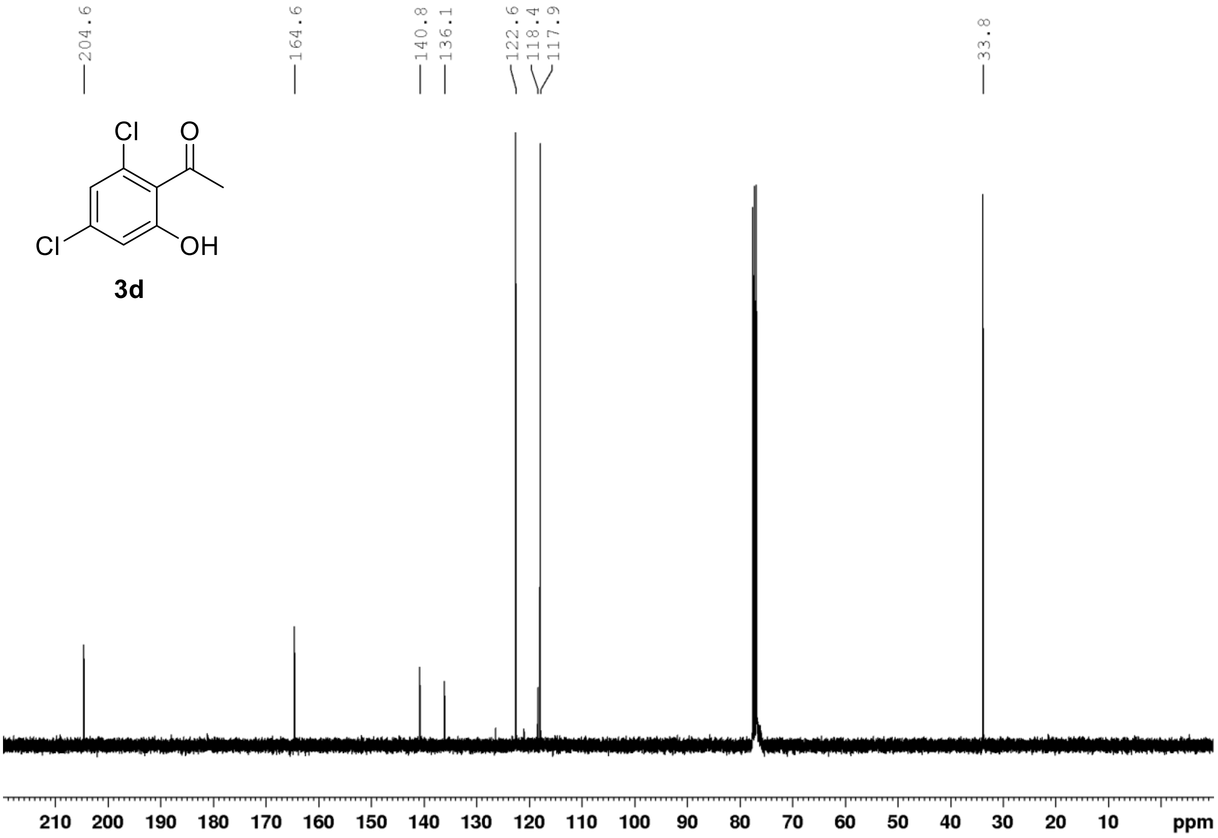

1-(2,4-Bromo-6-hydroxyphenyl)ethan-1-one (3e)

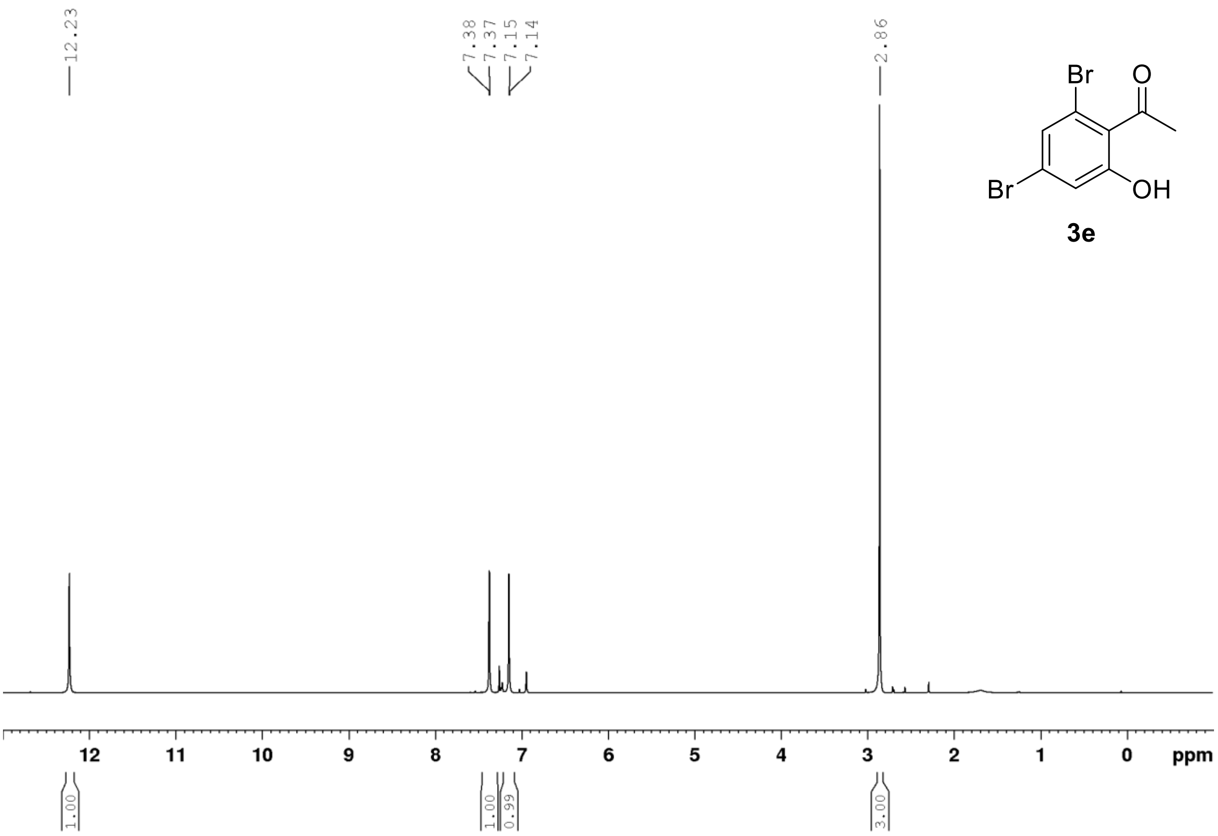

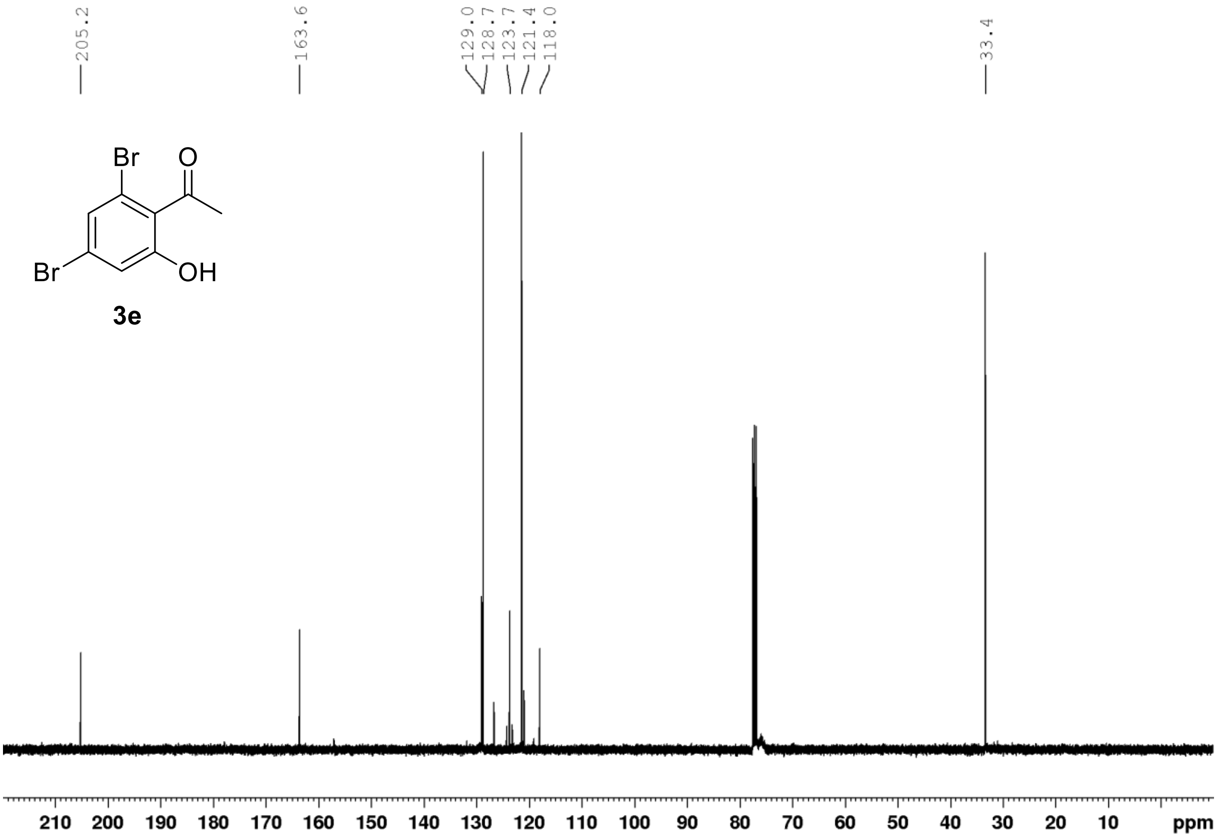

1-(4-Bromo-2-chloro-6-hydroxyphenyl)ethan-1-one (3f)

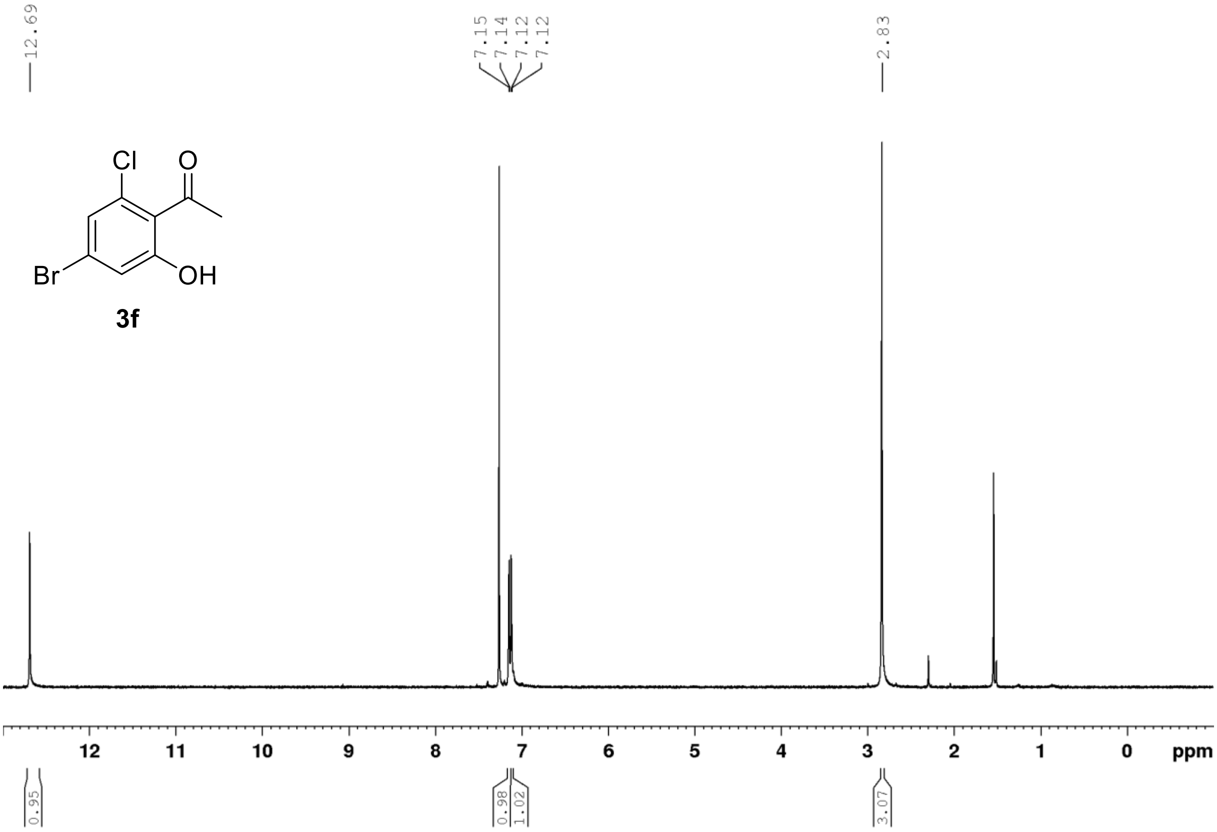

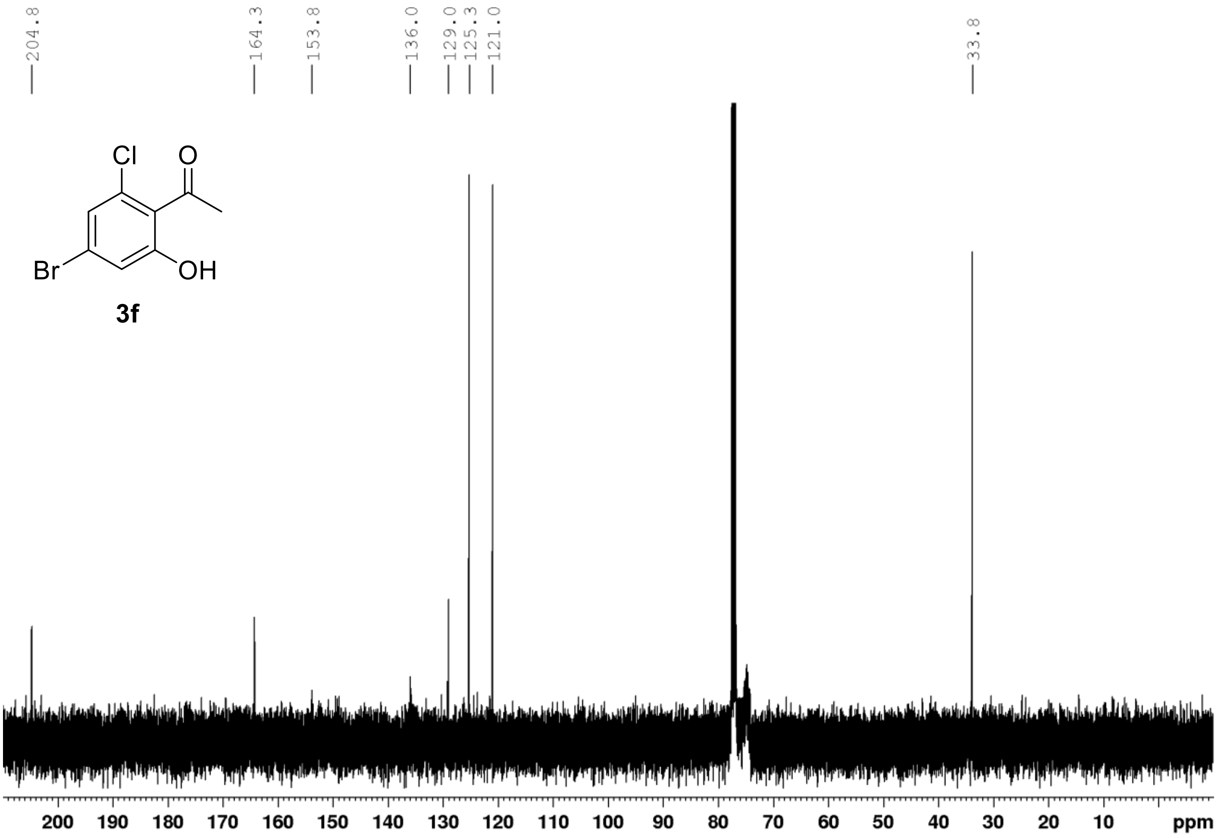

1-(2-Bromo-4-chloro-6-hydroxyphenyl)ethan-1-one (3g)

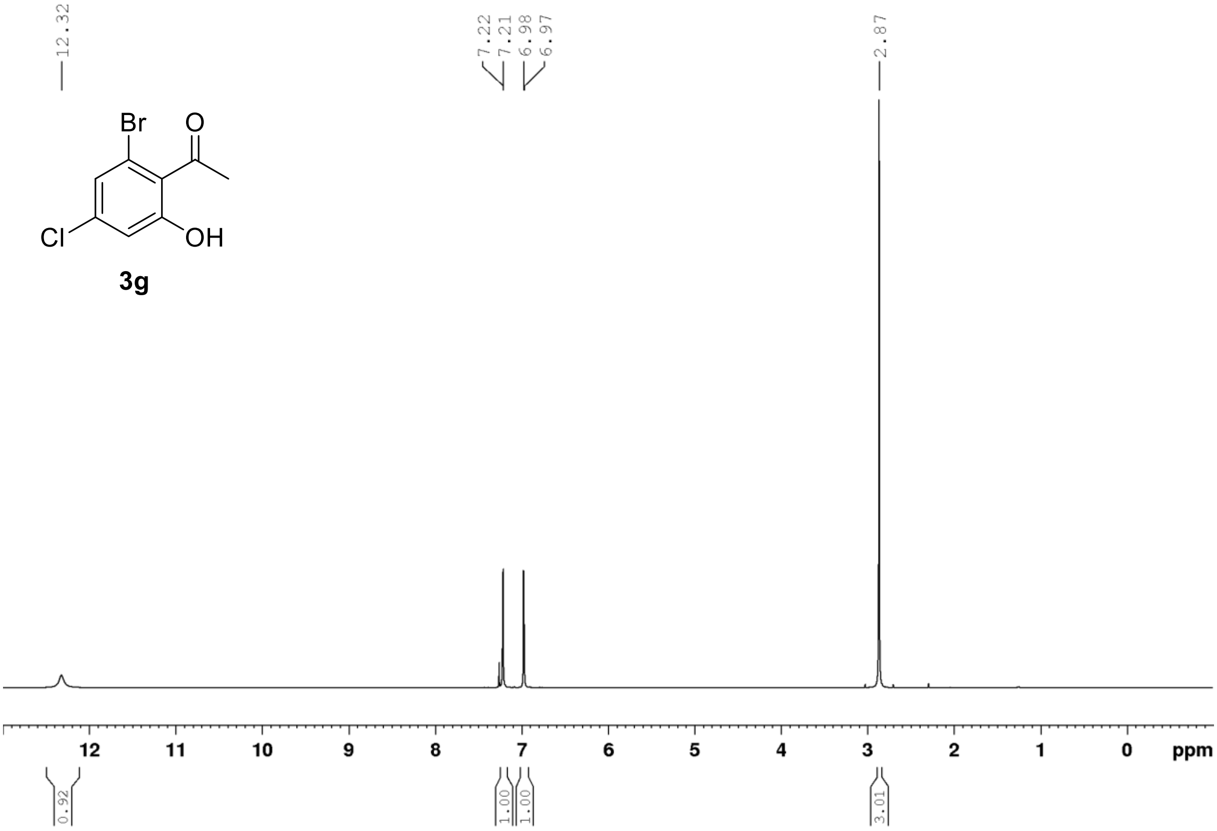

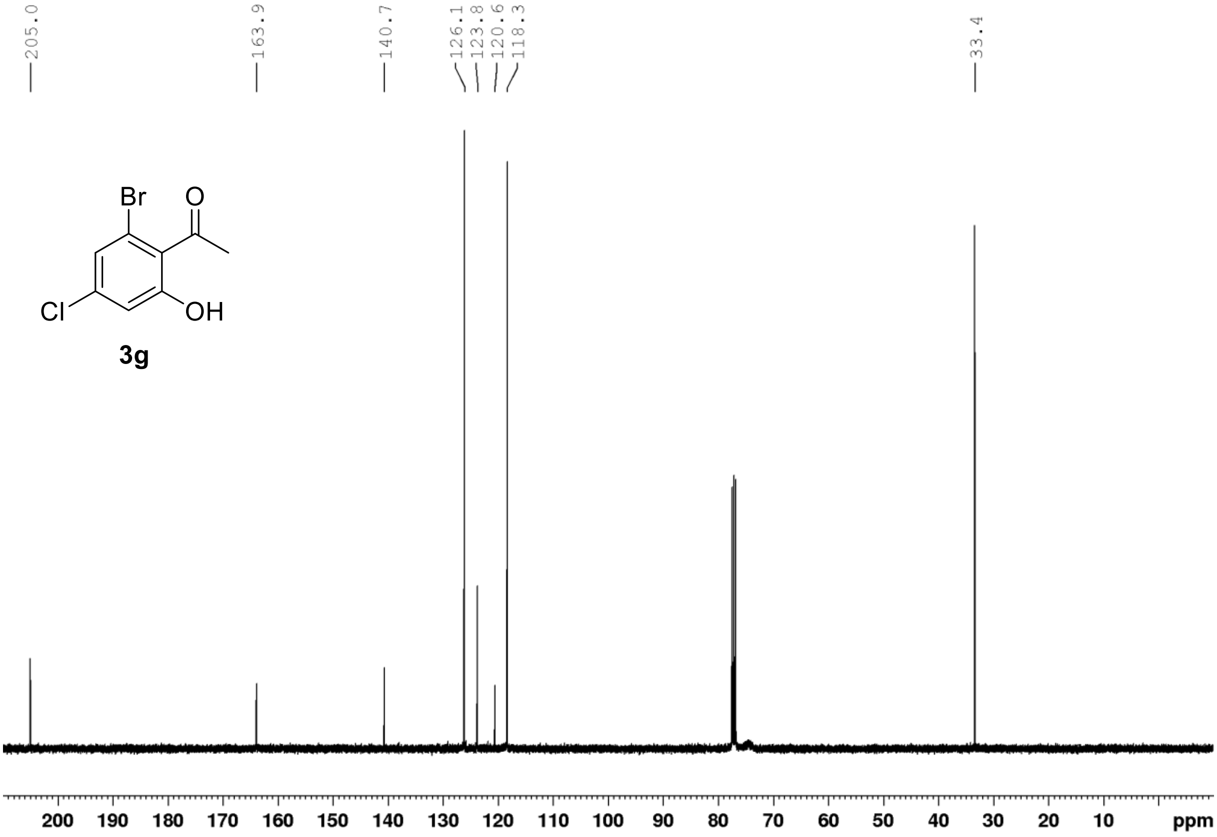

1-(2-Fluoro-6-hydroxy-4-methoxyphenyl)ethan-1-one (3h)

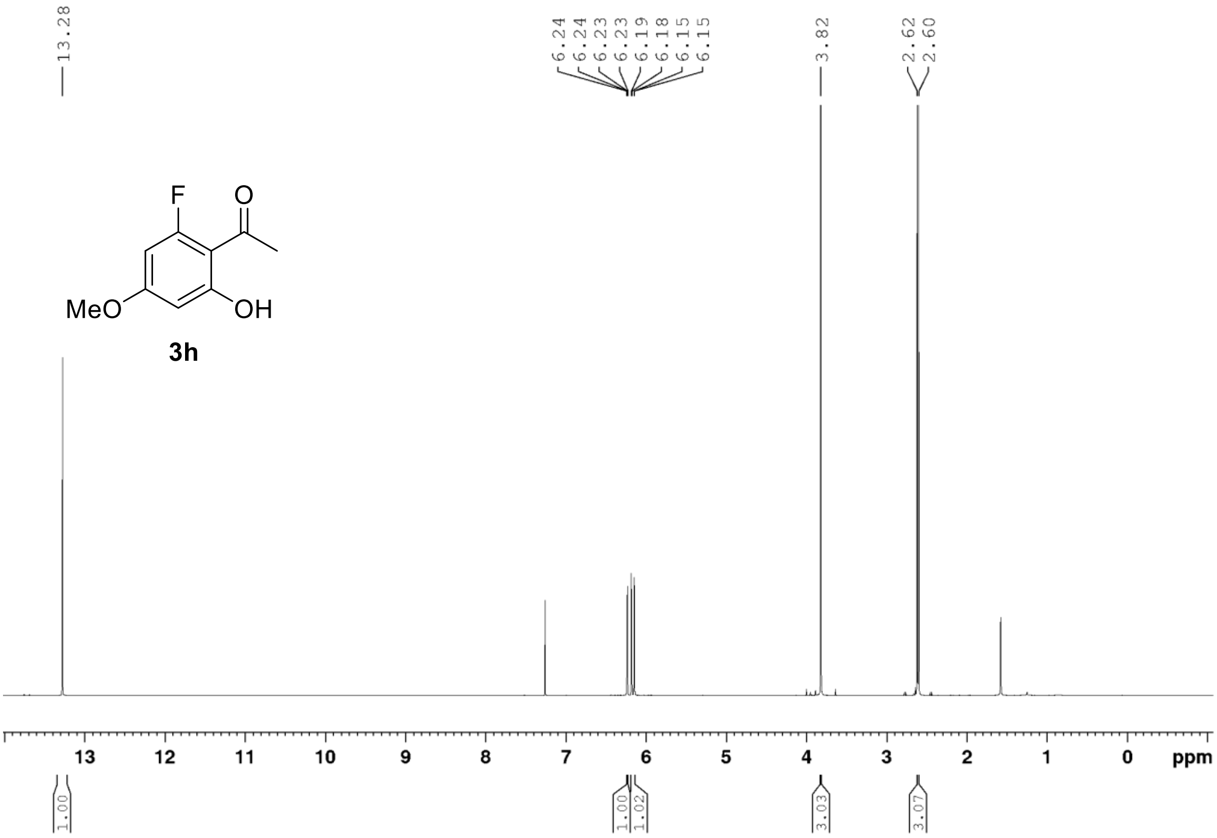

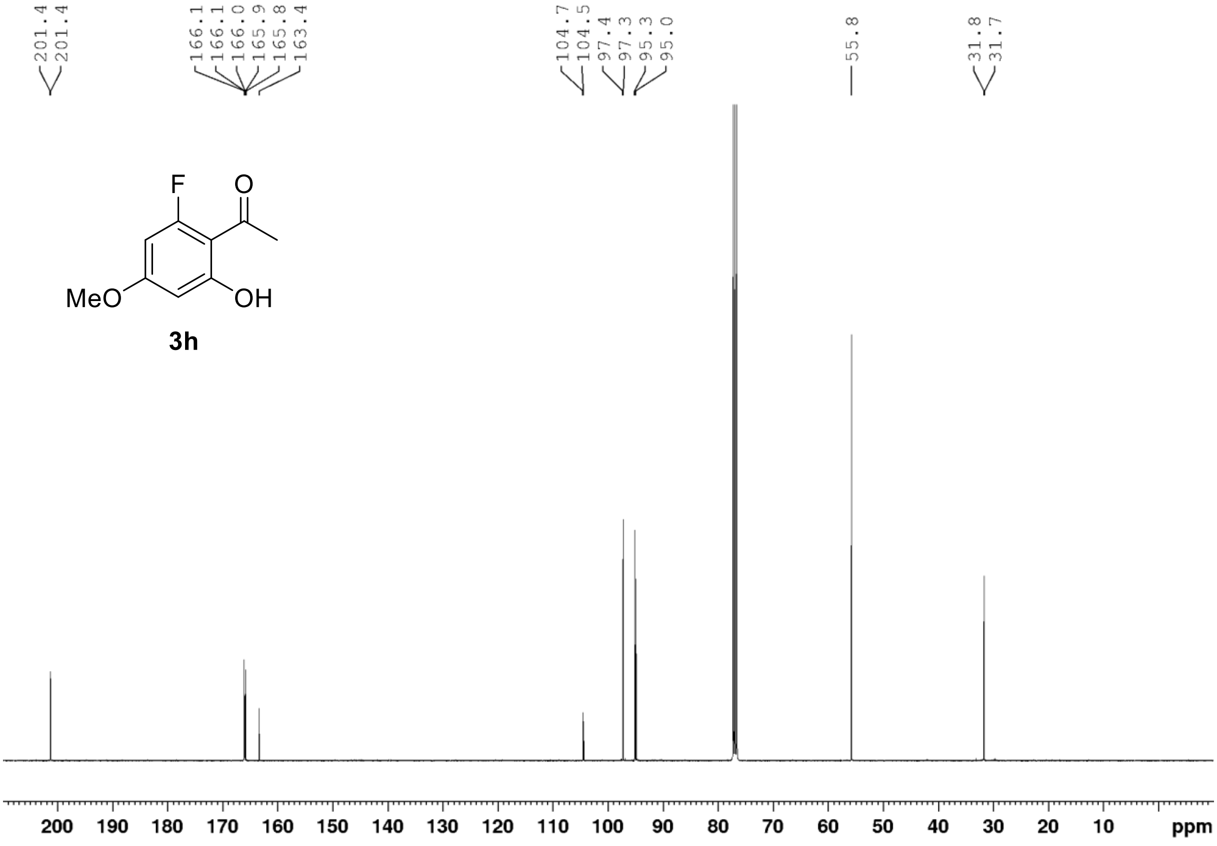

1-(4-Fluoro-2-hydroxy-6-methoxyphenyl)ethan-1-one (3i)

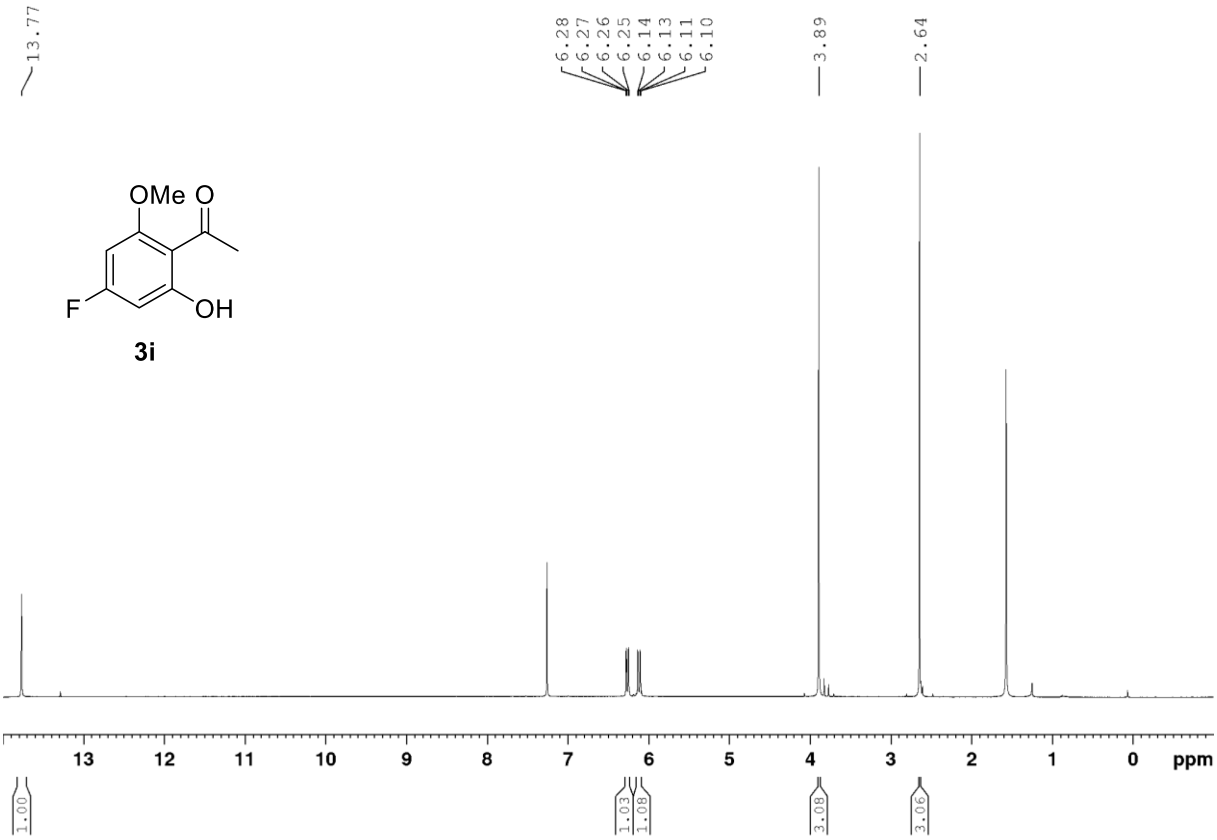

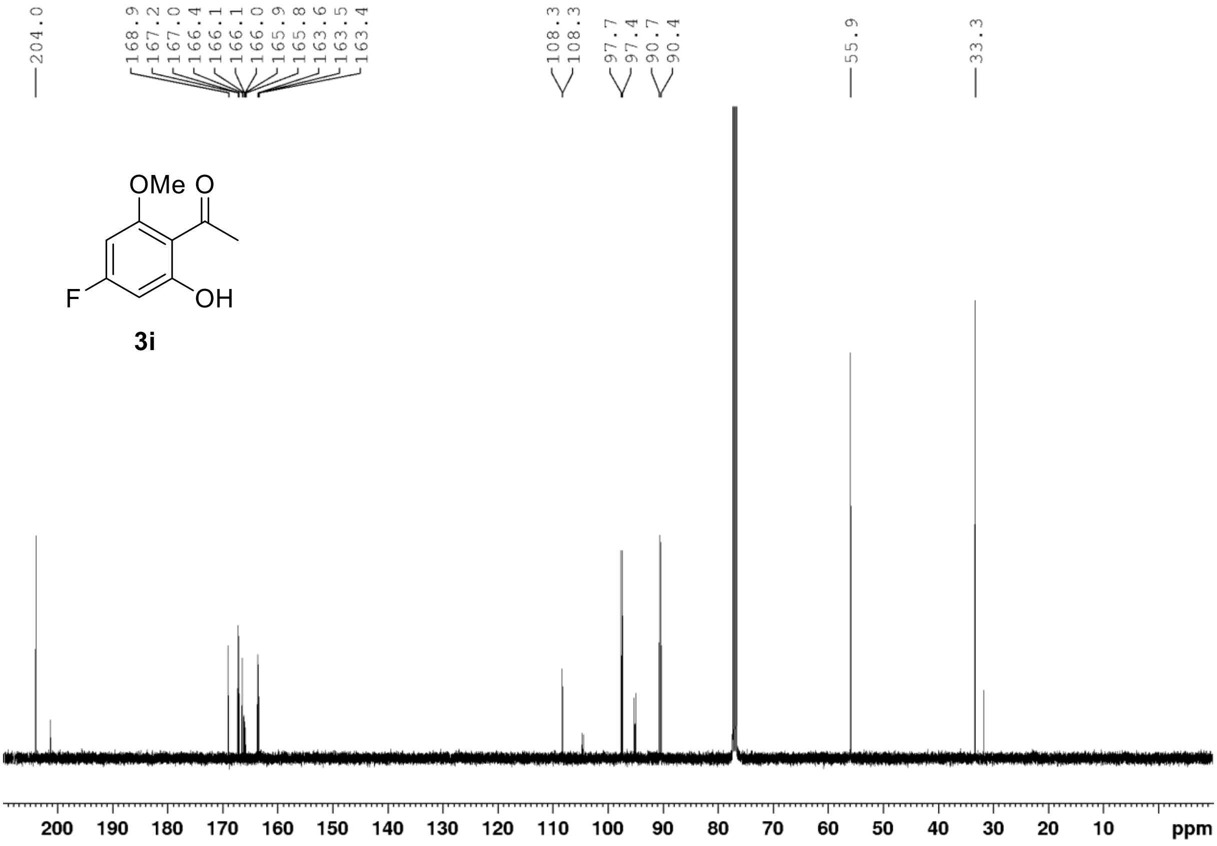

1-(2-Chloro-4,6-dimethoxyphenyl)ethan-1-one (SI-10)

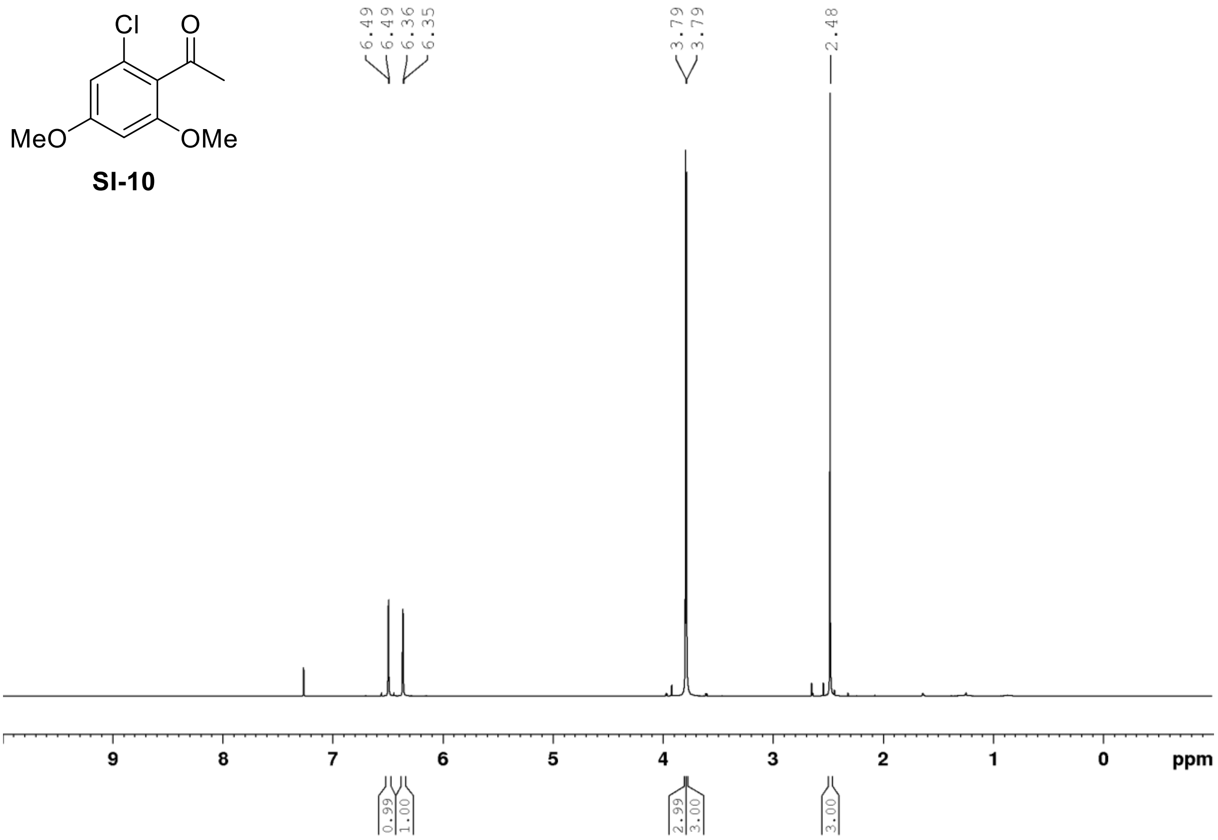

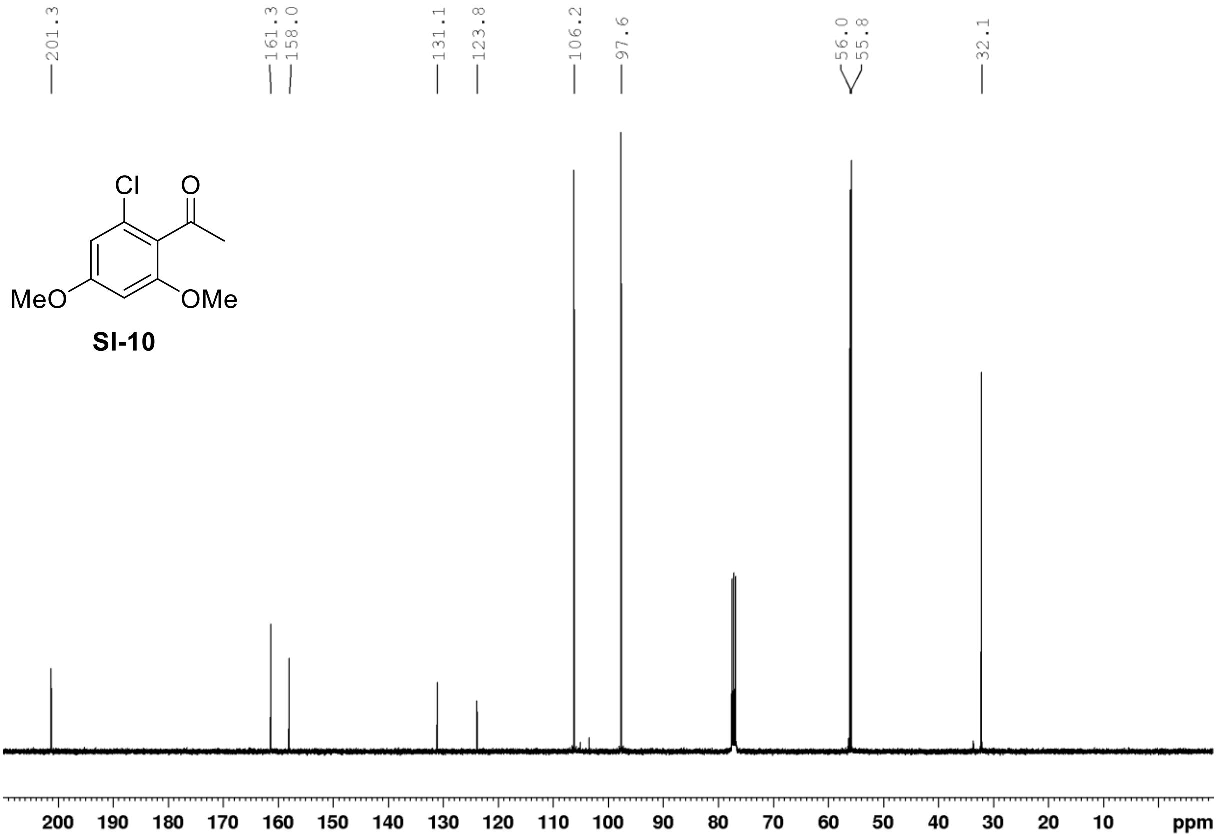

1-(4-Chloro-2,6-dimethoxyphenyl)ethan-1-one (SI-11)

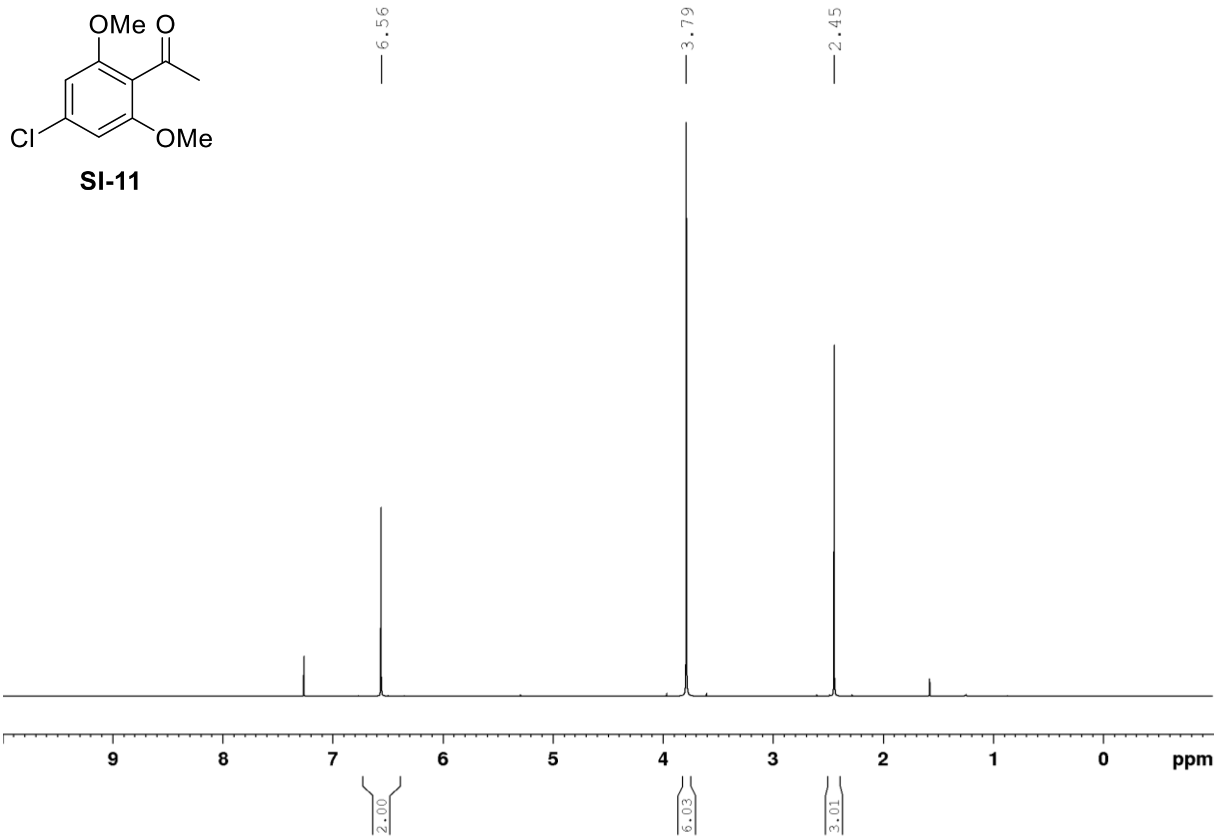

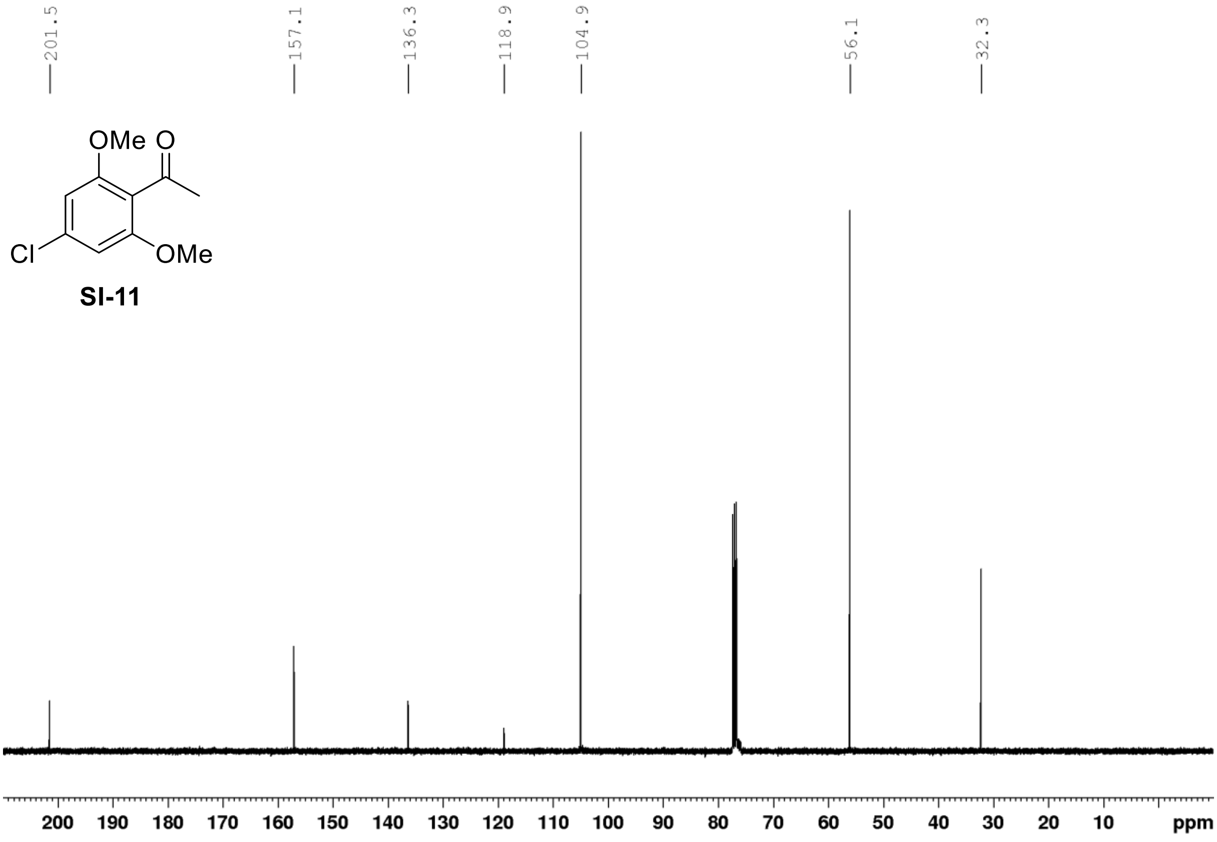

1-(2-Chloro-6-hydroxy-4-methoxyphenyl)ethan-1-one (3j)

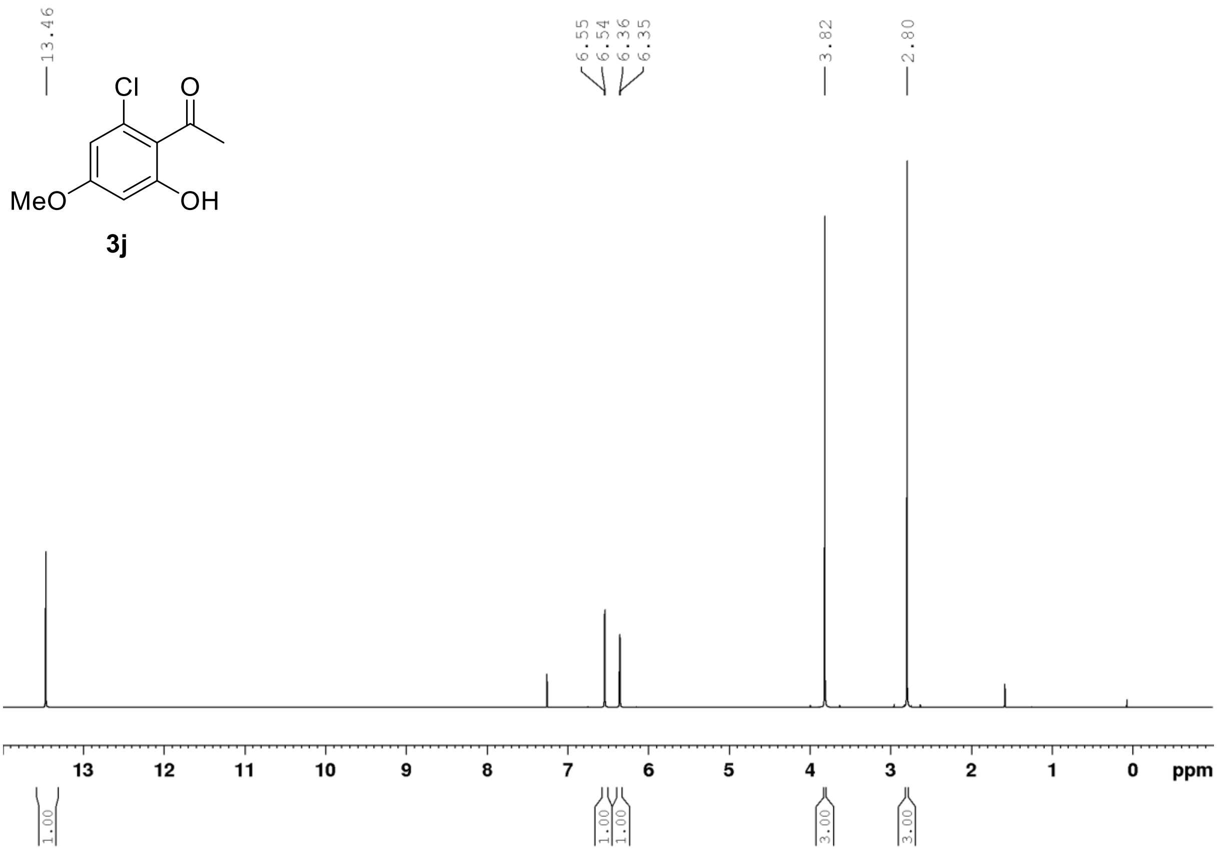

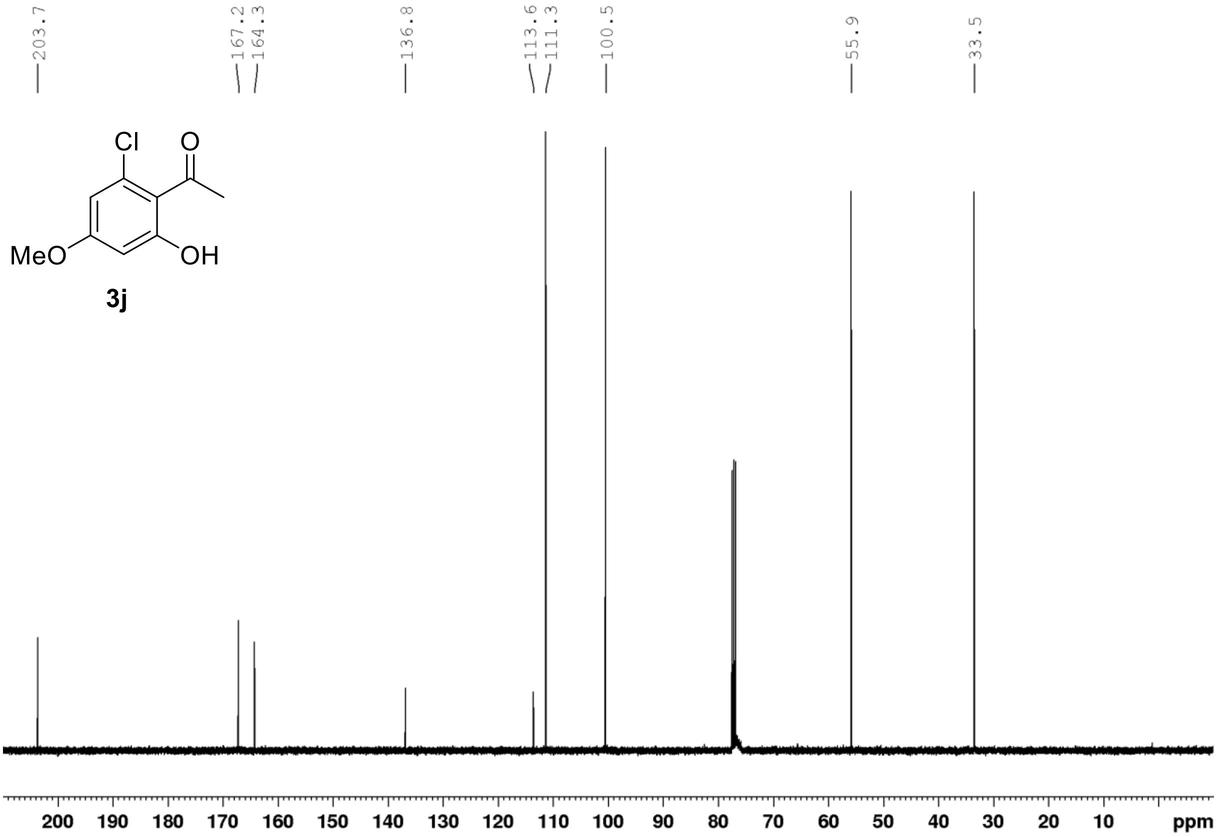

1-(4-Chloro-2-hydroxy-6-methoxyphenyl)ethan-1-one (3k)

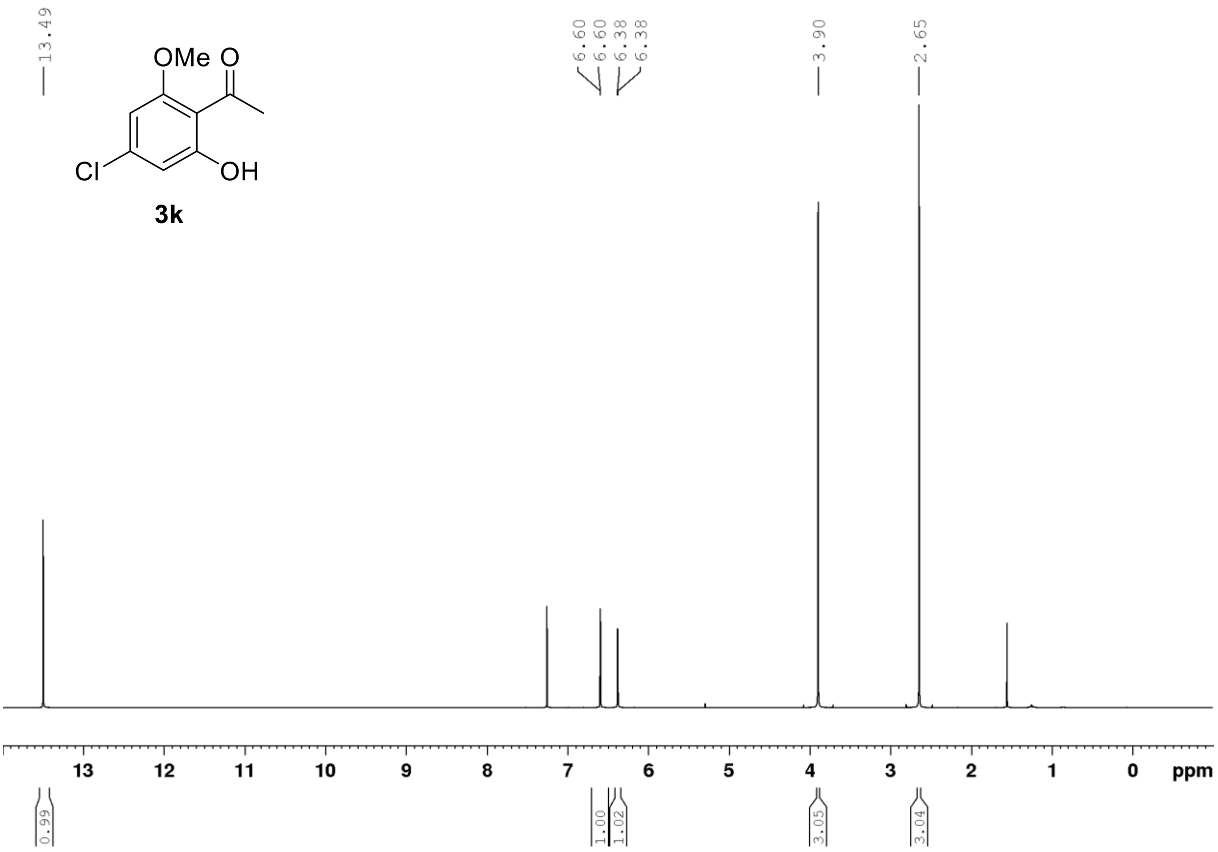

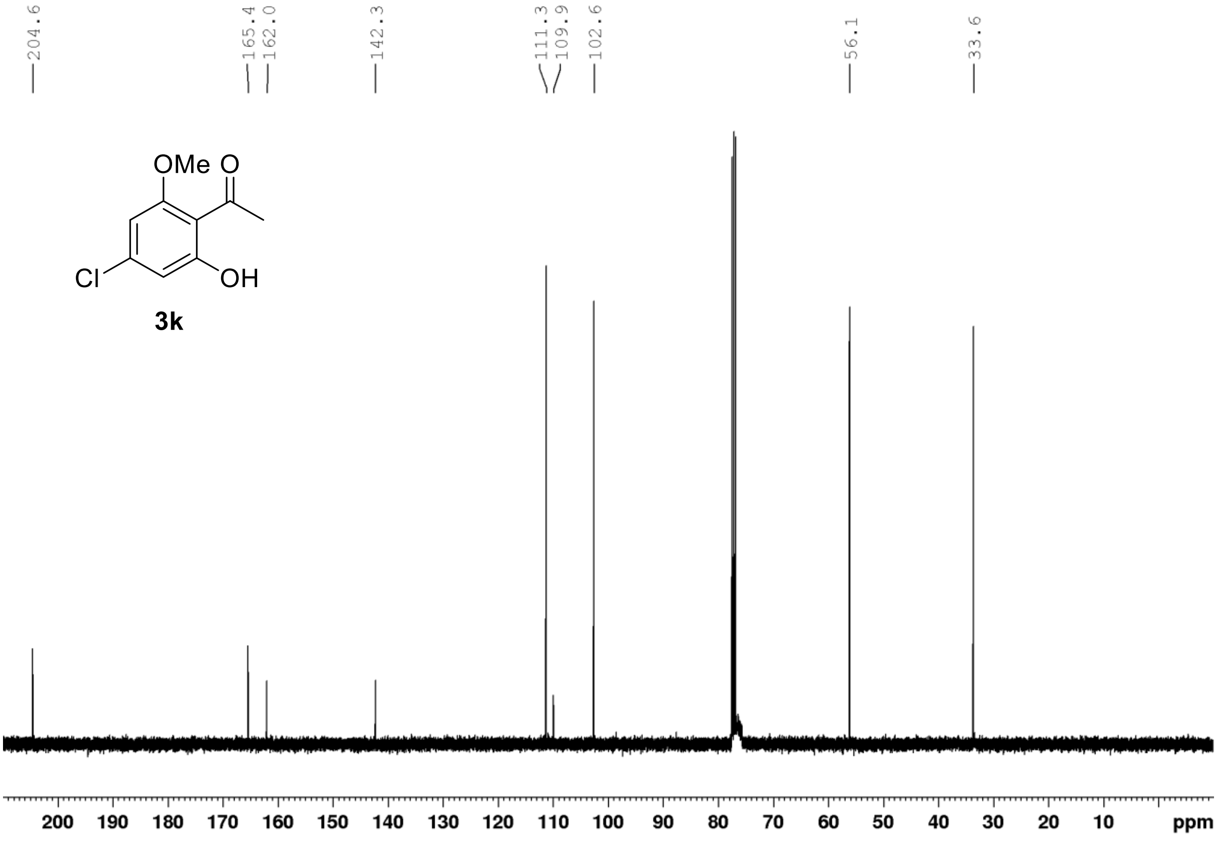

1-(2-Bromo-4,6-dimethoxyphenyl)ethan-1-one (SI-12)

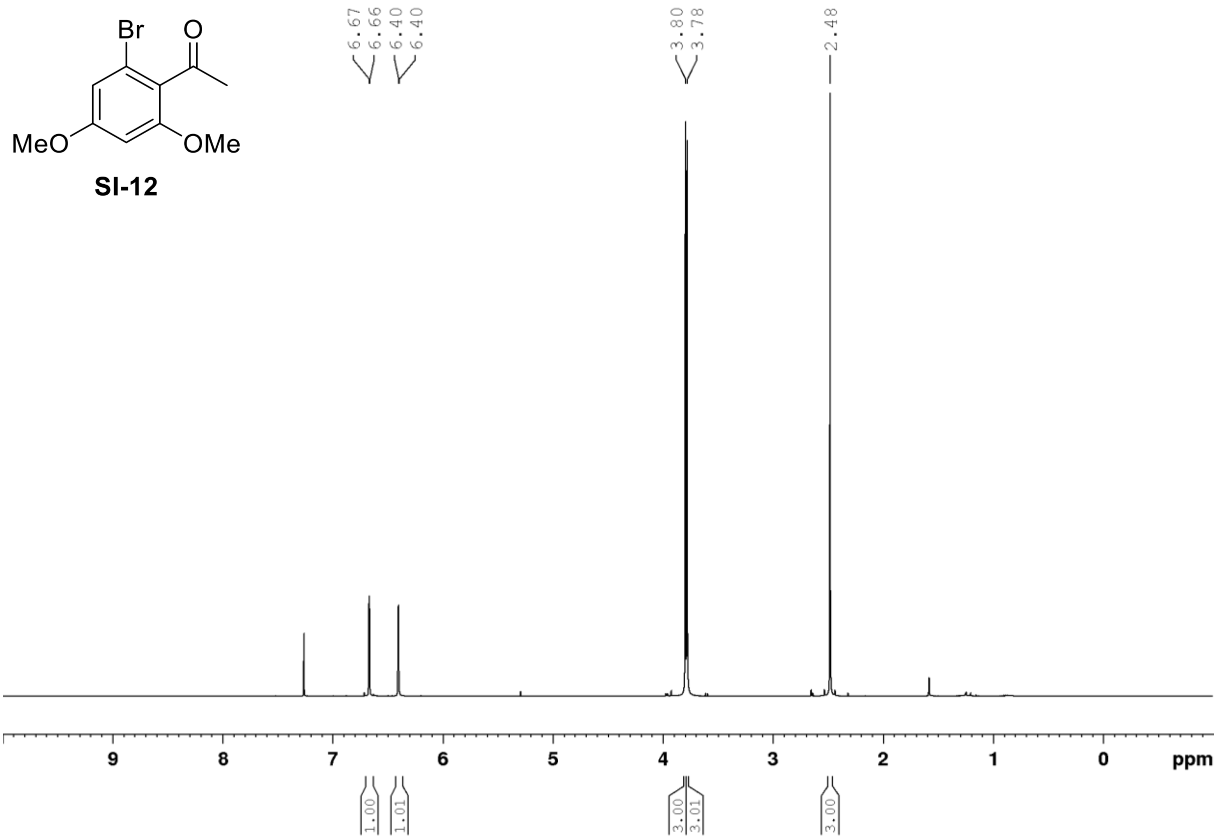

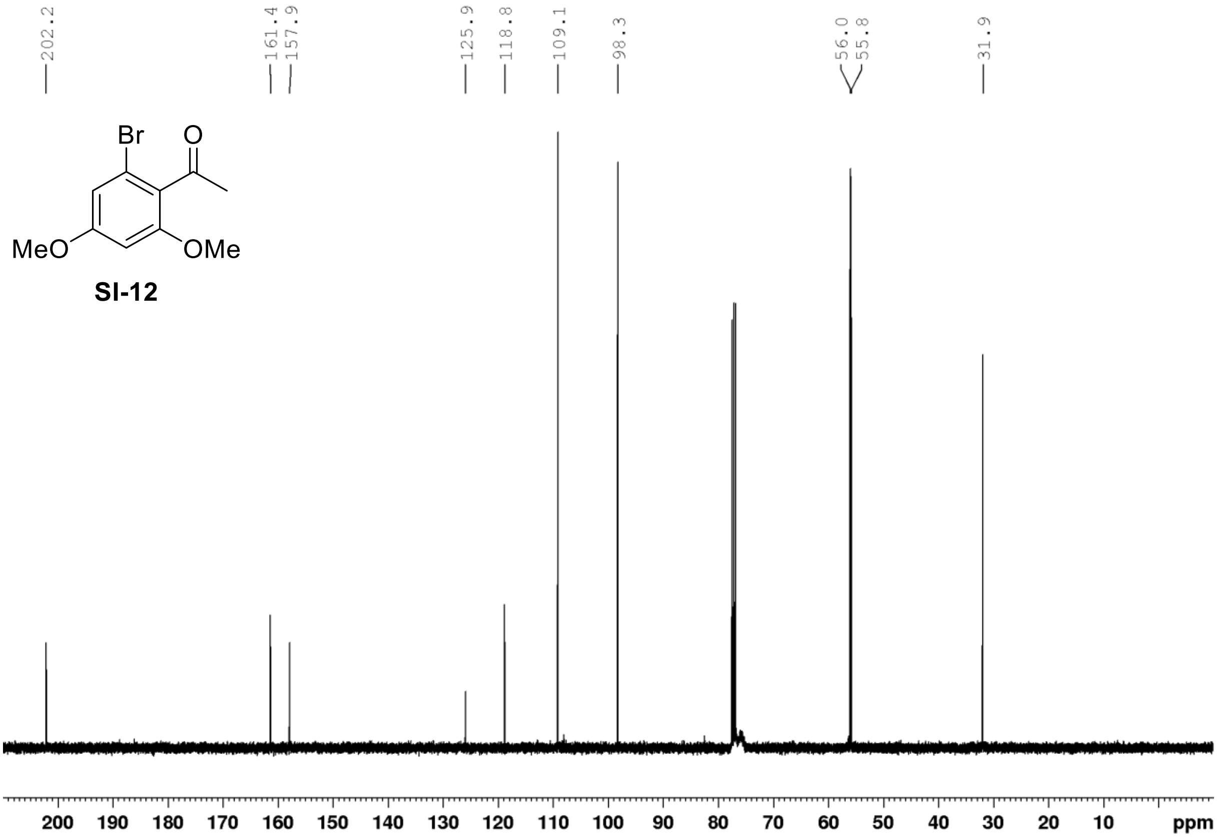

1-(4-Bromo-2,6-dimethoxyphenyl)ethan-1-one (SI-13)

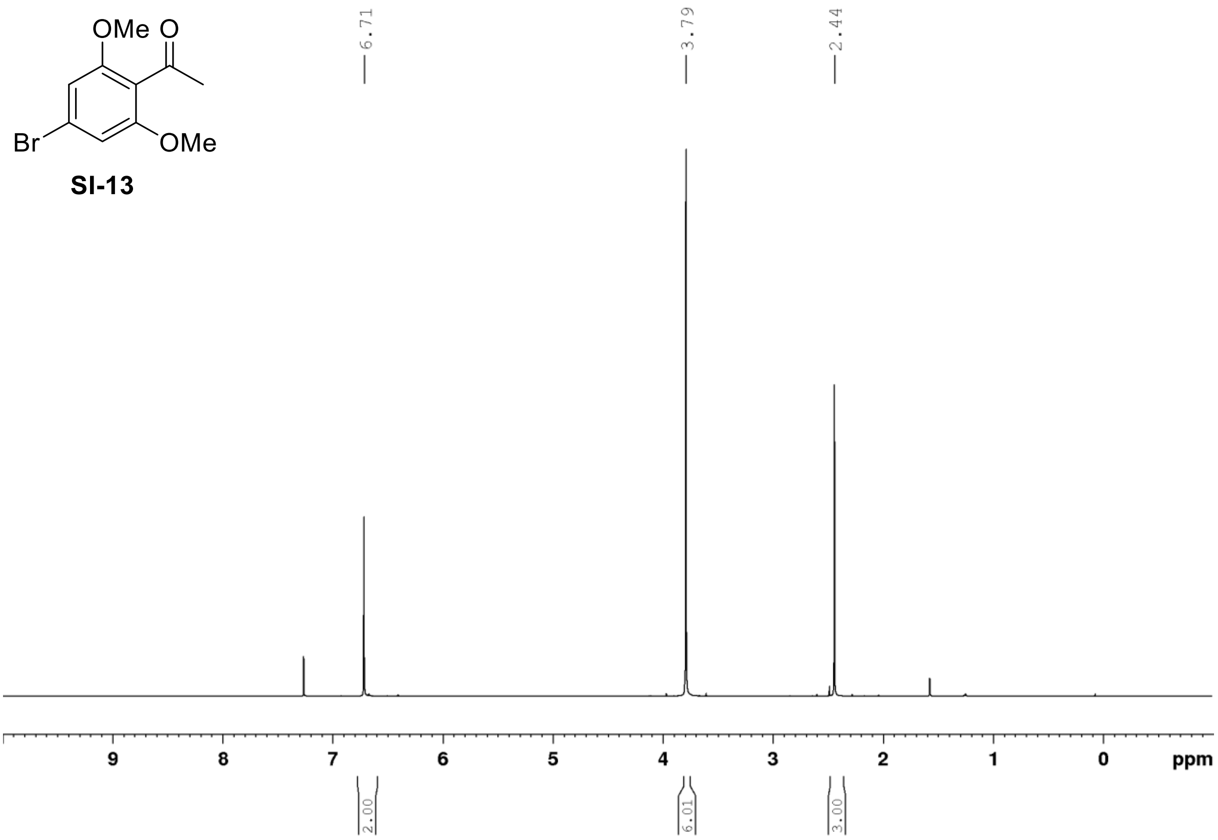

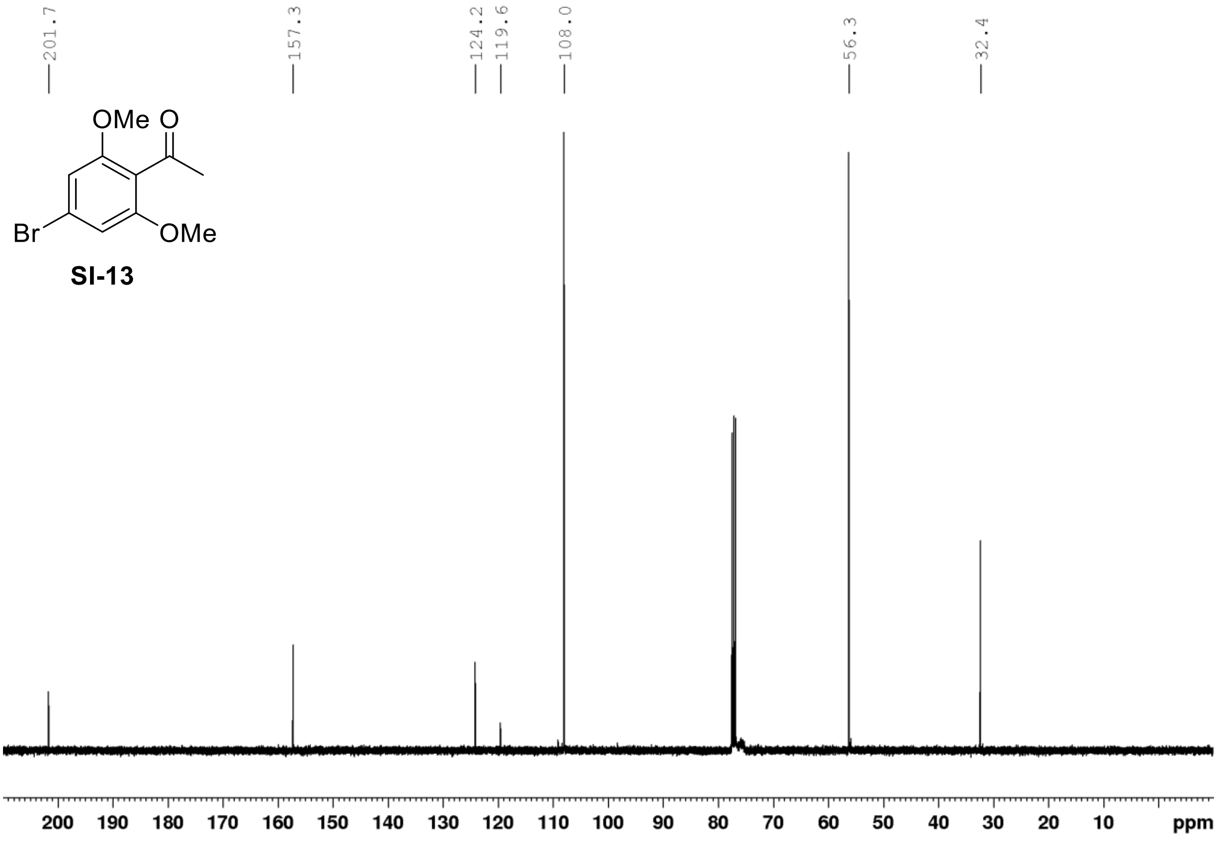

1-(2-Bromo-6-hydroxy-4-methoxyphenyl)ethan-1-one (3l)

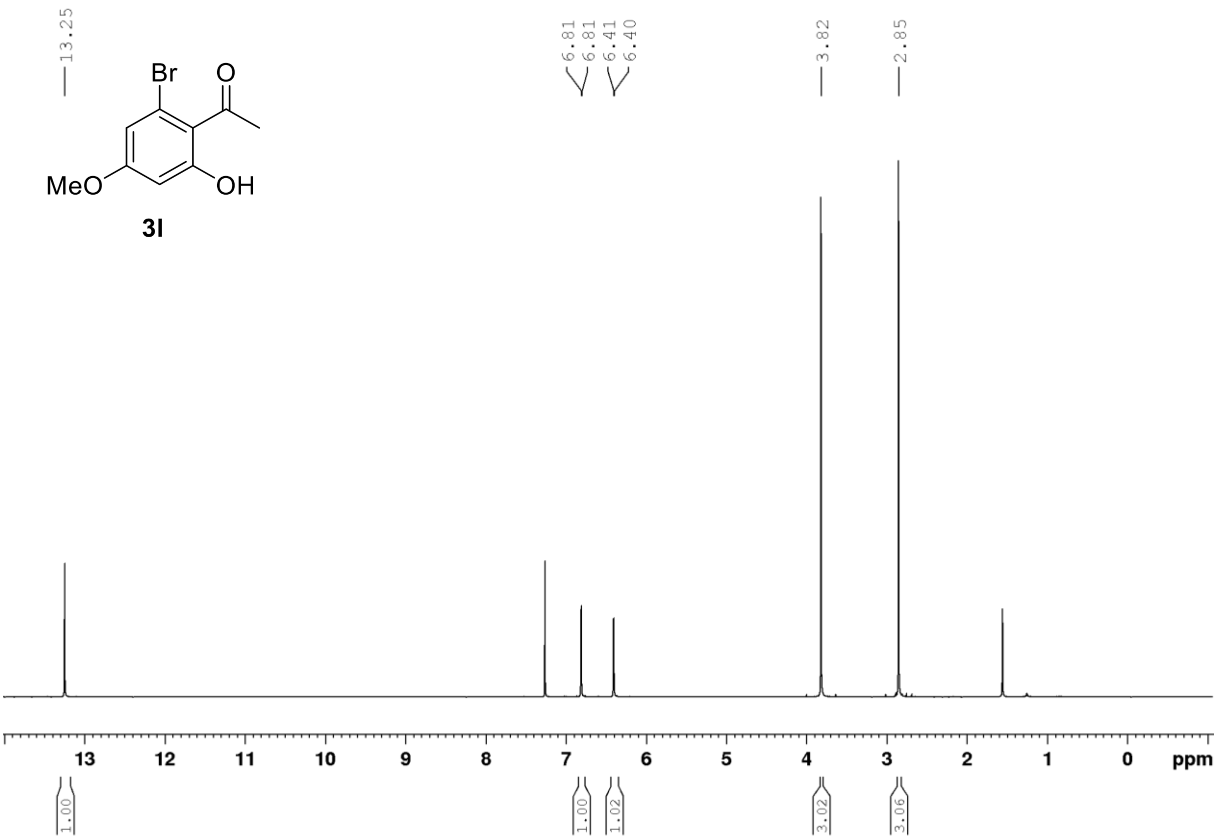

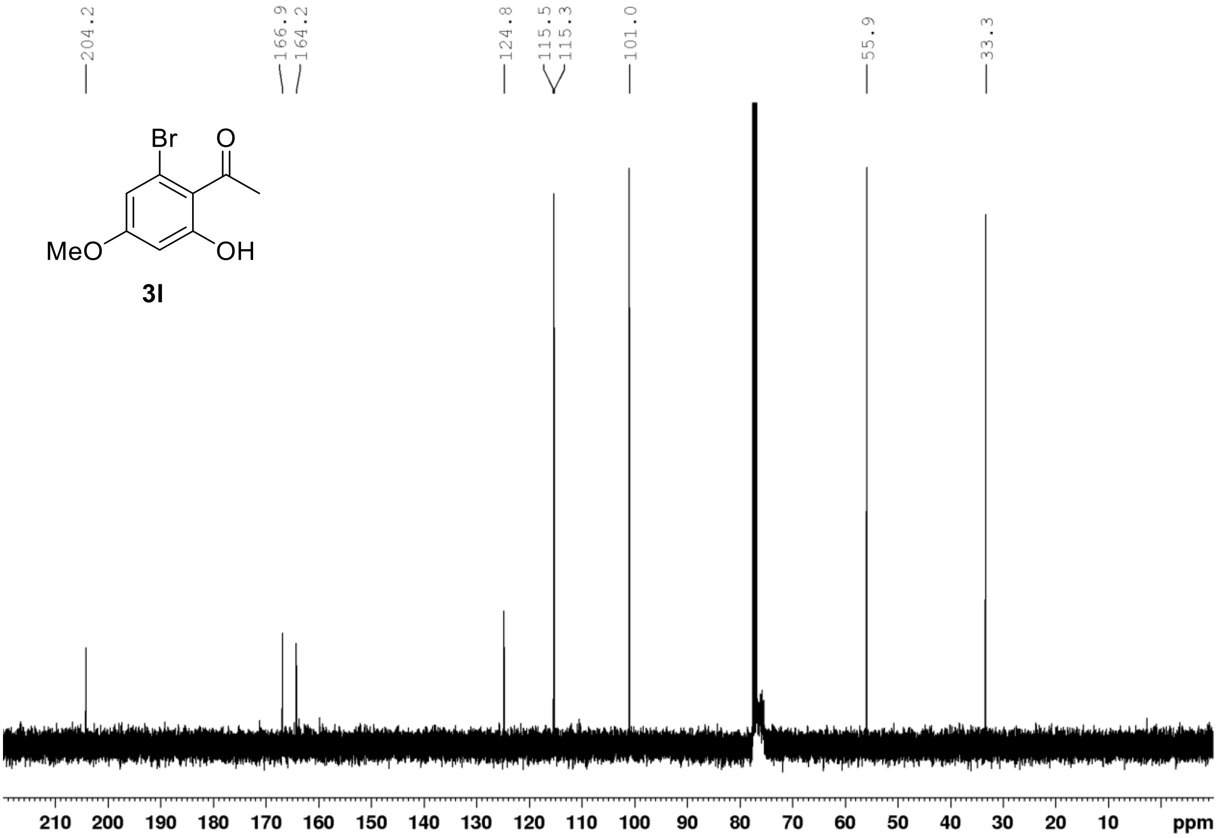

1-(4-Bromo-2-hydroxy-6-methoxyphenyl)ethan-1-one (3m)

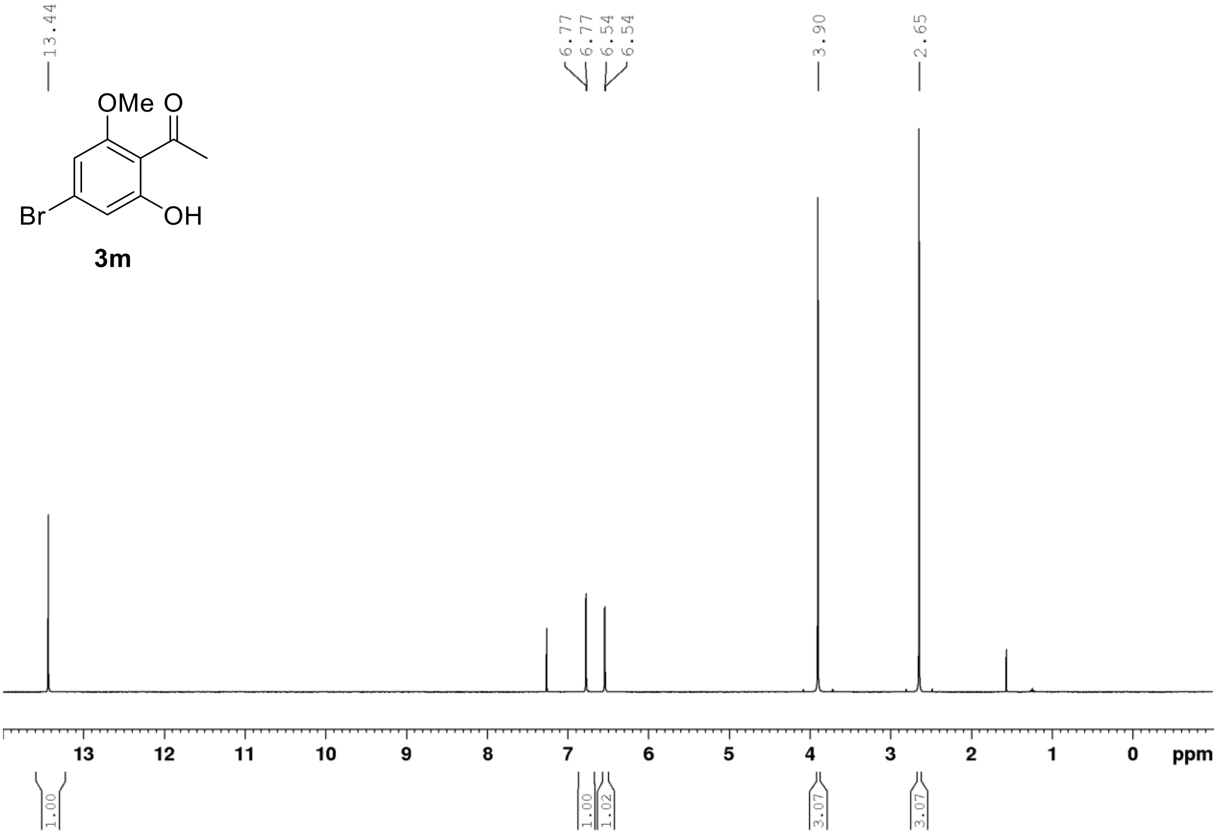

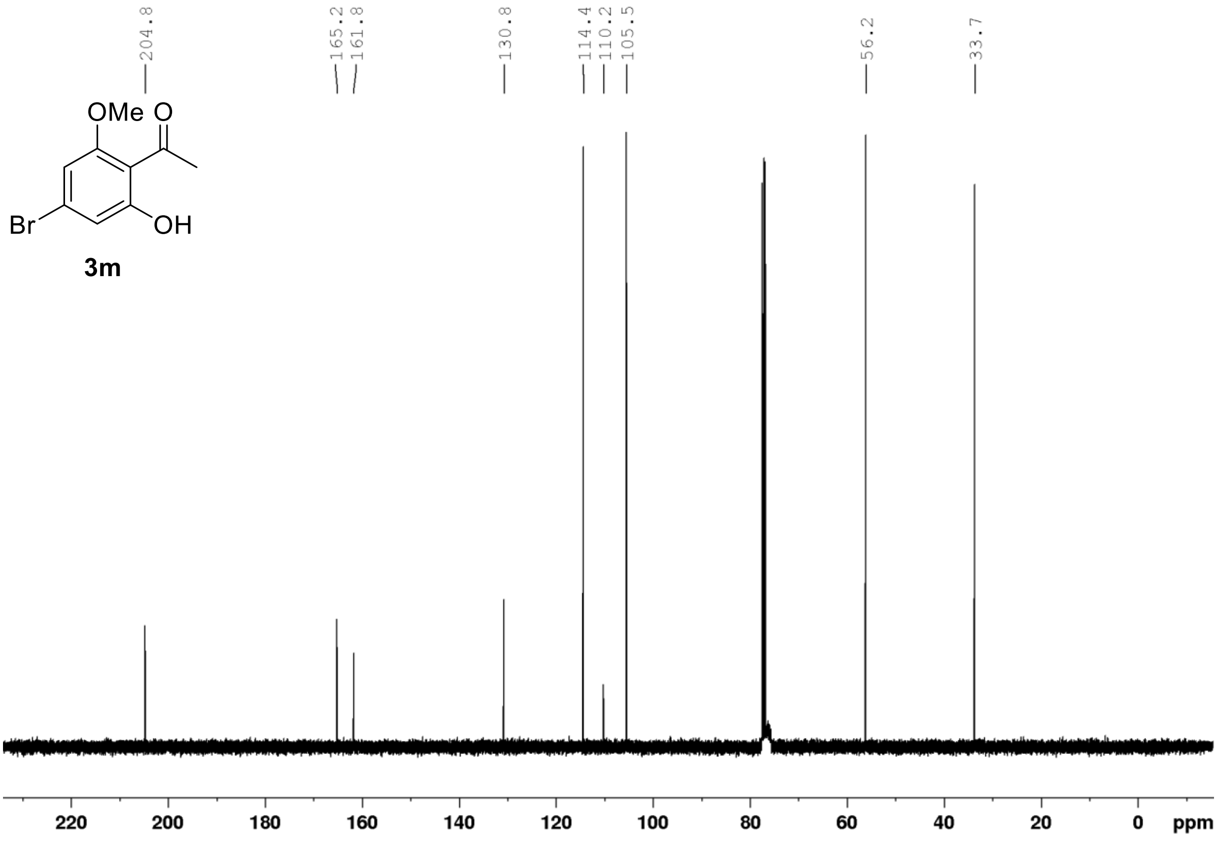

1-(2-Fluoro-4,6-dihydroxyphenyl)ethan-1-one (SI-15)

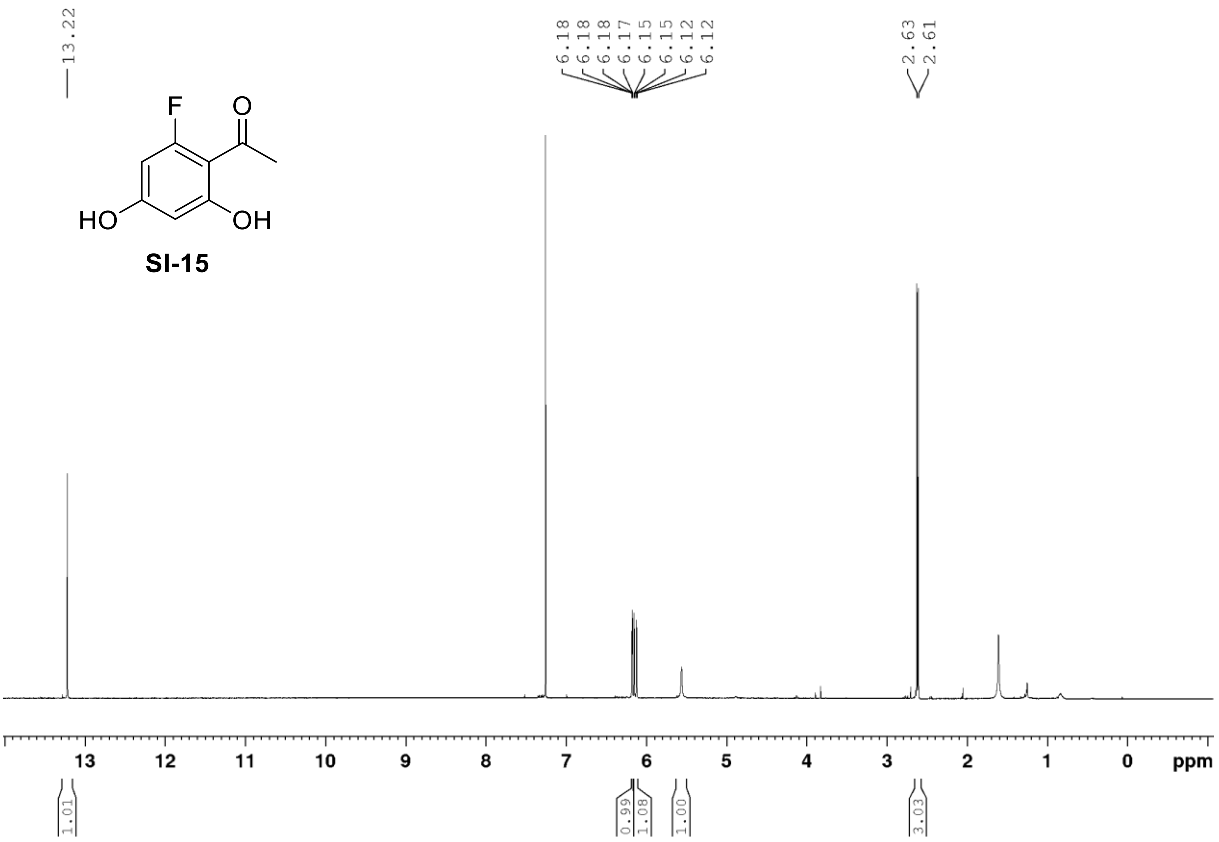

1-(2-Fluoro-6-hydroxy-4-(methoxymethoxy)phenyl)ethan-1-one (3n)

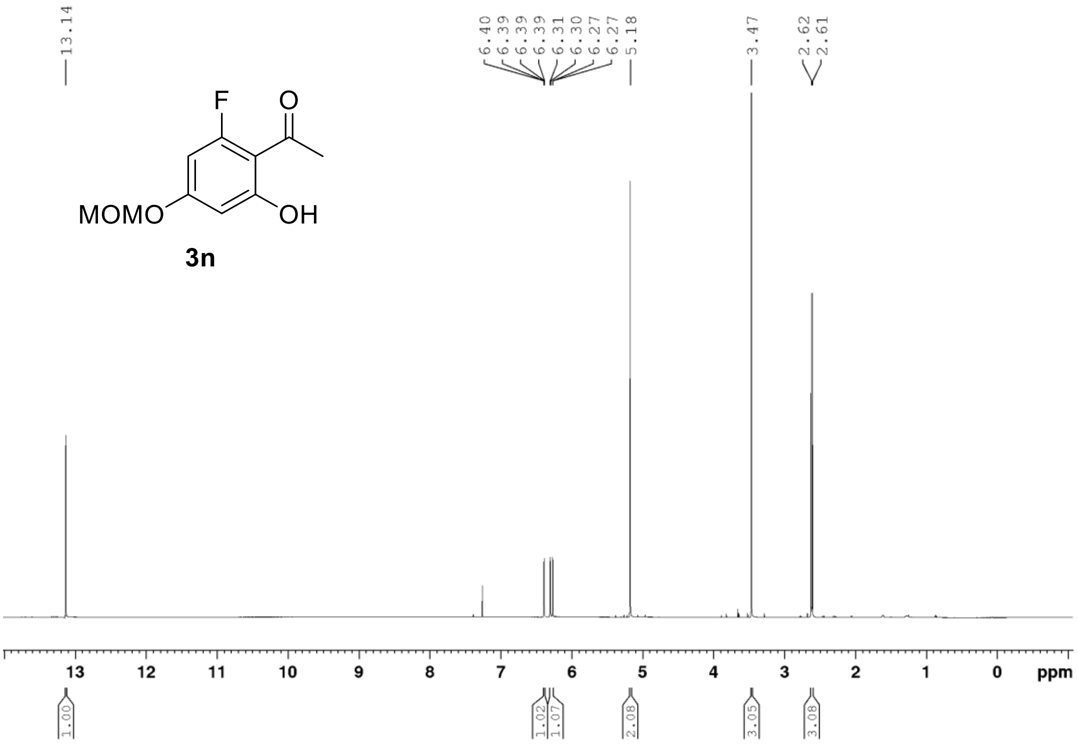

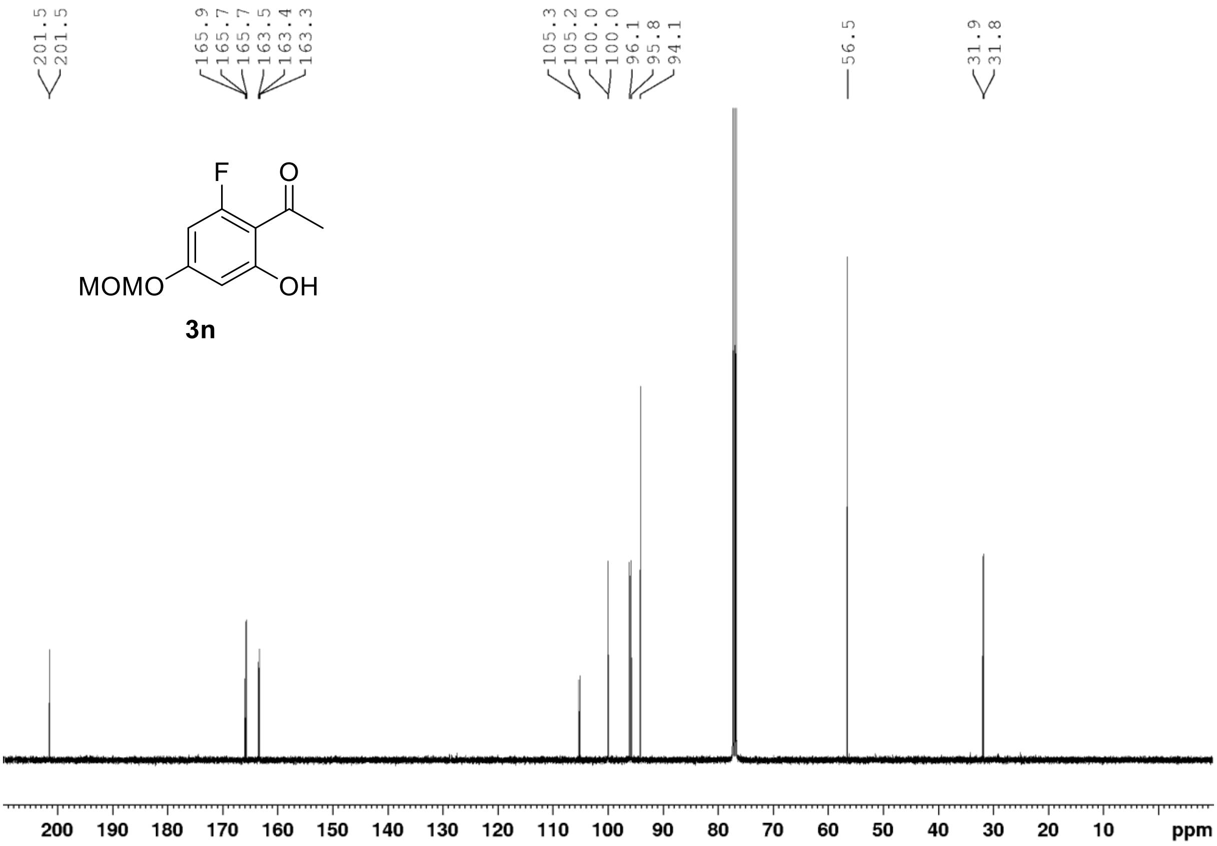

4.2. NMR spectroscopic data for the synthesis of 9a

2-Hydroxy-1-(2-hydroxy-4,6-dimethoxyphenyl)ethan-1-one (4a)

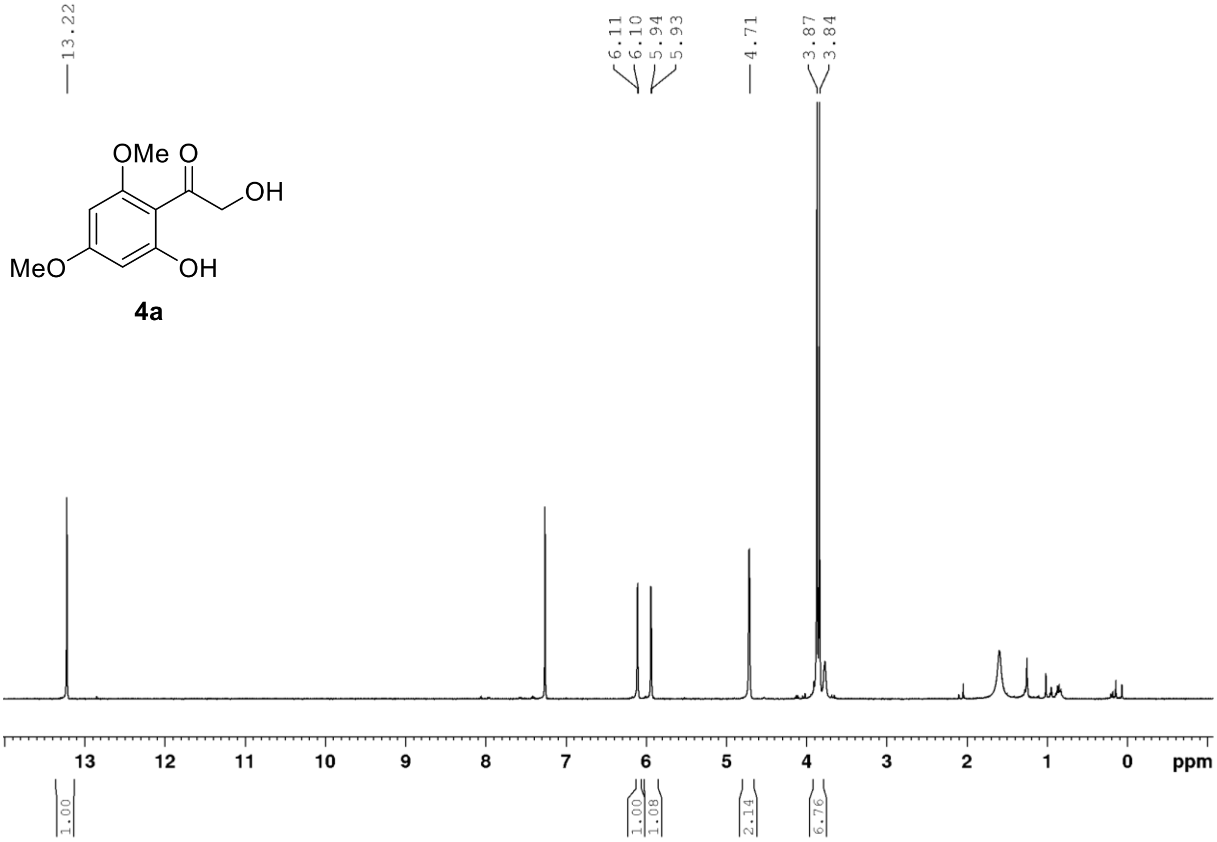

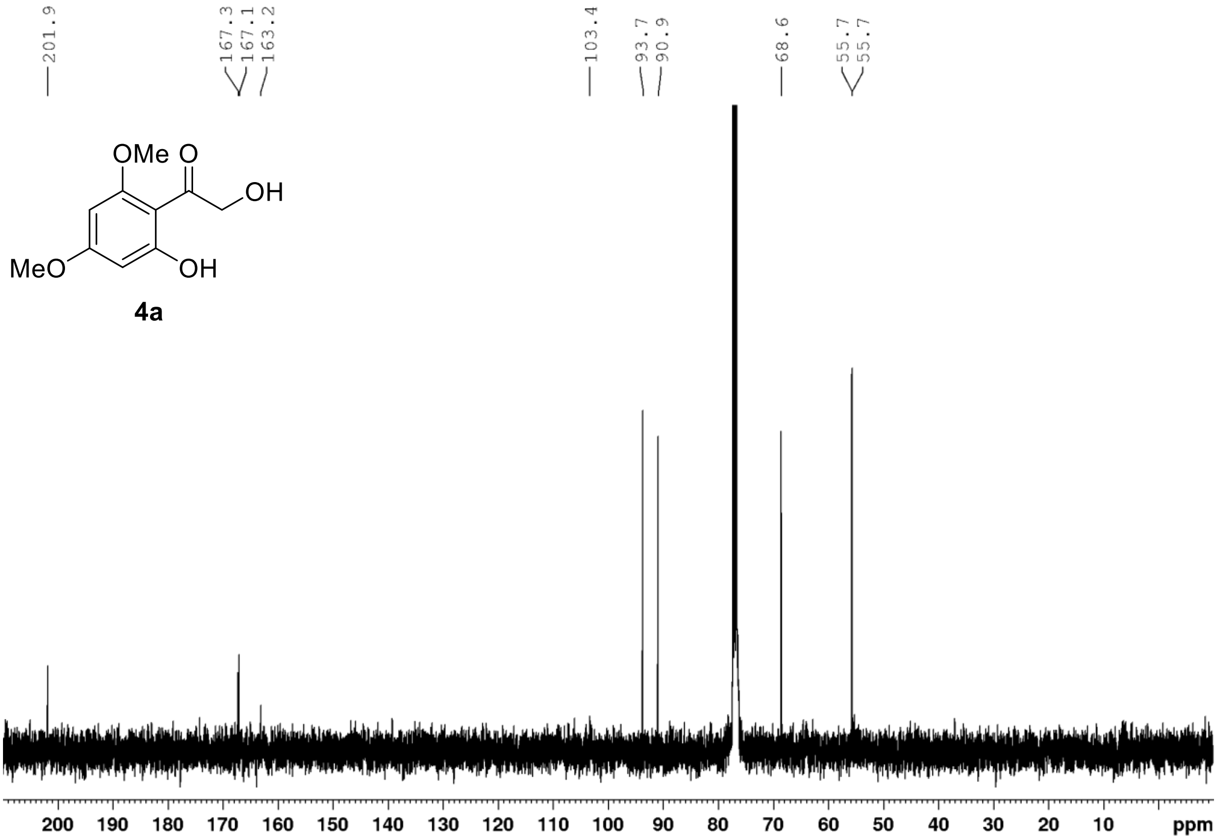

2-(2-((4-Chlorobenzoyl)oxy)-4,6-dimethoxyphenyl)-2-oxoethyl 4-chlorobenzoate (5aa)

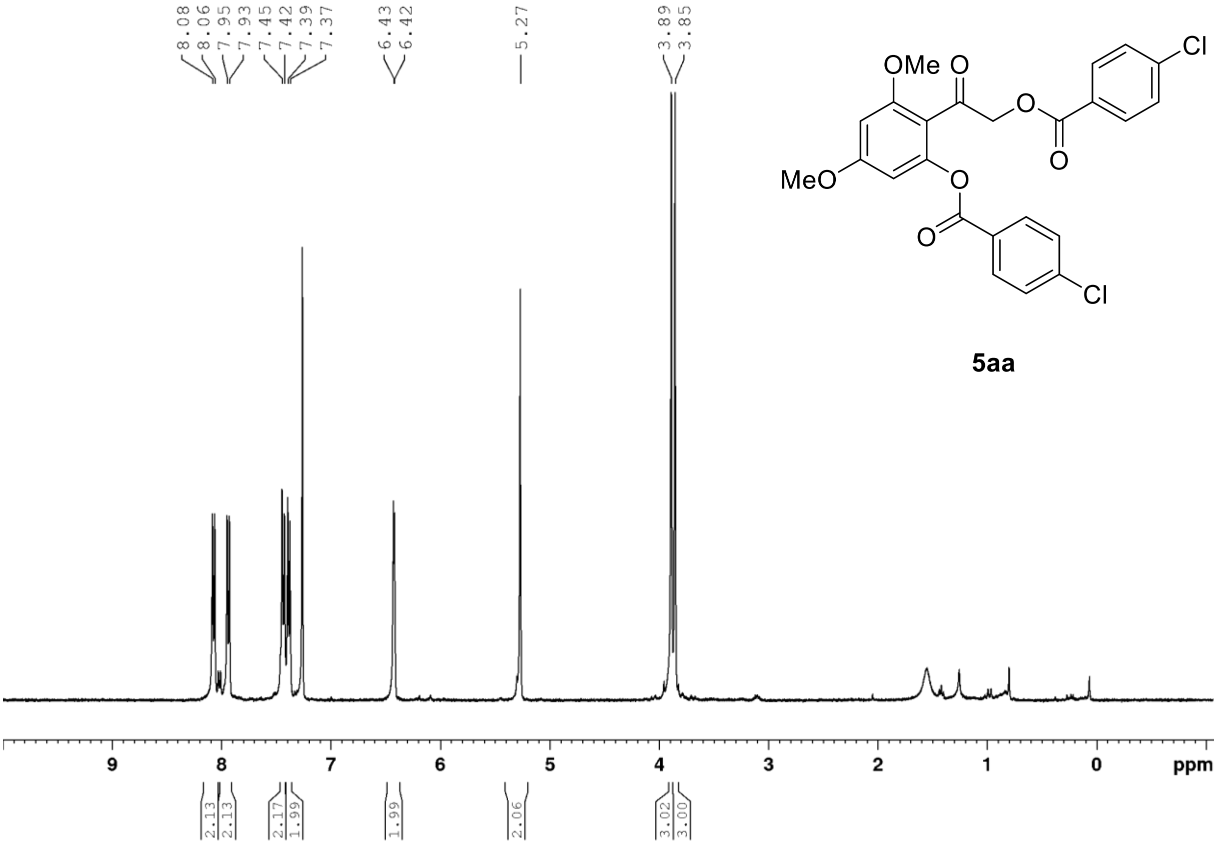

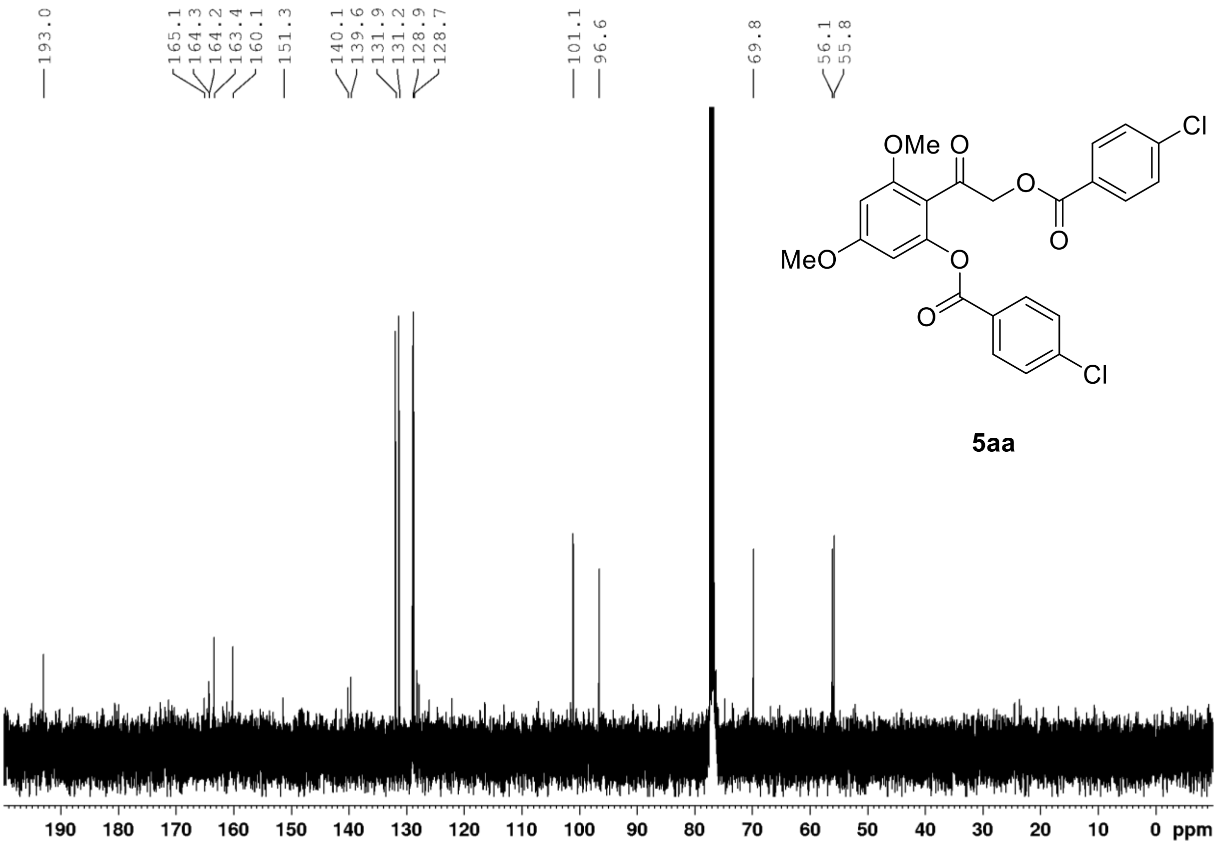

1-(4-chlorophenyl)-3-(2-hydroxy-4,6-dimethoxyphenyl)-1,3-dioxopropan-2-yl 4-chlorobenzoate (6aa)

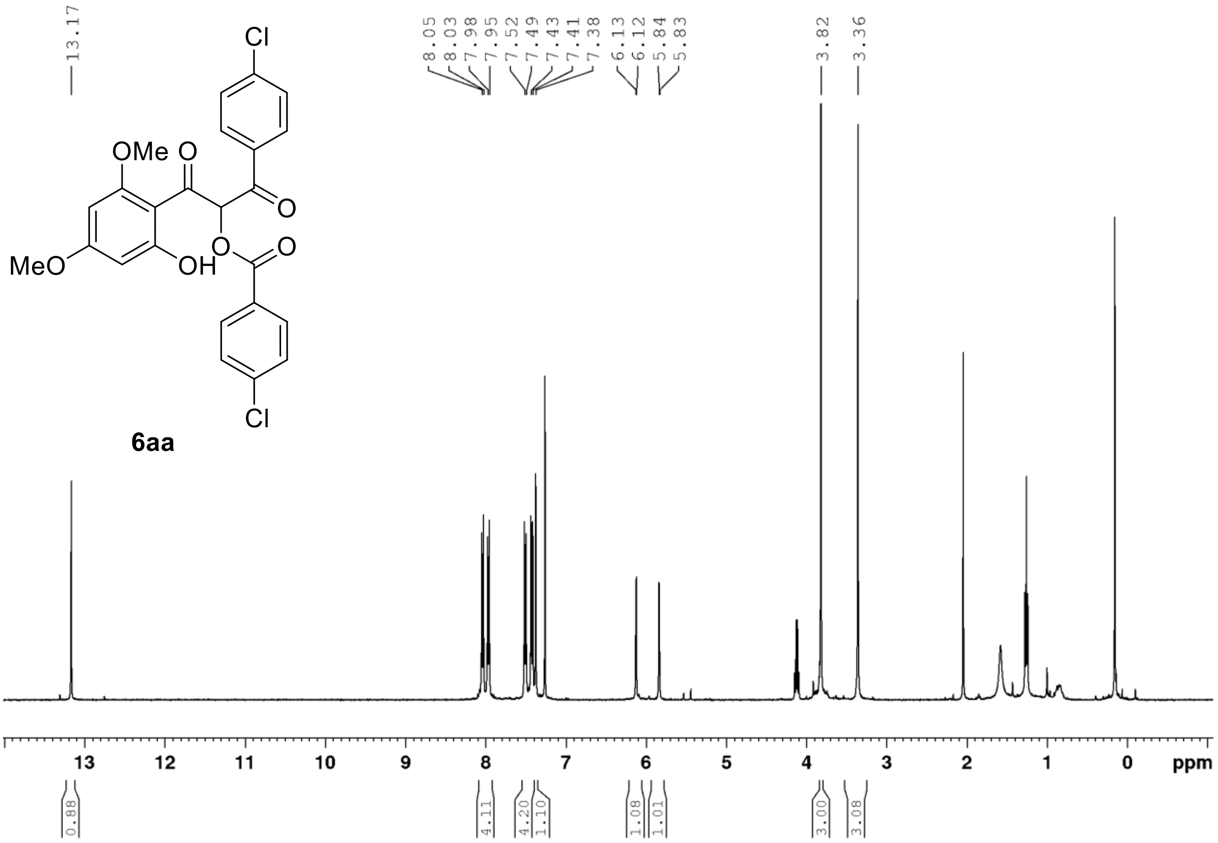

2-(4-Chlorophenyl)-5,7-dimethoxy-4-oxo-4H-chromen-3-yl 4-chlorobenzoate (7aa)

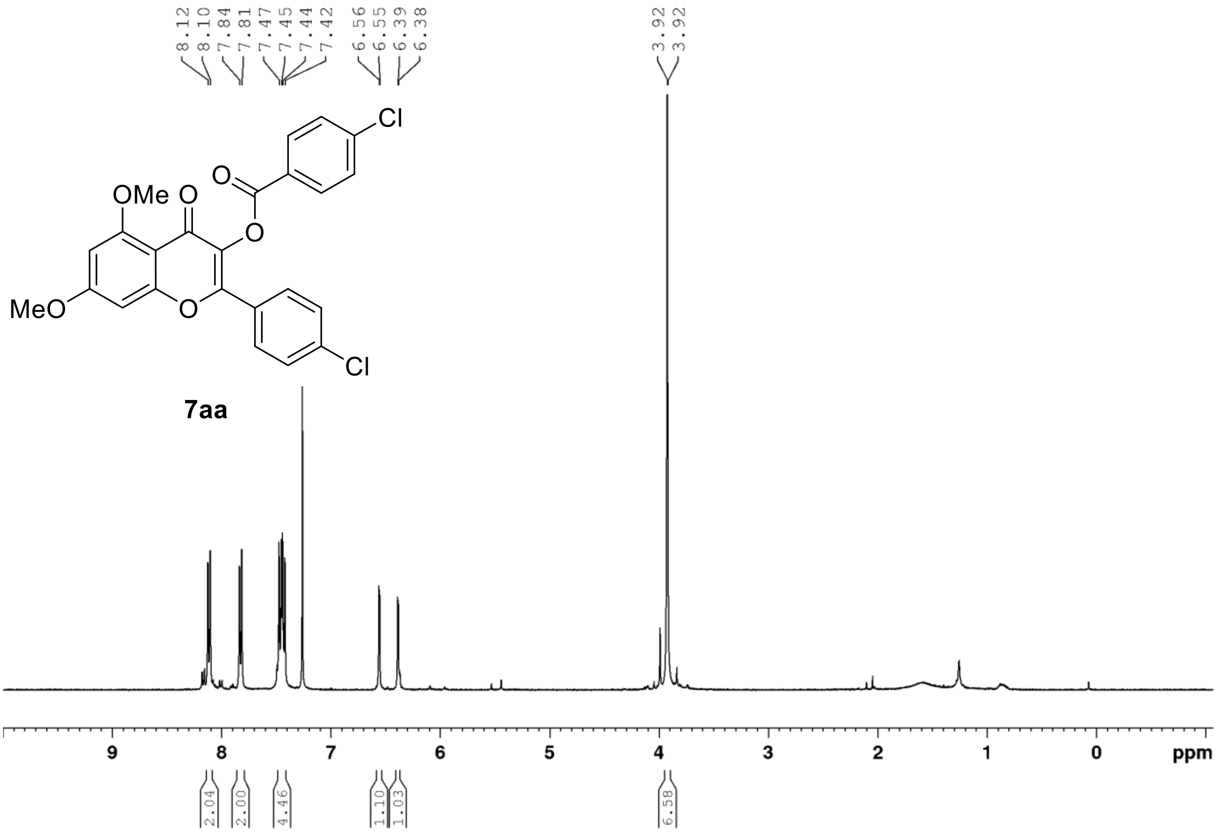

2-(4-chlorophenyl)-3-hydroxy-5,7-dimethoxy-4*H*-chromen-4-one (8aa)

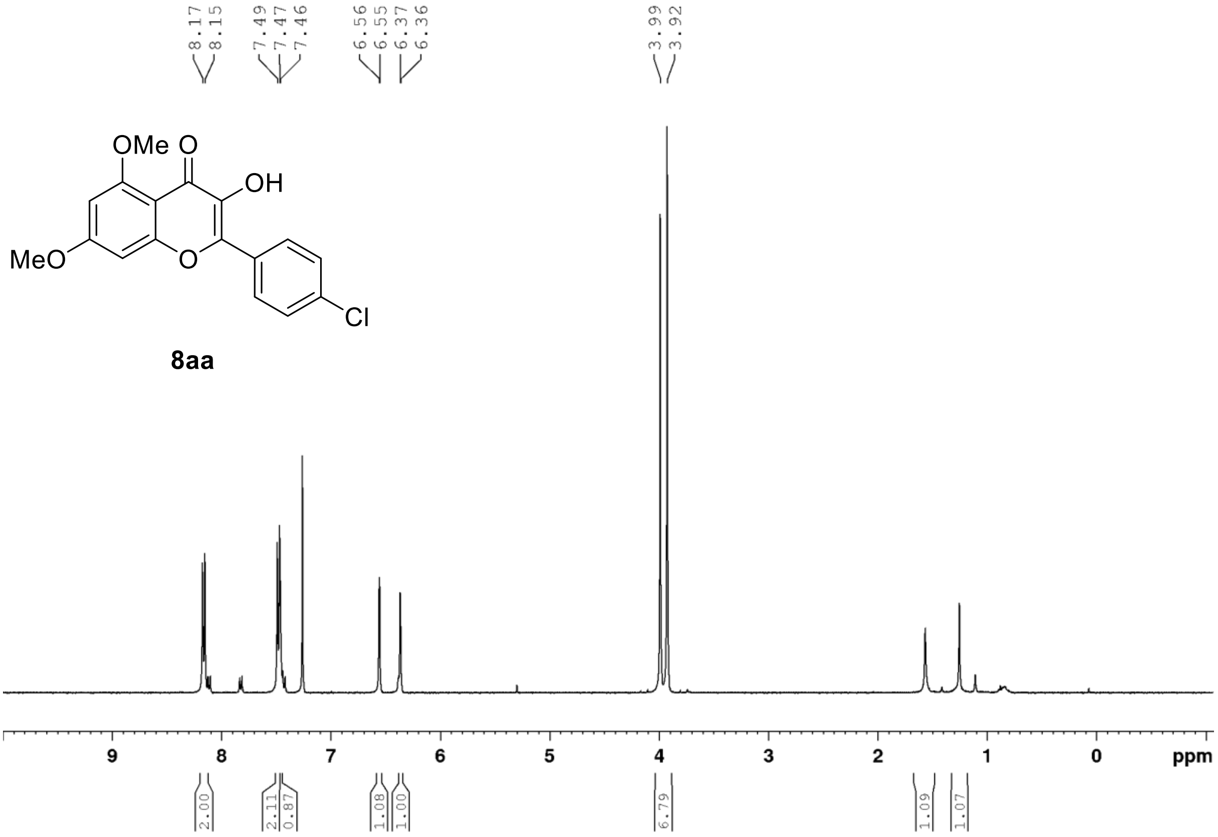

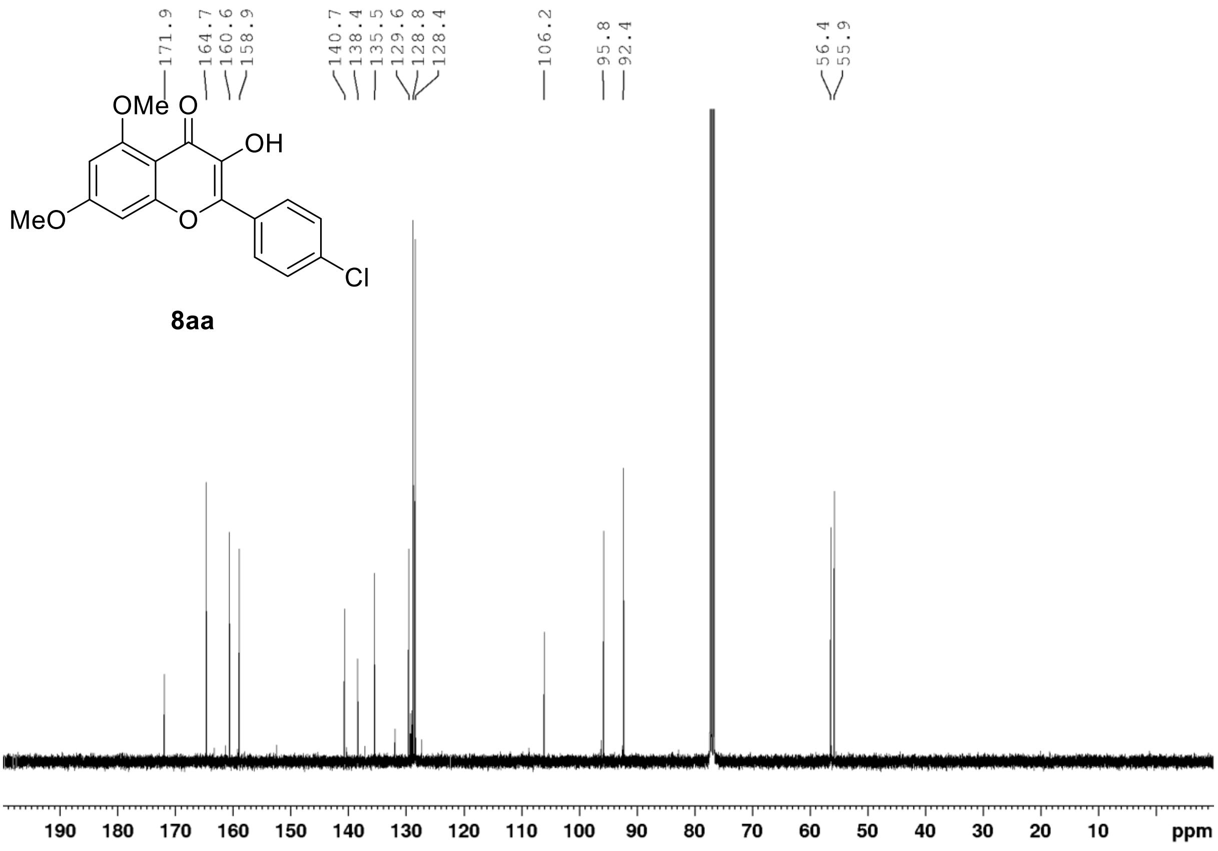

(±)-Methyl (1*R*,2*R*,3*S*,3a*R*,8b*S*)-3a-(4-chlorophenyl)-1,8b-dihydroxy-6,8-dimethoxy-3-phenyl-2,3,3a,8b-tetrahydro-1*H*-cyclopenta[*b*]benzofuran-2-carboxylate

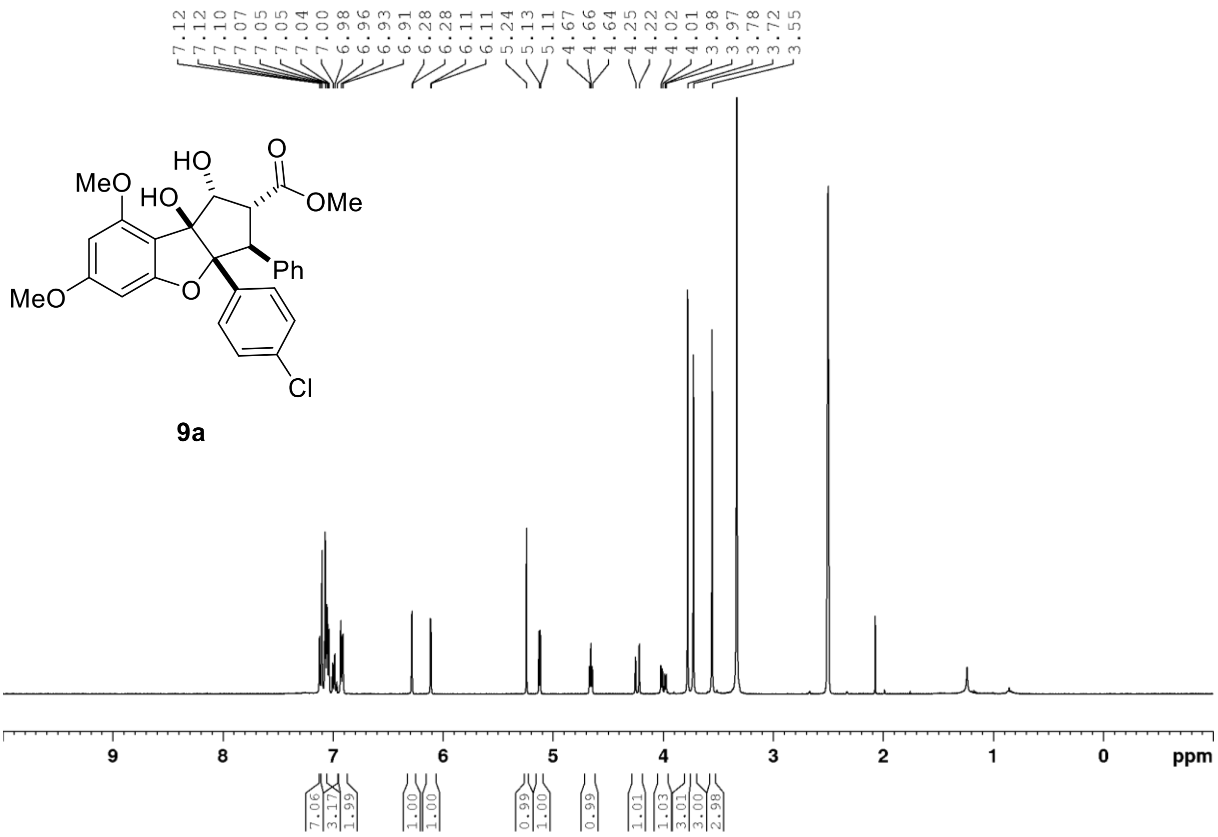

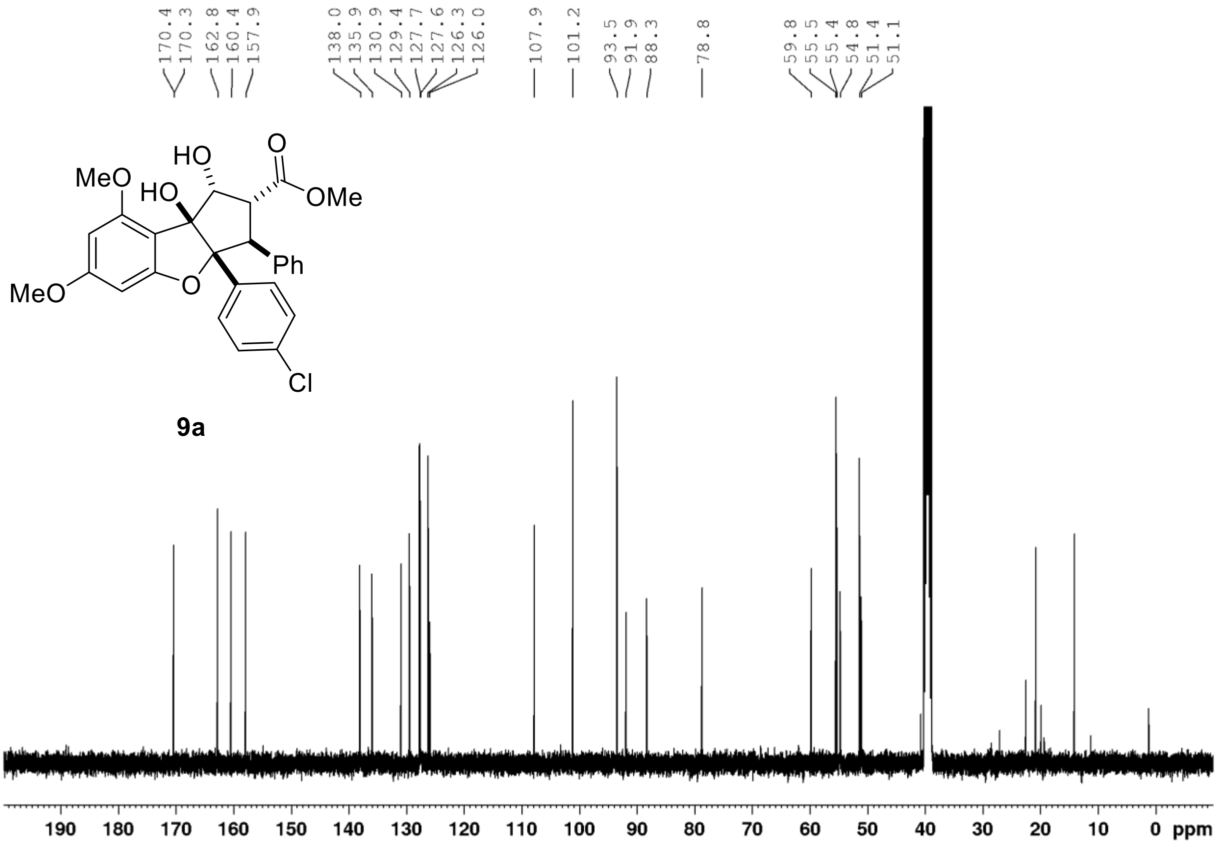

4.3. NMR spectroscopic data for the synthesis of 11ba

1-(4-(Benzyloxy)-2-hydroxy-6-methoxyphenyl)-2-hydroxyethan-1-one (4b)

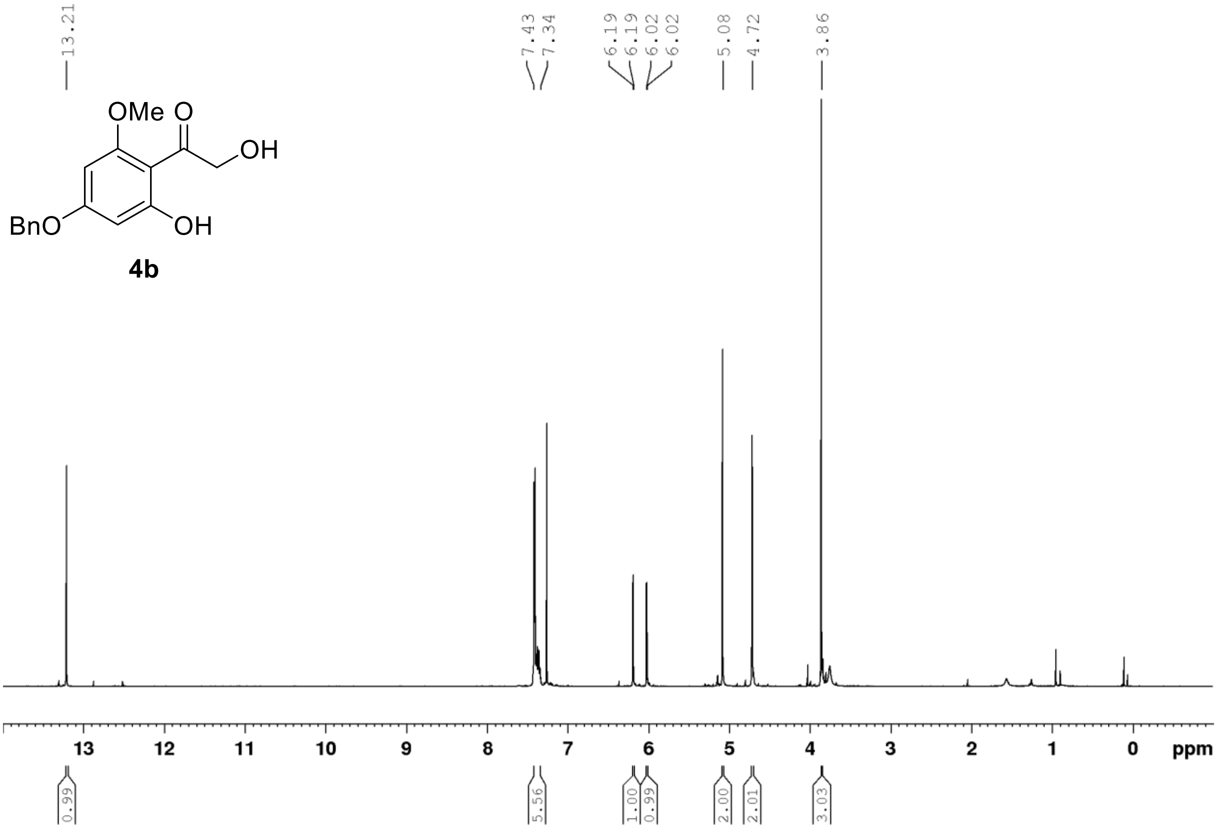

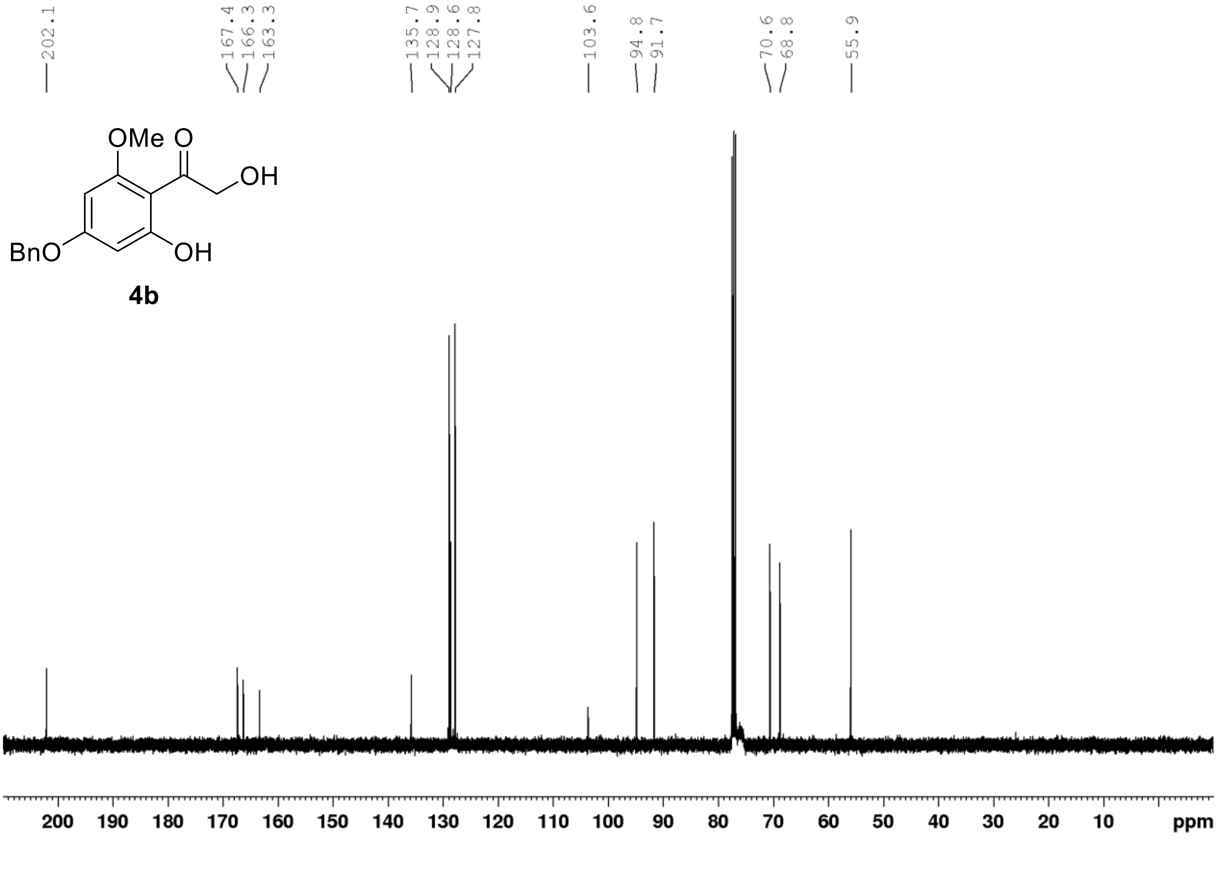

2-(4-(Benzyloxy)-2-((4-fluorobenzoyl)oxy)-6-methoxyphenyl)-2-oxoethyl 4-fluorobenzoate (5ba)

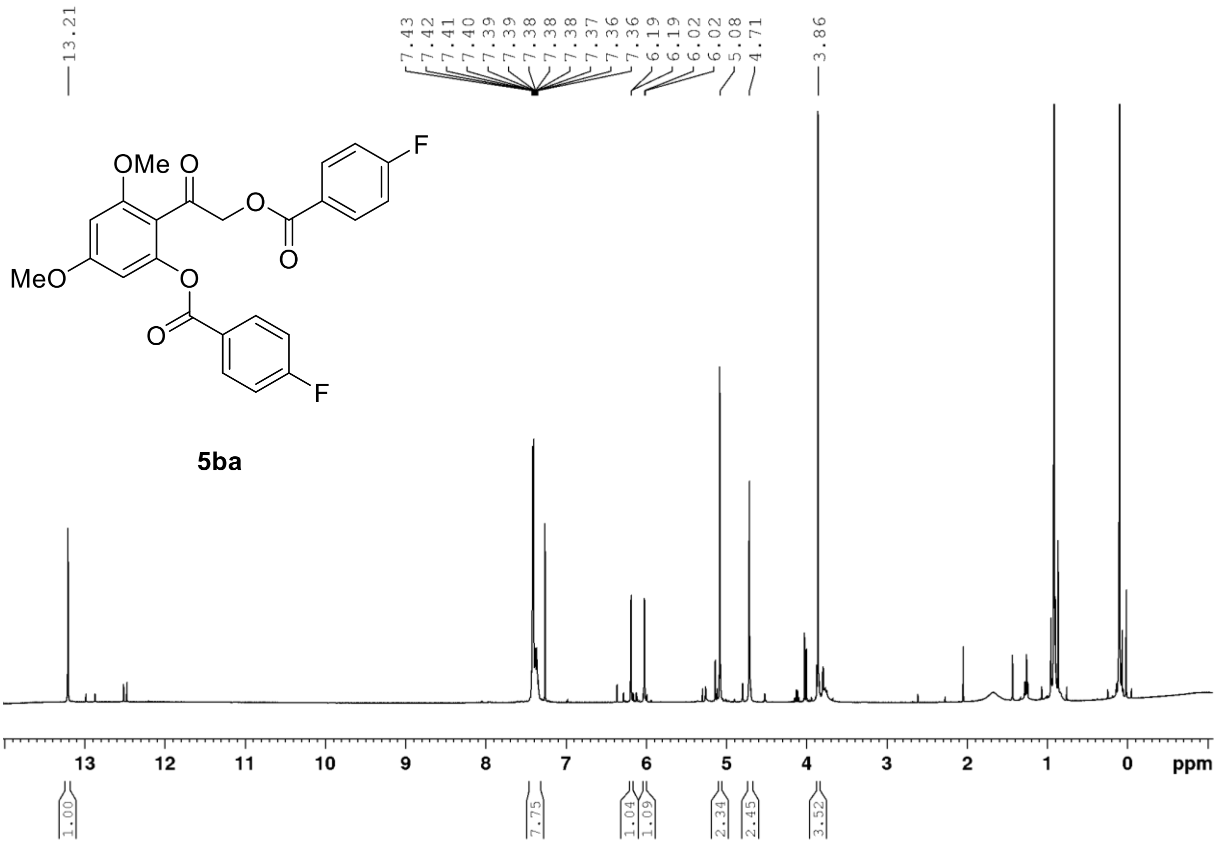

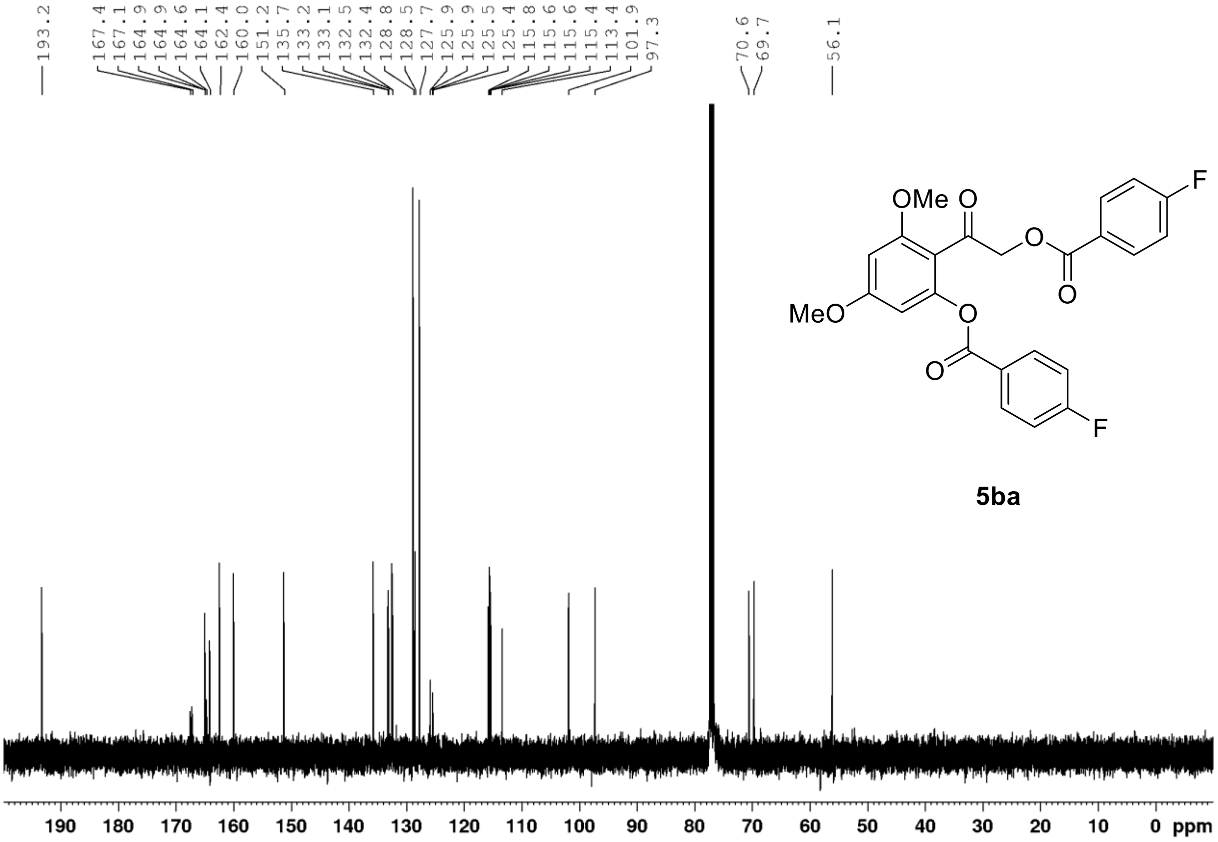

1-(4-(Benzyloxy)-2-hydroxy-6-methoxyphenyl)-3-(4-fluorophenyl)-1,3-dioxopropan-2-yl 4-fluorobenzoate (6ba)

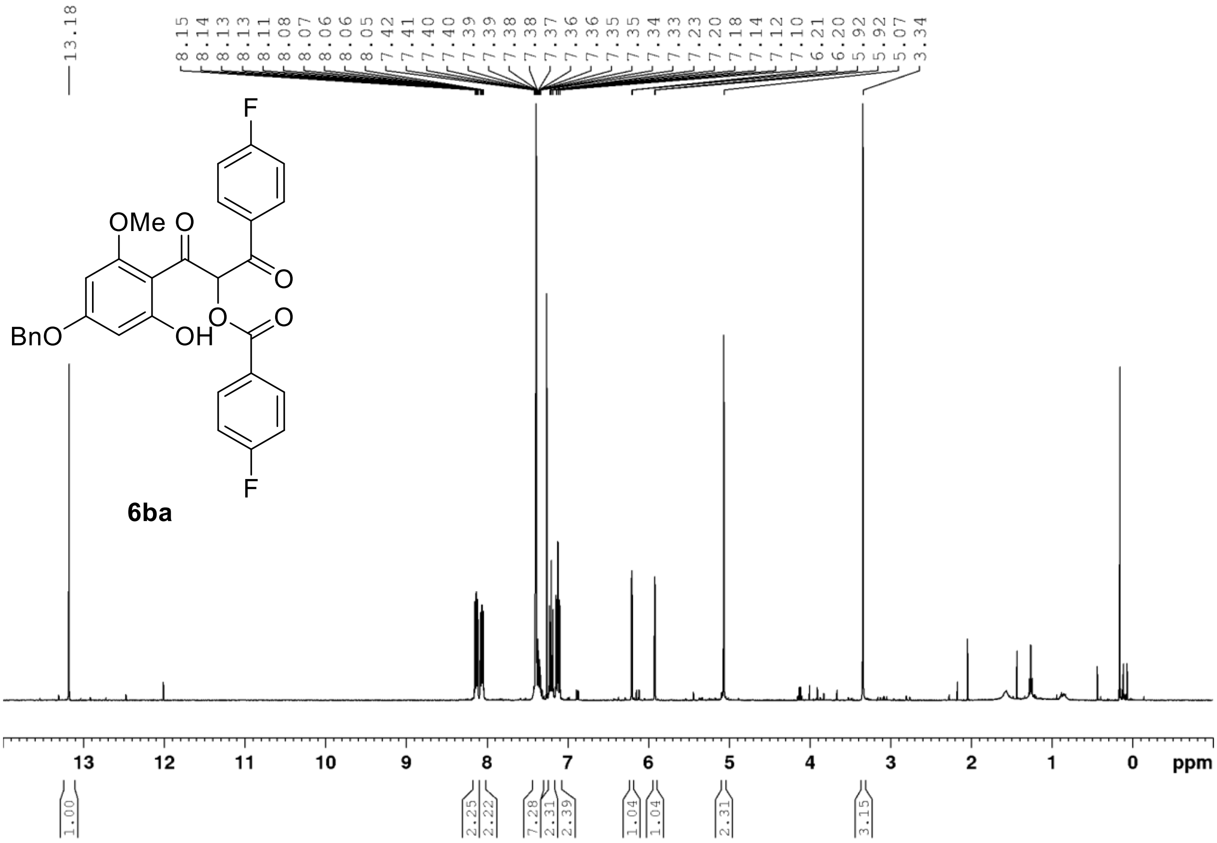

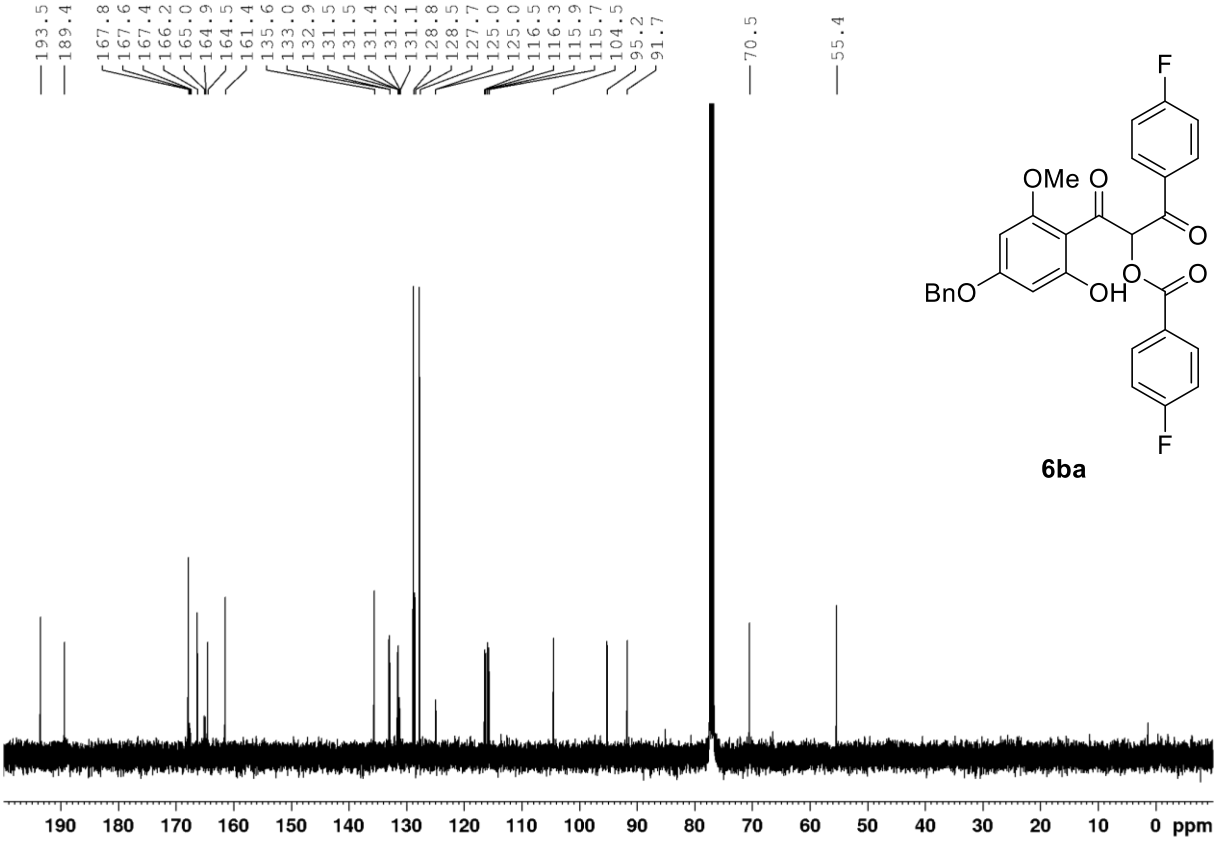

7-(Benzyloxy)-2-(4-fluorophenyl)-5-methoxy-4-oxo-4H-chromen-3-yl 4-fluorobenzoate (7ba)

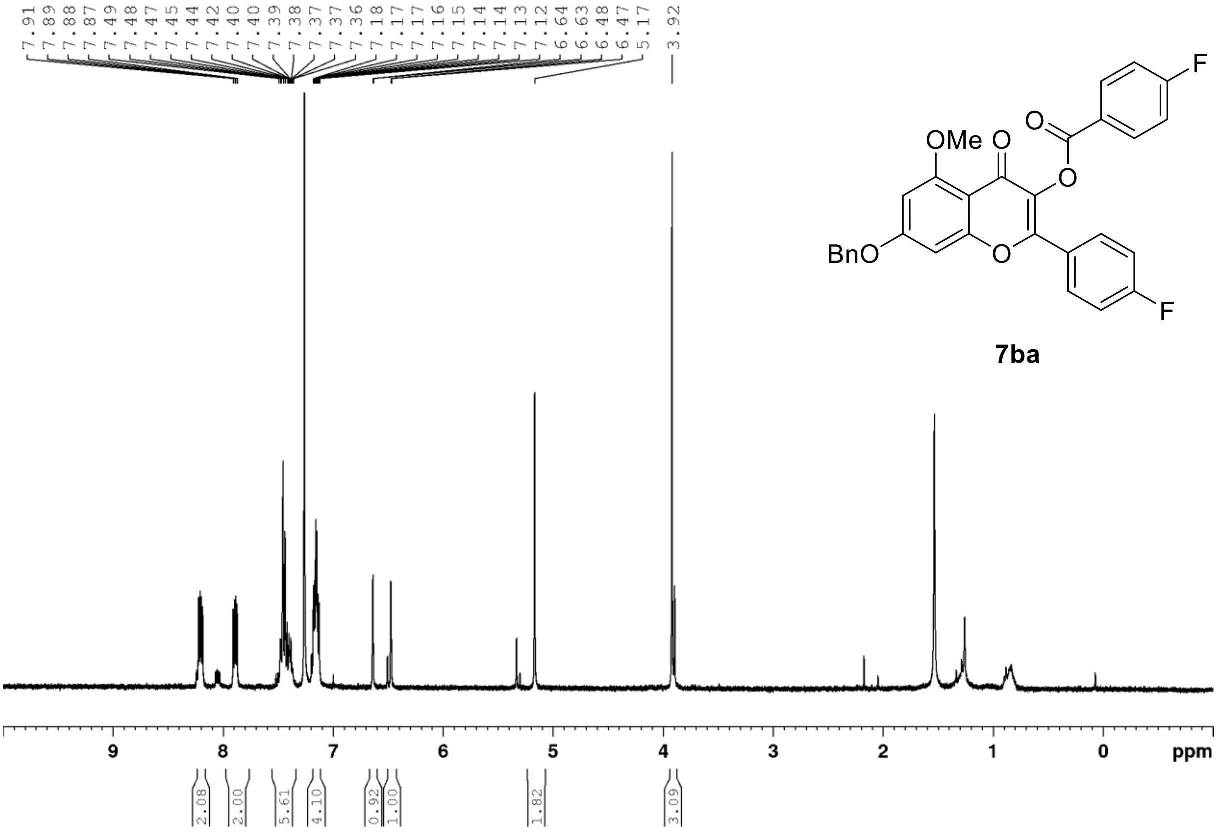

7-(Benzyloxy)-2-(4-fluorophenyl)-3-hydroxy-5-methoxy-4*H*-chromen-4-one (8ba)

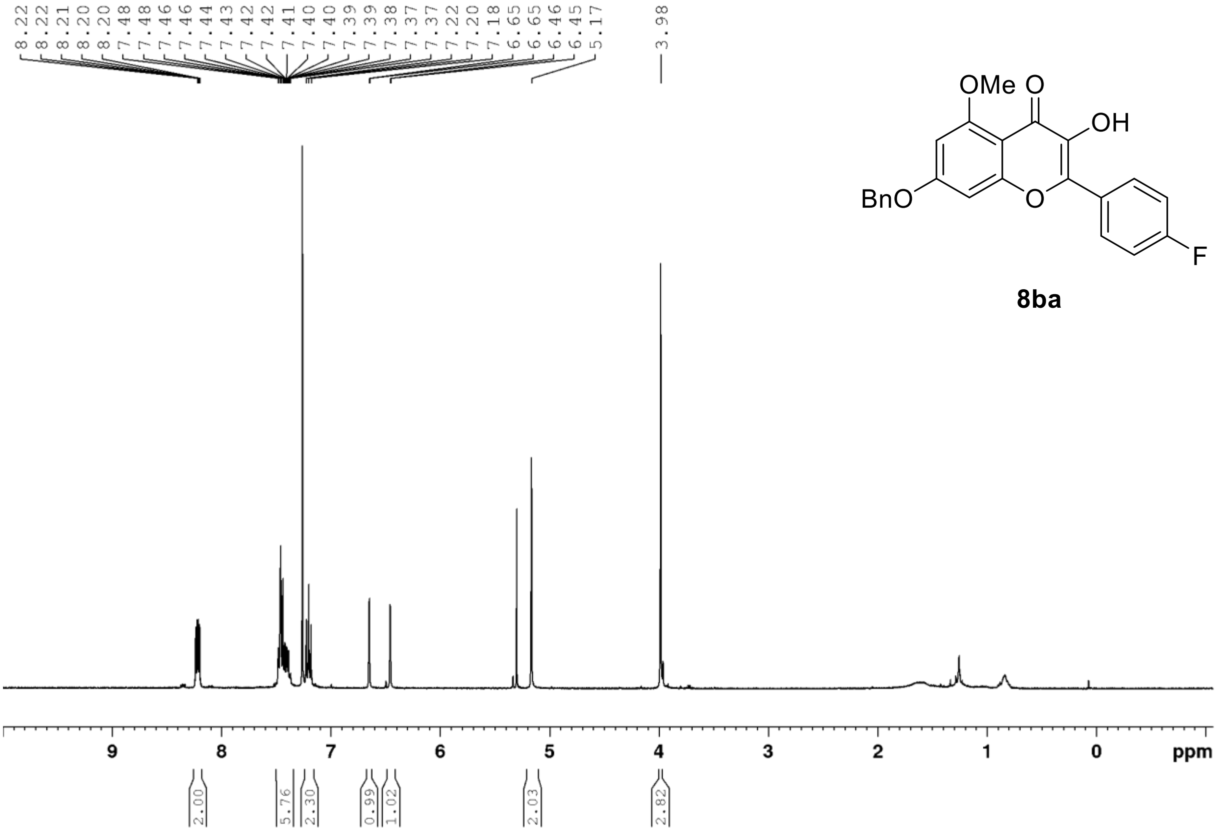

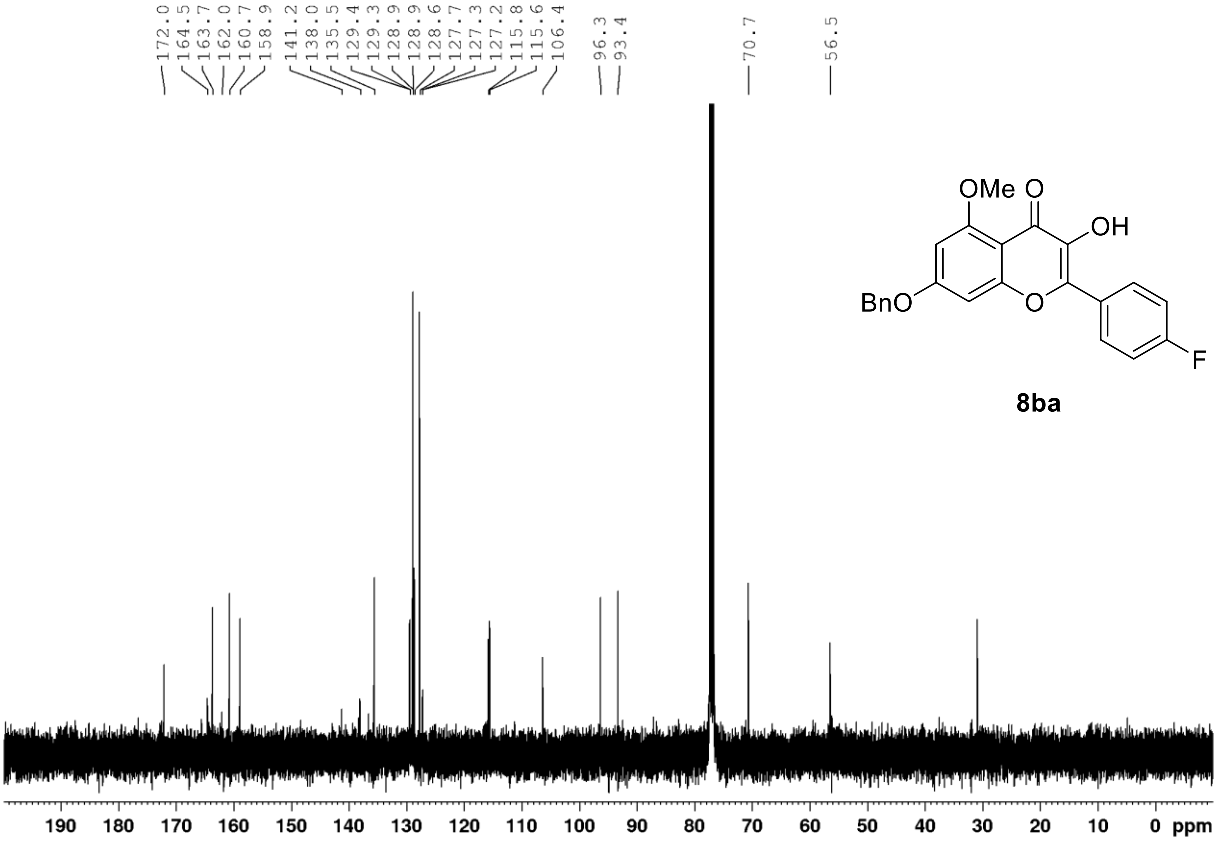
172.0
164.5
163.7
162.0
160.7
158.9
141.2
138.0
135.5
129.4
129.3
128.9
128.9
128.6
127.7
127.3
127.2
115.8
115.6
106.4
96.3
93.4
70.7
56.5
OMe
O
OH
BnO
O
F
8ba
190 180 170 160 150 140 130 120 110 100 90 80 70 60 50 40 30 20 10 0 ppm

(±) Methyl (1*R*,2*R*,3*S*,3a*R*,8b*S*)-6-(benzyloxy)-3a-(4-fluorophenyl)-1,8b-dihydroxy-8-methoxy-3-phenyl-2,3,3a,8b-tetrahydro-1*H*-cyclopenta[*b*]benzofuran-2-carboxylate (10ba)

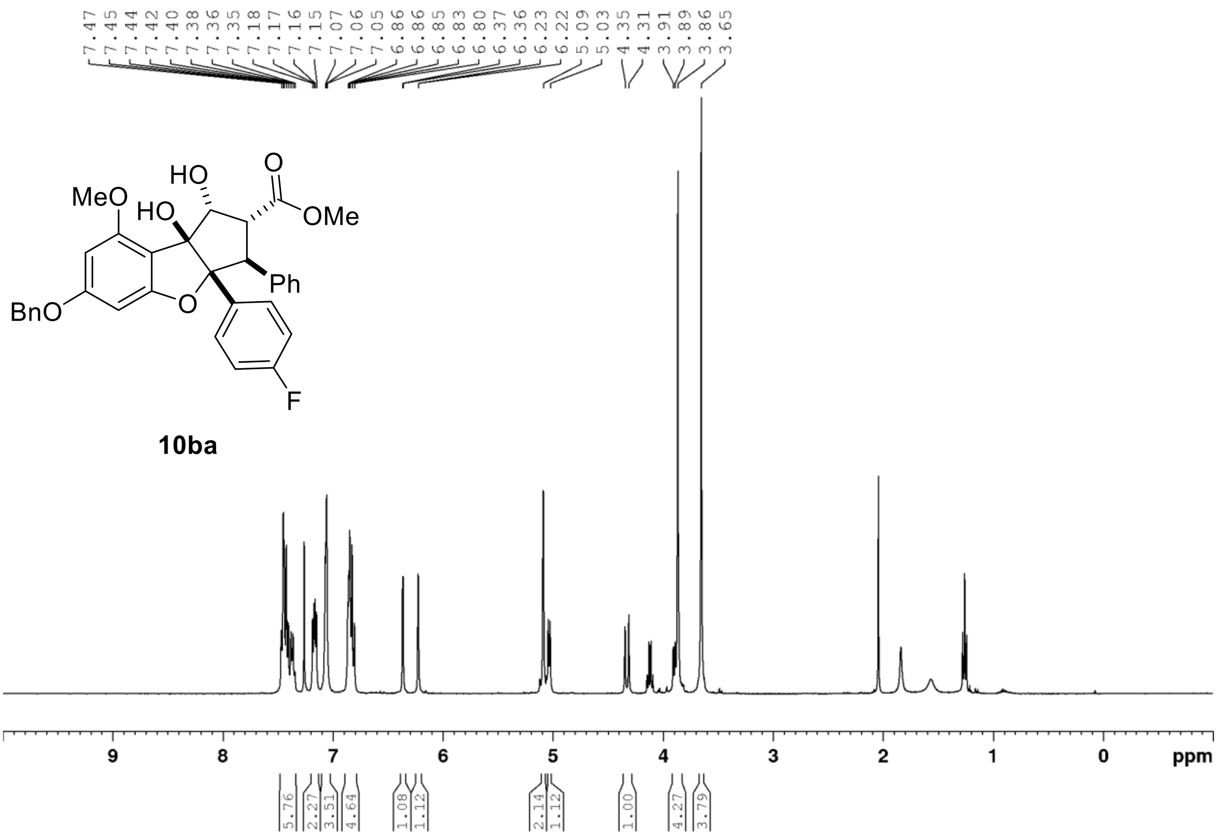

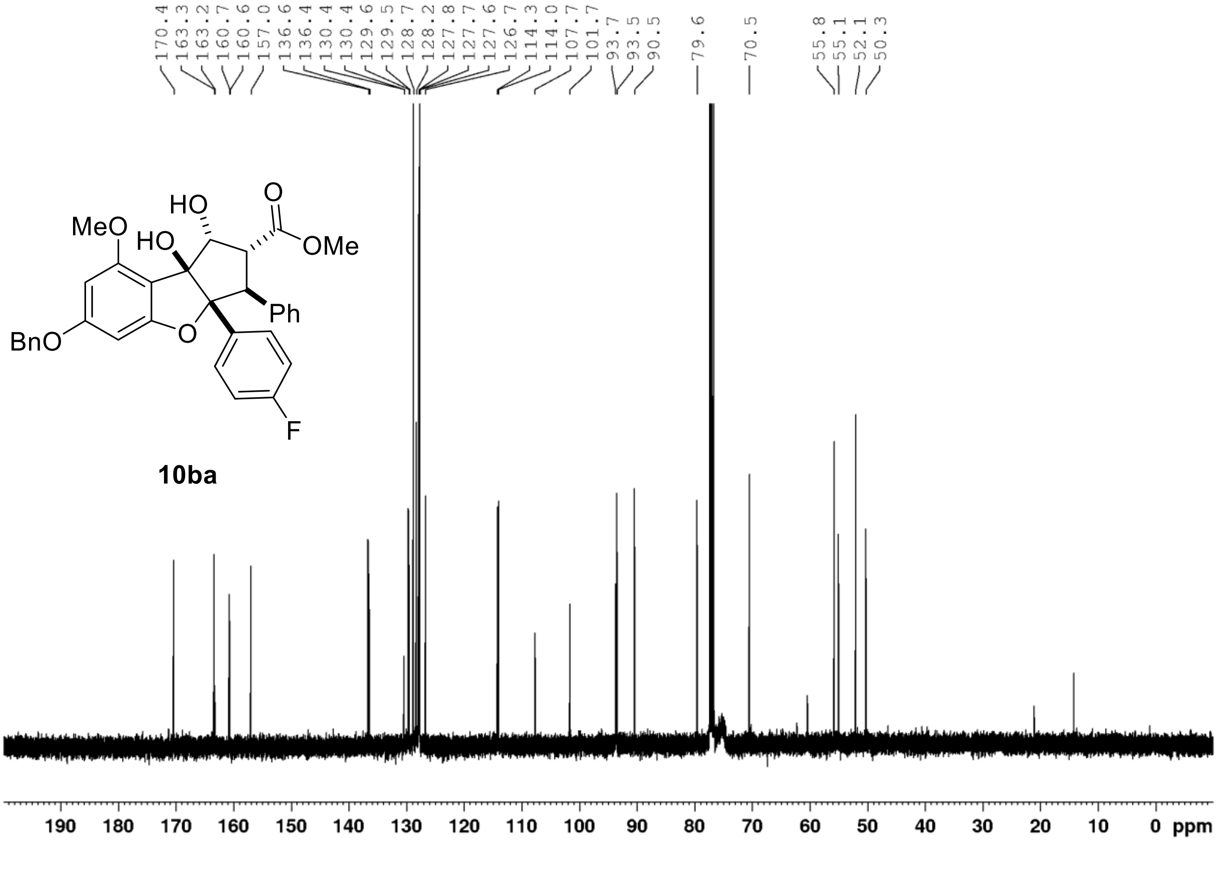

(±)-Methyl (1*R*,2*R*,3*S*,3a*R*,8b*S*)-3a-(4-fluorophenyl)-1,8b-dihydroxy-6,8-dimethoxy-3-phenyl-2,3,3a,8b-tetrahydro-1*H*-cyclopenta[*b*]benzofuran-2-carboxylate (11ba)

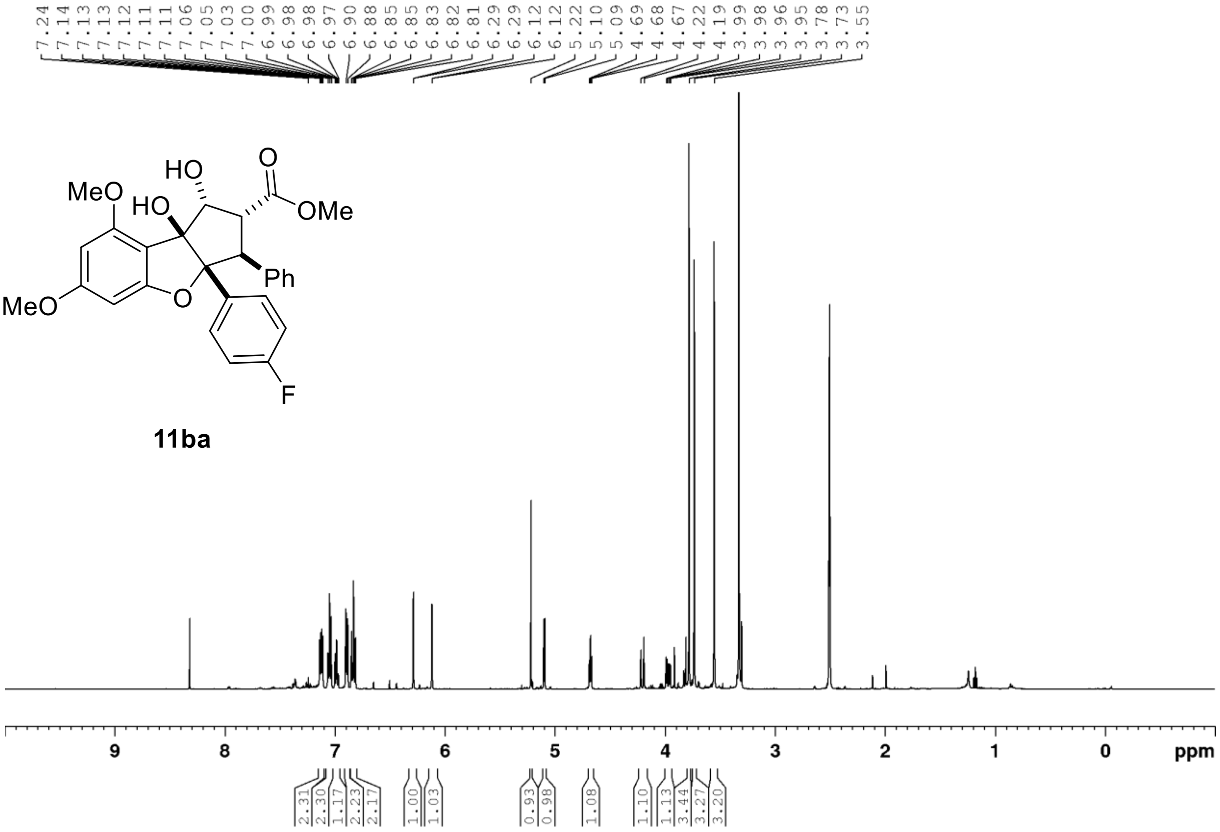

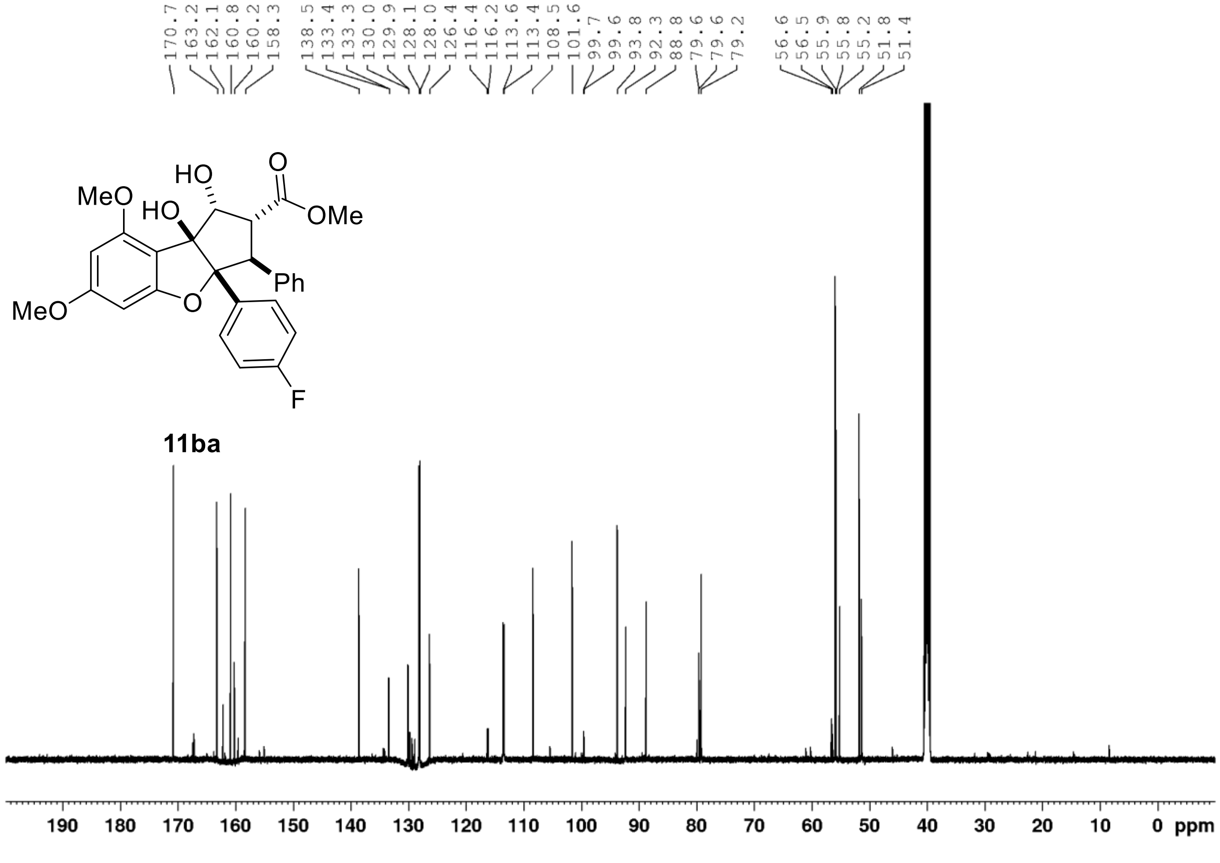

4.4. NMR spectroscopic data for the synthesis of 11bb

1-(4-(Benzyloxy)-2-hydroxy-6-methoxyphenyl)-3-(4-bromophenyl)-1,3-dioxopropan-2-yl 4-bromobenzoate (6bb)

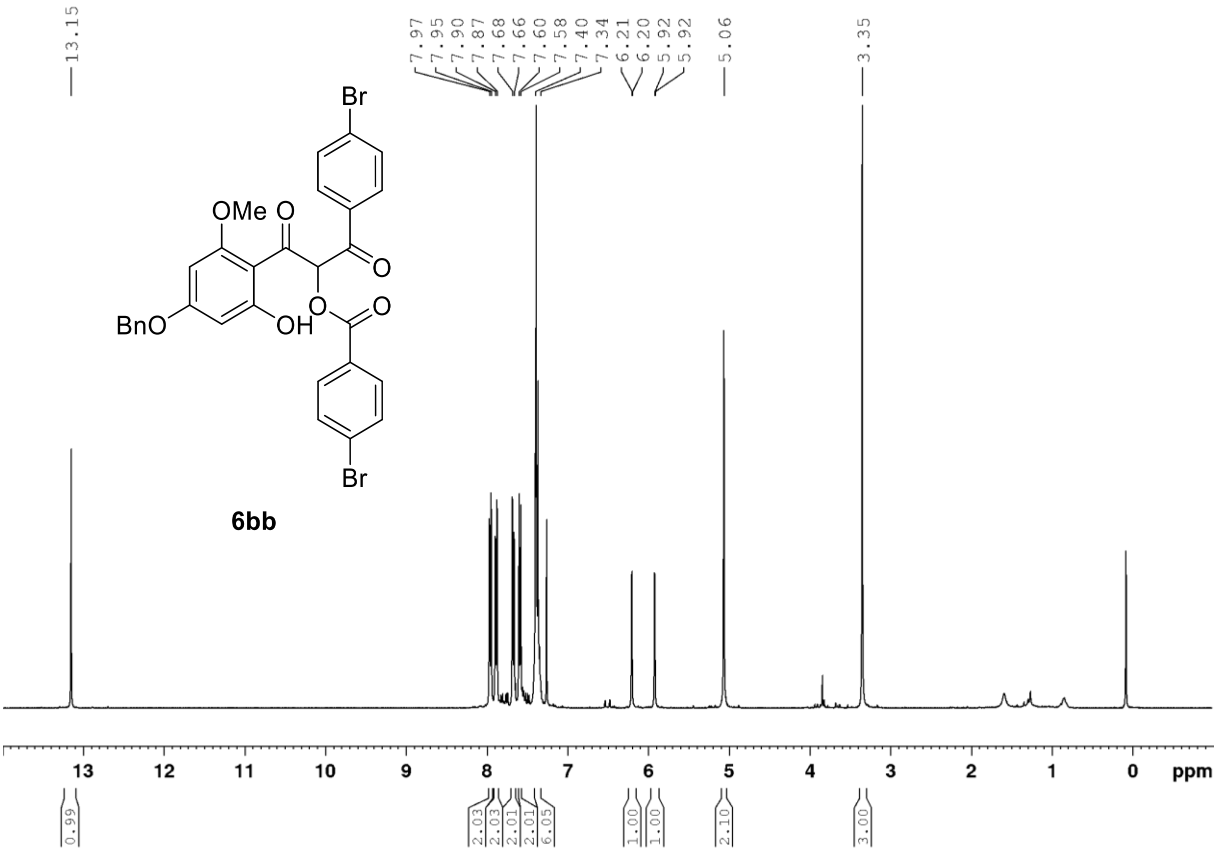

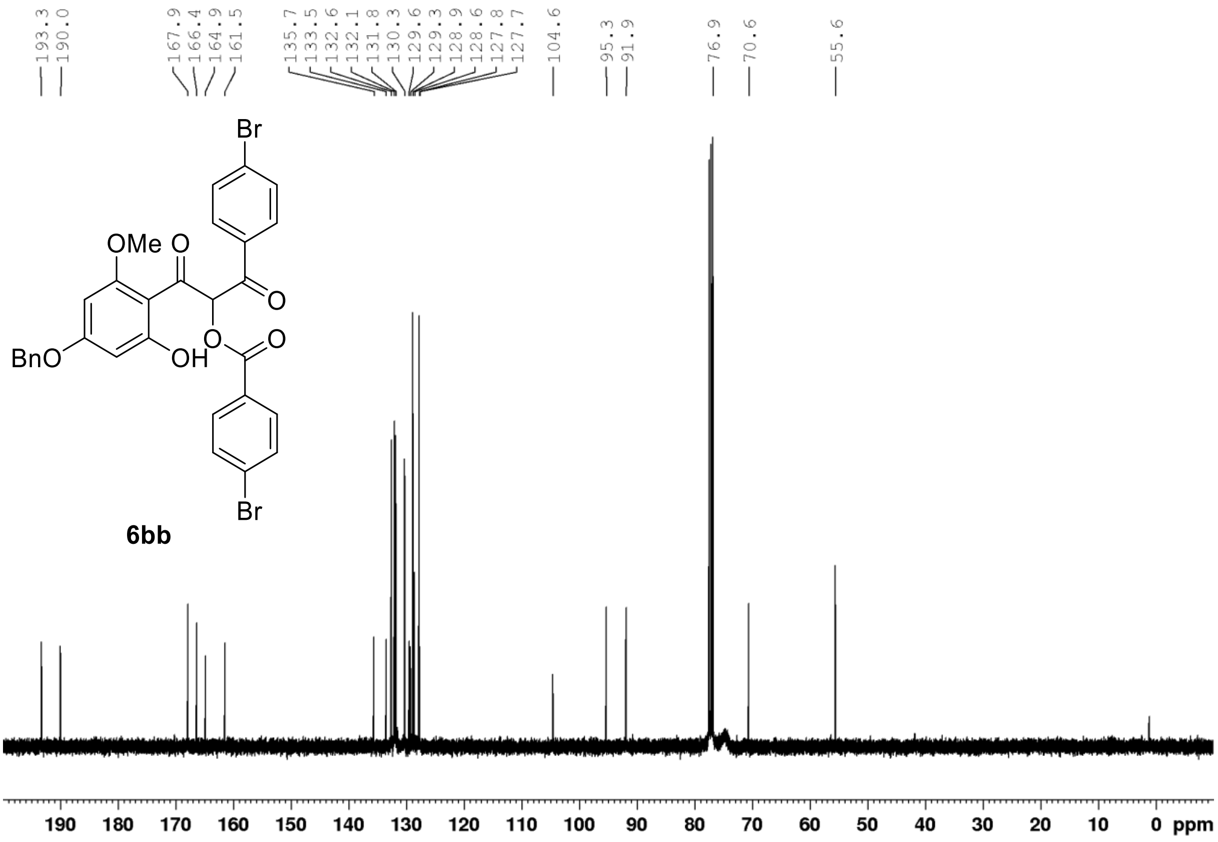

7-(Benzyloxy)-2-(4-bromophenyl)-5-methoxy-4-oxo-4*H*-chromen-3-yl 4-bromobenzoate (7bb)

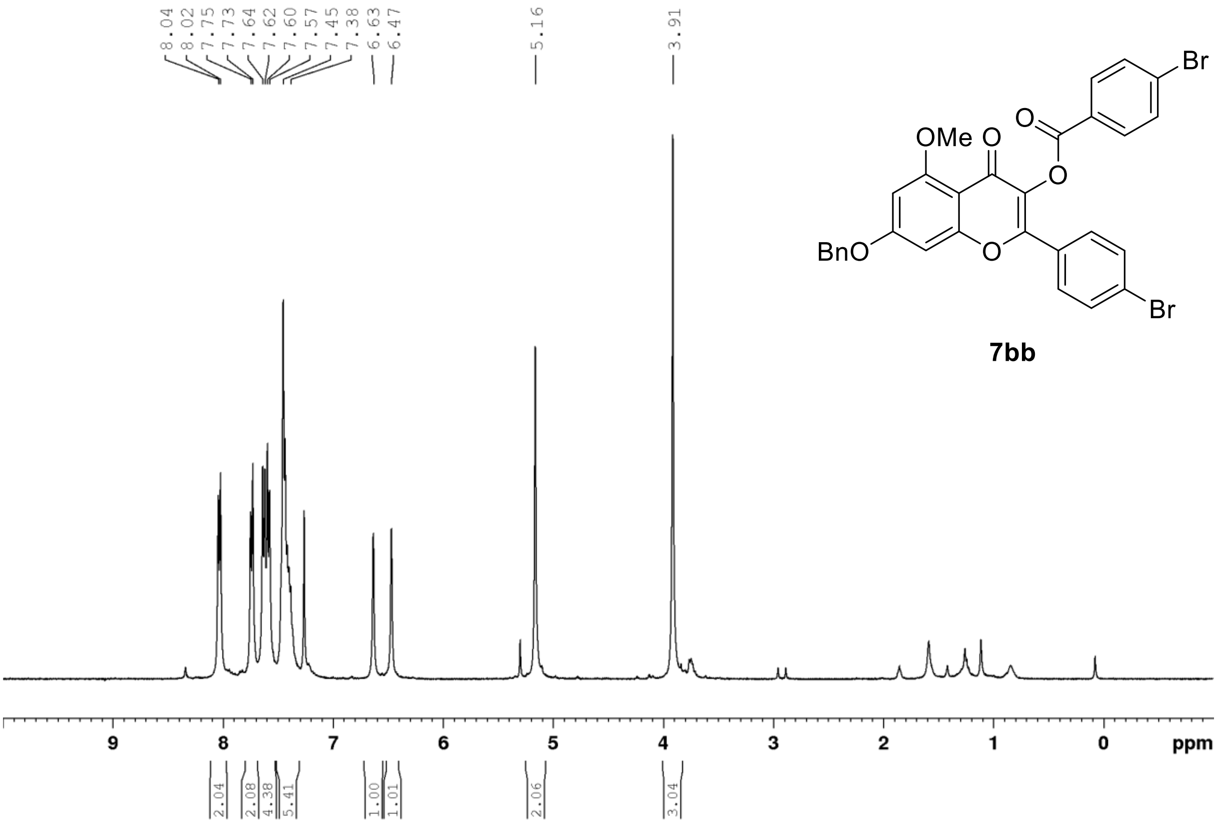

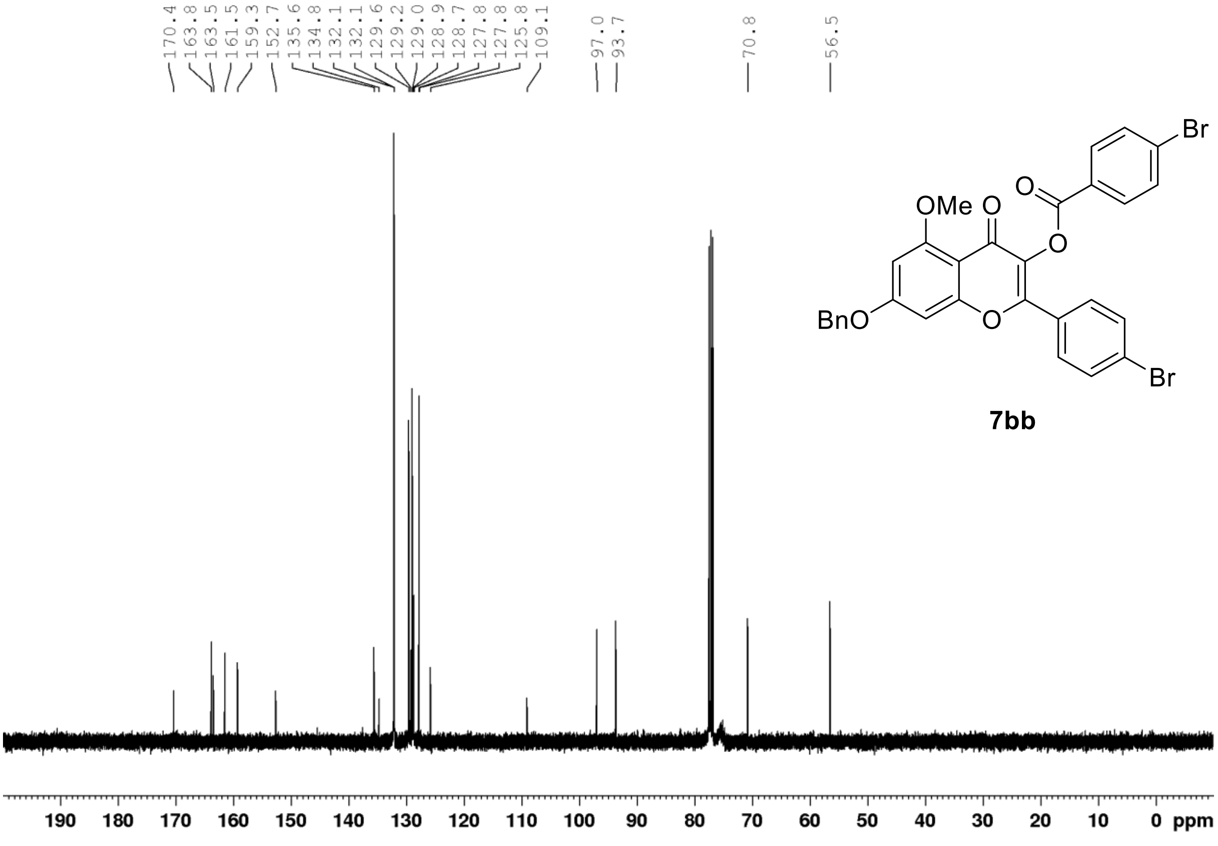

7-(Benzyloxy)-2-(4-bromophenyl)-3-hydroxy-5-methoxy-4*H*-chromen-4-one (8bb)

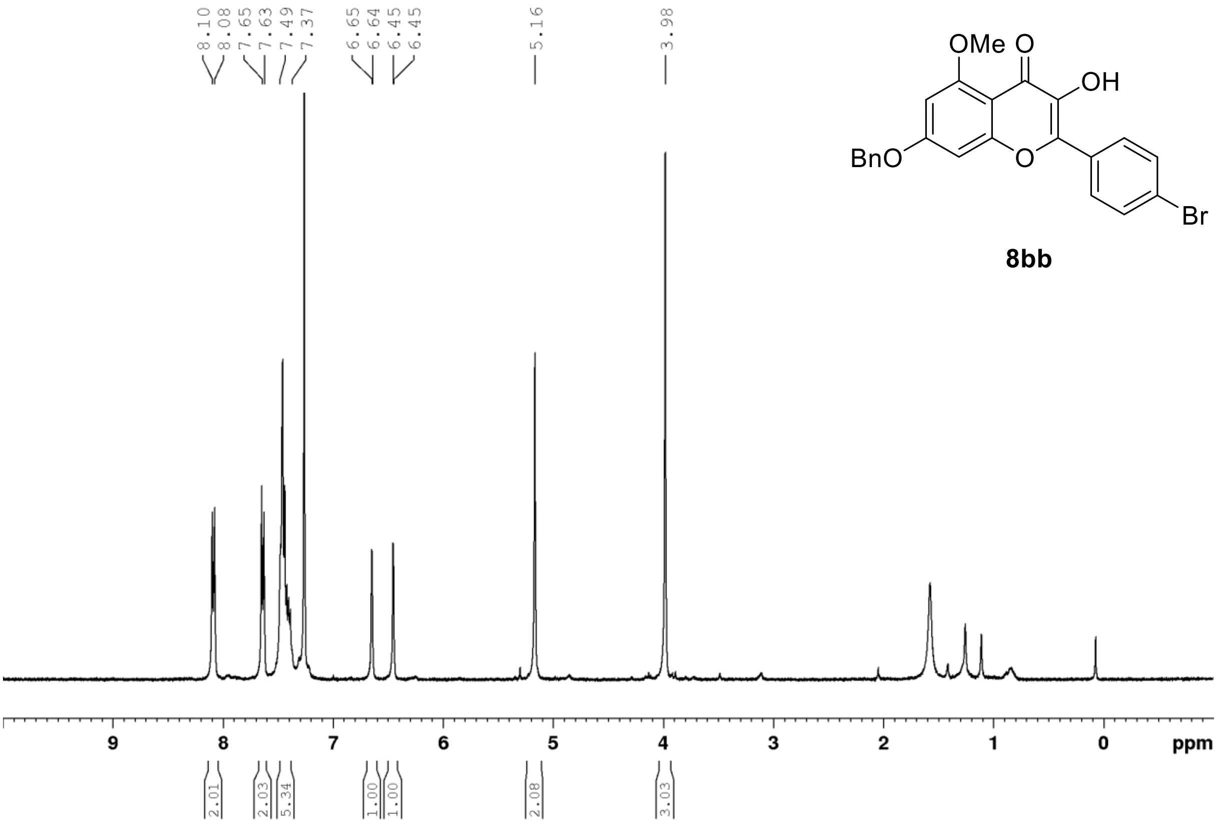

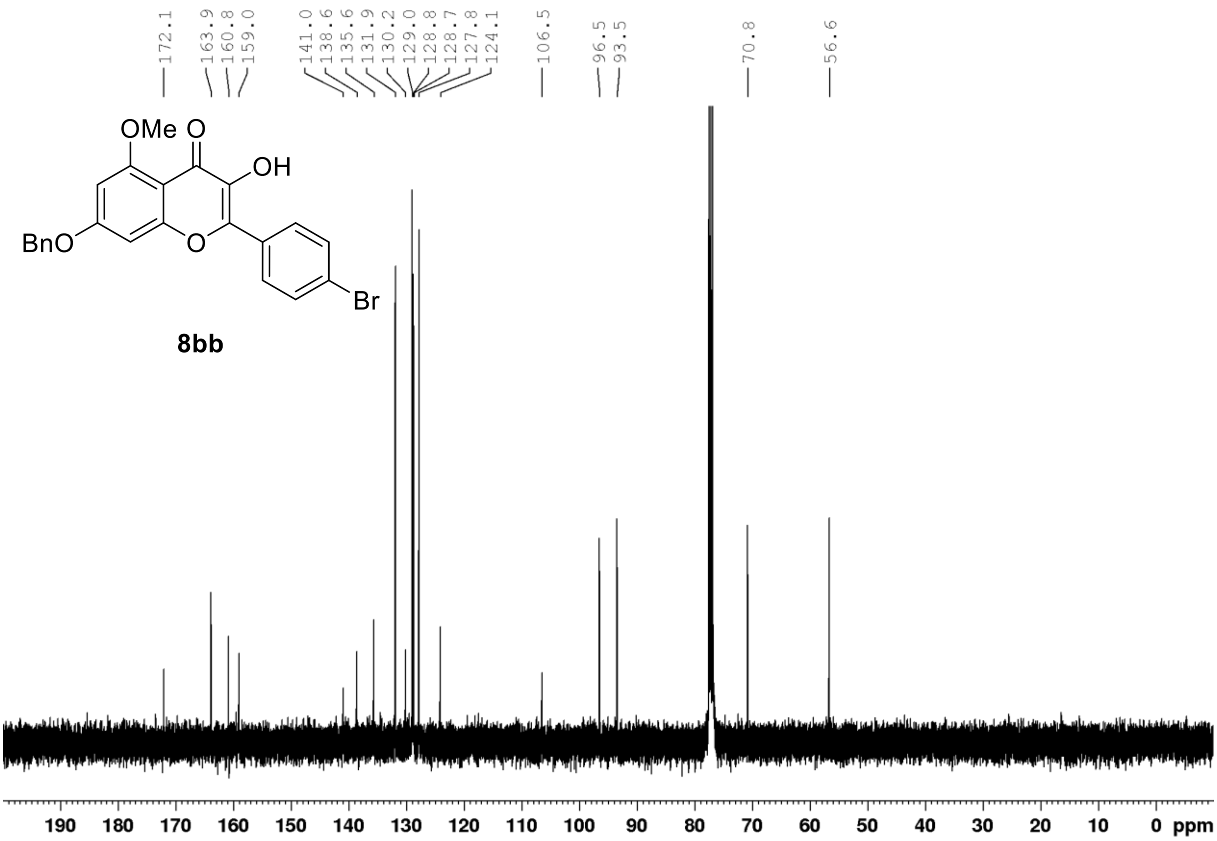

(±)-Methyl (1*R*,2*R*,3*S*,3a*R*,8b*S*)-6-(Benzyloxy)-3a-(4-bromophenyl)-1,8b-dihydroxy-8-methoxy-3-phenyl-2,3,3a,8b-tetrahydro-1*H*-cyclopenta[*b*]benzofuran-2-carboxylate (9bb)

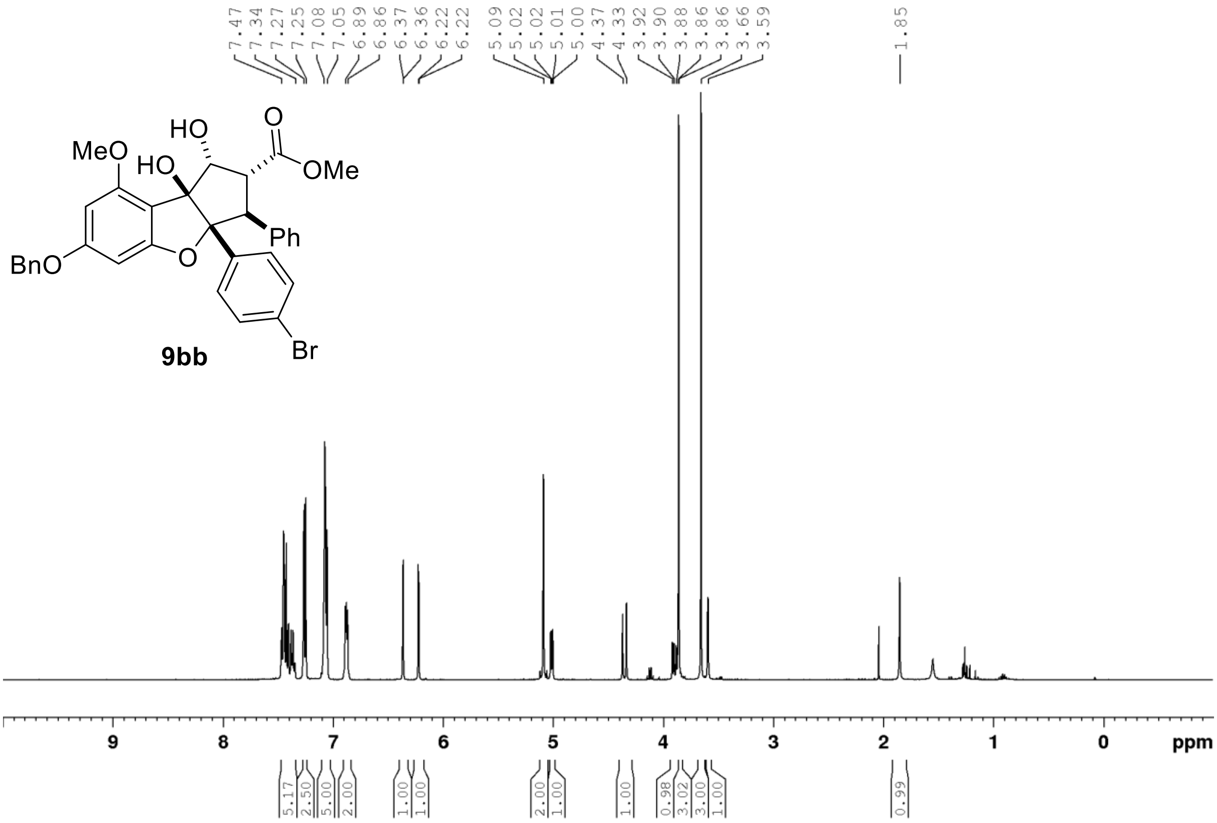

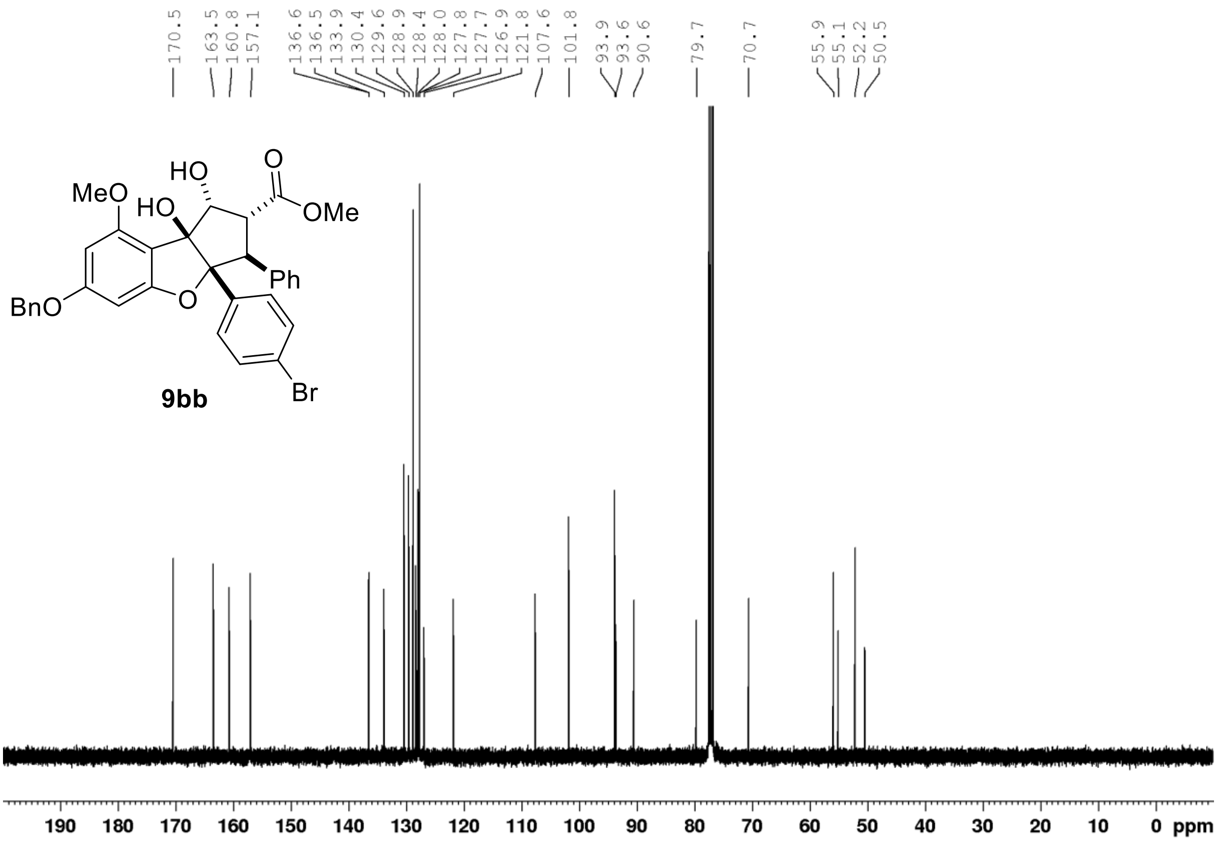

(±)-Methyl (1*R*,2*R*,3*S*,3a*R*,8b*S*)-3a-(4-bromophenyl)-1,6,8b-trihydroxy-8-methoxy-3-phenyl-2,3,3a,8b-tetrahydro-1*H*-cyclopenta[*b*]benzofuran-2-carboxylate (10bb)

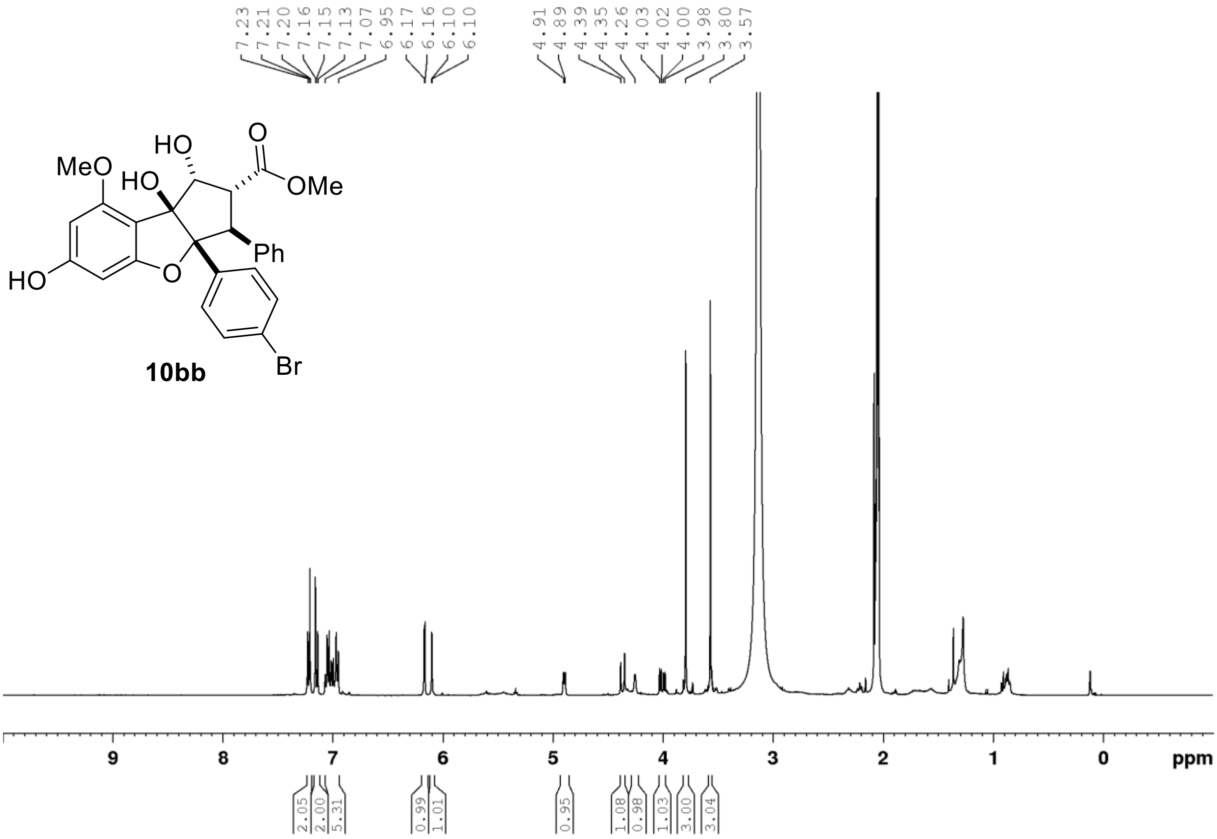

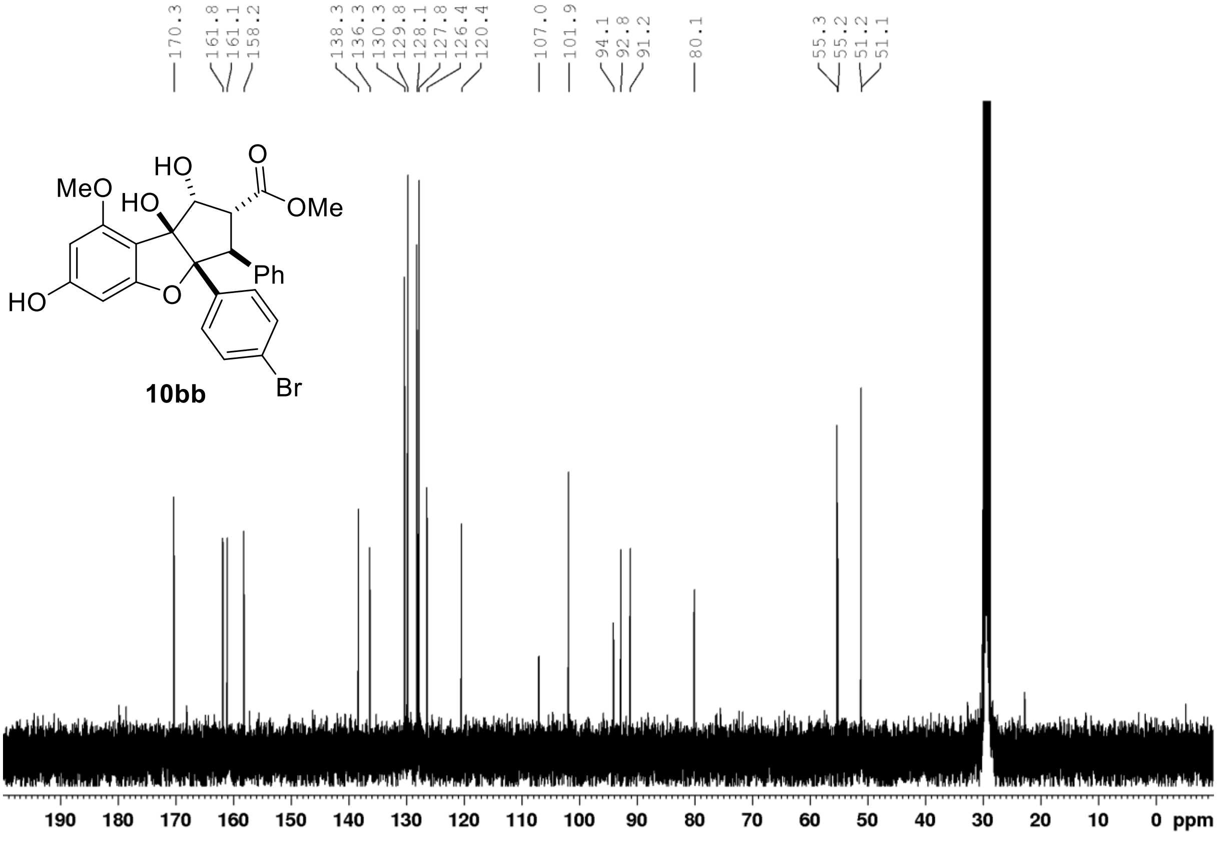

(±)-Methyl (1*R*,2*R*,3*S*,3a*R*,8b*S*)-3a-(4-bromophenyl)-1,8b-dihydroxy-6,8-dimethoxy-3-phenyl-2,3,3a,8b-tetrahydro-1*H*-cyclopenta[*b*]benzofuran-2-carboxylate (11bb)

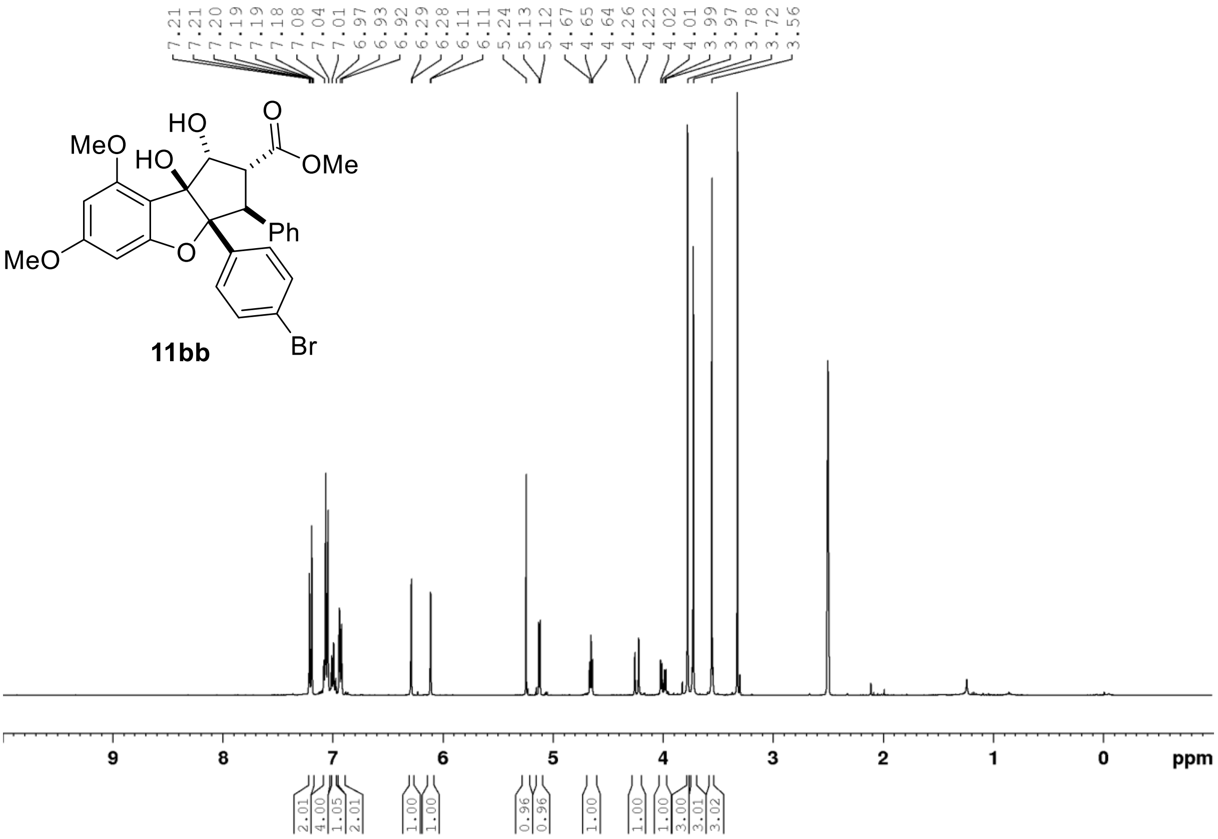

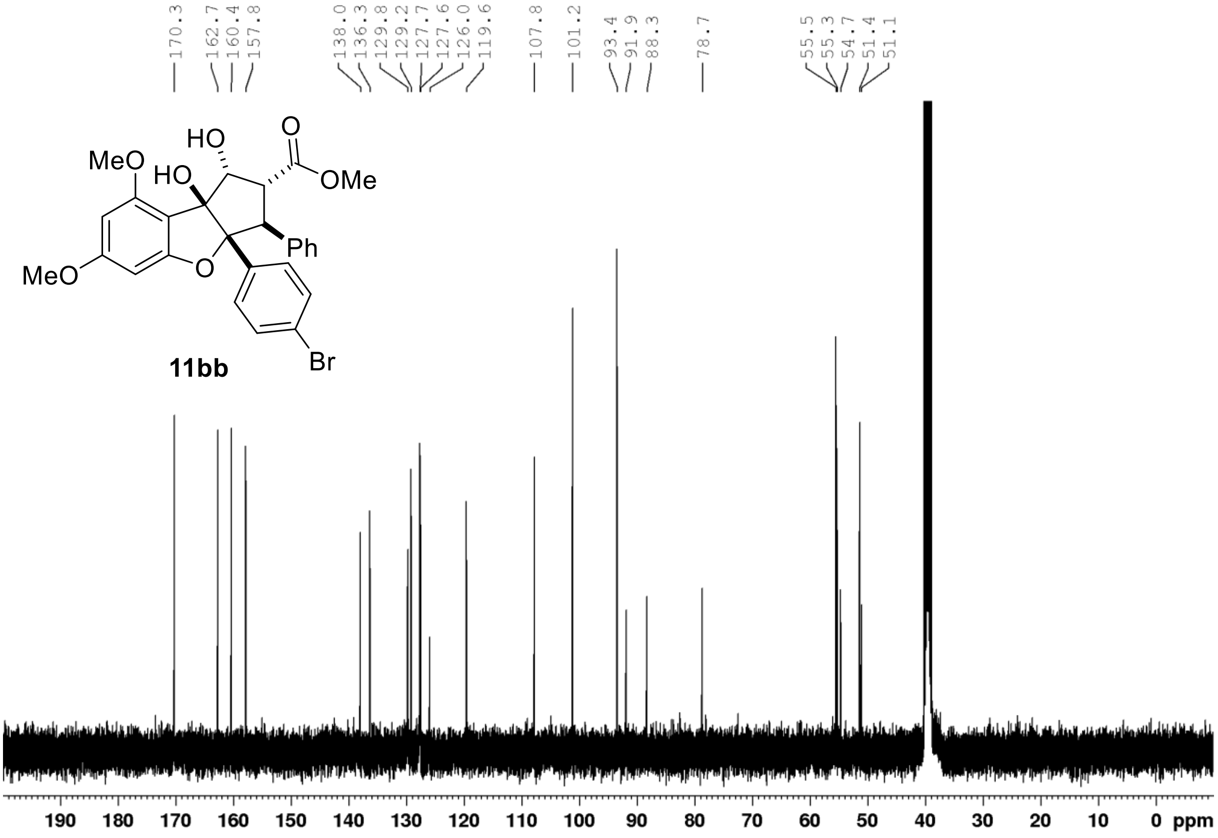

4.5. NMR spectroscopic data for the synthesis of 11bc

7-(Benzyloxy)-5-methoxy-2-(4-methoxyphenyl)-4-oxo-4*H*-chromen-3-yl 4-methoxybenzo-ate (7bc)

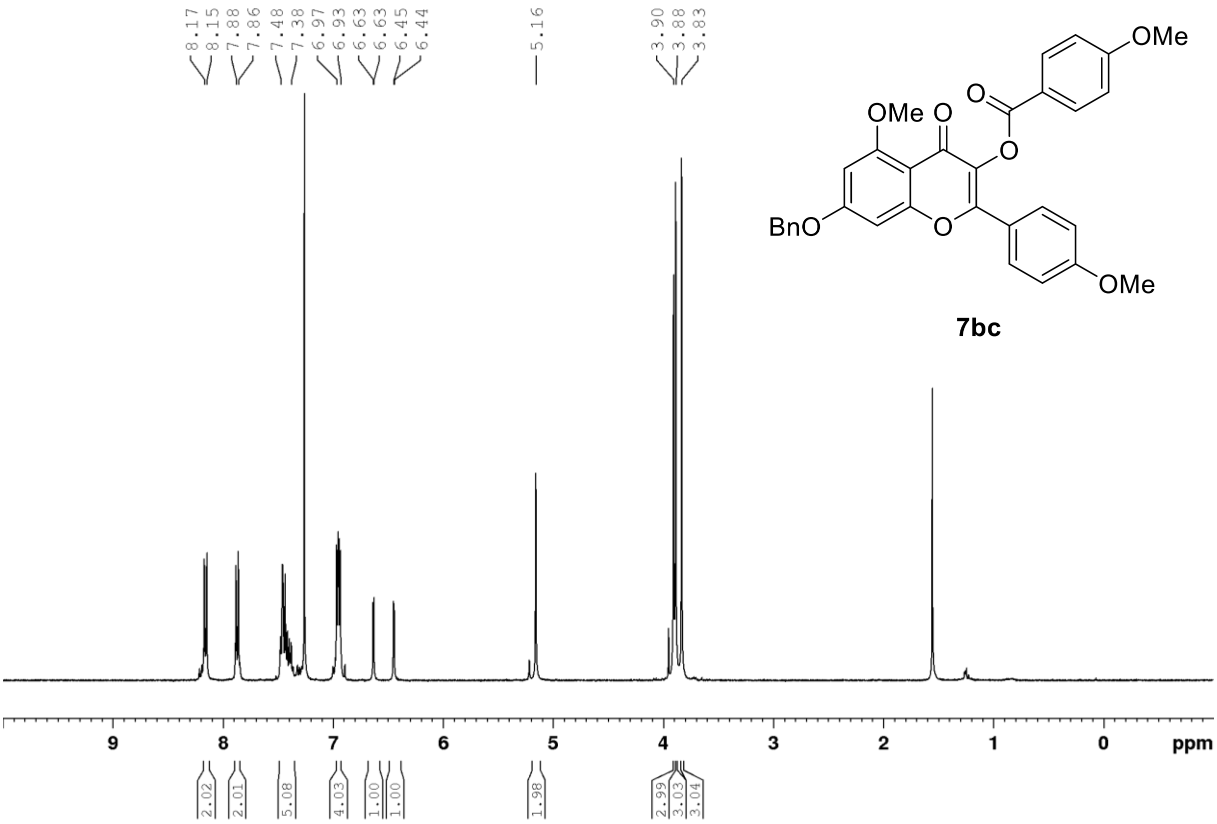

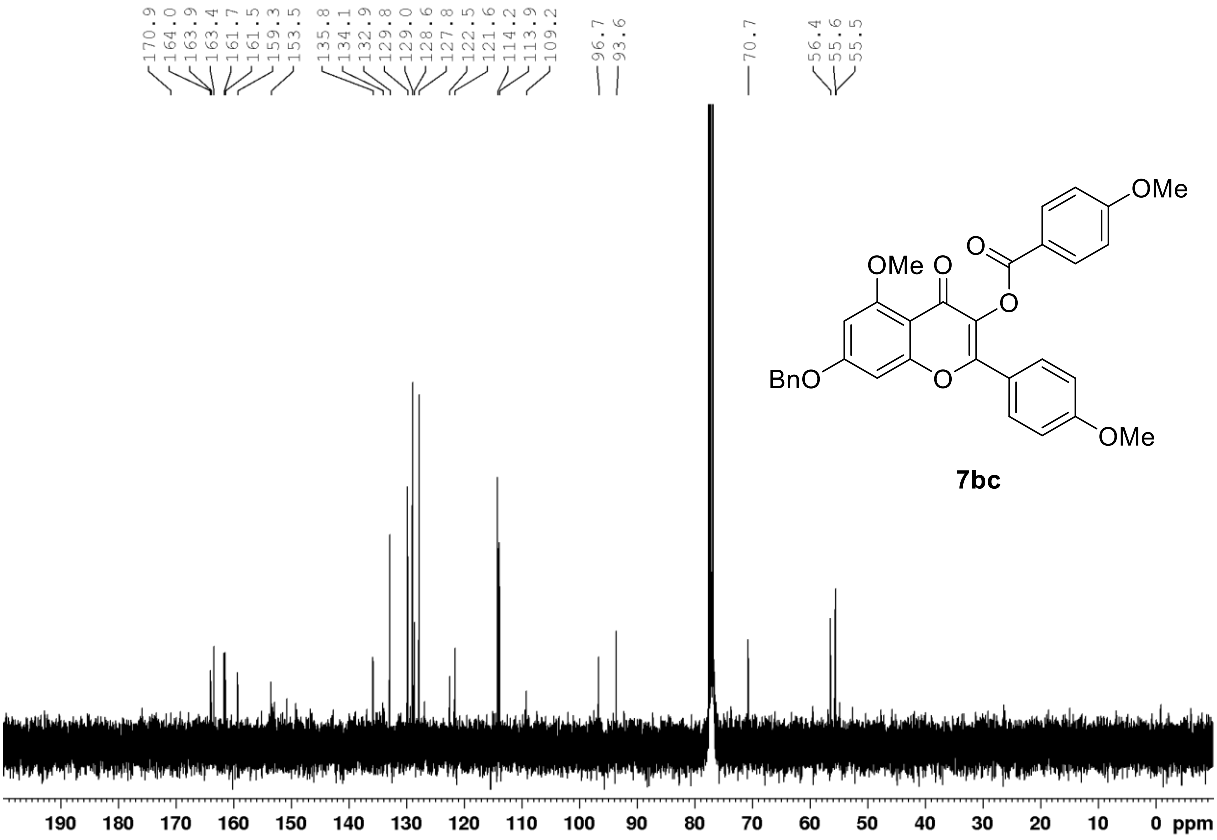

7-(Benzyloxy)-3-hydroxy-5-methoxy-2-(4-methoxyphenyl)-4*H*-chromen-4-one (8bc)

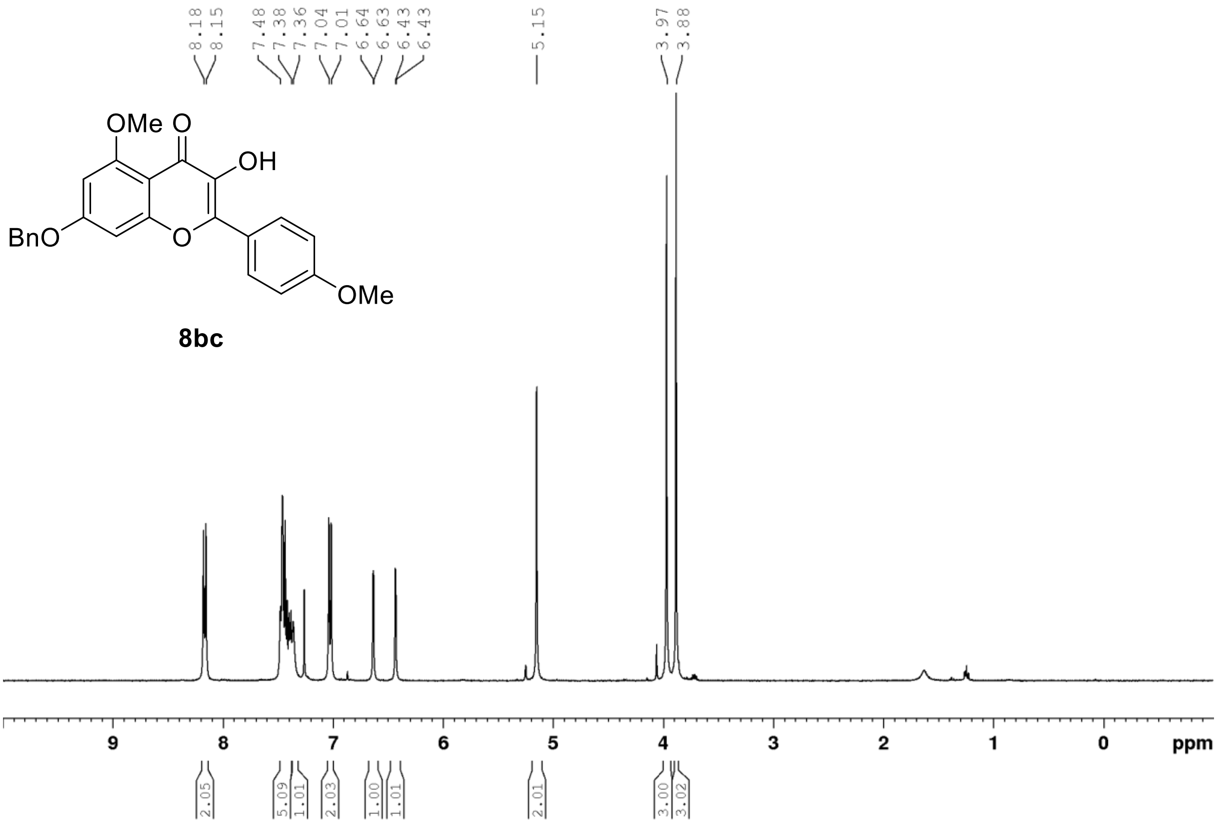

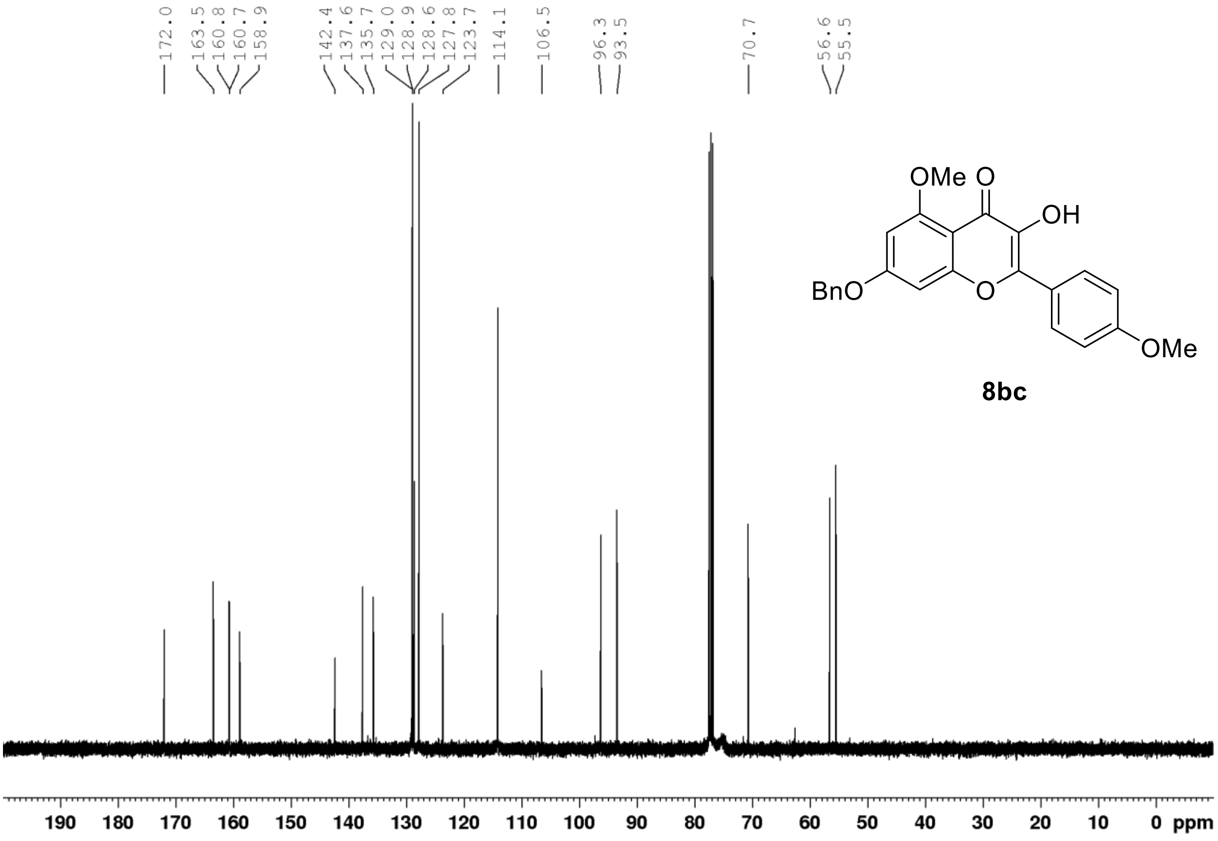

(±)-Methyl (1*R*,2*R*,3*S*,3a*R*,8b*S*)-6-(benzyloxy)-1,8b-dihydroxy-8-methoxy-3a-(4-methoxyphenyl)-3-phenyl-2,3,3a,8b-tetrahydro-1*H*-cyclopenta[*b*]benzofuran-2-carboxylate (9bc)

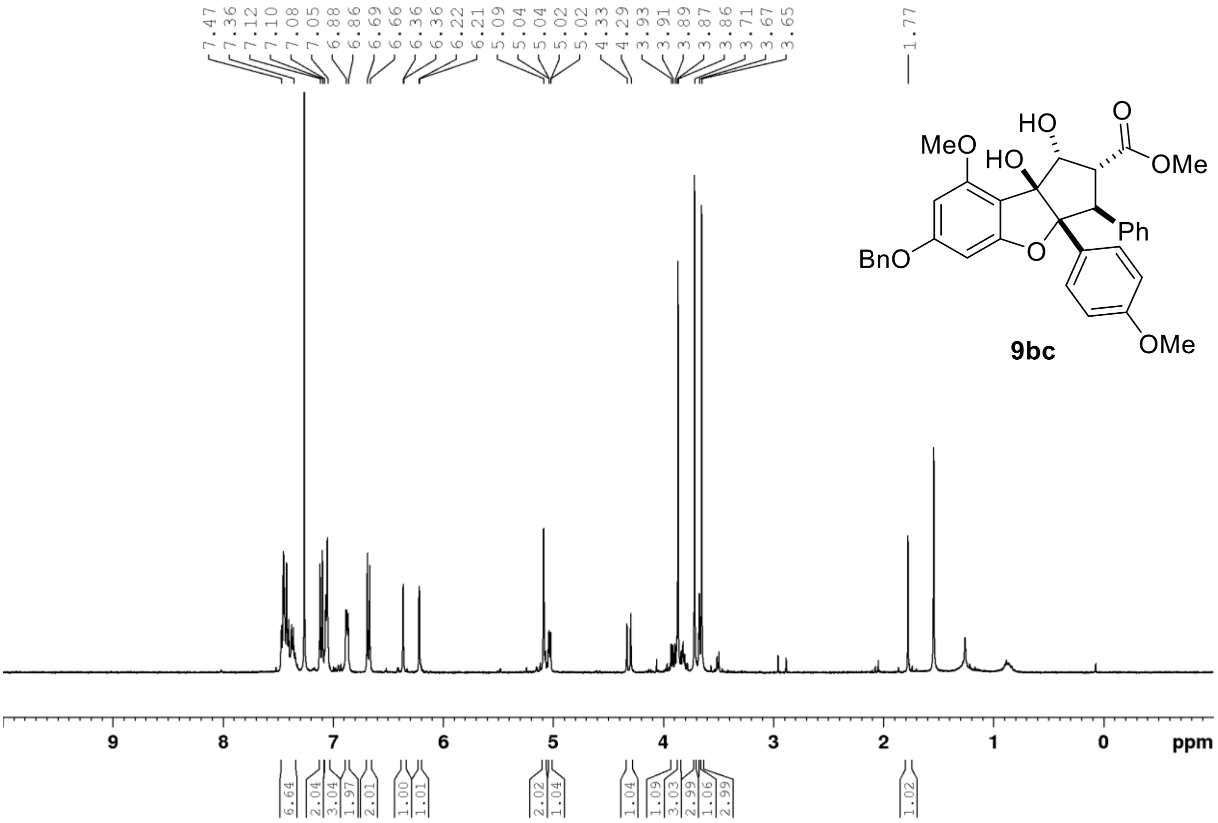

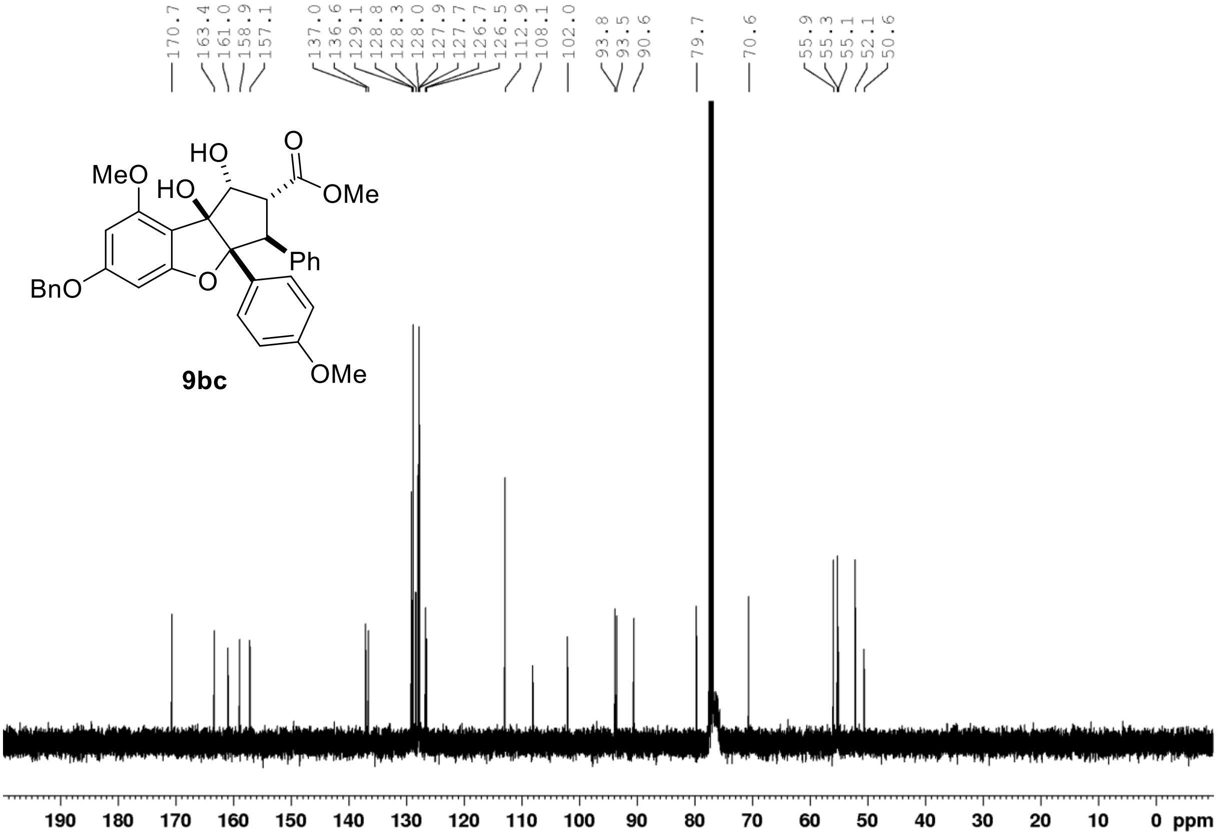

(±)-Methyl (1*R*,2*R*,3*S*,3a*R*,8b*S*)-1,6,8b-trihydroxy-8-methoxy-3a-(4-methoxyphenyl)-3-phenyl-2,3,3a,8b-tetrahydro-1*H*-cyclopenta[*b*]benzofuran-2-carboxylate (10bc)

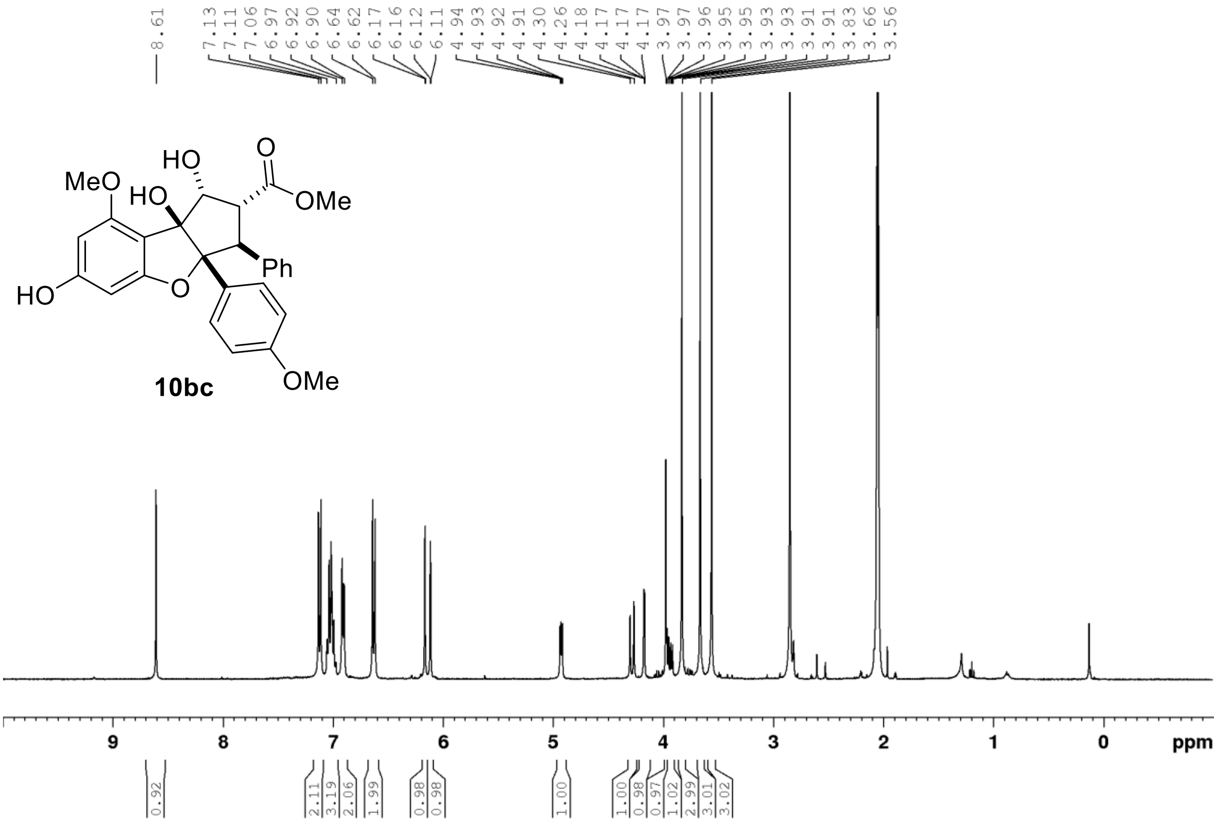

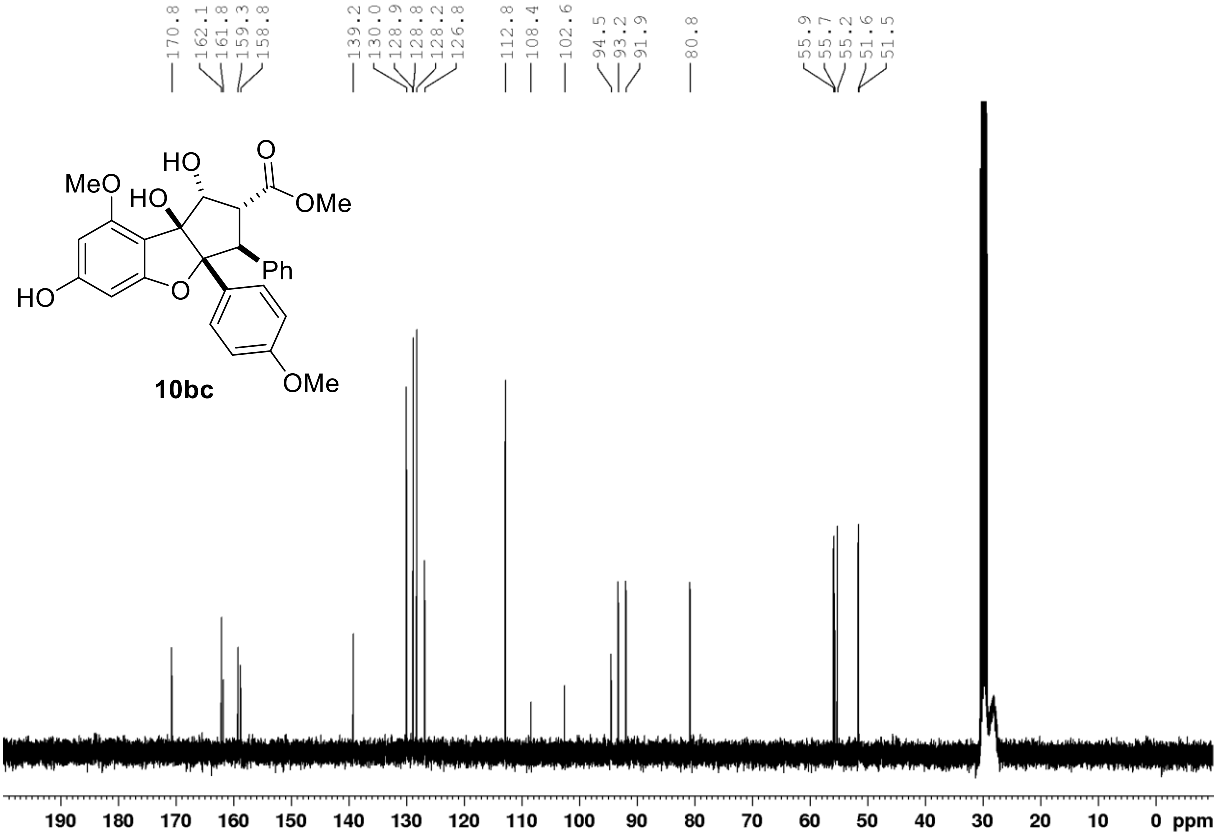

(±)-Methyl (1*R*,2*R*,3*S*,3a*R*,8b*S*)-1,8b-dihydroxy-6,8-dimethoxy-3a-(4-methoxyphenyl)-3-phenyl-2,3,3a,8b-tetrahydro-1*H*-cyclopenta[*b*]benzofuran-2-carboxylate (11bc)

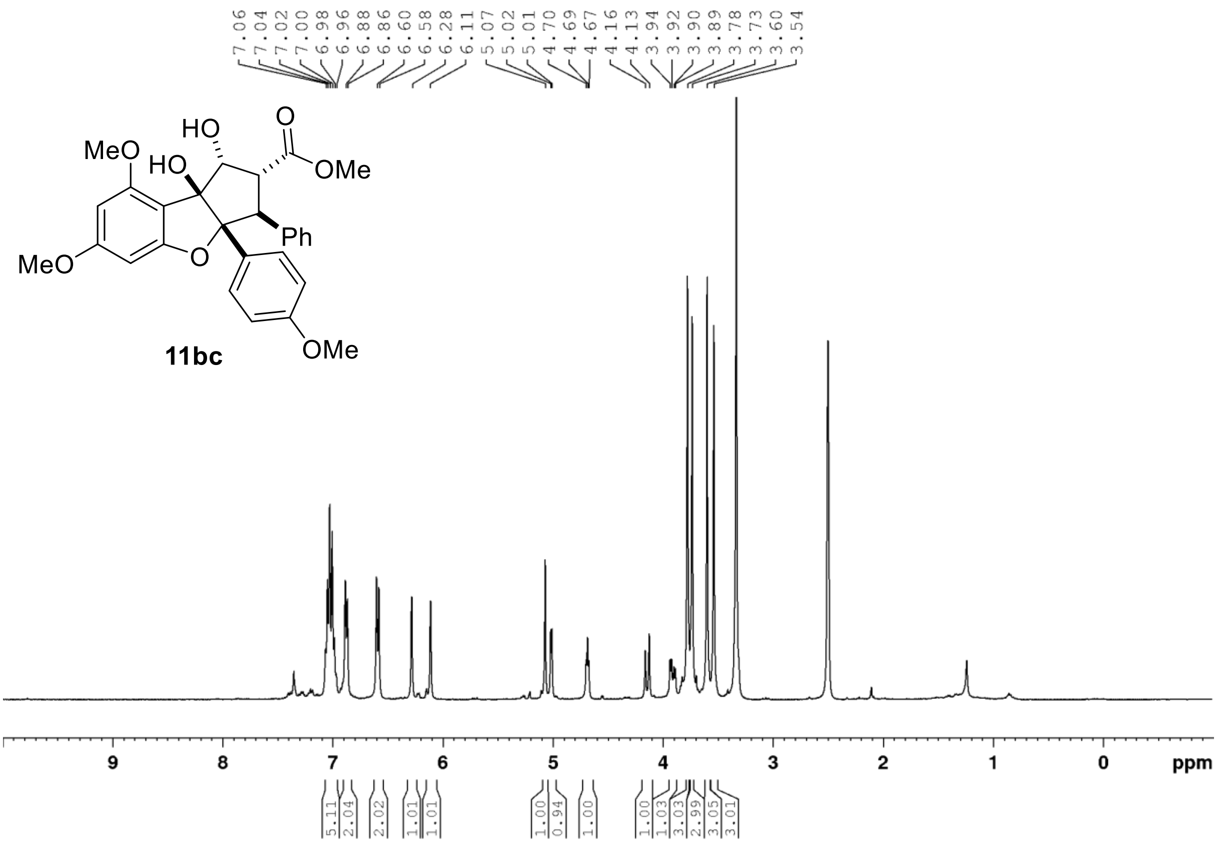

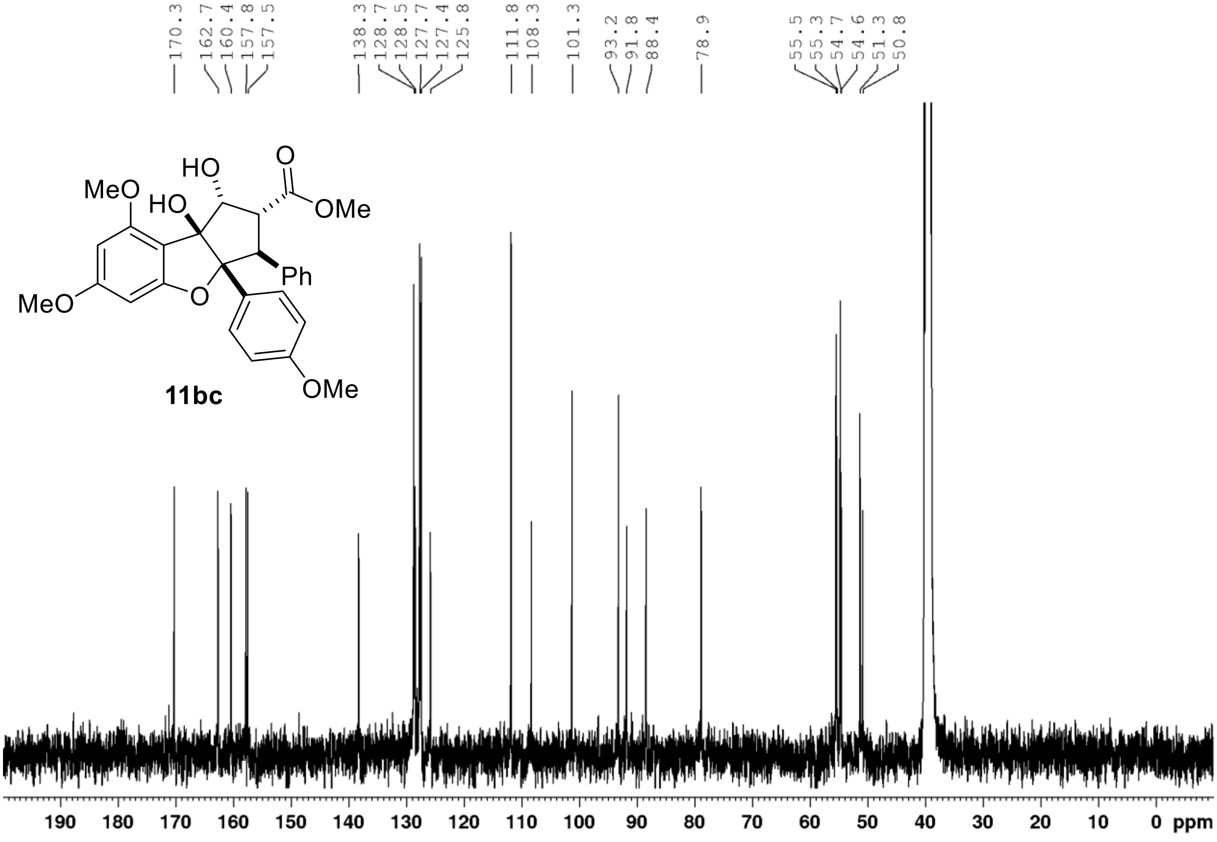

4.6. NMR spectroscopic data for the synthesis of 9c

(*E*)-1-(2,4-Difluoro-6-hydroxyphenyl)-3-(4-methoxyphenyl)prop-2-en-1-one (12c)

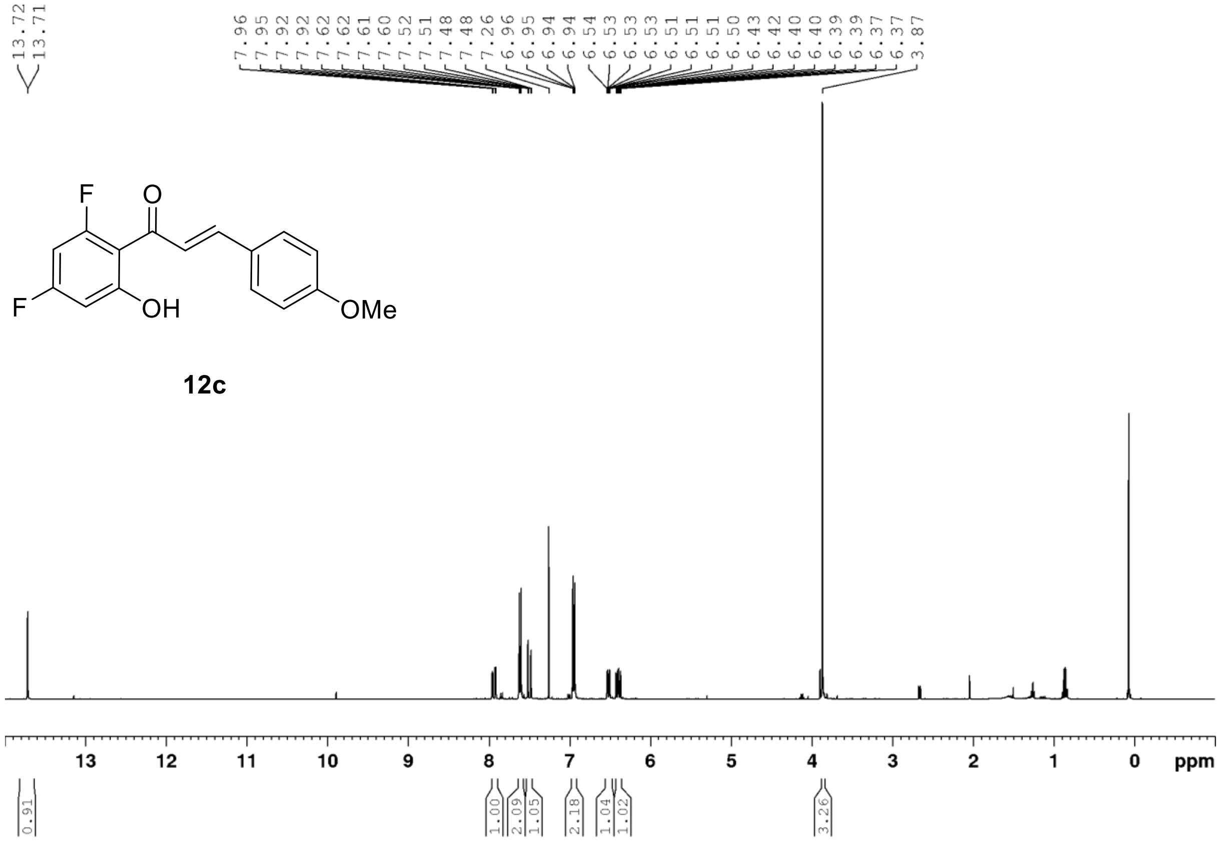

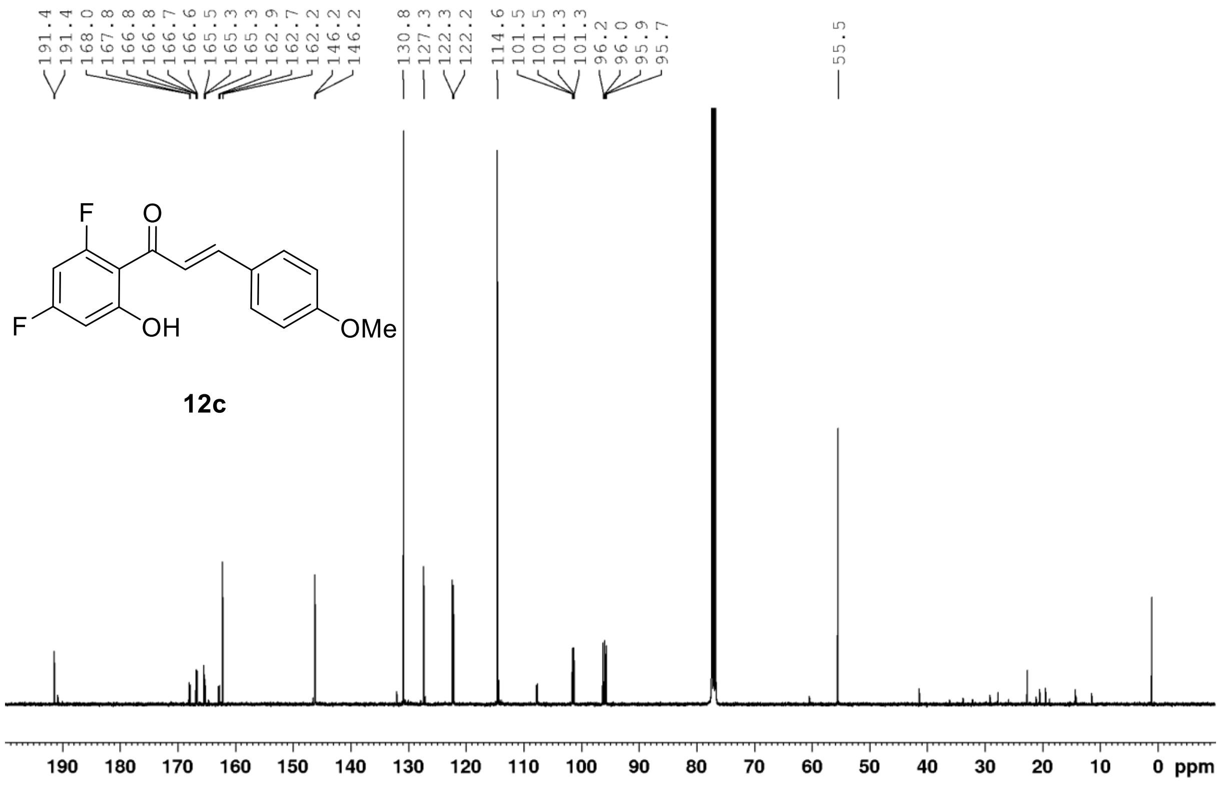

5,7-Difluoro-3-hydroxy-2-(4-methoxyphenyl)-4*H*-chromen-4-one (8c)

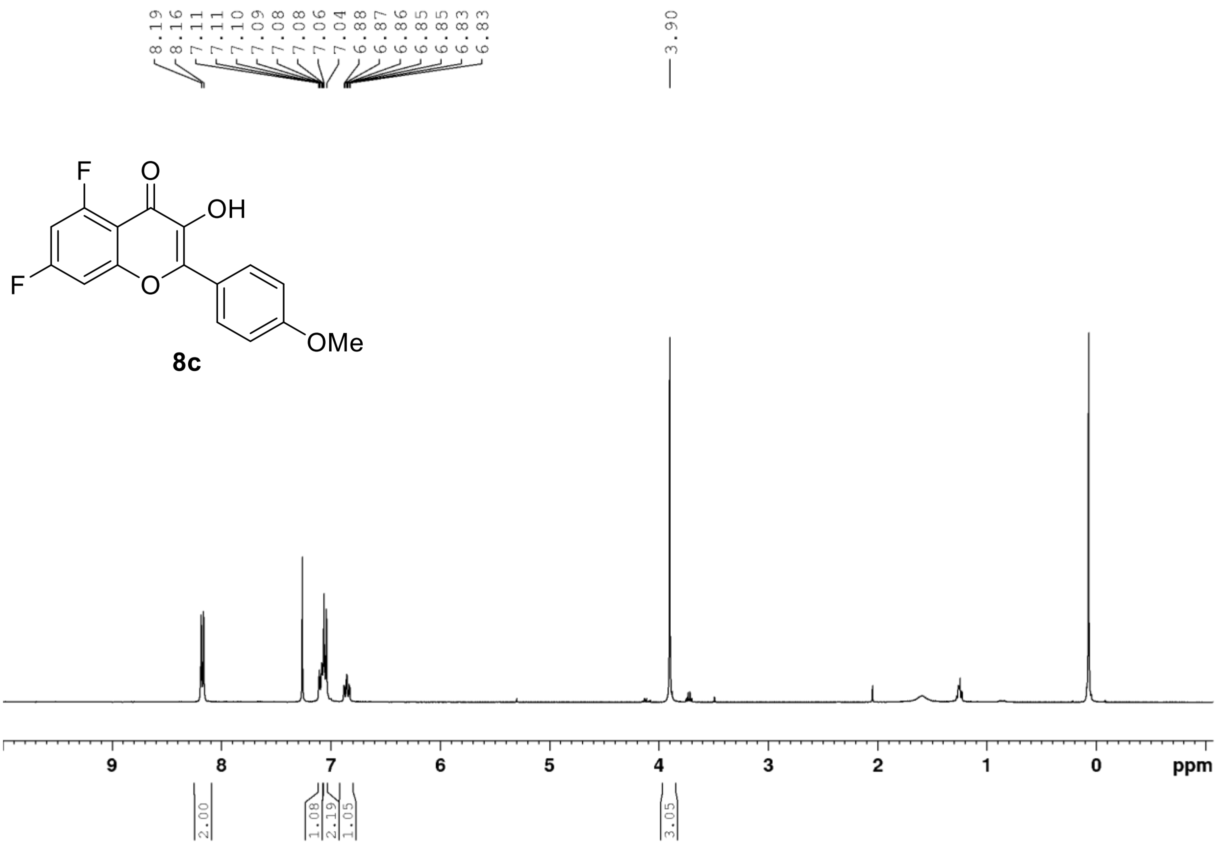

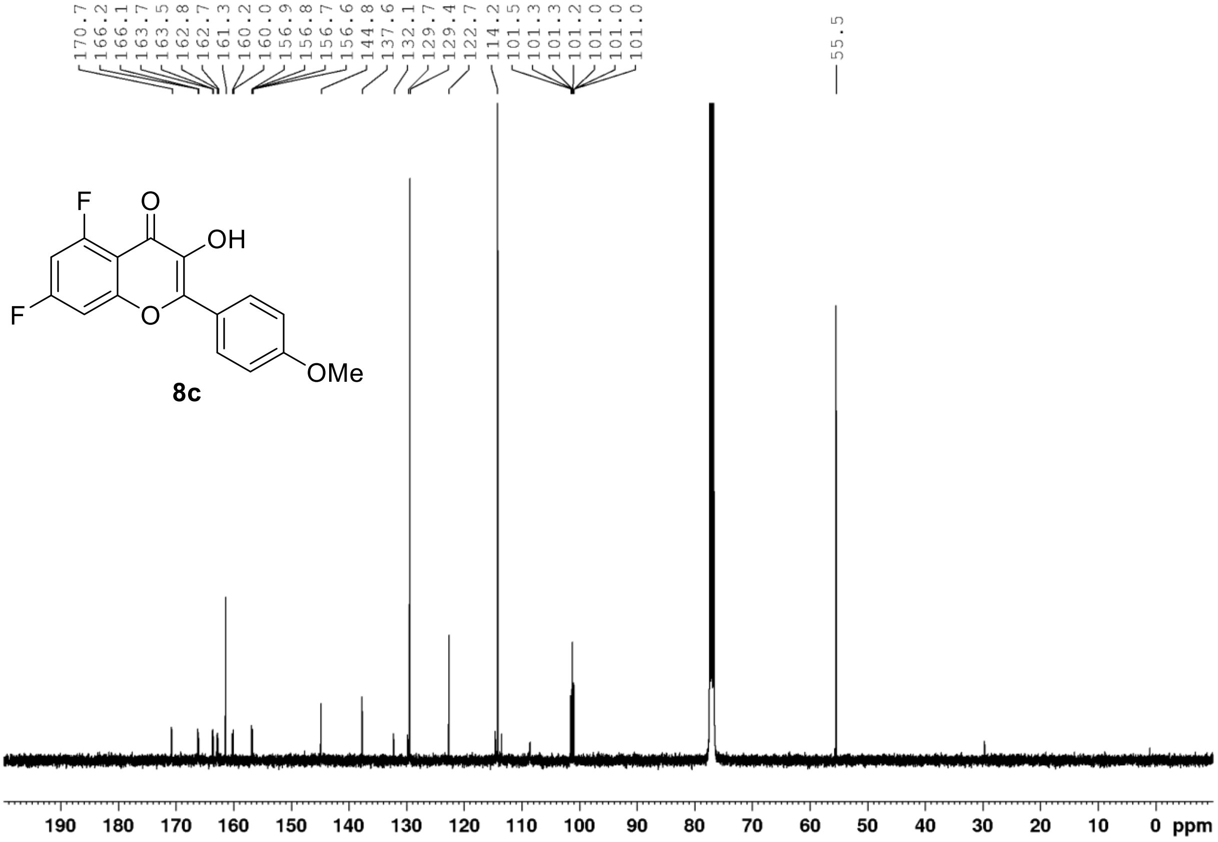

(±)-Methyl (1*R*,2*R*,3*S*,3a*R*,8b*S*)-6,8-difluoro-1,8b-dihydroxy-3a-(4-methoxyphenyl)-3-phenyl-2,3,3a,8b-tetrahydro-1*H*-cyclopenta[*b*]benzofuran-2-carboxylate (9c)

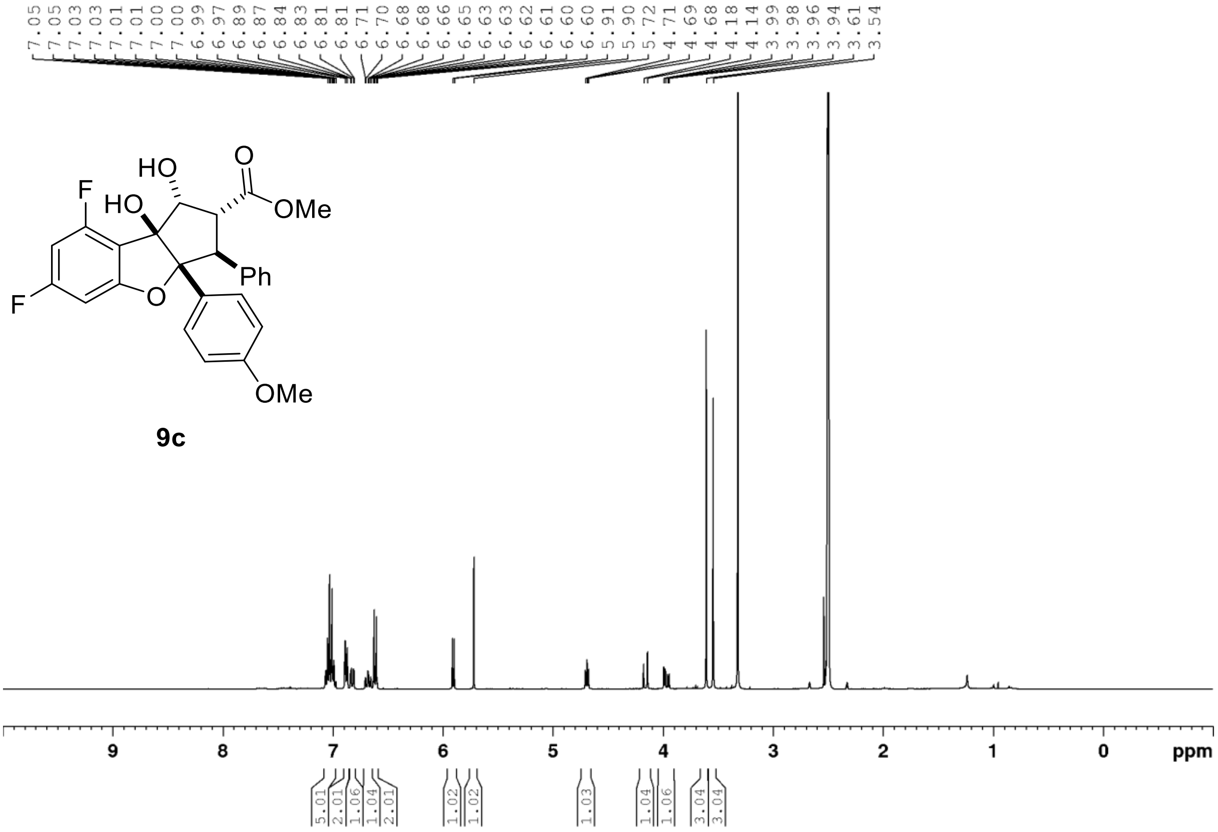

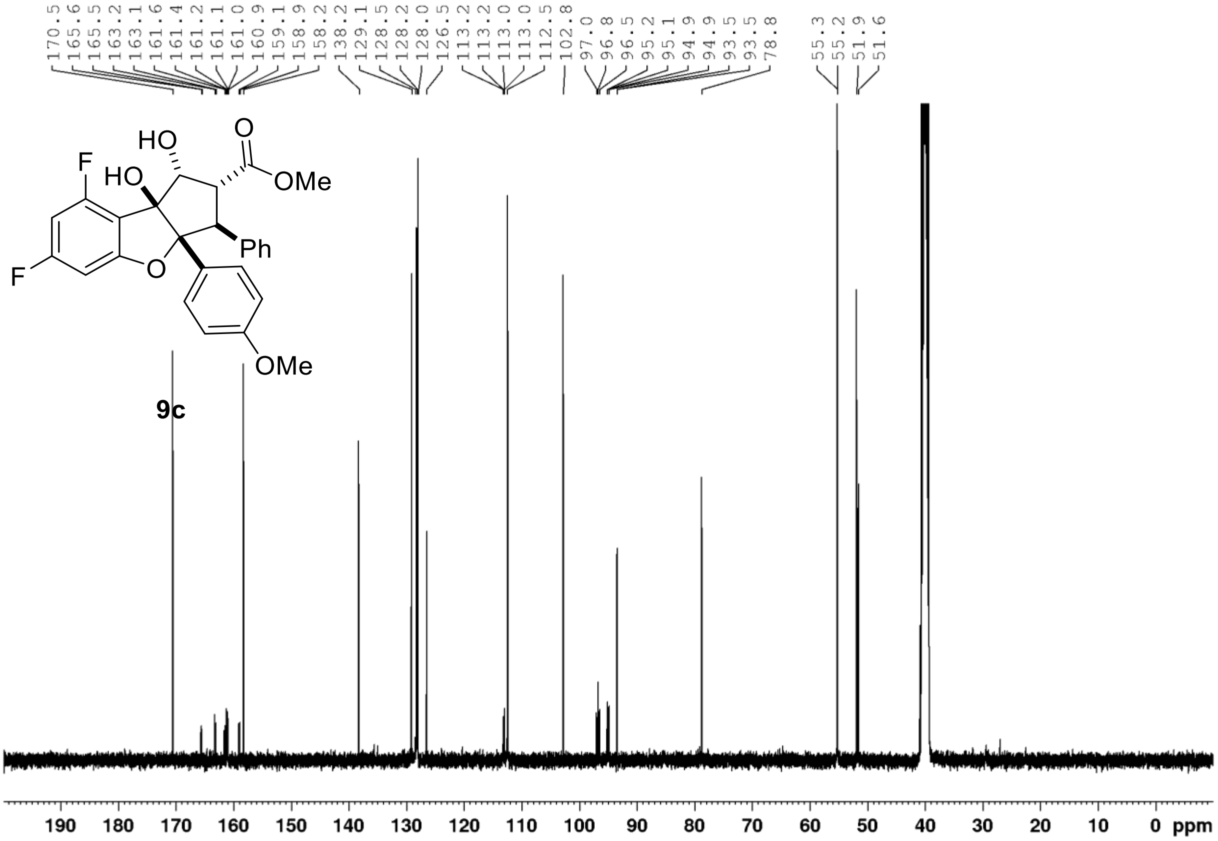

4.7. NMR spectroscopic data for the synthesis of 9da

(*E*)-1-(2,4-Dichloro-6-hydroxyphenyl)-3-(4-methoxyphenyl)prop-2-en-1-one (12da)

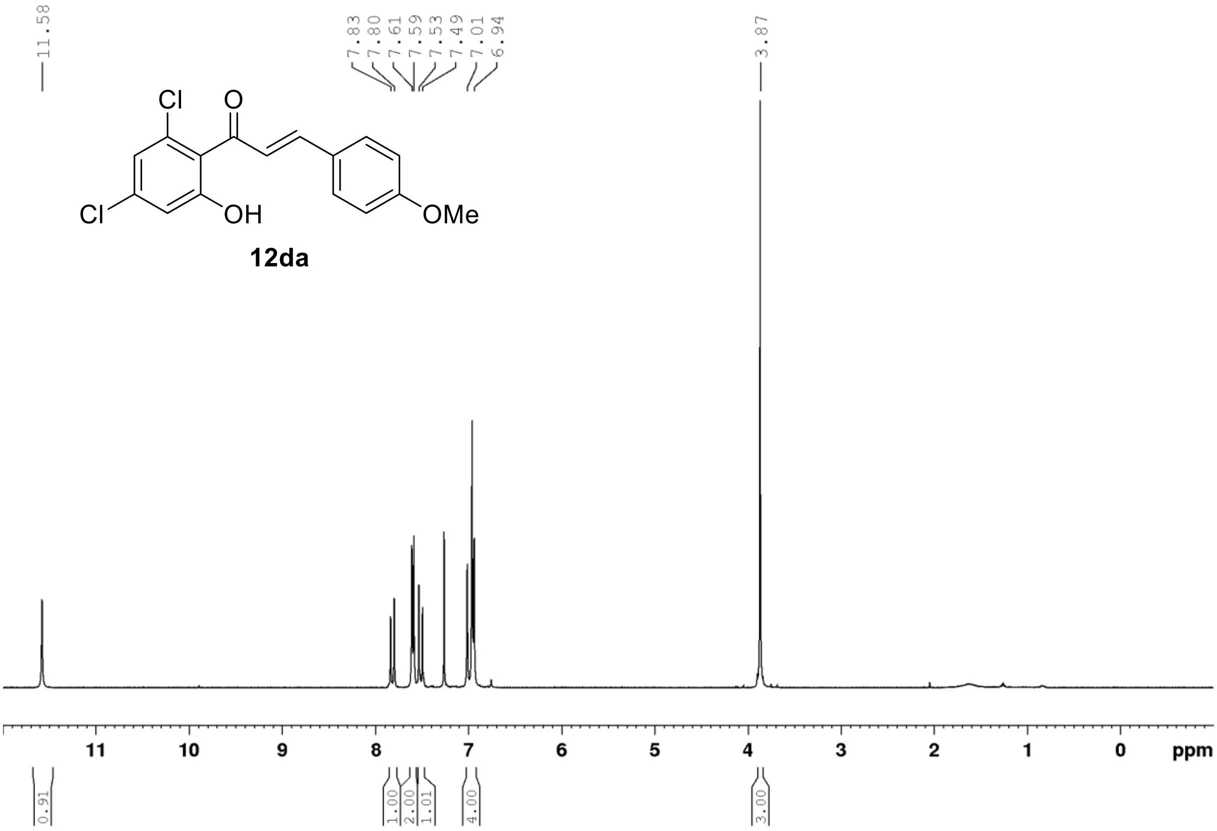

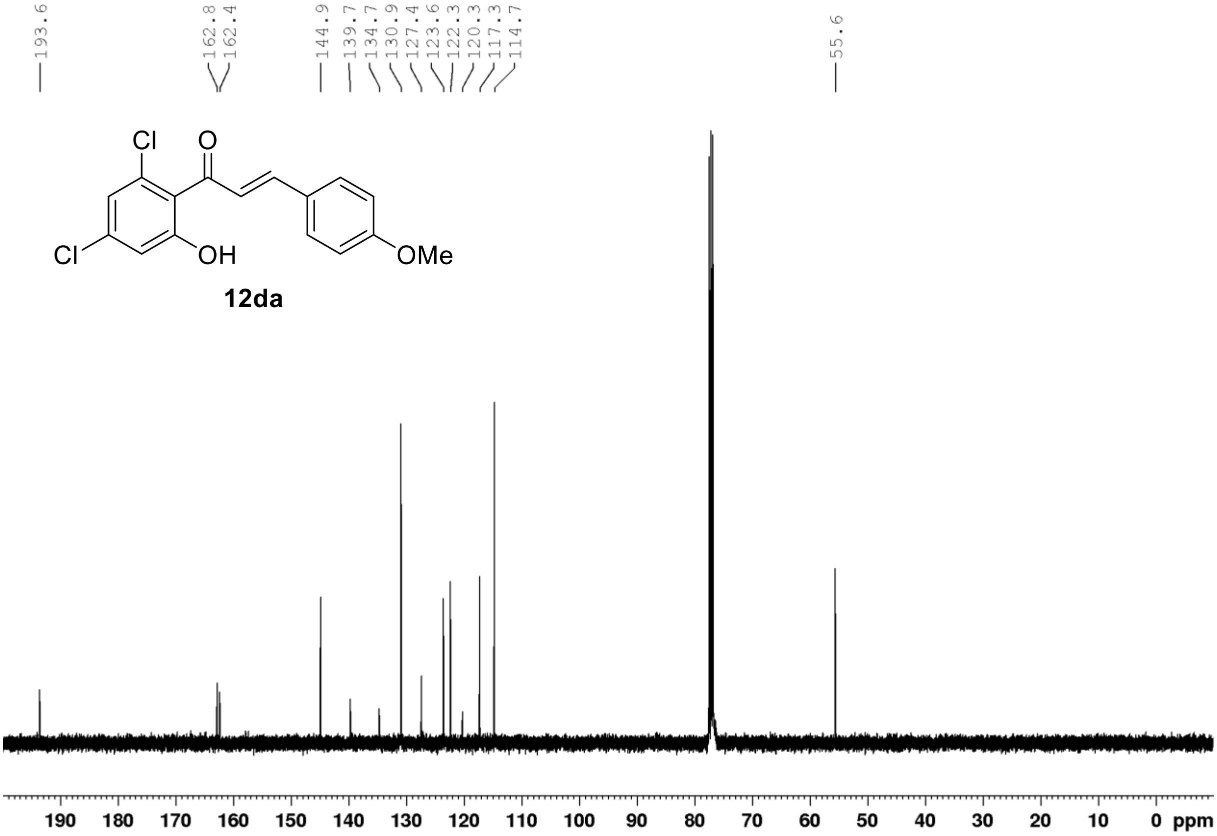

5,7-Dichloro-3-hydroxy-2-(4-methoxyphenyl)-4*H*-chromen-4-one (8da)

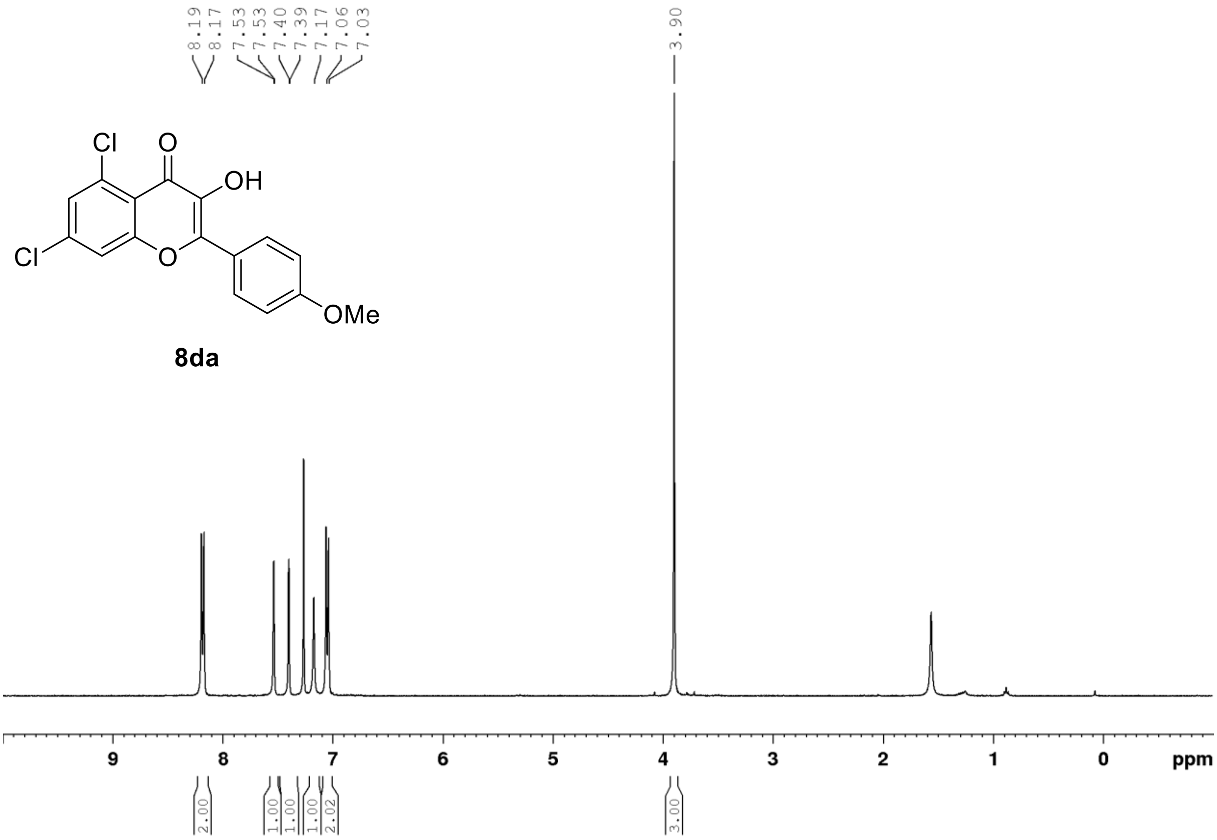

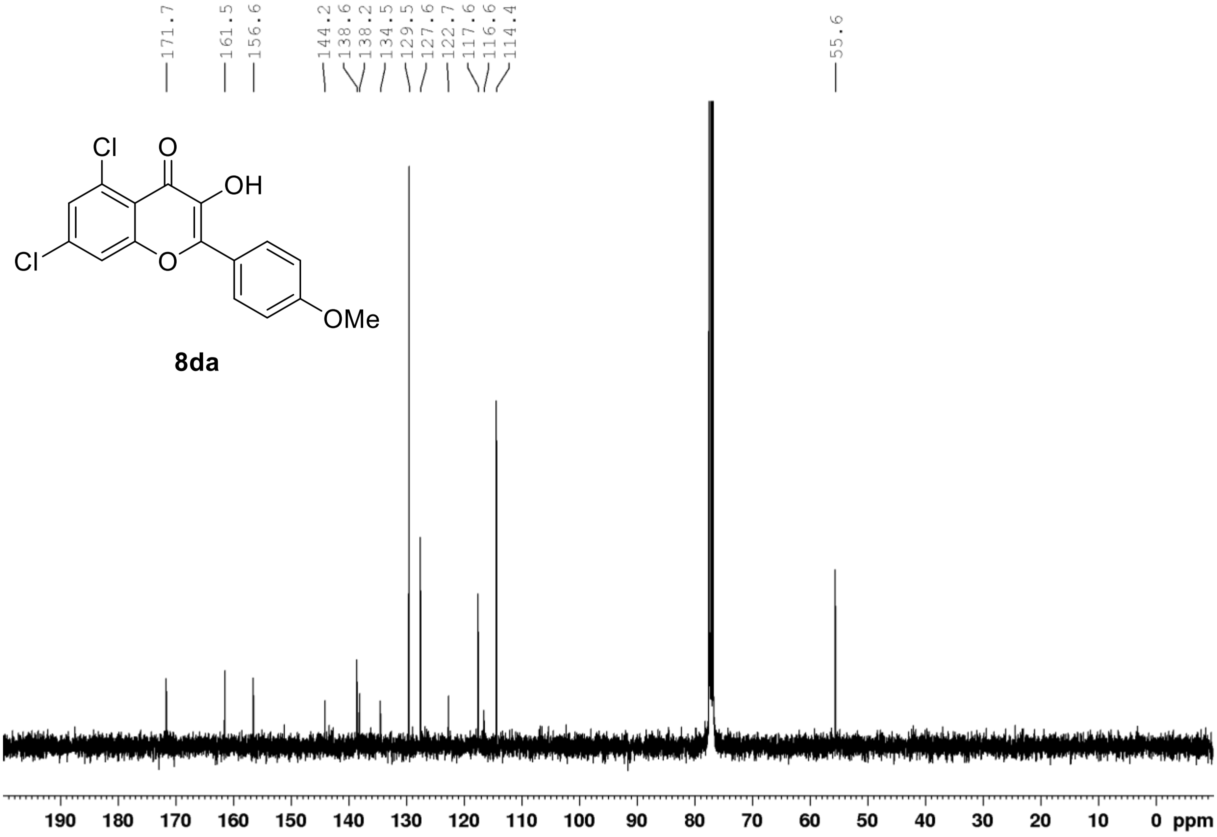

(±)-Methyl (1*R*,2*R*,3*S*,3a*R*,8b*S*)-6,8-dichloro-1,8b-dihydroxy-3a-(4-methoxyphenyl)-3-phenyl-2,3,3a,8b-tetrahydro-1*H*-cyclopenta[*b*]benzofuran-2-carboxylate (9da)

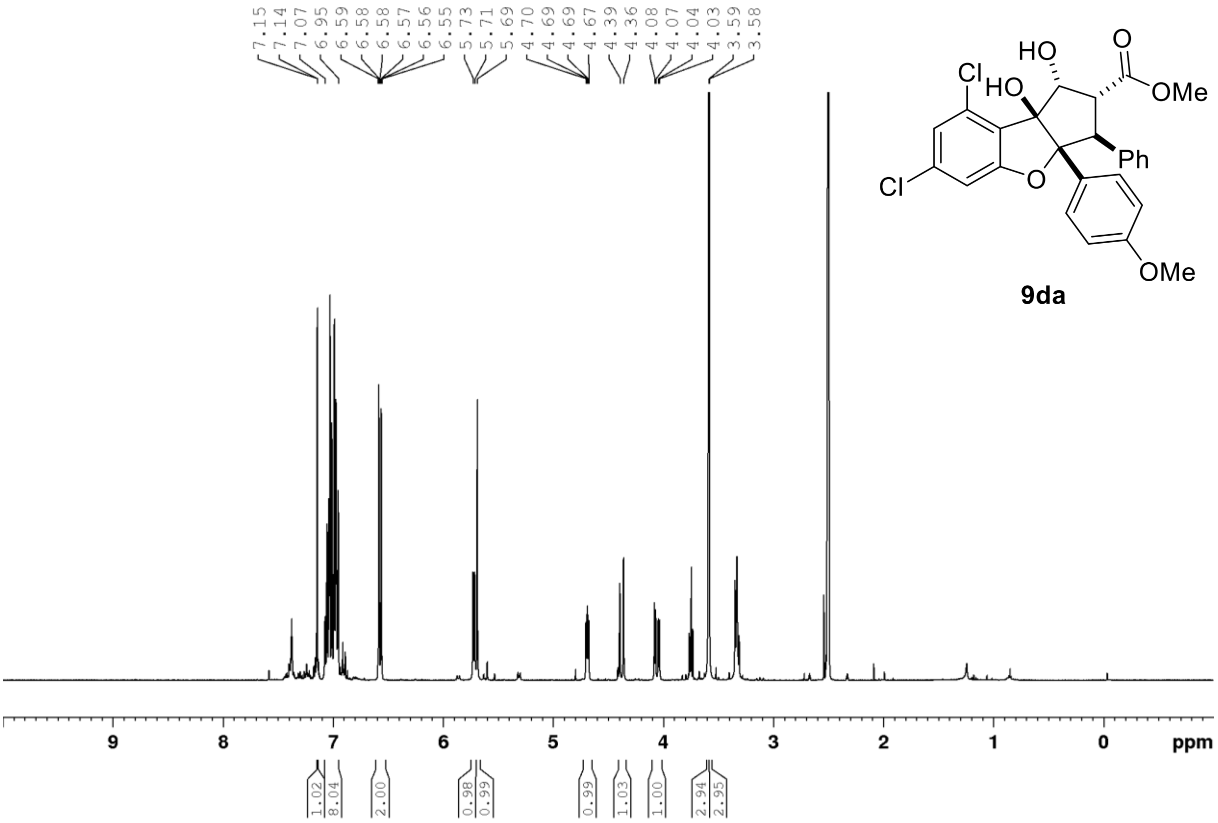

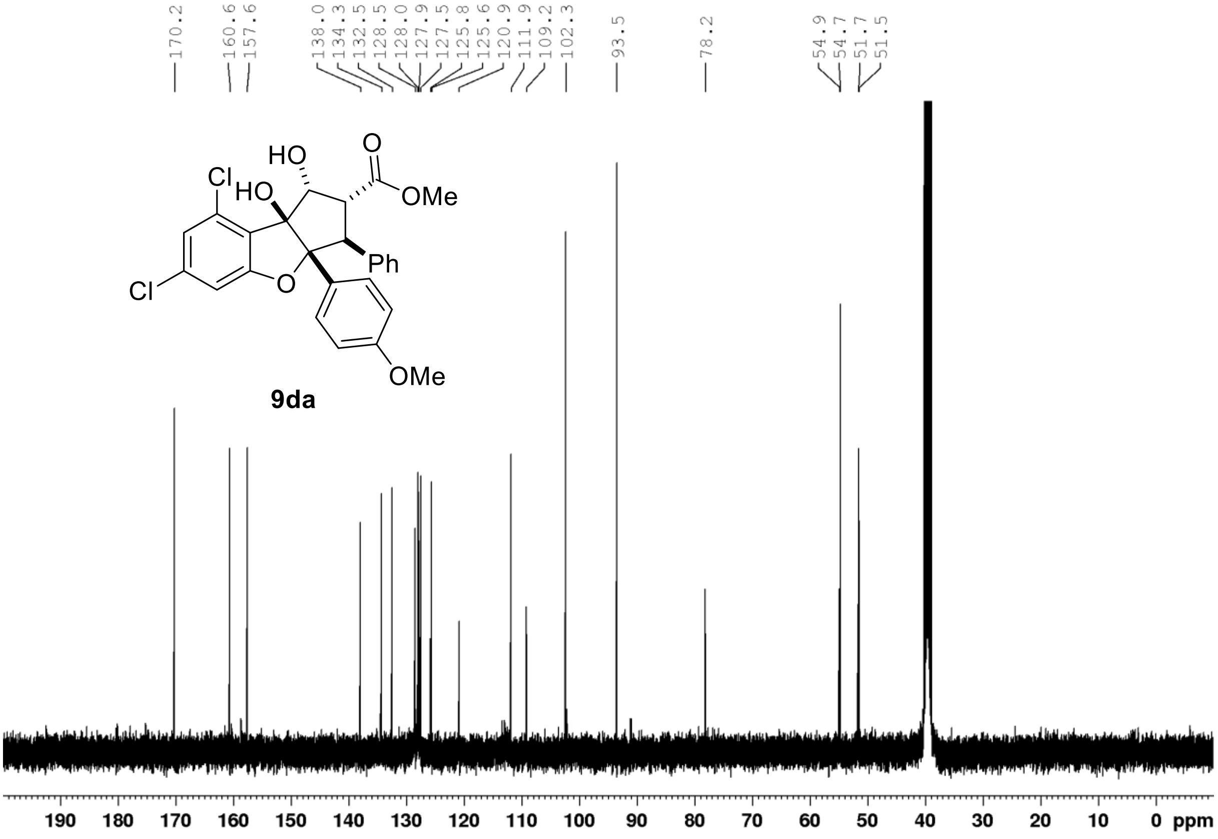

4.8. NMR spectroscopic data for the synthesis of 9db

(*E*)-3-(4-Bromophenyl)-1-(2,4-dichloro-6-hydroxyphenyl)prop-2-en-1-one (12db)

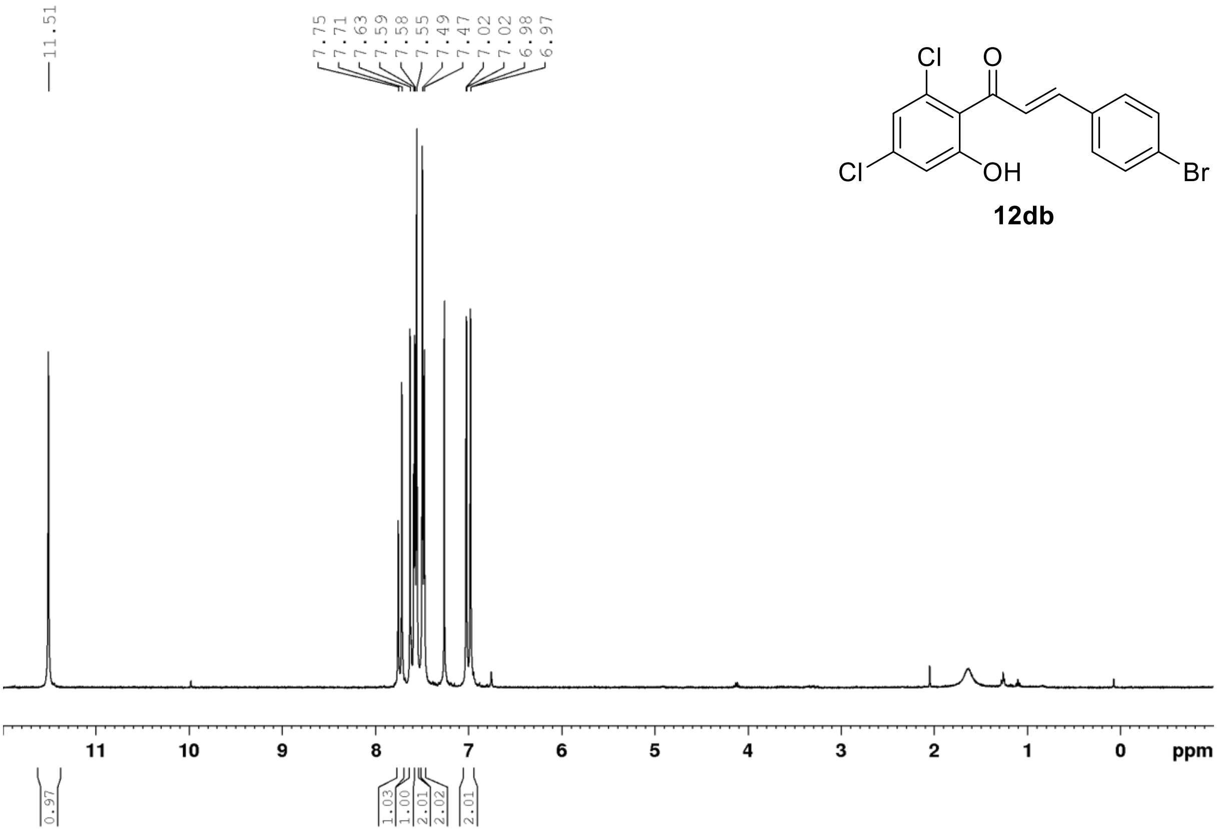

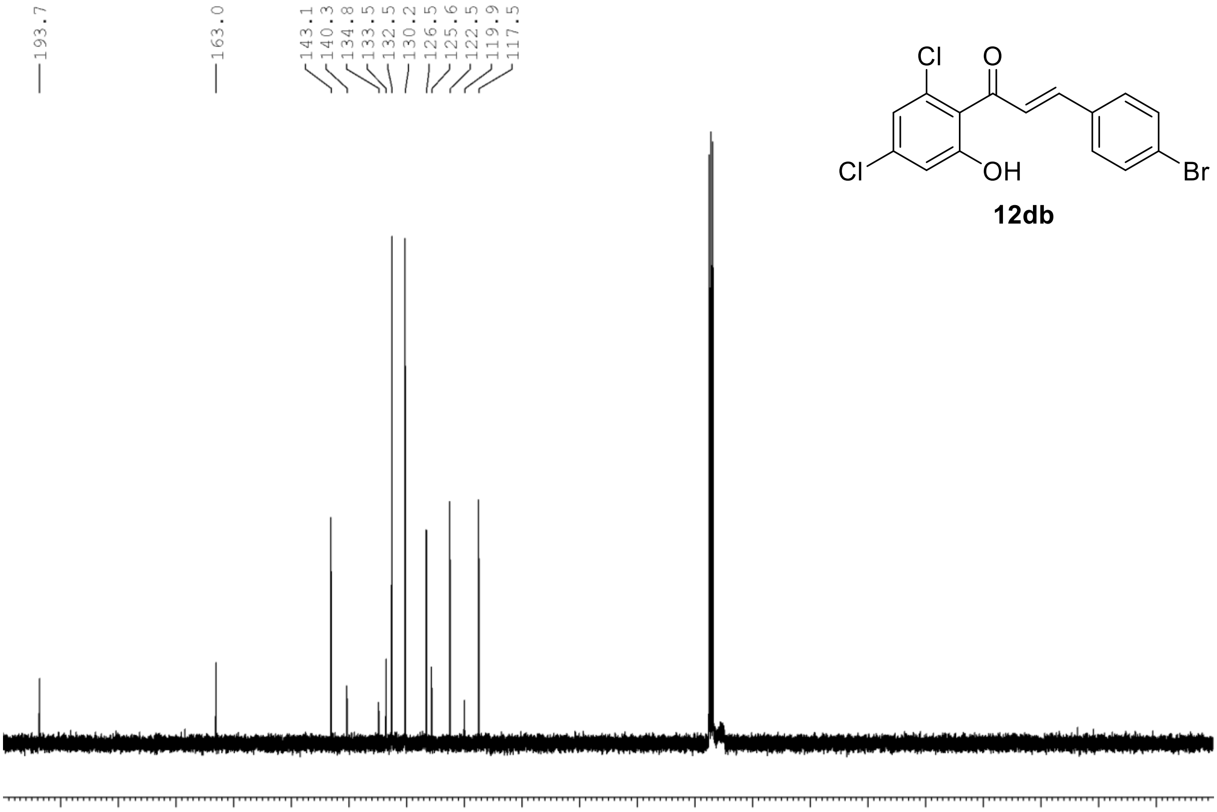

2-(4-Bromophenyl)-5,7-dichloro-3-hydroxy-4*H*-chromen-4-one (8db)

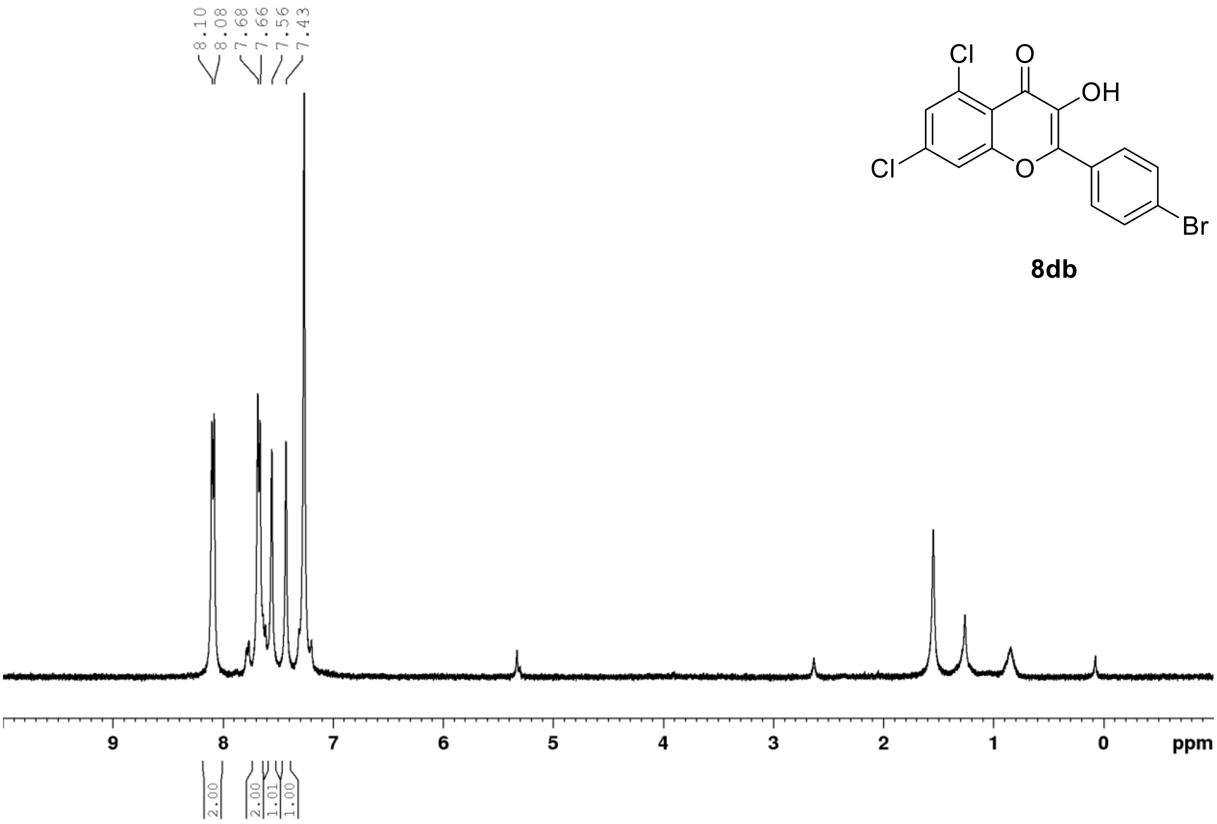

8db

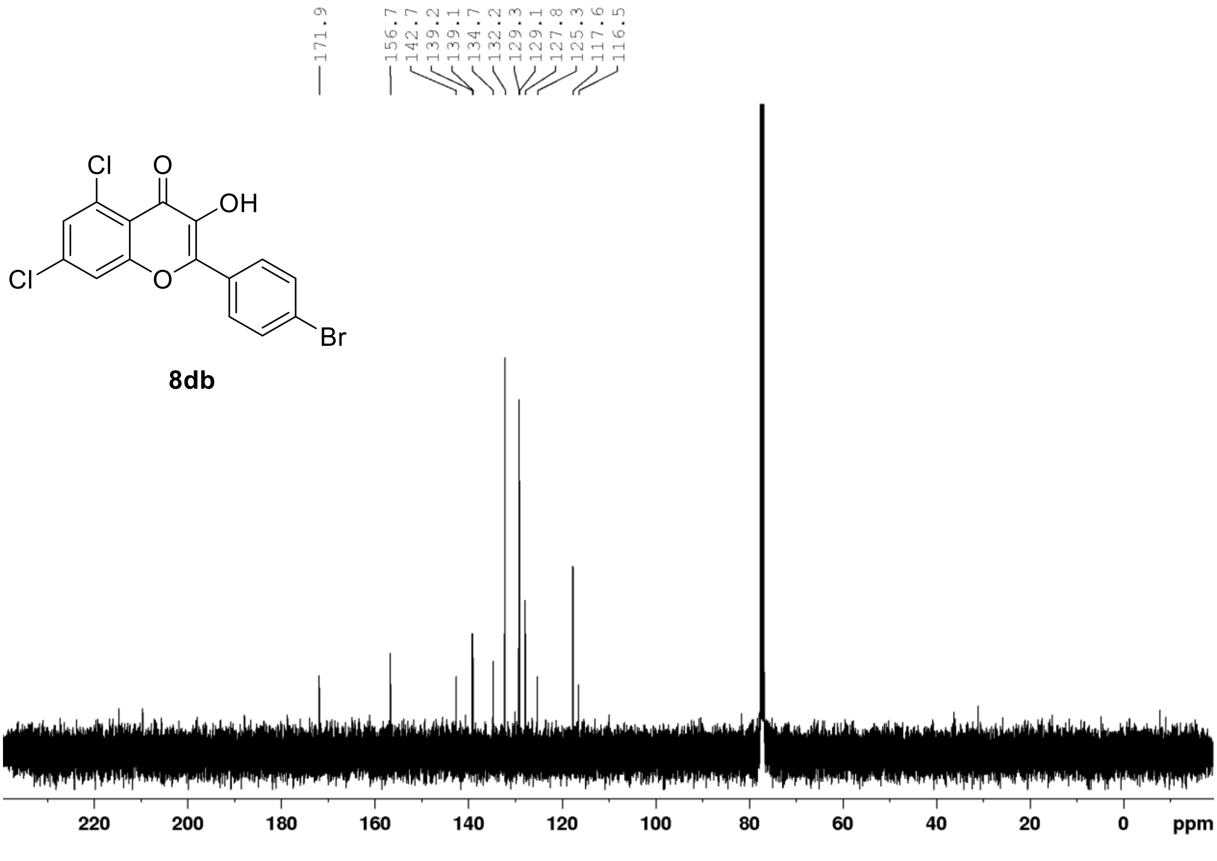

8db

(±)-Methyl (1*R*,2*R*,3*S*,3a*R*,8b*S*)-3a-(4-bromophenyl)-6,8-dichloro-1,8b-dihydroxy-3-phenyl-2,3,3a,8b-tetrahydro-1*H*-cyclopenta[*b*]benzofuran-2-carboxylate (9db)

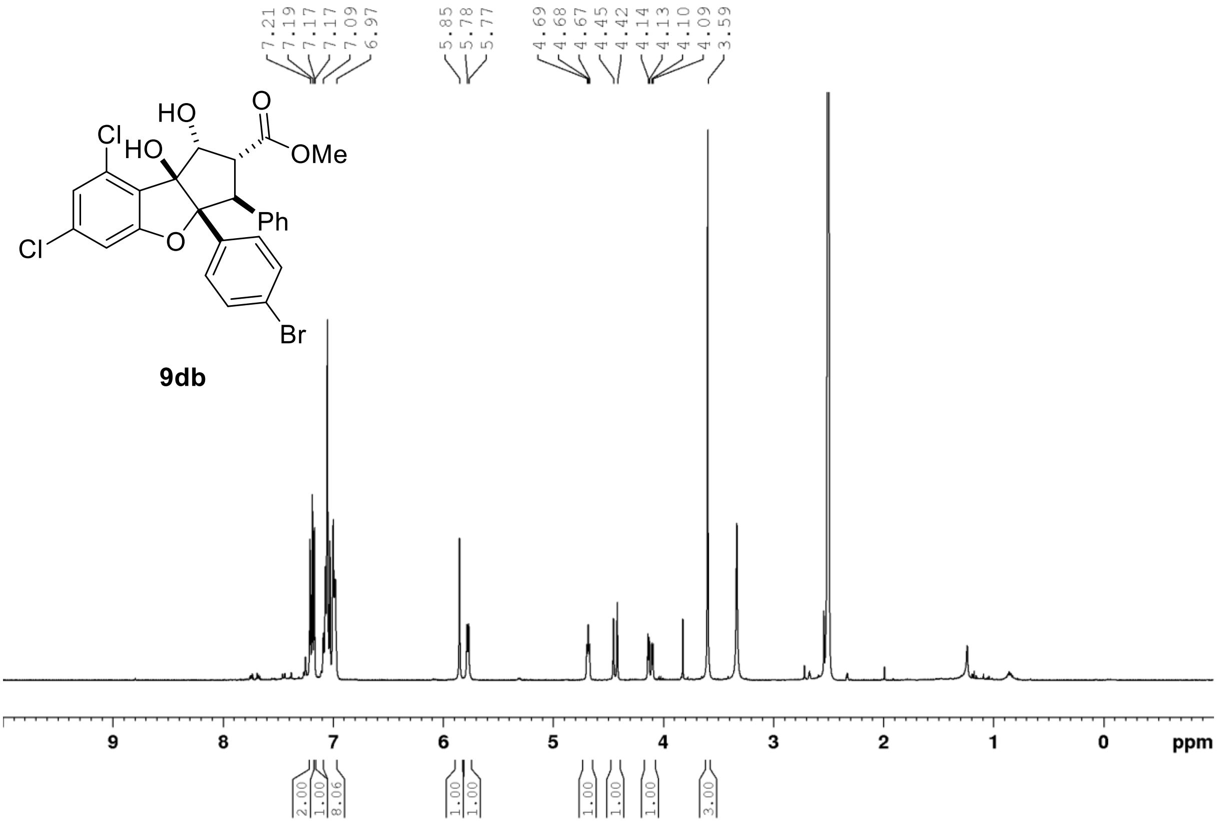

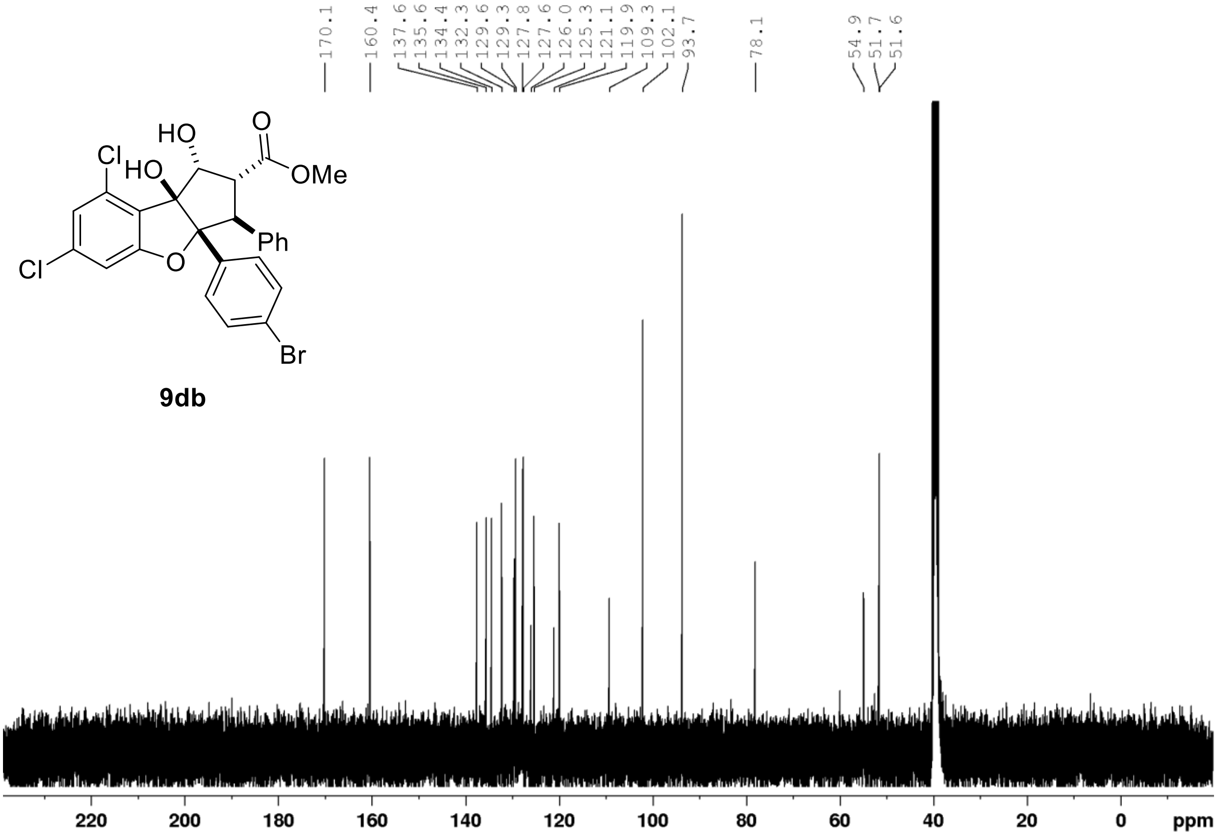

4.9. NMR spectroscopic data for the synthesis of 9e

(*E*)-1-(2,4-Dibromo-6-hydroxyphenyl)-3-(4-methoxyphenyl)prop-2-en-1-one (12e)

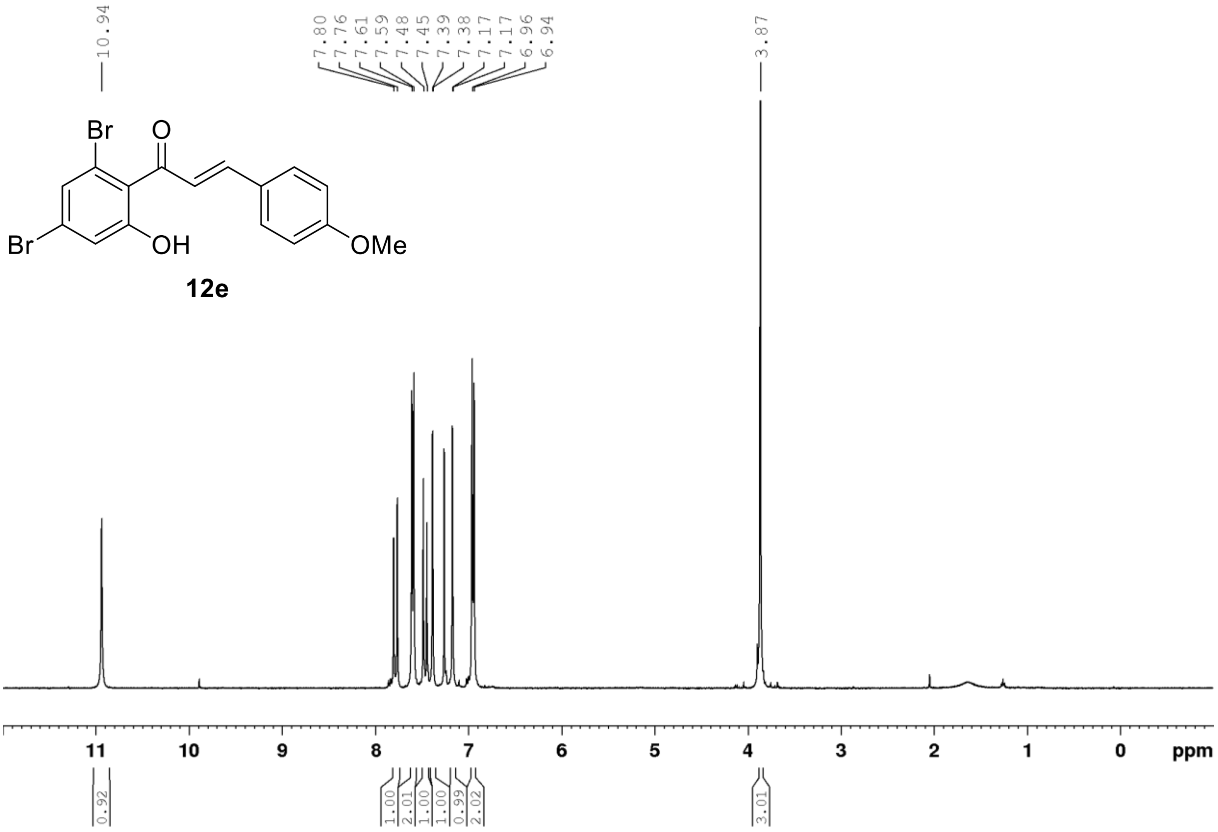

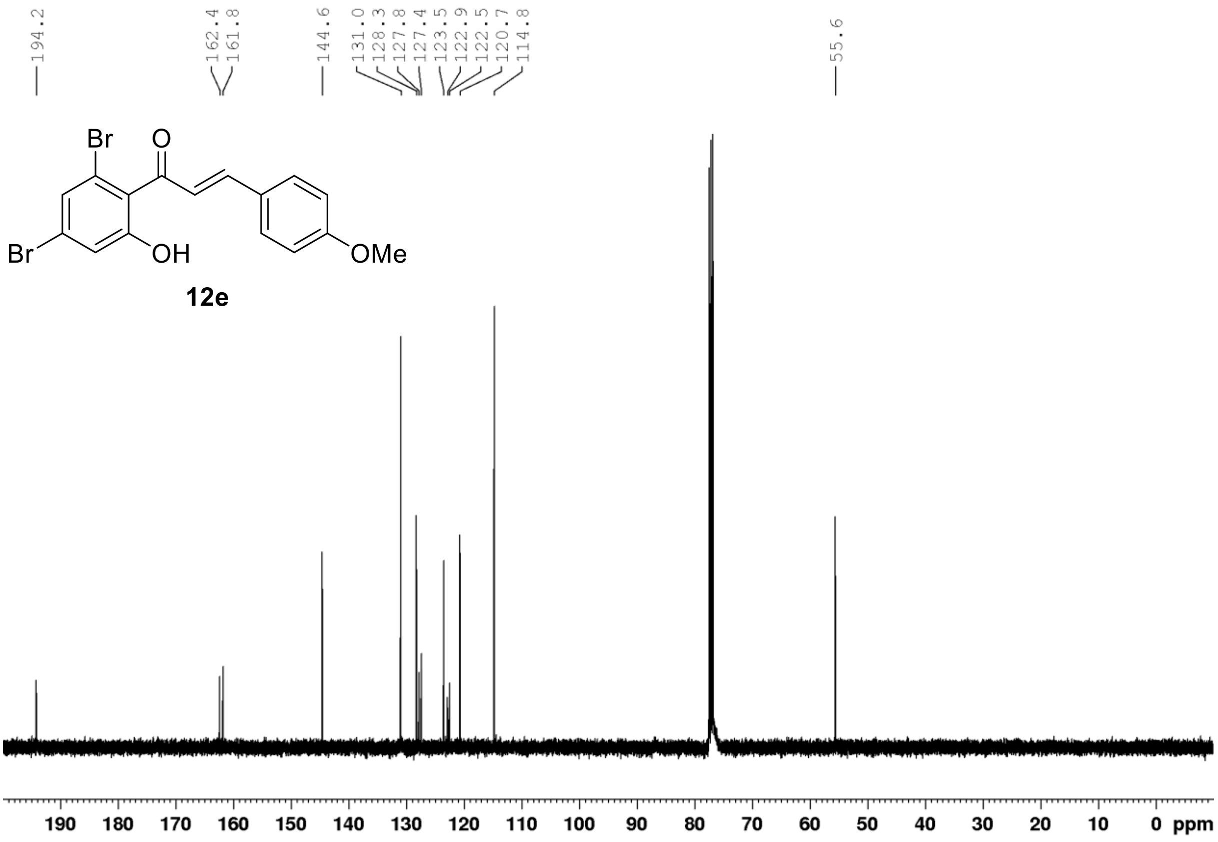

5,7-Dibromo-3-hydroxy-2-(4-methoxyphenyl)-4*H*-chromen-4-one (8e)

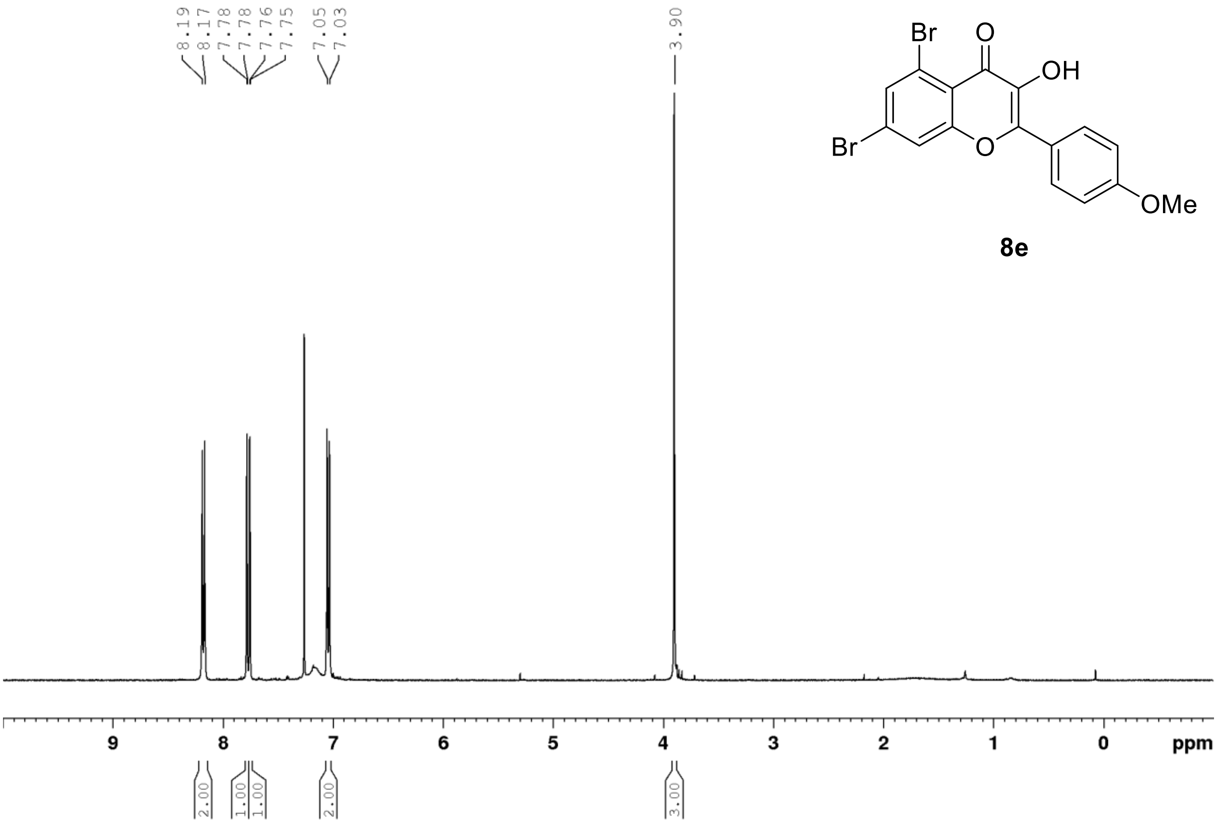

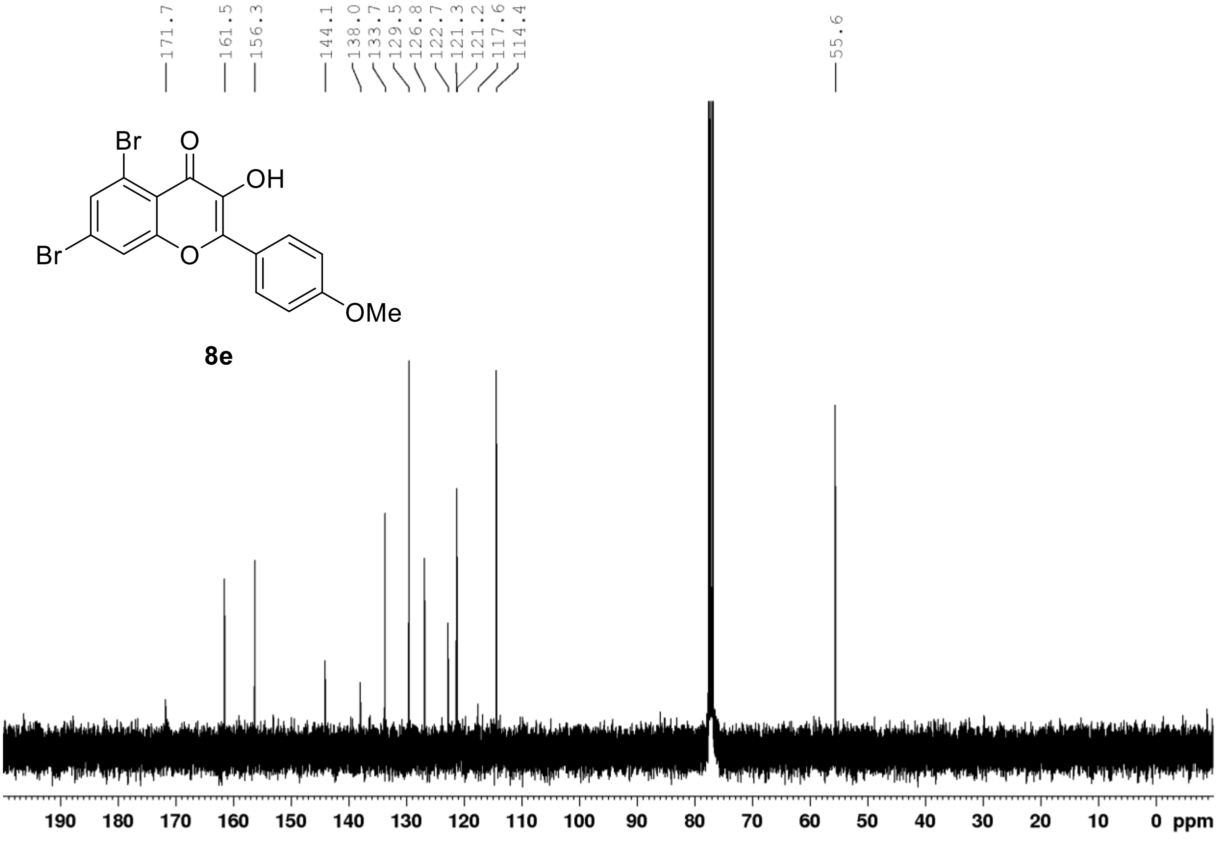

(±)-Methyl (1*R*,2*R*,3*S*,3a*R*,8b*S*)-6,8-dibromo-1,8b-dihydroxy-3a-(4-methoxyphenyl)-3-phenyl-2,3,3a,8b-tetrahydro-1*H*-cyclopenta[*b*]benzofuran-2-carboxylate (9e)

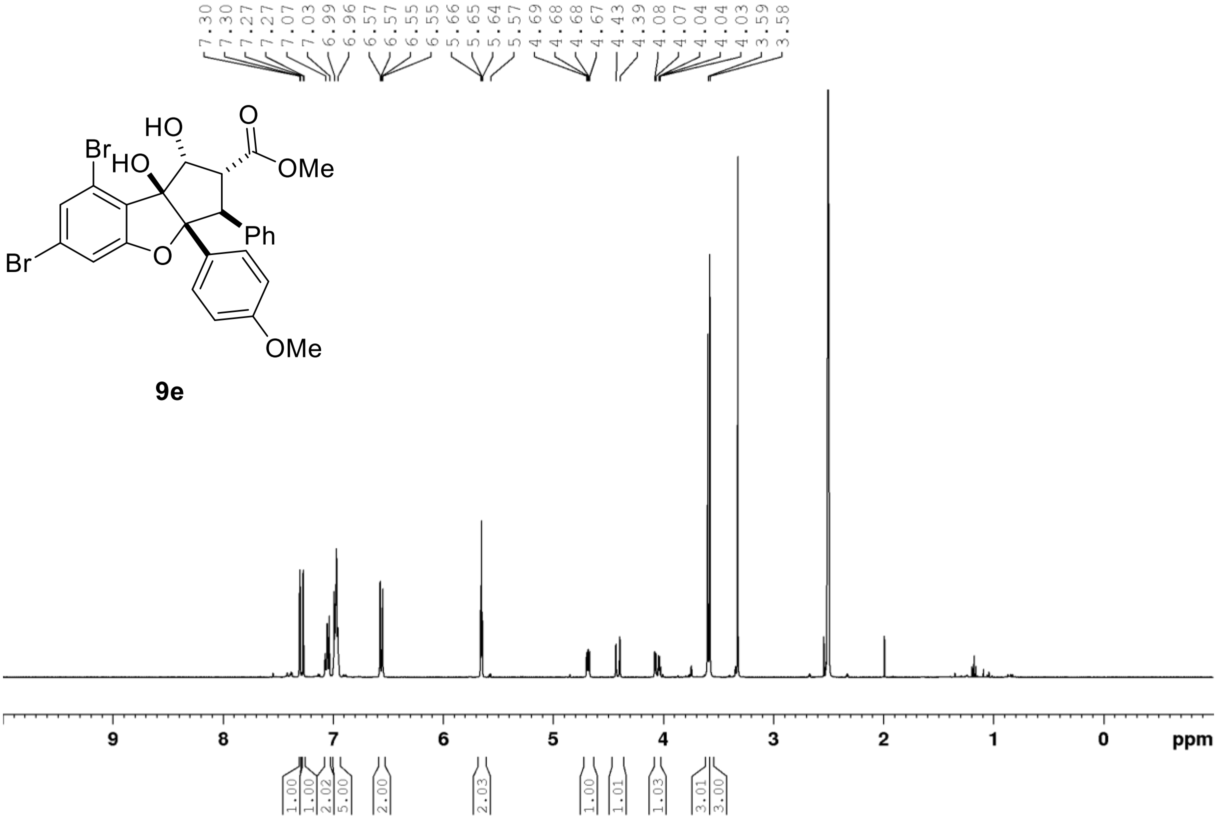

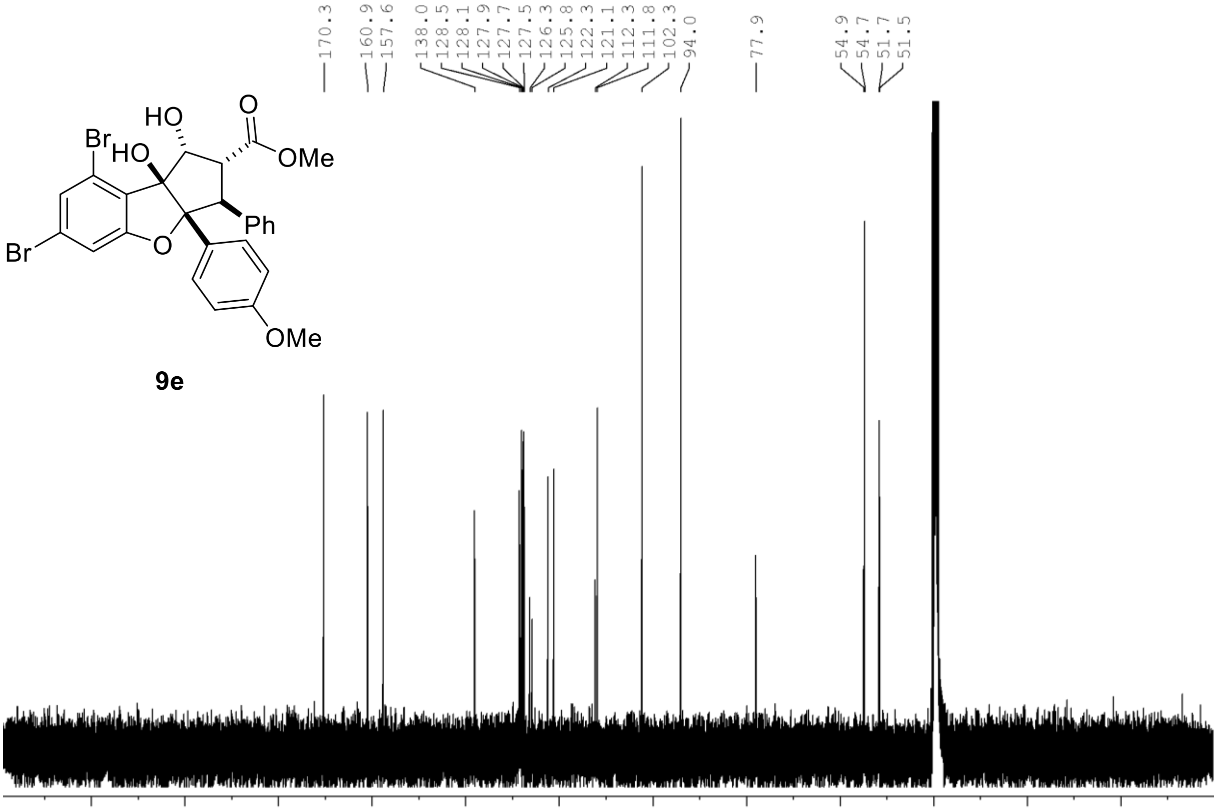

4.10. NMR spectroscopic data for the synthesis of 9f

(*E*)-1-(4-Bromo-2-chloro-6-hydroxyphenyl)-3-(4-methoxyphenyl)prop-2-en-1-one (12f)

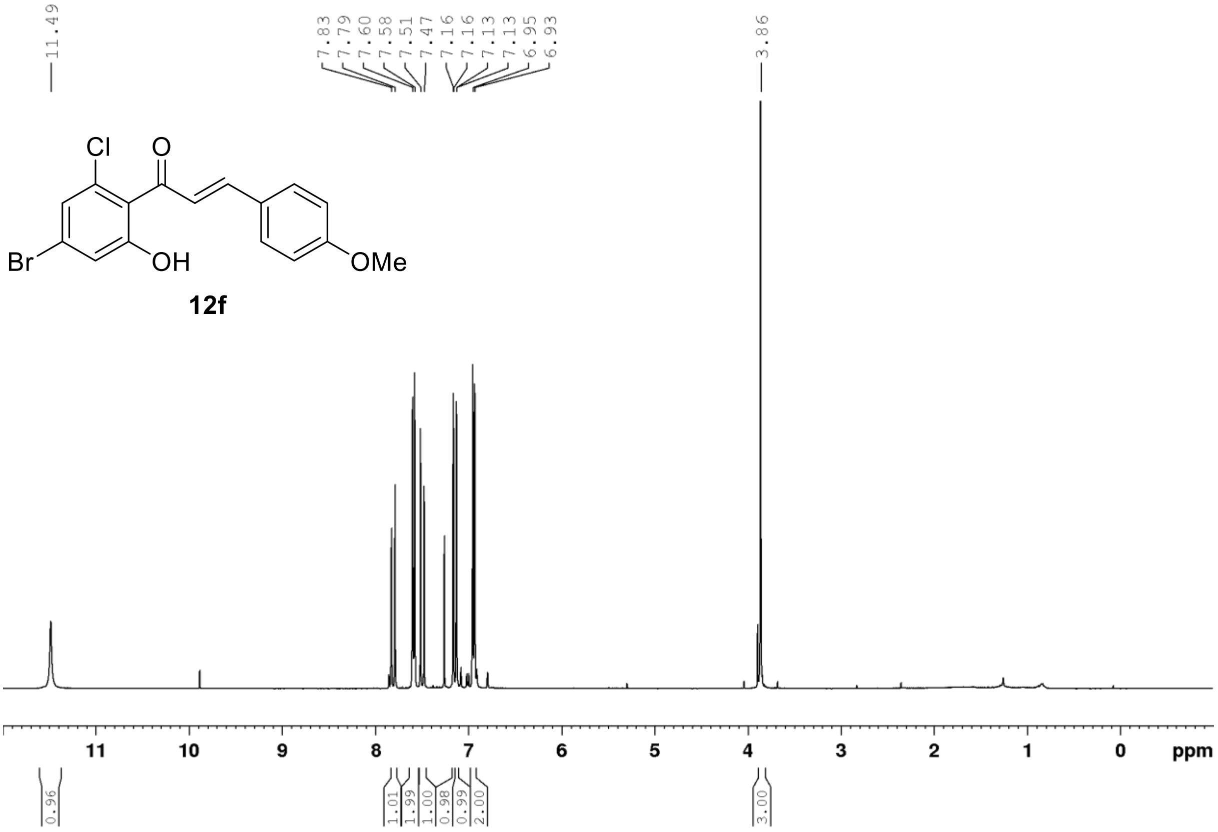

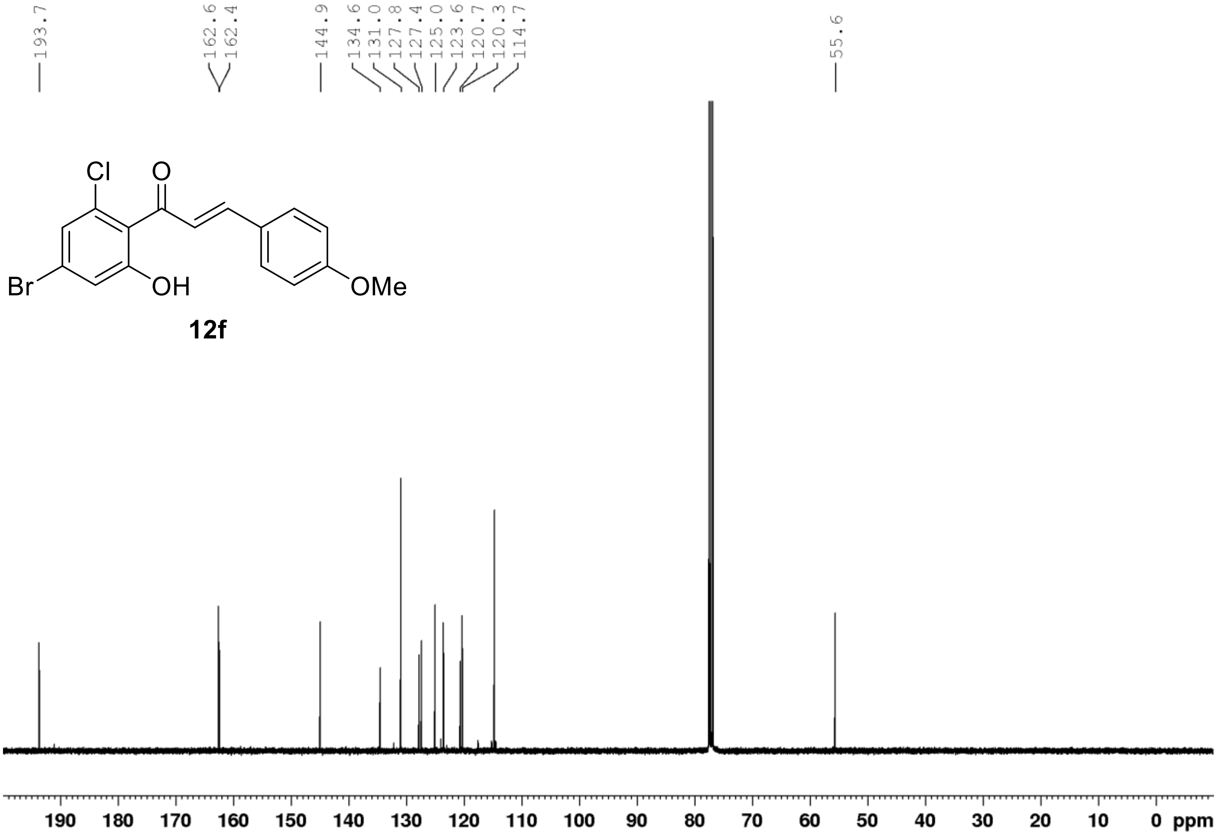

7-Bromo-5-chloro-3-hydroxy-2-(4-methoxyphenyl)-4*H*-chromen-4-one (8f)

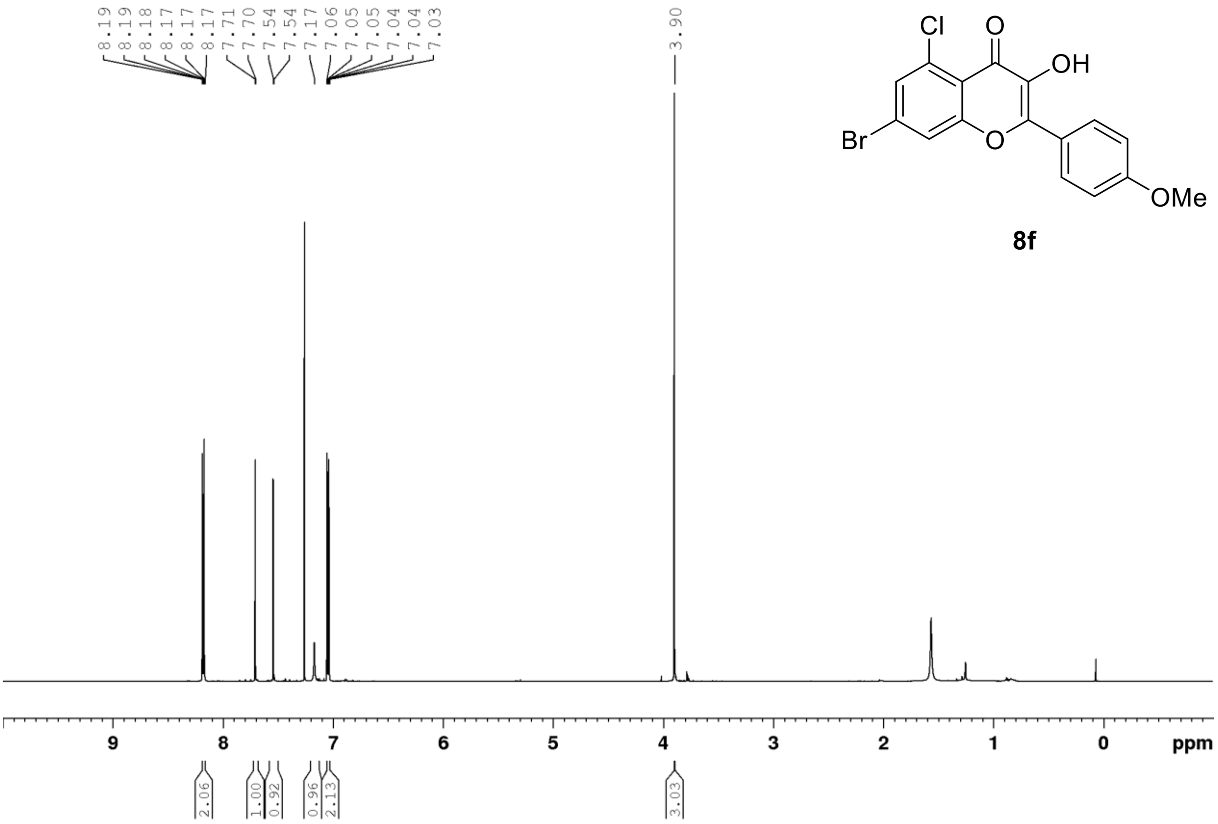

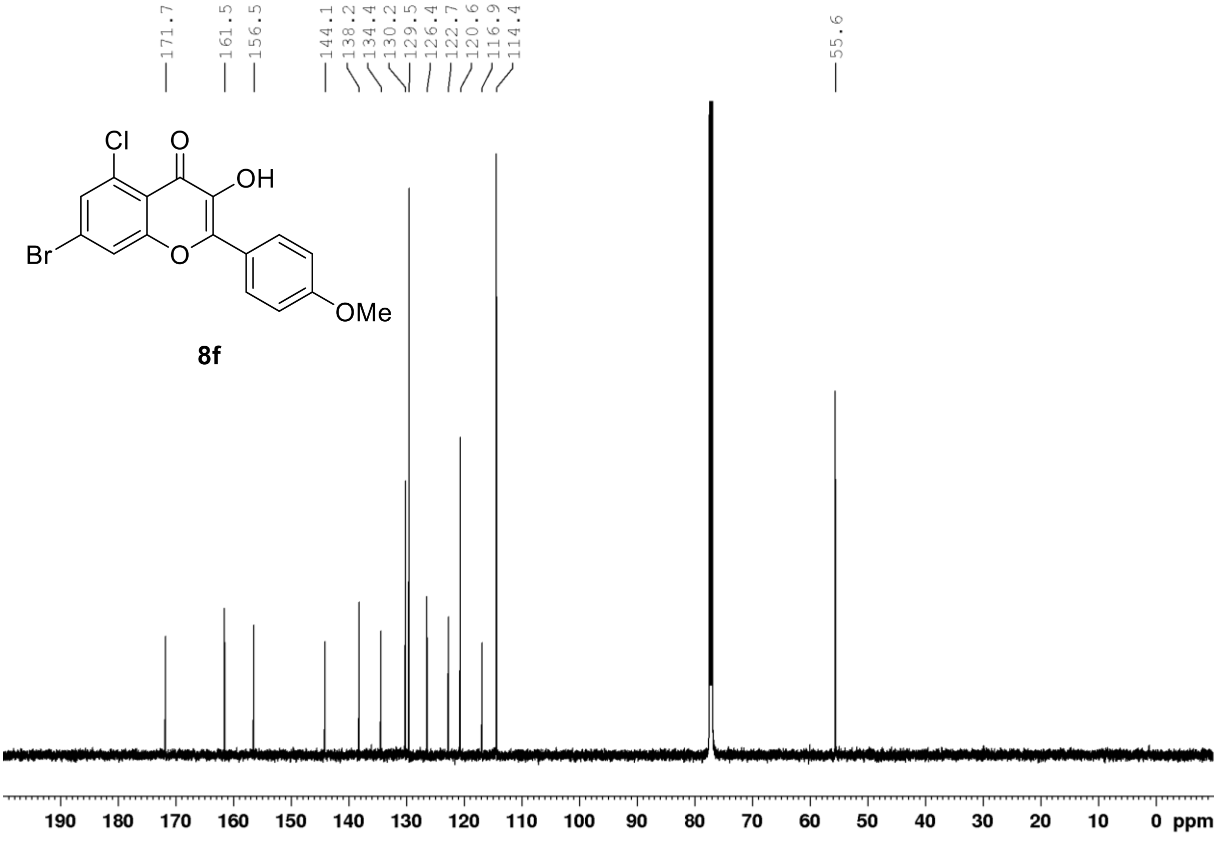

(±)-Methyl (1*R*,2*R*,3*S*,3a*R*,8b*S*)-6-bromo-8-chloro-1,8b-dihydroxy-3a-(4-methoxyphenyl)-3-phenyl-2,3,3a,8b-tetrahydro-1*H*-cyclopenta[*b*]benzofuran-2-carboxylate (9f)

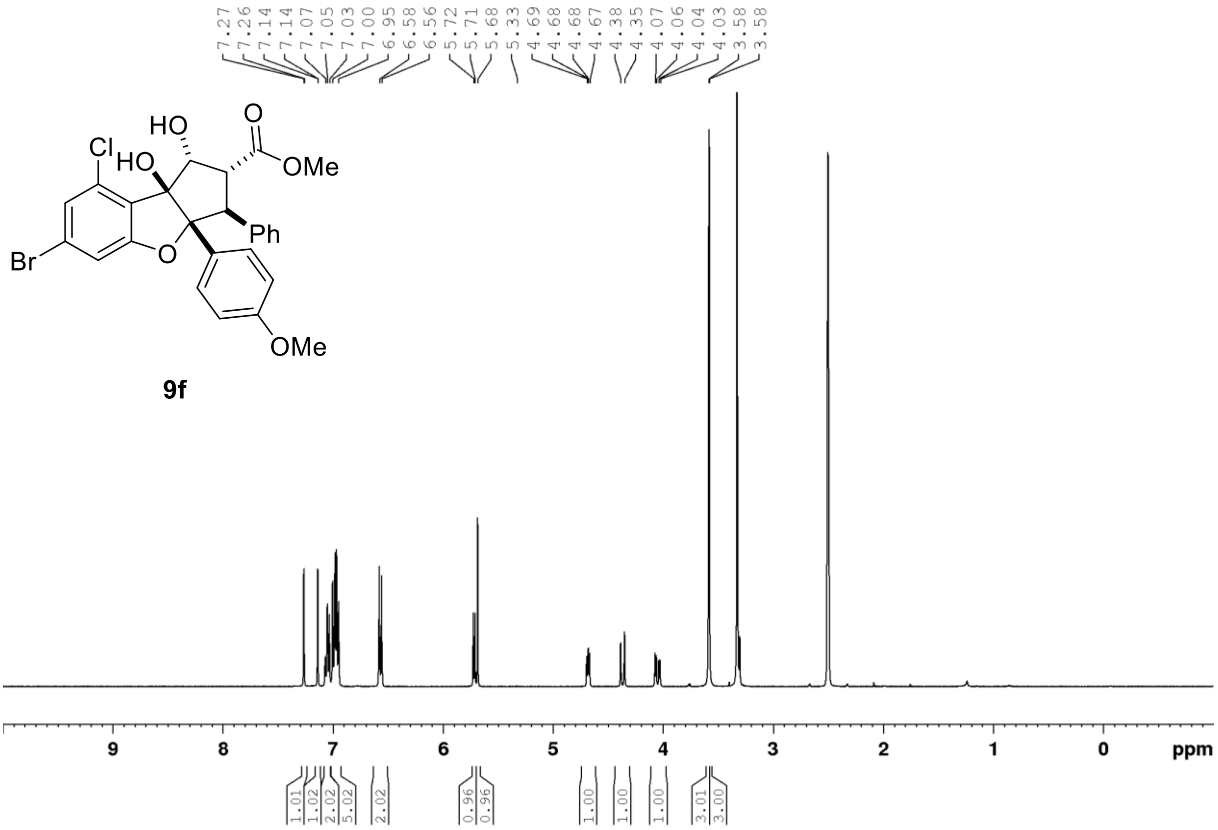

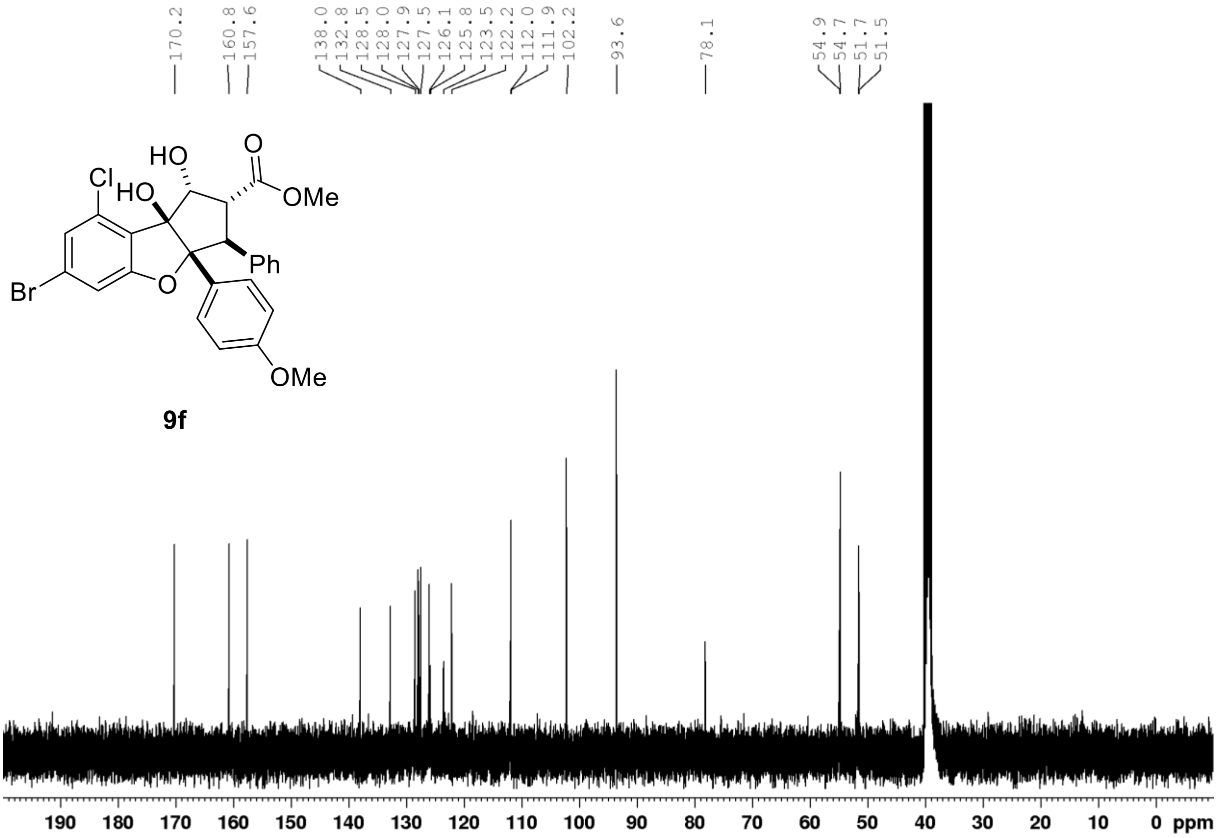

4.11. NMR spectroscopic data for the synthesis of 9g

(*E*)-1-(2-Bromo-4-chloro-6-hydroxyphenyl)-3-(4-methoxyphenyl)prop-2-en-1-one (12g)

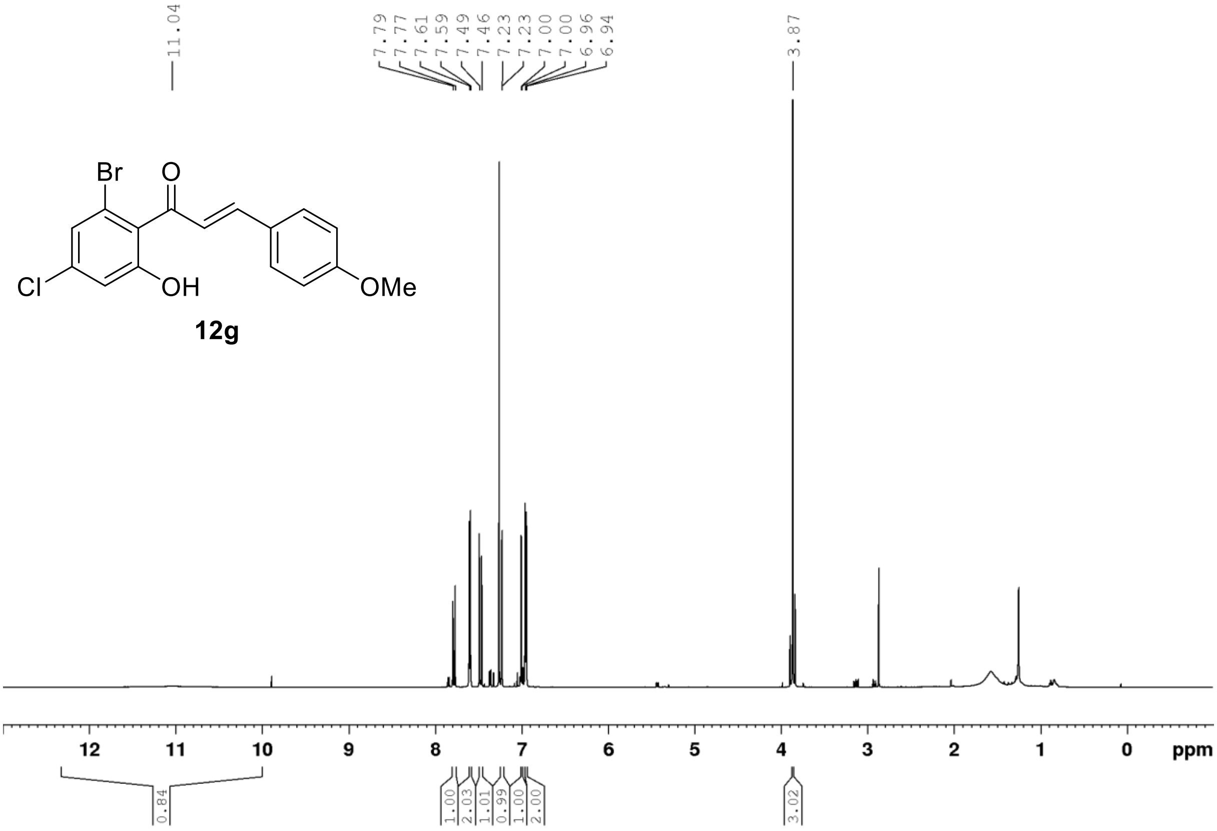

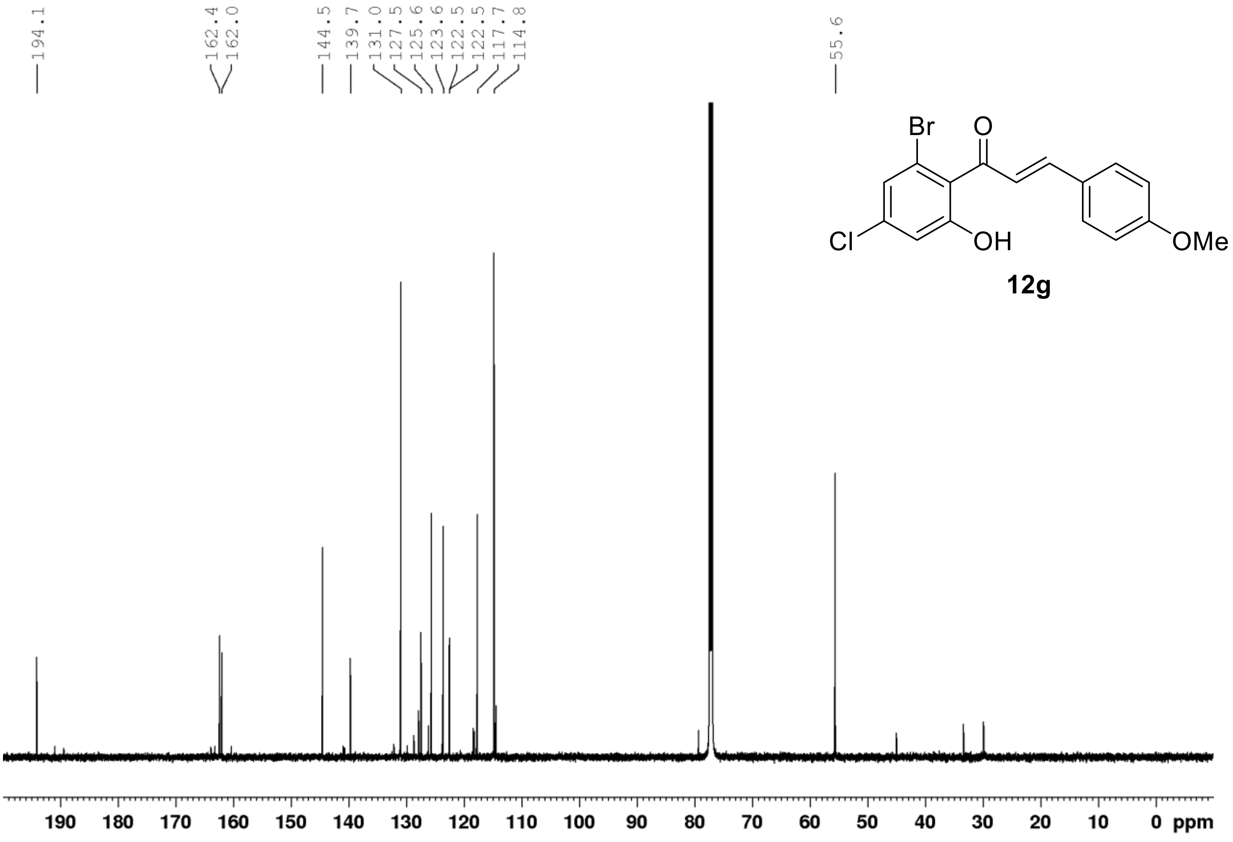

5-Bromo-7-chloro-3-hydroxy-2-(4-methoxyphenyl)-4*H*-chromen-4-one (8g)

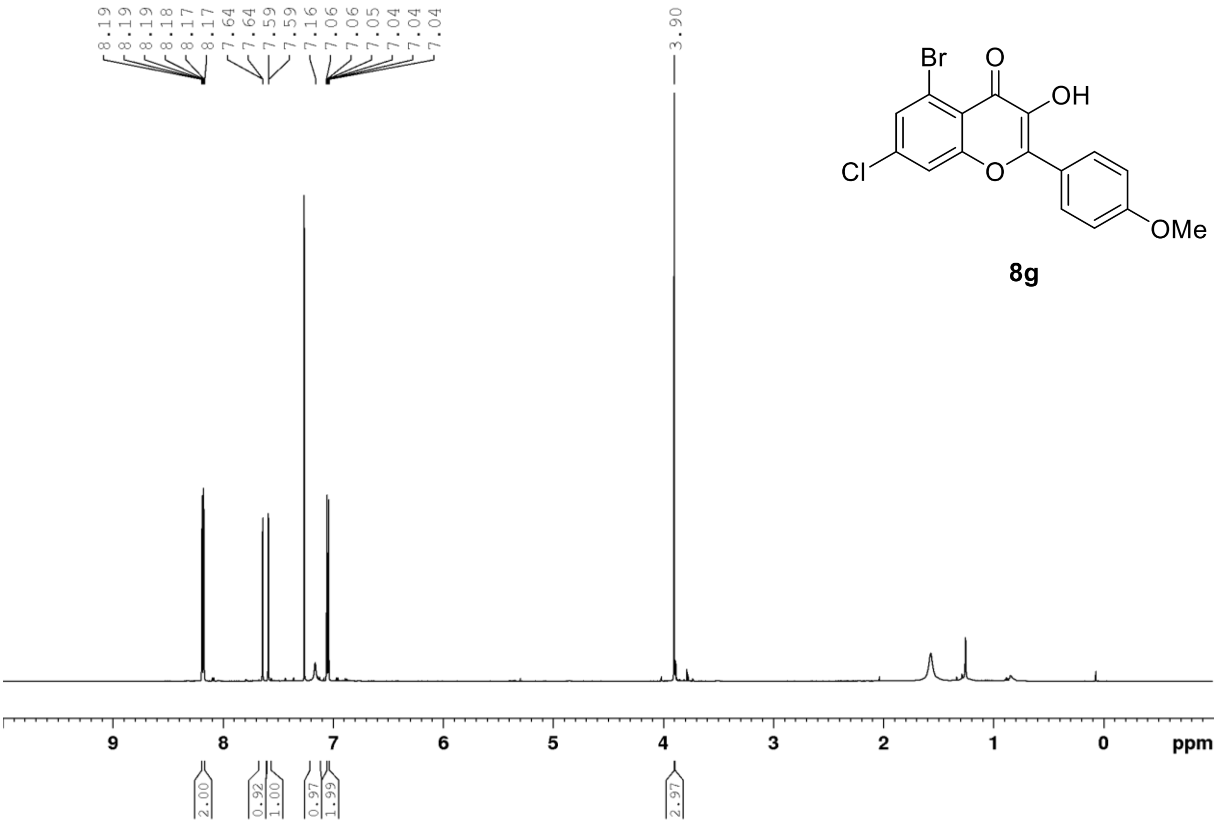

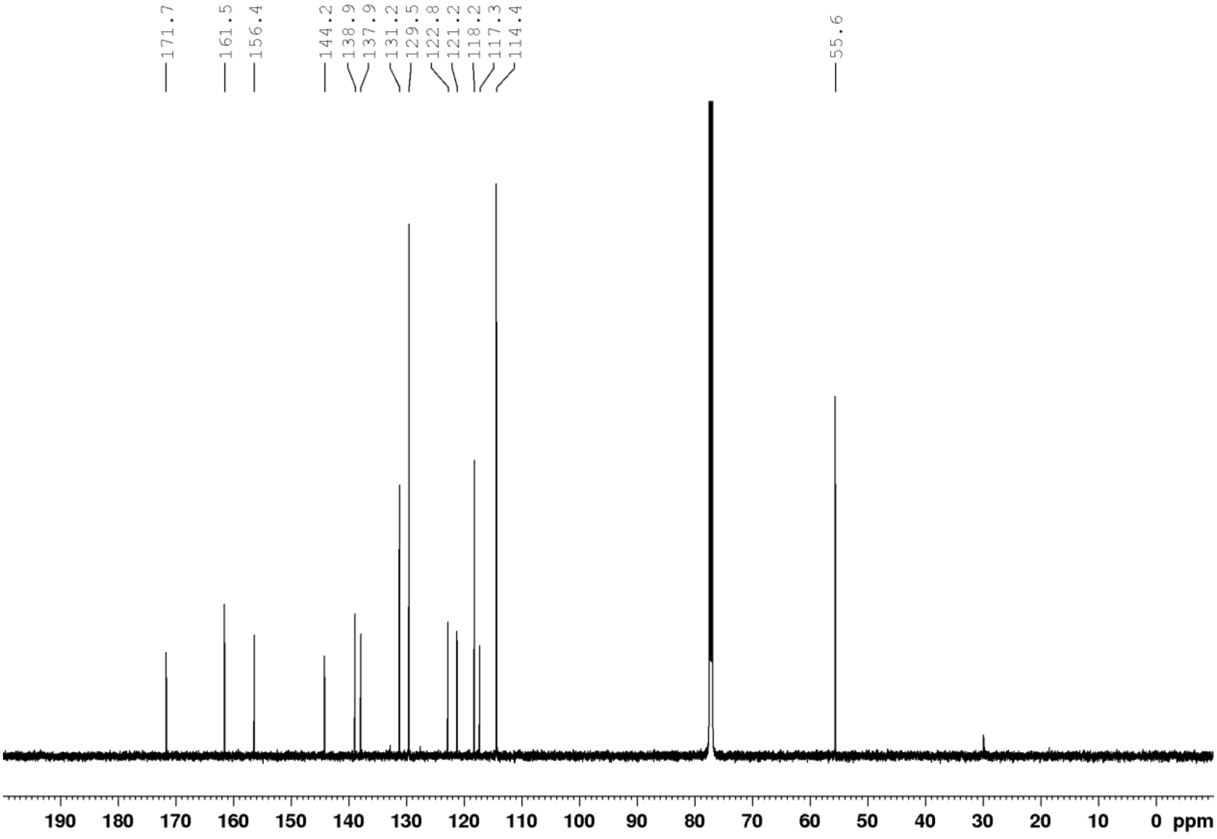

(±)-Methyl (1*R*,2*R*,3*S*,3a*R*,8b*S*)-8-bromo-6-chloro-1,8b-dihydroxy-3a-(4-methoxyphenyl)-3-phenyl-2,3,3a,8b-tetrahydro-1*H*-cyclopenta[*b*]benzofuran-2-carboxylate (9g)

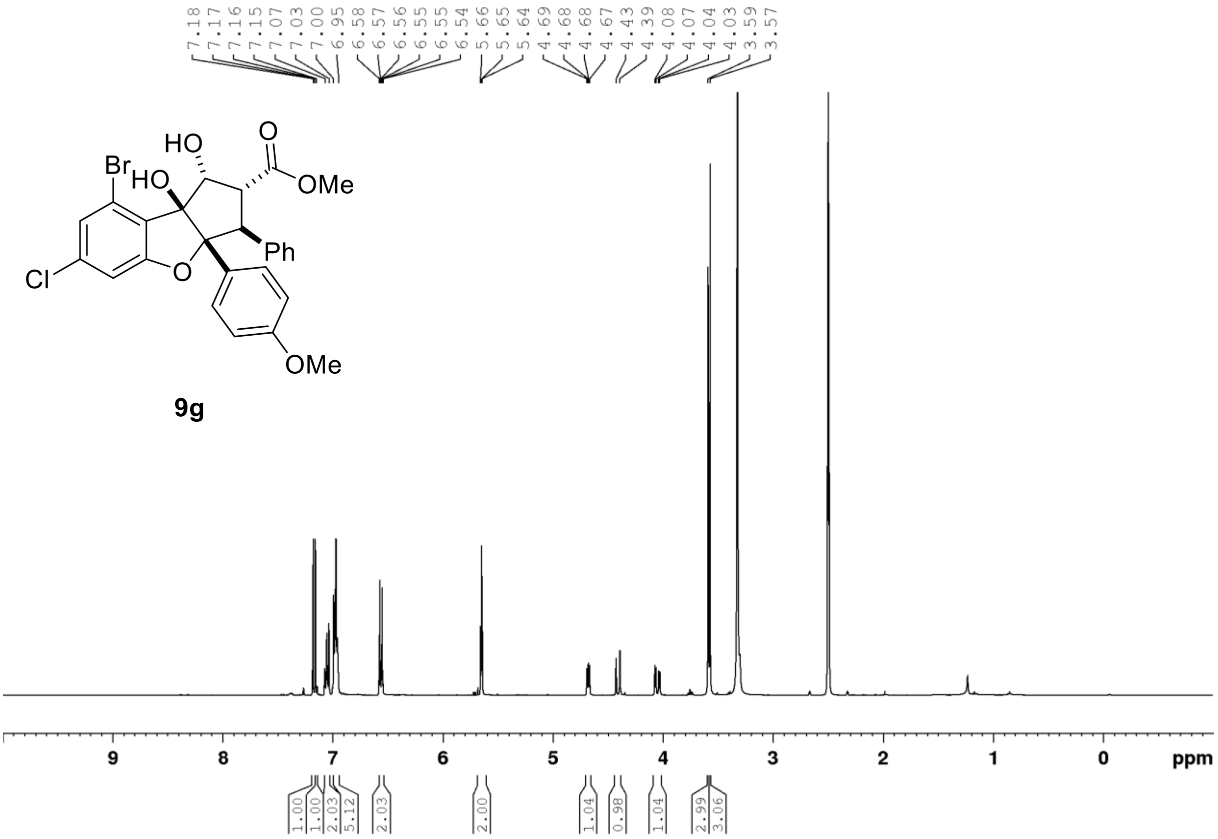

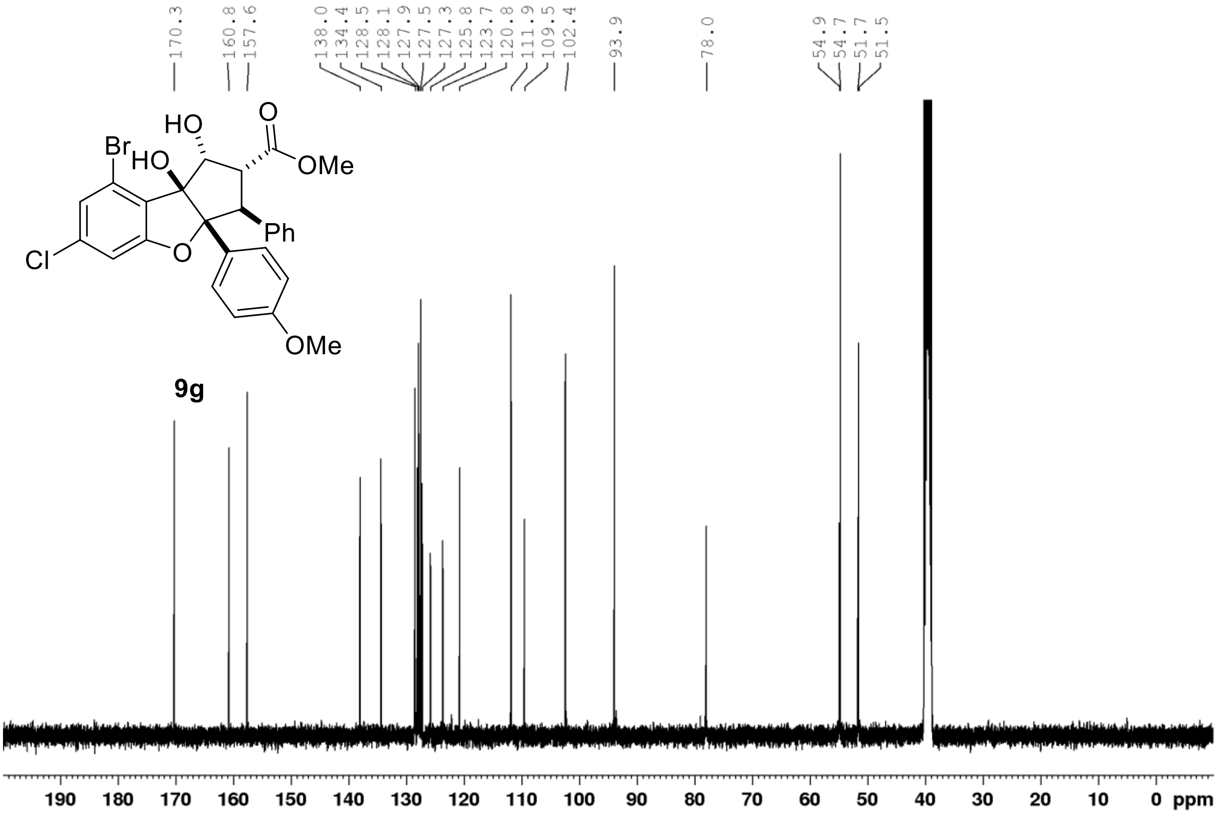

4.12. NMR spectroscopic data for the synthesis of 9h

(*E*)-1-(2-fluoro-6-hydroxy-4-methoxyphenyl)-3-(4-methoxyphenyl)prop-2-en-1-one (12h)

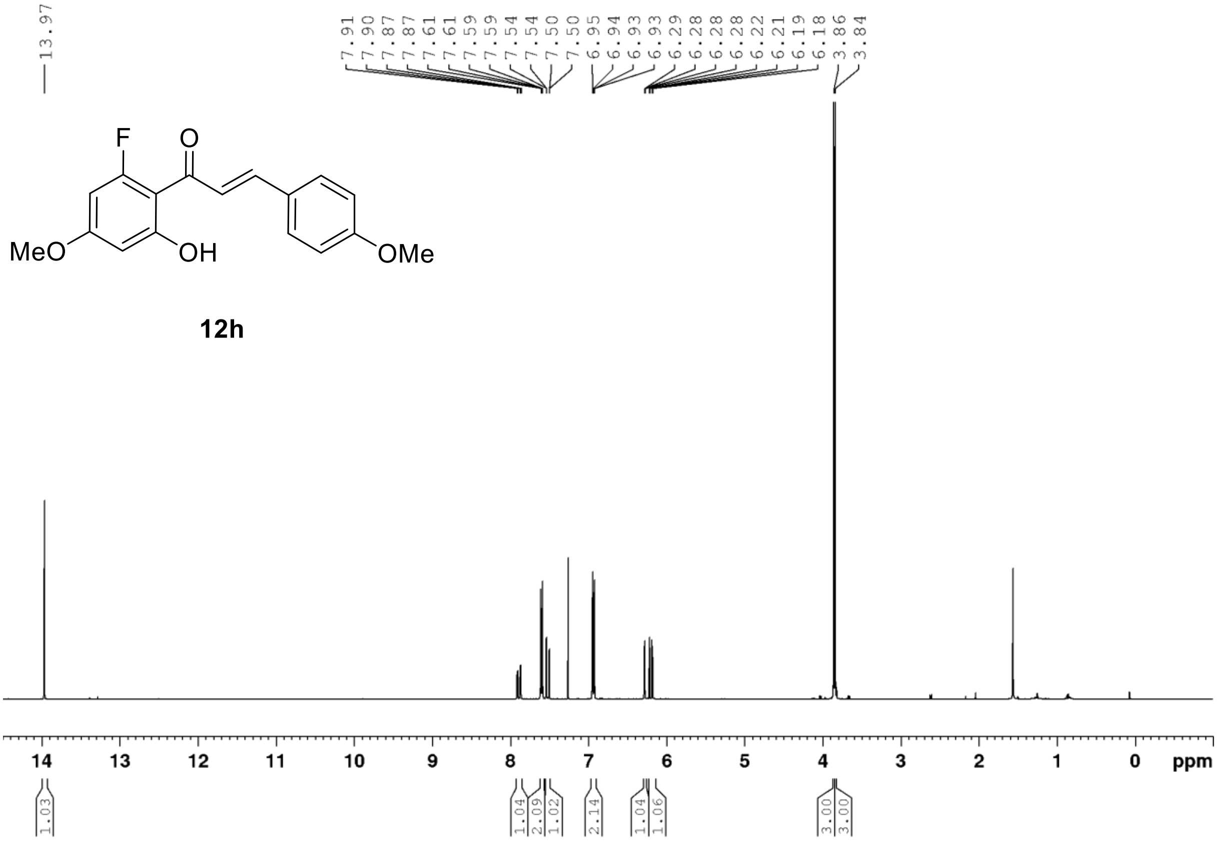

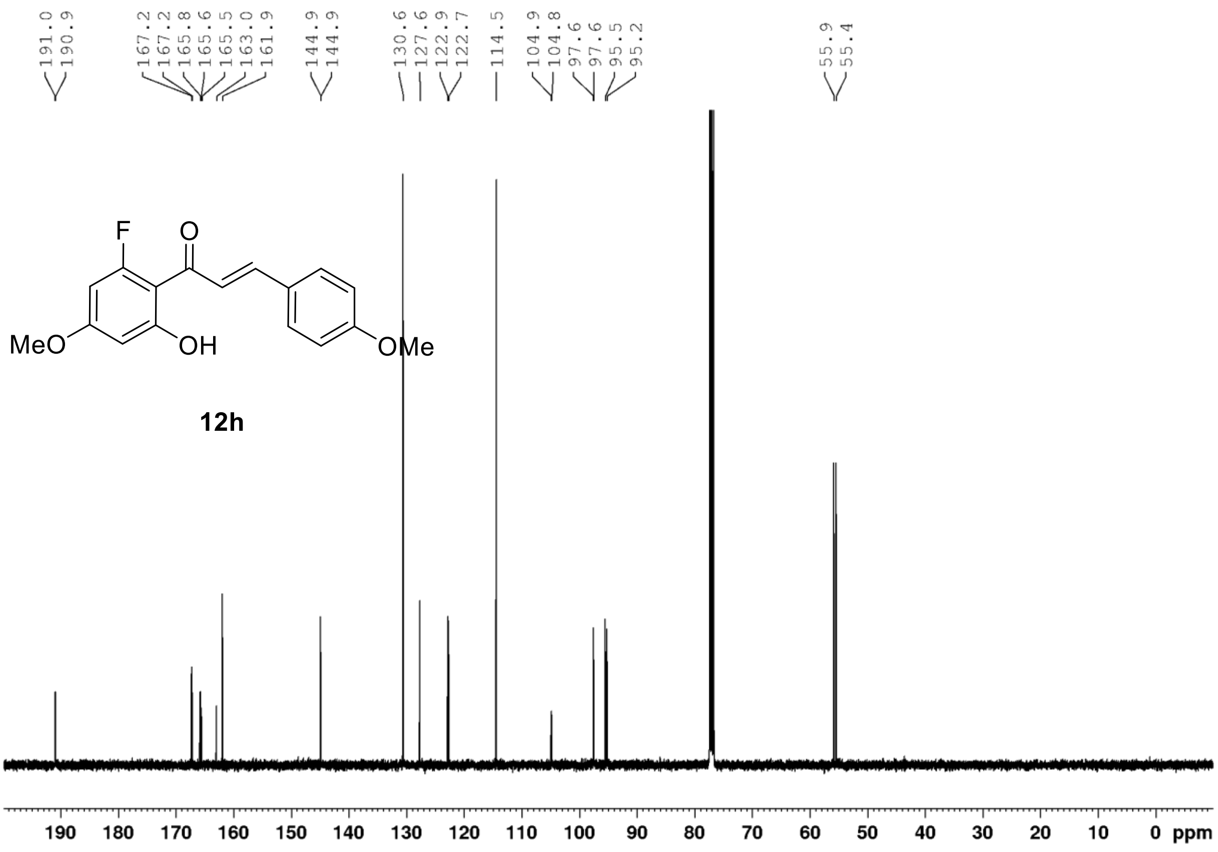

5-Fluoro-3-hydroxy-7-methoxy-2-(4-methoxyphenyl)-4*H*-chromen-4-one (8h)

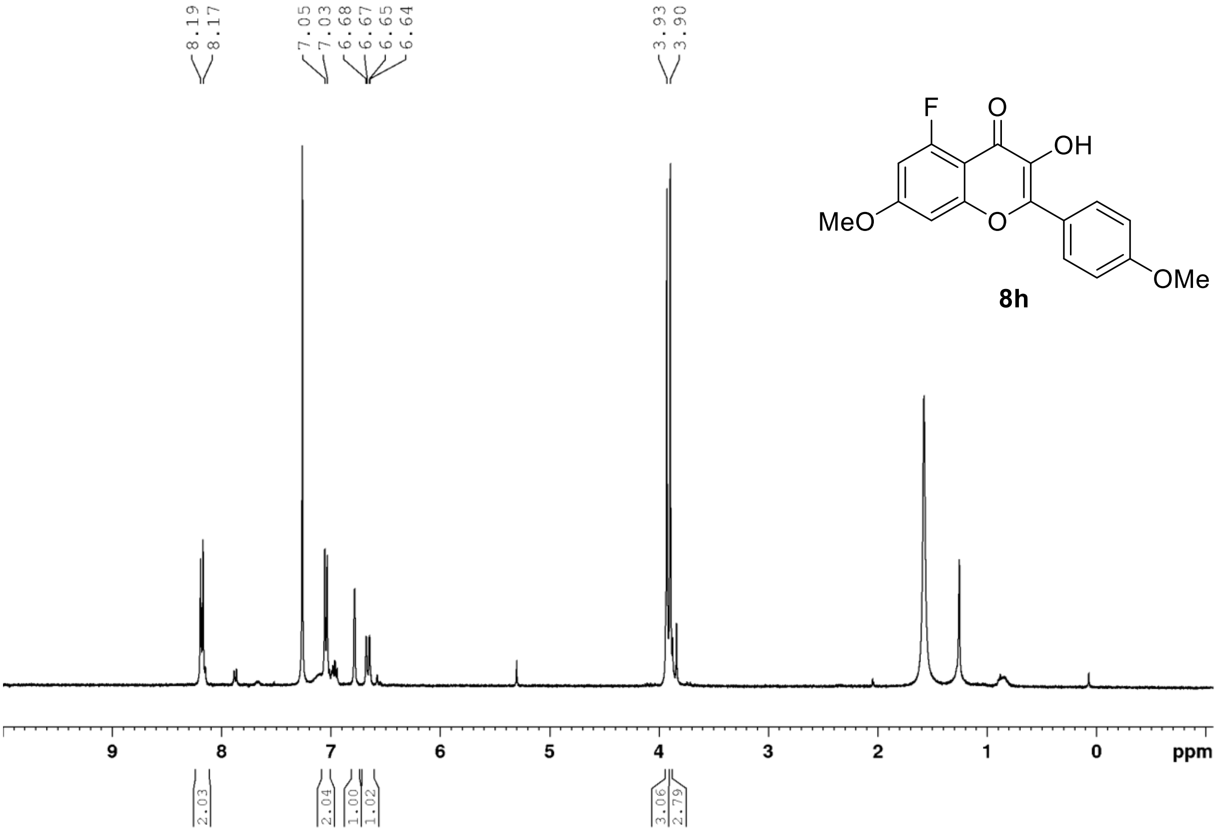

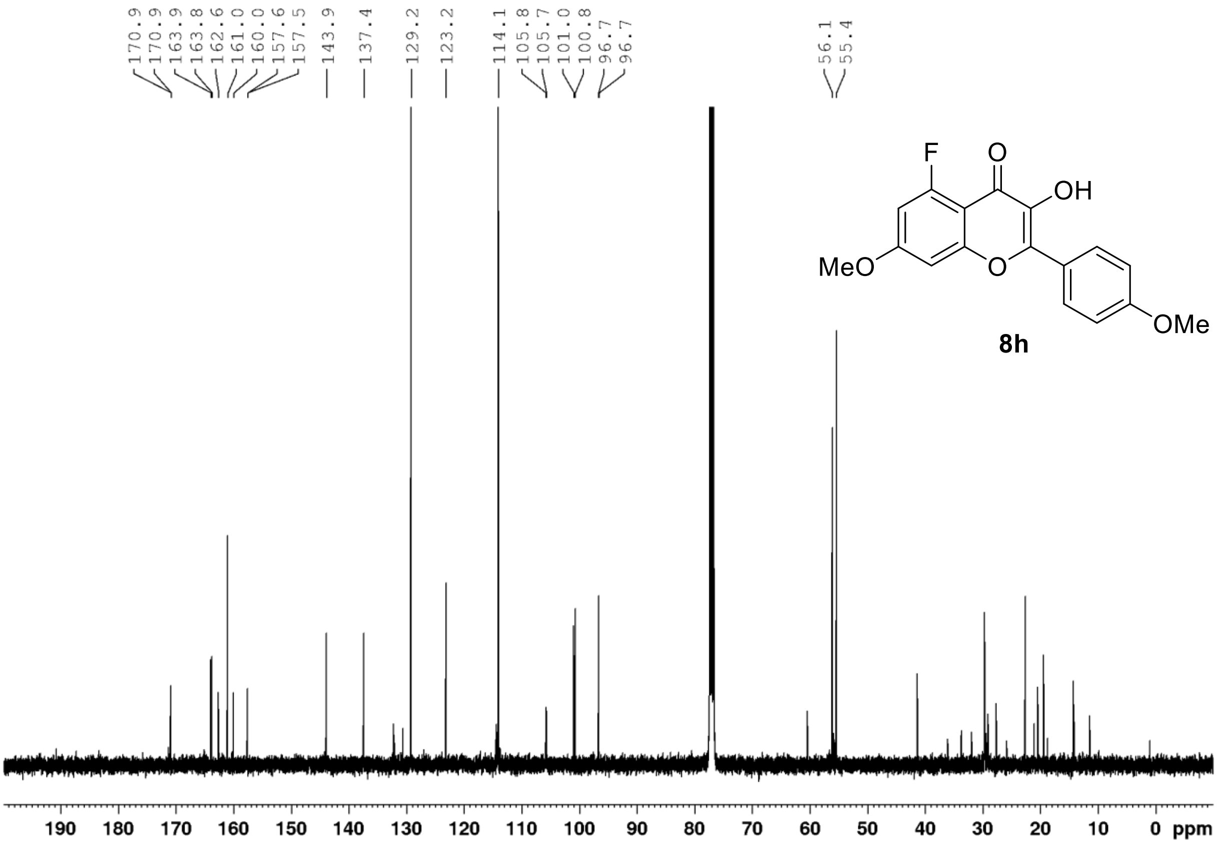

(±)-Methyl (1*R*,2*R*,3*S*,3a*R*,8b*S*)-8-fluoro-1,8b-dihydroxy-6-methoxy-3a-(4-methoxyphenyl)-3-phenyl-2,3,3a,8b-tetrahydro-1*H*-cyclopenta[*b*]benzofuran-2-carboxylate (9h)

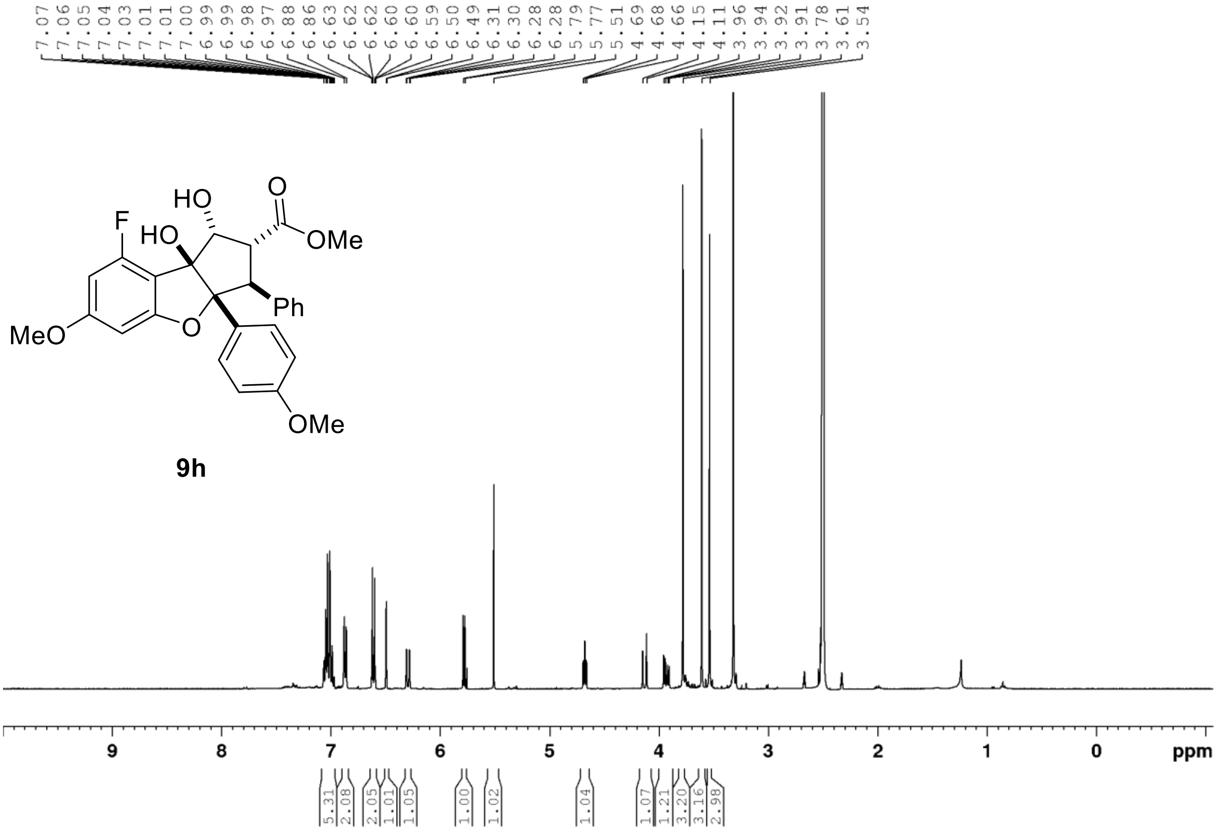

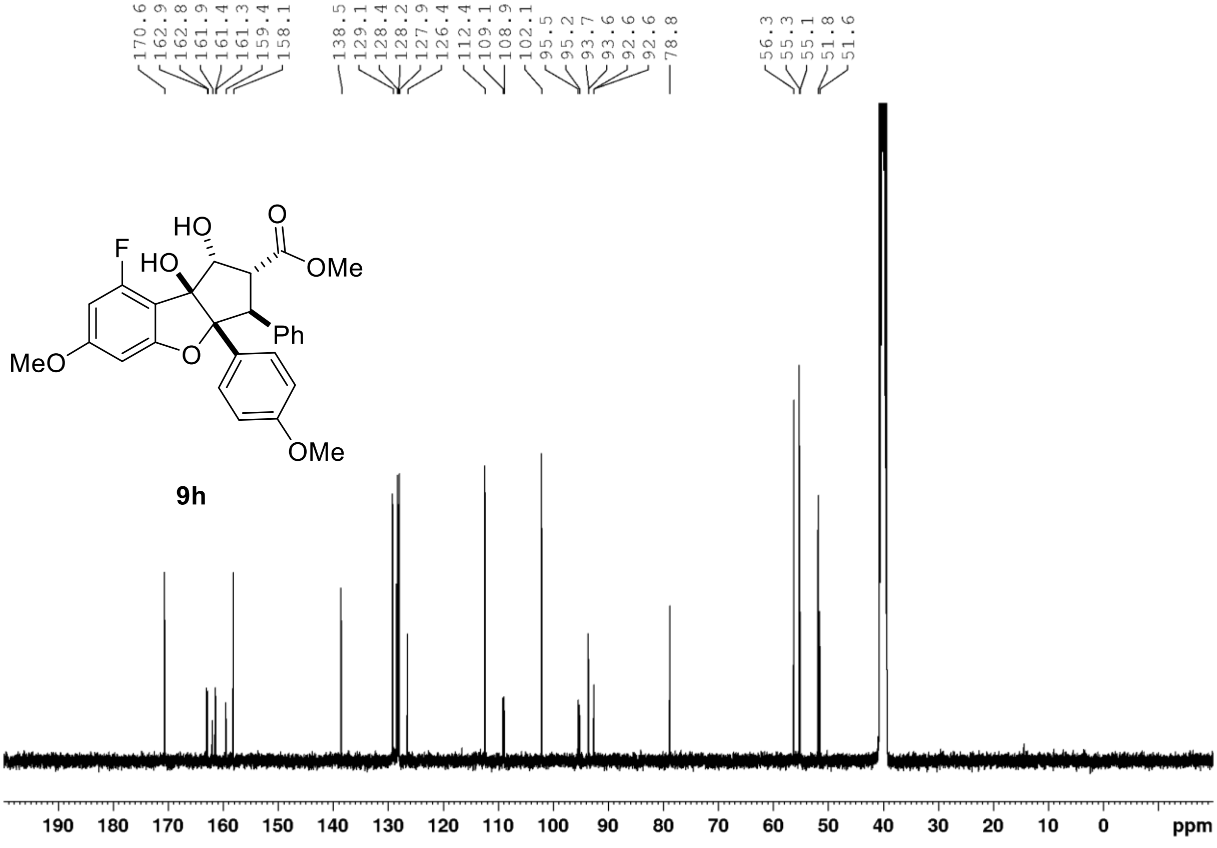

4.13. NMR spectroscopic data for the synthesis of 9i

(*E*)-1-(4-Fluoro-2-hydroxy-6-methoxyphenyl)-3-(4-methoxyphenyl)prop-2-en-1-one (12i)

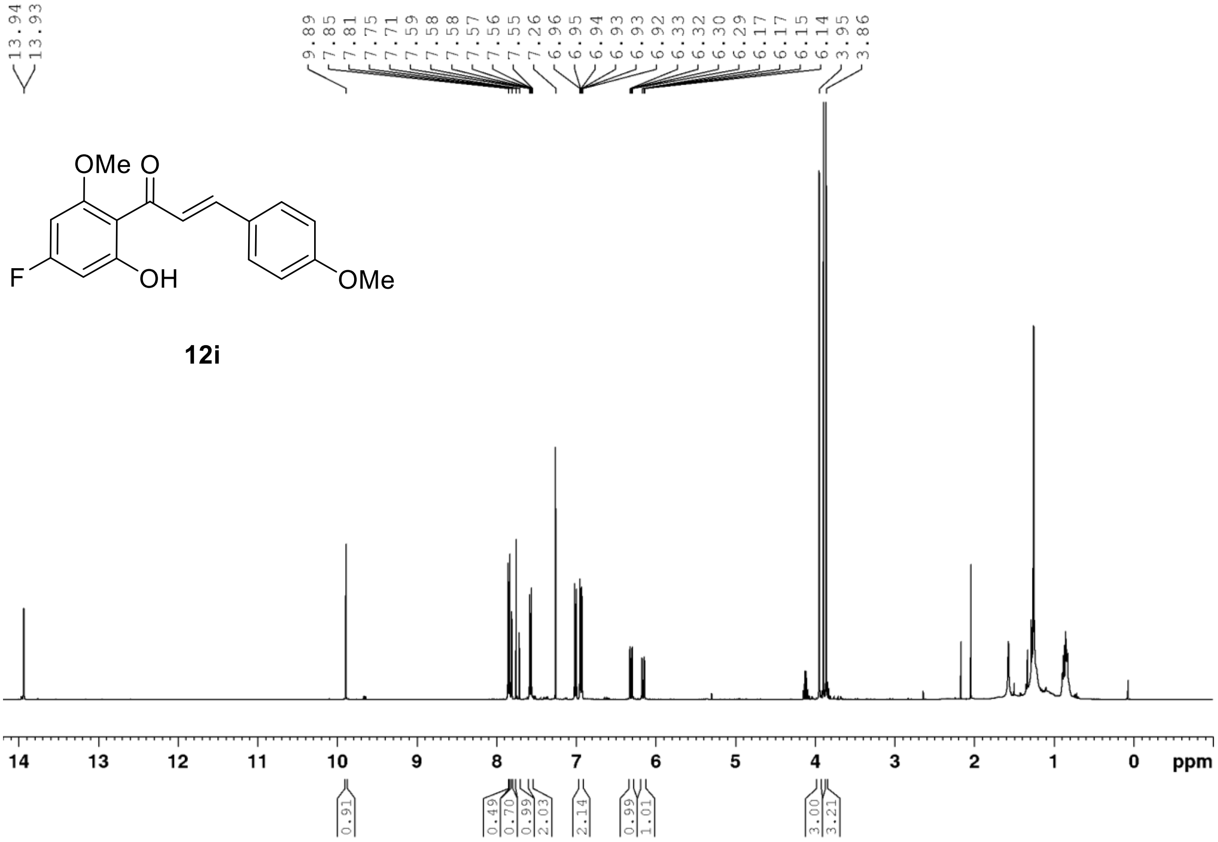

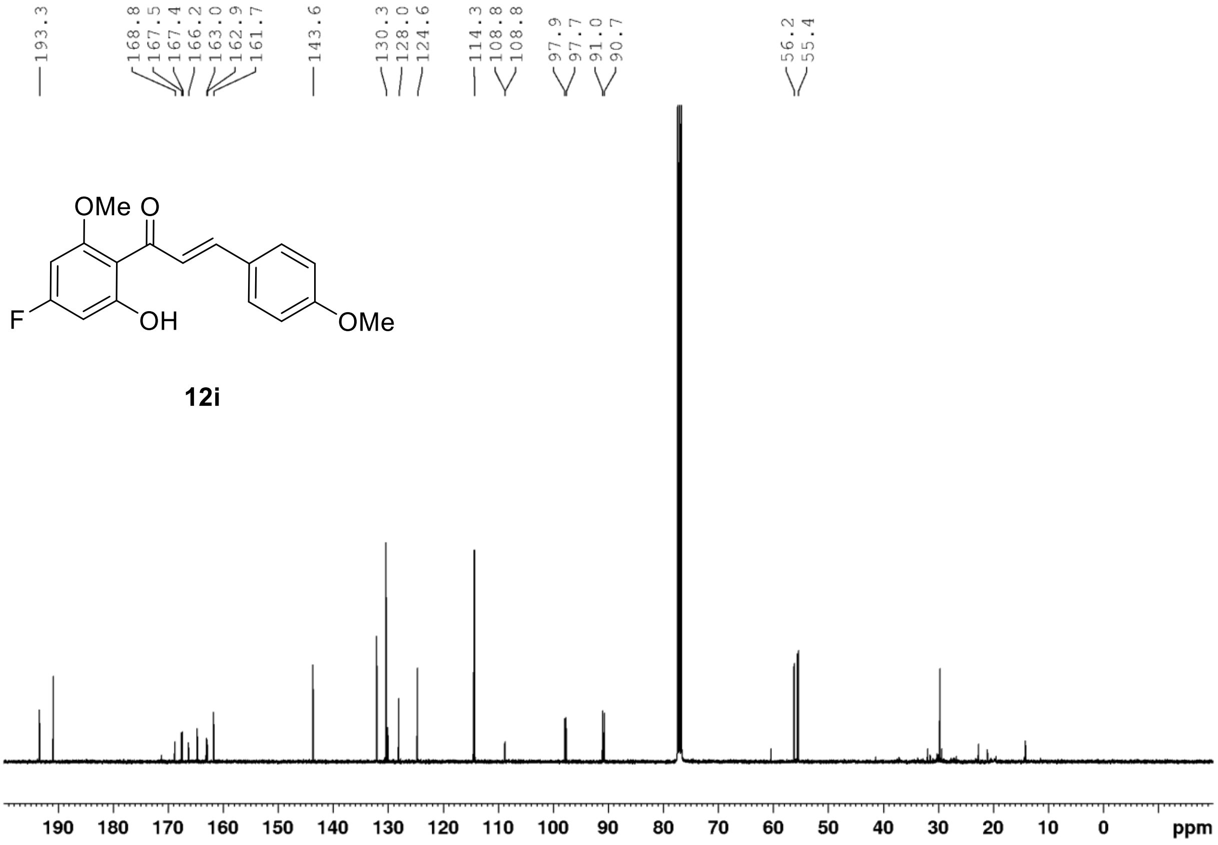

7-Fluoro-3-hydroxy-5-methoxy-2-(4-methoxyphenyl)-4*H*-chromen-4-one (8i)

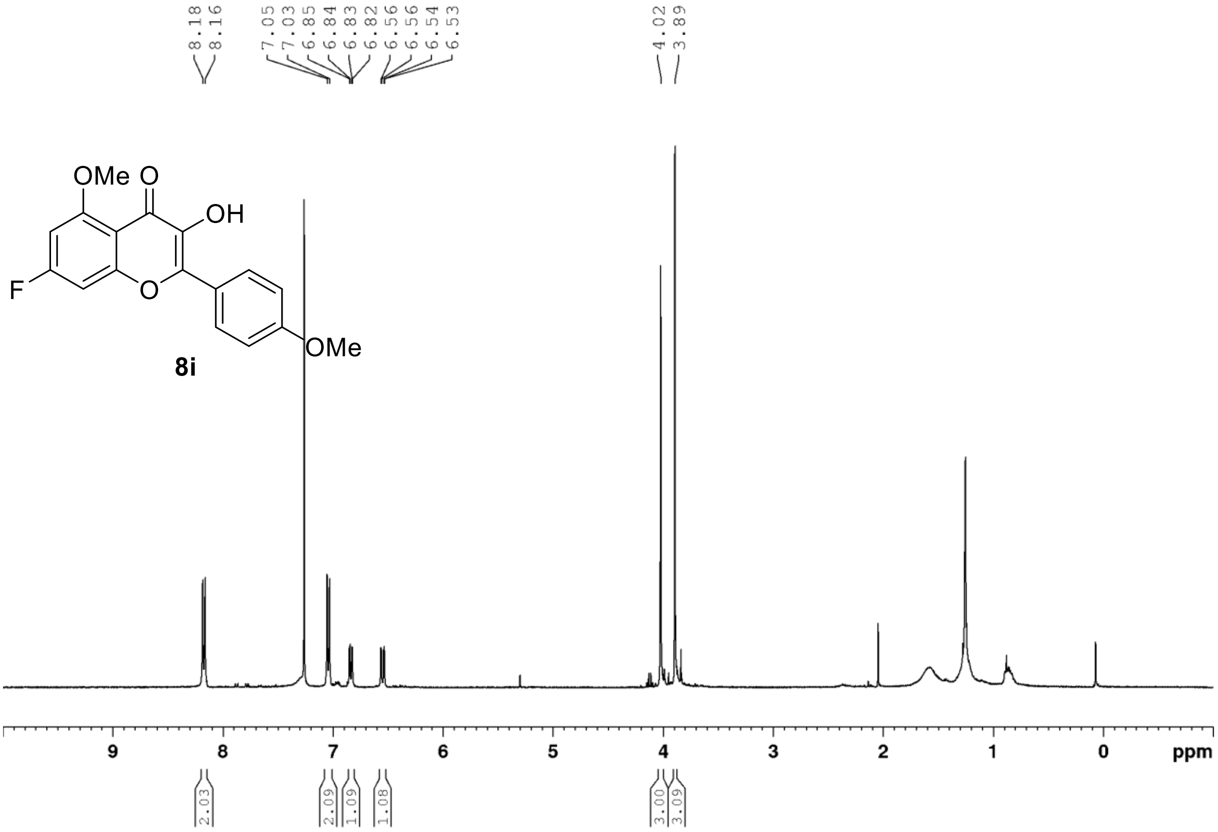

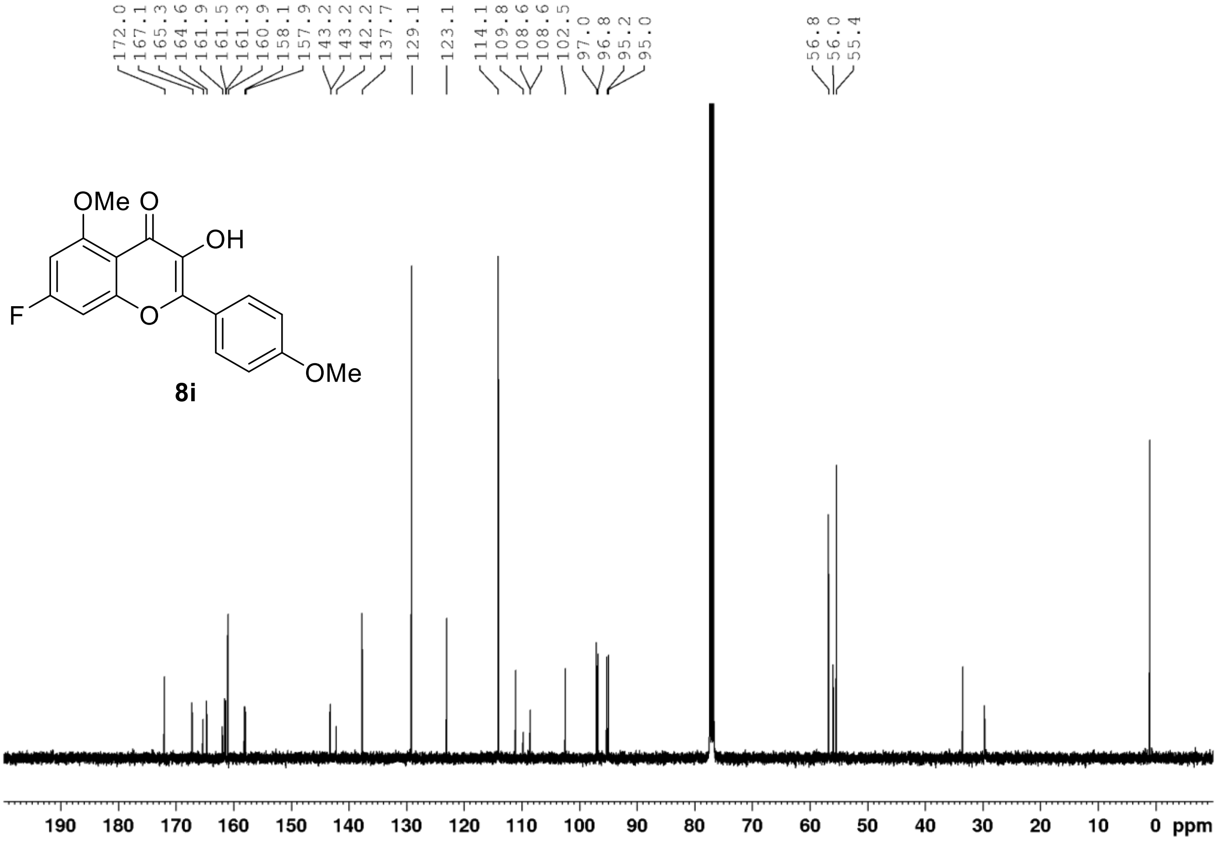

(±)-Methyl (1*R*,2*R*,3*S*,3a*R*,8b*S*)-6-fluoro-1,8b-dihydroxy-8-methoxy-3a-(4-methoxyphenyl)-3-phenyl-2,3,3a,8b-tetrahydro-1*H*-cyclopenta[*b*]benzofuran-2-carboxylate (9i)

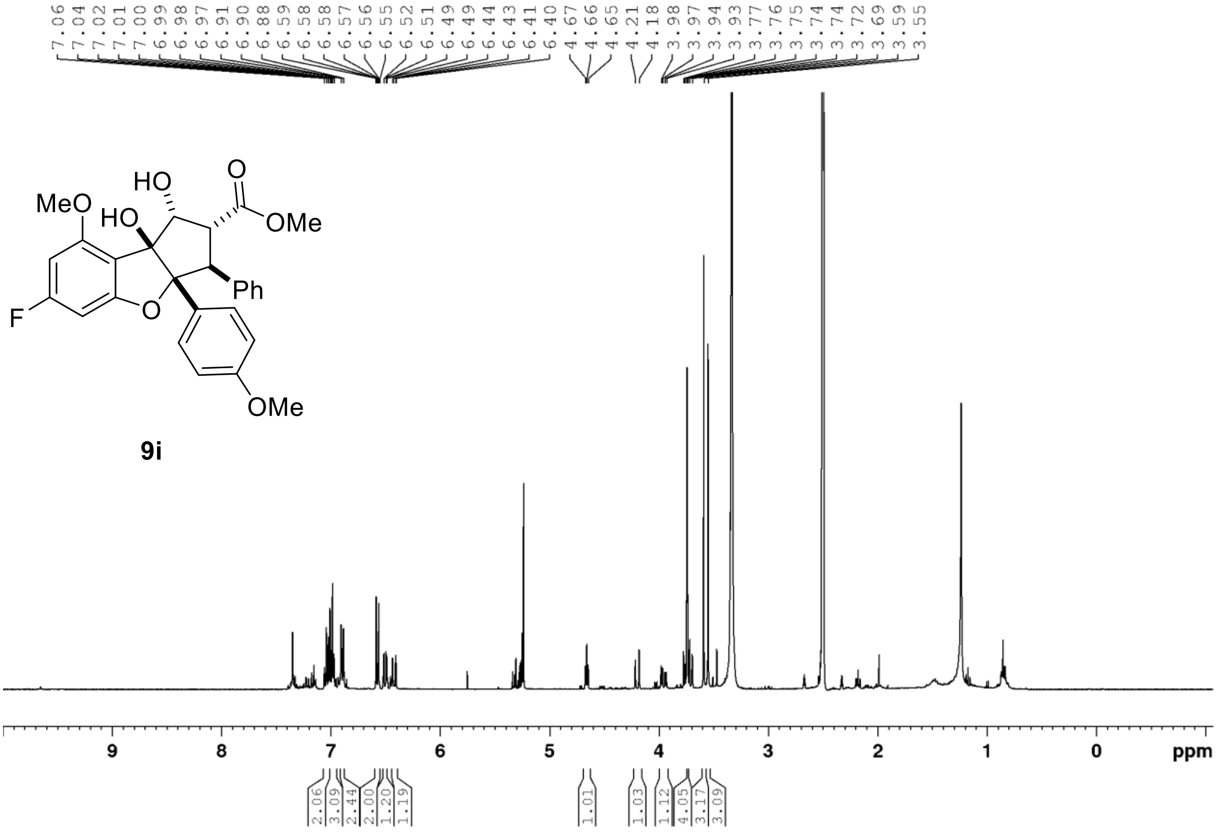

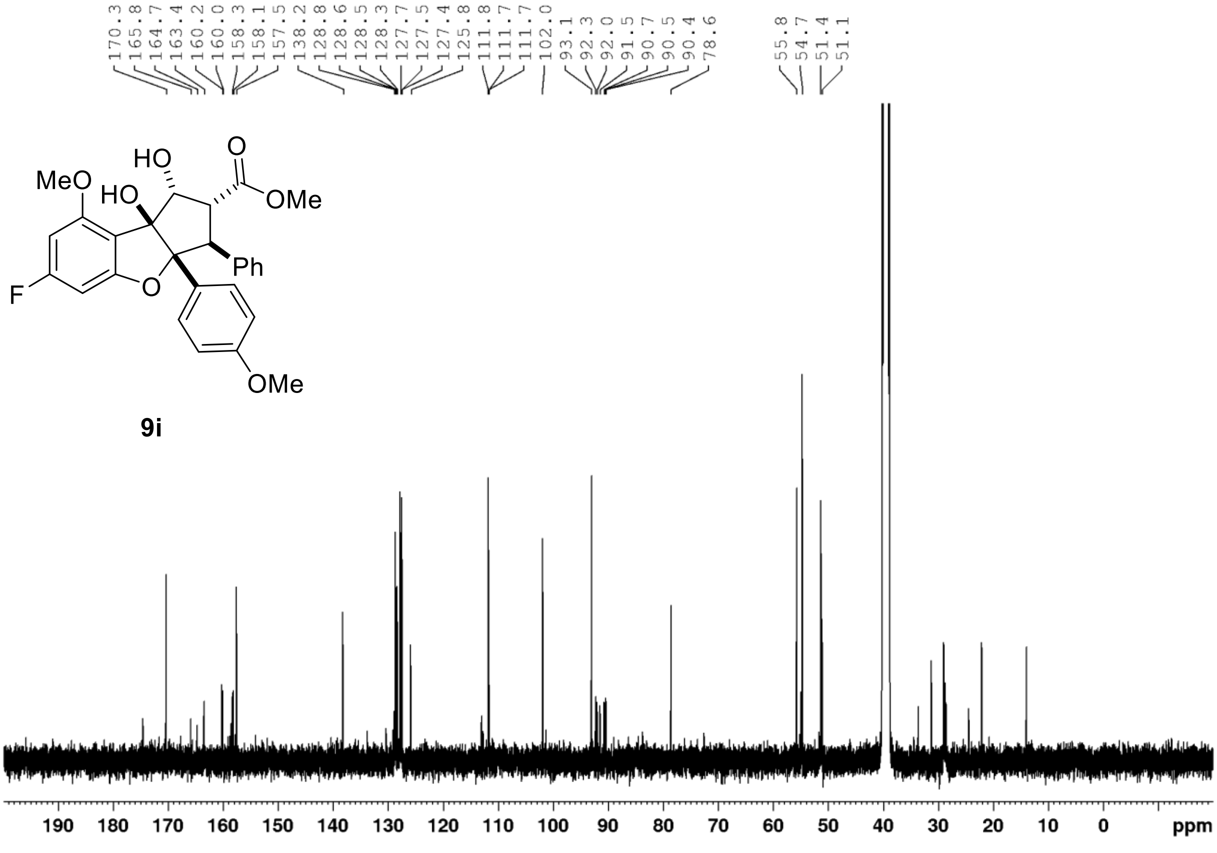

4.14. NMR spectroscopic data for the synthesis of 9j

(*E*)-1-(2-Chloro-6-hydroxy-4-methoxyphenyl)-3-(4-methoxyphenyl)prop-2-en-1-one (12j)

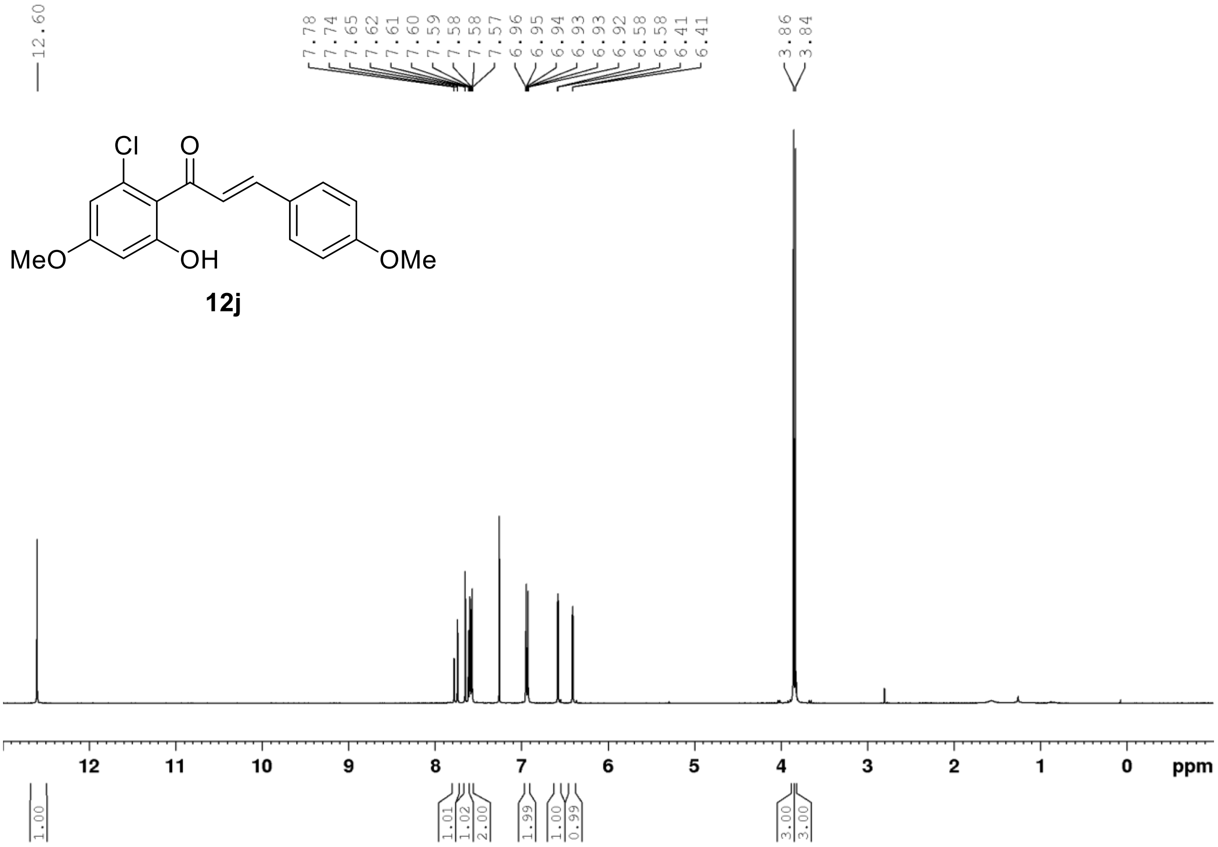

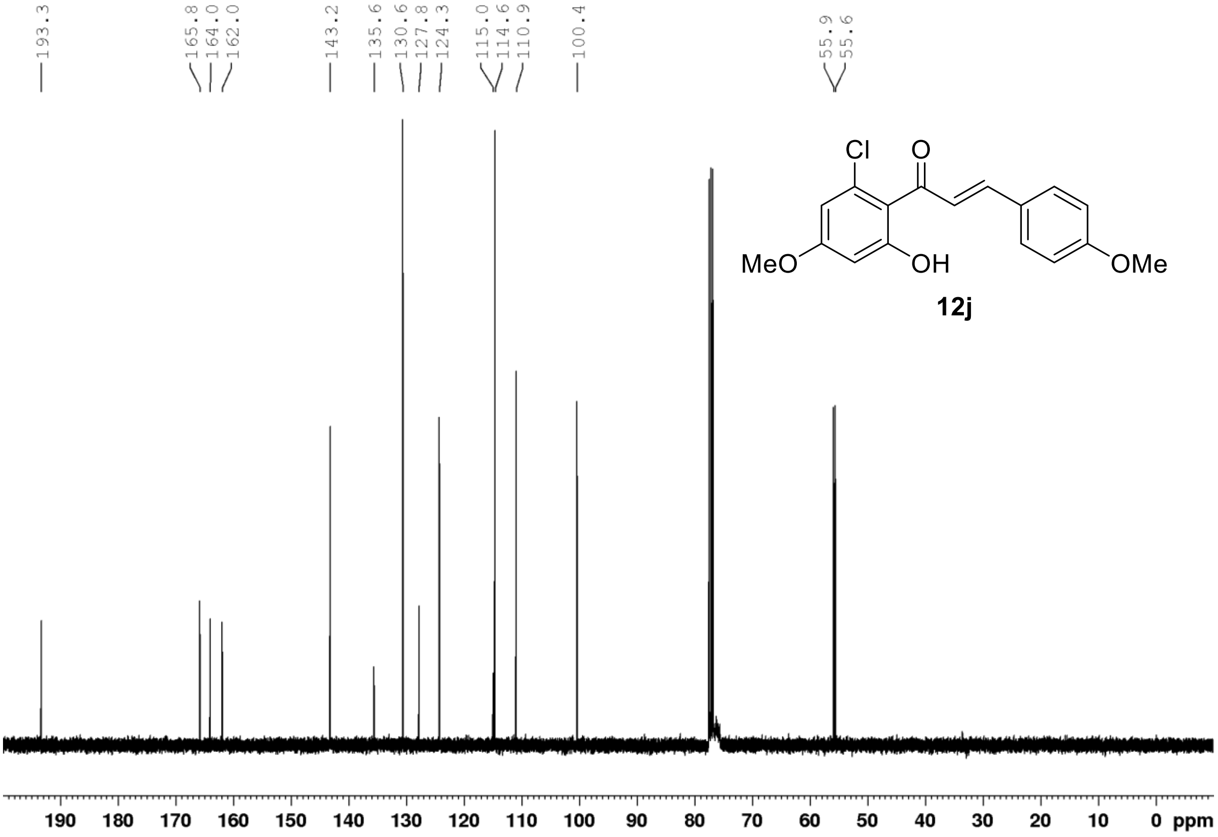

5-Chloro-3-hydroxy-7-methoxy-2-(4-methoxyphenyl)-4*H*-chromen-4-one (8j)

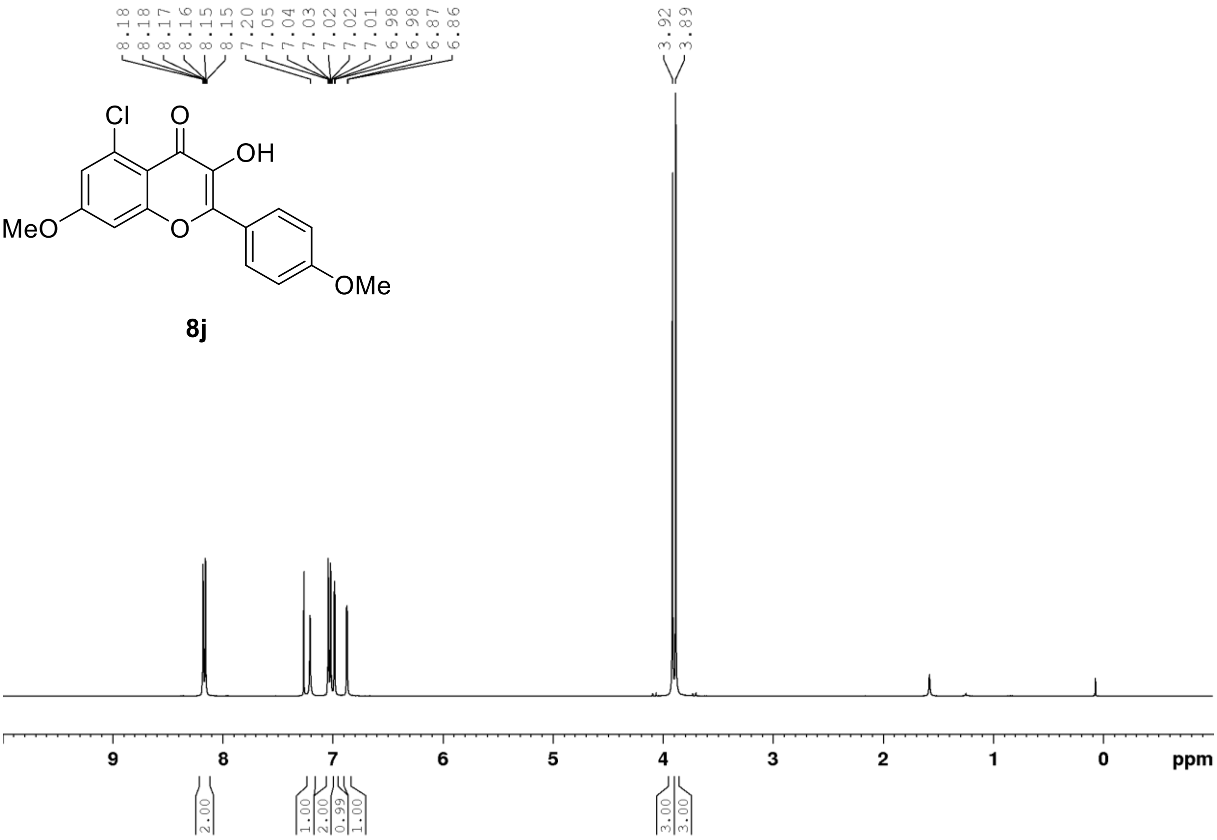

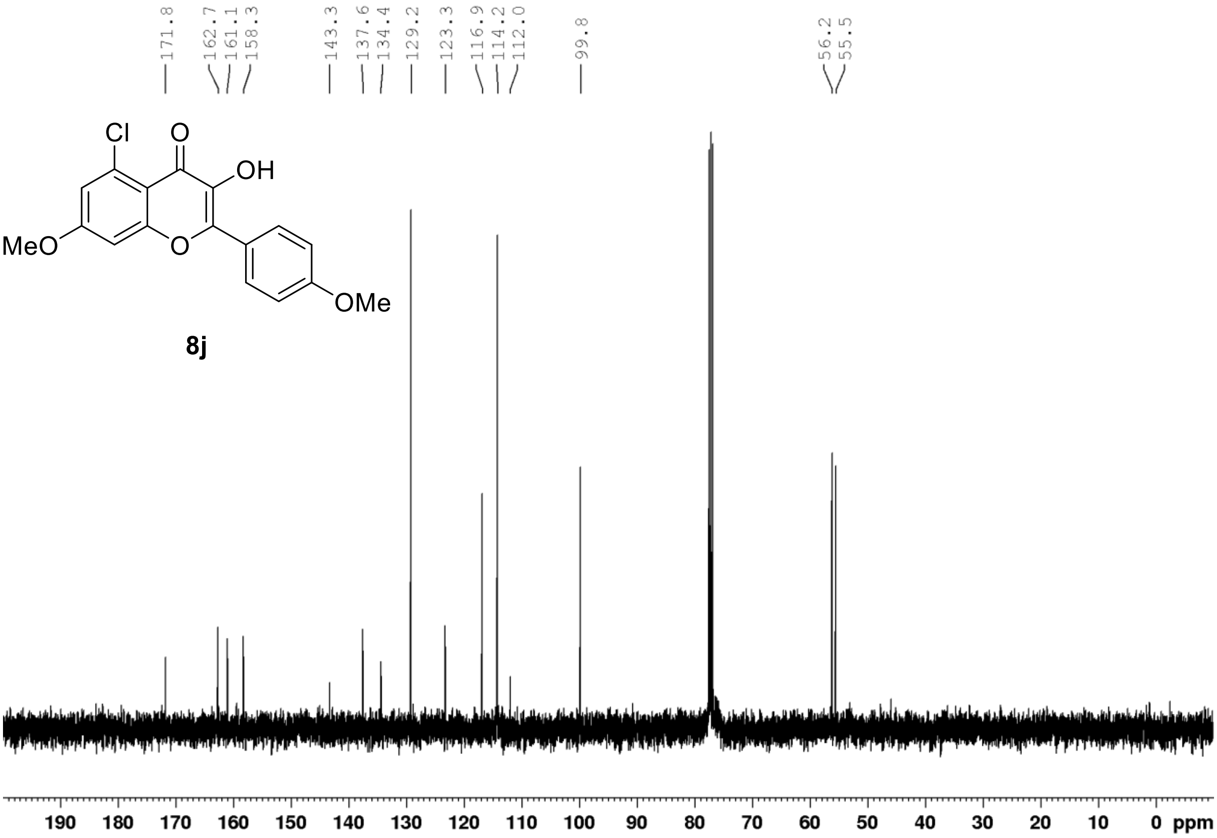

(±)-Methyl (1*R*,2*R*,3*S*,3a*R*,8b*S*)-8-chloro-1,8b-dihydroxy-6-methoxy-3a-(4-methoxyphenyl)-3-phenyl-2,3,3a,8b-tetrahydro-1*H*-cyclopenta[*b*]benzofuran-2-carboxylate (9j)

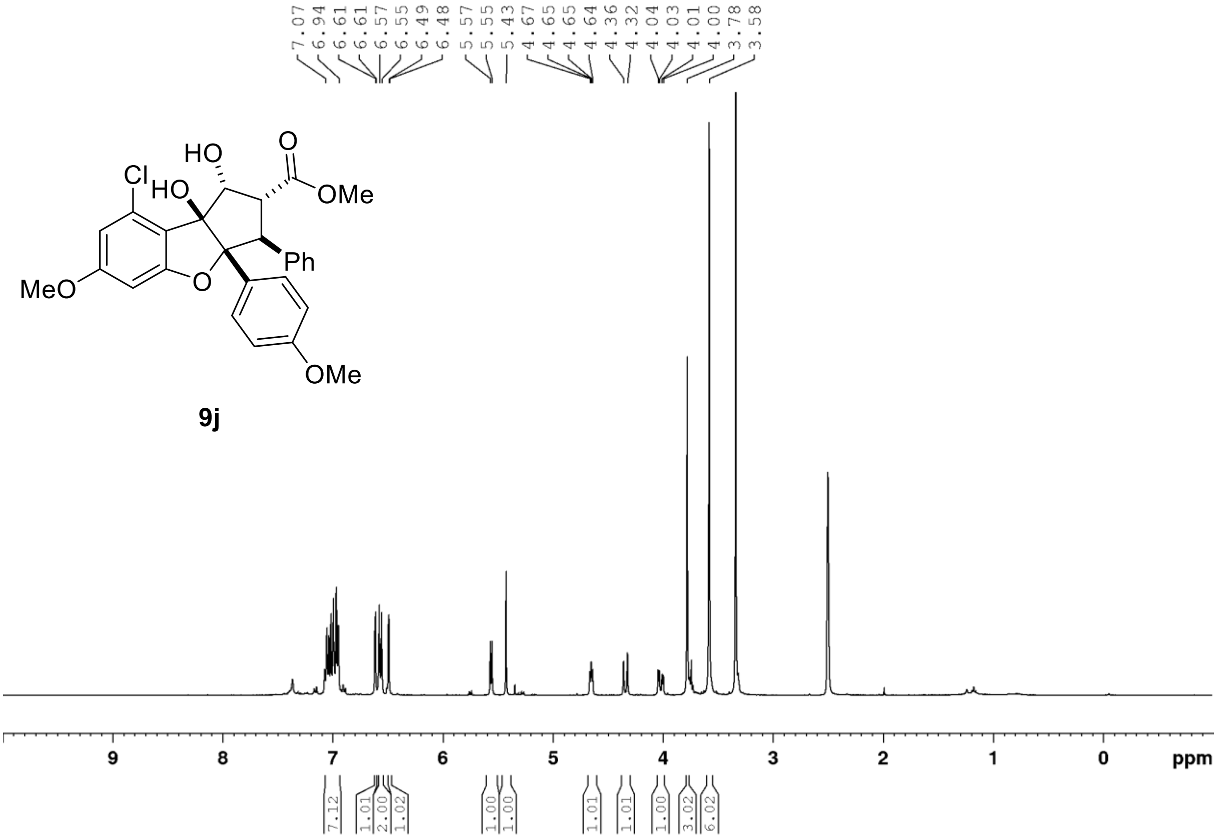

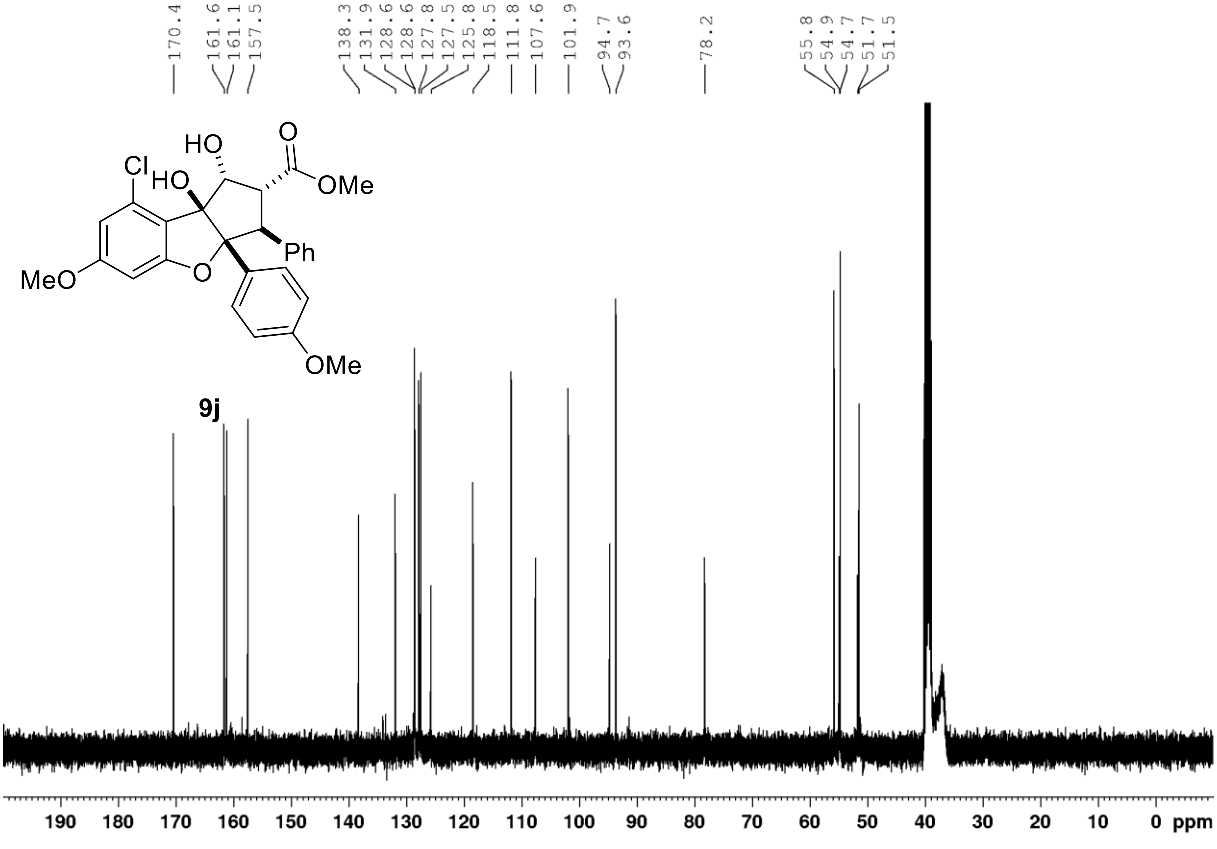

4.15. NMR spectroscopic data for the synthesis of 9k

(*E*)-1-(4-Chloro-2-hydroxy-6-methoxyphenyl)-3-(4-methoxyphenyl)prop-2-en-1-one (12k)

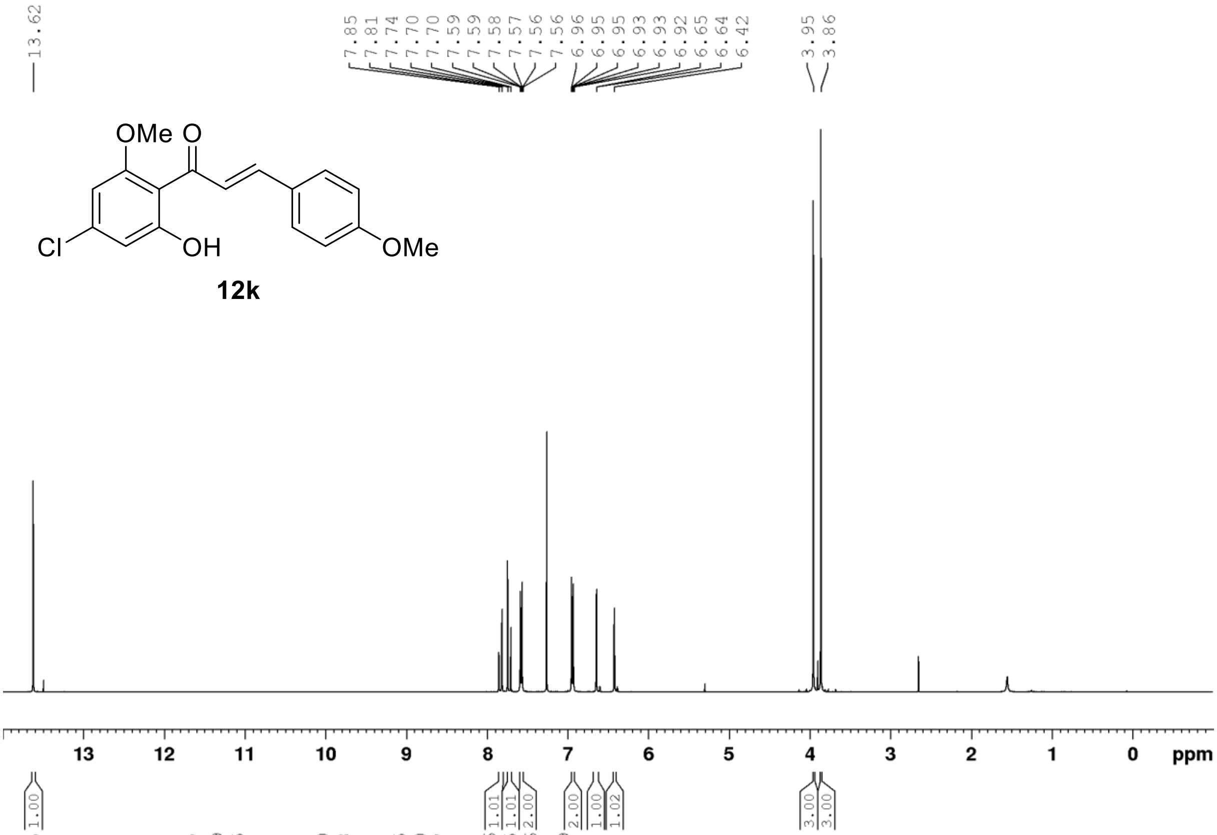

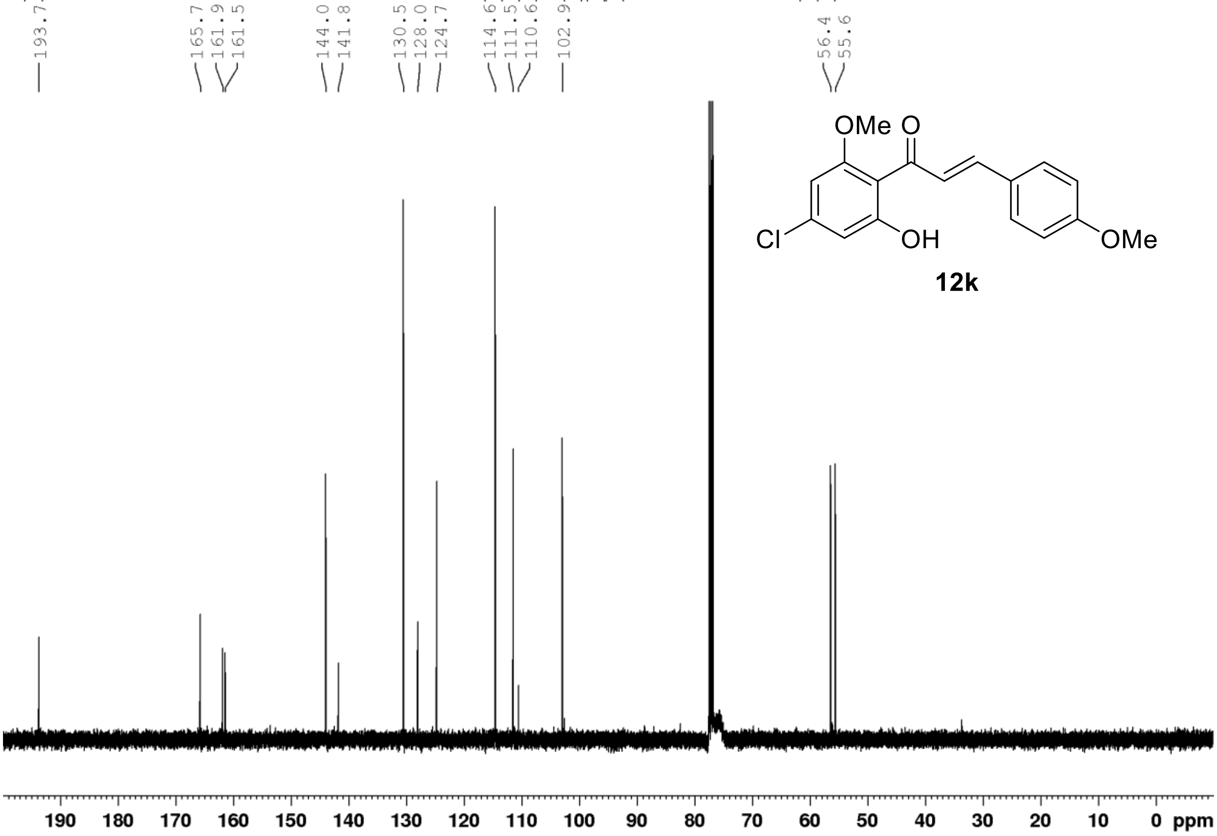

7-Chloro-3-hydroxy-5-methoxy-2-(4-methoxyphenyl)-4*H*-chromen-4-one (8k)

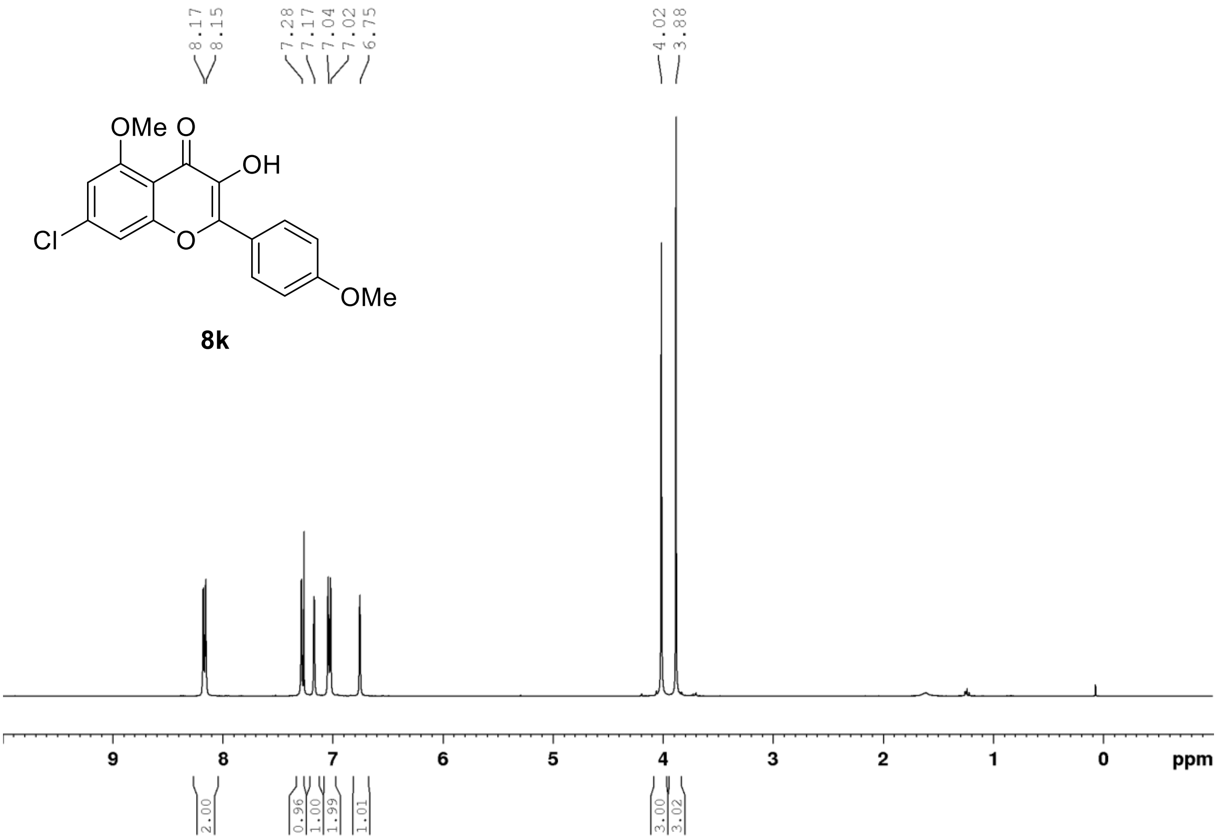

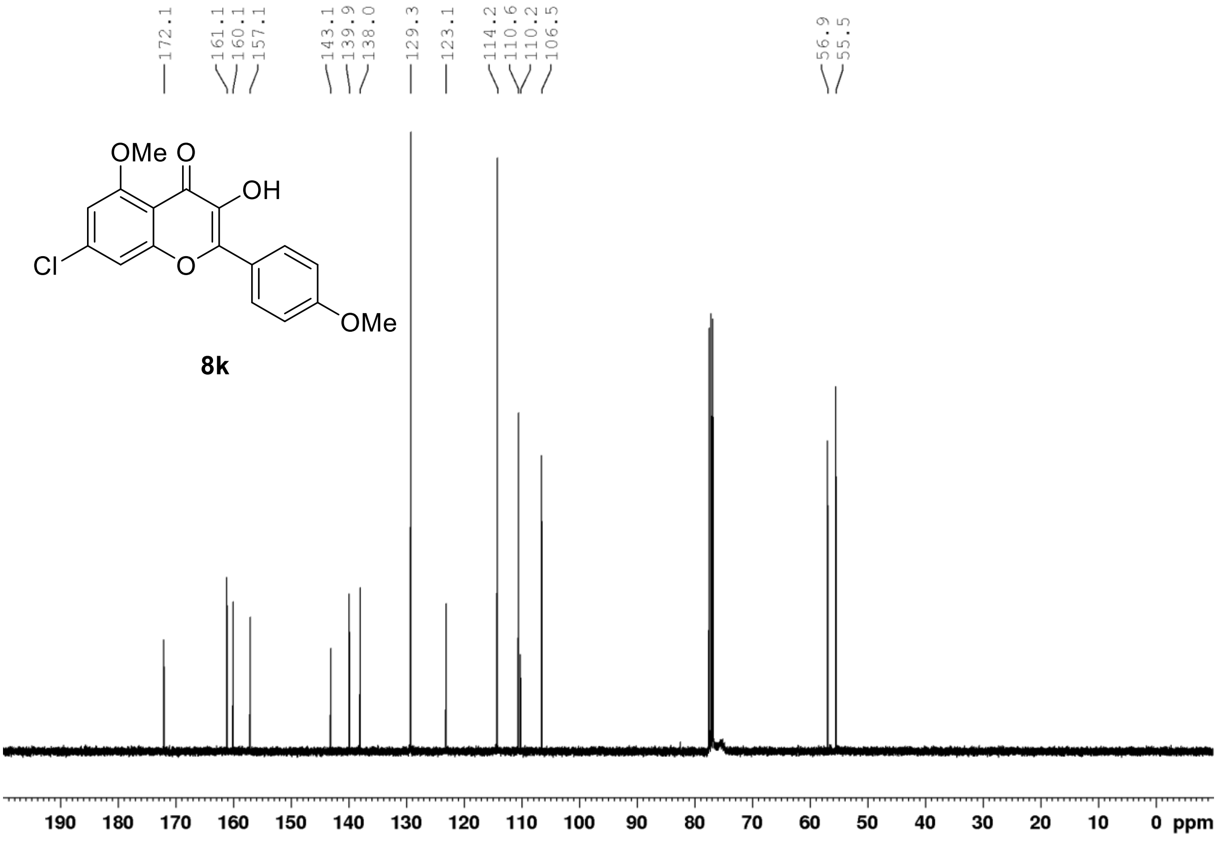

(±)-Methyl (1*R*,2*R*,3*S*,3a*R*,8b*S*)-6-chloro-1,8b-dihydroxy-8-methoxy-3a-(4-methoxyphenyl)-3-phenyl-2,3,3a,8b-tetrahydro-1*H*-cyclopenta[*b*]benzofuran-2-carboxylate (9k)

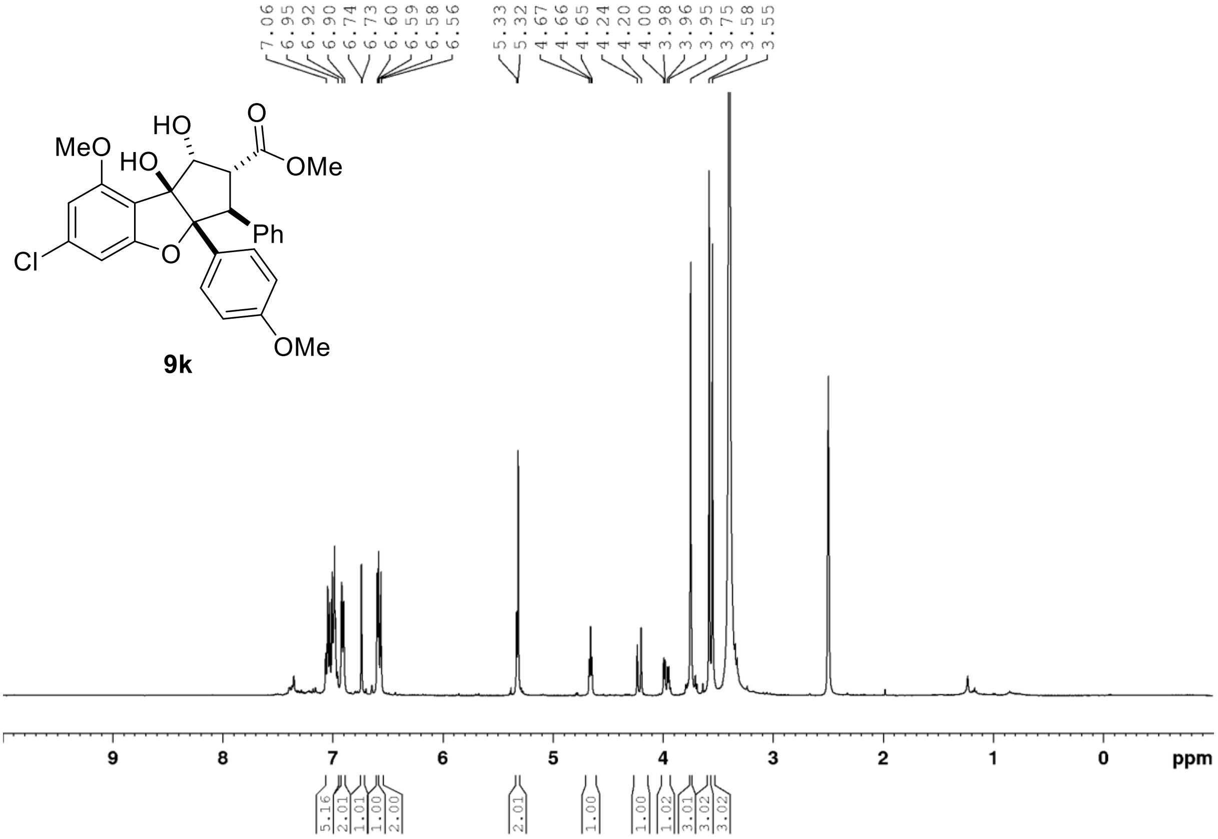

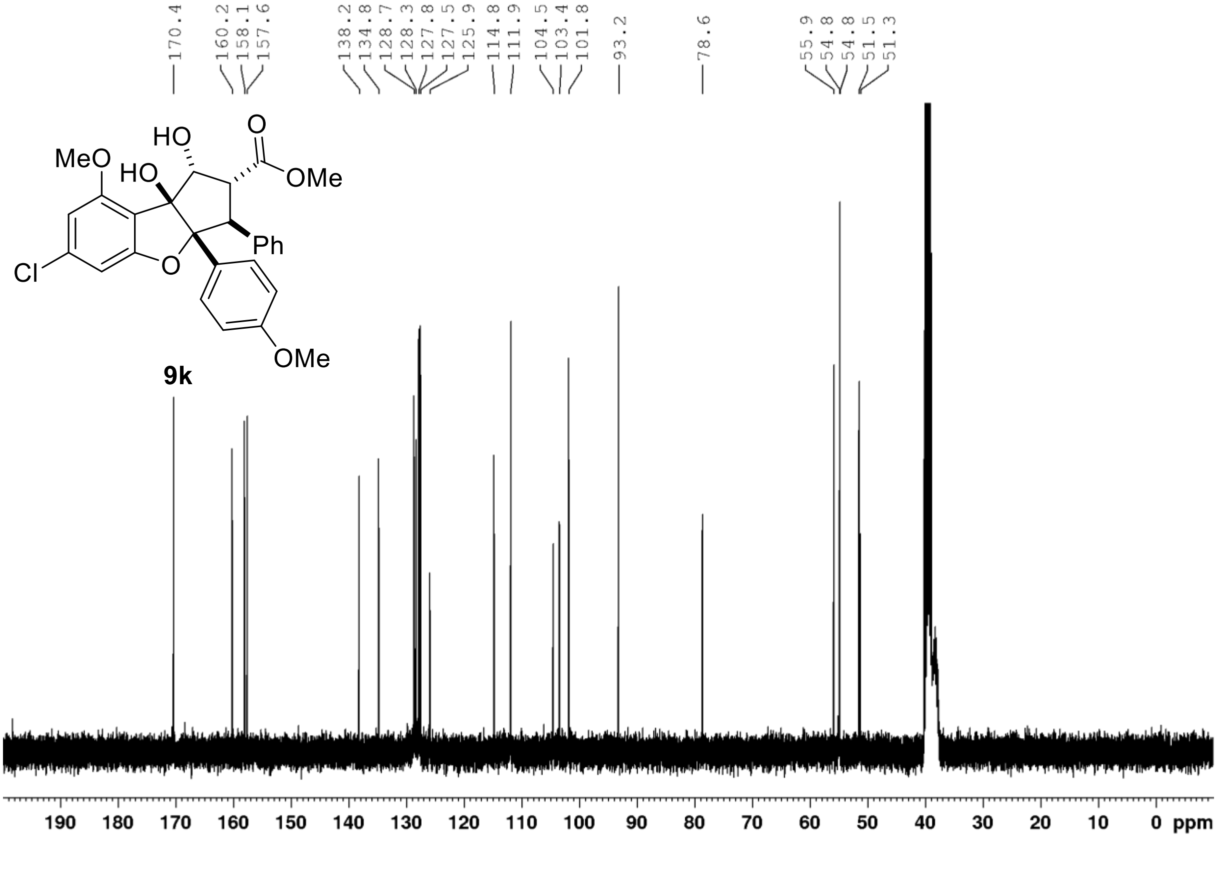

4.16. NMR spectroscopic data for the synthesis of 9l

(*E*)-1-(2-Bromo-6-hydroxy-4-methoxyphenyl)-3-(4-methoxyphenyl)prop-2-en-1-one (12l)

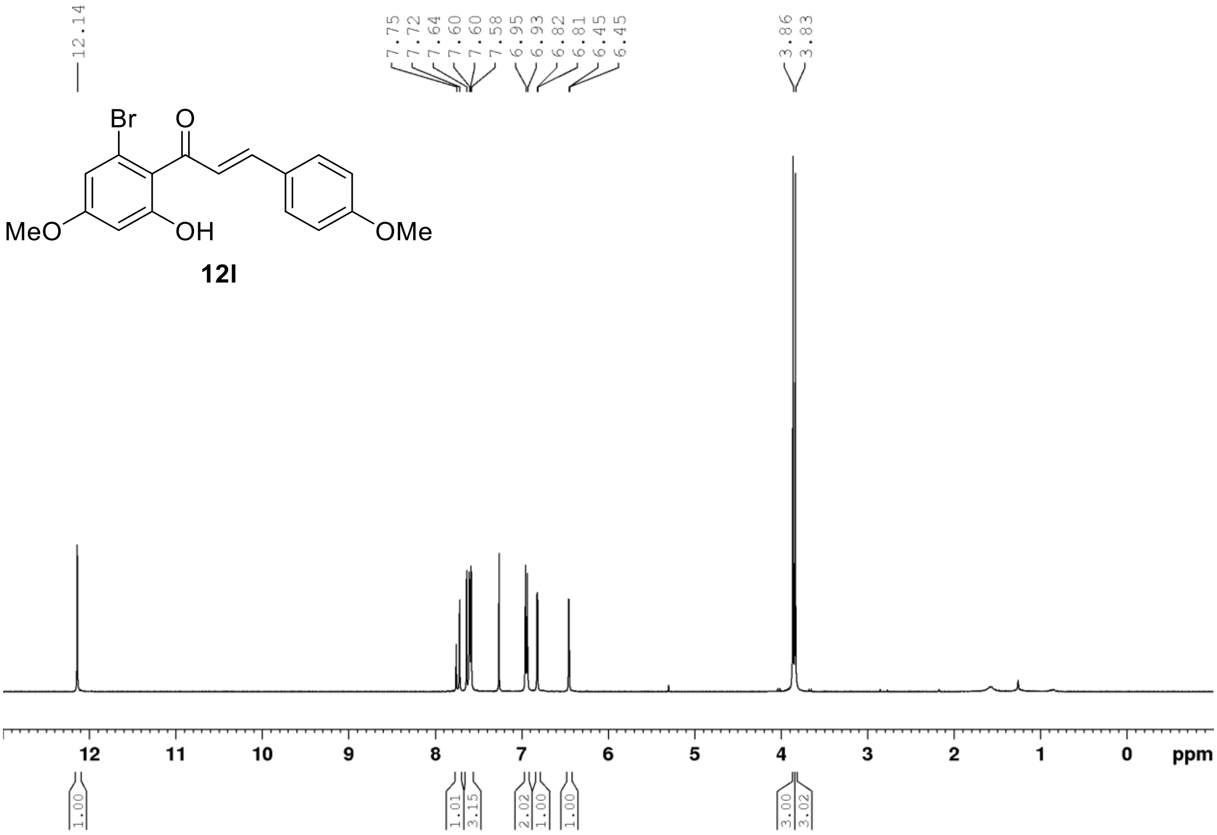

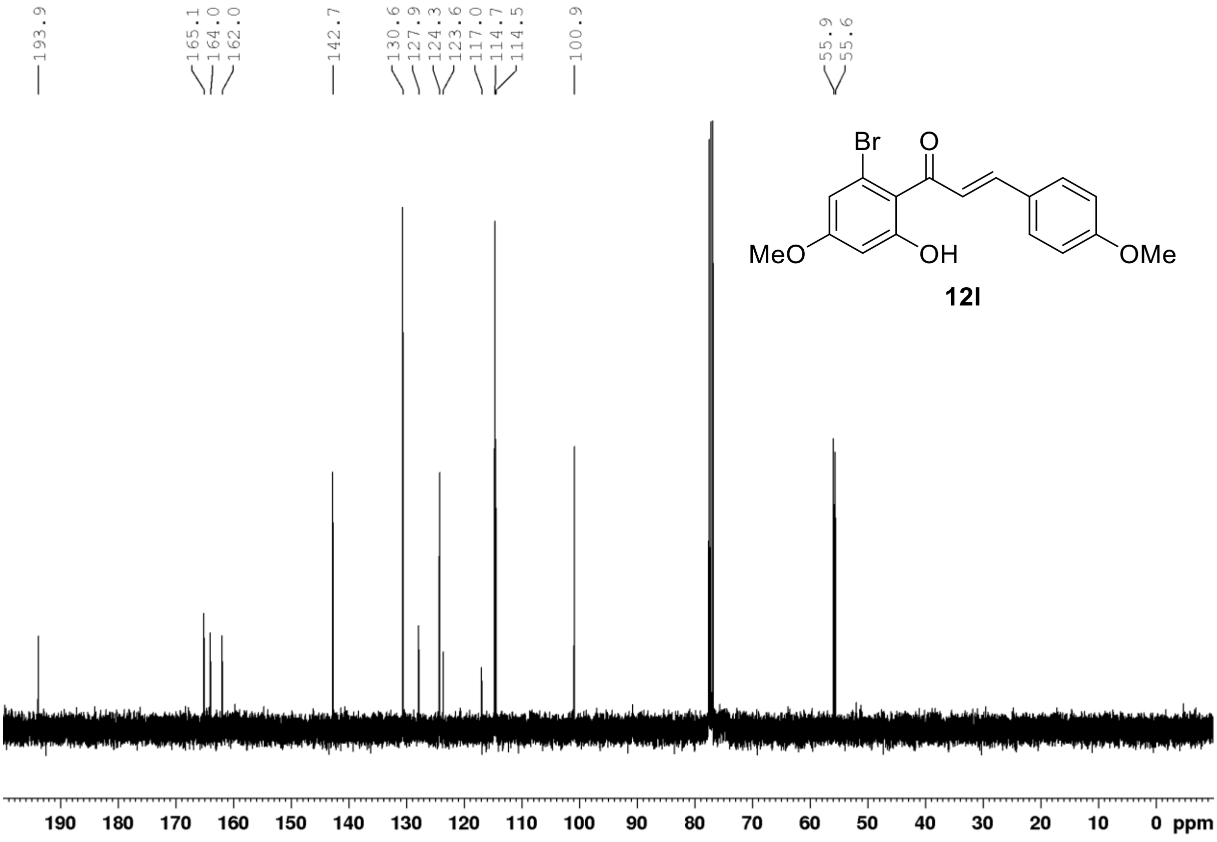

5-Bromo-3-hydroxy-7-methoxy-2-(4-methoxyphenyl)-4*H*-chromen-4-one (8l)

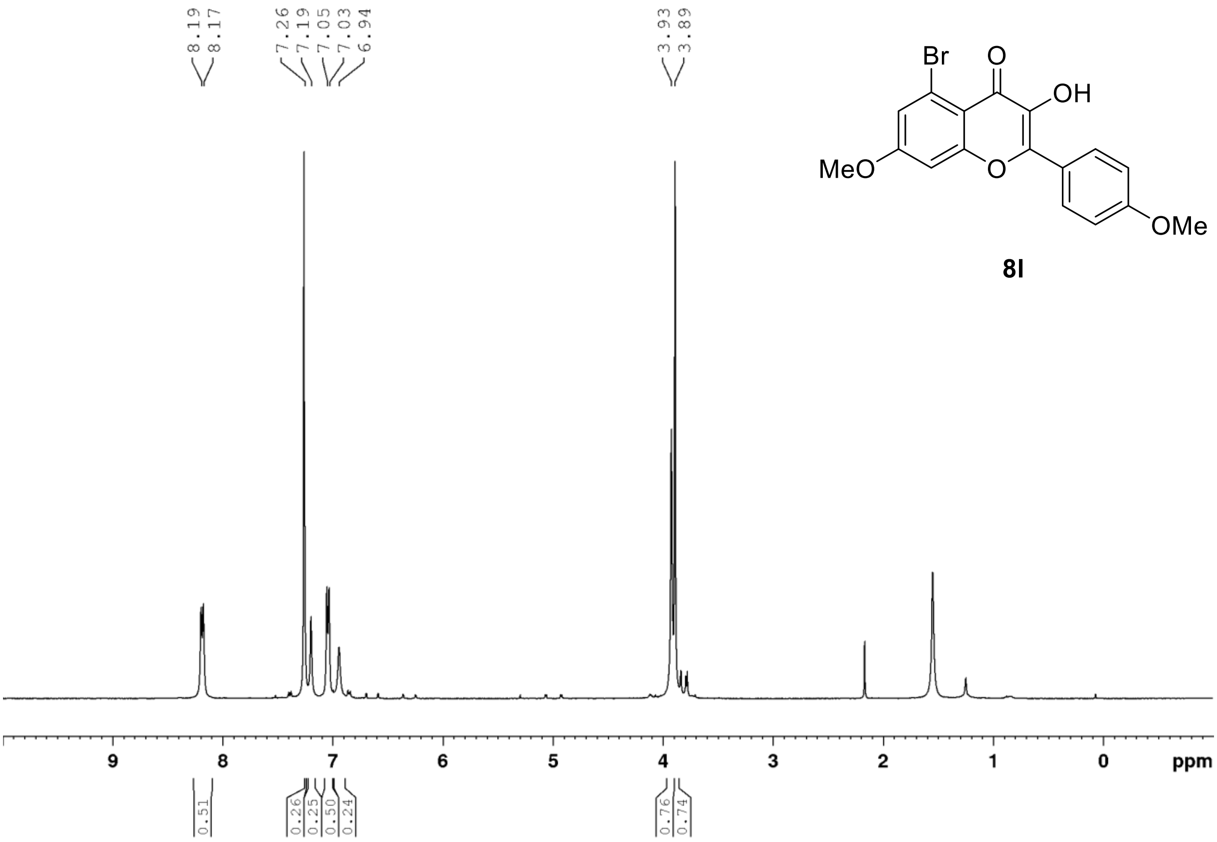

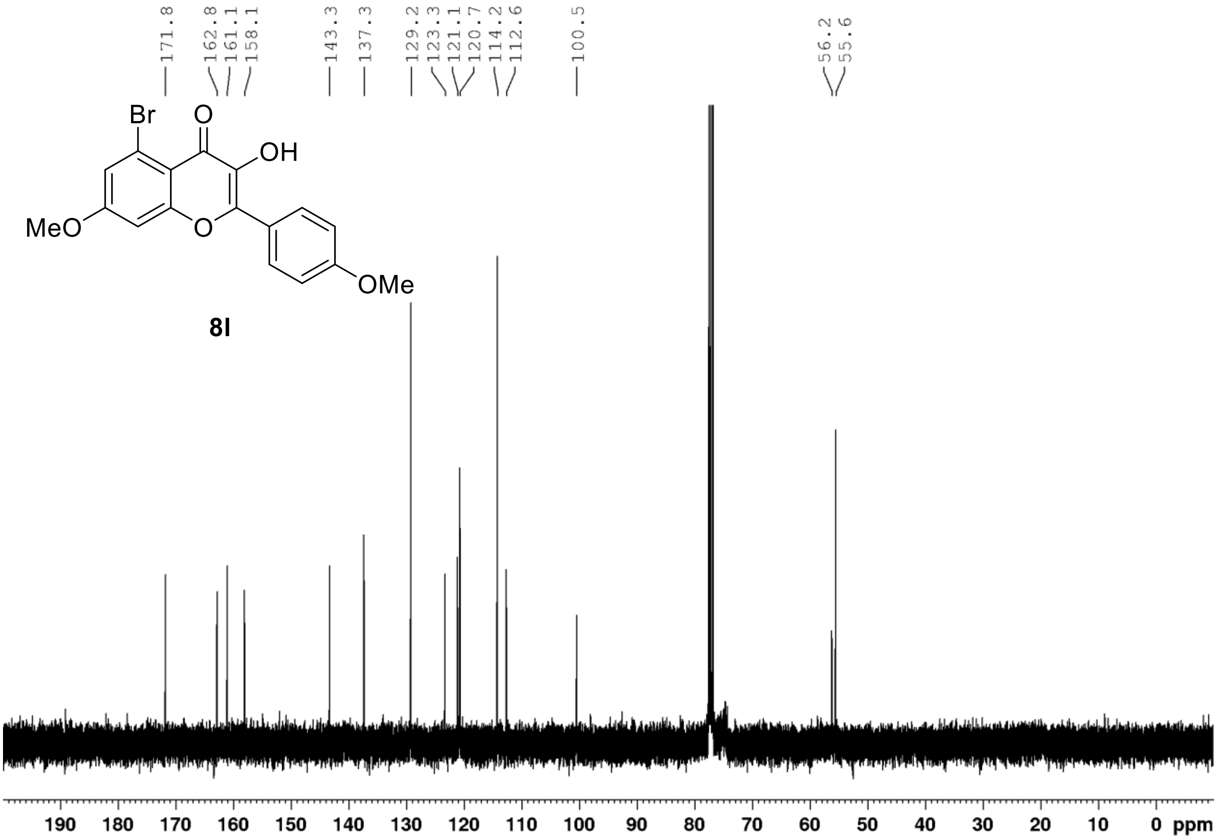

(±)-Methyl (1*R*,2*R*,3*S*,3a*R*,8b*S*)-8-bromo-1,8b-dihydroxy-6-methoxy-3a-(4-methoxyphenyl)-3-phenyl-2,3,3a,8b-tetrahydro-1*H*-cyclopenta[*b*]benzofuran-2-carboxylate (9l)

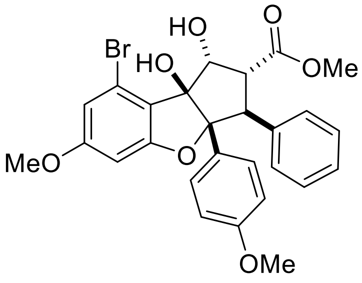

9l

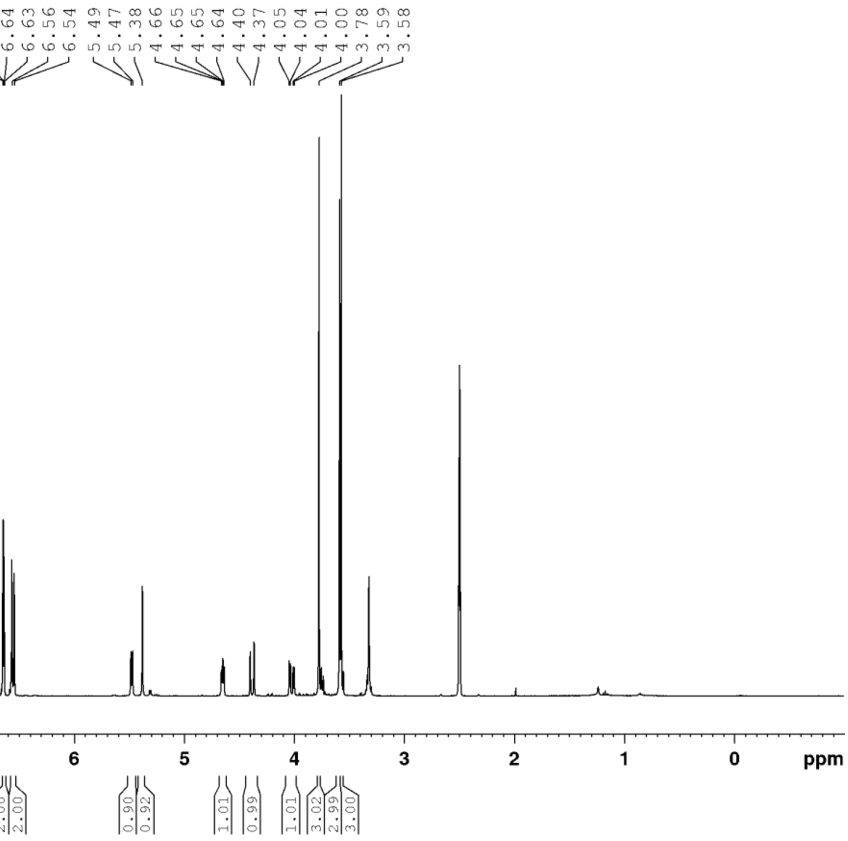

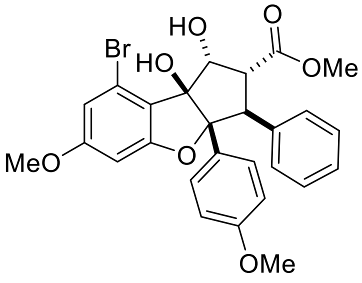

9l

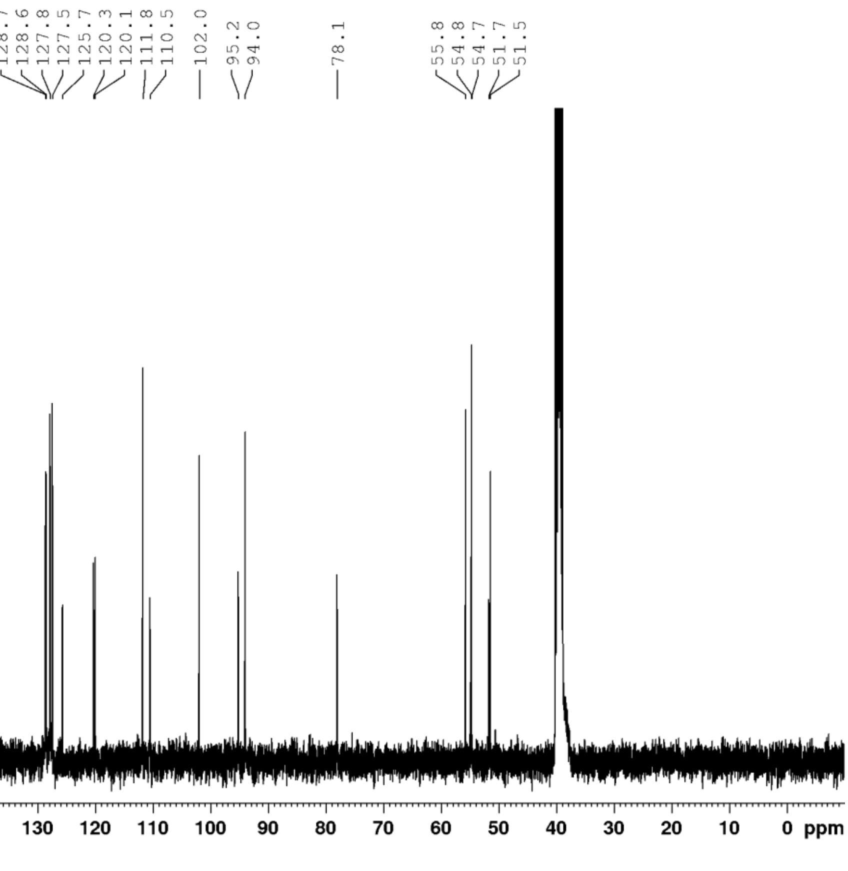

4.17. NMR spectroscopic data for the synthesis of 9m

(*E*)-1-(4-Bromo-2-hydroxy-6-methoxyphenyl)-3-(4-methoxyphenyl)prop-2-en-1-one (12m)

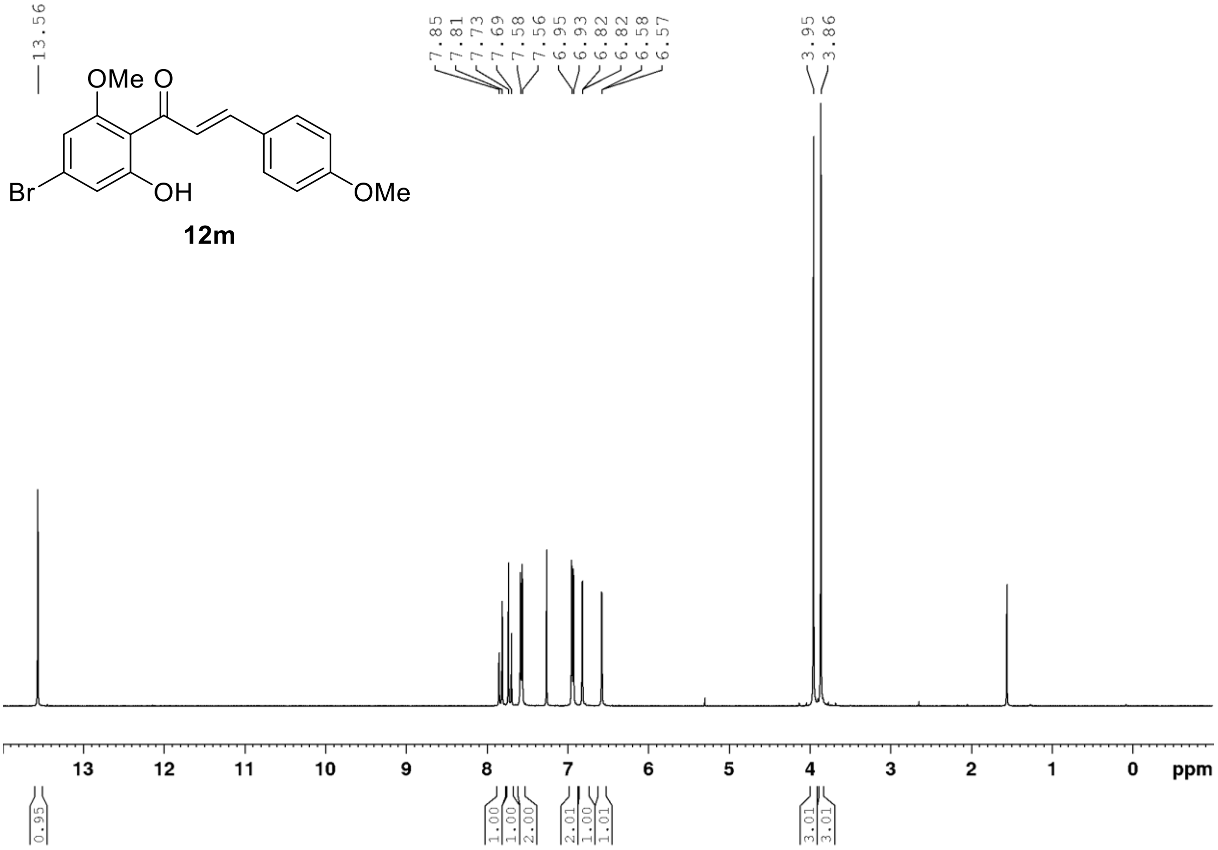

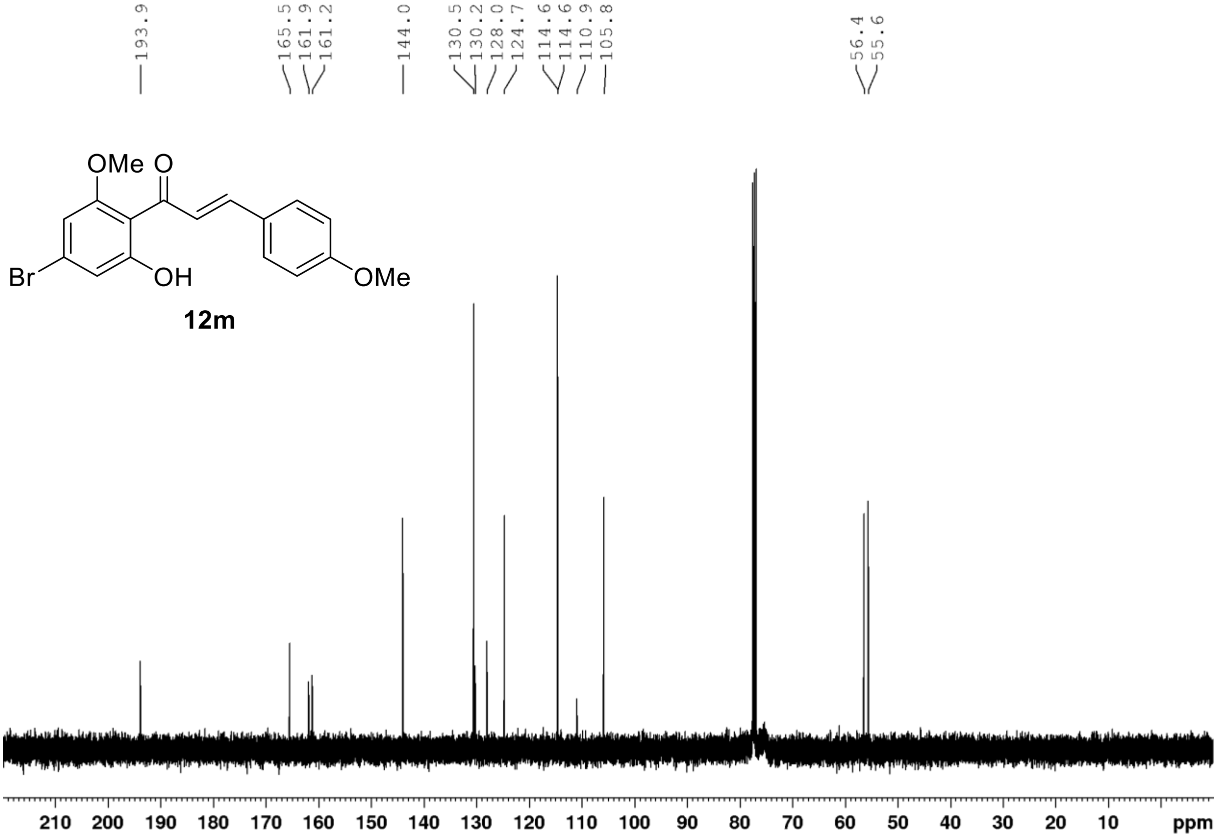

7-Bromo-3-hydroxy-5-methoxy-2-(4-methoxyphenyl)-4*H*-chromen-4-one (8m)

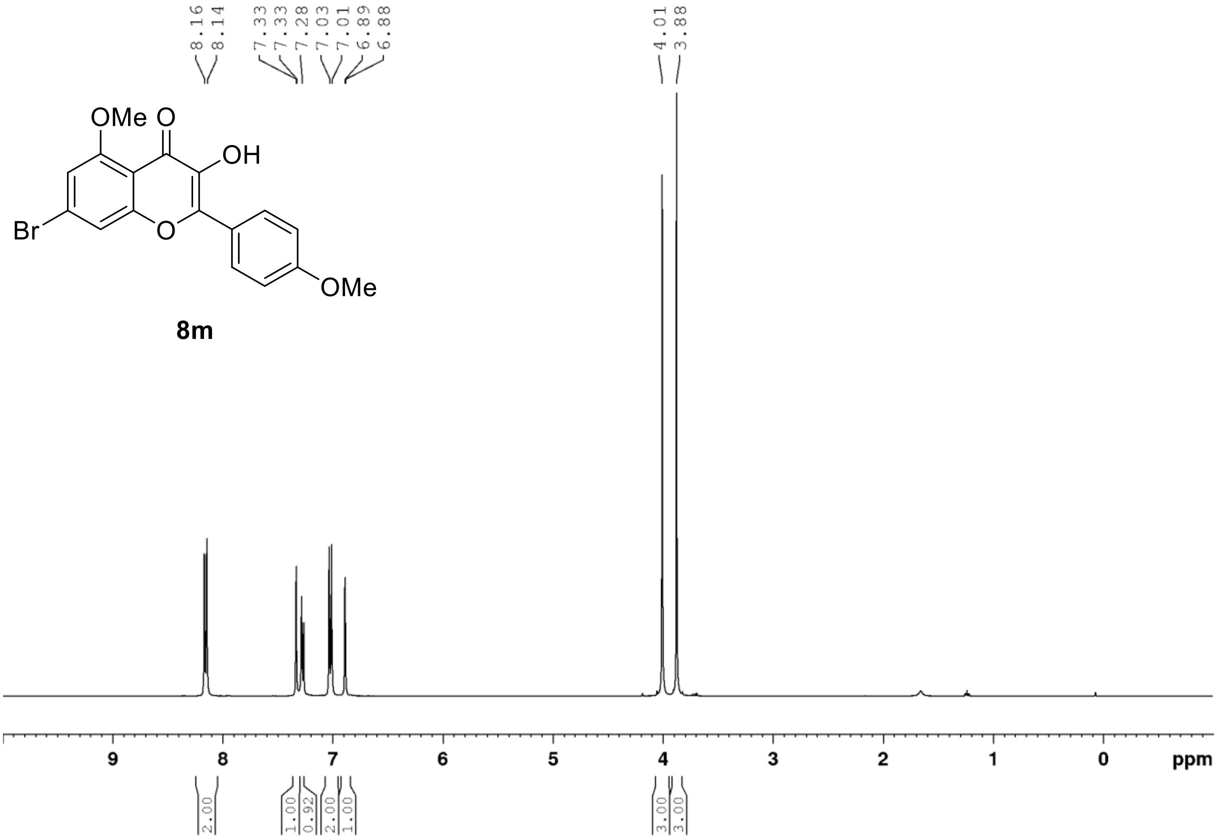

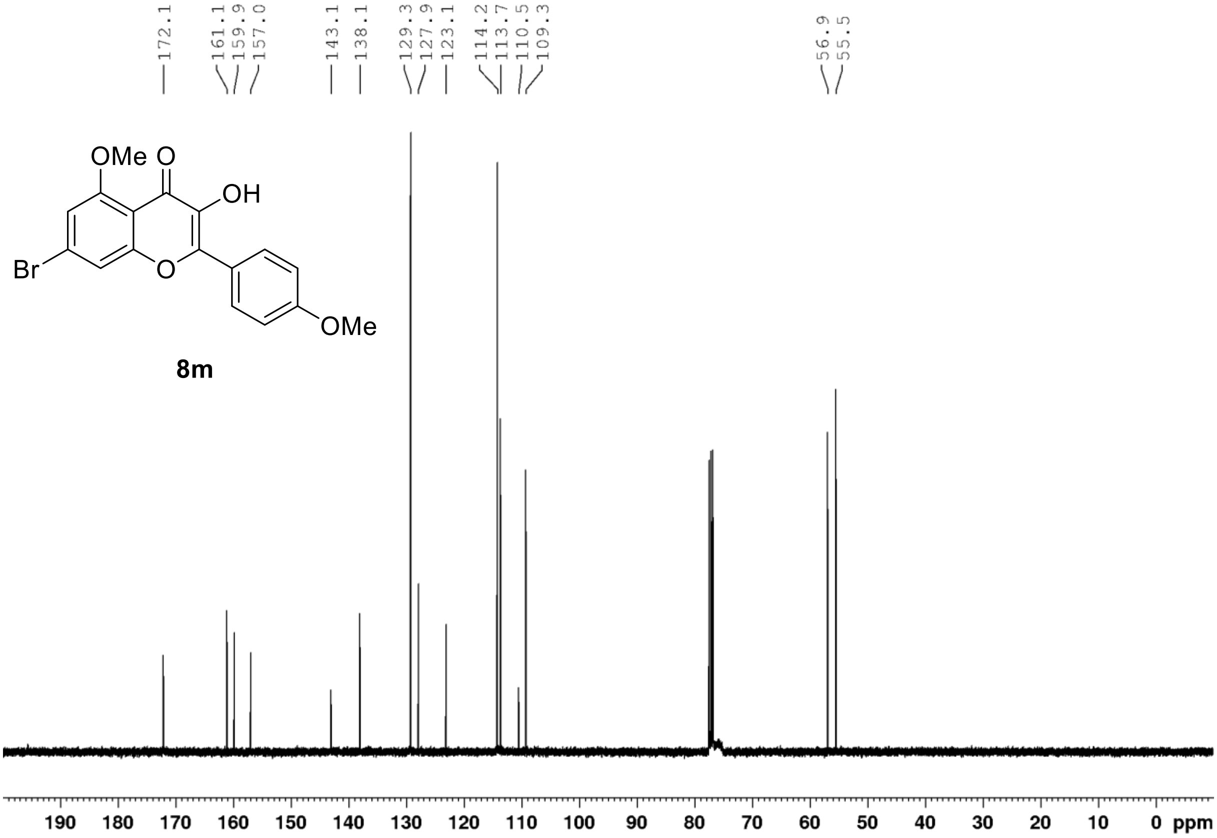

(±)-Methyl (1*R*,2*R*,3*S*,3a*R*,8b*S*)-6-bromo-1,8b-dihydroxy-8-methoxy-3a-(4-methoxyphenyl)-3-phenyl-2,3,3a,8b-tetrahydro-1*H*-cyclopenta[*b*]benzofuran-2-carboxylate (9m)

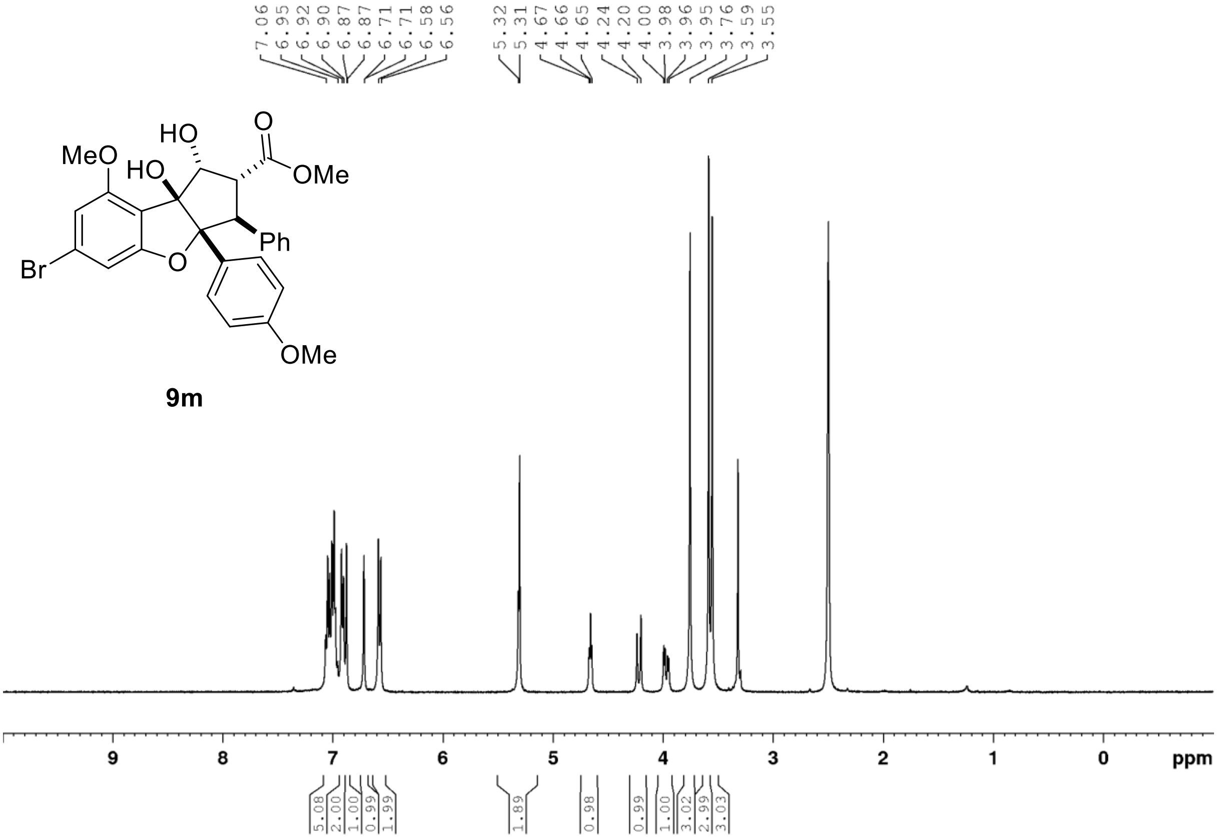

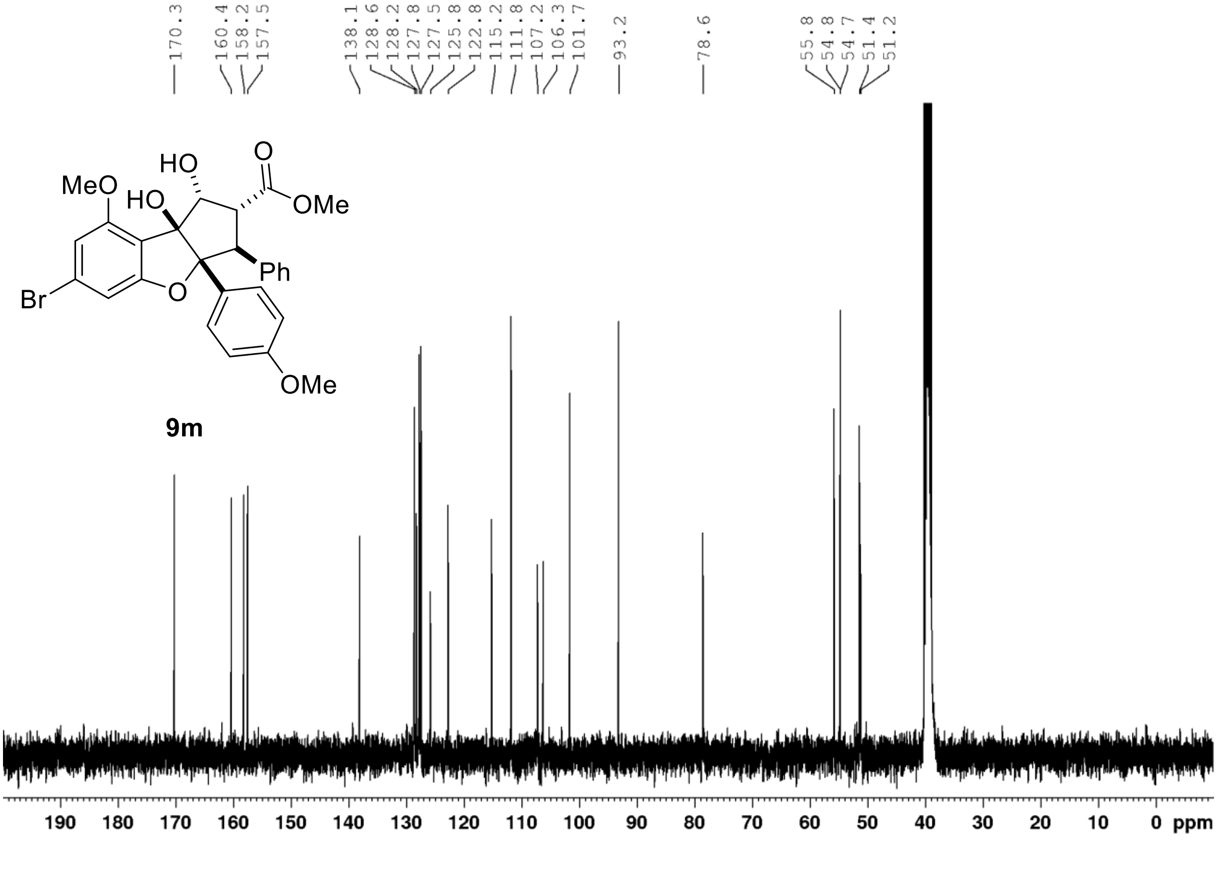

4.18. NMR spectroscopic data for the synthesis of 9na

(*E*)-1-(2-Fluoro-6-hydroxy-4-(methoxymethoxy)phenyl)-3-(4-methoxyphenyl)prop-2-en-1-one (12na)

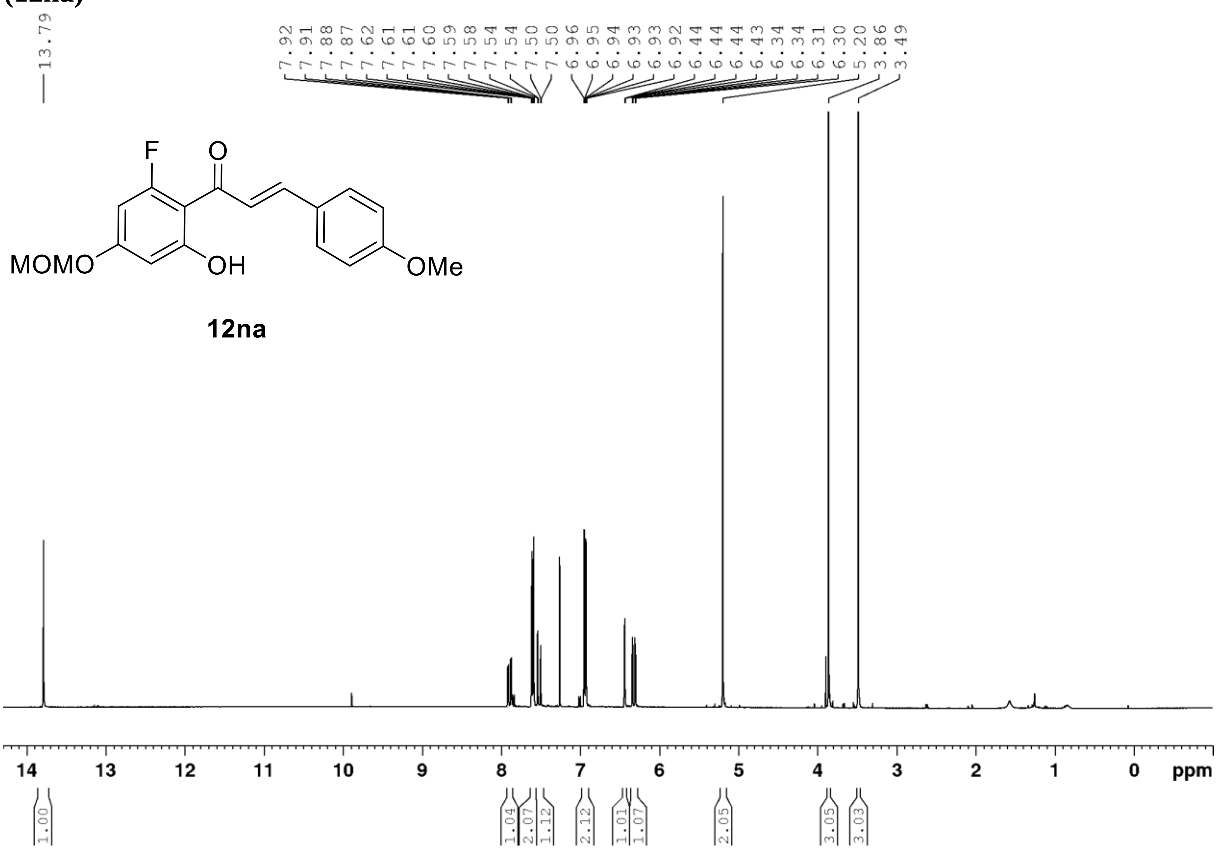

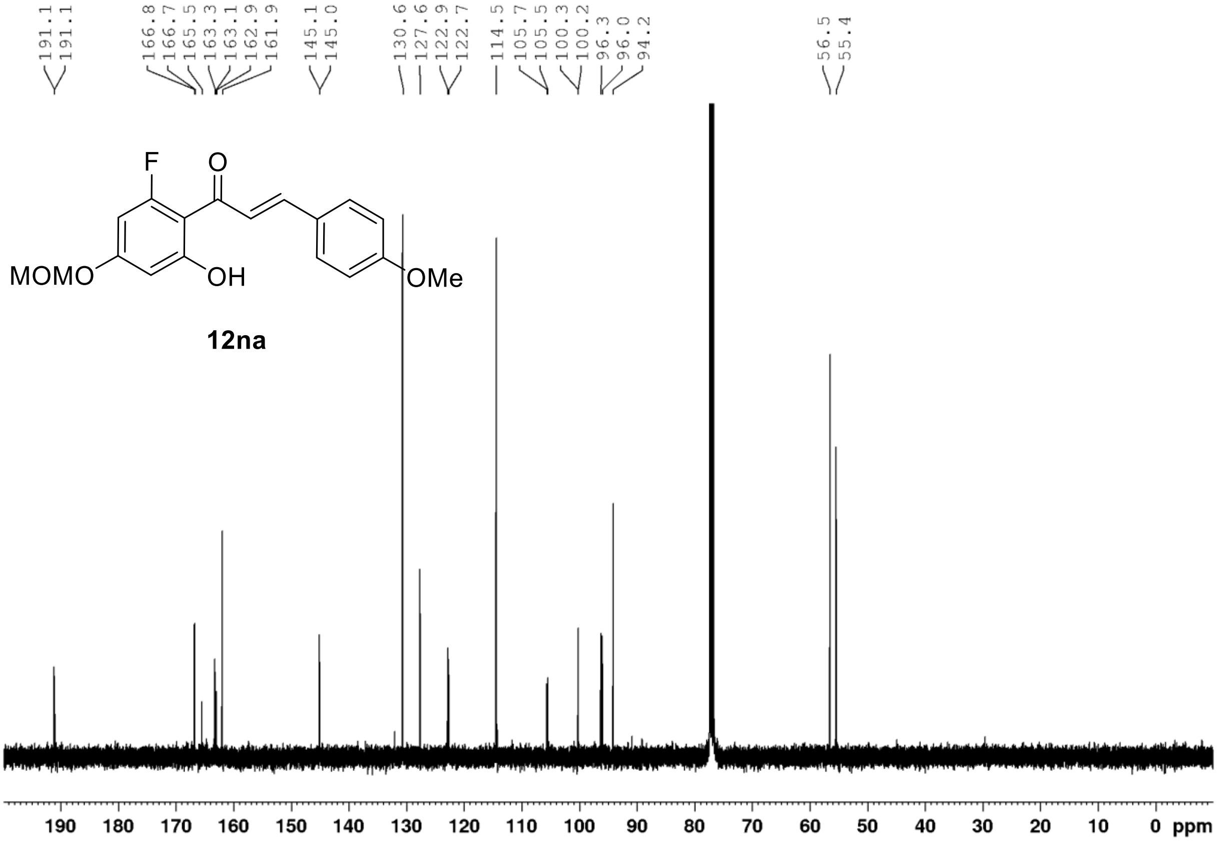

5-Fluoro-3-hydroxy-7-(methoxymethoxy)-2-(4-methoxyphenyl)-4*H*-chromen-4-one (SF24)

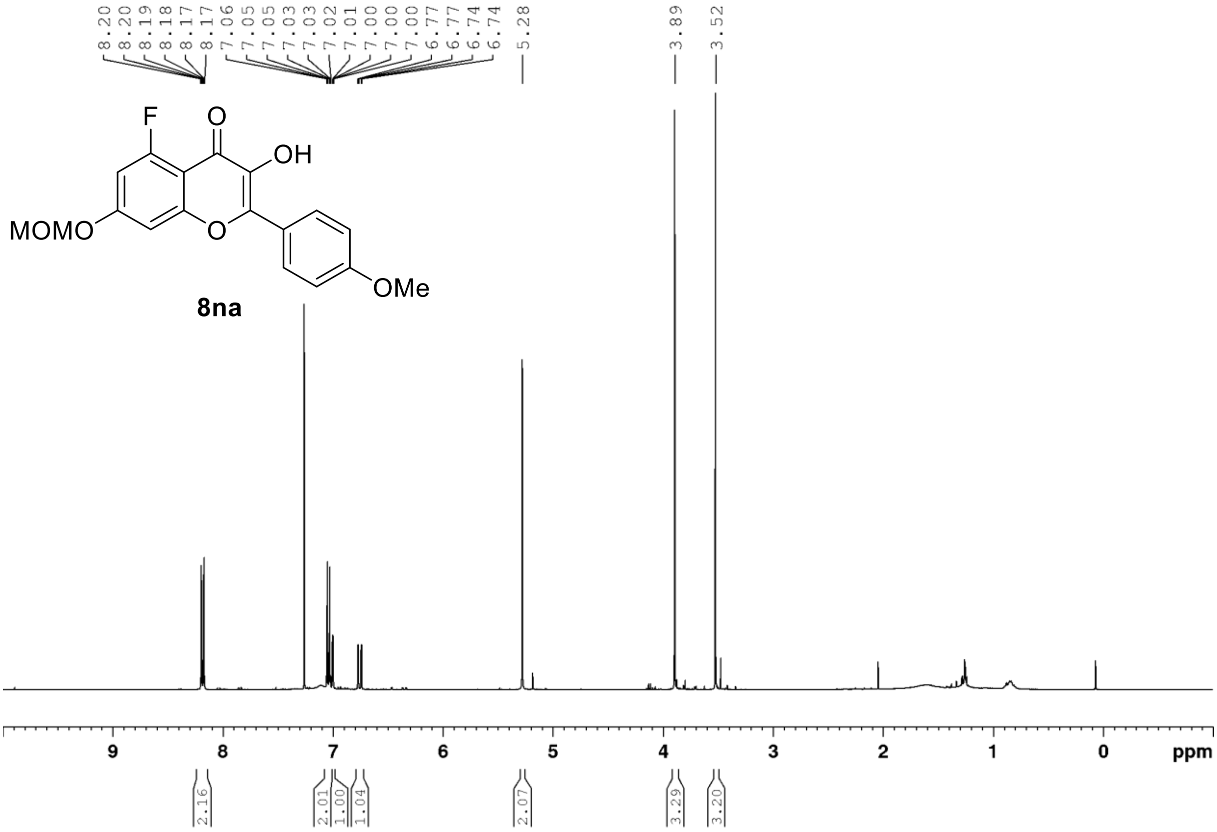

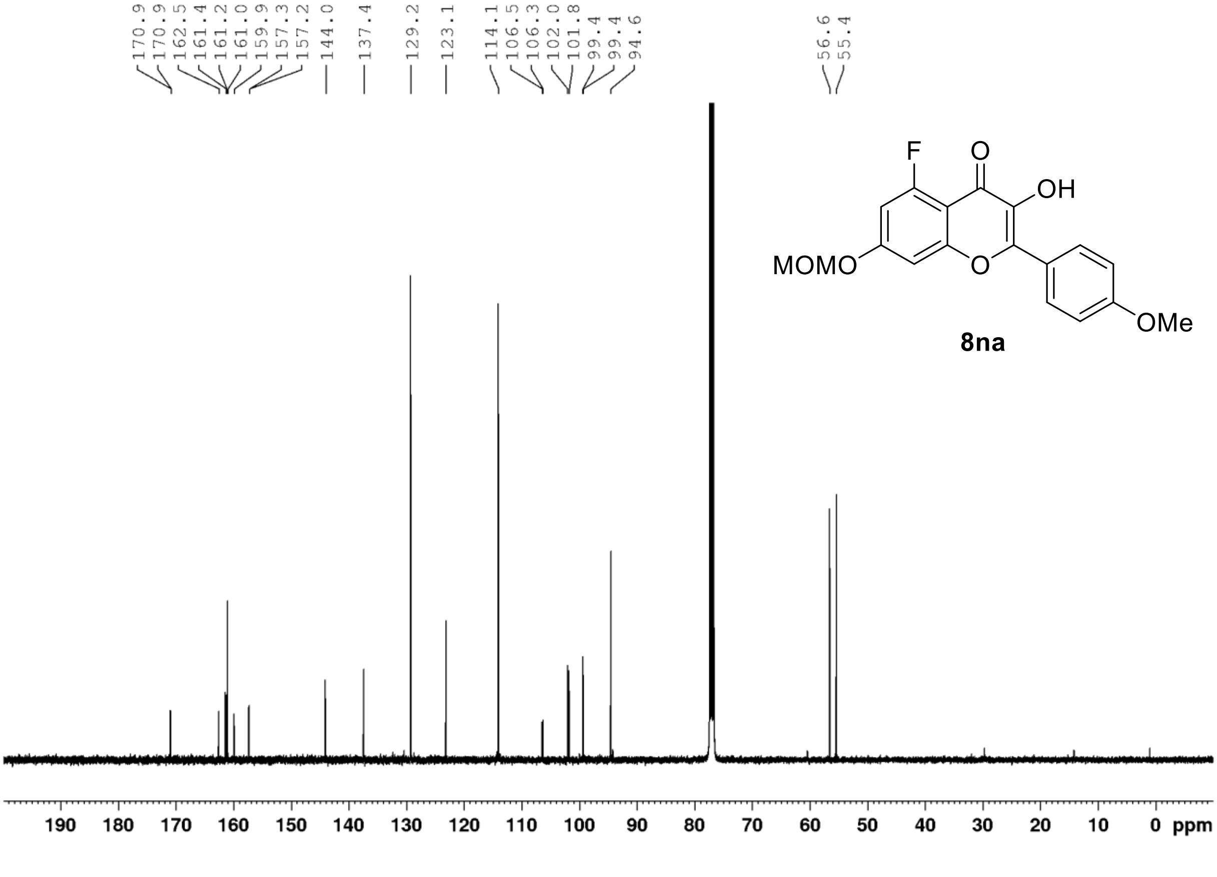

Methyl (1*R*,2*R*,3*S*,3a*R*,8b*S*)-8-fluoro-1,8b-dihydroxy-6-(methoxymethoxy)-3a-(4-methoxyphenyl)-3-phenyl-2,3,3a,8b-tetrahydro-1*H*-cyclopenta[*b*]benzofuran-2-carboxylate (9na)

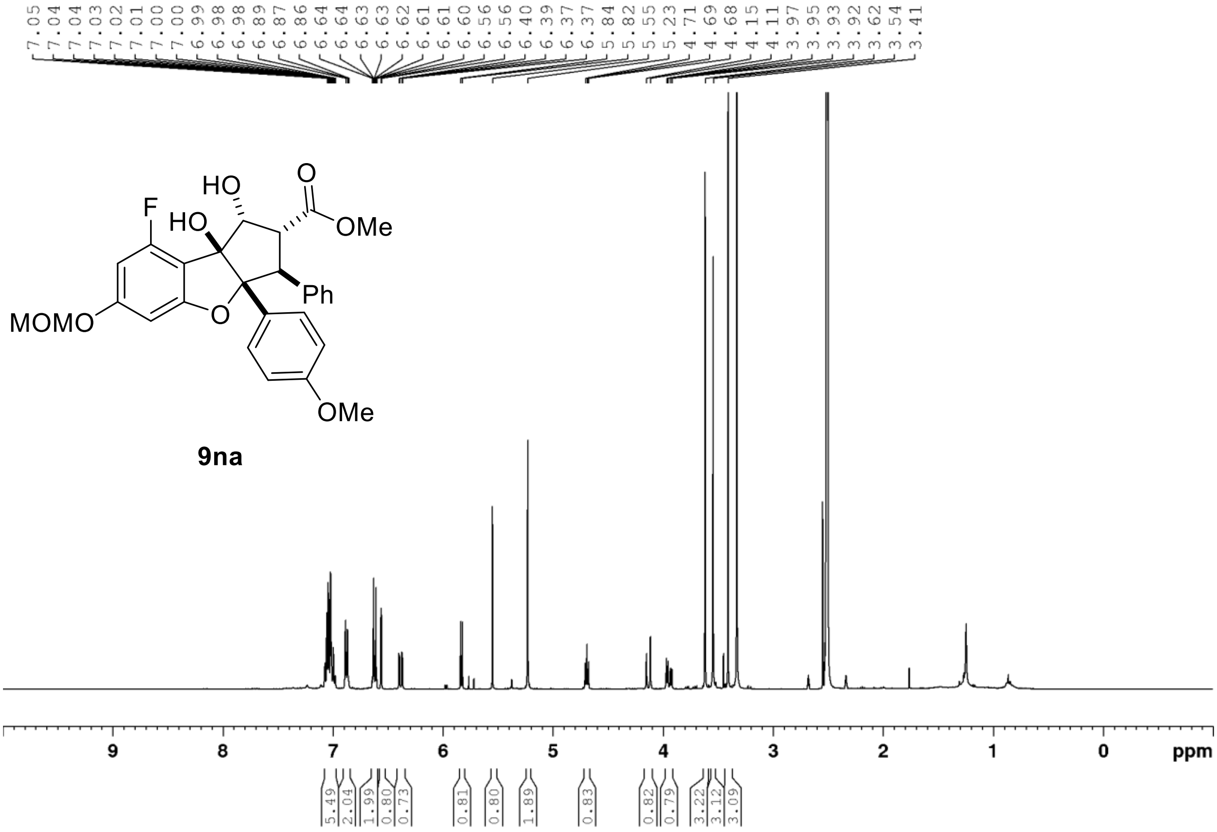

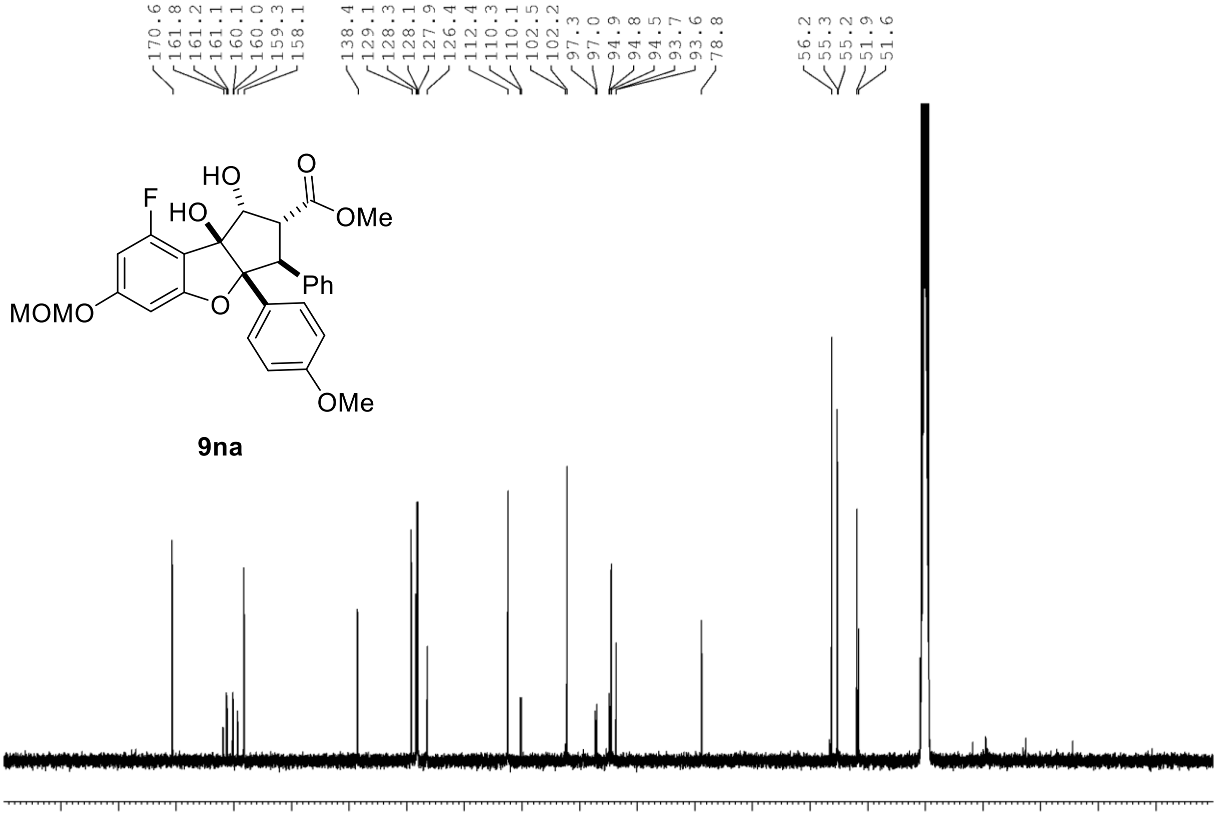

4.19. NMR spectroscopic data for the synthesis of 9nb

(*E*)-3-(4-bromophenyl)-1-(2-fluoro-6-hydroxy-4-(methoxymethoxy)phenyl)prop-2-en-1-one (12nb)

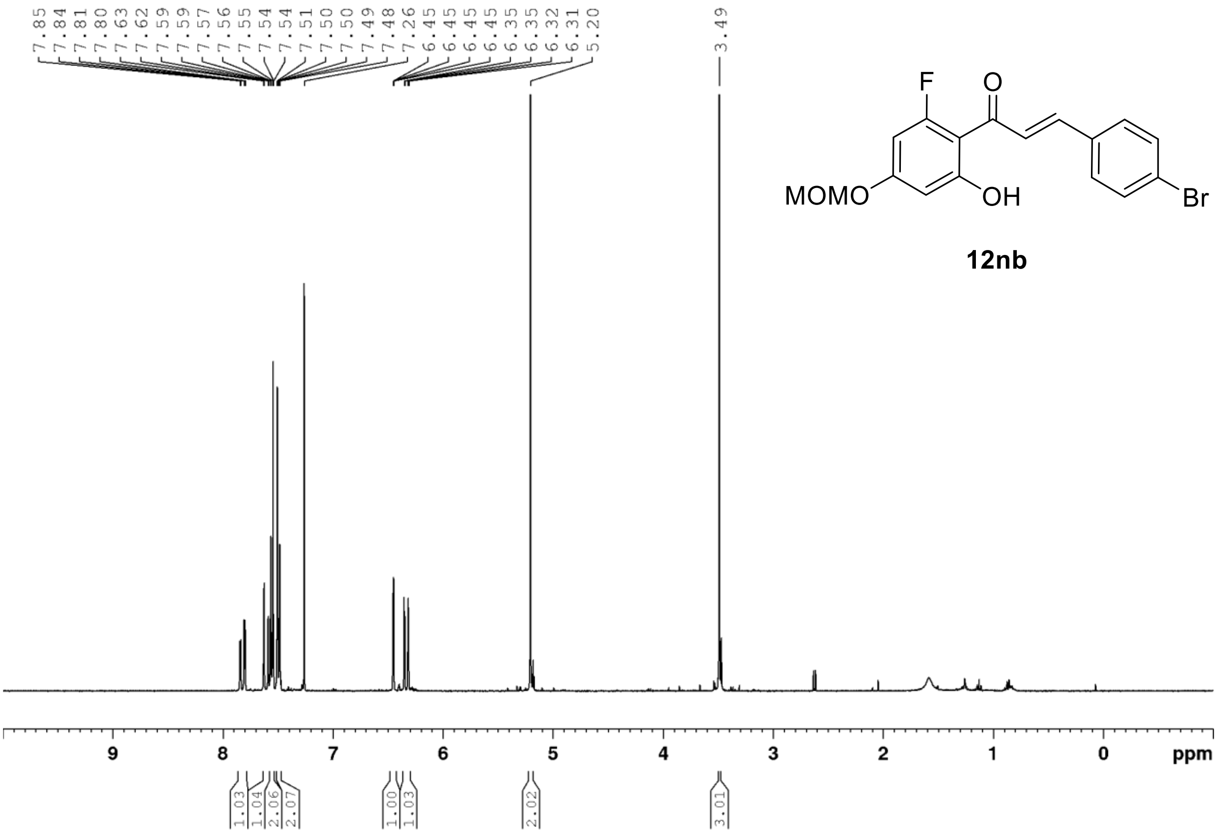

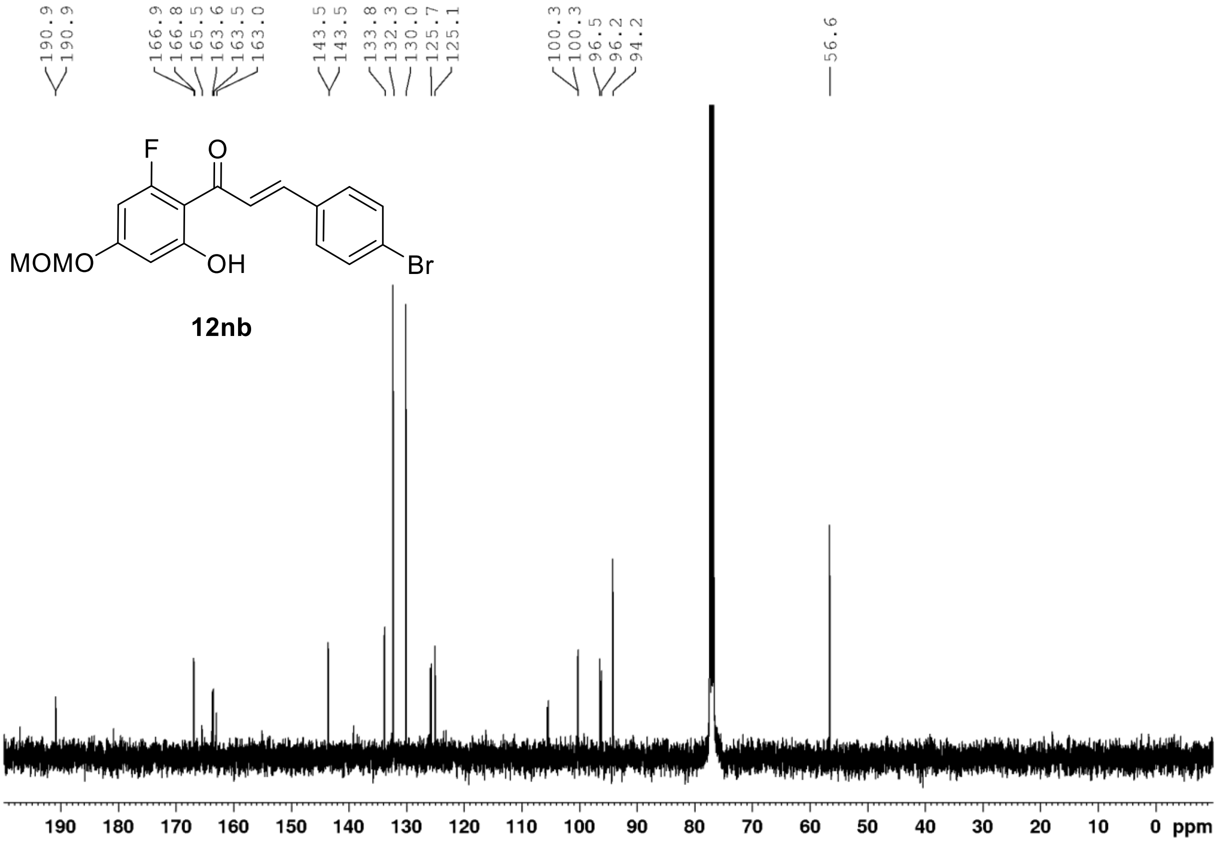

2-(4-Bromophenyl)-5-fluoro-3-hydroxy-7-(methoxymethoxy)-4*H*-chromen-4-one (8nb)

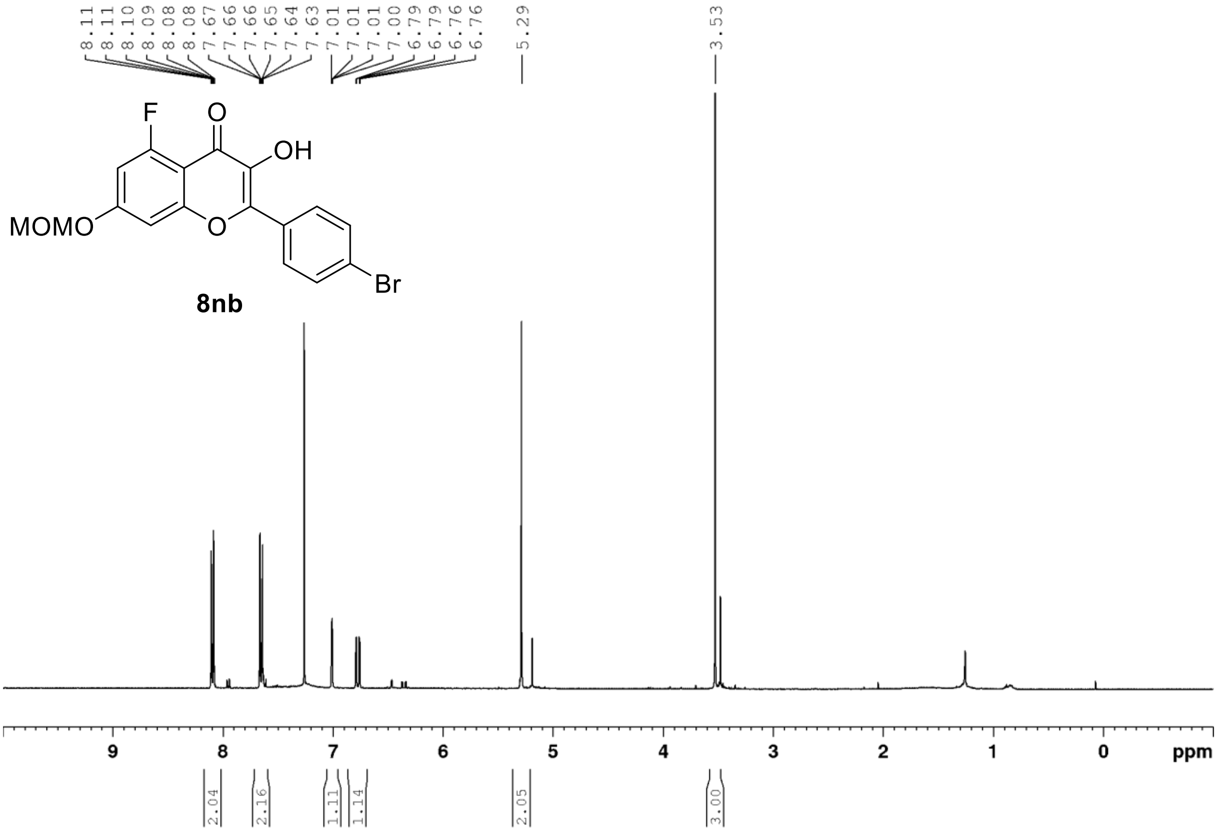

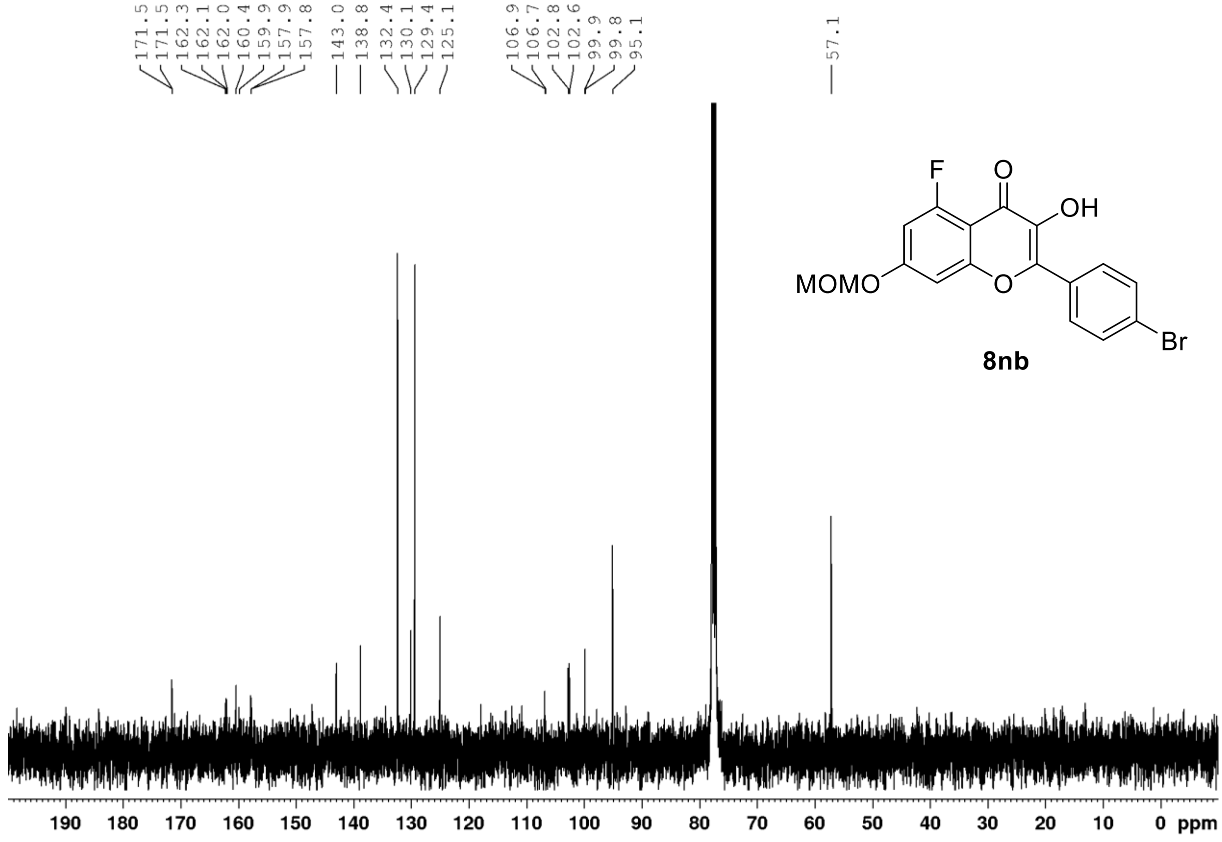

Methyl (1*R*,2*R*,3*S*,3a*R*,8b*S*)-3a-(4-bromophenyl)-8-fluoro-1,8b-dihydroxy-6-(methoxymethoxy)-3-phenyl-2,3,3a,8b-tetrahydro-1*H*-cyclopenta[*b*]benzofuran-2-carboxylate (9nb)

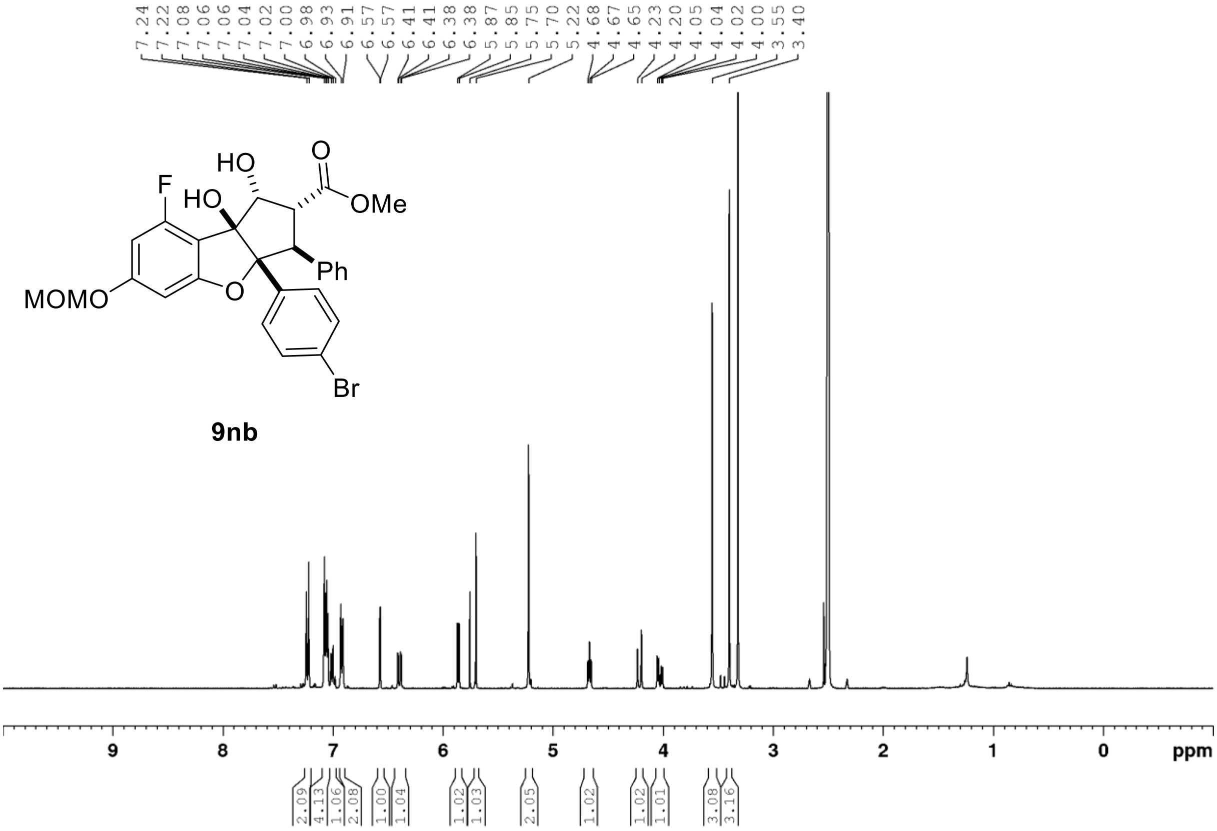

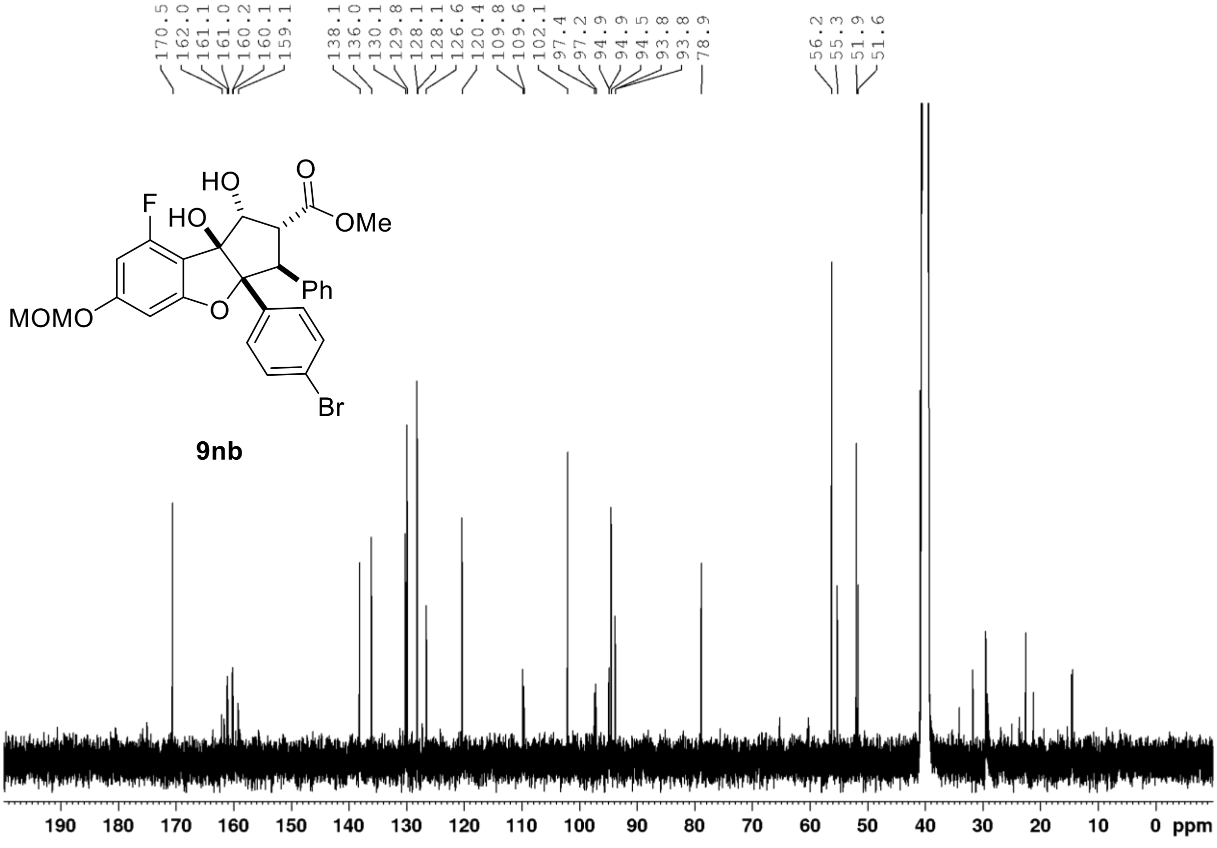

4.20. NMR spectroscopic data for the synthesis of 14aa

(±)- (1*R*,2*R*,3*S*,3a*R*,8b*S*)-3a-(4-chlorophenyl)-1,8b-dihydroxy-6,8-dimethoxy-*N*,*N*-dimethyl-3-phenyl-2,3,3a,8b-tetrahydro-1*H*-cyclopenta[*b*]benzofuran-2-carboxamide (14aa)

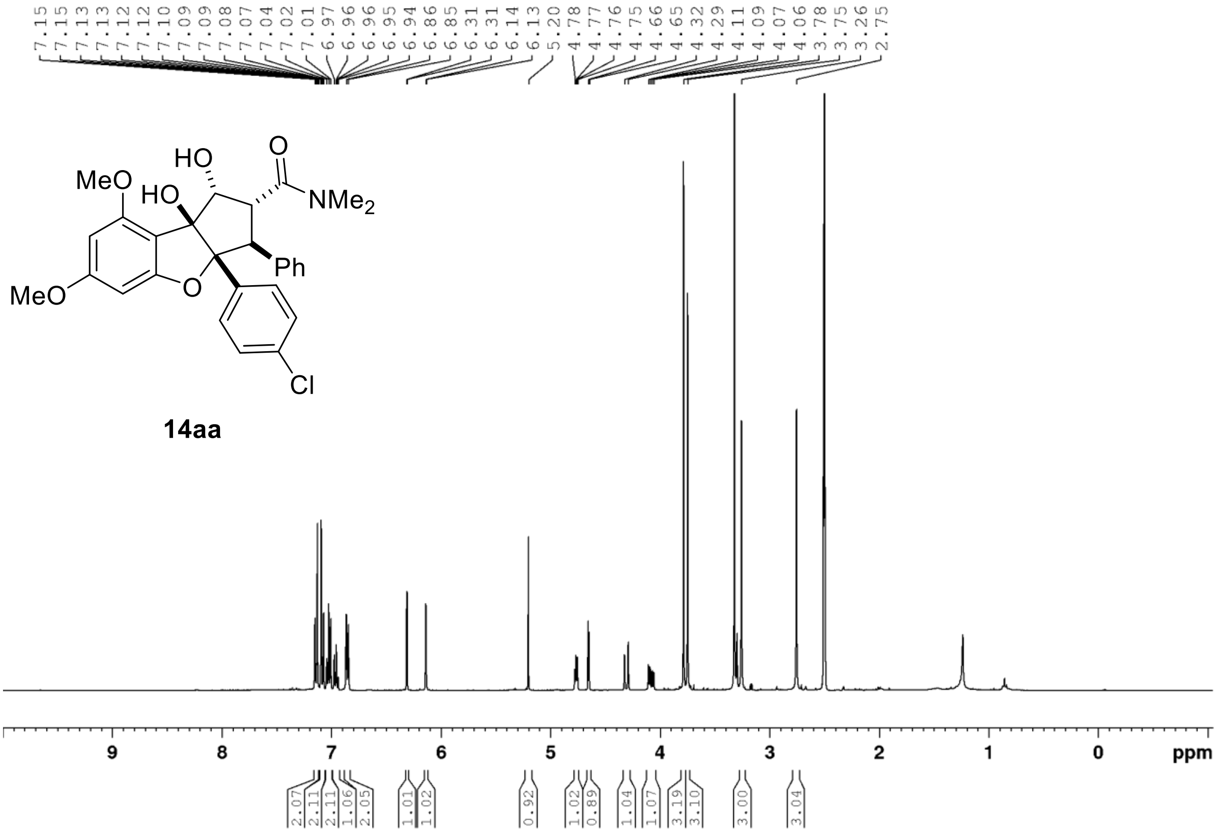

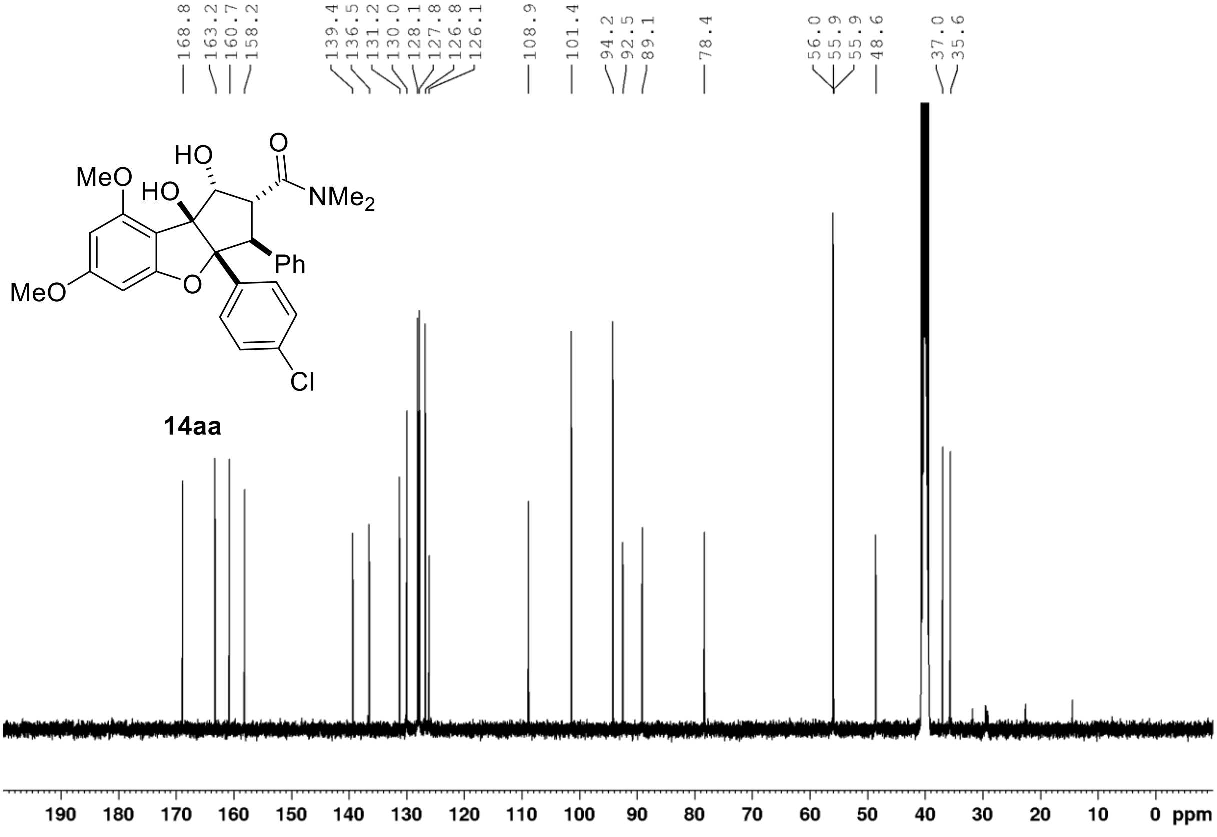

4.21. NMR spectroscopic data for the synthesis of 14ab

(±)- (1*R*,2*R*,3*S*,3a*R*,8b*S*)-3a-(4-chlorophenyl)-1,8b-dihydroxy-*N*,6,8-trimethoxy-3-phenyl-2,3,3a,8b-tetrahydro-1*H*-cyclopenta[*b*]benzofuran-2-carboxamide (14ab)

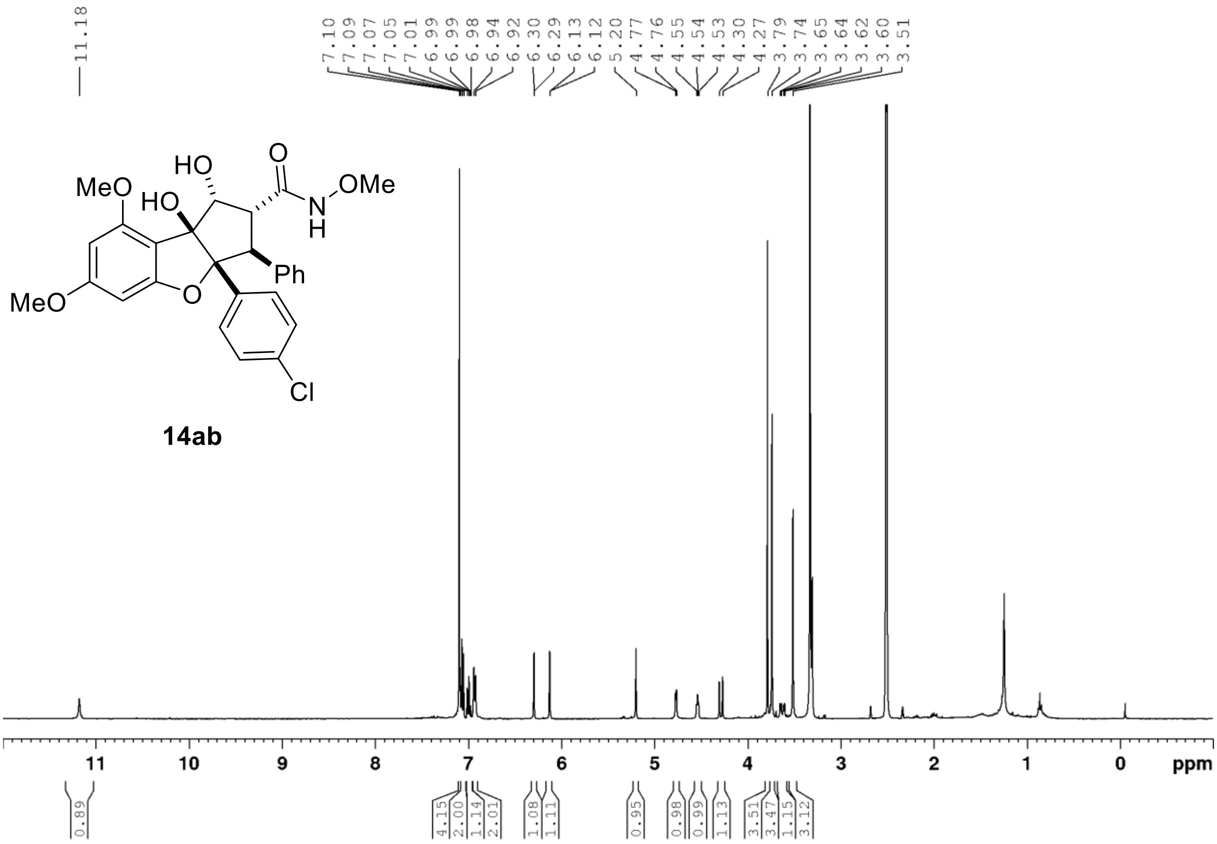

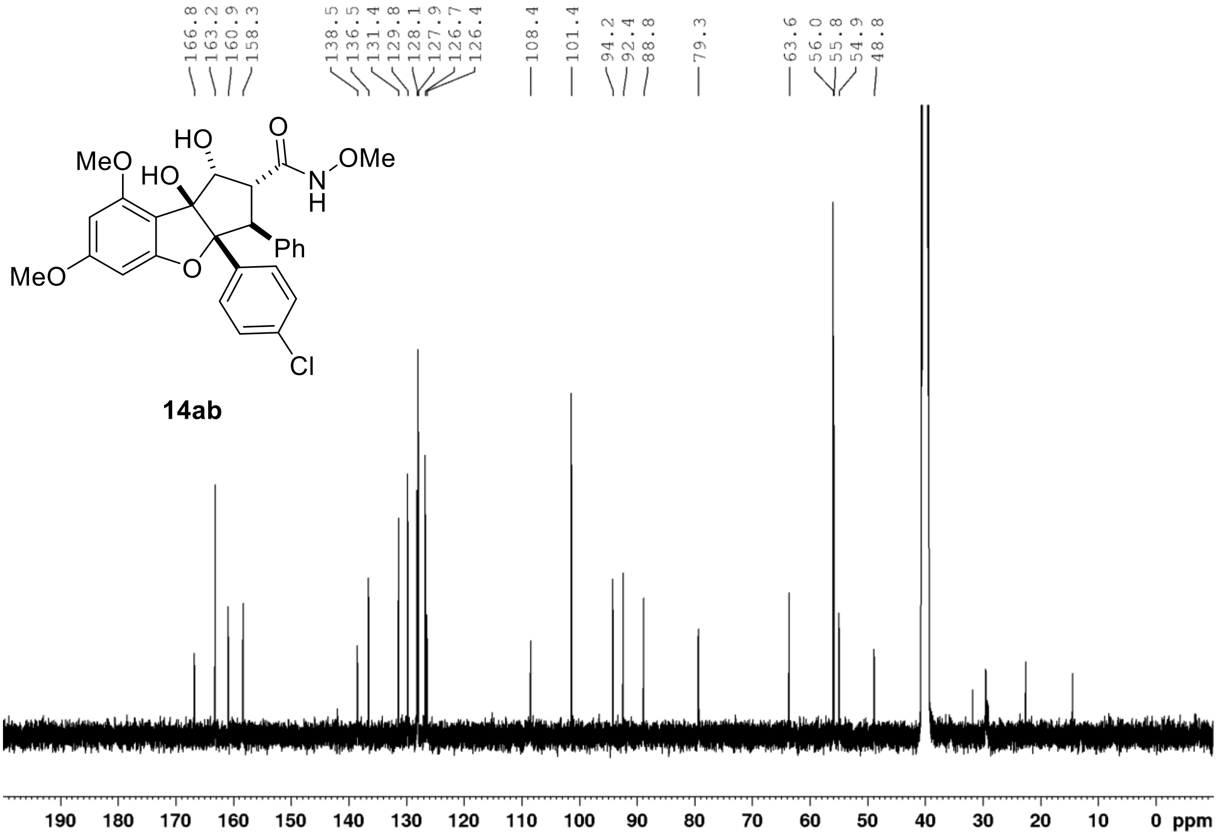

4.22. NMR spectroscopic data for the synthesis of 14baa

(±)- (1*R*,2*R*,3*S*,3a*R*,8b*S*)-3a-(4-Fluorophenyl)-1,8b-dihydroxy-6,8-dimethoxy-*N*,*N*-dimethyl-3-phenyl-2,3,3a,8b-tetrahydro-1*H*-cyclopenta[*b*]benzofuran-2-carboxamide (14baa)

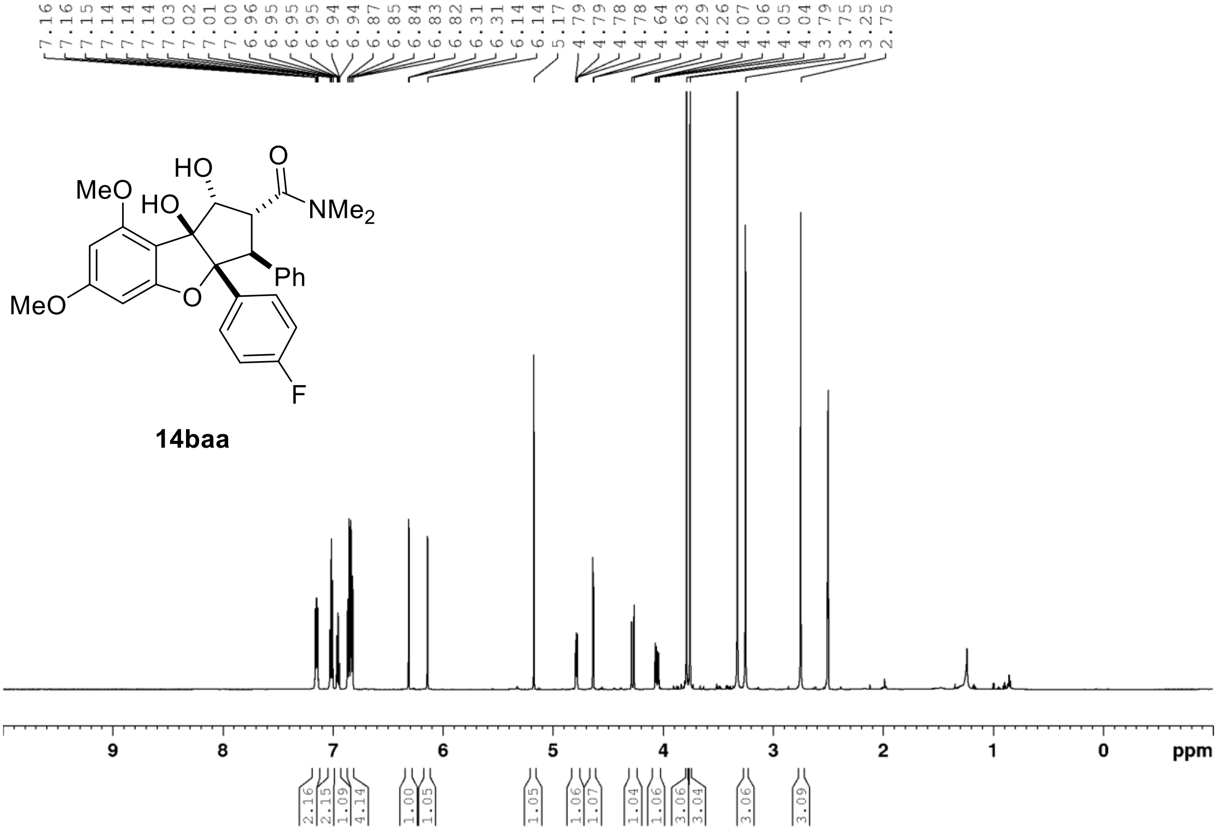

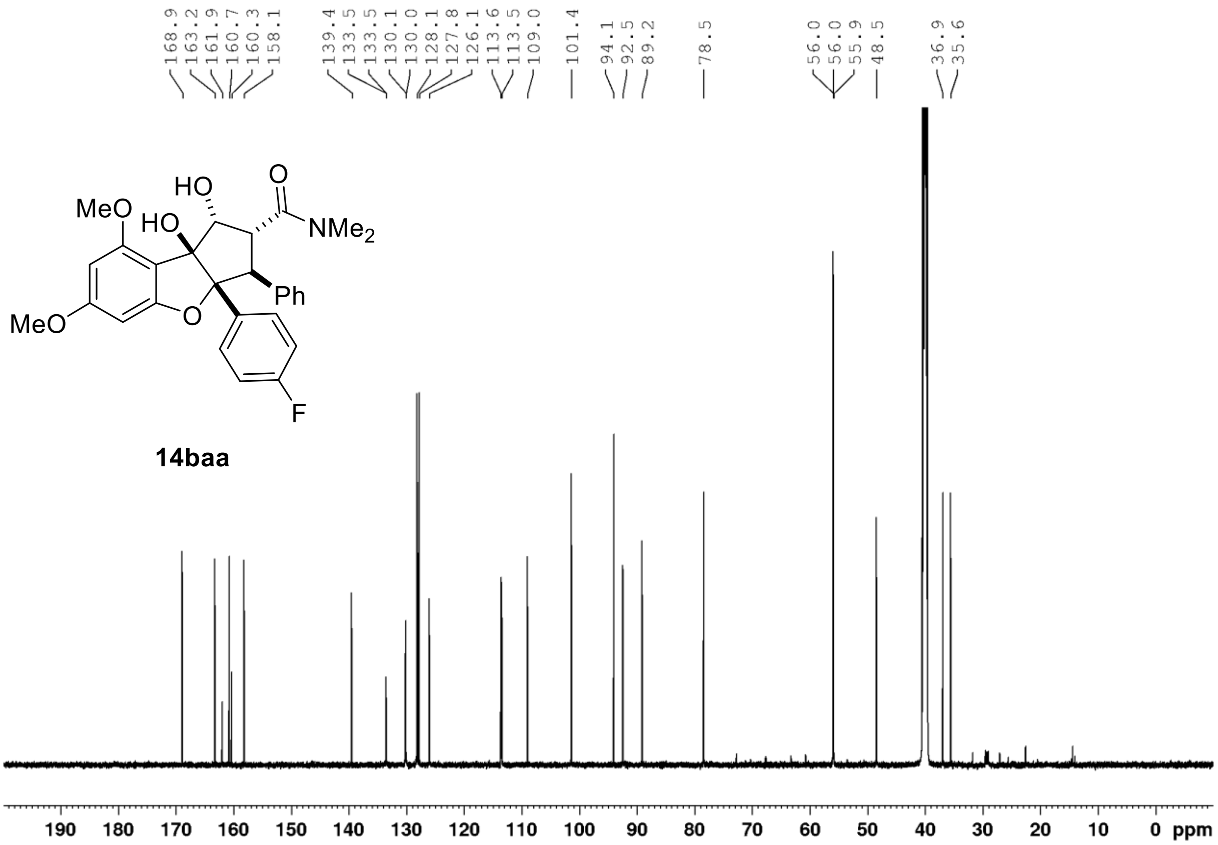

4.23. NMR spectroscopic data for the synthesis of 14bab

(±)- (1*R*,2*R*,3*S*,3a*R*,8b*S*)-3a-(4-Fluorophenyl)-1,8b-dihydroxy-*N*,6,8-trimethoxy-3-phenyl-2,3,3a,8b-tetrahydro-1*H*-cyclopenta[*b*]benzofuran-2-carboxamide (14bab)

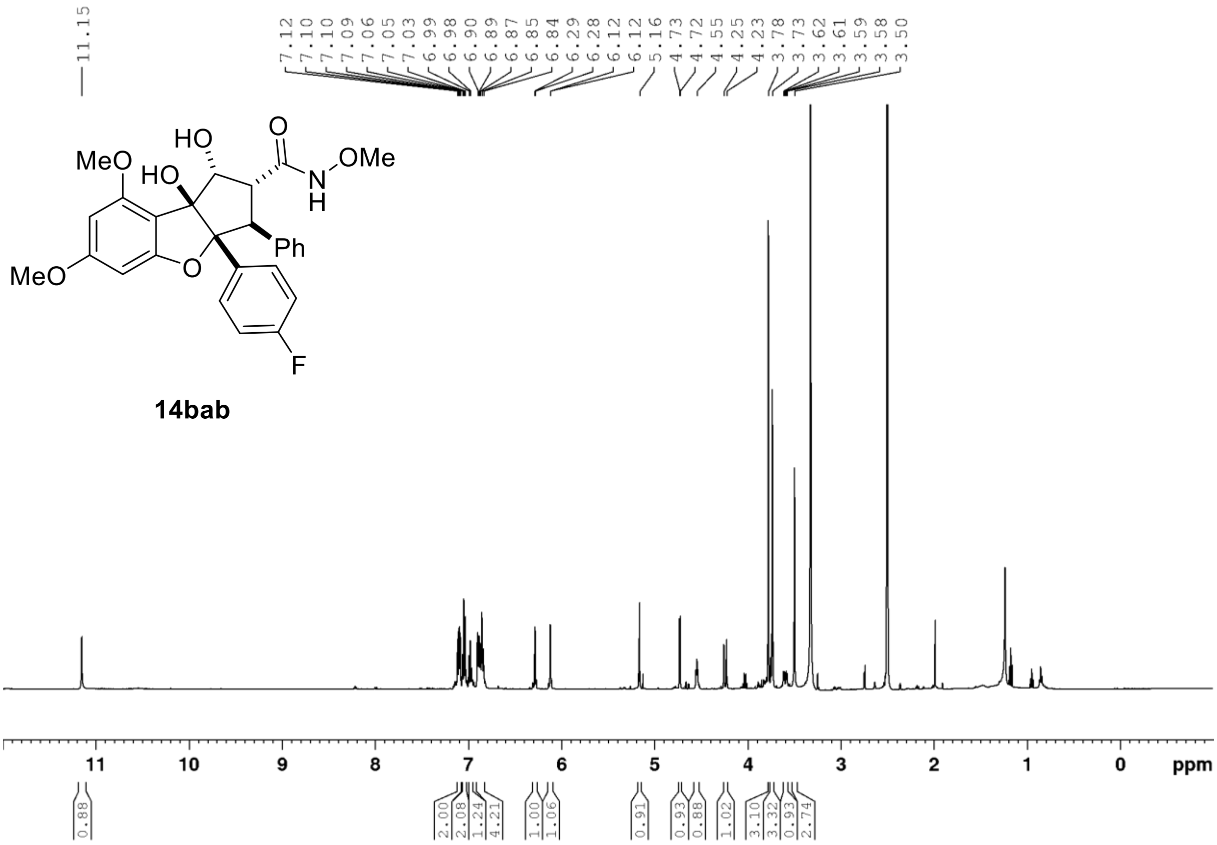

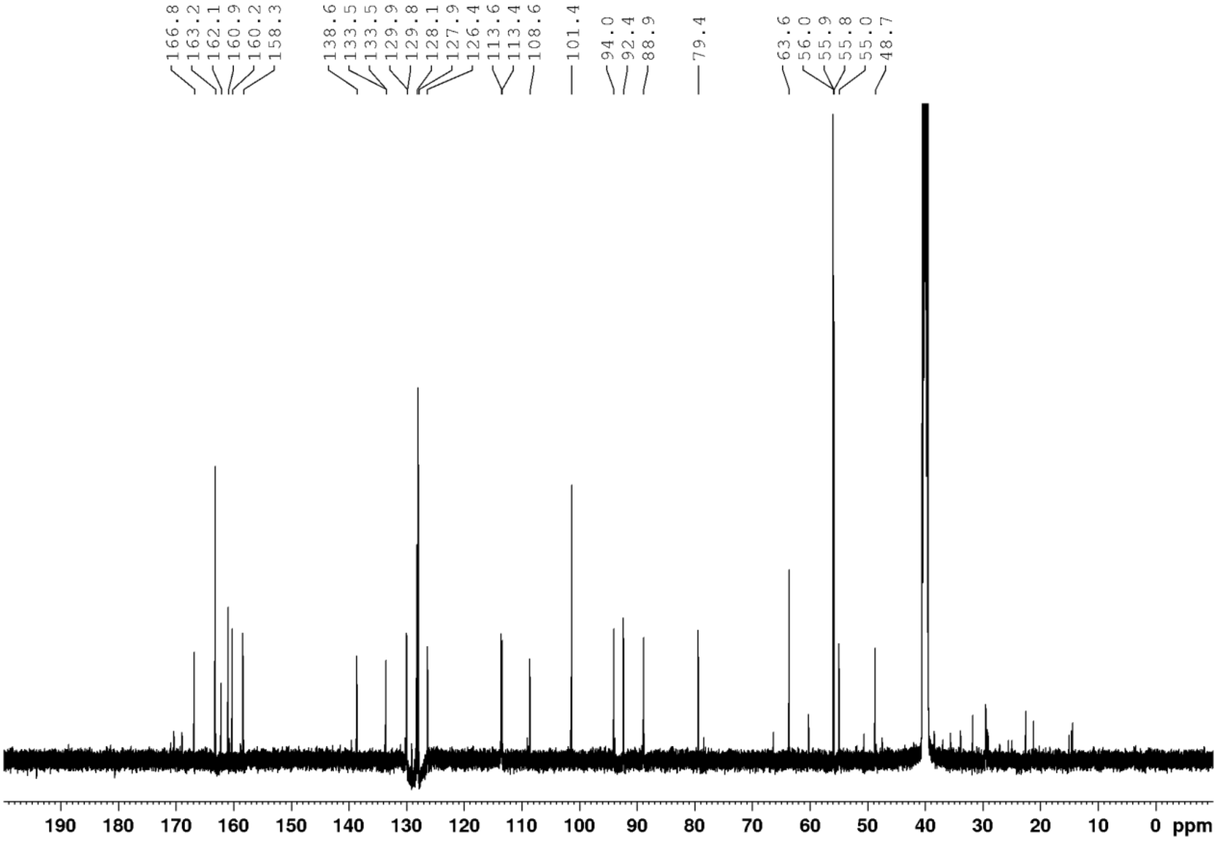

4.24. NMR spectroscopic data for the synthesis of *rac*-1b

(±)-(1*R*,2*R*,3*S*,3a*R*,8b*S*)-1,8b-Dihydroxy-6,8-dimethoxy-3a-(4-methoxyphenyl)-3-phenyl-2,3,3a,8b-tetrahydro-1*H*-cyclopenta[*b*]benzofuran-2-carboxylic acid (13bc)

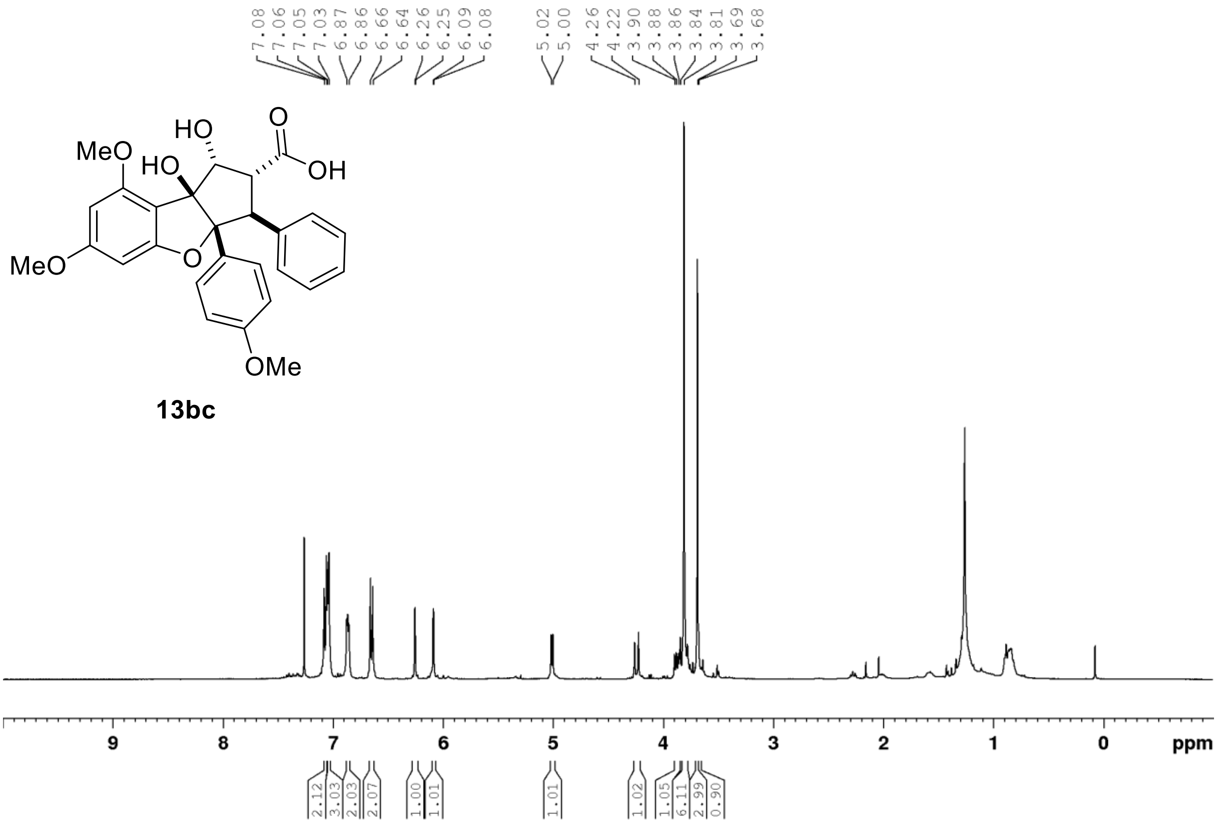

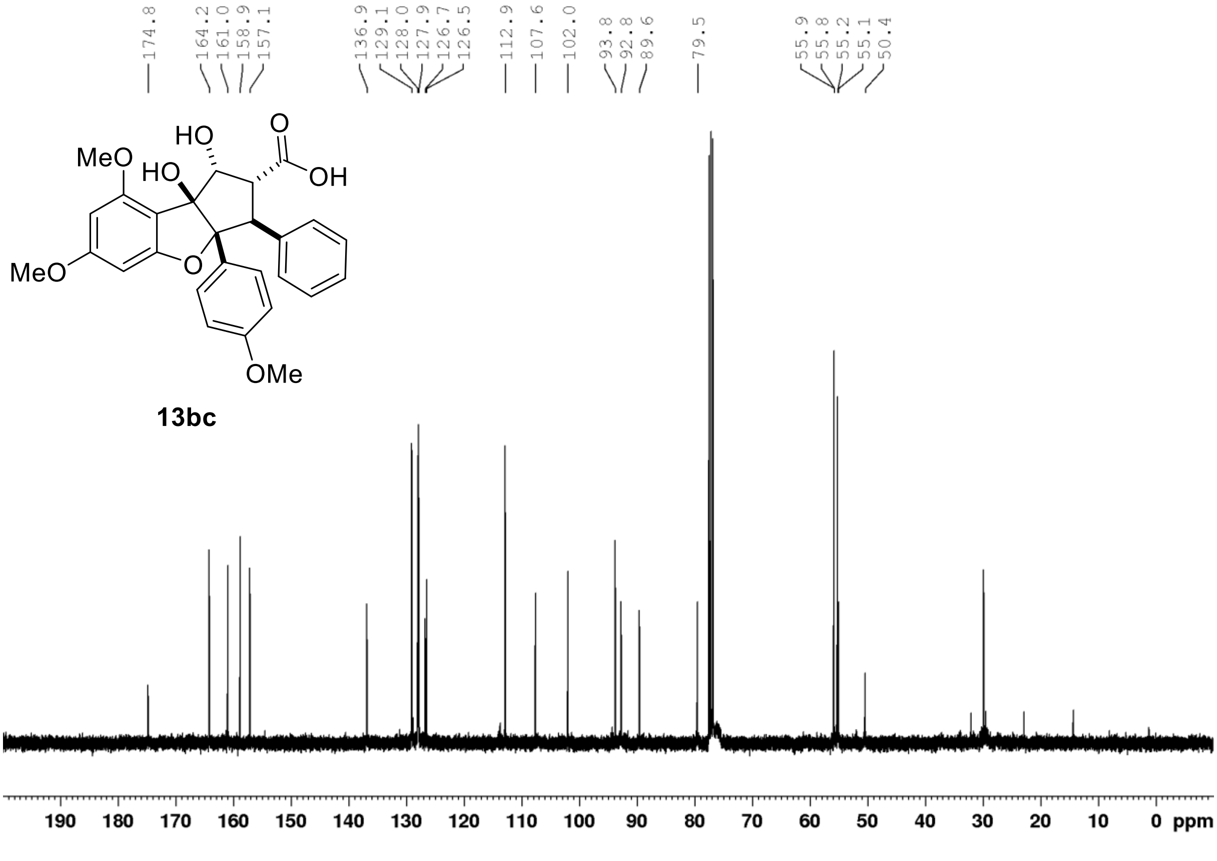

(±)-(1*R*,2*R*,3*S*,3a*R*,8b*S*)-1,8b-Dihydroxy-6,8-dimethoxy-3a-(4-methoxyphenyl)-*N*,*N*-dimethyl-3-phenyl-2,3,3a,8b-tetrahydro-1*H*-cyclopenta[*b*]benzofuran-2-carboxamide ((±)-rocaglamide, *rac*-1b)

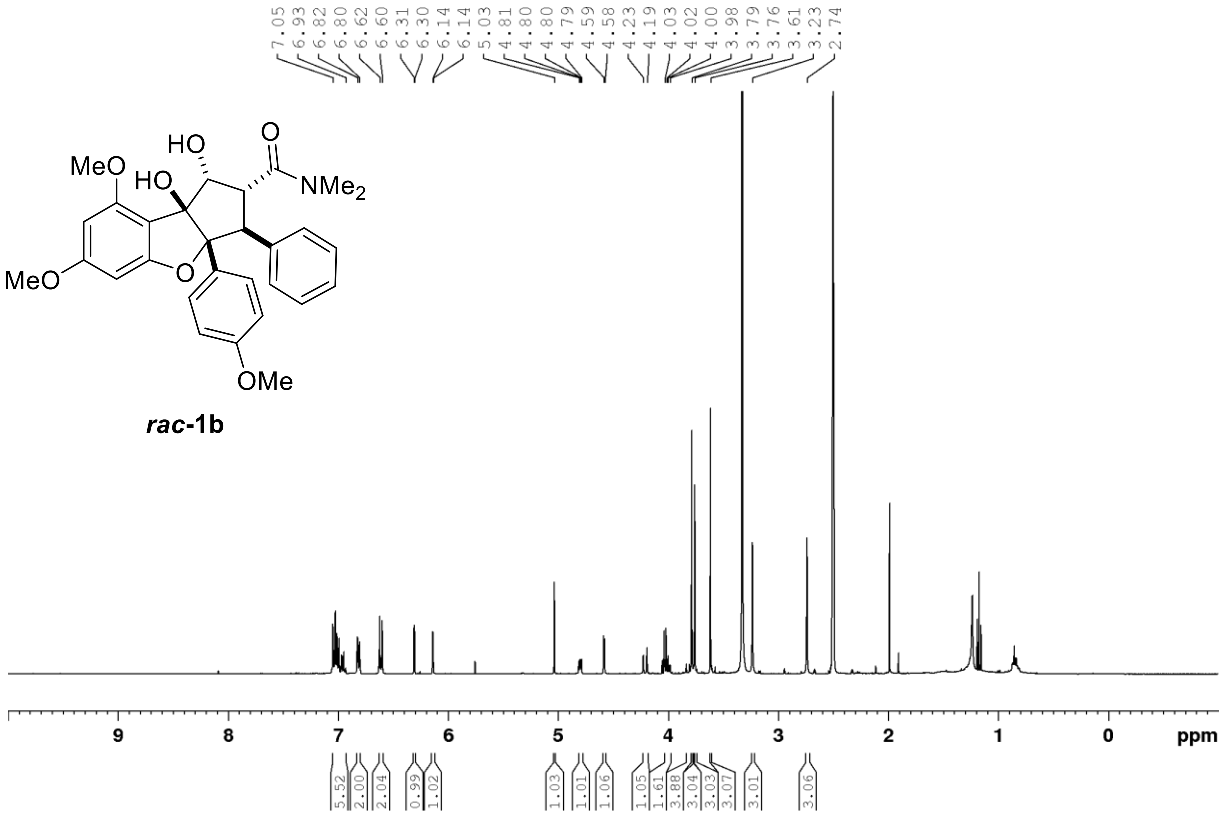

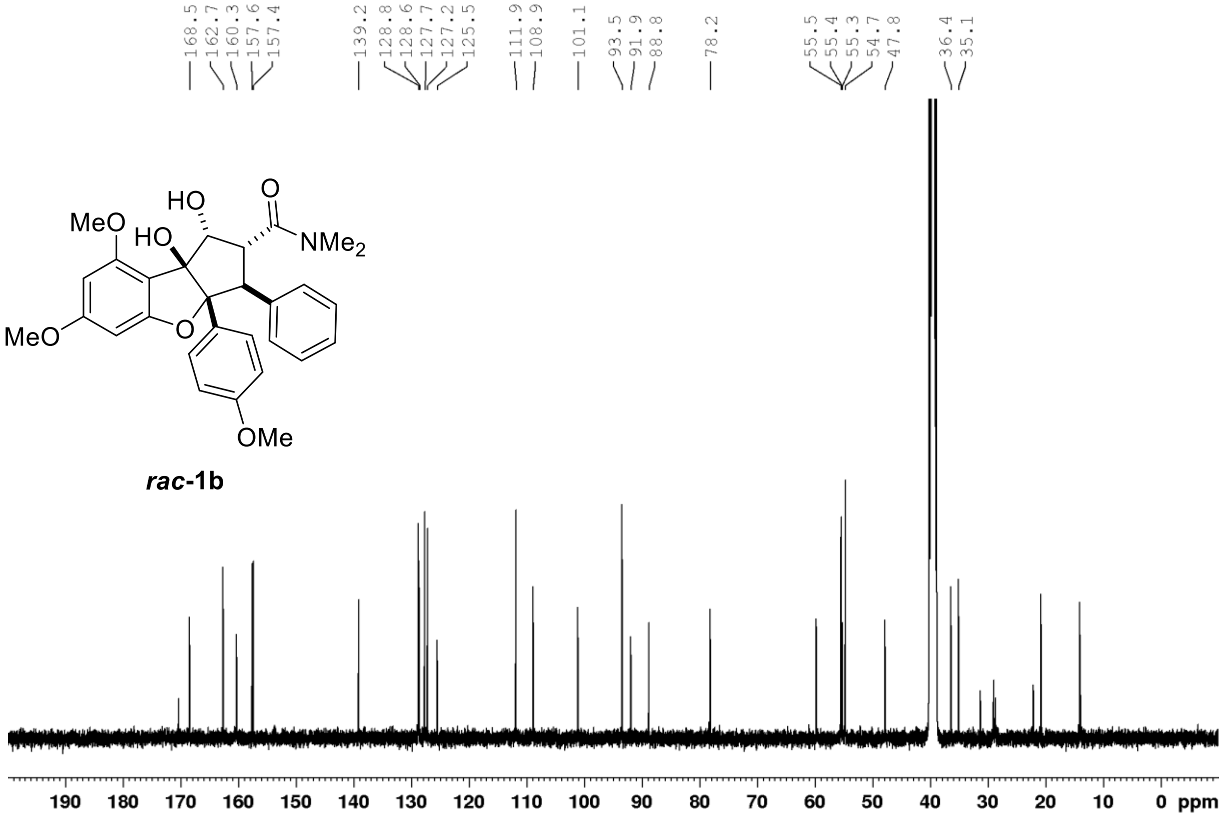

4.25. NMR spectroscopic data for the synthesis of *rac*-1c

(±)-(1*R*,2*R*,3*S*,3a*R*,8b*S*)-1,8b-Dihydroxy-*N*,6,8-trimethoxy-3a-(4-methoxyphenyl)-3-phenyl-2,3,3a,8b-tetrahydro-1*H*-cyclopenta[*b*]benzofuran-2-carboxamide ((±)-CR-31-B, *rac*-1c)

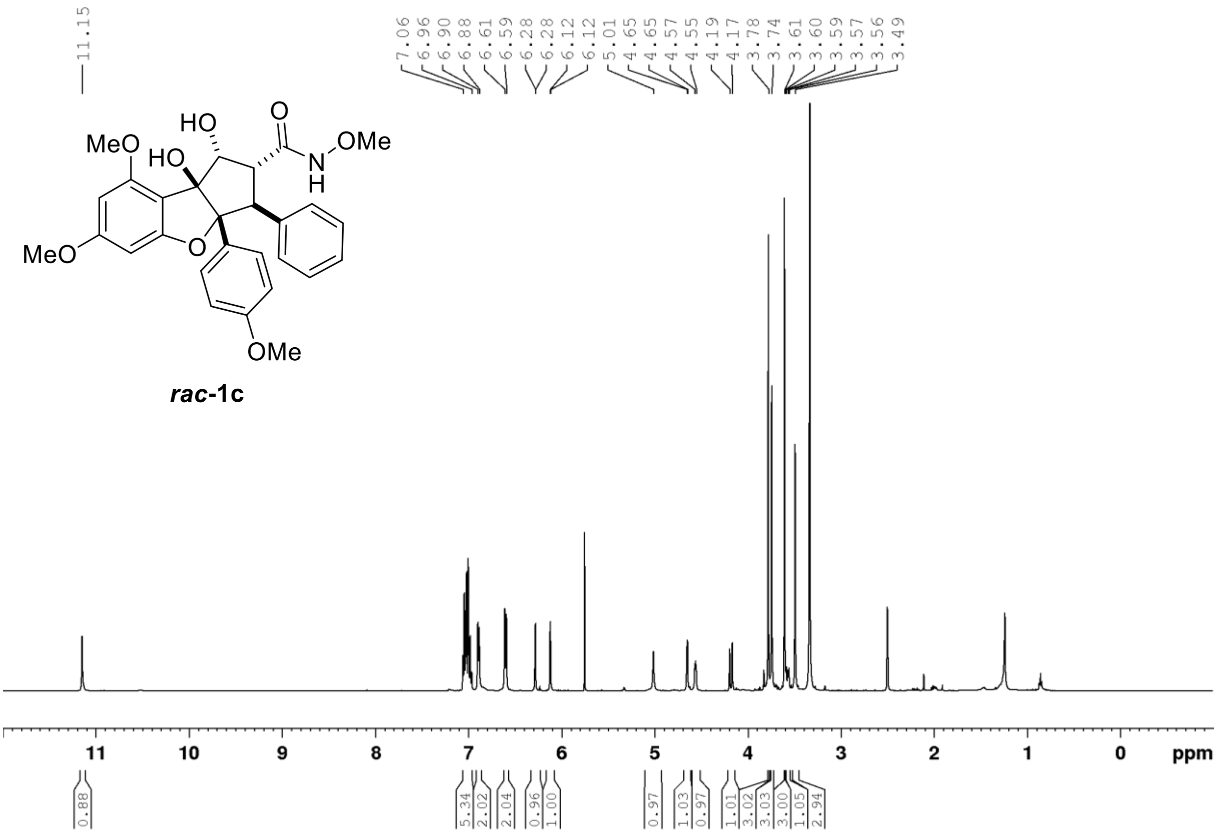

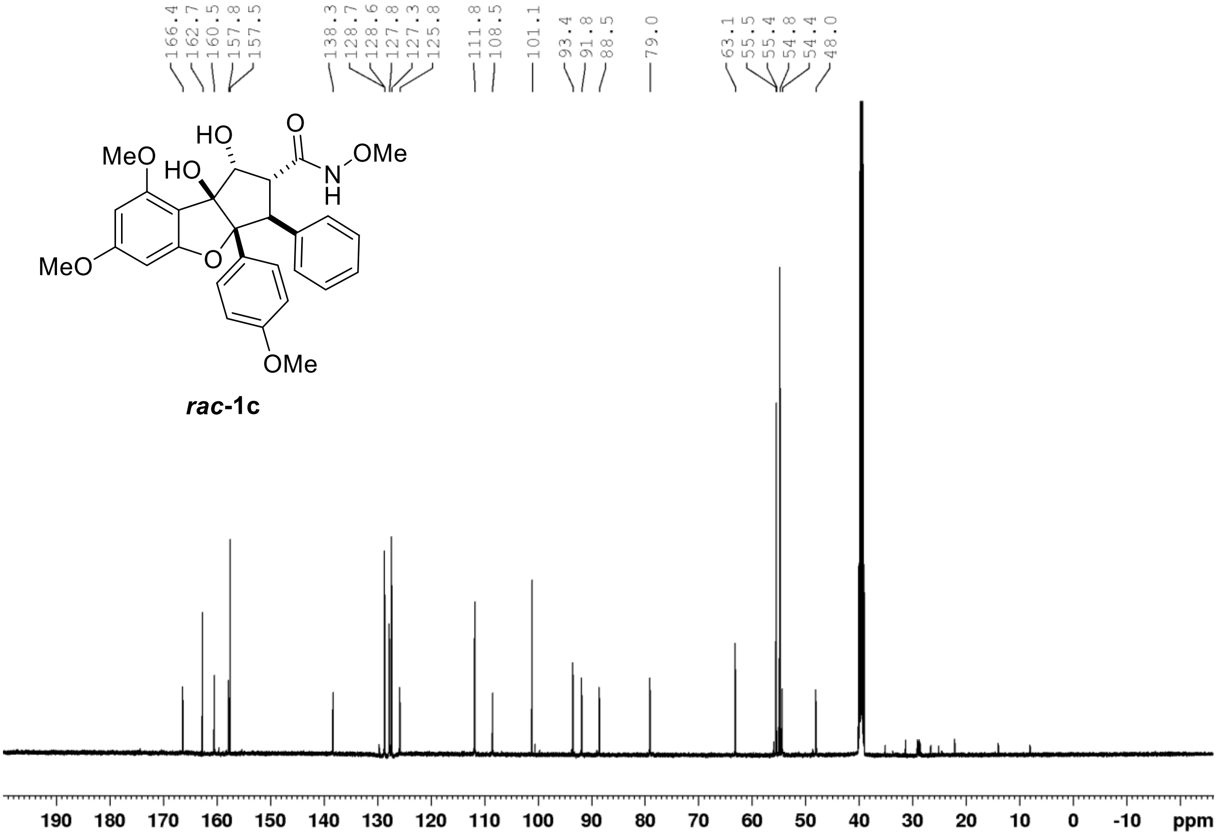

4.26. NMR spectroscopic data for the synthesis of 14da

(±)-(1*R*,2*R*,3*S*,3a*R*,8b*S*)-6,8-Dichloro-1,8b-dihydroxy-3a-(4-methoxyphenyl)-3-phenyl-2,3,3a,8b-tetrahydro-1*H*-cyclopenta[*b*]benzofuran-2-carboxylic acid (13da)

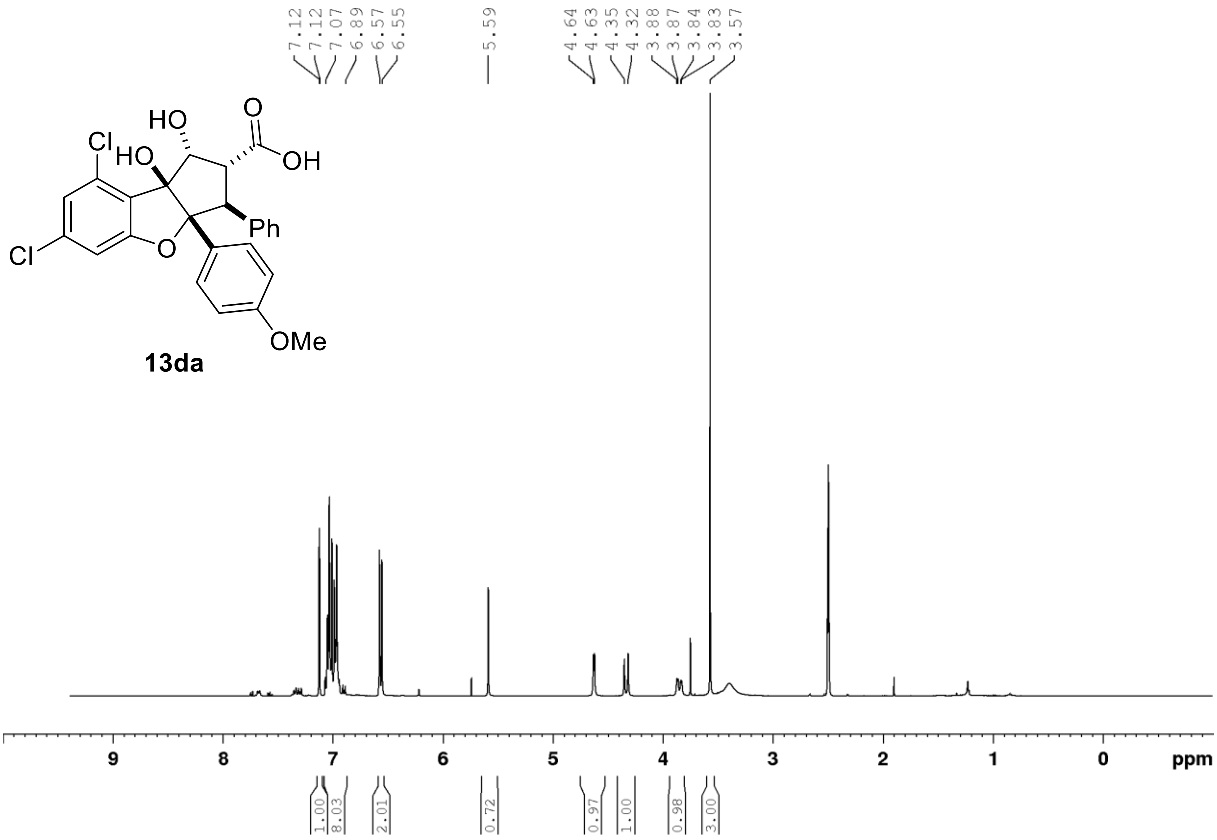

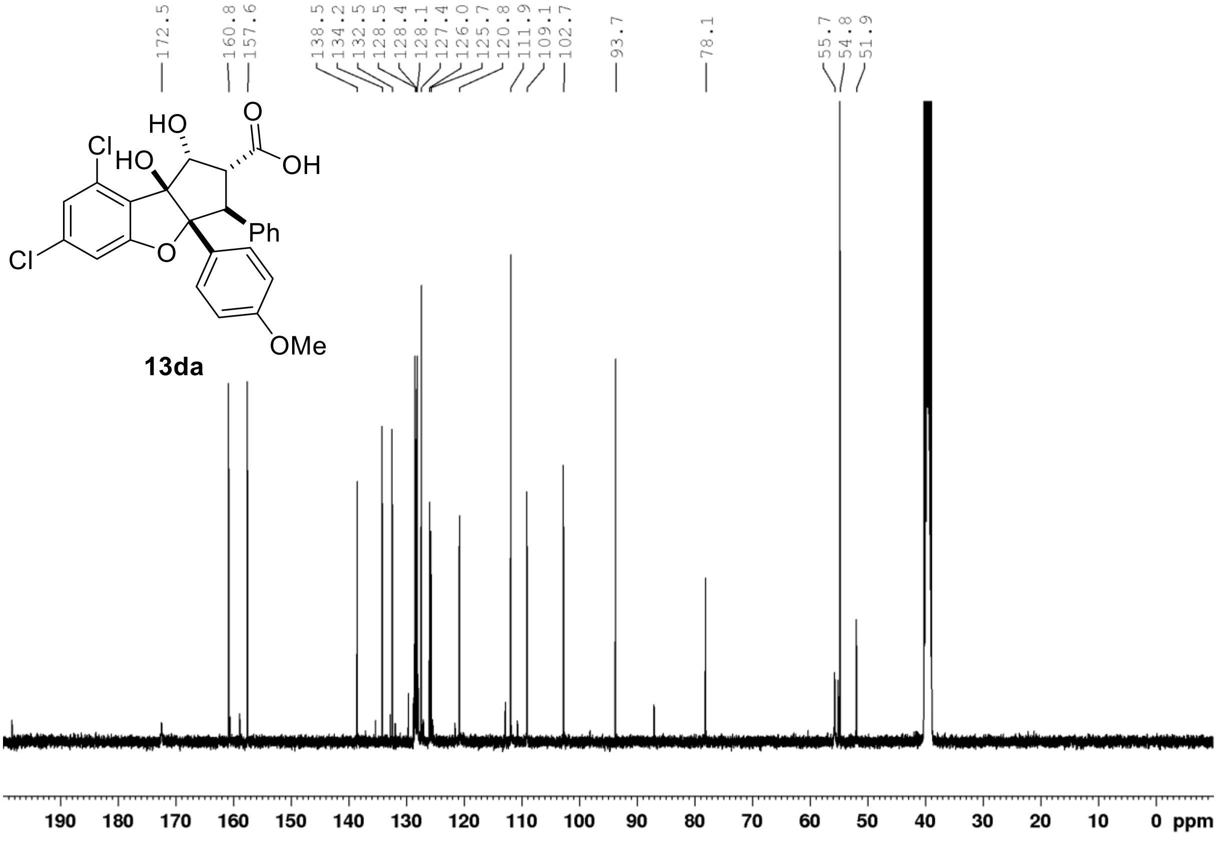

(±)-(1*R*,2*R*,3*S*,3a*R*,8b*S*)-6,8-Dichloro-1,8b-dihydroxy-*N*-methoxy-3a-(4-methoxyphenyl)-3-phenyl-2,3,3a,8b-tetrahydro-1*H*-cyclopenta[*b*]benzofuran-2-carboxamide (14da)

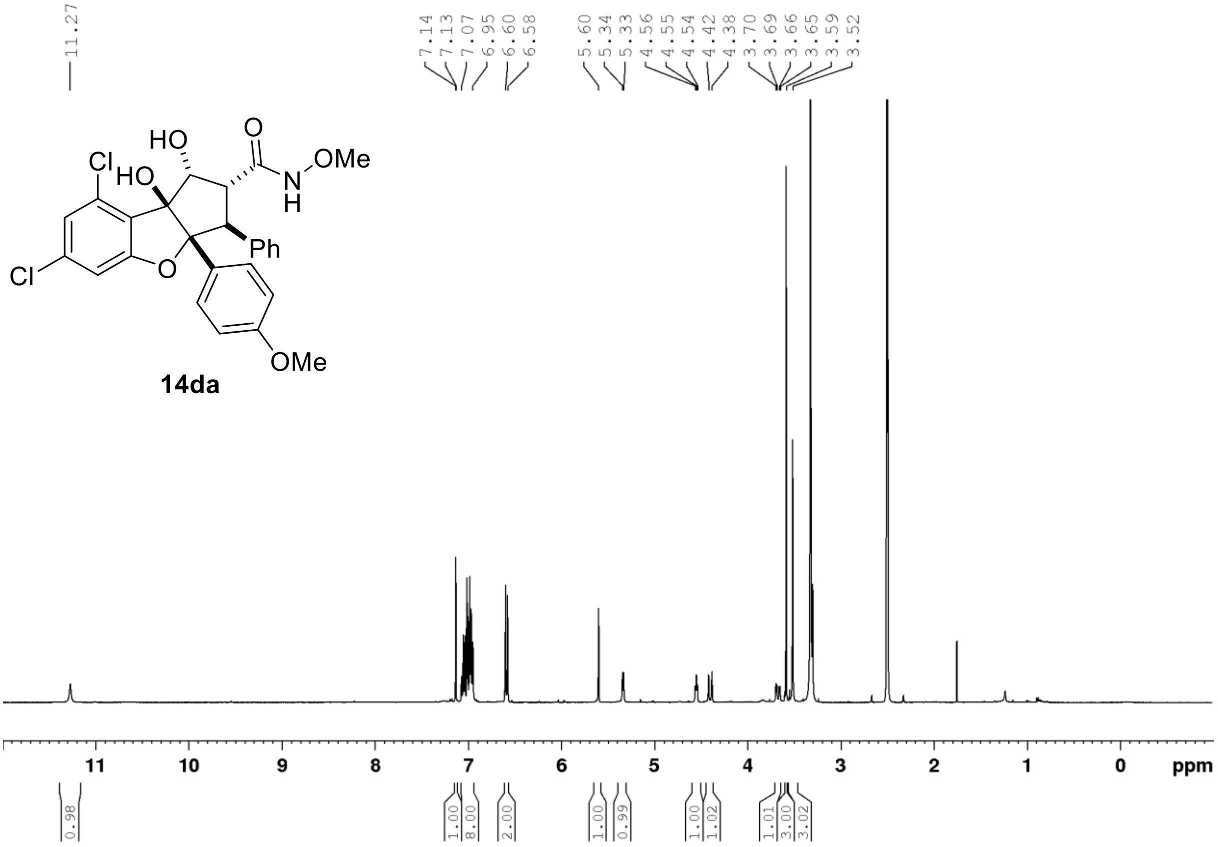

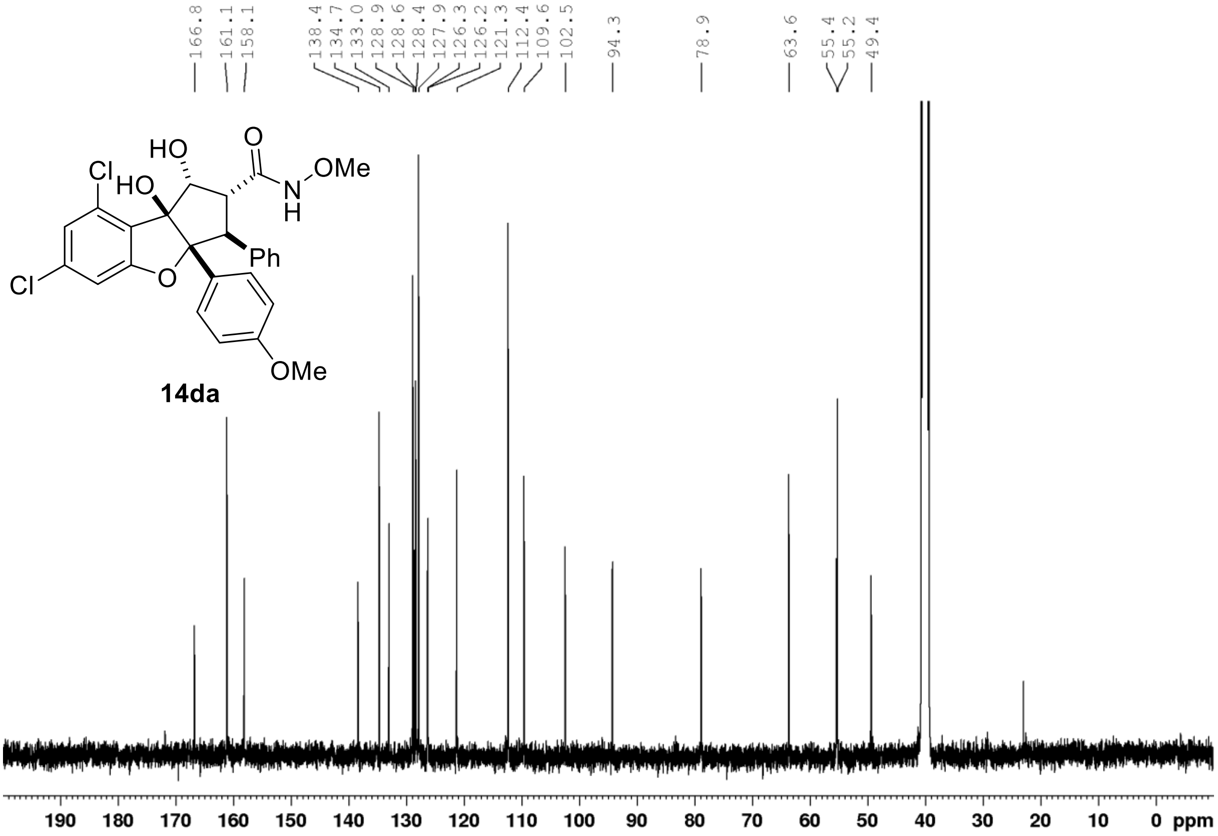

4.27. NMR spectroscopic data for the synthesis of 14f

(±)-(1*R*,2*R*,3*S*,3a*R*,8b*S*)-6-Bromo-8-chloro-1,8b-dihydroxy-3a-(4-methoxyphenyl)-3-phenyl-2,3,3a,8b-tetrahydro-1*H*-cyclopenta[*b*]benzofuran-2-carboxylic acid (13f)

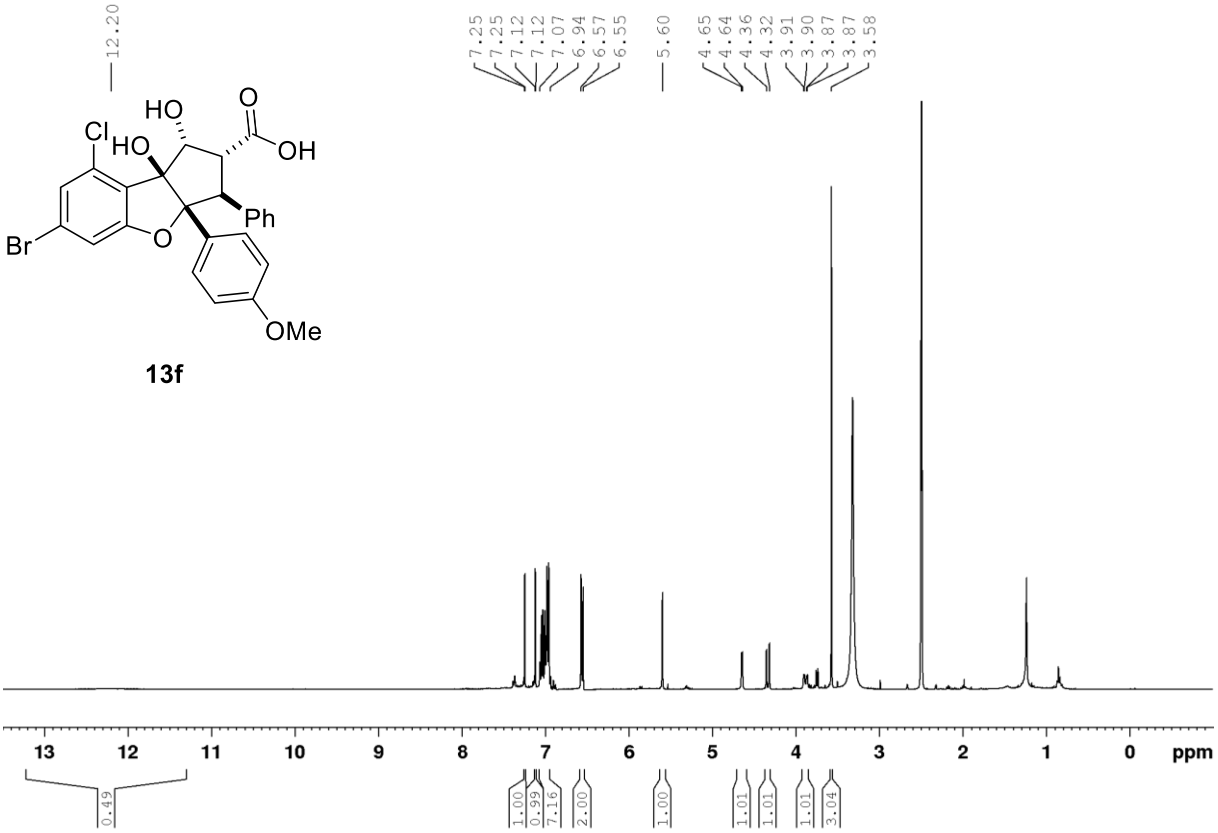

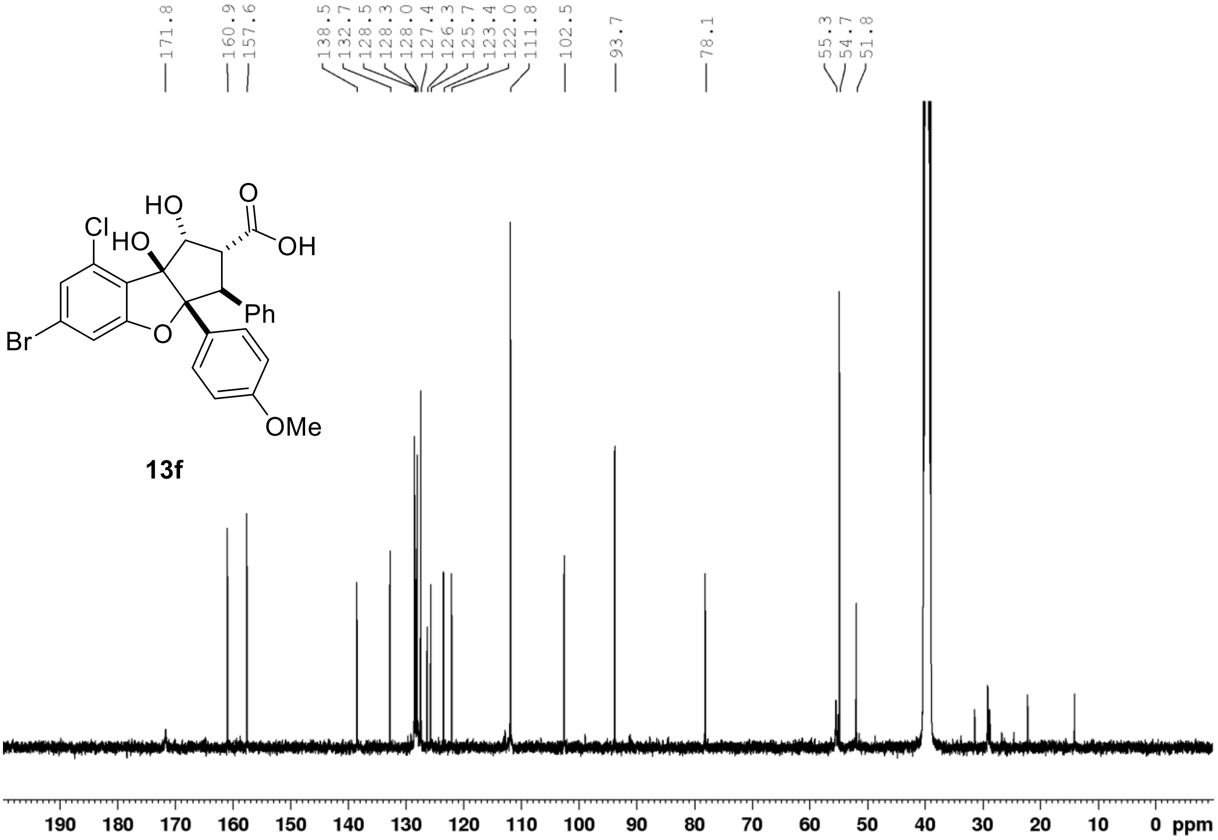

(±)-(1*R*,2*R*,3*S*,3a*R*,8b*S*)-6-Bromo-8-chloro-1,8b-dihydroxy-*N*-methoxy-3a-(4-methoxyphenyl)-3-phenyl-2,3,3a,8b-tetrahydro-1*H*-cyclopenta[*b*]benzofuran-2-carboxamide (14f)

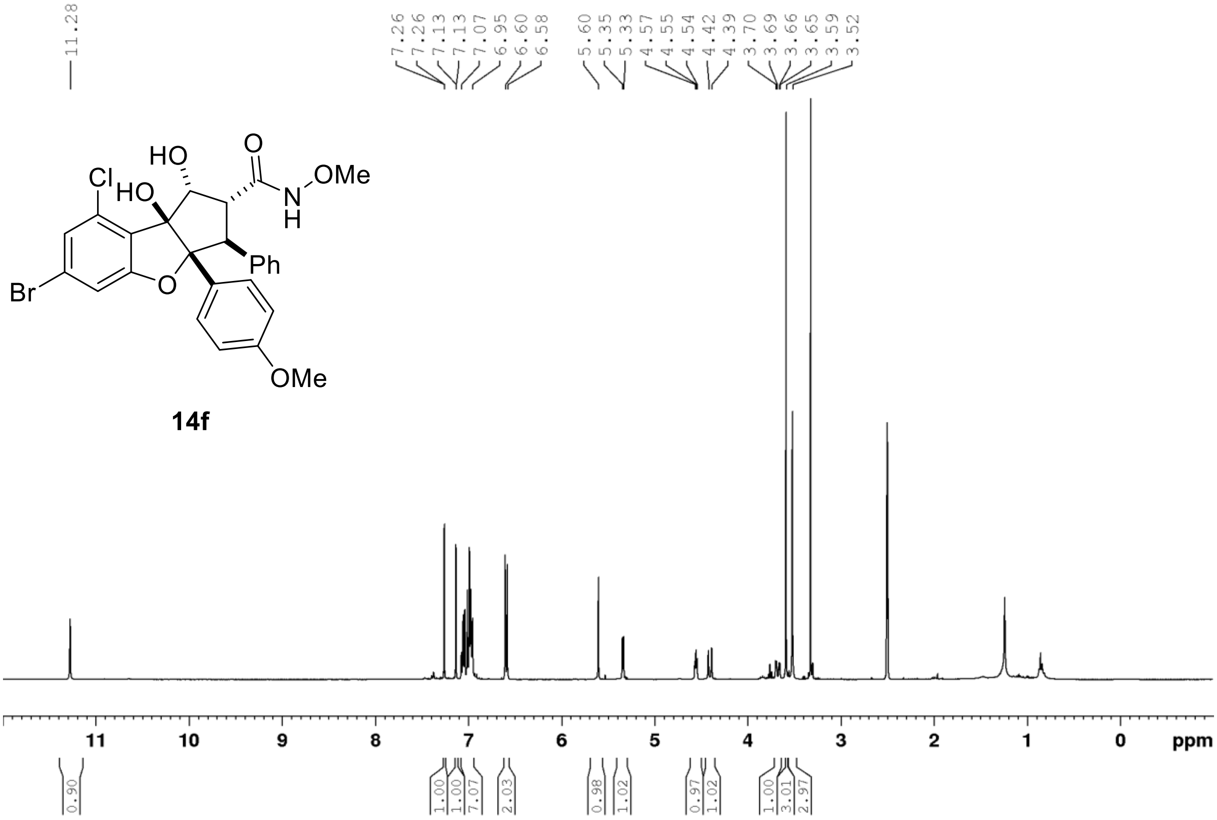

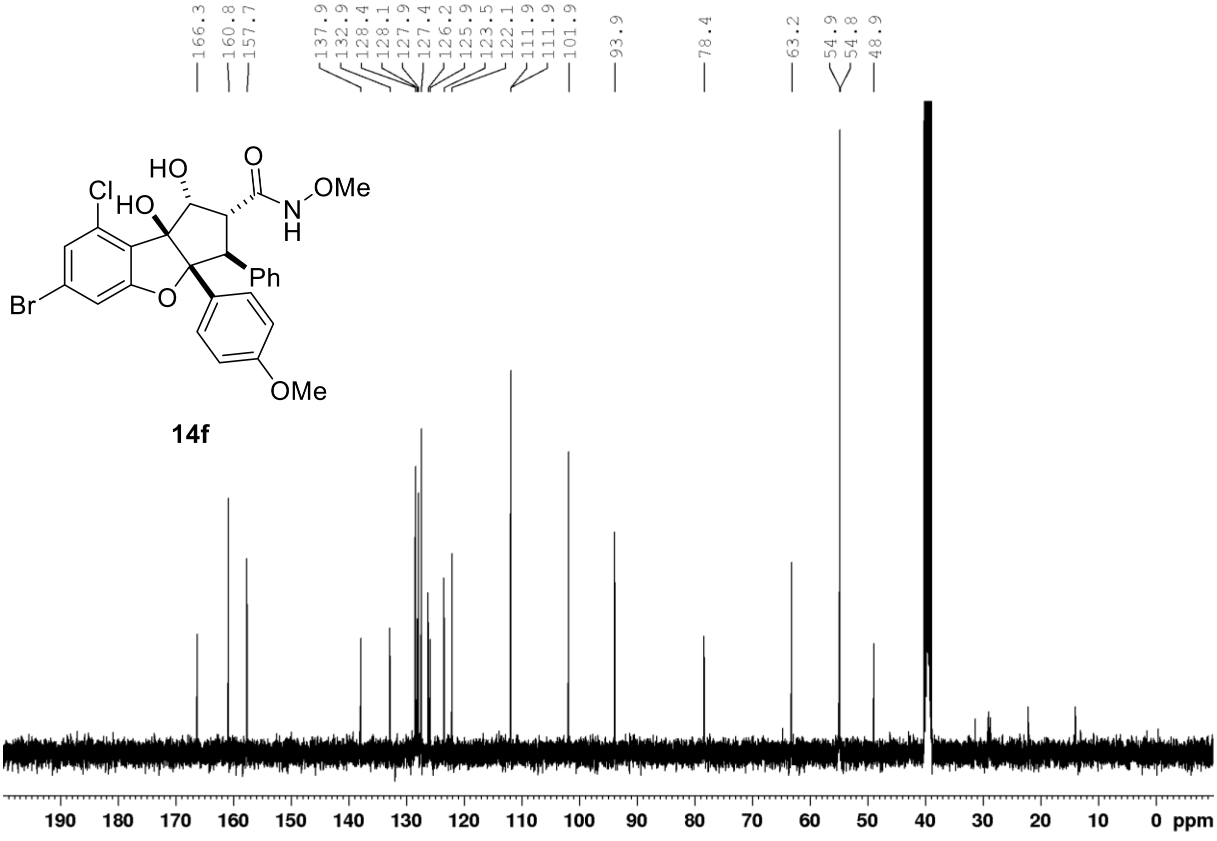

4.28. NMR spectroscopic data for the synthesis of 14g

(±)-(1*R*,2*R*,3*S*,3a*R*,8b*S*)-6-Bromo-8-chloro-1,8b-dihydroxy-3a-(4-methoxyphenyl)-3-phenyl-2,3,3a,8b-tetrahydro-1*H*-cyclopenta[*b*]benzofuran-2-carboxylic acid (13g)

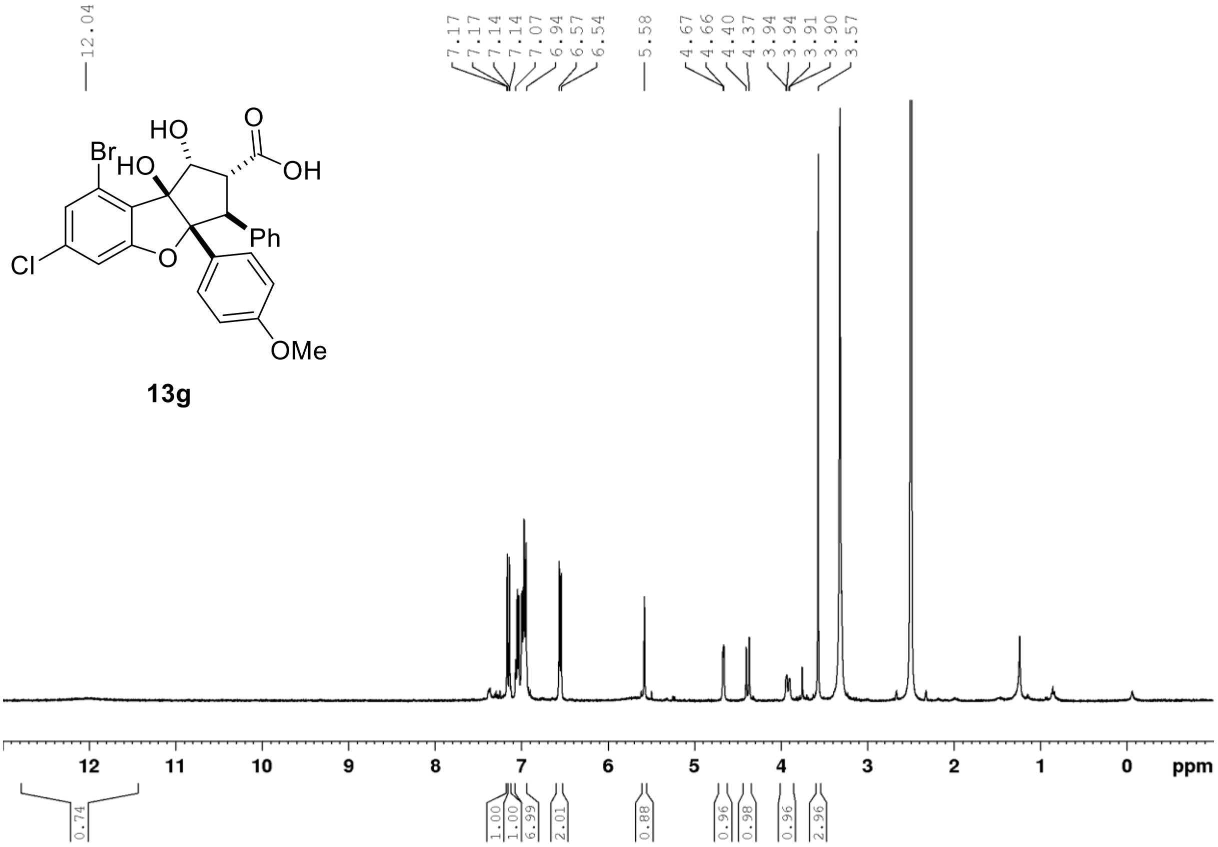

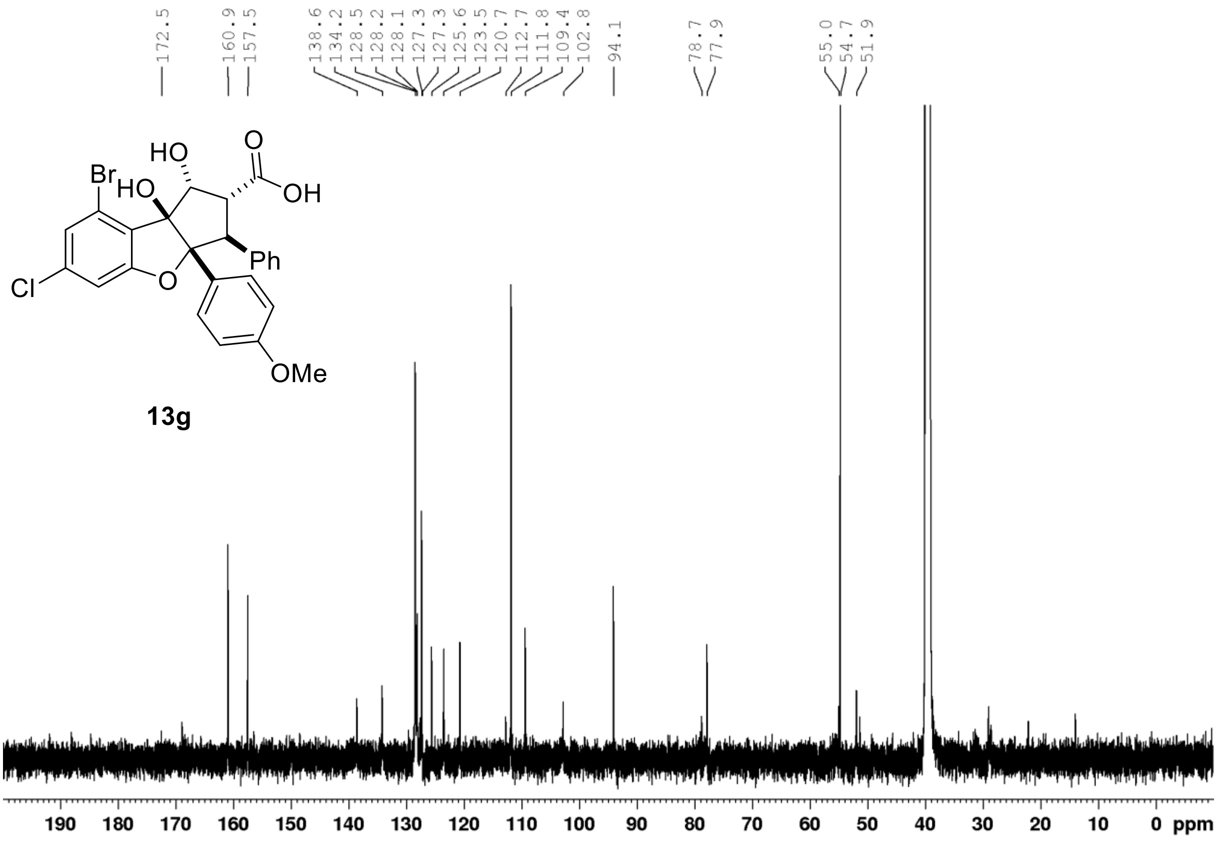

(±)-(1*R*,2*R*,3*S*,3a*R*,8b*S*)-8-Bromo-6-chloro-1,8b-dihydroxy-*N*-methoxy-3a-(4-methoxyphenyl)-3-phenyl-2,3,3a,8b-tetrahydro-1*H*-cyclopenta[*b*]benzofuran-2-carboxamide (14g)

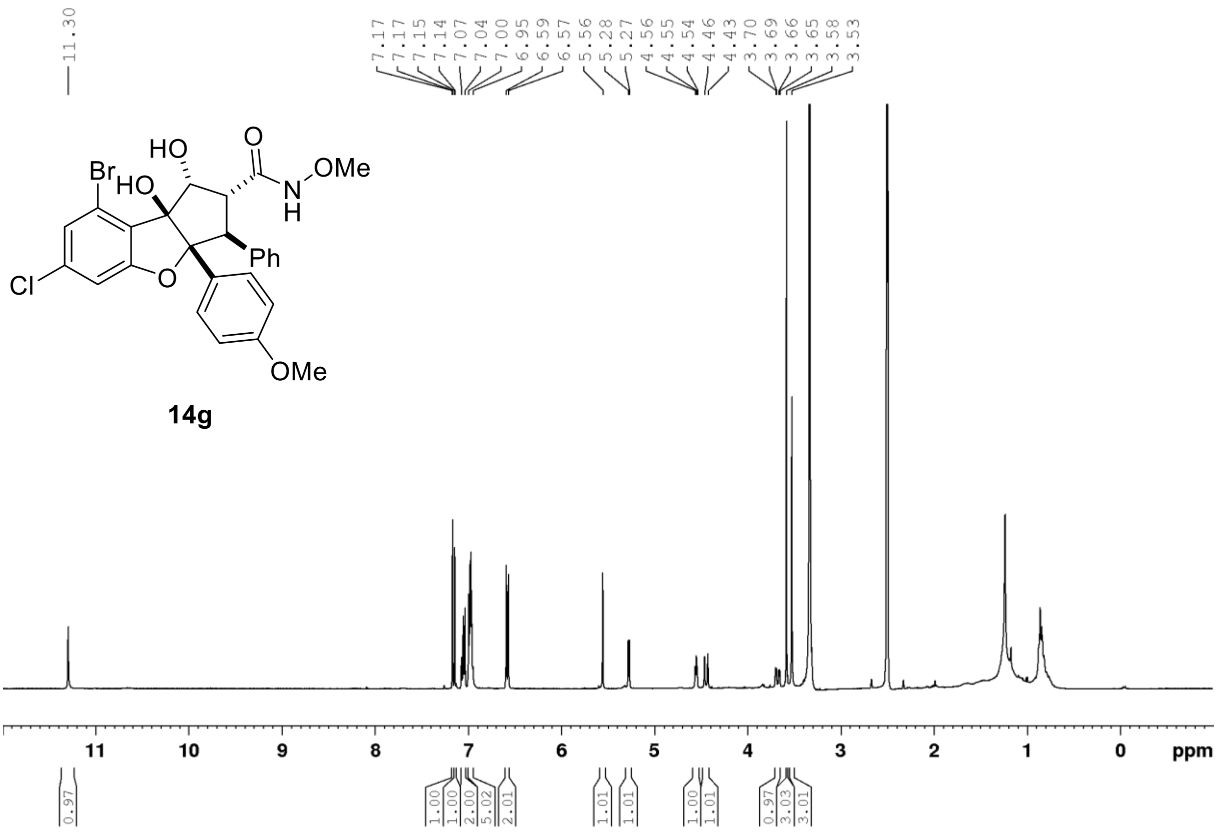

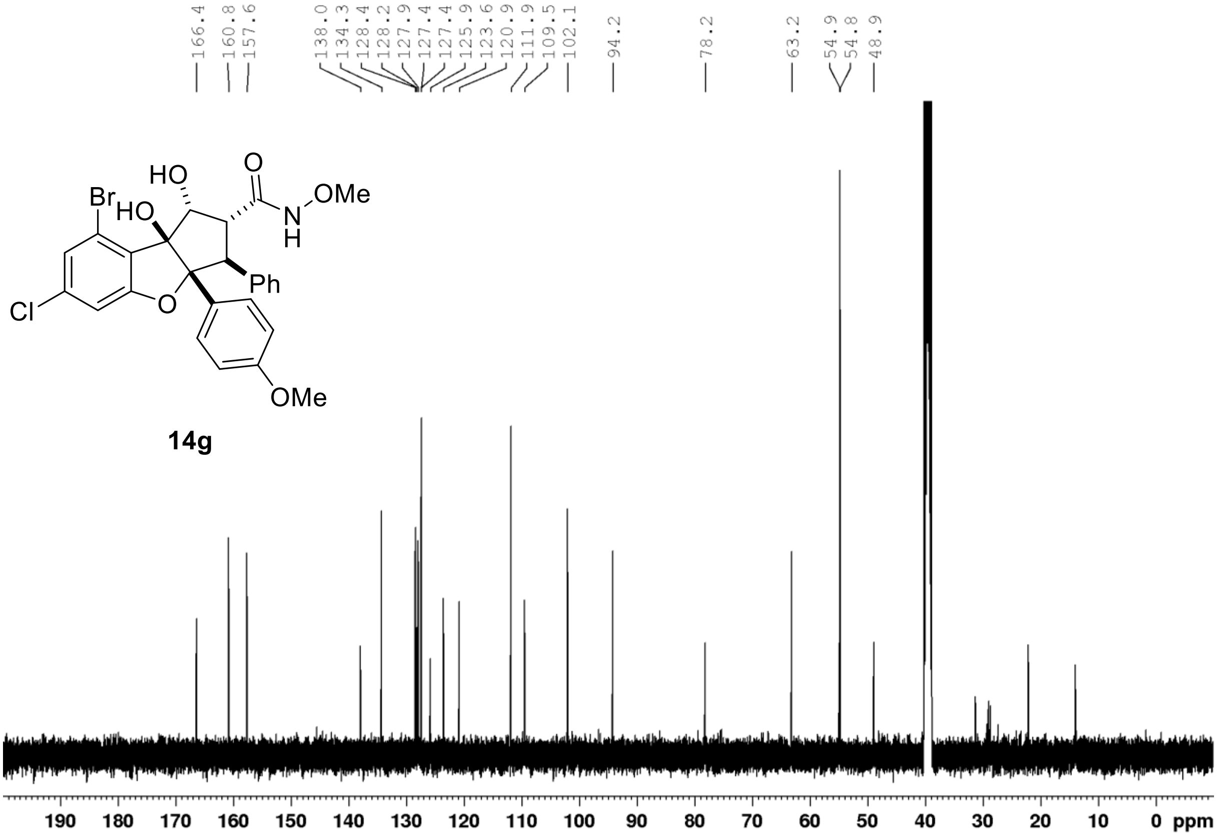

4.29. NMR spectroscopic data for the synthesis of 14ha

(±)- (1*R*,2*R*,3*S*,3a*R*,8b*S*)-8-Fluoro-1,8b-dihydroxy-6-methoxy-3a-(4-methoxyphenyl)-*N*,*N*-dimethyl-3-phenyl-2,3,3a,8b-tetrahydro-1*H*-cyclopenta[*b*]benzofuran-2-carboxamide (14ha)

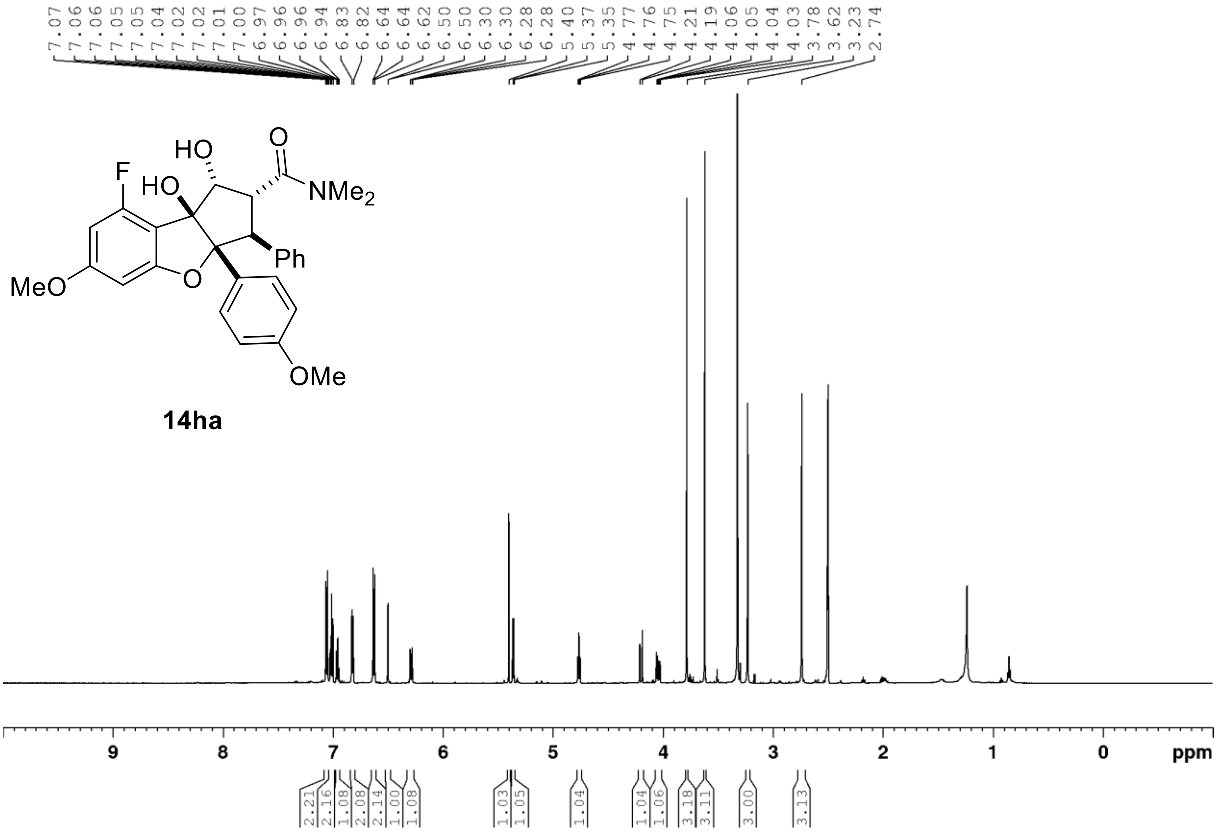

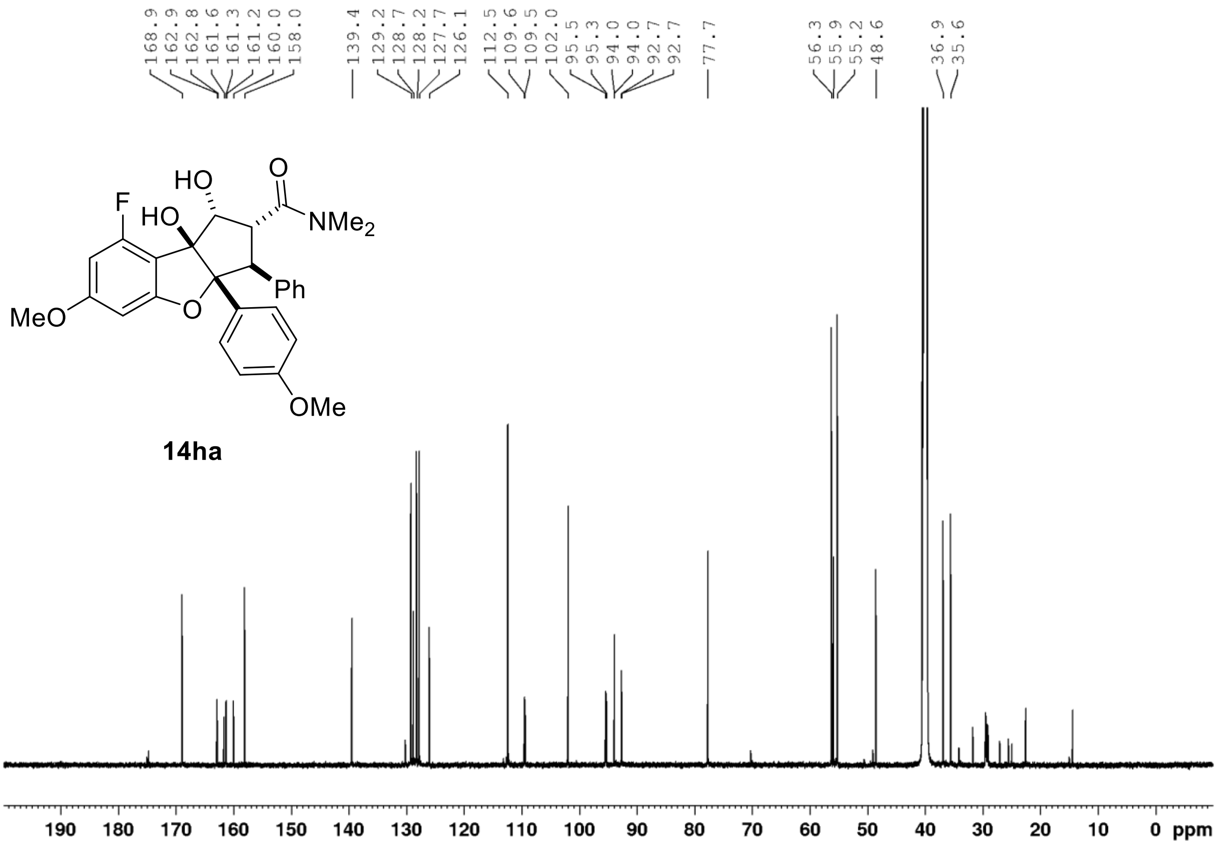

4.30. NMR spectroscopic data for the synthesis of 14hb

(±)- (1*R*,2*R*,3*S*,3a*R*,8b*S*)-8-Fluoro-1,8b-dihydroxy-*N*,6-dimethoxy-3a-(4-methoxyphenyl)-3-phenyl-2,3,3a,8b-tetrahydro-1*H*-cyclopenta[*b*]benzofuran-2-carboxamide (14hb)

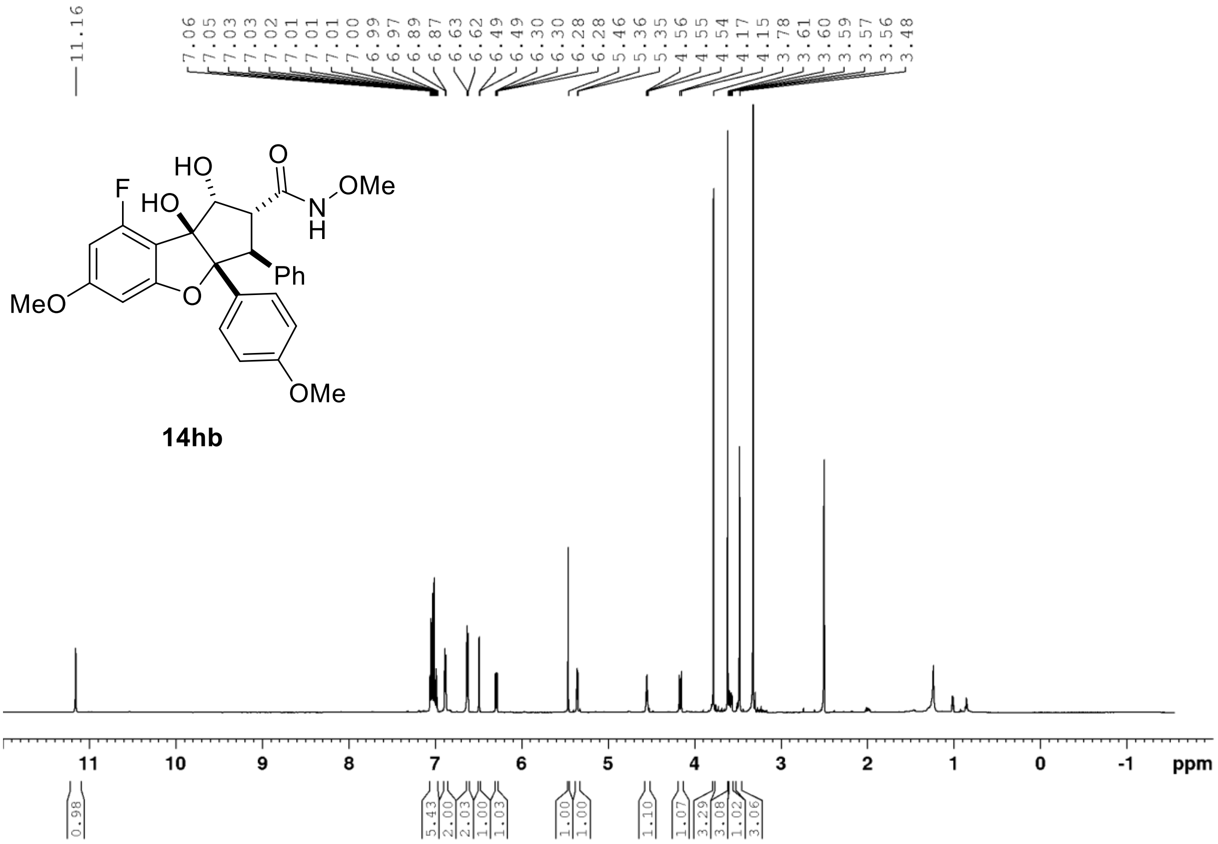

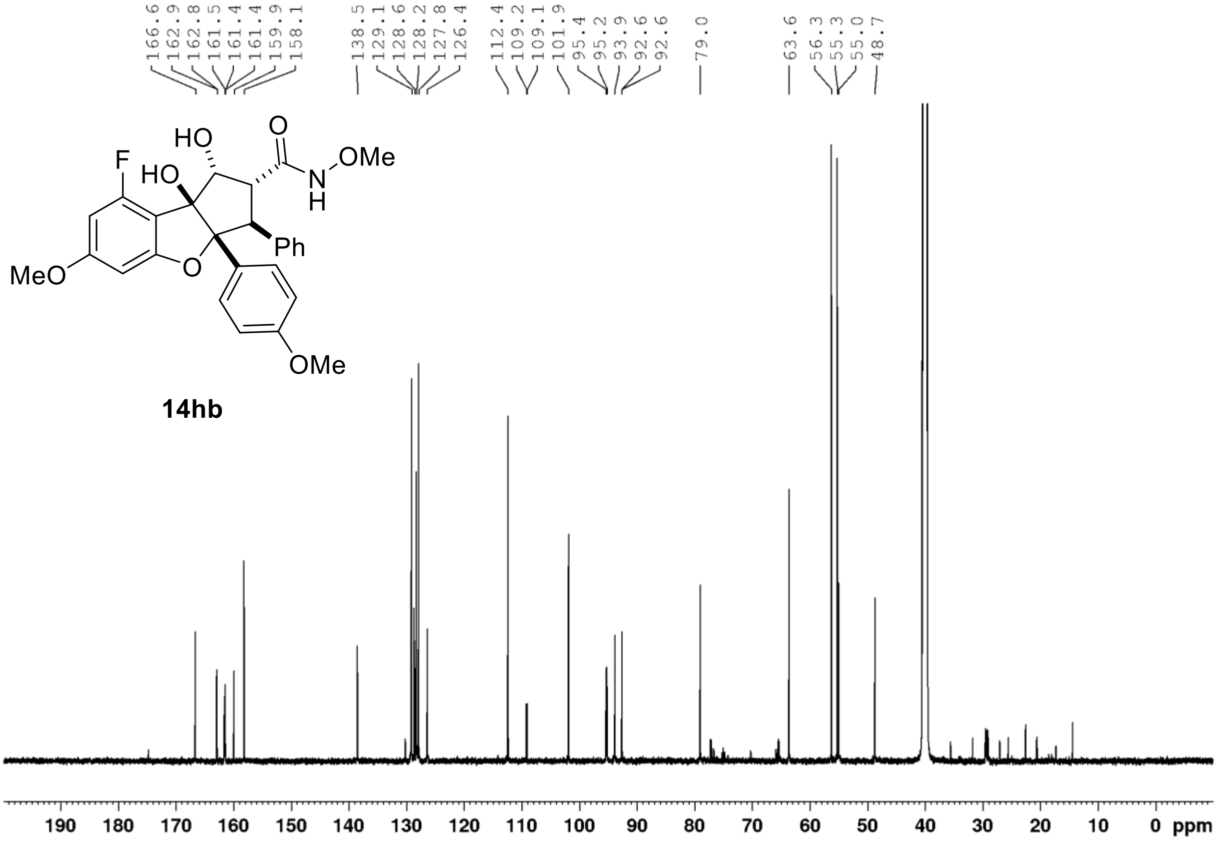

4.31. NMR spectroscopic data for the synthesis of 14m

(±)- (1*R*,2*R*,3*S*,3a*R*,8b*S*)-6-Bromo-1,8b-dihydroxy-*N*,8-dimethoxy-3a-(4-methoxyphenyl)-3-phenyl-2,3,3a,8b-tetrahydro-1*H*-cyclopenta[*b*]benzofuran-2-carboxamide (14m)

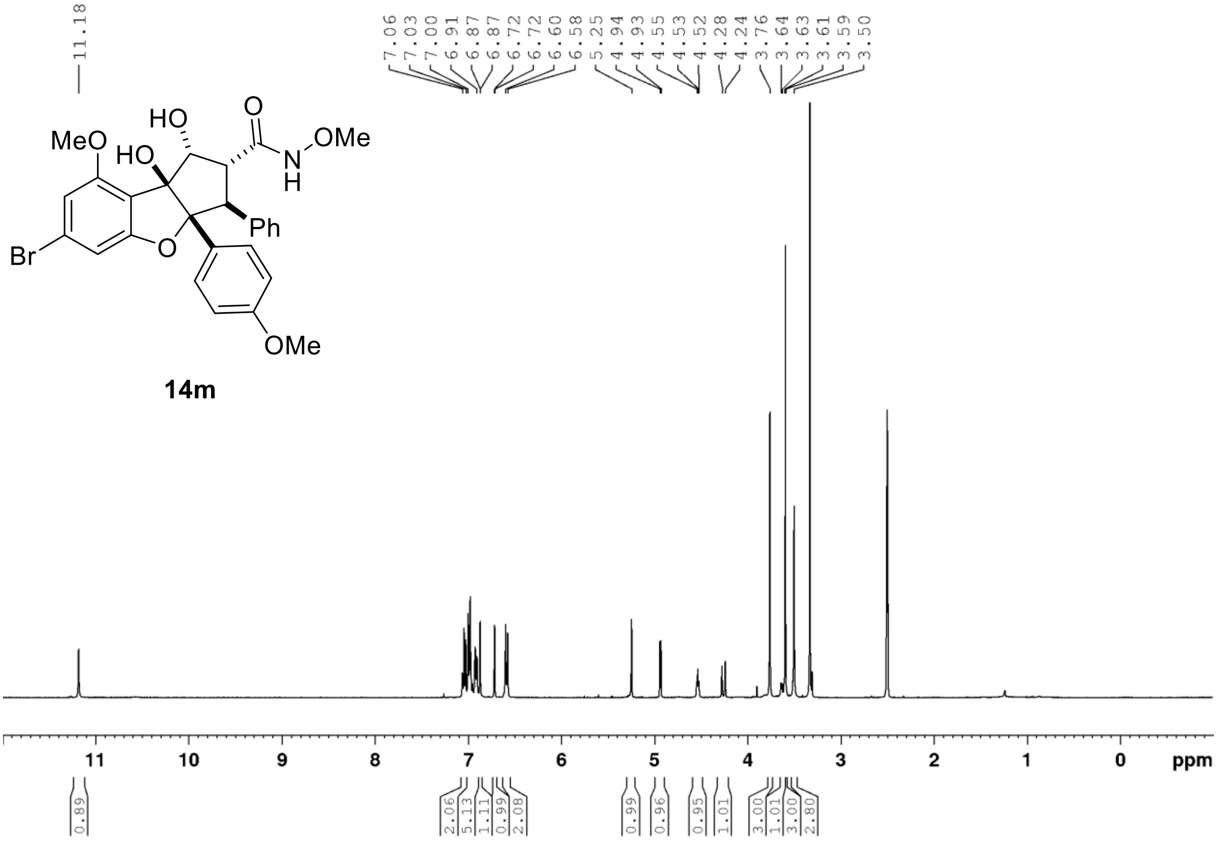

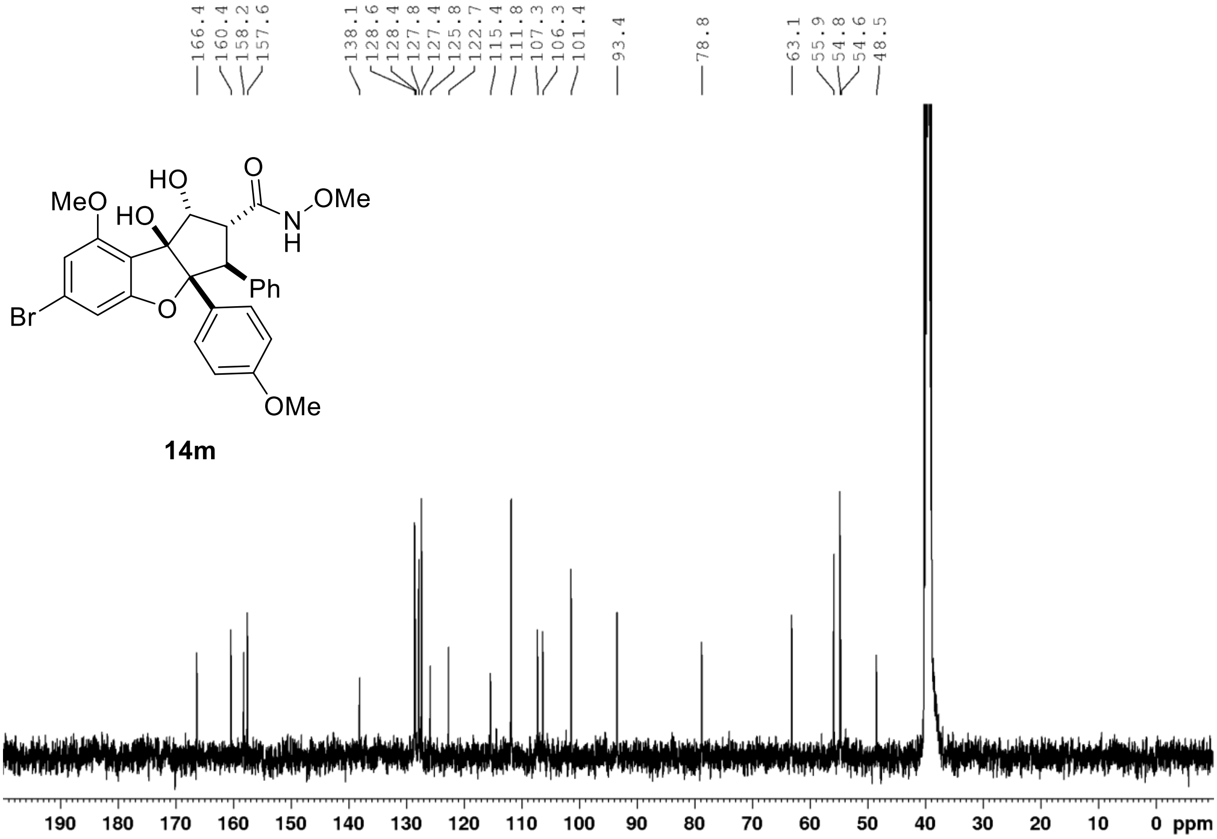

5. HPLC traces for biologically tested compounds

5.1. HPLC trace of 9a

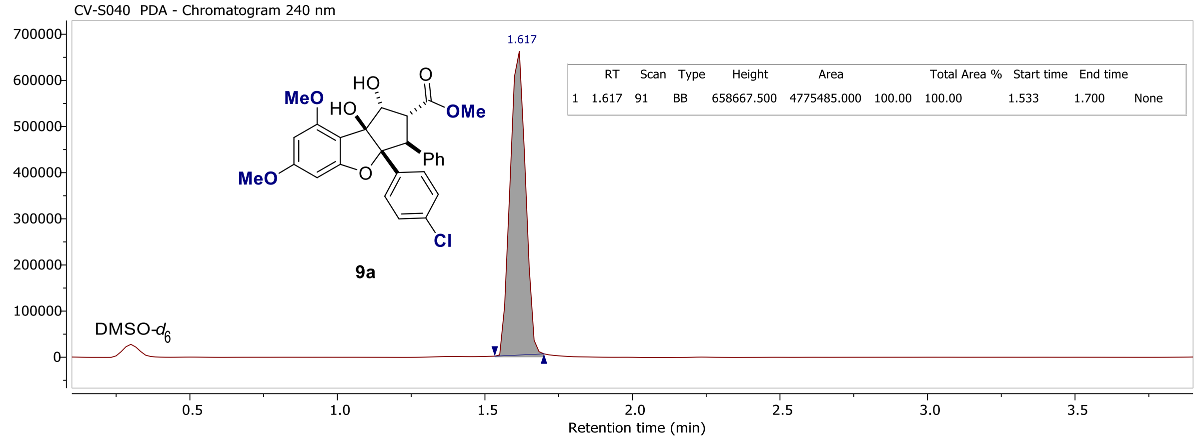

5.2. HPLC trace of 11ba

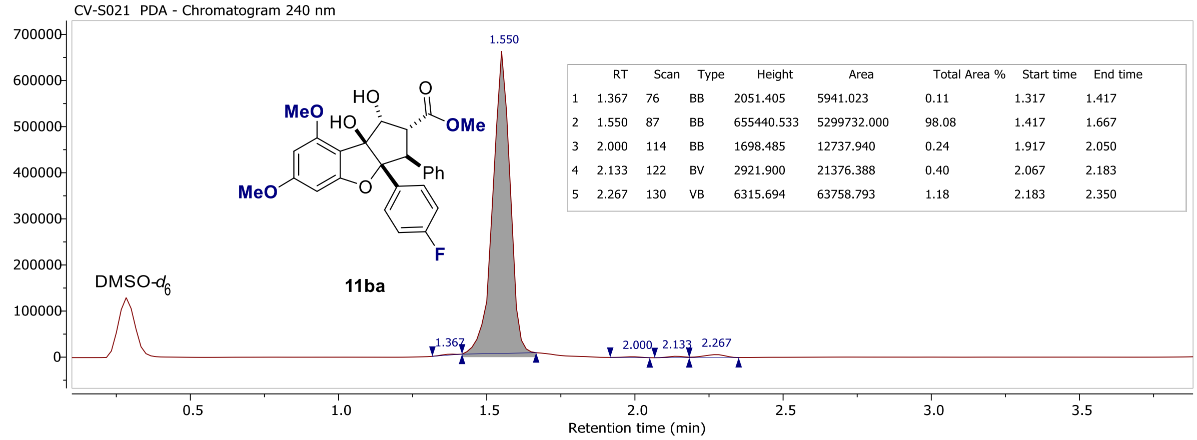

5.3. HPLC trace of 11bb

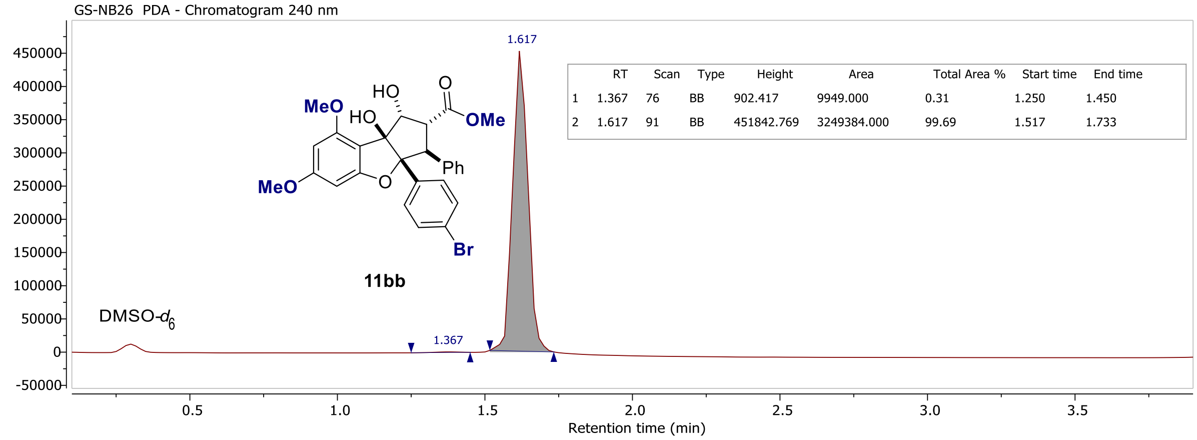

5.4. HPLC trace of 11bc

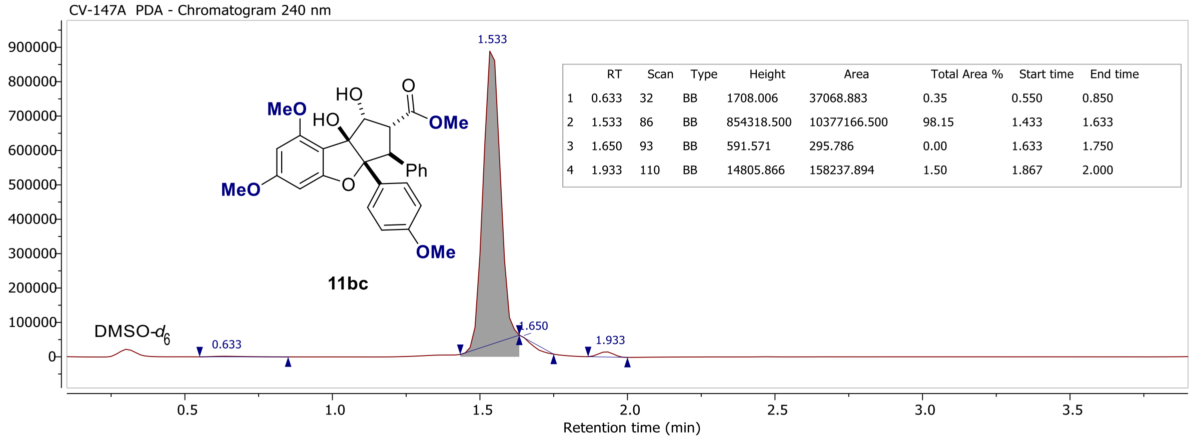

	RT	Scan	Type	Height	Area	Total Area %	Start time	End time
1	0.633	32	BB	1708.006	37068.883	0.35	0.550	0.850
2	1.533	86	BB	854318.500	10377166.500	98.15	1.433	1.633
3	1.650	93	BB	591.571	295.786	0.00	1.633	1.750
4	1.933	110	BB	14805.866	158237.894	1.50	1.867	2.000

5.5. HPLC trace of 9c

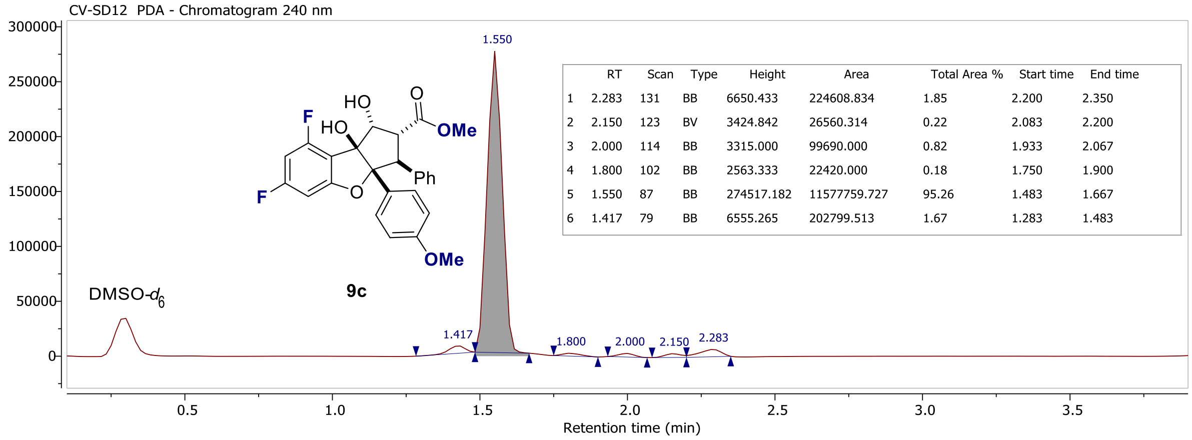

	RT	Scan	Type	Height	Area	Total Area %	Start time	End time
1	2.283	131	BB	6650.433	224608.834	1.85	2.200	2.350
2	2.150	123	BV	3424.842	26560.314	0.22	2.083	2.200
3	2.000	114	BB	3315.000	99690.000	0.82	1.933	2.067
4	1.800	102	BB	2563.333	22420.000	0.18	1.750	1.900
5	1.550	87	BB	274517.182	11577759.727	95.26	1.483	1.667
6	1.417	79	BB	6555.265	202799.513	1.67	1.283	1.483

5.6. HPLC trace of 9da

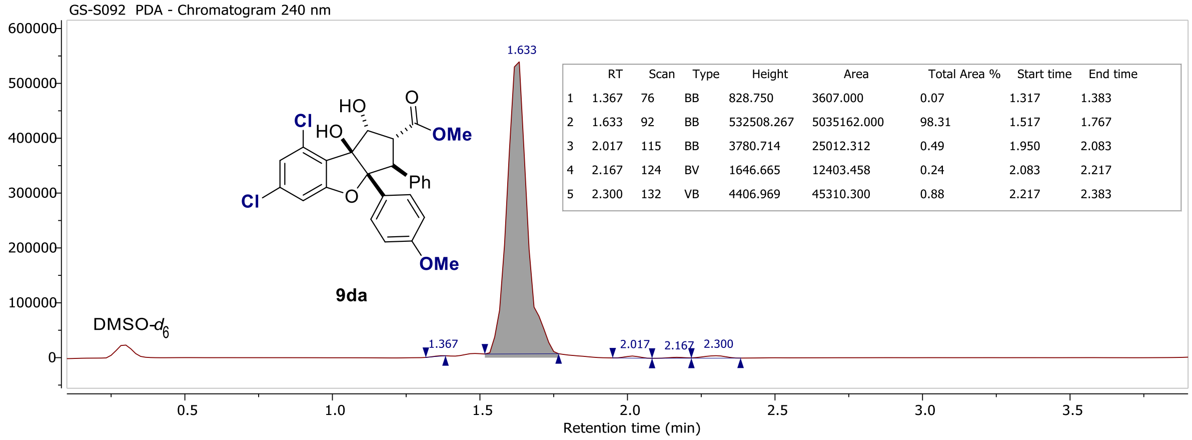

	RT	Scan	Type	Height	Area	Total Area %	Start time	End time
1	1.367	76	BB	828.750	3607.000	0.07	1.317	1.383
2	1.633	92	BB	532508.267	5035162.000	98.31	1.517	1.767
3	2.017	115	BB	3780.714	25012.312	0.49	1.950	2.083
4	2.167	124	BV	1646.665	12403.458	0.24	2.083	2.217
5	2.300	132	VB	4406.969	45310.300	0.88	2.217	2.383

5.7. HPLC trace of 9db

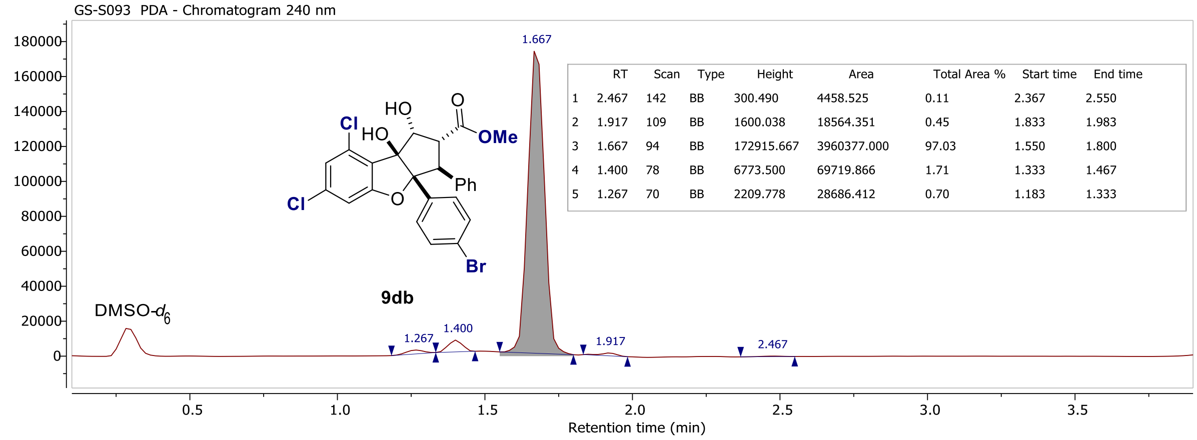

	RT	Scan	Type	Height	Area	Total Area %	Start time	End time
1	2.467	142	BB	300.490	4458.525	0.11	2.367	2.550
2	1.917	109	BB	1600.038	18564.351	0.45	1.833	1.983
3	1.667	94	BB	172915.667	3960377.000	97.03	1.550	1.800
4	1.400	78	BB	6773.500	69719.866	1.71	1.333	1.467
5	1.267	70	BB	2209.778	28686.412	0.70	1.183	1.333

5.8. HPLC trace of 9e

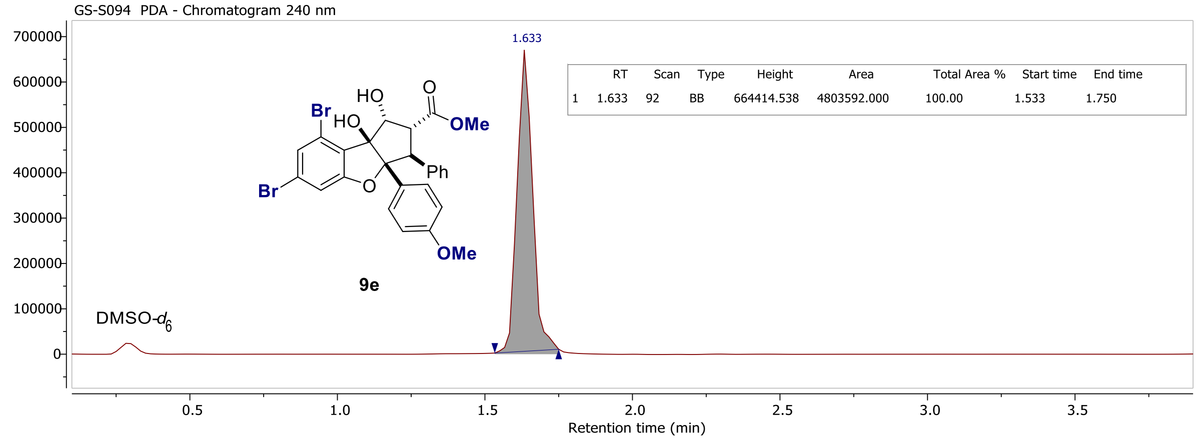

	RT	Scan	Type	Height	Area	Total Area %	Start time	End time
1	1.633	92	BB	664414.538	4803592.000	100.00	1.533	1.750

5.9. HPLC trace of 9f

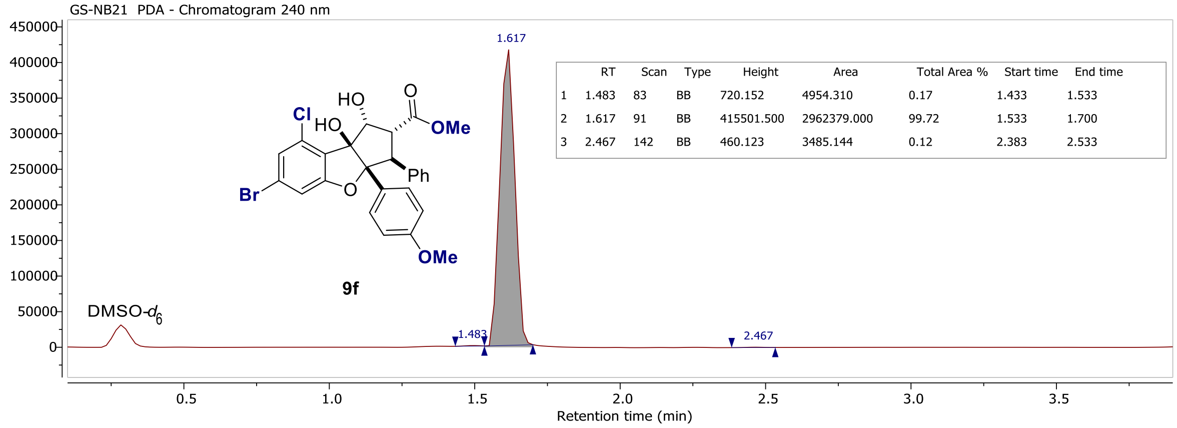

	RT	Scan	Type	Height	Area	Total Area %	Start time	End time
1	1.483	83	BB	720.152	4954.310	0.17	1.433	1.533
2	1.617	91	BB	415501.500	2962379.000	99.72	1.533	1.700
3	2.467	142	BB	460.123	3485.144	0.12	2.383	2.533

5.10. HPLC trace of 9g

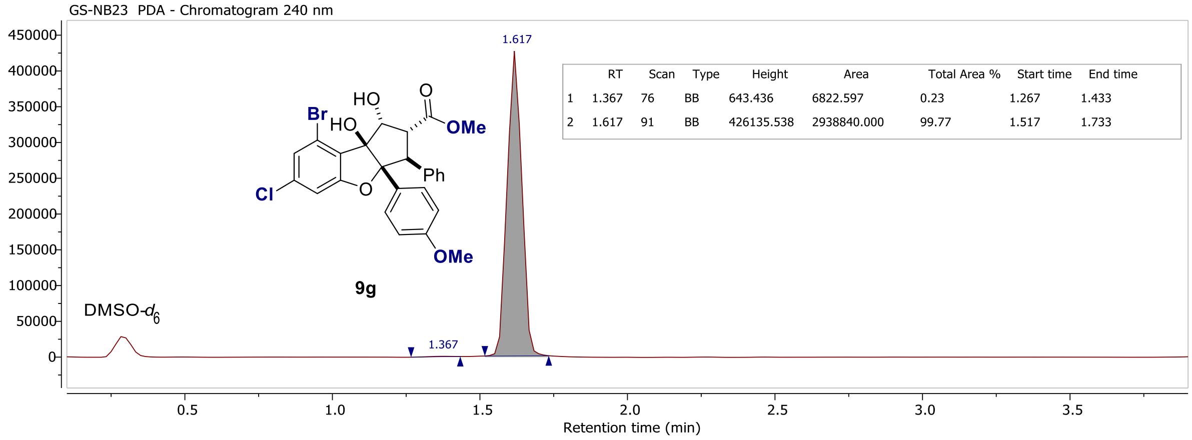

	RT	Scan	Type	Height	Area	Total Area %	Start time	End time
1	1.367	76	BB	643.436	6822.597	0.23	1.267	1.433
2	1.617	91	BB	426135.538	2938840.000	99.77	1.517	1.733

5.11. HPLC trace of 9h

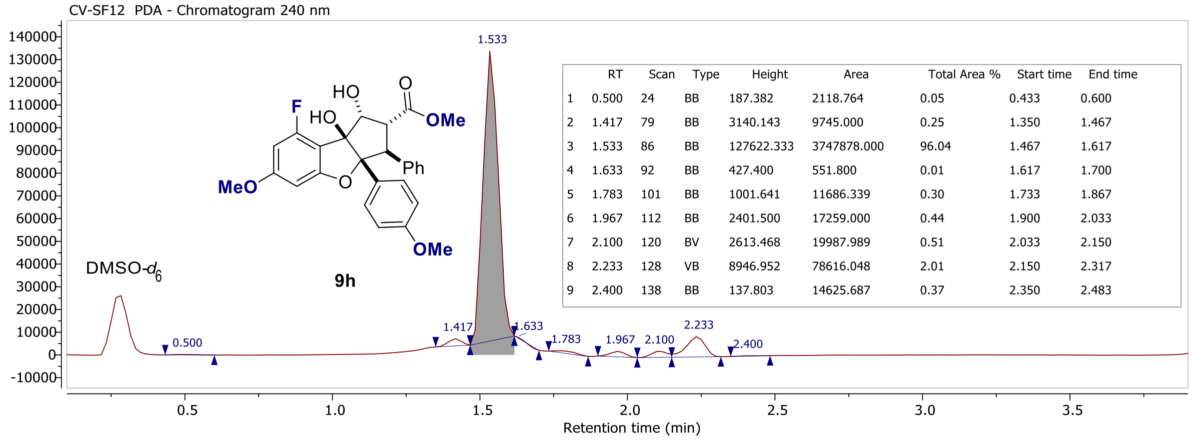

	RT	Scan	Type	Height	Area	Total Area %	Start time	End time
1	0.500	24	BB	187.382	2118.764	0.05	0.433	0.600
2	1.417	79	BB	3140.143	9745.000	0.25	1.350	1.467
3	1.533	86	BB	127622.333	3747878.000	96.04	1.467	1.617
4	1.633	92	BB	427.400	551.800	0.01	1.617	1.700
5	1.783	101	BB	1001.641	11686.339	0.30	1.733	1.867
6	1.967	112	BB	2401.500	17259.000	0.44	1.900	2.033
7	2.100	120	BV	2613.468	19987.989	0.51	2.033	2.150
8	2.233	128	VB	8946.952	78616.048	2.01	2.150	2.317
9	2.400	138	BB	137.803	14625.687	0.37	2.350	2.483

5.12. HPLC trace of 9i

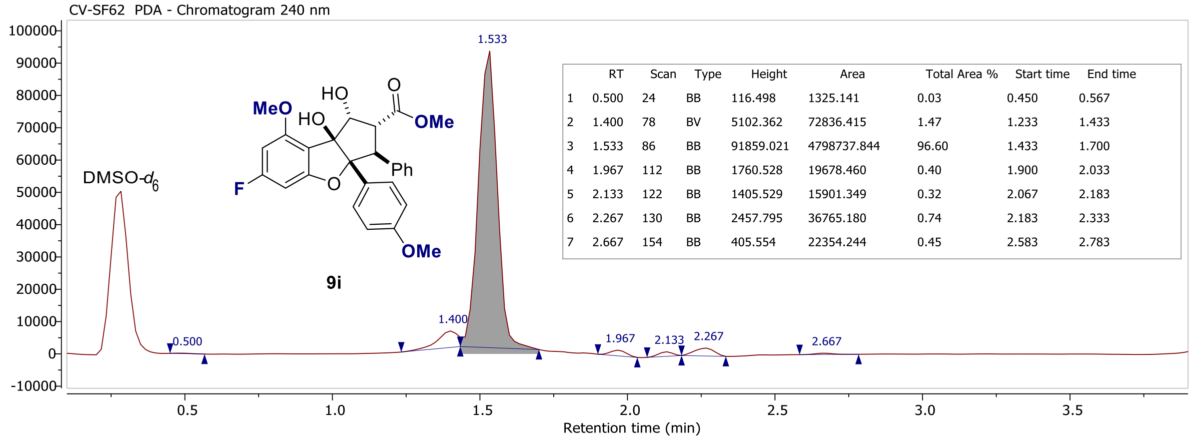

	RT	Scan	Type	Height	Area	Total Area %	Start time	End time
1	0.500	24	BB	116.498	1325.141	0.03	0.450	0.567
2	1.400	78	BV	5102.362	72836.415	1.47	1.233	1.433
3	1.533	86	BB	91859.021	4798737.844	96.60	1.433	1.700
4	1.967	112	BB	1760.528	19678.460	0.40	1.900	2.033
5	2.133	122	BB	1405.529	15901.349	0.32	2.067	2.183
6	2.267	130	BB	2457.795	36765.180	0.74	2.183	2.333
7	2.667	154	BB	405.554	22354.244	0.45	2.583	2.783

5.13. HPLC trace of 9j

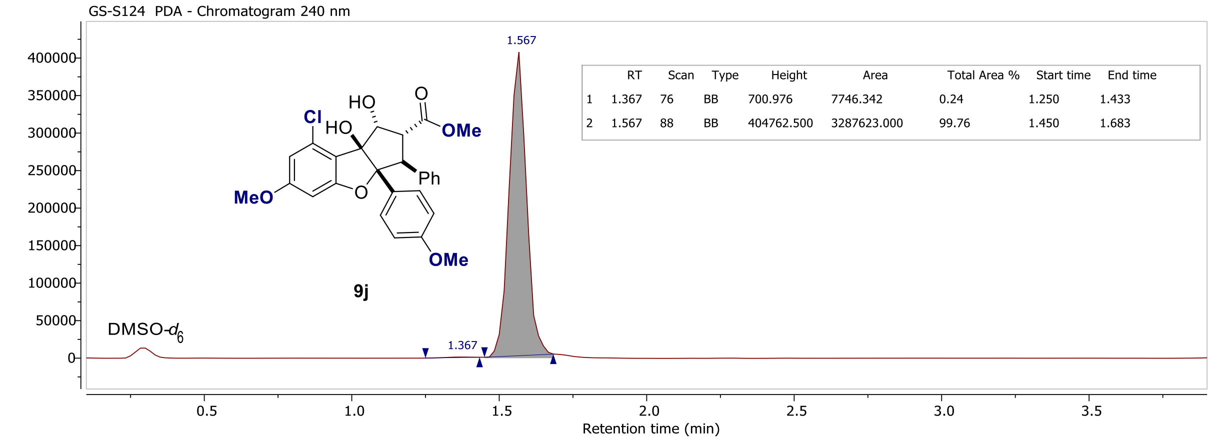

	RT	Scan	Type	Height	Area	Total Area %	Start time	End time
1	1.367	76	BB	700.976	7746.342	0.24	1.250	1.433
2	1.567	88	BB	404762.500	3287623.000	99.76	1.450	1.683

5.14. HPLC trace of 9k

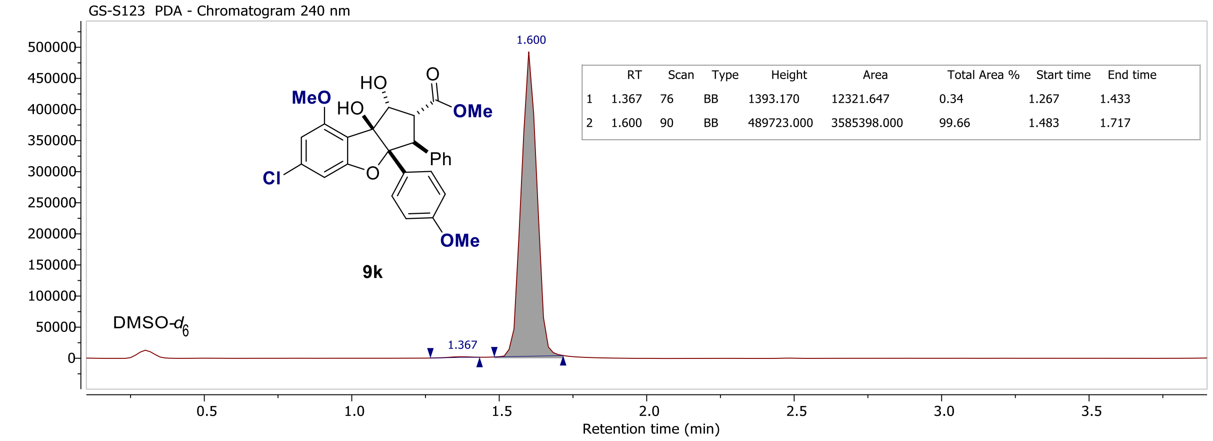

	RT	Scan	Type	Height	Area	Total Area %	Start time	End time
1	1.367	76	BB	1393.170	12321.647	0.34	1.267	1.433
2	1.600	90	BB	489723.000	3585398.000	99.66	1.483	1.717

5.15. HPLC trace of 9l

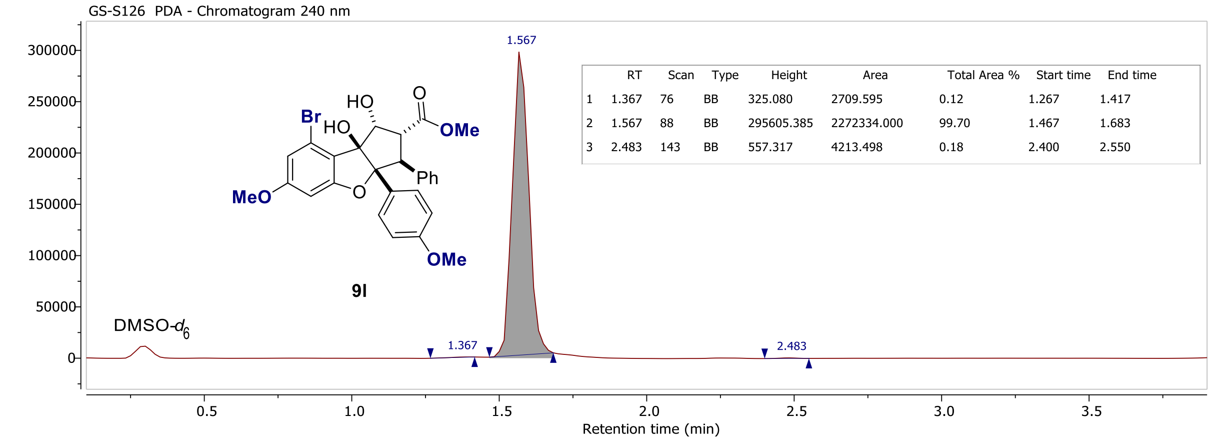

	RT	Scan	Type	Height	Area	Total Area %	Start time	End time
1	1.367	76	BB	325.080	2709.595	0.12	1.267	1.417
2	1.567	88	BB	295605.385	2272334.000	99.70	1.467	1.683
3	2.483	143	BB	557.317	4213.498	0.18	2.400	2.550

5.16. HPLC trace of 9m

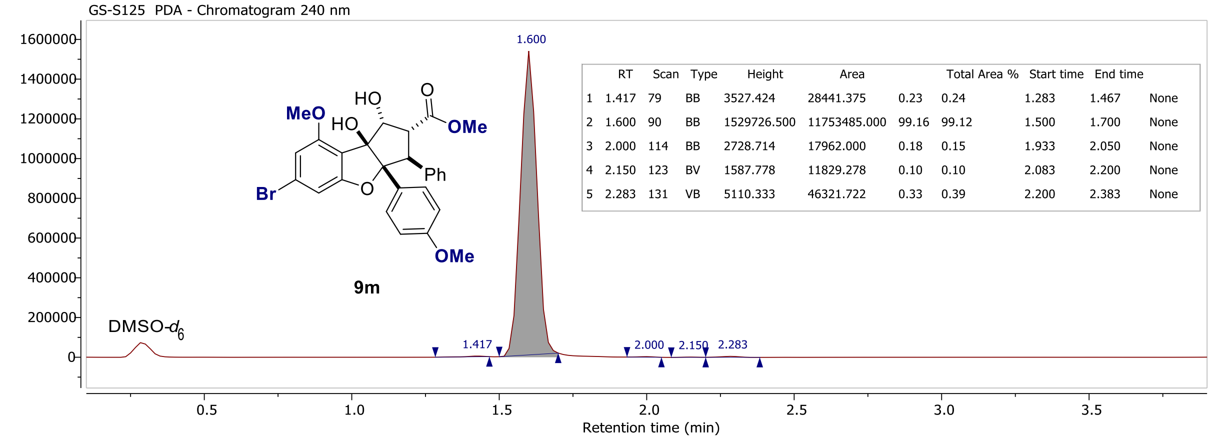

	RT	Scan	Type	Height	Area		Total Area %	Start time	End time	
1	1.417	79	BB	3527.424	28441.375	0.23	0.24	1.283	1.467	None
2	1.600	90	BB	1529726.500	11753485.000	99.16	99.12	1.500	1.700	None
3	2.000	114	BB	2728.714	17962.000	0.18	0.15	1.933	2.050	None
4	2.150	123	BV	1587.778	11829.278	0.10	0.10	2.083	2.200	None
5	2.283	131	VB	5110.333	46321.722	0.33	0.39	2.200	2.383	None

5.17. HPLC trace of 9na

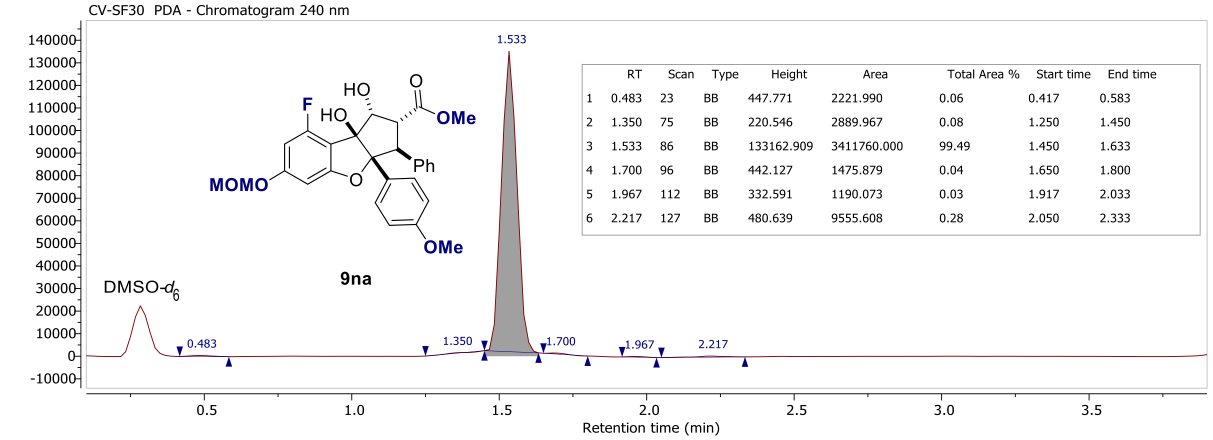

	RT	Scan	Type	Height	Area	Total Area %	Start time	End time
1	0.483	23	BB	447.771	2221.990	0.06	0.417	0.583
2	1.350	75	BB	220.546	2889.967	0.08	1.250	1.450
3	1.533	86	BB	133162.909	3411760.000	99.49	1.450	1.633
4	1.700	96	BB	442.127	1475.879	0.04	1.650	1.800
5	1.967	112	BB	332.591	1190.073	0.03	1.917	2.033
6	2.217	127	BB	480.639	9555.608	0.28	2.050	2.333

5.18. HPLC trace of 9nb

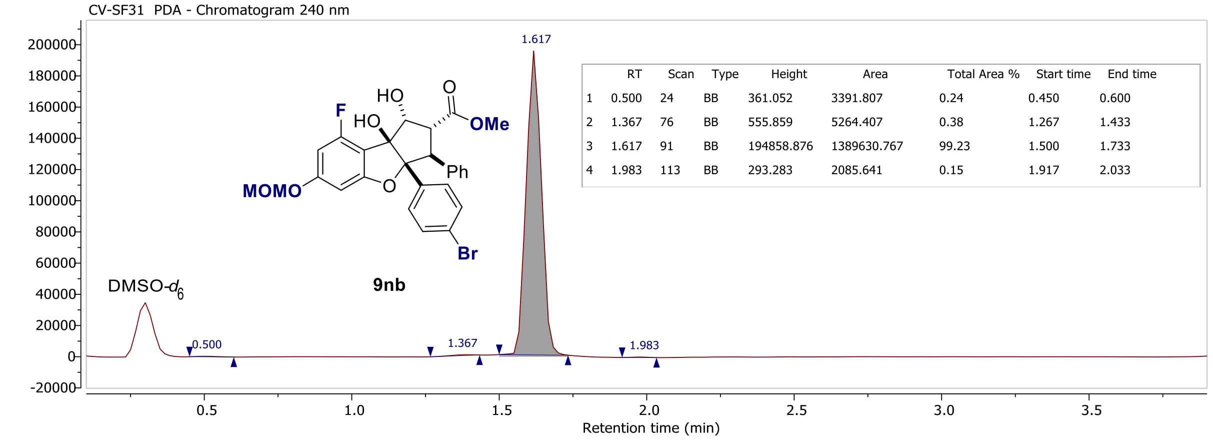

	RT	Scan	Type	Height	Area	Total Area %	Start time	End time
1	0.500	24	BB	361.052	3391.807	0.24	0.450	0.600
2	1.367	76	BB	555.859	5264.407	0.38	1.267	1.433
3	1.617	91	BB	194858.876	1389630.767	99.23	1.500	1.733
4	1.983	113	BB	293.283	2085.641	0.15	1.917	2.033

5.19. HPLC trace of 14aa

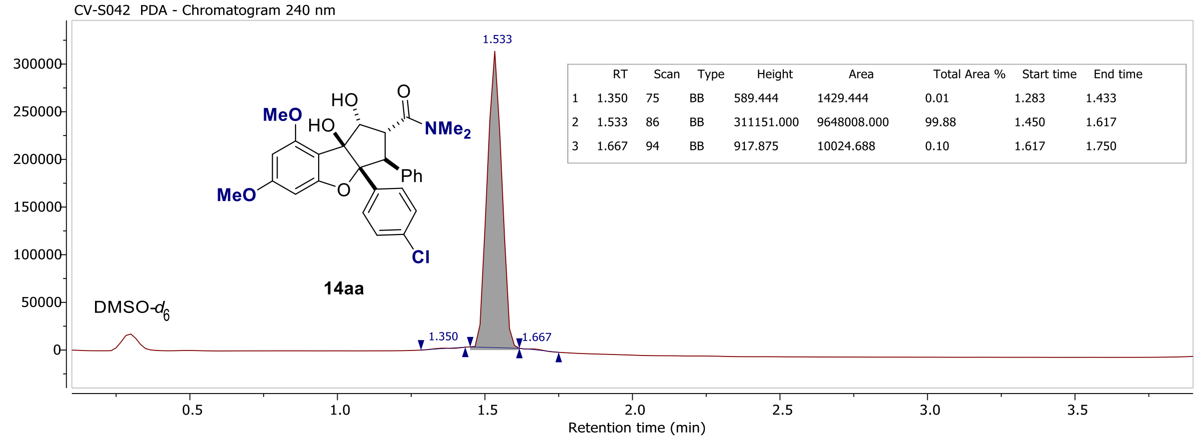

	RT	Scan	Type	Height	Area	Total Area %	Start time	End time
1	1.350	75	BB	589.444	1429.444	0.01	1.283	1.433
2	1.533	86	BB	311151.000	9648008.000	99.88	1.450	1.617
3	1.667	94	BB	917.875	10024.688	0.10	1.617	1.750

5.20. HPLC trace of 14ab

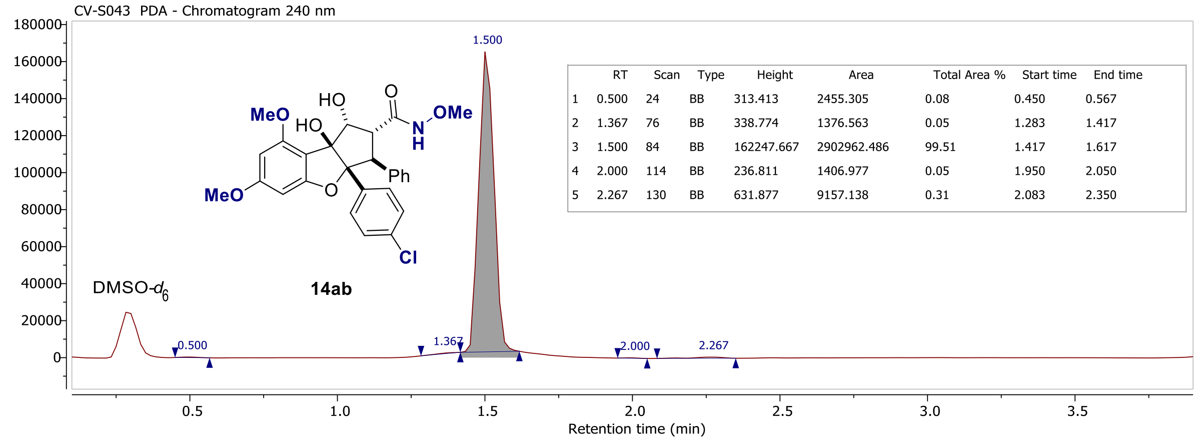

	RT	Scan	Type	Height	Area	Total Area %	Start time	End time
1	0.500	24	BB	313.413	2455.305	0.08	0.450	0.567
2	1.367	76	BB	338.774	1376.563	0.05	1.283	1.417
3	1.500	84	BB	162247.667	2902962.486	99.51	1.417	1.617
4	2.000	114	BB	236.811	1406.977	0.05	1.950	2.050
5	2.267	130	BB	631.877	9157.138	0.31	2.083	2.350

5.21. HPLC trace of 14baa

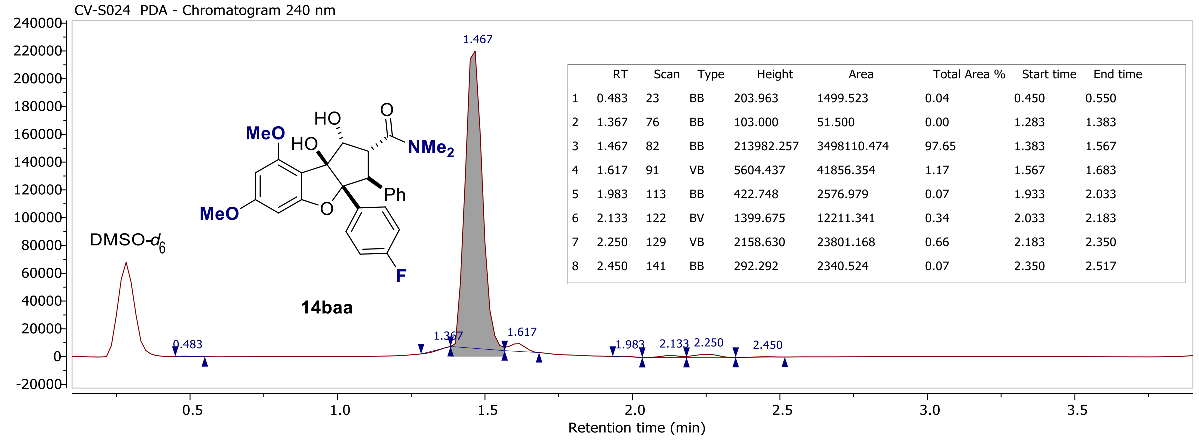

	RT	Scan	Type	Height	Area	Total Area %	Start time	End time
1	0.483	23	BB	203.963	1499.523	0.04	0.450	0.550
2	1.367	76	BB	103.000	51.500	0.00	1.283	1.383
3	1.467	82	BB	213982.257	3498110.474	97.65	1.383	1.567
4	1.617	91	VB	5604.437	41856.354	1.17	1.567	1.683
5	1.983	113	BB	422.748	2576.979	0.07	1.933	2.033
6	2.133	122	BV	1399.675	12211.341	0.34	2.033	2.183
7	2.250	129	VB	2158.630	23801.168	0.66	2.183	2.350
8	2.450	141	BB	292.292	2340.524	0.07	2.350	2.517

5.22. HPLC trace of 14bab

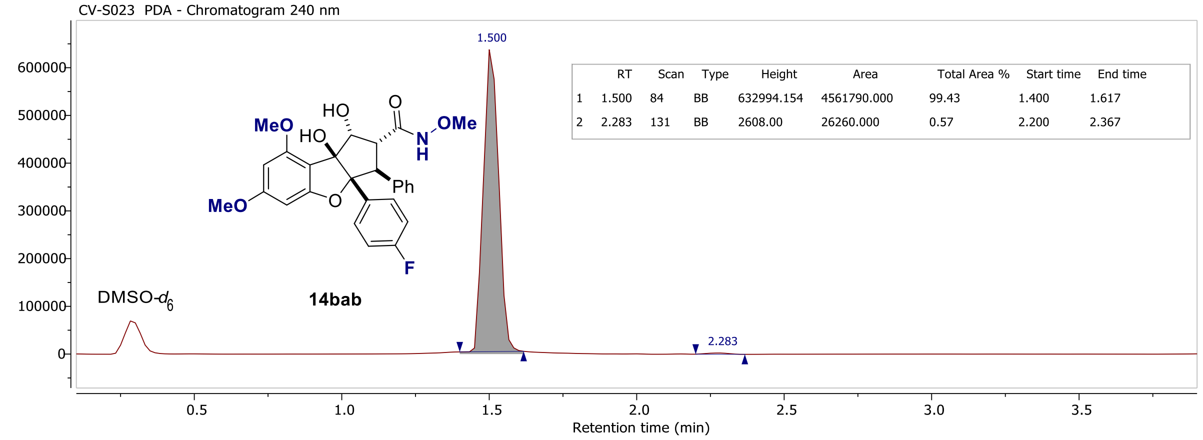

	RT	Scan	Type	Height	Area	Total Area %	Start time	End time
1	1.500	84	BB	632994.154	4561790.000	99.43	1.400	1.617
2	2.283	131	BB	2608.00	26260.000	0.57	2.200	2.367

5.23. HPLC trace of *rac*-1b

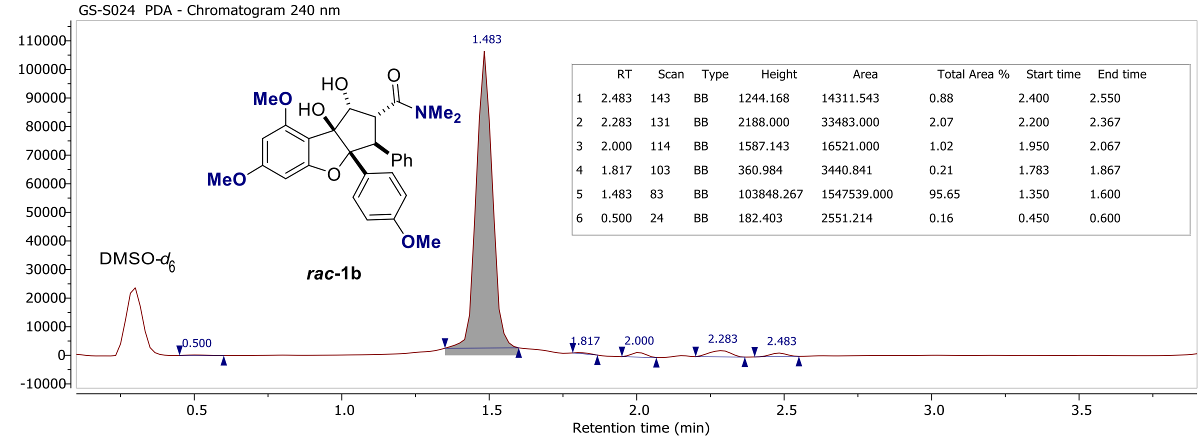

	RT	Scan	Type	Height	Area	Total Area %	Start time	End time
1	2.483	143	BB	1244.168	14311.543	0.88	2.400	2.550
2	2.283	131	BB	2188.000	33483.000	2.07	2.200	2.367
3	2.000	114	BB	1587.143	16521.000	1.02	1.950	2.067
4	1.817	103	BB	360.984	3440.841	0.21	1.783	1.867
5	1.483	83	BB	103848.267	1547539.000	95.65	1.350	1.600
6	0.500	24	BB	182.403	2551.214	0.16	0.450	0.600

5.24. HPLC trace of *rac*-1C

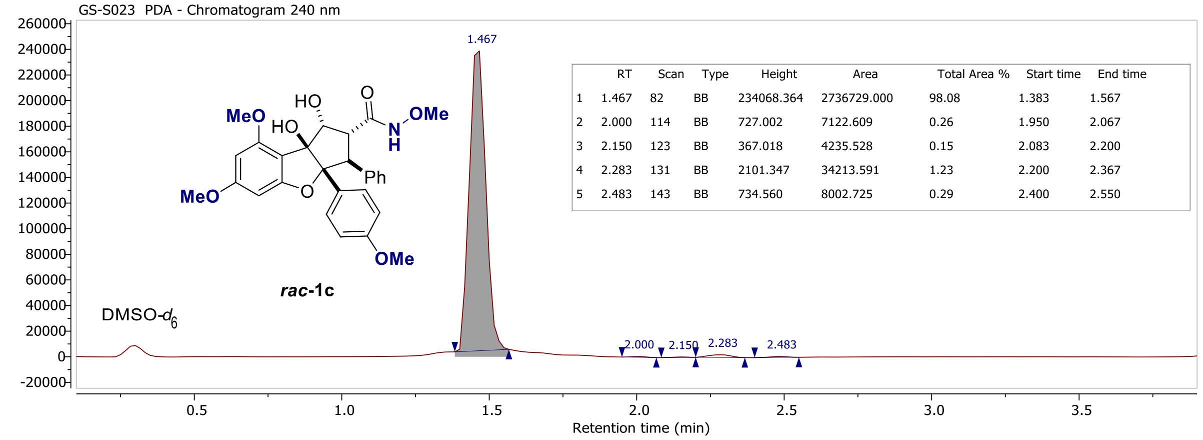

	RT	Scan	Type	Height	Area	Total Area %	Start time	End time
1	1.467	82	BB	234068.364	2736729.000	98.08	1.383	1.567
2	2.000	114	BB	727.002	7122.609	0.26	1.950	2.067
3	2.150	123	BB	367.018	4235.528	0.15	2.083	2.200
4	2.283	131	BB	2101.347	34213.591	1.23	2.200	2.367
5	2.483	143	BB	734.560	8002.725	0.29	2.400	2.550

5.25. HPLC trace of 14da

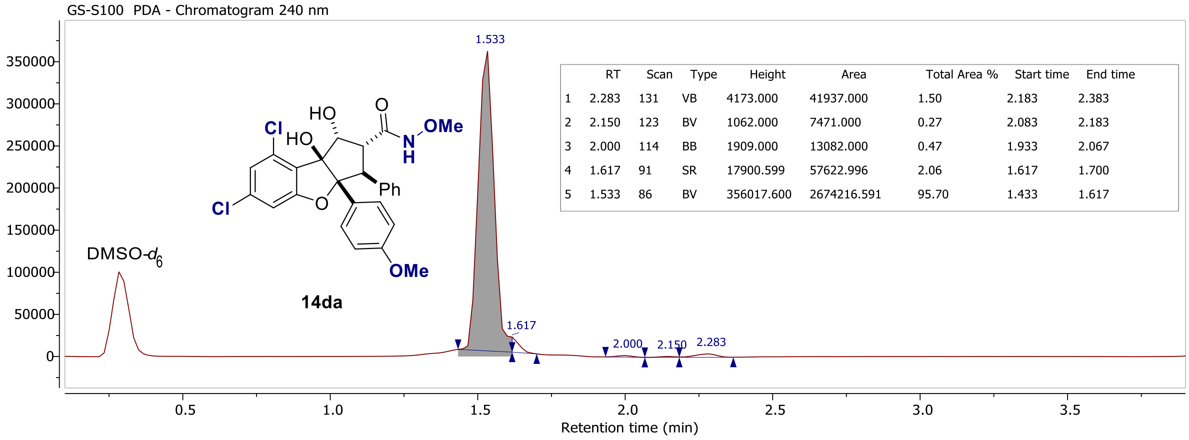

	RT	Scan	Type	Height	Area	Total Area %	Start time	End time
1	2.283	131	VB	4173.000	41937.000	1.50	2.183	2.383
2	2.150	123	BV	1062.000	7471.000	0.27	2.083	2.183
3	2.000	114	BB	1909.000	13082.000	0.47	1.933	2.067
4	1.617	91	SR	17900.599	57622.996	2.06	1.617	1.700
5	1.533	86	BV	356017.600	2674216.591	95.70	1.433	1.617

5.26. HPLC trace of 14f

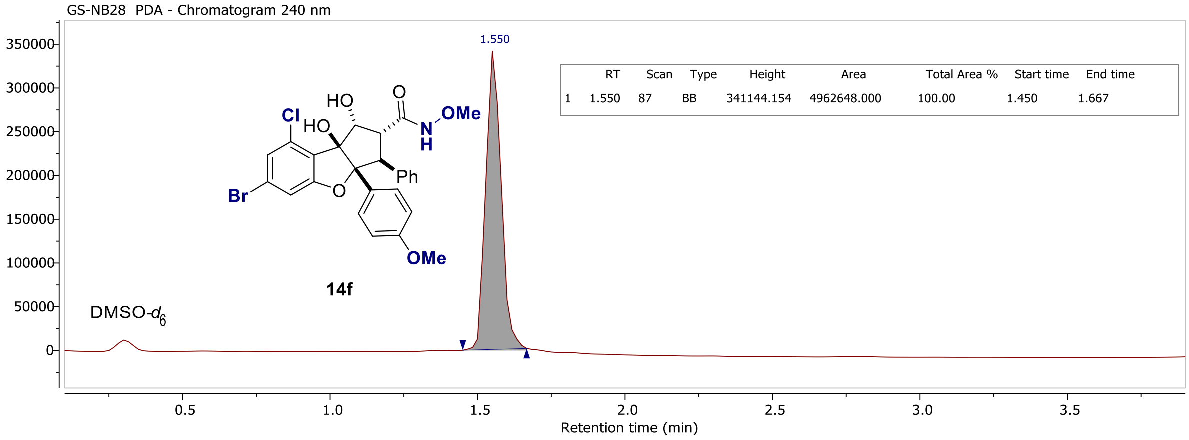

	RT	Scan	Type	Height	Area	Total Area %	Start time	End time
1	1.550	87	BB	341144.154	4962648.000	100.00	1.450	1.667

5.27. HPLC trace of 14g

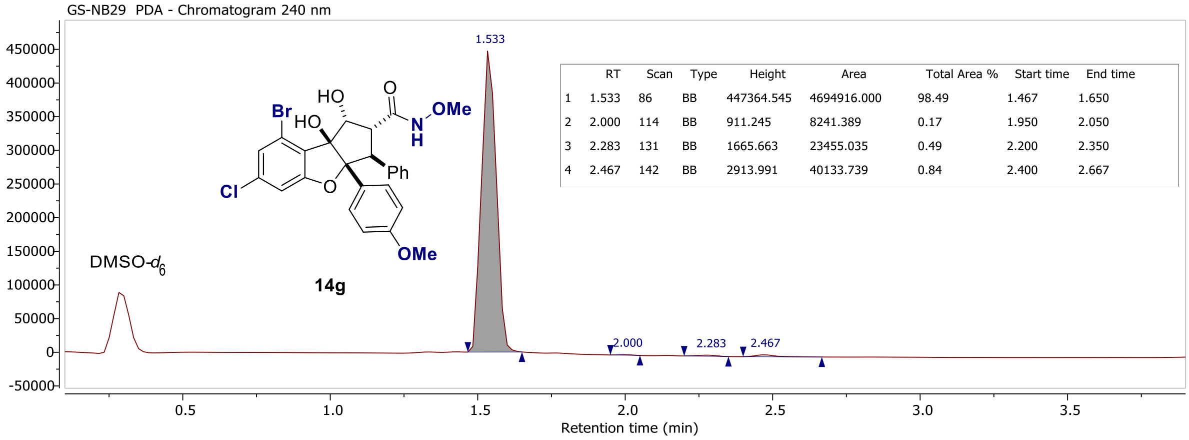

	RT	Scan	Type	Height	Area	Total Area %	Start time	End time
1	1.533	86	BB	447364.545	4694916.000	98.49	1.467	1.650
2	2.000	114	BB	911.245	8241.389	0.17	1.950	2.050
3	2.283	131	BB	1665.663	23455.035	0.49	2.200	2.350
4	2.467	142	BB	2913.991	40133.739	0.84	2.400	2.667

5.28. HPLC trace of 14ha

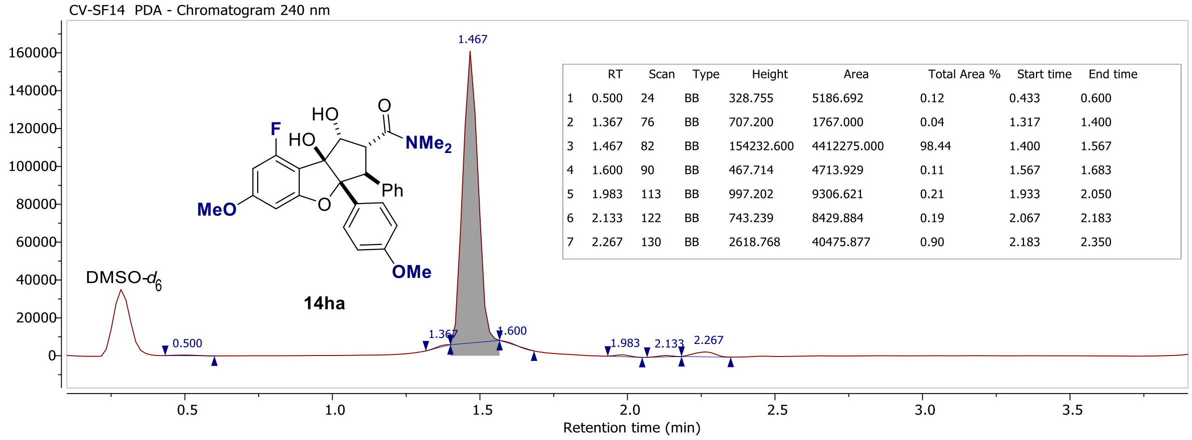

	RT	Scan	Type	Height	Area	Total Area %	Start time	End time
1	0.500	24	BB	328.755	5186.692	0.12	0.433	0.600
2	1.367	76	BB	707.200	1767.000	0.04	1.317	1.400
3	1.467	82	BB	154232.600	4412275.000	98.44	1.400	1.567
4	1.600	90	BB	467.714	4713.929	0.11	1.567	1.683
5	1.983	113	BB	997.202	9306.621	0.21	1.933	2.050
6	2.133	122	BB	743.239	8429.884	0.19	2.067	2.183
7	2.267	130	BB	2618.768	40475.877	0.90	2.183	2.350

5.29. HPLC trace of 14hb

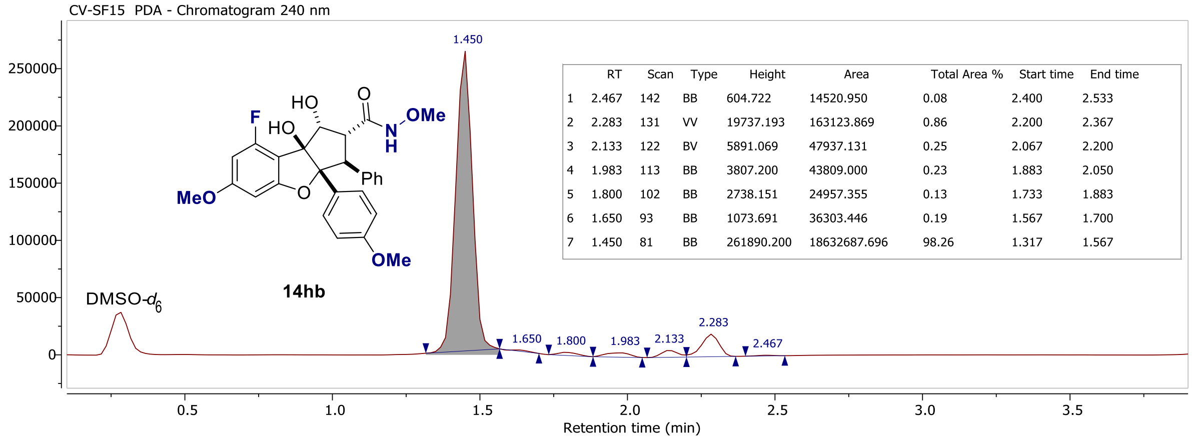

	RT	Scan	Type	Height	Area	Total Area %	Start time	End time
1	2.467	142	BB	604.722	14520.950	0.08	2.400	2.533
2	2.283	131	VV	19737.193	163123.869	0.86	2.200	2.367
3	2.133	122	BV	5891.069	47937.131	0.25	2.067	2.200
4	1.983	113	BB	3807.200	43809.000	0.23	1.883	2.050
5	1.800	102	BB	2738.151	24957.355	0.13	1.733	1.883
6	1.650	93	BB	1073.691	36303.446	0.19	1.567	1.700
7	1.450	81	BB	261890.200	18632687.696	98.26	1.317	1.567

5.30. HPLC trace of 14m

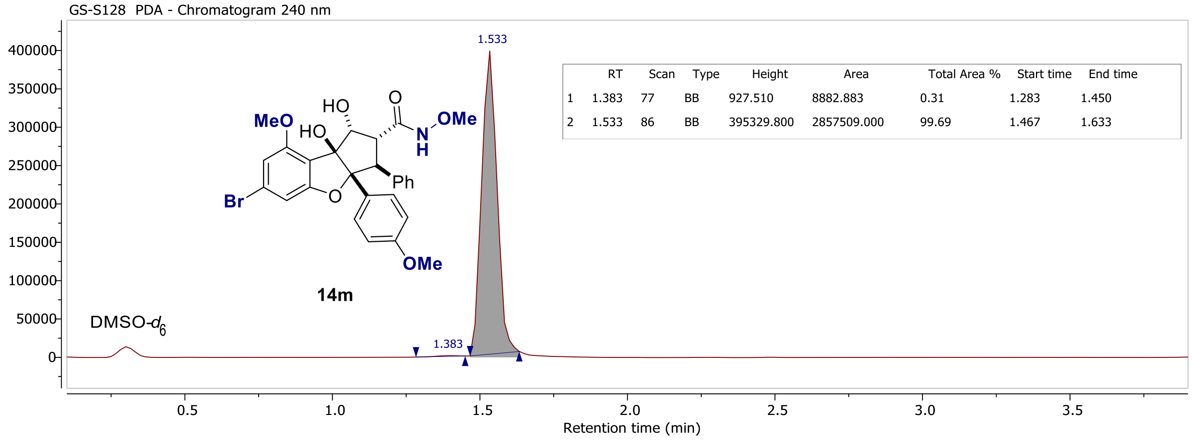

	RT	Scan	Type	Height	Area	Total Area %	Start time	End time
1	1.383	77	BB	927.510	8882.883	0.31	1.283	1.450
2	1.533	86	BB	395329.800	2857509.000	99.69	1.467	1.633

6. Supplementary Figures

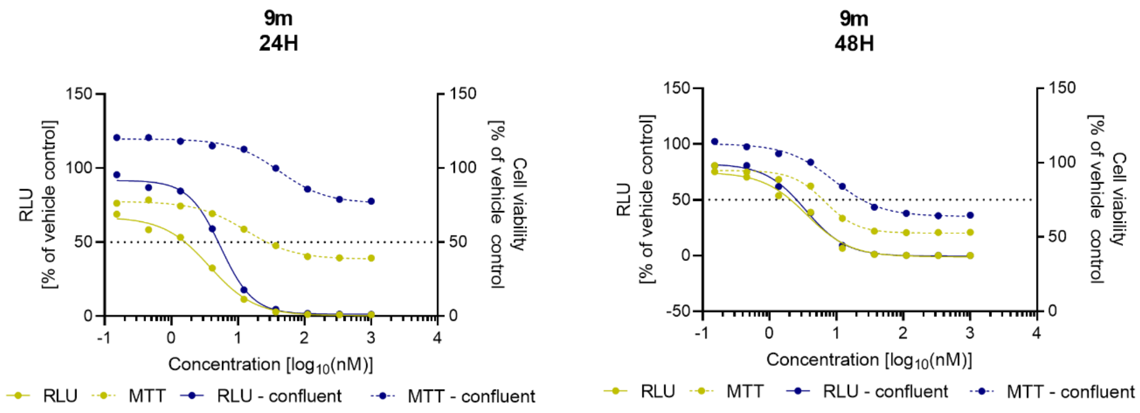

Figure S1: Antiviral efficacy of halogenated rocaglates against HEV in confluent cells. HEV subgenomic replicon HEVp6-Gluc was electroporated into non-confluent or confluent HepG2 cells. Cells were treated with **9m** at concentrations ranging from 0.15 nM to 1000 nM for 24 h and 48 h. Depicted are non-linear fit response curves representative for confluent (dark blue lines), non-confluent (yellow lines), and cell viability was monitored by MTT assay (respective colored dashed lines). Error bars indicate standard deviation of three technical replicates.

7. References

(S1) Pandey, G.; Tiwari, S. K.; Singh, B.; Vanka, K.; Jain, S. p-Selective (sp2)-C-H functionalization for an acylation/alkylation reaction using organic photoredox catalysis. *Chem. Commun.* **2017**, *53*, 12337–12340.

(S2) Grayfer, T. D.; Grellier, P.; Mouray, E.; Dodd, R. H.; Dubois, J.; Cariou, K. Mallotojaponins B and C: Total Synthesis, Antiparasitic Evaluation, and Preliminary SAR Studies. *Org. Lett.* **2016**, *18*, 708–711.

(S3) Wei, Y.; Tang, J.; Cong, X.; Zeng, X. Practical metal-free synthesis of chalcone derivatives via a tandem cross-dehydrogenative-coupling/elimination reaction. *Green Chem.* **2013**, *15*, 3165.

(S4) Liu, T.; Nair, S. J.; Lescarbeau, A.; Belani, J.; Peluso, S.; Conley, J.; Tillotson, B.; O'Hearn, P.; Smith, S.; Slocum, K.; West, K.; Helble, J.; Douglas, M.; Bahadoor, A.; Ali, J.; McGovern, K.; Fritz, C.; Palombella, V. J.; Wylie, A.; Castro, A. C.; Tremblay, M. R. Synthetic silvestrol analogues as potent and selective protein synthesis inhibitors. *J. Med. Chem.* **2012**, *55*, 8859–8878.

(S5) Britton, R. G.; Horner-Glister, E.; Pomenya, O. A.; Smith, E. E.; Denton, R.; Jenkins, P. R.; Steward, W. P.; Brown, K.; Gescher, A.; Sale, S. Synthesis and biological evaluation of novel flavonols as potential anti-prostate cancer agents. *Eur. J. Med. Chem.* **2012**, *54*, 952–958.

(S6) Zhao, X.; Liu, J.; Xie, Z.; Li, Y. A One-Pot Synthesis of Aurones from Substituted Acetophenones and Benzaldehydes: A Concise Synthesis of Aureusidin. *Synthesis* **2012**, *44*, 2217–2224.

(S7) Buckman, B. O.; Nicholas, J. B.; Emayan, K.; Seiwert, S. D. Lysophosphatidic acid receptor antagonist, WO2013025733 (A1), 2013.

(S8) Cai, X.; Qian, C.; Wang, Y. D. 3,5- disubstituted pyrazoles useful as checkpoint kinase 1 (CHK1) inhibitors, and their preparations and applications, WO2017132928 (A1), 2017.

(S9) Weber, M.; Owens, K.; Masarwa, A.; Sarpong, R. Construction of Enantiopure Taxoid and Natural Product-like Scaffolds Using a C-C Bond Cleavage/Arylation Reaction. *Org. Lett.* **2015**, *17*, 5432–5435.
